# What happens to the osteoporotic bone mesenchymal stem cells? Evidence from RNA sequencing

**DOI:** 10.7150/ijms.88146

**Published:** 2024-01-01

**Authors:** Mingyang Li, Rong Cong, Huadong Wang, Chao Ma, Yongwei Lv, Yang Zheng, Yantao Zhao, Qin Fu, Li Li

**Affiliations:** 1 Senior Department of Orthopedics, the Fourth Medical Center of PLA General Hospital, Beijing, China.; 2 Senior Department of Obstetrics & Gynecology, the Seventh Medical Center of PLA General Hospital, Beijing, China.; 3 Department of Orthopedics, Shengjing Hospital of China Medical University, Shenyang, China.; 4 Beijing Engineering Research Center of Orthopedics Implants, Beijing, China.

**Keywords:** bone mesenchymal stem cell, osteoporosis, RNA sequencing, mRNA, miRNA, lncRNA

## Abstract

Evidence presented that osteoporosis is closely related to the dysfunction of bone mesenchymal stem cells (BMSCs). But most studies are insufficient to reveal what actually happens to the osteoporotic BMSCs. In this study, BMSCs were harvested from ovariectomized and sham-operated rats. After checking the characteristics of rat models and stem cells, the BMSCs were carried out for RNA sequencing. Part of the findings were verified that seven mRNAs (Abi3bp, Aifm3, Ccl11, Cdkn1c, Chst10, Id2, Vcam1) were significantly up-regulated in osteoporotic BMSCs while seven mRNAs (Cep63, Fgfr3, Myc, Omd, Pou2f1, Smarcal1, Timm10b) were down-regulated. In addition, potential miRNA-mRNA and lncRNA-mRNA regulatory networks were illustrated. The changes in osteoporotic BMSCs covered a large set of biological processes, including cell viability, differentiation, immunoreaction, bone repairment and estrogen defect. This study enriched the pathophysiological mechanisms of BMSCs and osteporosis, as well as provided dozens of attractive RNA targets for further treatment.

## Introduction

Osteoporosis (OP) is a common skeletal metabolic disease hallmarked by the loss of bone mass and the deterioration of bone microarchitecture, as the result of systemic imbalance between bone creation and resorption^1^. With the increasing of worldwide aging population, osteoporosis poses great medical and financial burden to the whole society. It's estimated that patients suffering from osteoporosis might increase to 300 million globally by 2023^2^. In particular, postmenopausal women are taking at a higher risk due to the deficit of estrogen which has great influence on the bone turnover. Antiresorptive drugs, anabolic drugs, and combination or sequential therapies are the most common treatments for osteoporosis, but it is worth noting that some frustrating adverse effects are limiting the drug safety for long-term use, so considerable efforts should be made to improve the existing therapies^3^.

Evidence has been presented that senile osteoporosis (SOP) and postmenopausal osteoporosis (PMOP) are partly caused by the dysfunction of bone mesenchymal stem cells (BMSCs), which decline in numbers as well as preferentially differentiate into adipocytes rather than osteoblasts^4^. BMSCs are thought to be vital sources of osteoblasts and maintainers of bone homeostasis. In view of their outstanding characteristics, including self-renewal ability, multipotency, low immunogenicity, homing ability and secretory ability, BMSCs are increasingly accepted as attractive seed cells in tissue regeneration to treat with osteoporosis, as well as other diseases^5^-^7^. Considering the enormous therapeutic potential of BMSCs, great efforts have been given to probe what happens to the osteoporotic BMSCs. However, the study is hampered due to the long time taken to establish osteoporosis models and the inevitable senescence of BMSCs during culture^8^. Although some scholars tried to study BMSCs with osteogenic reagents or some other treatments^9^, it might be insufficient to reflect the actualities happening in osteoporotic BMSCs.

The ovariectomized (OVX) rat model has been approved by the US Food and Drug Administration (FDA) to be an ideal model to illuminate PMOP^10^. In this study, we harvested BMSCs from OVX rats and sham-operated (SHAM) rats for further high-throughput RNA sequencing (RNA-seq). It's aimed to illuminate what actually happened to the osteoporotic BMSCs and help more scholars to elaborate the underlying mechanisms of BMSCs dysfunction and osteoporosis progression.

## Materials and methods

### Establishment of OVX and SHAM rat models

All the animal experiments were approved by the Institutional Review Committee of the Ethics Committee of Shengjing Hospital of China Medical University. A total of 24 specific pathogen-free Sprague Dawley rats (female, 8-week-old) were randomly divided into the OVX group (n=12) and the SHAM group (n=12). All the animals were housed under standard temperature and humidity, with a 12h-light/12h-dark cycle and free rodent diet. After one week of acclimatization, the OVX group rats underwent bilateral OVX through lumbodorsal vertical incision, while the SHAM group rats were resected similar sizes of adipose tissue around bilateral ovaries. 12 weeks later, the OVX and SHAM rat models were established.

### Confirmation of osteoporosis model

The OVX and SHAM rats were anesthetized and executed by excessive pentobarbital sodium following the operation after 12 weeks. Blood was taken from the abdominal aorta and centrifuged at 3,000 rpm for 15 min to collect serum. Serum ALP levels were qualified following the procedure of alkaline phosphatase kit (Solarbio, Beijing, China). Bilateral femurs were fixed in 4% paraformaldehyde solution for 48h and then decalcified in 10% EDTA solution for 1 month. Afterwards, the bones were dehydrated, permeabilized and embedded in paraffin and sliced in 5 um thickness. The slices were stained using Hematoxylin-Eosin Staining Kit (Solarbio, China) following the instructions. The staining was observed and recorded by the microscope (Eclipse, Nikon, Japan). The metaphysis of proximal tibias were measured by micro-CT (Y. Cheetah, YXLON, Germany) and analyzed by VG Studiomax 3.0 (Volume Graphics, Germany).

### Isolation and culture of BMSCs

Bilateral femurs and tibiae collected from OVX rats and SHAM rats were dissected free of soft tissue under aseptic conditions. Then both ends of the bones were removed with scissors, and a syringe needle was inserted into the medullary cavity. All the bone marrows were flushed out using Dulbecco's Modified Eagle Medium/Nutrient Mixture F-12 (DMEM/F12, Hyclone, UT, USA) supplemented with 10% fetal bovine serum (FBS; BioInd, Kibbutz Beit Haemek, Israel) and 1% penicillin-streptomycin solution (BioInd) into cell culture dishes. The marrow mixture provided suitable environment for BMSCs culture. The cells were incubated in 5% CO2 humidified atmosphere at 37°C. The culture medium was replaced every 2 days. When cells density reached 80%, adherent cells were digested by 0.25% trypsin (KeyGen, Nanjing, China) and passaged into new dishes.

### Identification of BMSCs

According to the guidelines announced by The International Society for Cellular Therapy, BMSCs should be identified in terms of morphology, immunophenotyping and multipotency (Clinical Application of Bone Marrow Mesenchymal Stem/Stromal Cells to Repair Skeletal Tissue).

Morphology: The primary-generation and the third-generation BMSCs were washed by phosphate-buffered saline (PBS) and observed by the microscope (Eclipse, Nikon, Japan). Immunophenotyping: The third-generation BMSCs were harvested with trypsin, centrifuged at 1,000 r/min for 5 min, and then washed and recentrifuged by PBS twice. The cells were counted and resuspended with PBS into four tubes (1x10^6^ cells/500ul). Fluorochrome conjugated mouse anti-rat antibodies (CD29-FITC, CD45-FITC, CD90-PE, BD Pharmingen, USA) and another equivalent PBS were mixed into individual tubes. The cells were incubated in the dark at 4°C for 30min and detected by flow cytometer (BD Biosciences, USA).

Multipotency: For osteogenic differentiation, the third-generation BMSCs were seeded into cell culture plates and cultured in high glucose DMEM (HG-DMEM) medium containing 10% FBS, 10nmol/l dexamethasone, 50mg/ml ascorbic acid, and 10mmol/l b-glycerophosphate. After 14 days, cells were fixed by 4% paraformaldehyde and stained with BCIP/NBT Alkaline Phosphatase Color Development Kit (Beyotime, Beijing, China) or Alizarin Red S solution (1%, pH=4.2, Solarbio), following the manufacturer's instructions. For adipogenic differentiation, the third-generation BMSCs were cultured in high glucose DMEM (HG-DMEM) medium containing 10% FBS, 10nmol/l dexamethasone, 0.5mmol/l 3-isobutyl-1-methylxanthine, 0.2mmol/l indomethacin, and 10ug/ml insulin. After 14 days, cells were fixed by 4% paraformaldehyde and stained with Oil Red O stain kit (Solarbio, Beijing, China) The staining was observed and recorded by the microscope (Eclipse, Nikon, Japan).

### High-throughput sequencing analysis

The third-generation BMSCs collected from OVX and SHAM rats were harvested and underwent high-throughput sequencing analysis by Novogene Bioinformatics Technology Co., Ltd. (Beijing, China). The detailed procedures of RNA-seq have been described in our previous study. Cytoscape 3.9.0 (Free Software Foundation, MA, USA) was applied to describe the miRNA-mRNA networks predicted by RNA-seq.

### RNA extraction and quantitation

Total RNA from OVX and SHAM BMSCs was fetched by TRIzol (Invitrogen, USA) following the manual's instructions, and then reversely transcribed into cDNA using TB Green® Premix Ex Taq™ II (Tli RNaseH Plus) (Vazyme, Nanjing, China). The cDNA was used for qRT-PCR using HiScript II Q RT SuperMix for qPCR (+gDNA wiper) (Vazyme) with ABI Prism 7500 Fast Real-Time PCR system (Applied Biosystems, StepOnePlus, USA). Glyceraldehyde phosphate dehydrogenase (GAPDH) served as normalized reference for RNA detection. The relative expression levels of RNAs were calculated by means of the comparative 2^-⊿⊿Ct^ method. All the primers were synthesized by Sangon Biotech (Shanghai, China) and the sequences were listed in Additional file Table S1. The BMSCs for verification tests were different from the RNA-seq samples.

### Statistical analysis

Statistical analysis was performed SPSS 22.0 statistical software (IBM, USA) and GraphPad Prism 8 software (GraphPad, USA). Comparisons between two groups used two-tail t-test, while comparisons among multiple groups used one-way analysis of variance (ANOVA). P<0.05 was considered to be of statistical significance after multiple test correction.

## Results

### Increased weights, decreased ALP levels and osteoporotic bones were found in OVX rats

**Figure 1:**
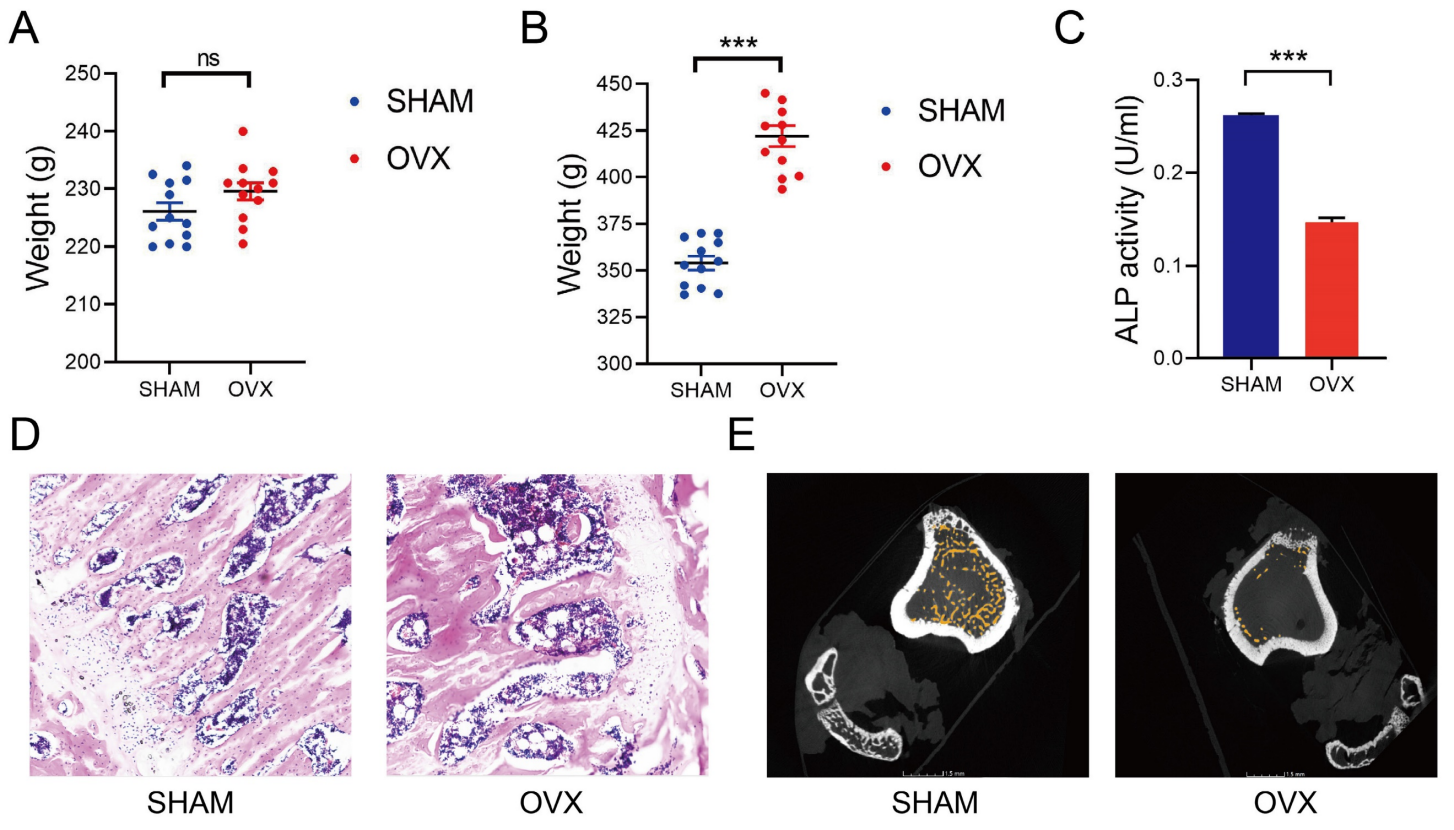
Establishment of OVX and SHAM rat models. **A)** The weight of OVX and SHAM rats before operation.** B)** The weight of OVX and SHAM rats at 12 weeks after operation. **C)** The ALP activity of OVX and SHAM rats at 12 weeks after operation. **D)** The HE staining of OVX and SHAM rats femurs at 12 weeks after operation. E The micro-CT photos of OVX and SHAM rats tibias at 12 weeks after operation.

We established the OVX and SHAM rat models as reported in the former studies^11^. The median weight of OVX and SHAM rats before operation were 230g and 227g (n=12), respectively. There was no statistical difference in weight between the two groups (Fig. 1A). By contrast, the median weight of OVX and SHAM rats after operation were 423g and 355g, respectively. Significant increases in the weights of OVX rats were found at 3 months after the operation (Fig. 1B). After detecting the blood samples, the ALP levels in OVX rats were significantly lower than those in SHAM rats, indicating a decreased osteogenic activity in OVX rats (Fig. 1C, n=3). In Fig. 1D, thinner bone trabecula, sparser cancellous bones, wider trabecula spacing and more fatty vacuoles were found in HE staining slices from the OVX rat femurs. In Fig. 1E, micro-CT displayed sparser cancellous bones in the OVX rat tibia. The findings in OVX rats conformed to the osteoporotic appearance.

### Harvested cells tallied with the identification criteria of BMSCs

**Figure 2:**
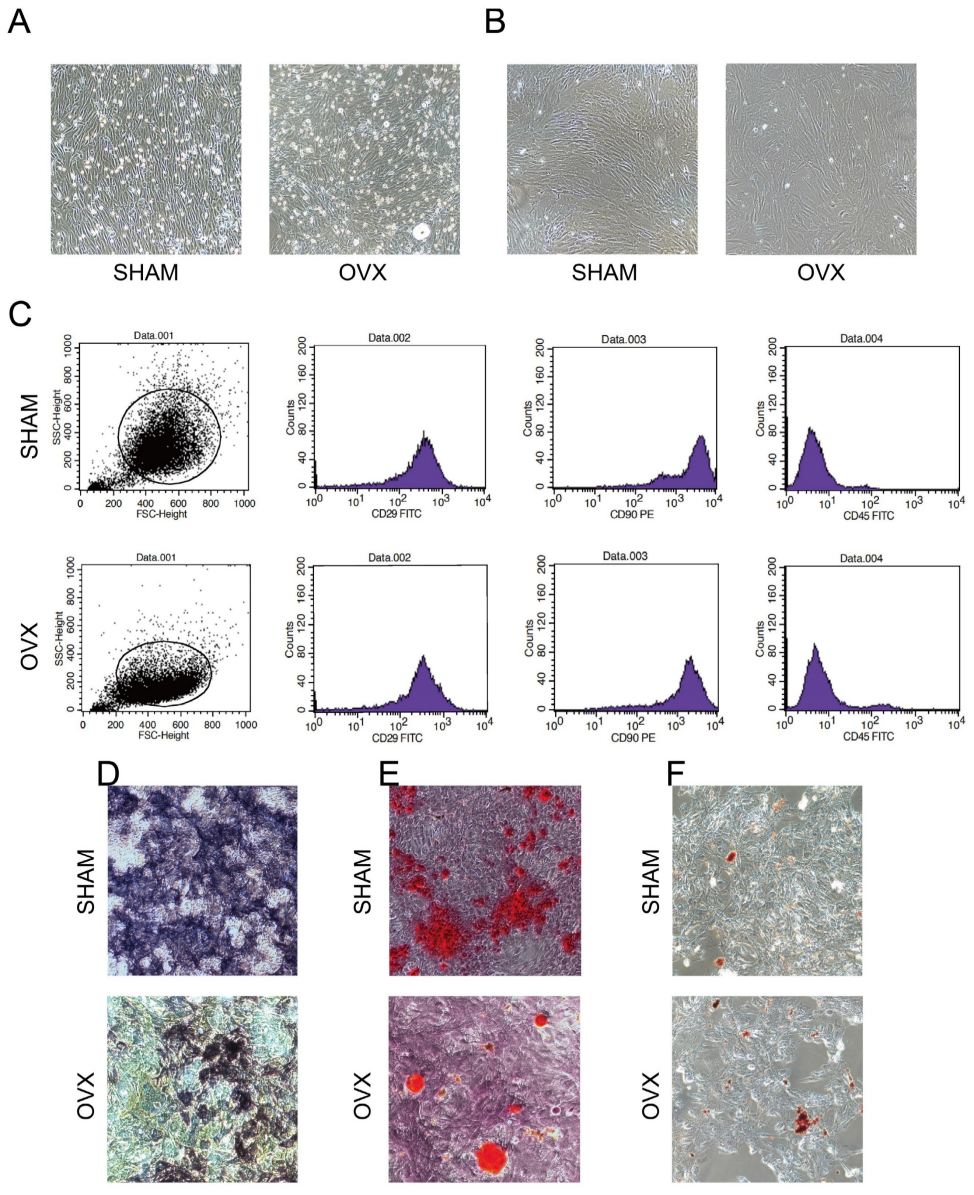
Identification of BMSCs. **A)** The morphology of primary OVX and SHAM BMSCs. **B)** The morphology of third-generation OVX and SHAM BMSCs. **C)** The immunophenotyping of OVX and SHAM BMSCs. **D)** The Alkaline Phosphatase staining of OVX and SHAM BMSCs after culturing in osteogenic medium for 14 days. **E)** The Alizarin Red S staining of OVX and SHAM BMSCs after culturing in osteogenic medium for 14 days. **F)** The Oil Red O staining of OVX and SHAM BMSCs after culturing in adipogenic medium for 14 days.

Under the microscope, both OVX BMSCs and SHAM BMSCs adhered to the surface of culture dishes and presented typical spindle shapes with gathering in volute shapes. Notably, more fatty vacuoles were flushed out into the OVX culture dishes (Fig. 2A) and the third-generation OVX BMSCs became less regular in shapes (Fig. 2B). Moreover, it was observed that OVX BMSCs were growing more sparserly than the SHAM BMSCs, and the proliferation rate of OVX BMSCs was obviously slower than the SHAM BMSCs.

Flow cytometry analysis (Fig. 2C, n=3) showed that the SHAM BMSCs carrying CD29 and CD90 accounted for 99.80% and 99.52% of the total cells, respectively, while cells carrying CD45 accounted for 0.92%. By contrast, the OVX BMSCs carrying CD29 and CD90 accounted for 97.31% and 95.26% of the total cells, respectively, while cells carrying CD45 accounted for 4.02%. The results indicated that both OVX BMSCs and SHAM BMSCs were positive for CD29 and CD90 surface markers, and negative for CD45.

Fig. 2D-F presented that Alkaline Phosphatase, Alizarin Red S and Oil Red O staining in both OVX BMSCs and SHAM BMSCs were positive, indicating that the cells could differentiate into osteoblasts and adipocytes. Moreover, the areas of NBT-formazan and mineralization nodes in OVX BMSCs were smaller than those in SHAM BMSCs, while the amounts of red fatty vacuoles in OVX BMSCs were more than those in SHAM BMSCs, indicating that OVX BMSCs were inclined to adipogenic differentiation instead of osteogenic differentiation.

The comparison on morphology, immunophenotyping and multipotency proved that the harvested cells tallied with the identification criteria of BMSCs.

### RNA-seq and qRT-PCR verified fourteen differentially expressed mRNAs

**Figure 3:**
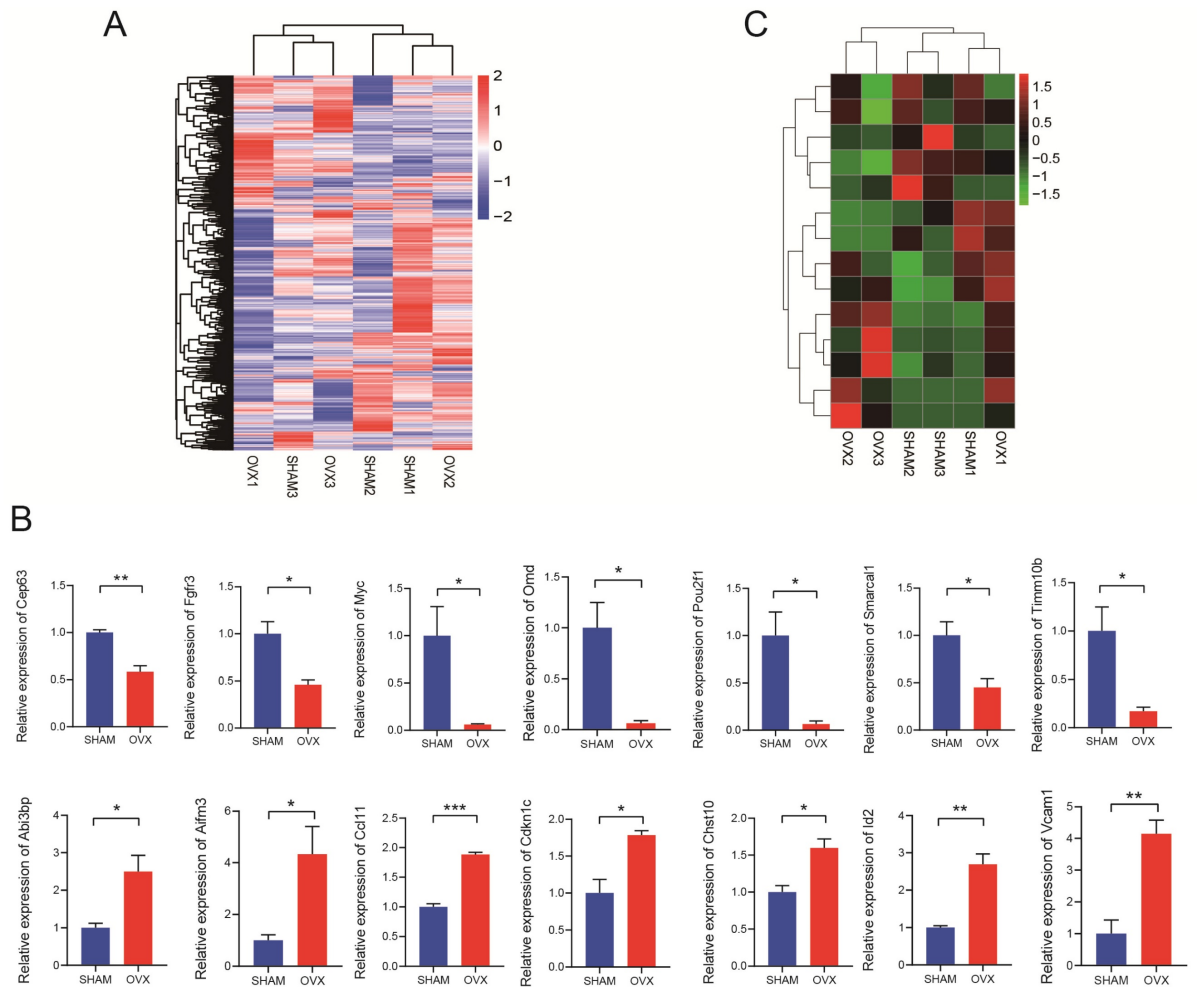
Analysis of RNA sequencing. **A)** The hierarchical clustering heatmap of differentially expressed mRNAs in OVX and SHAM BMSCs. **B)** Relative expression of the mRNAs (Cep63, Fgfr3, Myc, Omd, Pou2f1, Smarcal1, Timm10b, Abi3bp, Aifm3, Ccl11, Cdkn1c, Chst10, Id2 and Vcam1) in OVX and SHAM BMSCs. **C)** The relative expression of the mRNAs (Cep63, Fgfr3, Myc, Omd, Pou2f1, Smarcal1, Timm10b, Abi3bp, Aifm3, Ccl11, Cdkn1c, Chst10, Id2 and Vcam1) in the RNA-seq.

The RNA-seq results were displayed as a heatmap in Fig. 3A showing the significantly differentially expressed mRNAs. In total, 1998 mRNAs were found relatively down-regulated in OVX BMSCs comparing with SHAM BMSCs while 2204 mRNAs were up-regulated in some ways (n=3, P<0.05, |fold change|>1.5). Based on the findings, 24 pairs of PCR primers were synthesized and underwent quality testing. Excluding 10 debased or insignificant pairs, fourteen eligible mRNA primers were chosen for further study. The primer sequences are listed in Table S1. We performed qRT-PCR for verification and confirmed that seven of the mRNAs (Timm10b, Pou2f1, Myc, Cep63, Smarcal1, Fgfr3, Omd) were relatively down-regulated in OVX BMSCs while the other seven mRNAs (Abi3bp, Id2, Ccl11, Chst10, Vcam1, Aifm3, Cdkn1c) were relatively up-regulated (Fig. 3B, n=3). The heatmap drawing the relative expression of the fourteen mRNAs in RNA-seq was displayed in Fig. 3C, the trend of which generally matched with Fig. 3B.

### RNA-seq predicted potential miRNA-mRNA and lncRNA-mRNA regulatory networks

**Figure 4:**
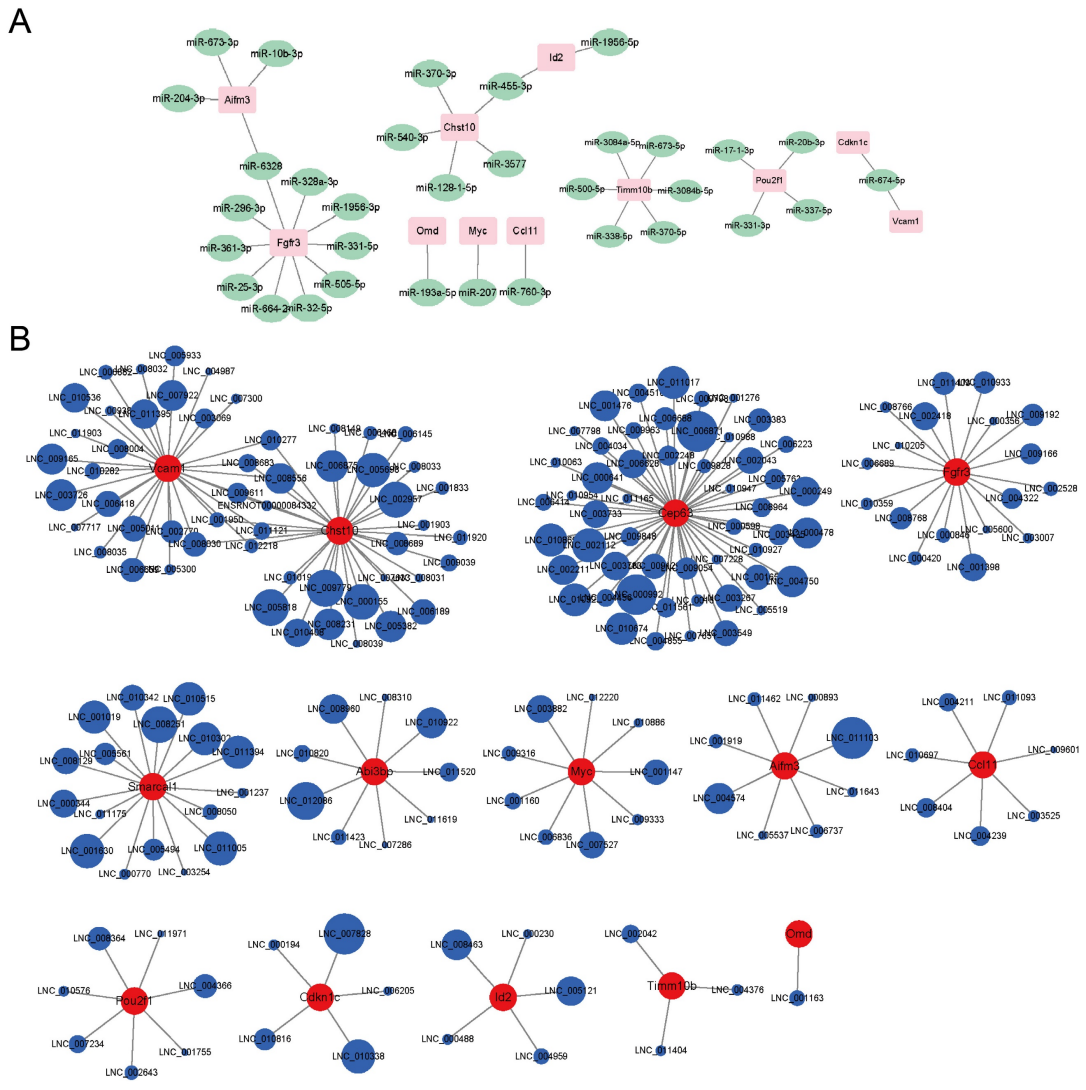
Potential miRNA-mRNA and lncRNA-mRNA networks. **A)** The potential miRNA-mRNA regulatory network analyzed by the RNA-seq. **B)** The potential lncRNA-mRNA regulatory network analyzed by the RNA-seq.

The RNA-seq also found some differentially expressed miRNAs and lncRNAs whose expression levels were significantly connected to the mRNAs' levels. A potential miRNA-mRNA regulatory network was displayed in Fig. 4A. Fgfr3 had the most miRNA targets. Aifm3 and Fgfr3 shared the same miRNA target, miR-6328. Chst10 and Id2 shared the same miRNA target, miR-455-3p. Abi3bp, Cep63 and Smarcal1 didn't match proper targeted miRNAs. A potential lncRNA-mRNA regulatory network was displayed in Fig. 4B. Cep63 had the most lncRNA targets. Vcam1 and Chst10 shared nine lncRNAs. Omd only matched one lncRNA. As most of the lncRNAs were not annotated, we provided the lncRNAs sequences in Table S2. The circle sizes of the lncRNAs in Fig. 4B were in line with the Pearson's correlation (absolute values between 0.97 to 1).

## Discussion

As the global aging problem becomes increasingly prevalent, osteoporosis, particularly PMOP affects millions of people worldwide and imposes great socioeconomic burden^12^. The present first-line agents, including bisphosphonates (BPS), selective estrogen receptor modulators (SERM), parathyroid hormone (PTH) and denosumab, might inevitably lead to a series of adverse effects, such as myasthenia gravis, osteonecrosis and tumorigenesis, along with long-term usage in treatment of osteoporosis^3^. It is of utmost importance to find a safe and efficient alternative therapy, while BMSCs become a hotspot.

Accumulating evidence indicates that BMSCs present lower osteogenic capability but higher adipogenic capability in cases of aging or ovariectomy, leading to the dysfunction of bone formation and finally osteoporosis^13^. Their findings are in line with our staining assays in Fig. 2D-F that the areas of mineralization nodes in OVX BMSCs were smaller than those in SHAM BMSCs, while the amounts of red fatty vacuoles in OVX BMSCs were more than those in SHAM BMSCs. Thus a lot of studies are trying regulating the proliferation or differentiation of BMSCs to promote osteogenesis. However, few studies chose to compare the differences between the osteoporosis individuals and the normal ones to probe into the underlying pathogenic mechanisms. Although some scholars investigated the expression profiles of RNAs in peripheral blood of PMOP patients respectively^14^-^15^. The changes in blood might fail to figure out what exactly happened to BMSCs. Geng found some differentially expressed RNAs from BMSCs of SOP patients, but further studies might be limited by the acquirement of human BMSCs and ethics approval^16^. It's worth noting that the OVX rat has been taken as an ideal model to illuminate PMOP as approved by FDA^10^. Yousefzadeh provided a practical guide for inducing OVX models and introduced that the model was suitable for mimicking the estrogen deficiency-induced bone loss and showing clinical manifestations of postmenopausal osteoporosis^17^. Thus our study harvested the BMSCs from OVX and SHAM rats and performed RNA-seq. Considering the highly conservation characteristics of mRNA, this study could help find out some crucial changes during the development of osteoporosis and provide some significant targets for treatment of osteoporosis.

Thus, firstly we established osteoporosis rat models as approved by FDA. The weights of OVX rats were significantly higher than the SHAM rats, which might attribute to the lack of estrogen. Estrogen depletion led to disturbances in lipid metabolism and caused fat accumulation and overweight^18^. The HE staining of femurs and the observation of first-generation BMSCs also revealed increasing fatty vacuoles in OVX rats. Meanwhile, the lack of estrogen led to the attenuated regenerative competence of BMSC and impairment of bone formation^19^, which supported our findings that the declined ALP levels in blood and sparse cancellous bones in HE staining and micro-CT in OVX rats. Thus we successfully establishd the OVX and SHAM models.

According to the guidelines of The International Society for Cellular Therapy, the MSCs should be characterized by: stem-like morphology, specific positive and negative immunophenotype, and multi-differentiation potency. Under the microscope, the BMSCs presented typical spindle shapes with gathering in volute shapes. Flow cytometry analysis showed that the cells were positive for CD29 and CD90, and negative for CD45. The results are consistent with existing evidences on BMSCs surface markers^20^-^21^: CD29 is an adhesion marker and CD90 is a marker of undifferentiated stem cells, while the lack of CD45 means the cells are not haematopoietic cells. In addition, after treating with differentiation inducers, the cells presented osteogenic and adipogenic differentiation ability. Taken together, the BMSCs conformed to the criteria and were reliable for performing RNA-seq.

Based on the analysis of RNA-seq, we chose some of the differentially expressed mRNAs for verification via qRT-PCR. It's confirmed that seven of the mRNAs (Timm10b, Pou2f1, Myc, Cep63, Smarcal1, Fgfr3, Omd) were relatively down-regulated in OVX BMSCs while the other seven mRNAs (Abi3bp, Id2, Ccl11, Chst10, Vcam1, Aifm3, Cdkn1c) were relatively up-regulated. The trends in Fig. 3B were in line with the heatmap generated by RNA-seq in Fig. 3C.

Some of the targets have been well studied, such as Myc, Fgfr3, Abi3bp and Omd. Myc (MYC proto-oncogene, bHLH transcription factor) is a well-known oncogene. Via FGF/FGFR signaling, it promotes MSCs proliferation, and attenuates rather than abrogates their differentiation^22^. The protein MYC forms a heterodimer with MAX to activate transcription while MAX also forms another heterodimer with MAD to antagonize MYC/MAX activation aiming at the same targets. The heterodimers regulates the fate of cells between proliferation (MYC/MAX) and differentiation (MAX/MAD)^23^. Decline of Myc in osteoporotic BMSCs might interfere their proliferation. Fgfr3 (fibroblast growth factor receptor 3) belongs to the highly conversed FGFR family. Its protein binds to fibroblast growth hormone and participates in skeletal development through a cascade of downstream signals, especially through the ERK and p38 pathways^24^. Activated Fgfr3 enhances the proliferation of osteoblast progenitors and promotes bone formation in mouse models, while anti-Fgfr3 treatment blocks BMSC proliferation and osteogenic differentiation^25^. Abi3bp (ABI family member 3 binding protein) encodes an extracellular/interstitial matrix protein, which takes part in membrane ruffling and lamellipodia formation and thus influences cell-substrate adhesion and cell motility^26^. It is considered as a tumor suppressor that suppressed cell proliferation, migration and invasion^27^. Hodgkinson found Abi3bp-knockout increased BMSC proliferation but limited their motility and ability of osteogenic and adipogenic differentiation, indicating that Abi3bp played a role in switching BMSCs between proliferative and differentiating states^28^. Omd (osteomodulin, also known as osteoadherin; OSAD; SLRR2C) encodes a keratan sulfate proteoglycan and usually highly expressed in mineralized tissues^29^. The protein binds to BMP2 via its terminal leucine-rich repeats and thus promotes BMP/SMAD signal activation, osteogenesis-associated gene transcription and mineralized nodule formation^30^. It's reported that Omd levels rose by 35 folds in dental pulp stem cells at the late stage of osteogenic differentiation^31^. Thus lacking Omd in osteoporotic BMSCs weakened their osteogenic differentiation.

Some of the other targets might have influence on osteogenic differentiation, including Timm10b and Pou2f1. Timm10b (translocase of inner mitochondrial membrane 10B, also known as Fxc1, Tim9b, Tim10B) is a member of the evolutionarily conserved Tim family, whose protein locates in the mitochondrial intermembrane space. The hetero-oligomeric TIM9-10 complex hand over the carrier precursors carrying hydrophobic proteins to its membrane-associated TIM9-10-12 complex, which is tightly associated with the TIM22 complex in the inner membrane. Then the proteins are inserted into the mitochondrial inner membrane along TIM22 in a dynamic manner following the membrane potential^32^. One study found that it increased 3-4 folds during the production and deposition of matrix proteins along the osteogenic differentiation of MC3T3 cells, but the mechanisms were not studied^33^. Pou2f1 (POU class 2 homeobox 1, also known as OCT1) is a member of the POU transcription factor family that regulates transcription via binding to the octameric sequence ATGCAAAT found in the promoters and enhancers of diverse genes. It stimulates RUNX2 recruitment to the β-casein promoter by interacting with the C-terminal region of RUNX2 and thus relieves the autoinhibition of RUNX2 and regulate bone development^34^. It can be modulated by AMPK signaling and acts on the promoter region of miR-451a to regulate osteoblast differentiation and mineralization^35^. Taken together, inadequate Timm10b and Pou2f1 in osteoporotic BMSCs harmed osteogenic differentiation.

Some of the targets might have influence on cell viability, including Cep63, Smarcal1 and Aifm3. Cep63 (entrosomal protein 63) encodes a protein making up part of the annular structural base of the centrosome. It promotes the replication of centrioles and mitosis, linking the centrosome and the cell-cycle machinery via recruitment for CDK1^36^. Knockdown of Cep63 leads to centrosomal distortion, chromosomal entanglement and telomere clustering defects, resulting in cell cycle arrest in the S phase^37^. The role of Cep63 in osteology hasn't achieved much attention. Mutations in Cep63 cause Seckel syndrome, a skeletal disease characterized by microcephaly and dwarfism, indicating a potential connection between Cep63 and bone formation^38^. Smarcal1 (SWI/SNF related, matrix associated, actin dependent regulator of chromatin, subfamily a like 1) is a member of the SWI/SNF family, whose protein presents helicase and ATPase activities and regulates gene transcription by altering the chromatin structure around the gene. It takes replication protein A off DNA and anneals complementary strands, as well as keeps replication fork and genome stabilization^39^. Downregulation of Smarcal1 leads to transcriptional repression and G2/M checkpoint overriding, leading to mitotic abnormalities^40^, and the damage remains observed beyond stem cell differentiation^41^. Mutations in Smarcal1 also cause severe skeletal disorders, such as Schimke immuno-osseous dysplasia (SIOD), characterized by growth defects, immune deficiencies and other complex appearance^42^. Lack of Cep63 and Smarcal1 might affect osteoporotic BMSCs viability and the mechanisms deserved more study. Aifm3 (apoptosis inducing factor mitochondria associated 3, also known as AIFL) induces cell apoptosis in a caspase-dependent manner by changing the oxidoreductase activity and mitochondrial membrane potential^43^. It's reported that MSC-derived exosomes protect cardiomyocytes from apoptotic cell death via downregulated Aifm3^44^. However, apoptosis usually presents two-sideness in regulating the life-death balance: higher Aifm3 levels are found associated with shorter overall survival in breast cancer, while Aifm3 also decreases stem-like properties of breast cancer stem cells and has potential to suppress tumor progression^45^-^46^. Aifm3 might play similar roles in BMSCs apoptosis comparing with tumor stem cells^47^, but the molecular functions haven't been set forth clearly.

Evidence has found that estrogen deficiency affects the level of inflammation which contributes a lot to osteoporosis. The inflammation microenvironment affects cellular physiological processes and leads to cellular senescence. Senescent BMSCs can't function normally and will secrete more inflammatory cytokines forming a vicious circle^48^. Vcam1 (vascular cell adhesion molecule 1) is a member of the Ig superfamily, which is up-regulated in response to inflammatory cytokines such as tumor necrosis factor-alpha (TNF-α)^49^. It is a principle adhesion molecule in the interaction between HSPCs (hematopoietic stem and progenitor cells) and BMSCs^50^. Burja used inflammatory cytokines to stimulate different kinds of MSCs and found BMSCs expressed the highest levels of Vcam1. In return, the increased Vcam1 in BMSCs strengthened the interaction with HSPCs and hindered HSPC mobilization, leading to a systemic immunodeficiency^51^. Moreover, Vcam1 repressed the MURF1-mediated ubiquitylation of PPARγ2 and maintained PPARγ2 stabilization, which enhanced adipogenesis and consequently reduced osteogenesis of BMSCs^52^. Ccl11 (C-C motif chemokine ligand 11) is an antimicrobial eosinophil-specific chemokine involving in immunoregulatory and inflammatory processes, which induces cell chemotaxis and angiogenesis^53^. Studies have reported that serum Ccl11 levels elevate in rheumatoid arthritis patients and osteoarthritis patients^54^. The accumulation of Ccl11 increases preosteoclasts migration and promotes osteoclastic bone resorption^55^. Comparing with healthy subjects, serum levels of Ccl11 in osteopenia and osteoporosis patients significantly increased^56^, which is in line with our findings in osteoporotic BMSCs.

Some targets have multiple functions. Id2 (inhibitor of DNA binding 2) belongs to the ID family, a group of helix-loop-helix (HLH) transcriptional regulators. Basic HLH transcriptional regulators usually have an HLH domain, which allows proteins to homodimerize or heterodimerize and recruit complexes to act with DNA, and a basic domain, which is essential for binding with DNA. But the ID family lacks of the basic domain. Thus the family can't bind to DNA directly but binds to other basic HLH transcription factors in a dominant-negative manner, preventing them from binding and interacting with DNA^57^. It is found that Id proteins promote the proliferation of osteoblast progenitor cells but they must be down-regulated during the late stage of osteogenic differentiation^58^. Similarly, Id2 has been found to promote self-renewal of MSC and suppresses osteolineage commitment^59^. Thus high levels of ID2 in osteoporotic BMSCs might fail to activate osteogenesis. In addition, Id2 strongly promotes osteoclastogenesis in rheumatoid arthritis models^60^, indicating it might strengthen osteoclasts in some way. Chst10 (carbohydrate sulfotransferase 10, also known as HNK1ST) encodes a sulfotransferase targeting to the human natural killer-1 (HNK-1) glycan and contributing to the HNK-1 carbohydrate biosynthesis^61^. HNK-1 was present in the osteoblasts, osteocytes and osteogenic cells during maxillaries osteogenesis and bone defect repairment^62^. Thus Chst10 might contribute to bone repair when osteoporosis happens. Evidence also found Chst10 modulate cell adhesion, recognition and migration^63^. Misa Suzuki-Anekoji generated Chst10-deficient mutant mice and found serum estrogen levels in Chst10-deficient mice were higher than in wild-type mice, suggesting that Chst10 could regulate steroid hormone through glucuronicdation^64^. The changes of Chst10 in OVX BMSCs might also attribute to the lack of estrogen. The underlying mechanisms deserve further study.

Taken together, the pathogenesis of PMOP is a complex process. Changes have been found affecting cell differentiation and viability, or in response to inflammation, bone repairment and estrogen defect. In addition, the RNA-seq also found some differentially expressed miRNAs and lncRNAs that might interact with above mRNA targets. MiRNAs usually act as a negative regulator over the process of gene expression by either mRNA degradation or translational inhibition^65^. Some of the miRNAs have been well studied. For example, Hu found miR-1224-5p was positively correlated with fracture healing progression and identified miR-1224-5p as a key bone osteogenic regulator by targeting ADCY2 via the Rap1-signaling pathway^66^. But the roles of most of the miRNAs remain unclear. LncRNAs are commonly found as competitive endogenous RNAs to sponge miRNAs and modulates downstream mRNAs function. For example, Wu found lncRNA SERPINB9P1 regulated BMSCs osteogenic differentiation via altering SIRT6 mRNA levels through its suppression on miR-545-3p^67^. However, most of the lncRNAs are found by RNA-seq for the first time and their functions are not annotated. We provided the lncRNAs sequences in supplementary file in anticipation of extensive studies.

Limited by the difficulties in harvesting BMSCs from OVX samples, we haven't tested and verified the differentially expressed miRNAs and lncRNAs as well as possible networks. We hope our findings could be a rich source of inspiration for more scholars. In addition, for similar reasons, we didn't have enough cells to compare the protein levels of above targets between OVX and SHAM samples. In the following studies, we will cover the shortage and deeply study the underlying mechanism of each target to make the findings more reliable.

In conclusion, benefit from RNA-seq technology, we compared the differentially expressed RNAs from OVX and SHAM BMSCs. 7 up-regulated (Abi3bp, Aifm3, Ccl11, Cdkn1c, Chst10, Id2, Vcam1) and 7 down-regulated (Cep63, Fgfr3, Myc, Omd, Pou2f1, Smarcal1, Timm10b) mRNAs in osteoporotic BMSCs were proven by qRT-PCR. In addition, potential miRNA-mRNA and lncRNA-mRNA networks were illustrated. The changes were found covering a large set of biological processes, including cell viability, differentiation, immunoreaction, bone repairment and estrogen defect. This study enriched the pathophysiological mechanisms of PMOP and provided dozens of attractive RNA targets in treatment of osteoporosis for further study.

## Supplementary Material

Supplementary tables.
