# Supplementary material for: What happens to the osteoporotic bone mesenchymal stem cells? Evidence from RNA sequencing
Source: Int J Med Sci. 2024 Jan 1;21(1):95–106. doi: 10.7150/ijms.88146 (PMC10750345; doi:10.7150/ijms.88146)
Supplement: Supplementary file 1 — Supplementary tables. [file ijmsv21p0095s1.pdf]

Table S1 The primer sequences of mRNAs

Name	Sequence
Abi3bp	F: AATTCACGGAGGCTGTGGTTGATG<br>R: CTTCTTCTGGCTGGGAGGAGGAG
Aifm3	F: GCACAGGAGGCAGAGATCAACAC<br>R: AGCCTTCTCCGTAGCCAGCATAG
Ccl11	F: ATCCCAACTTCCTGCTGCTTTACC<br>R: GCGTGCATCTGTTGTTGGTGATTC
Cdkn1c	F: AAGCGAACAGGCAGGCAAGC<br>R: AAGGCTGGCTAATTGGTGATGGAC
Chst10	F: ACCTCTCTCCTGAACGCACCTC<br>R: GACAGCACTGGACTCCGCAATG
Id2	F: GGTCCGAGCCGAGAGTCCTTC<br>R: GCGTGACCAGCAGCAGTTAGAG
Vcam1	F: GCTGCTGTTGGCTGTGACTCTC<br>R: GCTCAGCGTCAGTGTGGATGTAG
Cep63	F: TGCTCGATGCCCGTCTCTCC<br>R: TGCCTCACCGTCTTCTCACTCTC
Fgfr3	F: AGGAGGAGCTGATGGAAGTTGAC<br>R: ACCACCAGGATGAAGAGGAAGAAG
Myc	F: CGCCTACATCCTGTCCGTTCAAG<br>R: AGATTCCAGCTCCTCCTCACTTCC
Omd	F: TGGTGAGCAGAGGAGCACTAATGG<br>R: TGTGGTCCTGACTGTCGTCTTCC
Pou2f1	F: CCTCCTTGAGTCCCTCTCCTTCTG<br>R: GTGGTCTGTGTTGTGCTGGTCTC
Smarcal1	F: CTGCTGGTGGTTGTGCCTTCC<br>R: CGTTGATGTCCTCTGGGCTAAGTG
Timm10b	F: GGAGCACCAGCAGCAGCAAC<br>R: GCCTCCTCCTCAGCGTCCAG

Note: F: forward primer, R: reverse primer.

Table S2 The sequences of lncRNAs

>LNC_000155
CTCCATCATGATTTCCCTGTATAGGCACTTCTGTTGGAGGTCCAGCTGTGC
CCACTCTTCCTTGGTAAATGTCAGTGATACATCTTCAAACTTCACAGATTC
ATGGGGCCAGGTTGTTGGAAGCGTGGAAGCCATACTGTCCTCAATGTTCT
CTCGCGCGAGTAGGCAGAATGCTGACGAAACTTCTCCCGGGTCCGTAGAA
GTGGAGTCACTAACGAAGCCGACACTAGGCTCCAGAAGTTACGAATAAAA
CATAAGATGGGTGCTGTCCGACGGACTCTCACGAAGCGTTACAAGTGACT
GCGAAAGACACAATGGCGCTCGCCCGTCGGTCTTGTCCCGAACTGCGTCA
GCTCACTGGTCCG
>LNC_000194
CGCAATTCAGAGTCTTGAGCCTAGCACGCAGATTGCGCTCTGACGCCTTTA
CCTCCTCGAGCTCCTTAGCTAGTCTGCGACAATCCTGGCTGCGGCGGCCAG
CAGCCCGACCCCCAAGGCCACGCTCTTGCCGTAACTCCAGCTCTAGGCCC
CTCACCAGCCCCCGCAGCGACTCGGCTTCCTGGCGAGCTGCCCGACCCGC
CAGAGCCTCCTCTCGGGAGCGGCCCAGCTGCGCCTCCAGCTCCCGCTTCTC
TGACGCCAAGCGGGTCACCTGCTCCCTCAGATGCCAGATCTCTGTCTCCCG
AGCATCCTGGCCGCCATTCATCCCTCGAAGGTGACCCAACTCCTCCTTCAG
TGAGCGGATGATGCCCTGCAGAACCACGGGGTCAGGCTTGCCCTGGTACG
GGAGGGGCAGCGGGTAGTGAATCCTGTCAAACTCCACGGAATAGATGAG
GATGAGGTAGCGTTTGGAGTTGAGCTGGGCCGACCTGGGGCAGGGACCCG
GGCGGCCCCCCAGCTTGCGACTGCGCAGAGACTCCAGGTCTGTGTAGGTG
AGCAGGTCCAGAGTGACAGACTCACTACTCTGGGTGAGGGCTGACTCCAG
CATGTTACAGAAGATGCTGAACTGTTTAAAGTTCCCGGTCTTATGAGTCAA
GTCTTCAATGAAGTTGGCATCAAACTCTCCACGCCACTGGTCAGCTGTCAT
CCGGTCCTCCACCTCCAGCTCCAGCACCTGCCCAGAAACCACCACCCGCA
CAGCATGCTCCACACCCCGGAACATGTAGTCCACCTGCAGGCCAGCTGGC
TGCTCCATGGCCAGGGTCCCTCACAAGCTCTAGTGCAGTATTCGGTAAGG
GACCTCTAAGTTCTAGCCGCGTATATCGGGAGCGACAACATAGCGCAATA
AAGACTCTGTGAAATCTCCAGCTTGCATAGGTTCTGGCGTCCACCTGCGTG
AAAGGACCTGTGCTCCGGGGACTCCCCAATCCCCGCGCCTGGAGCCCGCG
CCTGGAGCCCGCGCCCGCAGCCGCGTACTCGCTGGTCACCGCCACAGACG
CCCTCTTCA
>LNC_000230
TTGCCTTTTCCAGGGTGAAGGCCTCCTGGCCTGTTGCTAGCAATTGTGATG
GTCTGCCCTCATTGGTCAGGGCTTCCACAGAGTCCACGCACAAGTGGCTTT
TTTTTGGTCAGGCAAGCATCTGGTAGGTTGTGGGTGTATTCGGCAGGCCTT
CTTAGGTACCATCGGGGGAGCAGACAGACAGTGGGTACAGATTTTACCTC
ACCTGTGGCCAGCCCCACTCTGCAGTCATTTCGAGTATTGAGATCTCGGGC
AAAAAGACGCTCCCTGACTCCAGGTAAGTGATTTTTTTGTTTCCATGGCAA
TTTGGAGCCAGCTGTCTGTCTCCCAACCAGTTTGTAAGAATCACCTGGTTC
TCGCACAAGGGGAGGGTGCGGTGACTCCTGGGGGGGAGGTGGAGTTTCTA

CACGGCCAGCTCATTGCTCACTTTTCTTGAGAACTCCAAAGCCAGACGTCT
CTGCAGACATGGCTGAGGCTGGTGTTAAGTAAGCTATAAGGCATTATGGC
TCAGATGTTGGAGGATACATAGTACAGGTTGATTGGATGGTGCTGGTGAG
CAGGAATAGGGTGGGTAGGGAGGGAGAGCACTCAGCCAGCCTTTCACGG
CCAAGCAATGGAGAGGACATGAGCAAGAGCCTGTTGTGGGAAACTGACG
GCAGTGGTCGTGTTGAGAATGCCAGTTAAGATTTTTTGGAGATCTTTGGTT
ATCTAGAGAGCTGGACTTTTTTGAAAGGACTGAGGCACCATGAGAAAGGA
AGGTGCACAGCTCAGTTTTGCCACAGTGTGGAAGTACATTACCAGGAAAT
GACAGCTCATGGCAGTGCCCTAGGCAAGAGTGGCCTAGGATGTGACAGGG
GGTGGGGGGTGTTGAGAAGGCCAAGAAATGACAGGTGAGCTCTCCAGAC
AACTAAGTTGAAGGATAAGGTAGAGAGAGGCAGGAAAGGGAGGTCTCCA
ATATATACAGCACCCCGTCCCCTGTCAATAGAGTCTTGGTTGGTGGTTTTG
TTTTTTAATTATGTGTTCTCTTGGTGCTGGGGTGGAATCTAAGAAACCCCA
GGTGCTAGGCAAGTGTTCTACATGCCTAGCCTCCCAGTAAAAAGATTTTG
AATGAACAGTCGTAGAAGACAGGACCTTTCTAAACTTGGGAATAATTCAG
GCAAAGGGAAGTTATTCTTCTAAGGTCACACAACCAGGAAGTGGGCTCAG
GTTGCTGTGCGTGGGTGCACTGGTCATTAATTTAACAAGGCTTATTTGAGC
CTAGAGGCATCTGCGGGGCCAGATCCCGTAGGCCCTGTGAGGAATGTGGA
ATGGGCATGGAATATCATTTGTATTGTGGTTGAGAACCAATGCCCAGGCT
CCAGGCTGACTGATGACCTGATACTTCTTTGGTATTTCCAGGGTACTCTCA
TAAGTTCAGGCAGGTGAAAGCCTACAGCATCCTCCTCATTGGTCTTCTGCC
CTCACCATTTCCCCATGTAACCAAAGAGATTTTGGACACCTATTTTTCCCT
CCCTGCTGAGAATCCCTCTGGACTCCATCTCACTCAGGGTAAAAGTCCACA
CACATCCTTTCTGTGACCTCTGCATCCTCACCATCCACCCTCATCACCTGTC
TGAACCAGTTGACTCTTTCATTCTGCTCCAGCCACACTGGGCTCAGCCTTT
ACTCTTGGTATTCTGTGCCTGGATCTTTTGTCGATAGACAGCCTGGACTCA
ATGCCTGACGCTCTTCTGAATCATACTGCTAACAGCCTGACCTGACCATTC
TTCCTTTCACTTTTTCCTTGTATCAATCATATTGCTGCTGGCACATGCTAAA
ATTTATCTTGTCAACTGCCTCCCCTTCGGTGAGGCCTTGAACTTCTTGGAA
GCAAGTATTTGGGTCACTCATGCTTTATTTTATCTCCCACACTGACGAATG
TGTGACACCCCTCAGGCTCACTGTCAGATGAGTACTTGAGAGCATAGATG
CAGCCTCCTGGGTGTCCCACATCCCCTTCTATACAATAAACATTGCTGCGA
ACTCCAGCGGTGAGCAATGTGGGAAGACAACAGAGAGGAGAGGATAGTT
GCTGTGCCGTGAAAGACCTCAGTCAAGGTGGAGTCAGTCGGGTGGCATAG
CAGTGGGAAGGGACTTGAGATCAAGAGCATTGAAGTCTGGATTAGGGGTT
TCTGACCTGGCTCCTTGGAAGACTATCCAGGTCATAGGGTCGGCGTCAGCT
CTATTCAGAGAAGTGCATGTGATGATTGGGACCCAGAGGCTGAGGAGGAA
CACAAGGCCATACCTTGTCCATCTGTCCCCTATACCCTGTGTTCTCCCTCAT
CCCCATGTCATCCCGTACCCTATCCCACCCCATGTATGGAGAGTTGCAGCA
GAATTTTAATCAGTGGGACGCAGTGTAAGGTTAGCTATGTTGGCACTTTGT
TTCATTTTAGATTCATATACATGCATATTTTGCCATGTTCGTGCCTTGCGCC
CTCAGAAGTCAGAAGAGGGCATGAGATCCCTGGAACTGGAGTTAGGGAT
AGTTGCAAACTACTATGTGGGTGCTGGGAACTGAACCCAAGTCCTCTGGA
AAAACAGCCAGTATTAACCACTGAGCCATCTCTAGCCCCAGTTGTATATTT

TTAAAGGTACCCCTGATGGGTCTTACAATGGCTACACTTGAAGACAGTCC
CTATTGTGTGCTGTTTCTACTCAAGATGAGGAATCCCTTCCCCTTGAGTAA
TGGCTGTGCCAGAGATCAACACAAGAACACTGAGCCAAGTGGCCTTAGCA
GGTCTGACAGCCTCCTCTGTCATCCCTGCTTCCCTGATTTCTTTTTTGGTTT
TTTCAAGACAGGGTTTCTCTGTGTAGCCCTTGCTATCTTGGAATTCACTCT
GTAGACCAGGCTGGCCTCAAACTCAAGAGATCTGCCTGCCTCTGCCTCCC
GAGTGCTGGGATTAAAGACATGTGTCATCACCACCAGCTCCCTGATTTCTT
TATAGAGGTCAGCATCTGCCCTACCTATTTCAGCTGTGCCAGGTATGCTCA
TGGCATGGTGGACTTTCTAACTTAGCTGACACTACCCTGGAGCAGAGACC
TGAGTCTTCCTCAGGCCACTCCCCTGGGCCTTGTCCATATTCCAGAATCAT
GAAATAAACAATTGCGATTTTTTTATGACACTGTTTTAGGATGGTTTGCTT
CTCAATAGCTAACCAAAATAAGGCAGTATTTTAAGTCATACAAAGGTAAG
ATGTATAATATCACGTGATAACTGATTGAATCCTCACAGGTGTTGTCATAC
CCCCCATACAGATGAAAAGTCAAGGCATTGCAAGGTACCTTGTCCATAGG
AAGTCCTGAGTGCTCAGTCACTACTACCAACATTGTGTCTGTCCTGATAGG
TGTTGAGGAGGTGGTGGACAGGTGAATGGCCTTACTCTTAGGAGAGTAGC
TGTGGGCTAGTCCCTTGGCAAGATGGAAAGAGCAGCCCATGCTTGGTGTG
GTTGGAGTTCCATGGAGTCAGTTTGCTTCCGACTCCATAGGGAATCCAGCA
GGTTGTGGTAGAGCCAAATCAGATTGAGCCTCTGGTTACTGGGAAGCCAT
TATGTCCCAGTGAGCTGTTGCTCCTGTTCACTCCTTGTTGGTTCTTTCAGTC
ACAGGCACTTGCCACTTTTGGGCGATGCAAGTGCTACAGCAGCAGTCAAG
TGGTTCCAAAGCCTGCATCCCAGGGTCAAGGGGAATGTTAGTGAAGAGCC
ATAGACACTGAGAATCAGAGGGAGGGCCAGAGACAGGAGAGCTCTGTAA
GGAAGGTGTCTTTGAGGAAAACATTAAAGAACTAGGCCAAAGGGGAGGG
AATCATATAATCTTAAGGAAAGATATTCCTGCCAGGAAAAGAAGCCAGTT
CGAAGACCCCAGGGTATGCAAGAGAGAAGGGAAATTGGACAGGAGTGAA
GAGAACAAGGACAAAGTCCACGAGATGATGTCATGAGGGCTTGACTCTCG
CACTGAGTAACATGGAAAGCATCAGCCCCTTTGAGCTAGAGCGCTGAGCT
CTGAGGTGCCTCCACCGACAGCTGTGTGGGCAGTCCTGCTGGTGTTGATGC
ATGTGGCTTGGCCATAGCAGTAGCAGGAGAGGTGCTCAGTGTGGGGTGGA
AGATGAAGGGTGAGAGAAGCCCATAGCTCTAAGTCTGGGAAGGAGGAAG
GATGCTGGCCTATGTATGGGAAAGAGCAGGCAGGTTAGGCACATTTGACA
GTGGTCATTGTGTTGCTACTGTGAAATCTCTGTGAGACCACAGAAGGGGA
AGATGGAGCCTAGGACTGGACGGCCACAGTAGTCTCTATGTAGGATGACT
AACATGGAGGTTACTGGGTCTGAATAATGTTGAATTCAGGACTTCCCCAC
CTCATCGTTGGTCCTGAACTGTGAAAGCAAGAGAAAGTCCTCCTGGGAGT
AGAACTGAGTCTACTCAGTCCTCCTGGGACAGTGCCTGGCTCTCCAGCGCC
AGACTGAGTCTGGCTCCTGCCTCAGCATCCACTGATTCCTCTTTCTGTGCA
GGTGGCTAGCTGCTCTTGGGAAAACTCTTACCCCAGCTCTTTGTGGCTTTC
CTGTCTAGCTCCCTGCCGCTCTTCCACTCTTGGCCCCAATTCAGCTAGCTA
GAGTTGAGTTGCAAGTGCCAGGAATACAGGTCTGCAGCAGCTGATGTGTG
TCTGCTGCAAAGGGCTCTGTGACCCCAAGTCTAACAGTTAAAATGTACTC
ATGGTTGGTATAATAGCTCAAGGATTTGAACCTGGAATTTTGGTGGGGGA
GGGAGGTAAGCGGTCCGTTTATGTGCGTGGATCTGAGAAGTAGGCAGGAA

ACACTAGCAGCGGGCATCCCTGTAGAAAGTAGTGTAGCAGGCTCTGAGCT
GAGGCGGGCTCACAGTTAGGTCAGGAGCATTTCTTCCCACATGTGTTCCTG
AGAAGCAGACATAATGATGGTAGGGCCAGCCTCTGCCTTCACCATACACA
AGCCGGTGGGAGGGCAGGCTGCCTTCAGACAGTGGTCAAGGCTGAGACG
GGGAAACAGGCTTGAGTAAGTCATGGTGGCACAATGAAGAGGTTGCAAA
GGGCTATGCCTGGCCCAGTCTGAGGTGTCAGAGAAAGGAGATGGGGGTG
AGGCGTACAGGAGTCAGATGGTAGCTGTGGACCACTGGAGCCCAATCAGG
GAGGAAACTTTCATTCAATAATTTTAGGTTTGTAGAAAAGTGTCAGGGAT
CATGCAGAGGCTGTCGCCAGATGGACCCAGCTCCCCTGGTGTCAGTATCTT
CTATTCTAGGACACCCACCAAAGCTAAGACACCGATGCCTGCTGGTGTTG
GCTTAGCTCCAGTTTTCTAATCCCATCTCTCCCATTTGCCTTTGAACATCCT
TCTTCTGTTCCAGGTCTAGCCCAGGGGGCCATACTTCGTGTGTCTTCAGTC
CCAGACCGGGACCGTTCCTCCAGCCTTCTCTTCTCTGCAGGGCAGTGTTGA
GTGACGGTGCTCAGCGATTCACAGAGTGATGTCTGTCTGGATTTGTTAGTT
TTTTTCTCATAGCTATATAGGGATTGTAAATTCCACTTCTATTGTCTTTCTT
TAAGAAGTGGTCACAAACTTCACCTCACACTCAAAGTGAGGAACACTAAG
CTCTAATCCCTGGAGAGAAAACTGTCTGAAGGGTTTGTATCCATGTGCTAA
AACTACTGCAGCCCAATAGTCTGCTTTCTGTTGATCTAGCAAAGTACCTGA
GACTGGGTCATGTATGGAGAAGAGCTCAGTCCTGGAACTGAAAGTCCAAG
GCAGAGCAGCCCCATCTGTGTTGATGACTTGAGGCAGACGCCATTGTGTC
TGAAGGATCAGCAGTAAGACAGGCAGCCAGACAGTGAGGAAAGTCAGGC
TTGGTGTTTATATGTTCATGACAACATTACCAAGAACATCAAGCTTCTAAT
ACATGCCCTTCTGAGCAATGTTCACACCTTCCAGGGCATCATATACAGTAG
TTAGCAGACGCAGGACACGGTGGGACAGTGACAGTGGCCCCGCTCCTGAG
GATCACCACTGTTGGCACTCCTCCCCAGTGGTTCTGACCTATGGCATTCTG
ATGAGAATGTGTTTTGTTAGCTTATTTCTGTCACAGTTGTGTTGGCATGCA
CTCGAGGCATCCGTTTCCTGTCGGTGTAGCATACCCAGTGTTGCTCTGACT
GGGTTTGTTTTGTCCAAGCTGCTGCTGCCTTCCAGGCAGGCTCCTGTATCC
TCCCTGCTGTCTCTCCTTGAGTTTTCTTTTTCTACACCATGTCACAGGTTCA
AGGGCGTTTCTCCAGGCAGCTCTGAACCCTTTTACTGAAGAACGGTATTAG
ACTCAGGTCCAGGCATGGGGCATGCCGAAGGCATCACCCATCTGTAACCC
CACACACACGCAAACTCTGCATTCCACTGTTCAGGCATCTGACTCAGGTCA
GCACCCAGGAGCCACACAGATTCCCACTTCCTTTGGACCTTCTTTGTGGCA
AGCAAAGCCCTTTTCTGTGCTCTTTGTTCTTGTGGCCATCTCAGCAGAAGC
CAGCAGCTTGGCCAGGCCTGTGTTCAAGGCACTTCTCTCTGCAGGCAGACT
GCCTGCCATTCTGCTGGCCCAAGAGACAGCATCTAGCAAGTCCCAGTGTTT
TCCTATTTACTGTCTGAGGGCTCTCCACGCTCTCCTCACTCACCGAAGTCT
CACAGTCAACCTTGTTTCCTACCTGTGCTGGTGTGTCTCCTAGTCCTTACCC
CTGGTAGTCCTTGGGCTCTTAGCTCTATATGCTGCCTCACCCTGACCTCTCT
CAGAGTGACTCCTGCATGGCCACCAGCTCACCTAGCTTGTCCACTGAGGCT
TGCATCTCCCTTCCGTCACTGCCTTACCTACTGCCGTCCTCCCACTCCCAAC
CTCCTCCGTTTTCAGCCCAATTAAACTCTGGGAAACCAGTAAGAAGTTGCA
CAGTGTGTAGCCACAAGGAAACAAACCTCCAGGAACAGTTCATTCCTGCA
AAGGCCCCACCAAGTGGGAGGGGAAGGAGGTTTGCCTCTTTGATCTTCTT

GAATCACGTCTACGCAGCAGCTCCTGTCTCTTACACATATTCAAGCCGCCC
CTCCAGGCACCTTCAGATGTCACCATCCCTGCCTAGAGTCAGGGCTGTGCT
GTGCTGTGTGTCATAGCATCTGGAGTAGCTGACAGATTTTCTGGGTGTTGG
CCACGTTAAGAGACTACTACTGTGGAGTCTGGCTGTGAGGACAGCGAGTT
GGTGTCCATGAGCTGTCAGTGTGGCCATTCGATCCATCTTAGAATGACCAG
TGTGGTGACATGGACCTGTCCCAGCTCTTGAGAGGCAGAGGCAGATGACC
ACGTTTTGAGGCTAGCCTGGTCTCAAACTGTACCAATAACTGCTGACCCTT
CACAGTAGTGTCTTCAAGCCAGGCAGTCTGGAGTCCTTTGTAGGCTTGTGT
AGTTACTGCTTAGTTTCCTAGAAGATGTTCTGGGGATAGAGCTGGGCCTGA
AGACTCAGCCACACCGTAGCCAAGATTCAGTCACCTTAGAACAGGTTCTC
AGTGAGGCATCTGGCATTTAAACTTCCCACCTGTTTTGTTTTGCTTATGAC
AGAGCTTCAGCCCTTAGTACCTACATGGCAGCTCACAACATCCATAACTTC
AATTCCAGACTCCAGTGCCCCCTGCTGACCTCTACAGTCACCAGGCACATA
TACATACATGCAGGCAAAACATTCATACACAAAAAATAAGTTGTTTTTTTT
TTTTTTTTTTTCTTGGAGCTGGGGACCGAACCCAGGGCCTTGCGCTTGCTA
GGCAAGCGCTCTACCACTGAGCTAAATCCCCAACCCCGCAATAACTTATTT
TTTTTTTTAAAGGTATGGTGGACCAGCCTTTAATCGTGGCATTTGGAAGCT
AGAAGCAGGCAGAGTTCTGAATTCAAATTCAGCCTGGTTCTACATAGCAC
CTGGGAGCTACCCAGGGAGCCCTGTCTCAAAAACAAGCCAAGGCCTCGTG
CGGCCCAGAGCTTTGCCTCAAATTCACTGTGGTTTGGCCTTGACCCCTGAC
TCCTGCTTCCCCTCCCAGCACGCTGGACCTCCAGGCCTGTGCCCACAATTA
GGCTCCAGCTTTTTCAGAGAGAAAGGCCCTTGGGCGCCTTTAGCCACTTTC
TTCCTTGACCCCTCTGAGAGGAGGCAGTCGGAGTTCTGGTTGTGTCATTTC
TCCCTTTCAGAAGAGCTTCGGTGCCTGCCTTCACCAGCGGCTCAGACTTTC
CTTTGTCCTCGGTCGTCTGAAGAAAAGGAATTCTTCATTTCAGGGAAAGAT
TTAAACACCTAAAAGCAGAAAGCCCTAATATACTTCTGTGTCCCAAAATT
CTGCCTTCATCTTTGTCCTGCTGGCTTCATCCCCCACTTTGCTTGCTGCCCT
AGCAAACTCACAAACGCAATTATGTCTAATAGAGAAACTTTTTTTTTTTT
TAGCTTTTTGAGACAGTGTCTCAGTGTAGCCCTGGCTGGTCTTAAACTCAC
TCTGTGGATCAGGCCAGTCTTGAACTCACAGAGATCCACCCGCCCCTGCCT
TCCAAGGGCTGGGATTAACGGTGTGTACCACCAGGCCTAGCTAATGGAAG
GCTCACCACGATTTGATTCTCTGTCCCAAGTTACTCATCTGCTGGCCAGCT
GAGTACCTTCCTAGTCCTCTGTTCCCTTTCTCCTACTTTTCTGTGCATGGTG
TCGTGTCGCCCTGGAGATCTTCCTGTAACCTTACACTCAGGGCCTGTCTCC
TCAACTCTCTACGGCCTGAAAGTGATCCTTCTTAAAGATGGGCCAGGCTGT
TTGGCAGTGATGCCCCTGGAGTAGGACATTTAATCTGCCCATGGCTGCTGG
CCGCACTTGTGGCCTCTAACCTTATTTGGCTTAGTTTCTGGGTCCCTGTCCT
TTCCGGCCAGCTCAGACCTCCTGGGGTGCTCCTCACTCACTGTGACTTTGT
GCAGAGCATGTGGCATCCGTACTCGTGGATGATGGCCATCGCCTGCCTGG
GAGCTTCGAGAGGGAATAGATCAGGCCAGTCTGCCCTGCTGTGTCCCCAG
CACCACCCAGACCACCCACCATGGGGCCCAGCACTCAGTGCTCAGGAGCC
AAGTATGGAGTGAGTGAAGTTAGGAGGGTGGGTGGGTGGGTTGATAGAT
GGATGCTTGTCAAGCTCAAAAGTTAGGCACTTACCATGAAAGCAATAGGC
AGCCACAGAAGTGTTGTCAGCAGAACTGGCCCATTTCCTCAGTCTTGAAT

GTGCATTTGAGTTCTCTGGGATCTGGGAAGGGCCCAAGATACCACACTTCT
AAGGGGCCCAAGATATGATGGCCTGAGACAGCAAGGGCCTTGCTGTGCTG
TTTAGCCAGTAGCAACCAGAACTAGTCAGTGTCTTAGAGCACTTGACAAT
GTGGCTCGTGTGAATTAAGATATATTTTAAGTGTAAAATGCCCACCTGATT
TGAAAGATGTGGTACAAAAAATTTTTATATTGCTCACATGTTGATATGGTA
ATATTTTGGATATCTTGGGTTAAATAAAATATATTATTAAAATTAACTTAT
ATTTGTTTTTATTAAGTGTCTACTAGAAAATTTAAACTTAGGGCCATCAAA
ATGGCTTATCAGGTGAAAATGCTTGCCCCAAACCTGCCAAGCTGAGCTCA
AGCCCCAGAACCCCCAAGTTGTCCTCTGTCCACATAGGTGCTGTGTAGCGC
CTGCCGACTACACATACAAAGATGGATTTGGTTTTGTTTTTGCAGGGATAG
AAGAGTATGATAAGTTTTTAATGCAATAAATTTTTAATAAGATTCAAATAA
CACTGGCTACTTGTGTTTTGGATGTACTCCTGCTGAGTCACTCTGGTCCTC
AGTGCTCTGAATTCCCTGTCACCACAGCTTCCAAGACTGCCCTCTCTCTGG
GGCATCCTTCTCCTTTTTCCCTGTAACCAAGTCCAGGTGTCTGAATTTGCTG
TTAGCATGTCTCTTCCTAGGTAACTGTGGCATATGCCTGTACTCCCTGCTCT
CTGGAAGGCTGAGGCAGGAAGATCGAAATTTTGAGGCCACCGTGGGCTAC
ATGAGATCCTGTCTCACACAAGAGGTCCTTATCCAGAGACTTCCCTGGTAG
TCCTGCCAGAGACGCTTCCAGGAGCATTGTGGGTTTCCACAGTTCTGTGAG
TTCCCCCATCCCAACTCTGGCCACTGTCAGCATCTATGTATGAGCTTCACA
TGTGGAGTGTGTGCATTTTATGTACATGTGTGTAGAAGCCAGAGTTGGCAT
CAAGGGTCCCTCGCTTGAACCCAGAGCTCACTGACATGGCTACTCTGACT
AGTGAGCTTGCTCCAGGGAGCTCCAACCTGCCTCCTGGTCGCTGGGATTAT
AGGCAGCTACGACTACCCAGTTTAAAGTCACCTAAAAGTTTCTGTTGGTTA
GGGCTGGTTAAATGATGACTGAGAACGCTTGCTCTTCTCGTAGAAGACAT
GGGCTTGGCCCCAAGTTCCCGCATGACTGTTCACCTTCTGTAAATCCAGTC
AGTACAAGGGACCTGACCCACTCTTCTGACCAAAGGAACGGGTGTAGTAC
ATAAGCAT
>LNC_000249
CTCCAGTGCCTGCTTCAGCAGGTTCAACAGCTTCTTCAGTGTCTGCCCCAT
GTTTTCATTCTGTTCCTTTTCCTGGGACTCCTGGATCTGCAGCAGCTCCTGG
GTGGATGGTCCCGGAGGCAGACTGGTCTTCACACTGAGATTTCCTCCTCGG
AGACCCCACTGGTCTTGGAGAAACAGAAGATCTTGTCGGAGAGAGGCCGC
CCTTGGAAGCAGTAGTTCGGGTGCTTTCCCTACAGGCAGTCCCAGTGTGTG
ACTCAGCA
>LNC_000344
GGAAAGATTTTTAAAAGTCCATCAACGAAGGTTTGTTGTTATTTAACGCTA
AGTATCAAAGTAACTCACCGGAGCAAGCAGGGGTTCCACAGAGCGGCCG
CCGCCCTCTCCCCGCTGGCTCGGACCGCAGCGCGGCGCCGGGTTGGGTGT
GCAGCAGGAACCGGGTGGCCCGACCCGGTGCTCCAGTGGCATCGGCCCTC
CGCACCCAGGGCGGGTCGAAGCGCGGCCGCAGGAGCCATCGGCTTGCTCT
TCCTCGCCCGTCCAGGAAGGGTCGCCCGCCGCAGCATCCCGCTCACGGCG
CGAGCCGCCTCCGGGCCGCGCGCGCCACCAGCACCGCCGCTGCCGCAGCG
GAGCCACGGGCGTCGTGGGCCAGGCCTCCCCGGGCGCCTCTGCGGCGAGC
TCCCGCATTCGCACGCGACCGCCTCGCCTCAGCCGTGACCTCCCCGCTATT

CCGGAGCCCTCCGGGACGCCCAGGAACTGCTCCCAGCGCAGCGGCGAGCC
CTTGGGCCGCGGCGGGCCGCCTGACAGCTGGAGTTGCTGTTGCCTCTCCG
GCCATCTTGTGCCGTGTGTGGTGTCTCTCCAGTCTCCTCGCTCGGCTTTACT
CCCTCCTCCTCCTCCCCCCGCCCCACACACCACACAACATGGCCTCTTCGC
TGCGGCGGTAGCTGCGGCGG
>LNC_000356
GCTTCATTCACAGCTGCGGCCTGCCTGTGGAGTCTAGCCTCTCTCTCTATG
CTGAATGGGTCTTTAGGAGCTCCGAGAAGGTCCCAAGATGGCCACACAGA
AGTTCCTGGCCATCTCTTTGGAGCACTGGTTGGAGAGGGAGCAACTGCAG
CAAGAGCAGCTTGCTCTTGGTCCATTCGAGACCCAACTCCCATCTTTAGAG
GGTCTCTGGGACCATTTCCCGTCATAGAAAGAAAGGGCAGAGGAGGGGA
AATGCGAAGGCTTGAAATCAAATCTGAGAGATGATCTGATCCAGAGCTGA
AGCCACTCGTGTCTGAGTCAGAGGGGCTGCTAGAACGGGAGTCCAGGATA
GGCCGGGTGTCCAGGCGAGATCCTCTAACTGAGGGCGCTGGGAACTTGTC
CAAGTCAGACCCAAGGGGATCCAGAGACAGGAAGCTCAAGGGGGTTTTTC
CTAAGACATTTCCCAGTGGATTTTGGAGCATGCTTAATACTGACTGTGTGT
TGCTCTGGGCTGAGGAGTCTGAGTCCTGGGTGCTCCAGGGTCGGTCCCAG
CCAGTCAGACTGAGTGACTGCAGGCCAAGGCACAAACCGTTTGCATCTGG
GAGGCCGCGGGGAGATTCTTGCGCAGAGGTGGGGAAAAGCATCCTGCTTG
TAATTGCTTCTTCAGAGTCCTGGAAGTCTGGCAACTGATCATGAATTCCTC
GATTTATAGGTGTTGTGCAGACTTTACTGAAGTCCAGTGAATCATCCAAAA
TGGCATTTATCCTTCGAAAGATATTGGCATTGCTACATGTAGAGAGTGCAG
GAACCTCTTGGTTGTCCCAGCAGTCCTTTATCCTTCCTGATTCTTCTTCCTG
GTCCGGTCCACCCTCAGGGCTAGGGCTCCAGAAGCAATCGAACGTGTGAA
GCCACGTGACTTCCCCGGGCTCGGAAAGCGCGCGGCCCGCACGAGGGGCC
CGAATACTCAGCTTTAAAGGAGCCATGTCGGCGCCGTCGTGGCGCGCCTC
CCCCAGCGCGGACCCAGCCTGAGGGGGTCTCCGCTGCTCACTGTGCGGCC
CGCAGCGAGCCGCCGGCTCCTGTCTCCTGCGGGACGCCAAGGCGACTCTC
GAGAGACGCCGGCACTGCCGGCCGCTCGGGCTCTCACGTGCGACGGCTGC
GGGAGGAGGAACTGTTGTGGGCGCCCGGGCTCCCGTCCGCGCCGCCAGCT
CCGGTCTGGACGTATGTTCGAAGCCACCGAGAGTCTGCAAGCGCAGCTCC
GAGGCCCCAGCGGCCGCCCCCGGAGGGCCGCGGCGCCTTTAAGCCCAAAG
GCCTGAGTCACGAGGAGGCTCCCTGCCCCTCCCGCGCGCCCCACACCGCT
CCACGAGGCCCACGACCGCGGGCATGTGACCGGCGCCCCGCCCGGGACGC
TAACCCGGGCGGCGGTGGGGGCCTGTGAGGCAGCGAGACTGCGCGCGGA
CCGTTAGCTGAGGCGGCAGGGACAGCAGCCCCGCCCGGCCCCGCCCCGCC
CCGGCGGCTCTTACCAGAGAAAAAGCCATGGGGTCCGGCGTGGCCCTAAG
GGACTGGCGGGCGGGCCGGCGGGTCTGTGCGGCGCGGGACGCGCACACA
CGTGGGCACTATTTTTGCAGCGAGCGGCGCGCGACTCCGCACACCGGCGG
CTTATAAAGCTCCGCGGCGCTGGCCTGATGAGGAGCACCTGTGTCGGCCC
CGCCCGGCCTCGTCCCCGCCCCCGCCCCCTGCGCTCCTTCCTGCCGTCCAT
ACCGGCGCTCCACCGCCCACCCTGGGCCTGGGGGTCCGAACCGGGGCTAG
TTCTGAAGCTCGAACCGATGCAGGCCACCGCTGCCTCCGGGAGTCCAAGG
GTTCAGGCCTGAGCACACCCCTCGCCTAGTGACCCTGGCCAAGTCACTCTC

CCGCAGGCCTCAGTTTCCCTACAGATTTACAAGACCCTGCGCTGCAGCTGC
GCTGGTGACGTGCGGCCCCCTTAAAACCGTATAACCACGCCTCTCCTTTTC
TAGTGACCTCTCCGGTGGAAGCCCCGTTGCACCGAACCCAGCCCCTCCAC
CCACCCCCACCTGCAGCAGACACGCCCTCACTTCAGACCGACTGCAACTG
CATCTGGAGCGAGCTGGAACTGGGAGCGCAGGCGTCAAGGGATTCGGGA
AAGTTCCGATGACAGCAGTGATCCTCTTCGGATTGCAGTTCCCCGGGGAA
ACTGCTTCCGACGCTCCCTCCAGATAGGATAGGACGCTTTACTGAGAAAA
TTCGGGGCAAGAGATCAGGGTGAGCTGAGCTGTTGTTCAGAAAGGCGTCA
TGGGCCAGCCAGGGCTGGACCGGACCGGAAGCCCAAGCGAGCGCAGAGG
CGGAGATGGCATTCCGGGCTGGAGACCAGGGAAGGCGGAGAGGGAGGAA
TGCACTGAGCGGCTCAATCTAACTAGGACCAGCTAATGCGTGTTGATGCC
ACAGGAGGGAAGCCGGAGCCCGGTTTATGATGGGCTTTTATTCCGCAGCT
AGCTGGAAACTAGTGAATGGTGAATGGAGCCTGGAAGTTGTCAGGTCTCT
TTTAAGGTGTTGCTGGAGACTGGGAGGTTGGAGATAAAGGCACTGGAGTG
GATAAGAAAGAGACCAAGAGTGTATTGAACATTTCTAAGTGTGTTATATG
TTATACATTTTATTCAGCATGCATTACACTCAAGGTTCCTCCTGACAGGTG
GGGAAGAAGCTGTTAGCAACTTGCTGGCAGGTTTATGATGCAGGAAGTTT
CAGCCCAGGGTATTAAGAAAGGAAGCCTATGGCCCTAGTCTAGTGTAATG
TTTGGAGGAGAAGATGGGAAAGAGGACGGATGCTGGAGATGAAGTCCAG
AGGGAGCTGAATAACAGCGAGAGCTGGTTAGTCTCCATTCTGGGGTTGTT
CCCAAGGGTTGCTGAAACCCTAAGCAGAAAGGGTGGTGAGTATGGAGAA
CCCATCCCCTTAGGAGCAGTCCAACTACAACCCCATCTTGGCAGCATAGG
CCTGATACAGTAAACCTAATATTTCTTGCTTTTCTTCTCTTCCTATTCTGAA
AGGGTGCCATACCAAGATGCTTATGACATCAGAACTGTGAGGCTGTGTGA
GATGTTCTGAAGGCCCAAGGTACATCCAGAAAGACTGATAACCCATCCCT
GGTGGCTTAGGCCCATTACTGCAACACTATCACAGAAGAGGCAGAAGGAG
GGGTGCCTTGCATTGGAAGGCAGTCAAAAACTACAGTCTGAAGAAAGTCT
GCCTCAAATAAATGAAGAAACACATAAATAGAAGGGGTGGGTTAGCTGA
TAGGCATCACACACCTCTAAAATCTCTCATATGAACCATAAGTCCAAGGA
CACCATGCACCTTAGTGGCTGCCAATTTGTGGTCCTTCGCATCCAGTCCTC
CCTGGCCCCAAAGTTAAACGAGATAAGAATCTAAGACATGAAATGGTTCC
TAAGGGCCTATGGGAATGCATATCTGAAAAATATAGTTTCAAGCTCCCAG
CCTATAAGAAAGTAACTTCAAACACCAATTTCTGCTGCTACATCACCATCC
CTAAACCTAGCCGAAGGTGGTAAGAATGGAGTATTTTCTGGGCACTCAAC
CATGTGCTTAGCACACTTCTAAGAGCTTTGCTTGTGTTAGCTTGTTTTGTAG
AAGTAGCATGCTTGGCTTCAGCTGTTTGTGAGACTAAGGCAGGAGAGAGG
ATTGCAAATTTAAAGCCAGCCTGGACAGCTTAGCCTGTGTGTCCCCACCCT
ACACCCCCTAACCACACTCCCAGACTCGAGTAGAGTTGGGAATATAATCA
ATGGGTGTGCTCGTCTAGTACATACAGGACTCCAGGGCCAACCTCCAGAA
TTCTACAAAAGAAGGACCCCTTGCCAACAGGAGCAAATCAAGAACTCAGA
CCTATGGTTTTCCATTTTCATACTTTCAGACGAAAGTAAAACCTCTGGCAT
CTCCAGCTTCCTTTCATATTTTACATTTATCATTTACTAGCGTGATATGGAC
ACAAGTGGCGGTCAGAAGACAACTCACAGGAGTCAGCTCTCTCCTTCCAC
TGTGTGGGCAGTAGGGCTTGAGCTCAGGTCATCAAGCTCGGTGGCAAACG

CCTCCACCTGGTGAGCCGTCCTGCCCTGCCTCACCCCACCCCCAGTTTTG
AACAGGTTCTCAATATGTTTGTAGTCTACATTGTCCTGAGTCCTTGATCCT
CCTGCCTCAGCATGTTACAGTGTGTGTGTGTGCACACGTGCTTGTGTGT
GCACAGACTAATGAATTTTTTTAAAGTACAGAGGATCAGCCCCACCCTTA
TCATATATAAATGTAAATTTTAAATAGGTTAAATATATAAATGTGAAAAG
TTGTTTTGGGATTTTAAAGAGGAATATAGGAGGACAACTTTTTTAATTTGT
TAAAGTCTAGGCTCACAACCAAACTATTTCAATTTGTTGTTTTGGTTTGTT
GAGACAGAGTCTCACCATGTAGCCCAGGATAGTCTTGAACTGAGATTCTC
TTGCTTTTACCTAAGGTAAAATTCAGAGGGTTATAAGTCTGTGCTATCTGG
CCTTGGAGGAGAACATTTTTATTGCCTCAGTCTGTGGACAAATTCTTCAAA
TTAAAAACAATAATAAAACATGAAGATGATAAGTTTGACCACAGTATAGT
TTAAAACTTTATGAAAATATATAAAATTGGAAATGCATACCAAGTCTTCA
CAGATCTAAGATAGCTTGGATATCTAAGAACTTCTATTCTCCACAATGCAG
TCAATCAATATGTTCTGTGAGCAAAGTTCCCCAAG
>LNC_000420
CGGAGTTCTGCAGCATGTTCTCGAGTTTGGCAAAGAGCACAGCCATGAAG
GGGCCGGTGAAGGGCTGGCTGGGCAGCTGGTGGAGAGTCCGCAAAGGGC
TGCTGAGGAAGGGGCCAGGGGGCTCAGGAGGGCAGGTGAAGCTCTCGTA
GGCCTCCTCCTCCTCAGGAGGTAGTGGAGGCTCTAAGGGAGGCTCTGAGC
CAGCTCCACCATTGCTCAATGCCACCTCCAGCTCCTGGAGCTCTTGCCCAA
AGCTCTCTAGTCCTGCCATGCCCTCAGAGGTACCCTCTAACAATTCCCTAA
CTCCTTCCTGTGACACCAATCGTGGCACCAGACGAACCTTCTTGGCTCCTT
CTGGCCATGGCCCTGGTACCCCATTGAGCTGGGGTGGGGGTAGGTAGCCA
GGAGTATCCCCTACACCCACAGCTAGCCCCCGACTCCCTAGGTTTTCCTCC
TCTCCTTCCCTCACATTGTCCCTGTCTTCCTCTGGCAGTAGGCTGCGTTTCT
TAGTCCGGGAGCCAAGAGGGTTAGGCTCAGGTGGGGGACGCTCGCCATCA
TAAGGGGCAGACCAGGTTCGGCAGGCTCGGACACAGCGGTCTACACCACG
GCGTGCCTCACGCAGATACTCCAGGTAATTGTCTTCTAGCTCACCAGGCTC
CTCTGCAGGACTGGGTCTTCGACCAGGGCTGGAGGTTGGTGATGCAGTAA
GCCCTGGGGATCGAGGAGCTGGACCTGGGGACTCAGAGCCACCTAGGCTC
TGCTGACGGAGAAACAAAGCCAGACGAGATGGTGTGGAGGGCCGGGGCA
CTGTTACCACGGATGAAGAATCAACACTGGGGCTTCCAGGGCCTCGTGCC
CACGAGGCATGCTCAGGACGAGGTGGGCTAGGGGCATGGTGTCGACAGC
AGCGTGGGATTAGGGAGAGAAACTTGTCGGCTGCCCGTCCATAAAGATCC
ACATCACGCACAGCTGGCTTCTGGCTCAGCATCACATGGTTACATGGGAC
>LNC_000478
TGAACAGATCTGTTTATTGTGAAAATGGTCTTGGTACATATGAGGAACTTT
ATATACTTTGAGGACAGCATTCAGAACTGTTAAGATAAAAGACAAACTAT
AATTTTAAACTCATTCTTGATTTAGGGGAAAGGCTATAAATTACGGGCTGG
AATGAATTCAGCATGAACTCAAATTCTACAAGCATAAAGCATGAAAGAGT
ACAAAAACGTCGGAGTCCAATCAGCCCACACCTAAATTTTAACCTTACAC
TTTCGAGGACCGTTTGTTCATTCTTAGAGACTAGTCTTCTTTTGAGACAGG
TTGATTTTATCTCCAAGGCTCAAGGCCATTCCCTGACTTTTTGCACGAATT
CTTATATGGCCAGTAACAGCAGCATCTGGTCCCTGGTGAACTGGCTTCTCA

ATGCTGACATTTGCAAGTGCATCCTCTCCAAATATAGAGCGAGCATATAG
GTTGGCTGCCATGAAGCCACAGTAGCCAGAAAGGGCCTTCTCTGGAGTCA
GGCACTTCATGTTGGTGGACTTGAGGATGTGCTGTAAATAATCATTCAGGT
CAGTCACGTTGGTGTTAACTGTCACTTTATTTTCCCATTCAAATTCGGCCC
ACATCTGACGGAACTCAGCATCAGTGCAAGTTGCAGGCTGGATATAGTCC
ATGATGTCAATGTGGATGTCACTGAGGACCACACAGTTTCTGTCACTTGCT
GCTCCAGAGACATCATAAACTATATTGCCGAAAATTATTCCATTTTCTGTT
GATGCTACTTTGACATTGGCTTTAATATTCGCAAAGTCATGAGGAGCAAG
AGTCAAAGGAGATGGTTTTTCCACGAGTTTCAGATCCCCTAGAGTAGCTA
ACTCTAATGTGCAGTTCTGCAAAGTATCACTGGTTTGGTTTACAACAAGAA
CATCCAGGACAATATCATACTGATTGACATGAACATAAGCTTCTGCATAC
ACTGGATCAGAGAAACCTGTCAACTGAGTGACCTTGTTGAGTTTGGACGC
CAGGGGGTCTGCTGCCTCTTTCCTCTGAGTGTTACCCATCGCTGCCAACAA
ACTCAGCTGGAACTGATCCTCCTTGCAGTTCATCTCATTCTTAGCAGTTAG
TTGCATGAAGGAAATGGGGTCATCAGGCTGTACTGTTACATTCCTCTTTTC
AGATTCTTTCTTTTGGGATAGTTTCTCTTCTTCGAGTTTGGCAGACAACATT
TGGGAAAGAGACTGTCTGCACTCCTTATTAAAAATGTCATTCATTAAAGGT
GAGCATTCAGATAAGACCTTGAGGCACAGGGAAATTCGGTCTACATCATC
ATCAGTGATTGGCTTCTTAGGAAGAGAGGATTTTCCCAAATGAAGGATGG
TTGCCATAAGCAACATAGCCTCAGCAACAAAAGAGTTTTGCTTTTTCTTCT
CCTGAACCAATGCTACATAGCGTAATGCAATCTTGGTCAGAGTTGTGGCA
AGGGAGGCAGCAACAAAGAAATCTCCATCCAGGAGGAATCCTCTCAAGG
GTGGTCTGTCCTCTTCTTTCTTGGTAGGTCTAGAGCTGCTGAGTGCACTCT
GAGTAGCATAGGTGCCCATCTCTGTAACCAATTTCTGAACGGGCCCAACA
GTTATTTCTTCTTCTGGTTTTAACTCGCCAGCTTCTTTCTTTATTTCTGATTC
TACGATTGGGATCTCCCCAAGGGACCTGCGGACTTCAGTCATCACACTCTG
AATGTCTTCCTTAGTACTGCAGTACTCGCCCAGGATCCACAGTGCTCCTCG
ATAAATCTTGACAGACTTAATAGCATGGAAGACTTCCAGCATCTTCTCAAC
AATCAGCATTCTCAGGTTGTCAAAGCGCTGAATGGCTTCACGCACAAACT
CCAAGACATCAGCAGCTGCTGCTTCATTACTGTCACTGAGAAATTCCATTA
GCACAGGAATAACATTTGCAGCCATATCTGGAAATCGGACAGAGCAGGA
ATGCAGTGTTCGCACGAGAAGCTGTCGGTATTTGTCAGTGTCTTCATGCTC
AGACACGTTATTTGTTTTAATTACTTCCTTCTTCAAGACAATAACCAACTC
TTCAACATTCCTAGATGAGACAAGATCCAGTGCTAACTGCAGCGTTTTCTT
GCGCACTTCTAGGTCTGGTGTACTCAGTACTCGTAGGATGTCCATGACCAG
ATCTTGGAGGACTCGCTCGTGAGCAGGATGCTCTTTTAGTTCTACCAATCG
GTCCAGGACAATGAGCTTTACATTGTTATCACTTTCCTTAATAATTAAATC
AATGTAACACTGAGCAGCAGCCTTTATTGCAGTTGGAGCACTTGACAGTG
TCACCAGTGTCCCAGCAGCTTCGTATTTTACAGCAGGACTAGATGACTGCA
GTAAGTTATAGATACAGCGAATGAAACGGGCCCTCTCTGATGGATTAGCA
TGACAGACCTTATAAATTAGTTCAACAATAACCAACTGTAGAATGTCTCC
AAATGTCTGAACTTGATCAATACATGTACTTAAATAATCCAAAGCTCGATC
CTGATCTGCATGAATGAGCATCATAAATGCATTTCTTTTGCAGCTTGCATC
CTTCTCGTTTACCAGAAAATCATGTATCAGCTCAGGAGCATCAGGTATAA

GATTTTCAAAATTTCTGTAGATGGTGTAGATGGCCAAAACAGCGTTTCTCC
TAACATAGCTGTGACGATGTTCCAAACAAGCACGGATAGCAGGCATCAGA
GGTTCCAGCAATTCTGCTTCCTTCAATTTGCAAAGGAAACGAAGAGTAGA
TCCACGAATAAACTCATTAGGATGCTGGAGATCCTTTCTGTATGCATCACA
CACAAGAATCATTTCATGTAAGAGCCTCCCATCTGGAGTTGTTTTCGGAAC
AATTTCCCAGAAGACCAGAAGCAACTTTTTGATGGTGTGATCCTGAAGAG
GCAGCACGAAACGAATGATCGTCATCAGGAGTCCAGGAAGCTTTTCCCCA
TTCAGAATCATAATGATCACTTTCTTCAAAGCTTCAGTCTTTGACTTCACA
TCGCCTTTCTCCAGGTCATTTTTTAAGCTGATTTCAGAAGGGGGTTCTGAG
TCCATTGGCACGTTAATTAACGTATAGCACACGTTCTCAGCTGCGGTCATG
GTTCCTGTTTATAACCAACTCAAAACAAGATGAAAAGGACGAAAGGTGCC
AGGTAAATCTGGCTCCGTGGGGGTGGGGGTCGGGAAGGAACCGTAGGAC
CGTCGACTACCGGTCCAGACAGCCGCTGCCGCGACGGCTCCACTGAGTCC
TTGGCTGACGTGGAAGGAGGCGGGGAGGAACTAG
>LNC_000488
TTTTTTTTTTTTAATTTTTCCCCTTTTACCACAAAACAAAGTAGAAGAAATG
ATTAAAACTGCCAAAGTAGTTAACTAGTAGAACATGTATTAGTCTCACAC
ACACATATACATGTACACAGGAAGGAAGGCAGGCTTATTTACAAGAAAAC
GTGTAAAATCAAAGTGGGTGTCAGGAAACATTGAAAAACAAACACATAC
ATGCTACAAGAGGCACCACTGAGTACAGTGCTAGGGAGGGGAGTGAACA
GAGGCAGACAGACAGGTTCAGTCTTCACAGCATCAGTGCAATGGATCCAC
AAACCATGTTACAGCTAGTTCATGGGTTAAGGAGCTGTTCCCAAATGTGTC
CTATTTGGCCCTCAGAGGTTGAGTTCTGCAGATTCCGACTGCTCTAAAAGC
CTACCTACTGAGAGGGCACATGATCACAGTAAGCTTAAGGAGTTGCAAAA
GCTATGCAGACCAAAGTCACCGATCAGCAGTCTGCTCTCAGCTGCAGCCC
TGCATTTTTCTGAGAAATATCAAGGGGAAAGTCAAACACCAGTAAACACT
GTCTCTGAAGTGCAAAGCTGGAGTGACTGAAATACAGCCAATACTACGAA
AACAAATCAGATTTTCCAGGCTTTTAAAGCCTTGTTTTTCTGGTCTTAAAT
ATTAATAGATAATCTAGTTTTCTACTCAGTGAACAAAGGATACTTTTATCC
TTGGCTGGCTTAAAGTCACGAACATTTTAAGTCAGCTTAAATCCAGAACA
AAAACACTGCTAAAATTCCCAAATACAATCTTCTAGAAGCTTGTTGCTCAA
AAGCCATTATGGACTAGGACTAACCTTAAGCAGTGCAAGGGGAATGTGAG
GTCAGTCAAAGCCTGTCAGATTCCTCGAGGGAACAAAGGGGCAATTTGCT
CCCTGAAACTGTTGAGTTTCGTACGAGAGAGGAGCTTCCACACACTTGGG
TGAGGAATTAATTTTATCTTGTTCCATGGGTATTCTCCCACGGTGGAAAAT
CAGGAATGAATCTTAAGTGGTCTCTTCATCAGAAGTGGTAAACCTGACCT
CTGTACTCAAAGACAGTCTGTTAAGCAGACTGTGGAAGCAGACAGTAGAA
CCAGCTTCCTGTAGCCACAGACCACTACCTTGAATATAGCTAAAGCAAAC
ATCGACAATTATGCAAAGTCACAGTTCACTGTACAGAACAGTAGAGTTCA
CCACTTTAGTATTTTAAAGGAAGTTGTCAAAAATAAAAACTCAATAAAAC
AAGTCGTACTGACGGGAATGTACCCAGCTACACCTGGCACTAGACACAGC
ACGGAGCCTGGCCTGTGAGAATCTCTATTAGCAAGGGCAGAATGAGGGAA
GCAAAGAGCCACTGCAACATTTCAGTGTCCAGCCAGCACCGGACAACTGT
CCCCAGGGCCAGGGCACACTCCACGCAGCCACACTATTCGTTTTTCTTTTC

TTTTTGCTTGAGAAGCTTGTTTATCTCCCTTTTGGCCATTCCAATGTACTTC
AAGATGATTCCATGTTGGTTTAGTCCAGGAAGCAACAGCACAAAAGTCAC
AATCAGGTAGGTGAGAAGCAGGTTGTGGACTTGCTGTCCCACCCAAGCAA
CCGCAGCAAGAGAAATGATCATGGTCATGAAGTACATTTTAGGCTTTTCTT
CCTTTAGCGTAAAGAGGCGTTTCCACCAGCCCACAGCTCTGCGTCGAGTTT
TTACTAGATTACTGCAGATTTCATGAAATCTTTGCTGTTGTTCAGTGGTCC
ATTTATTAGAGCCAAAAATCCTTGGTGCTAGAATGGGAACAAGGTAGTCA
GCCAGGCACAAAAACATGACAAAACAGGAAACACCGGACAGCACAGATG
GATCGAGATAATAGATAATCAGGAACAGCAGGGAGACCACACCCATAAT
GGCAGGTGGAAACCAGGCTCTTTCCCACCGAAGGACTTTGTCAGCCATCA
GCATCACTTCTCCCCAGCCTTGCAGCTGCTCTTCAAGACTTGCAGTCTCCA
CGGCCAGCAGGTTGCTGCTGCGGTTATCCCCCTCGGCCATCGTTCCAGACG
CACGGTTATCAACGAGCGCGAACACACCAAACCCAACACCAGCACGGTA
ACCC
>LNC_000598
GGACAATGTCTTGTTGAAGGTGAGCTGGGCAGAATAAATAGACTGTGCCC
CCATGATGGGGATGAGGAAGGCCCTGACCAGACCAAGGCAGGCCAAGCC
AGGTGCTGAGTGTCCATGGCCCAAGCCAGGAGCAGGGGGAGAAGCCCAG
CTGCCGCCTGGAGCCTAGCTGTGTAGGGCAGGGGAGGATCCCCGATGGCA
GCTTCCCAGTCGAAGACTTGCCCTGGTCTAGTCTTGCCAGAATCTTGCCAC
AAGGGCCTGAGAGTTCTGCTGCCAAGACTACCATCTTGCCTTCTGGTCTCC
CCTCCATGAAAGCTGTTCCCATGGGGCTGGTAGGGAGTCCTAGAGCCACG
CTGGCAAGGCCACGGGGAGGCATGGGCCTCCCTGGGCCCCTGGGCAGAA
GGGATGGAGGGGCTGATAGCTCTGGGTCTAGCTTCTCCTGTTCTAGATTTA
TCTAGCTTTGACCCCCACTGGCAGCCACCTGGGGCGTAGGCCTTGGGACA
GGGGCTTCCTTGGCCCCTTGGGTCCCCTGTAGCCGGCGTAACCGCAGTTCC
TCCAAGCCTCGCCAAAGTTCCTGCCGCTCCTGGGCCTTCTGCTGCTCCTGC
TGTTTTTCTAGCTTGTAGGAGGCTGTAAGCTCATCAAACAGCTTCCCATTC
ATCTCCATGAAAGTCTTGAGCACGTTGTAGATCAGGGACACGATGGTTTG
ATTCCAGTGCTCCTTGGACACTTGGTAGAGGGTCCCAAATACCGCAGGCA
GCACAGTGTGGCAGTTGTCCTCAATGAGGCTCAGGATGTACTCATTGTTCC
AGAAATACAGAGCCCGCTCTGCAACCTGGAAATGGGGGCTGGAGACACA
GCGAGCCACCTGCTTGAAGAGAGGCTCCTGGATCTTCACAAACTGGGAGG
GCTCGATCACATCGAGAATCTCTTCCATCTCCCCCAGGAACATCACCTCCT
TCTGGGTGCAGGTTTTAGGCCAGTATTTGAGAAGCCCCCGGATAACATGC
TCTGTCAAGGTCGCATCCTTCTCCAGGAACTGCACC
>LNC_000641
ACAAAAACAAAAAAAAAAAAAAAAAAAAAACACAAACAAACAAACAAA
AGAGCAAGATGTGACTCTTACCATGGACTTCTTCACATTCTTGTAGTATGG
ATTCCATCCTATGCTCACCACCATTTTATGGACCTCTCCACTCCCAACGCT
GGCCCAGCCGTAATAGATGCCAGTGGACACATCAGCTGGAAGATTGTCTA
CTACTTGTTCAGGAAAATTGGCTGTGGGGATGCCCAGTTGCTTGGAGCCG
CGGCCGAAGCCACGCACCACCTGACCTCGGCAGAAGAACGGCAGGCTCCT
CATGACGCTGACCTCGGAGGACGCGGGCTGCTCCTCGCTCCGTGCCTGCG

ACGGAGCCGCCGTGAGTGCGGCACTCCGGCCCGGACCAGCCGGGGATTCC
GCAGGAGCTGGGCCGGCGGAGCGGGACACCCGCCGCTACCGGTGCCTGCC
GGCAGGACAGCCGCCAGCCGCCAAGAACCCAGAGGTCTCTCGGTCGGAAT
TGCGACAACCGCAGAGAACGCACGGATCTGCGGCCCAGGCGACCGGCCT
GCTCGGCTTCCGTTGC
>LNC_000770
TGGTGCAGTGTTATCCGGGGACGCTCCTGAATGAGACCTTTGACGAGCTC
CATTGCTCCCAATCTGCTCCGGGTCGGAAGGGAAAGAGTCCGAGCTACTG
TACCCATCTACGGTATTTCCAGAAGGAAATTTTACTGATGAATTGATTTA
TTTTCTTCTGAAAGATCAGATGGCTTCACAAGTAATGGGCTGGTGGGGTTA
GTATGATCACCGCTGCTTCTTTTCAGCTCCATCTCCTGCATGTGGATTTTGC
TTGGAGTCATACGAATGGGAACTGGATGCACAATGGCGGTATCCTCACTG
CTCTGATTCAGCTCTGGCCAAGTCTGGTTCAGTGACGGCATCGACGGTGAC
TTGCTCGGAGGTGCTTCTGCAGGAGAGCCTGGATAGCCTACCTCACCTGG
CTGGTCC
>LNC_000846
TGCAGACACTGCTGCAAAGGCTACATTGATTTCAAACACGTCAACATCCT
CCAGGGATCAGCCTGCATTTGCAACAGCTTGCTTTATGGCTGGAATCGGTC
CTACTCCCATGACAGAAGGCTCCACACCGGCTTGTGACCAGGAGACCACT
TGTGCTAAAGGTTTCAGCATCCGACTCTCAGCTTCCGTCTTCTTCATAAGA
ACCACAGCAGCAGCACCATCGTTCATTCCTGATGCATTCGCTGGGGTGAC
CGTTCCAGTCCCATCAGTAAGAAAGTAAGGCTTCAGCTTGCTCATGGCTTC
AAGGTTACTCCCATGACGAGGAAACTCATCGATTTTCACTTCAGTAAGAC
CTTTTCTAGAAGACACGTGCACTGGCACAATCTCCTTGTCAAAGTGGCCAG
CTTTCTGCGCATGCTCCGCCCTATTCTGGGACACAACAGCAACCTTGTCCT
GGGCCTCTCTGCTCACTTGCCATTTTTTGGCTACGTTTTCAGCTGTGATGCC
CATGTGGTAGTTGTGAAACGCATCTGTGAGGCCATCACAGAGGATGCTGT
CGGCGAGTGGCACCTCACCCATCTTCACTCCTGATCTCAGGTGAGCCAAGT
GAGGGGCCTTGCTCATGTTCTCCATGCCTCCAGCAACCACGATGGTGGAG
TCACCCATGGCTATGGACTGAGCTGCAAGGCACACGGCTTTTAGGCCCGA
GCCACAGATCATCTGGCAGCTCCATGCTGGCACCGAGTAGGGGATCCCCG
CACCCACACTGGCTTGTCGAGTAGGATTCTGCCCACAGCCTGCAGTCAAA
ACGTGTCCAAATATGACCTCGGACACCTCTTCTGGAGCCACTTTGGCTCTC
TGAAGGACTTCTTTGATAACAGTTGTCCCCAGGTTGTGGACAGGCACGGT
GGACAGGGCACCATTGAAGGAACCTATGGCGGTCCGCGCCGCTGAGATGA
TGACGACGGGGTCCGAGCCTGCATTCATCCTGTAGCTCCGAGGTCTGCTA
GCTCTCAGCCCGGCGCTCTCTGCGACTGAGGTGAACTTTTGGACCTAGCTT
AACCTTTTCCGGATTGGCATCTCCAGTCTCTTCTGGAAGGTCCTCCTCCTC
ATCGCTGACAGCTTTTGAGCGCGTAGCCTCTTTACTAACAGAGATCCTTAG
AGCAGTGTATCTCTGCTGTCCAATCCTTTCGTACTTACTCAACCAAAGTAA
GTACTTACTGTCTTGTGTCATTCATACCTTTTGCATATACCCTCTGGCCATC
CCCCAGTCCCAACCCTAGCACAGCCCTAGCTGCAGTGTTCCCAGTGGTTTG
AAAGGAGAGTCATCCTGATTTACCGCTGCAGCTTGCTGGCCCTCAGAGAA
TGCCGTCTGTTTAGGAAAGCAGAATTCTGTGGTGGAAGGAAAGTCTTTTCT

CGGCAAGTCTTGCCGTCAGTGGGTCAGTGGACAGTGGAGTAAGAGAACAG
CACCACAGGTTGTGAGAAAGAAAGACAAACAAACCAAACAACAACTGAT
CAACTGGTATATCCTCCTATTTCAGGAGTAAAGCAAACTGAAAAATTAAG
ATTTTGAGAGAGGCAGGTGGATCAAATAAGATGGATAAGTAGGCTCTAGA
GTTGAGGCTAAATTCTGGATTGTGTTCCCTGTTTCCTCAGGGGTCCAAGAT
ATGCAAAGGATTTCCCATGCTTCCATGCTTCCTACATTTTAAAAAGATGTA
TTATTCTATGCCTATGGAGGTTTTGTCTGCGTATATGTCTGGGTACTGTGTG
ACCATGCAGTGCATGAGTAGGCTAAAAAAGGTTGTTGGATCTTCTGGGAC
TGGAGTTAGAACAGGTTGTCAG
>LNC_000893
CCGGAAGCCATGGTCCTGTTGGGCACGTGGTAAGCACTCAAGTCTCTGAG
TTCGAAGGCAACACCAGTGTCCCGCCAGCGTCGGAATTCATGGAAATGGA
TGACTTGAGCCCCTCGATCATGCAGCTTCATACGCAAGTCCCAGTCGGCA
ACACCACGCCGGGCATCGTAGCGGGAACCCAGGTAGTGGCGCAACCTCGA
GTCCCAAAGGCGACTCATGGGGAAGACTTCCGGTCCCTTCTCCCCACCTG
ACCAGAAGCGGAACACAGCCTCCAGGGCATCCCGCTCCCGGAACTTGAGC
GGCCGAAGGCTAAGCCAGGGTAGTAAC
>LNC_000992
GGTAAAGTCTGAAGAAAACTCCAAGTCTGAGATAGATGTGGTGTGGCAAT
CTATATGCTTGATATCACAAGGGTCTGAAATGAGAGTCTTGTGATCTACAA
CTTCCACAACAGCTTCTGGTATAACTGCTTTCTTCATGCAGGACATGTTGA
AGCCATAGACGTCATCCCAGAAGGCAATTCTATCGGCGTGTTTGCTCACGT
CACTCACGGCCACAAGGCTGATGGTGCAAATGTCAGGGTAGACCGATC
>LNC_001019
CCCAGGCTGGGGTAGCTCTCGCTCAGGCTGCTTGGCACACCTCCCTTCGTT
GAACACATGTGGCAGGTTGGCTGTGTGCACCTGTCTGAGTTGCTCATAGTC
GCTTAGCGCAGCTGTCAGGTCCCAGTTTTTACCTTCCAGCAGGTCTCTGGC
CAGACCAGGTTCTGCCCCCGTGGACCGAACAAAGTCTGACAGGACTGCGT
CCATATCAAGCGTCATAGGATCATGTAGAAGTGCTGCCCAGCACTCAGCC
GAGGGCAGGTTTGGAAGCAGGCTAGAGACCATCCATCTGATGTATGTGGC
ACCAGTTAGGAGACTCCTTCTTCCTTCTCCTCATGTACCGCAGCTTCAGCC
GCAATGGTCTCCTCTTCTGTCACTGCCACTTTTCAGACTCCTCACTCTCTGG
AGGTGAATGGCACTCAGGCATTTGGGTCAGCATGGCACCGCCTTGGATGG
AAATGGCCCATTGCTGCAACAGGGGA
>LNC_001147
GCACATTACATTGTACATGTAGATAAGAATACTTTAAAATTATAAATATTT
TGTTCATTCTCAATACAGTAGATTCCAAAACATTTTCAAATGCTTTTCACT
CACAATTCCTACAGACAATCTCGCCTCTAGTTGGAGGGAAGGGTTGATTC
CAATGAAACACAGACATTCCCGAAGCTTCCTGTCGTTGTTACAGAGATGA
GGCAGGGGGTCTACAGGCACTTAGCTGAGAGCACCGTGAGTCTTAGGAGA
GGCCCACGAGCATCAGAGCATCGGCCAAATGGGAGTCGCACAGTCCACA
GCTGTGCACTCTTACACGCTGTTTAAAAAGGACTTTCACTTCCCAGACACA
GTGCTACTGAAGCCGGTGTGGCAACCAGTAACAGACGCTAGACTGCAGAC
ACCTTCTGCATAGCCCATACAGCACTGCAGGAGGGAGCACGCAGCATTCA

GGCTCGCAGACCAGACAAGGAGGGCTGTGGCAGCTACGGTCACGCTCTGG
TGTGCTTTAAGATAGTCCAGGTTTAAGGAGGGAGGCTGTGCCAGCCTGGA
CCCAGCAATTCCTTTATCCCAGTCTCTCCCGAACCCCAGGCCCCTGCTTCC
GTCCAGACATTTCATGCCACCCAGTACCTTCTTTTCCTCTTCTCTCCAGGGC
CACTGGGTCCCTAGGAGATATCATCAAGACAGGGGCACTCTGGTCCCCTT
GGTCTGACTCCCAGCAGCACTGGCAAGGAAACACGGTGAGAATGGTCAG
ACCCAAGCCTACACGCAAGTTATCTGCTAACCAAGAAGGTTCATGACACG
GTGATACTTTGCATATAAATCTAAACTGTATGAAGGCAGCTATAAACATT
AAATAAAATTACTTTTGTTAAGTATCCCAAAATCTTAAGAAAAATTGTTAA
GACTTCCATTGGCAAATGTTTTCCAATCATAAAAGGTAGAGATTAATGAA
TTCTATCCAGTCCTTATGTAGATCCAGGAACACTGGAATCTATTCTAGACT
TGCGCATGTCTAGAATGGCCTCCTTACTGCTTGCTCCAAGGCAAGGCTGGA
CCGACACTTGGGGTTCTCTGCTCTGCTACTGAATCCCACCTTTTCTGTAAC
ACACAGATCCCCTTCCTGCTCATATGTTAGCCTCTGGGCTGTCCATGGAGT
CACTGCTGTTACAGAGCCCAGCGATGTGCTGAGGCTTACCCTCATAAGCA
CTAGTCTCGGGCTCAGAGCAGGTTATAGCTAAAAGCAGTCCTCAGAGAGA
AGCACAGAAACCAAGACCTGTGTCACCCTAGGAGGGGAAGCAGAGAGAG
ATACAGTCCTAGAGAAAGCCACAGGGTCTGCCATCATCCTGTGACACTTT
ACATCTGACAGACCCACATACTGTCAGCTTCCACTGTGCCCACGTAACCTC
CCAAACTATCCAATCACCATGGATTCCCACAGGCAGCCGGGGACAGAACA
CCTGAAGAACTAAGAAGAGGCACTGAGGCAGGGTATGTTTTGCTTTGAGG
AGAGGGAAAGAAAATGCCATGAGGCGAAAGCCCTGTGTGTGGTGGTCAG
GAAAGGAAGTGTCTCAGCTGAAGCTGGGAAGCGTCACCAGCCAGATCCCA
TCCACAAGGGCAGTGGCTGGTGACCTGTCCATCAGACACCAACACGAAGG
CTTGCCGTGTCACTTGACACGAAAAGGCAAGCAGACTGGCCAATCAGTGG
AGGGGATGTGCTGCGTGAGGAGACAGCTGACCTCCTGTCCAAGGAGGGGC
TGAGACTGGGGGCTGCAAGGGCTGCAGAAGGCGACAGTGAAAGTGGCCC
CAGCCACACCCGTGCCTATGTTCTTGCTCAGGAGAGCTGAGACCTCGCTA
GAAGGTAAATATGGCATCCTCCTCACAGGGAACCTGCTGGAGTCCTGAGG
GGAGACTAGAAGCAGCAAAGCCAGTGTCCCTGTCCCAGGCACGAAGCTCG
ACTCCTCACTGATGTGACTGACTGGGGTGGCCGCTGGGACAGCTGTCAGT
CTGTGGTCTTCTTCTCTTTCCAGCTCTTCTCTCGCTCCTCGAGCTCTGCCTG
CGTGAATGCTGCAGGTCCCCAGTTGGTGATCAGGTGAGGGTTCTTGGAAC
CTGGTTCTATCAAGCGTATTAATCCCTCATGATGGGCCACTTTGTCCTTCC
AGCTCTTGGTCGTATTTGTTGCCATCTCTAGCTTCCTTTTGACTCCCTCATA
CTGCTCCAGGGCCTGCTTCCGCTCCAACTCCAGCTGCTCCAGCTGTGCCAT
GGCCTCCTGCAGGGCCTGCTCCTTCAGGACACGCTGCTGTTCCAACTCCTG
CTTCTCCGCCTCTGTTGTCTGAATGGCCTGCTGCTGTGCCAGGTGCCACTTT
TCCAGCTCTGCCCTCTTAGAAGACTCTTCCTCCAGCAGCCTGGCCTGAAGC
TTGCGCACAGTCTCTTCATCCTGCCGGGCCTGCCTCTCGTCCTCCAGTGCC
TCCTGCAGCTTTAGGTACATGTCTTCCAGCTCCCGAACTCGCTGCAGATAC
TGCTCCAGTTCAGAGGACTTCTGGGCAACCTGCTCCTCCATTTGCTGTCTG
ATCAGCTTCTCCCGCTCCAACTCTGTGCTGAACCTGGTCTGTAGCTCTACC
TGAGTCTGCAGGCGCTTCTTTTCCTCGTCTGCGGCACGAGATGCTGCTTCC

TCCAGTTTCTTCCGCACACTCTCCAGCTCCTGCTGCTTGTTTCATTGGCCG
CTTGGAGCTCCTTCATCTGCCGCTCCAGCTCCTCCTGCTCTGCCAGCAGCT
TCCTTCGGAGCTCTTTCCGACGCTGGCGGGCTTCCTTGTGTGGTGGGGGGC
TGCCCAGCTTCAACAGATGGATGGTAGAATGGATGGCCTGAATCCATTCT
TGTTTCTTCTTCTTATCCGAGGCACTGATTTCAAAGGTCTTATCAAAGCATT
TTATTAGAAAAAGACATTTCTTTCCATCTTTGTCAGGCAGAGACTCCACAC
AGCAGTTTTCATCCAACATGATGTCTCCTTTCTTATCTTTCAGATCCTCGCT
CACATAGTAGGAAATTATGTTGGGTTTTAATACAAACCAGCGCTCAGTCC
AGTTTTTCCGTTTGTGGCCTTTCTTCATCATGTAACCCTGTTTCAATACATC
TAAAATAAGCTCATTGAAGACCTCATTAATGGCCATAGACACTGTCTGAC
GATCCATGCCCTTGCTAAACTGTCCATTTCCAATTAGTTCAATAAGTTCCC
AGGCAGACAGGCCATCTTTATTGTCATCAAAGTTTATTTGTAATGTTCAA
ATTGCTCTTGTTGCCAACCTCCTCCCATAGCTTCTGTAAGTTTCTTAAGTAG
GTATTCGATCTCTTCTGGCACAATAATTAATGGATACTTGTCCTCCGACAA
AAAGTTGAAAATGACCCACACTTTGAATGCATCATCTTCTGTAATAAGGA
GTGGACTCTTTGTGAGGTTTTTCTTGACACAGAGGGTCCAACACATTCTAT
TGAATTCAATCTTGTCAAAGTTGTCTTGGACCTTTTCCAAAATGAACTTGT
TTAAATATGGCATGTAGCCCTGATTGGAGACAGGCCCCTCATCGTCATCCC
GGAAGTGCTCCTCAAGGGCAACAGGGTCGTGTGGAACCTTCAGCACCGTG
CACAGGTTATGGGAAAGGACCTTGAGTTGCGACTTGGAGACCTTGCCGCT
GCGGTCCAGGTCGAGCGCGGTGAAGGCGTGCCAGATGGCTTTGAGCAGTT
CGTCTTTCAAGCCCCCCATGGCCGCGGCCGCGCTGCCCCAACAGACCCGA
TTCCCCGTCGGCCCTTCAGCTACTCTGCTTCCAGCAGGATCTCCACAGACG
ACGCTGCCCG
>LNC_001160
TTTTTTTTTTAATTGAACAGGCTCTCTTGTGTTAAGGAAAAATACATATTC
AGGGGTTGTACATAGATGTGGCAGGAAATATTCTGATGTGTTTAATGCAG
ATCCTTAAAGTACTGTATTCATAGCACTGTGTGCATCTTGACCACAAAATG
TATTTGGATGAAGCGGTAATAAACAGACTTGCAAAAGGATGTGGTGGTGC
TTTTAACAGATGCATGAGTACAAACCTGCCAATGAGCTCATAGCTGAGAT
AAACATCCTCTTGCTCTGAATATTTGTTGACTCCTGGGTTTAGGGTGCTGC
TGATGGCTTGGAAGTCAGTGGGAACCCCTGGCCTTCCTTTGGACTATCCAT
GAAAATCAGGGGCCTGGTCCAGAAGTCAGGCACAGGCTTGACTGAGTAAC
CCTTTCTTGTAGGTTTGTGTCTTAGTCATGGGAATAGATTGTCTCTCTCTCC
TCTAACTATAAAATATCATGACTTAAAAAAAATTAAGTAGGGAAGATAGT
ATTTTTGTTGAAGAATTGACTTGGGGGCTTCCTGGCAGACTTATCAAGTAG
TTCTCAGAGCTAAAATGCTTTATTCTAAGGAAGTTTGGGCAACCTAAAAG
GTCTGGATATGTGTAGGAAGATTTTAAGTTGGCTTTGGAACCAGTGCCCA
GCTTACTGTCTACATTGGCTCAAAGAAGGATCTGCTGACAAGGCTGATGT
GTCCGTCCAGCATCCTAGGACATTTATGGCACCAACAATCAAAACTCAAC
GGAAATCACGGCTCACAATGAAATGAAGTAGCCATCATCACCTGCTGACC
TTGTGATGCCAGTAAAGCAGTATTGGGAGGTTTGTAACAAATGCCACCTC
ACCTCAGGGCTTCTCCTTCAGGGCACCGAAGAACCCATGACCCTGGAGAC
TTACCAAGATGCCTGGAGTTGTGCTAAATAAAAAAGGAGGCGGACAAAC

AGGTTTCTGTAAGAGTGAGCAGGCCAAAGGTGAGAGAGGAGGATCTGTCC
TTTGCAATTAATGGCTGTGTCTCCCGCATGCTGCATCCTGGTTGCTTCTCCT
TGTGGATGGCCAAAGTCTCCACCCTGCTTGACCAGGGGGATGCTCCAGAG
GGACACGATTCAGTTTAGTACAAGGTGAACCAACACTACTCAACTCAGTG
CAGGAAGATGGCGCCTGGGGACAAGATGCCCCGCTTGGCCTGTACTCACC
AAGAAGTATTCAGACAAGATGTCCCTCGGCCTCCATAAGACTATTGGGCA
AACCACACCGTAAAAATGAAAACTAGTGGACACAATTTAAATCATCTTAA
ACAAGCAAATACACCACCCAACCATTGGTGACTAGAATCTGGAATGCAGC
CCTGGGGAGTTGAACCCCTACTAACAAGGGTGCACGTTGCACGCTCTGAT
CTCCTTCTTGTCTTTGCAGCTGGTAAACTGGGAGCTTTTGAACCTCTTCTTC
ACAGTCATGTAGCGTTCTTGGATCCCACCTCCGCACAGTTTGGTGCACTCT
GACCAGGCTGTCCACGGGCGCATCCGACAGCCTGAAAGTAAGAGAGCGC
CCGGTGAGGCCAGCAGAGTACATCCAGCAGTTCAGTGAGGTGTCTTGCCC
TTCACTGCTTCTCTGTGTAATGAGTGTTGAATTCATTAATGCCTGCTGTCCT
CATTAGGGACTGTTAGCTCCTGTGTGACAATTTGCAAAAGCTATTAGTGTA
CTGGATCTTTTTGCCTGTCAAAGGGCAGAGTCTGTTTCCCCACCCCTTTGA
ACCTTGGCTTGGTTTGGTGGATGGGATGTGAGCAGACACCATGCCTGAGA
GCACTGTTGCGTGTGAGCATGTCTGGGTGACCTCACTGGAAAGGCCCTGT
GGAAAGAGGCCCCAGGAAAGCGGGTGAGGCTGGGGTGCTGTGCCAGCCC
AACTCATTGTGGTTGCACAAATGAGACTGAGTTTAACCTGGCTAAACCTC
AGAAGAGTGAACCCAGGCCAGCTTGCCTCTGGGCTTTGGGATCATGCTAA
TAAAGGTTTGTTTAAAGCACTAACTTGTAAGCAGTGACCAGCCATGGGCA
CTGAACCACCTCATTAGCCTCCCTGCCTCAGTTTTTAGTCCACCCAATACT
AAGAAAAACAATTCTTTTCTTCCTCTGAAACATGGACAAGTTTTCCTCAGA
ACCTCCTCTGACCCCCATAGATGAAGTCTCCTGTTTGCACGTTTGCACTTG
TCATAGCTCTCCATATATCTATCTCTCCTTTAAACACAGATGCTCATGGCT
ATTATTAGGTACCTGTTTGCCCCAACACAGCGGAGTTTGCTGGAGAAACT
GCTGAGCACAAAGCCTTTAACTAGTACGAACTGATGCCTGTCTGCCTGCCT
GCCTGCCTGCCTGCCTGCCTGCCTGCCTGCCTGCCTGCCTGCCAGCCAGAC
ATCTTGGTTTATGAACACTCCCGAAGGAAAGGATCTGTCAACTCAGGCCA
TCCCTATGCCAAAGAATGAACTGTCTTTTTATTTTTGTTTTATTTTCCTTAT
ACAGAGTGTGTGTGTGTGTGTGTGTGTGTGTGTGTGTGTGTGTGTGTGTGT
GTGTGTGTCCTTGAATTAGCTCTGTAGACCACCAGGCTGGCCTCTGCCTCC
CGAGTGCTGGGGTTAAAGGCATGCACCATGGCCAGATGGACTGTCTTAAA
GAAGAGTCAGGTGGCTTTCACTTTTCTATGGCTGCCCTTCTTTGCCCTCAG
TGAATGCCAGGACCACTTAATGGTCTGTGCCCAGTGCGTAAGCATTGGAT
TATCTTAACTCATCATTCCGTGTAAGGATTATTATTTAAATTTCTTTGGTGT
GTGTGTGTGTGTGTGTGTGTGTGTGTGTGTGTGTGTGTGTGTGTGTGTGTG
TGGTGTTTTGCCTGTCAAGTTCAGGAGGCATCAAATCCCCTGGGATGCTGT
GAGCTACCATGTTGTGAGCGTCTGCTGCTGGGAGCTGTACCTGGTCCTTTC
CTGGGTCCTTTGAAGAGCCATCTCTCCAGCCATGGATGCTGATCTTATTTC
TTCTAAGTCATGTTTTCCTATCTATTTGTTTATTGTGAGTTGGCATGTATGT
CTGTGTGAGGGAGTCAGATACCCTGTAACTGGAGTTACAGACACGTAACT
GTTGGAGTGTTAACTGCTTATGTGGCAACCTGGGTCCATATTAATTTGAAG

AAATAAAAATCTAAGTACTAAGCTTTGAGGTTAGCATTAAACCAAGAAGC
CACAAGGCCAGGGCGGTCTTGTCTCAGCCAACTCTCGATGGAATGATTTG
ATCTGAAGAGTTAAGAAATTTCCCTAAACAACGAGGCTCCCCTCAACACA
TCTGTGCCATCATTTCCCTTTTTGCAAATGGGGTTGGTTTGTCATAGCTGAC
TGTGAGCAAGGTGTTTTGCAAACGGAAAGAAATAACTACAATTTCTAGTG
GCTCTTTGCTAGAAAACTGATTTTGACAGCCAATTAACATCTCTAACCTTT
TTTGCTACAAGTATTAACCCAATTTCTTATAAATTATGCTTTCTTAGTATTT
GGGAATCGAAACTTCAGTCTGAATAGTCCAAGTCCCTTTCTCTTCTCCCTG
AGCCCAGAGCCGTCCCACATGTCCTGGAGGCCGGCACAGTCTCCTAGGGA
AGAGTACCTGGGAACTGCTCTCCATCTGACTCTTCCCTCAGCTGCTCACTC
CTCCTGCTCTCTCGGGCCTCCCTCCAGCGCAGCTTCTGGATCGATGGGCTG
CGAAGGCATTTCCGGGCACGGCACTTCTTGCGTTGCACAGTCTCTGGGCA
GGGTGCACCTCCAAACTGAGGTTCCATTTGGATTGTCCGGGTTCGAATCAT
GTGACCTTTCCCACAGGACTTGTTACATTCAGACCACTGGGACCACTCGCT
GAGTTCGCAGTCAATGGCTGGCAAGATACAGACAGGTGTGAGTATATCTA
TGTCTGAAACAAAATAGTCCACCTTTGGCCATCTCAGCCAGGCTGCCAAG
AGCAATAAACCATTTTCCAGGAGGAACTGGTCTCTAGCAGCAGGGGTGTT
ATTCAGACCATGTAGTAAGCAGTAGTAGGGGCAAGATTGGTGCTATGTCA
TGACCCACTCTCTTCCCATTCTGACTCCCGTATGACTGGAGAAAGTGATGG
GGAACTTTGAAGGTGTTCTCATAGCACCTTGGCCTGCCTGTGTCTTTCTGG
AATAGTCATATCAGCTAGTGGCACTGACCAGCCTGCCTCCATACTCCCAGT
CTCAATGACTCTAGGTTTCCTCAGGGAAGCCCTAGTGTGGTCTTCAGAGAA
TGGCCATCTACTTTGGAGGGAACAAGATAGCAGGTGGTTGCCTACAGGGC
CCACACCTAGGACTTTTCTCAAACCTAAGCAGCTTTTCTTAACTCTGCATC
CTGCATATGGTGATCTGTGTGAAGCACACTGGAGCCTGAGATAGGAAATT
CCACAAGGGATCAAGAGAAGGGGGTGTTTTATGGTAGCAAATTCTTAAAA
GCCAGCGCCTCACAATGCAGGGGGAGATAGCATTTTGGTCTGGTTTTTCA
GAGTGTGTCCAAGAAGGCAAATAATTCTACACTGTTCCCTGTTTCTGTAAA
GCCTGGACCAGTCCTCACTCTTAAGCATTTTCCAGCCTCTTTCCCTGAGGG
AGCTTGTCTGCCAAATGGGGATAATGATAGTTTTTGAATTAGGTTACAAG
GCTTAAATGAGCTGTTTGTTTCCAGCAGTGCCCTGCAGATGTTCATTTCCT
GATCCCCTGTGATGGACTCTATGGATATAAAGATTTATTTGAATTGGTGAC
TTGTCACCATTTGGTAAAGGCTCTAGAGTCCAGTCATCGAAGTAGCTGTGA
GCTAACCATGGTTAGTAGAAGCCCCAGGCTACTGAGTTCATTGGCTGGGG
CTTGCACCTGCTCACACTTGGTGCCAGTAGCTCCTTTTAGTCCTTAGAGTA
GCTTAGAAAGTTTTGCTGCTATAAGGACCTGGGAACCAAGGAACAAGGAA
GCACTATTGGCACGTGACTACACAGATTTCAGAGGGAGATTTGGGCACTG
TGTTTGAGAACCTGGCCTTTCCTTTTTTCTTTTGTTTTTCCTCTCTTCTTTCT
TACCGGTCTGTTGGTGTCACTGATTTAATGGGCAGCCTCAGCTCAAGGGTG
AATAAAGTGATCACGGGGACAGGATGGTTGACTTGGGCTACATATTTAAT
CAGTCTCCCATTCAGACTTCAGTCACTGTGTGTGTTTTGCTGACGATATCA
TTGAGACTTTCTGCTAACACAGATCCAAACTCCACTCTCTGAAAGATTAGT
TTCACTATAGGATCTAGCAGTATATATCCTTTTGCATGTGTATGTGTAAGC
CCAGACATCTCTAAGCTTGGAGATGATTTCTAGTACTTTCCTCCCGTGTTC

TCCACTGTATTTACTGAAAAAAGGCTGAACCCAGAGATCACTGATGGTAG
CCAGCTTGCCCCAGGCTTTTATGTGGGTTCTGGAGAGCCAAACTCTGTTCC
TCACGCTTGCACGCAGAACGATTTATCCACCTAGCCATCTTCTTATCTGGC
AGTATTAGCAGAGAGCGGTTAGGGGCTCACTCAACCAAGCGACTTTGGAG
GAGGGAACGGTGAACTTCATAACCGGGACTTACGGCACTCTGGCAGCATA
CACTTCTCCGCCTGCTCCAGATCCTCATTACAGTCCCCCAGCTCTGCCAGA
GACTTGAGCATCCGCTGGCGCGTCCGCATGCCCTTCCCACAGGTCACGCTA
CAGTCGCTCCACTCGGACCAAGGAGACAGCAAGCACGGGATGGTATGGC
ACTCAGGCATCATGCATTTCTCCGCCTGCGAAGTCTCCGCCTTGCACATGG
AGCCGTCCGCGGGGCTCATCTTGACCATGCGGTGCCGCTTCTTCATGCCCA
TTCCACAGGTGGCGCTGCAGTCATCCCACTCACCCCACTCAGTCACCAGGC
AGCTGCTAGGAGCTAGGGAGTCCAGACGTTGGAGTTGTGAGGCTGGTGCT
GGGATCCACCTGCTTGCCCCACCCACCTACAGCTTGCCCCACCCACCTACG
GGGCACCCCCCACTCACAGCACTCCTCGTTGACCGTGCACTTCTCTGTCTC
TTCCGTGGGCAGCATGCACACCGAGCCGTCTTCCGGGAACTGCTTCACGT
ACCTCTCCCGGGACCTCATACCCATGCCACACGAGACACTGCAGGGTGAC
CAGGTGATCCACTCCGACATGGTACAGGTGGAGCCATCTTCATCGCTGCA
GCCGGGGCCCATGCAGGGCTGGAAGTCCTGGGTGTCAGGACAGGGGACA
CTGAGGTCCAGCTGTGCCTTCAGCATGCGTTGCCGCATCCTCTTACCCTTT
TCACAAGTGGAAGAGCTGCAGGCCGACCATGGGGACCAGTTGGAGTAGA
TGCAGGTTTCAGGGGTGTCATCTGGAAAGAGTATTTGACATGTTCTCCCTG
GGAGCCTGTGGAGGTTATGTCTGGCTCTCTGCATGGAGTCTCATCTGTGTT
CAGGGACGGAAGCAGACGTCCTTTTCCTTTGAAAACGATGCTCTAACATC
CTGCTGGTAAATATCTAATGAACAGCGACCGTTCTCTTCCTGTAAGAGCCG
TTTGCATATGCACATCTTCTCGTCACTGCCCTGCCCTGGGCAGACATGTTT
CCAGGCATGTACACACTTCATAGACAGCTTTAGAAAGCTCACAGCTTTCCT
GCAACTTAATTTTTCCCTTGGCTCCACCGACTGGTGTCCATTCACTGAGCA
CCTGCCCTGTGCTGGCATCCTTAAGAATCTGACTTAATTGAGCCTACTAAT
CCTCATTTTCTCTGCTGTGAACATTTCTTAGTCAAGGGAAGACAGGTAGGA
AAGATAGCTTGTCAGGGTTCCGGACAGTAACAGAAAGTCTAGGGTCTGTG
GGGAAACTTAAATTCAAACCAAGGCTCTGCTTCTCTCTCCTGTCTGTAGTG
TCTGGACTCCTGTCCTCCATGAGGGGTAAATGAACTGTCAAGGCAGAACA
AACATCAAAGCAGCCAGGGATCTGGATGCTATGACAACTACCAAATGTCA
TCTTCACAGCCATGACAGACAGCTAATGTTAAGTACTCATTCAAATGTCAC
TCCTTCACCAGGTGTCTGATACTCAGTGCCCAGTGTTGGCCTTGGAGTTTA
GTCTTTTTCTTTGGCTAGGGTGCTGGGGATAGAAACCAGGGCTTGGTCCAT
GCTAAACACGTGCTAATATACGGACACCCCCCCCTCCCTTCCCCTCCTCCC
AGTCCTGGTTGTTTCAAAGACCATGTCAGAGGCTACTTACTGGTCATGTCA
CTTTGTGTTGCCACCTACAACAAAGGAGAACAACATACCTTCATCTTTCTC
TTCTGGAGCCAGGTCGGCTACAATATCATCCACATTGTCAGGTACAATGTT
GCATTGTTCTCCCTGCAAGAGAAATGGAGAAGGTACTTTAAGAAAGGAGG
ATGGGGGGCTGGGGATTTAGCTCAGTGGTAGAGCGCTTACCTAGGAAGCG
CAAGGCCCTGGGTTCGGTCCCCAGCTCCGAAAAAAAGAACCAAAAAAAA
AAAAAAAAAAAAAAGAAAGGAGGATGGGCAAGGGAGAGGTAAAATGGG

TAAATCTAATCCTTGCTTGGCACAAAACCCTGCAAGGGCAGATTTCTGAG
CCAACATTTACACCTTAAATGAAAAGAAATGCAAGAAAAAACAAAAGCA
ACAACTAAACAGATATCCTGAACATGGCAGACATTAGCAATTGACTGTAG
AACTCATGATAAACTTGATCAAATCATTCATTTGATCCTGGCTTCACTCTG
AGTTGCTTTTACTAATTTATACCTAAAAGGATAATTTTTATACTTGAACCC
TCAGGAGGCCCAGAAATTTCATATCCAGAGGTGCTTCCTGCCAGAGACCC
CAATGATTCTAGAAAGGTGTATAGGCATATTATCCCTCCTTATGGTACCTT
CCGGGCAATTCTCTCGATGACGACTCTGGCCACTTGTGTGATGGACCCACC
TTCCGGGTCATAGAAAGGACTCTGAGGATGGTCCAGACTAGTCAGGGGTC
GGATTTTTTCCTGAGGAATTGTGGGCTTGTTTGGTGACTGCAGGAATAAAA
CAGACAGCATGGGAAACACAATATGAAAACTTCAGAGCAATTAGTCATTG
AACAAAAACAGGGCCTTGCATGCATGTCTGCGTGTGTATTTGTTCCAGGG
ATCAAACATGGAGCGTTGCTCTGGTTGGAATTTGTAAAATGTAACTGTATT
TACGGCATTCCACTTGTGCAGATCCTGTGCTTAGAAATCCATAAACCTAGC
TTAGAGTCTTCCACATGCACCGAAAGTTTGTGCCCAGCTGGAGGAGCCAA
GTTCCAAAGAGGTCATCCAAGCTTAGAAATGGCTGAGCCAGCTTGACCTG
AATCCAAGGCGTTTTCTGCTGGACCTGGCAGCAAACTGTTCTCGTGTTGGG
TCAGATGTGAGTCACACCGCCTCTGACGACTAAGCAGCTCAGCCACTGCT
GTGGGAAGGCGGCCCCAGACTATGACAGGACGAAGTGTGGCAGGCTTGA
AAAACTATTTACGAGTGAGGCACTCTAGCACACCTGTGGGGCATGGTGAG
TCTGGTGCTGCTCGGGGCTCCATAGTGACAACGTGTCGAAGGCAGAACAG
CCAAACAACGGCTGGAAATGTAGCTTAGTGGTGGAGTGCTCCTCCTGGGT
TCTACGCAAACCTCCAAACCCACCAACAGCATAGCAAGTAAACAGAAACA
TTTAAACATCAGGGAATCAGGTTTGCCCTAGAAAATGACAGGGTGCTGAG
TGATCTCTCTCTCTCTCTCTCTCTCTCTCTCTCTCTCTCTCTCTCTCATTT
CCTTCCTTTTGGGTGTGAATGTAAGTGCACATGGAGTCCAGGCACATGGGT
ACATGTGTGTGGAGGCCAGAGGTCATCTTCGGTGTCATTCTTCAGGGACTC
TCCATCCTGCTTTTTGAAACAGGGTCTCTCCATGGTGTGGGACTTGCAGGG
TAGGGTCATGCTGTTGGCAAGCAGGCTCCGGGCTCAGTCTGTATCCCTCTC
TGGGCACTGGGTTCACAATCTAATGCTATCATGTCTGATGTTGTACCTAGG
TAATGGGGTACTGACTCAGCTCCTCCTGCTTACCTGGCAAGAACTTCACTG
ACTTCACTCTCTCCCTACCCATCTCCTACTGGTTTTCTGATGGCTCCAGATT
TTAAAAAAGTTATTTAAAAATATCTTTTTAAAAGTACTTACTTATTTTACA
TGTTTAAGGTTTTTGGTTTTTGTGGGGTTTTTTTGCCCATATGCATGTATG
TACAGCACTTGTGTGCCCGGTACCCAGTTAGGAGAGGGCGTTAGATCCTC
CAGTTATAACCAGAGTTACAGATGTTTGTTAGTTGCCTACAGGGGCTGGG
AGCTGAATTACTCCTCTCCACCACCAAGTGCTCTTAACCACTTAGCTACTT
CTTAGGCCTTGTGATAGCTTCAGTACCAGCTATGCAGTTGGTACATAGGAC
AATGCTGGCCATGCCACTAGATGAGGGACTTCTAGGATATACCTGAGTCA
CACCCATCTTTGTGGCCAGGACACCGAGAATAGGGCAGACCAGGAAGGGT
TGCTAAGGGAGTCTGCCTTCTGCCGTGGGCACACACCTACCTCGTAGGTCA
CCCCGCTGTCCGTGCCAGCATCCCAGGGAATTAGGTCCTGCACCACTTTCT
GGACCCAGCCACACTCCTTGGTGCACAGATCCTCTGCAGATAGGCCCACG
TTCCAGTCAGGACTGGGGCCCATCATGGTTAGGAAGGACATCAAGTGGCG

TGTCCTGTCCACTGAGAATTCGGCTGAGGGTGCTGCTCTCCTAGAGACCAA
ATAAAGAGGCGGTCACATTTGGGATAGGCTGCACACACTAGGTGGGGCTG
GATGGCTTGGCCATGGCTAGAAATGGGGAGGGACCGGGAGAAGGCGGAT
TTCCAAGCATCCTTGGTTTATCCTTGTGTTTCTGAGCCTCCCTGGTGGACCA
AGATGGAGAGAACAGCATCACTCTCACTCTGTCCATTTAGAAAACATCTA
CAGCATTTCCTTGGTTGATTTACTTGCCCAGGTGTGGACCGACTCATCACT
TCCTTGTACTGTAAAGCCCTGGGAAATCAGGTTTCTGTGTGTGTTGCGTAC
ACAGCTTTATTTCCTGTGTATAGAACAGCATCTAGCACAGAGTACTCAGTA
CATACATGTGAAGGGAAGAACAAATACCTCTGGTGTGTGTGTATGTGAGC
GTGGGTGTCCAGCCACGTGGGATGTGAGATGAACTTTACAATTAGCTGGT
CACCAAGGTCCCAAGACATACCTTCATTTCATCCTTACTAAGAACTAAGGT
AAGGATTATTGATTCTGACTTCAAGACGGTCATGGAACCTGTCCAAAGTC
ACAGTGTTAAGGGGCAGAATAGAACAGAGACAATGACCCACGCAAACAT
GTGTTCGAAGACAAGGTTCTTTCCAACATCAACTCCTAGGTTCTCCTCCTG
TCCACTCAACTGAGATCTCCAGAGTGCCATTGCGGTGAGGTCTTCCCATGC
ATTTTGTCTGTTTCCCTTAAGAGACCAGCTAAGGTGAGACCCTGCCTGGCA
TCTGGGGATACCCATCTGGTTGCCCTGGTTATCAGTTGAGTTGGCAAGCAA
GGCACCTTTGGCTGAATGTTTCAGCTGCTGGAAACCATGTCTCCAGACTCG
AGGTACCAGGAATAGCTCTTCAGCCGCCACCTTCTCAGGTTGCAAGGTCTC
AGGAGAGACTGCATTGCAAGAGATGGTGGAGGTGGTTCACTGAAGGGAC
TGAGGAGGACGAGTCACATTCTCTTAGCACATTCTAGGCGACAGACTAAG
TCTATCCCATTTTTCCTCCATGGTGACCCTGTGGGAGTAAAATTATGGCCC
TGTCTTGTCTCGAGGAAATGAAAGTGCATAGAGACTAACGAGCATGGGTA
GCAGGTGCCAAGGTTTAAAGGCAGCTTGGCATGACTAGAGCCTCAGGCTG
CTAGCTGTGGGGAATCCTTGGCTACTCACCAACTGGTCTGTTCATGCCATC
TGGTAGCCGGTCAGCCGTAGCTGTTTTGAGAGACCCTGTAGAGATCTGCT
GCTTCTGCCTACCCTCACCTGACCCAGAAAATGATGACTCCAGACCTGGTG
GGACCCACAGAATTAGATCATTGGCTCTTGGCAGACATGAAGCCAACCAA
TAAACAGAAGGGAAGAAAACGTGGTGAAGGCCCTGACATCATCCCATAG
AACTGTGTGGCCTTGCGCAAACTCAGACTTCTTTGTCTGAATATTGTAATC
ACTTAAAAATTATCCATCTGTCCGTTCATCCATCCACCCCCAACCCTCACC
CCACTCCCCCACCTACCTCTCTACCTATTTTTGTGTGTGATGTGTATGCACA
CATATCTGTGGATGCATGTACCTCTGCCATGTCTTTCTCTATAATTCTCCAC
CTTATAGTTTGATAGCATCTCTCATTCAACCCGAAGCTCACCATTTTGACT
CAAGTCACTGGCTAGTGAGCTCCCAGGATCCACCCGTTCTGCATCCCCAA
AATGGGGCTTCCGGGCACACACAGCCATGCCCAGCTTGTACATGGGATCT
GGAGAATCTGAGCTCAGGTTCTCTTGCTTGCACAGCGTCTCTCCAGACTCA
GAACCCATTGAGATAGGCATCACGTGACTGTATCAGACGGTTATAGTGAG
TGATTAAGCCGCTATCCTGCACACTGGGCATAATCCAGTGCCTGACACCTC
TGCTATAATTATAATCACCAGTTTAACATCTTTAGATCTTTATTCTGAAATC
CTCAAAGCTGGAACGCTCCAAAATCCTAAGCAGCTTGGGTGCTGATGCAA
TGCCACAGGCAGAAAACTCAGAAAAATCACCAAATATTTGCATGTACAAA
ATTCTTAGAAAAGGTATAATAGCCAGTGAACGGAACCAGTCTAGATGTCC
ATCAACAGAGGAACCAGTAACTGCTATATATACACAGAGGTTTAGTCATC

TGTAAGGAAAATGAAGCCGTGACATTTGCAGGAAAATGGACCGAACCAG
AGCGCAGTATGTTAAACAAAATAAGCCAAAGTCACAAACGCTGCAGTTTT
TCTCTCATCCGTGGAATCTTTTAAACATTATATACTGATGTGTGCTCTGGT
GGTGTGTGTGTGTGTGTGTGTGTGTGTGTGTGTGTGTGTGTGTGTGTGTAG
GTCATGGAATTAGAAAAGGGATCATGAGGAAAGAGGAGGGAATTTTAAG
TGAAAGGGAAGAGGAAGGAGTTCTCCACGTGGCATGAAAACAGAAGGCC
AGGGCTGCGGGGGACCAGCCAGAGGTGGGCAAGGAACACTGGGGCAGTT
GATGATGGAGTGGGATGAATTTGAACAAACCGTGACACATTTACATGAAA
ATCCTGTAATGGAATTTTGTATGCTAATTTTAGAAATTAATGAAACATTGT
GCAAAGTTATCTTTAGGAAGACCCCGGTGGGCTGACTGAATACATGTGTG
GTGAGTGAACTGATCTTATTGTCTCTAGGTCCCGAAAATCCTGCTCGCCAT
TTGGGTTTCAAGACTACCCACCTGTGTATGATGAACAAGAATAGCCCCCA
TAGGCTCATCTATTTGAATGGTCACCAGAGAGTGGAACTGCTTGGGAAGG
ATTAGAGGTGTGGCCTTGTTGGAGGAGGTGTGTAACTGGGGACGAGCTTC
GAGGTTTCAAAAGCCTACACCAAGCCCAGTCCTTCTCTCTCTGTCTCCTTT
TGGATCAGGGCTCTTAGCTACTACTCTAGCCCCATGCTTACCTGCCACCAT
GCTCCCCTCCGTGATGATCATGGACTAGCTCCCTTAAACTGTAAGCAAGTC
TCCAGTTAAATGCTTCATCTATAAATGTACCCGTCAATGGTGTTTTGCCAC
GGAGATAGGACAGTAACTAAGATACCCATTGCAGACAGGCCTGTCTTTCT
GTAGGATGAGGCCCAATGGAGACTCCTCTTCTCCAGTTCTTTAGGAGTGAC
TCTGGACTGTGGGTCGGTGGTTGGTTTCAATGACTTCTTTGTGTATATGGT
GTGTGCATGTATGCTTCAGCCTCAGCAGTTTGATTGTTTGTTTCAGTAGCG
ACAGGGTTTCTCTGTGTAGCCTTGGCTGTCCTGGAACTAGCTCTGTAGATC
AGGCTGGCCTTAAACTCAGAGATTGGCCTGCCTCTGCCTCCCAAGCCCTGG
GATTAAAGGCGTGCACCGCCACCACCCTGCAGCCTGGGAGCTGTGGCGTT
GCCCATCCAGCCTCTTTTGGAGTGGCTGCCCTGTCCTTCGGTGCCTGTCTA
ACATCCTTTAGGAATGTAGCCCCCCCCTTTCCAATGCCACACAGACTACCT
CACCCACTGTTCCCCCAGTATGGAAGTGTGTAATTCCAGACACGGTTATTT
ATCTCCTGAAACCCACAAGTGGCAGGATTCACGGTGACAACCTAAGGAGA
CAAATCTGGCTGTCTCGAGGAATCTGGAGCATTAGGAGCCACCGGACTCA
ACCCAGCACAAACTGGTCCCCATGTGTCTGACAGACTTGTGTGATGGTGA
ATCTTCATGCCGGCTTAACTGGATTGTGAATCACCTAGGAGGCCAGGGTCT
GAGTGTTCTCAAAGGTGTTTCCAGAGATTAACGGAGGACCTCTCCGAGTG
TGGGAAGGGCCATCCATGGGCTGGCACCCCTGACTGAATGCAGAAGGGG
AGAAAGGGAAACCCAGTGAGTCCTAGTCCATATCTCTGGGTTTCCTTGCTT
CAGGTGGACTGTGATAGGCACCTCACACCTTTCCTGTCACGCTGAGCTGTA
TCTCCTGAGACCATGAGCCAAAATACACCTTCCCTTCCTTTTCGTTCTTTCT
TTTTTTAATTTTAAAGATTTATTTATTTATATACACAAGTACACTGTAGCTG
TCTTCAGACACACCAGAAGAGGGCATTGGATTCCATTACAGATGGTTGTG
AGCCACCATGTGGTTGCTGGGAATTGAACTCAGGACCTCTGGAAGAGCAG
TCAGTGCTCTCAACCTCTGAGCCATCTCTCCAGCCCCCACCTTTCTTTTCTT
AGGTTGCTTTTTACCAGGTATTTGGTTACAACAAGAAAAGTAGCTGAGAC
ACCACTTGATGACCAGATCTCGCAGAAGCATTTTCAATCAAAGCAACTGG
CAGCCTGAGGGTGGTTAAAGGTTTCAAGGGATGCTGTCGGGTAACACAGC

AATTCTGAATGCACAGGATATAGGAGCAATTTTATTTTTAAAGGATCTTTA
GATCCACCTTGGCCCTTTCTCTTATCTAGAATAAGACAGTTCCCTGGGCTC
TGAGCTGCTTAGCACCGCTGACCTAGCTTCACTCACTGACCTGACAGGTCT
CTCAGAATTTCCTGTTAATGTTGCTTATCCCAGGGAGGTGCCTATAGAGTG
GAGGCCTCTGTGTGTGTGTGTGTGTGTGTGTGTGTGTGTGTGTGTGTGTGT
GTGTGGTTATTTCAGCGACATTTCTTTGCCTGGCACAGACCTTAGCATGCA
ATGGGCTCCTTCCAAATGTTTGTTGAAATAAATGAATTAATCTAGGAGATG
AATGTGGAGCTTTTCTATTTCCTTTGACCTTCAAATTTAATTAAAGGTCGC
ATCAAAGTATTTATTATTTCTCTTATAAAAACCTTACTTAAAATAATGAAA
TGGATGAGCGCATGTGTGACTTTTTCTTAGGAAATGCTAGCCGGAACTGG
GGCTCTTTAATTTAATCGAACTTTAATAAAAGCTCTTATTTATGCTCTCTCA
GGTGACAGGTCTTCCACGGCGATATGACATTCTGCCAACTTGGTGTCCTAT
TTTTCTTTTATCTAGGATTTAGATGTGAATTTTTTTTTCTTTTTTAAGCAGG
GAAAGTTGATAGAGACTTATTGCATGCCAGAATATATGACAAATCTAATT
AAAAATTATATGATATTTCAAAGTAGAACAGAAATTAGCGAGCTGGGAGG
GGAGAGAGAGAGAGAGAGAGAGAGAGAGAGAGAGAGAGAGAGAGAGAGA
GAGAGGAGGGGAAAGGCTGGTCACCAACTGCTGTCAGACCAAATTAACT
AAAATAAAAACATCAGATTTGCACTGAACAGCATATGGCAAGCAATTGCT
CTAGAACCCAACATCTAGATTGTAGGAAAATGTCAAACATTCGTGGAGAA
AAGGTTCTATTAAAGAAATGTTACTTTTCAAGGTGAGGATAAAAAGAAAA
GTTACGTTTCTCAGATTTGAAACAGGAAAAGCAACCACAAAAGGAATAGA
TTGAATTAACATCAGTTAAAACCTCTGTAAGATAATCAGAACTGAACATG
CTTAGTGTTACAAATCTTAGAACCCCACATCTGAGCTTCCTGAGCAGTAAC
ATGAAATCCAAACAGGGAATTCCACACTTGATCCCACACGATGGGTCACA
GGCAAATTGTAGATGCATTAAAATACTGTGCAAACTTGTTCTCCTGTAAAA
GGTGTACACGAAACCTGTTGCATGCTGAAACTTGGGTCCTGCCCTCGAGA
TATCTCATTATATCTATACACAGATATTTCAAAATTTAAAAGTCTCCATGG
ACCCTCTCGTTCTGGCTTTCTGTCACTGTCACAAAATGCCAGAGAAAAATA
TCTTCCAAGAAGGAAAGCTTATTTTGGCTCATGGTGTGAGAGAGTTTAGTC
CGTGGTCACTTGATCGTATTTCCTTGGGACTGAGGTGTTGAGGTCAGAAGA
AGGTGGTGAGGGGTGAGTGGGTAGGTCCTGACAGACCTTCCAAGCATACA
CTTCCATAGGCCTAACTTCTTCCCAATCTCCCTCACTCCAGGGTTTCCTGTG
TAGTCCCAGCTCTAGCTGTCCTAGAACTCACTCTGTAGACCAGGCTGGCCT
CAAACTCAGAGCTCCAGCTGCCTCAGCCTCCTGAGTCCTGGGATTAAAAG
TGTGCACCACCACTACTCAGCCTTTTTTCCAACTCTTAAAGTTTCCATCACC
TCTCAAGATTGCCTCAAGCTGGAGGGCACCTAAGATGGGAATCACACCAC
TTCTGGTGCCAAGAAACATACTGTGTCATAAAAAATGTCCCCATCCAGCA
GAATTGAACAGTGTCAACATCTATACATCCATAAGCCAAGTCTCTGAGAA
AAAGACCCAGTCCCAGGAATGAACAATTTACACATACACAAATTAAAAAT
AAGTGGGCATGTGAAGAAATGGCTTCTCTAACCTCTGAGAGGGTGTGGTG
CACAGGAGGGAGTGCTTTAACCTCACTTTTCTTTGCAGTTACTAAACCTGC
AAGATGGGAGTGTTGTACTTATTGTGAAGAAATTGCTTCTCCTTGGTCCCT
GACAGTCCTATGTGTCATTTTCAACAAAGCTTCCCAAAACACTACAGAGG
AAAAGAGGCAAGAAATGATTTTATGATCCATCTGCACTTTGGTTCTTGTCT

TCTAAGTTACAAATCCACACCAGTGAGACTCAGTATTTAGTTCATGAAAG
GTTTACTTTTGTCTCGTGATTTTGTTTTCTCAAGTGCAGGAACTCTGTTACG
CCACTAGGGAGCGCCCAAGCCATACATTAGGATGAAGCCCGTGTTGGCCG
ATCAGGGTTTGTCAAGTTCTTGCAACATTAGTTTCTAACCAGAAGGCATTT
GGGATTCCAAGTCCATCTGGACACGCAGCTCCGCCACCTGCTCTAGTCCGC
GTTCCTTTACTACCCCAGGGGCGTGGACGGACCCAGGTGACTCCTGCTAG
AAGGCAAGAGGGGCCTTGAAACCCTAGGGCTGATTTAAAAGGAAGATGTT
TCTGGTTTCATTTCTGGAGAAGTTGACCCAGTCTTTGCACCGCTGACCTCC
CAAACATGGGCCATGGTCTAAGCAAGAGATAACCAAGTGGAACGGATGG
GAATAATTTGGGGGGAATGACAGTCCTTTCCAGGAGATGAGTATCTTTAA
AGTTCCAGTAATTCTTAGCATTCAGACCACCCTGGAGCAGTGCAAATTCA
GAATGATTTGTAAGAGCTGTTTTCCACTTGCGTTTGTAAGGAGCCAGTGAA
GGATGTGGCTTTGCTCAGAATTTGTCTTTTCCTGACTCTACCTGAACGCTG
GGCTGTCAGGGCTCTGGCTTGATTTATTGAATTAAACATGTTACCCGCTGA
TTTGTTCACTTACTCCTGCCCTTTGTGAAGGGGTGCTGGGAATGCGGATGA
AGACTGCTGAAAAAAGAGTCACTTTCAACAGGCAGTGTTTTGCAAAGTAG
GTCACCCTGGCTTTTCCACTGGGTGAAGAGGCTGGAATCCCATTAACCTCC
AACA
>LNC_001163
TCTTTTAAAGATTTATCAAAATTTGGTGAGCCGAATCTGAGTAACATGGTG
AATTCGGTTAGTTCCCAGTAGGCCGCAGTAGTAATGAGTGGACTGTTCGG
GATATAGAAAATTTTCACGAATGGAATCAAAATTTCTAGGCGAATTCAGT
GAGTTCCCAATGGACATGCTTGTATGAGTCTGCTTTTCAATATAGAGCAAC
ATTTTGTGGAACGGAATCTAAGTATCATGGTGAATTCAGTAGGTTCCCAAT
AGGATTCGCATGTAATAAGCTTGTTATTCAAGATAGAGCAACGTTTTAAC
GAACTGAAACACTGTTTCTCTGTGAATTCAGTTAGTTCCCAGTAGCCTGCT
CTTGTACGATGTGTACTTTTAAAGATTTATCAAAATTTGGTGAGCCGAATC
TGAGTAACATGGTGAATTCGGTTAGTTCCCAGTAGGCCGCAGTAGTAATG
AGTGGACTGTTCGGGATATAGAAAAATTTTCACGAATGGAATCAAAATTT
CTAGGCGAATTCAGTGAGTTCCCAATGGGACATGCTTGTATGAGTCTGCTT
TTCAATATAGAGCAACATTTTGTGGAACGGGA
>LNC_001237
AGCAGGTCAAATAAGTACTCCTCAAGAGTAAGCACAGCACAGCTGGTATT
GGAGGCCTCAGAGGCTCCTGCTTCCTCCCCTCCGAGACCAGGAGGCAGAA
AGCAATGAGCCCTGTTGAGGAGGAGTGGTGCCCAGCTCCATCTAAGTCCA
CCTGTCTGAGGCCCTAAGCTCCACGAAGCAGCAGTGTATGGAGCACAGCG
GCCCCCTGCCAGGCCCAGTAGGTCCTACGAGGAGTGTGGCAGGCTAGCTG
GCCAAAATCCACCATCAGATTCTGAAGCTTTCCTCCCGGCCATCAATGTGG
CGGAGCCGTAGGTAGACATCTGTGCCAATGCCCTGCAGGGACTGCAGCTG
CAGGGAGCCACCGAGATACTCCGCATAGGCCCTGGACGTGGGCAACCCGA
AGCCAAAGCCATGCATAGGTCCTGATTGGCCACCACTGTGCATATCCAGG
TGGCCGAAGAGGGGGCTGATTCGGGGGTCCTGGGTGCTGGCCTCAGCTGT
GGTGAAGTGGTAGTCCATGACCCGATCCAGGTCCTTATGAGCGATTCCTCC
ACCCCGGTCTGAGATCCTGATGATGAGATCCACATCGTTATTGGCGATGGT

GATGACCACATCAGGAACATTGTAGGGCGTGTCTAGGTGACTCTCCATTG
TGGCTCTCATGGCGTTCTTGAGCAGCTCAGGCAGGATATAGTCCAGCGGC
ATAGGAATGAAGGGGAAACGGGCAGCCACGTGCCCATTGATGCGGACTCT
AGGGGCATTGCCATACTTGTGCTCGCACAGGCGTCTGGCAAAATCCACCC
ACTTCTCAATAATCTTCTTGGGTGACAGACGAGTGCAGATGATGCCAACA
AAATCAGGCTTGTCTTCATGTAGCGCCAAGTGGTGAGTAGCCAGCATTCG
GATCCCAAGTCTGGACGTTAGTGTTTATCCAGGAAGTAGCGGACCAGCT
TTTCATCCTCTATGTGTTTCCGGCTCTCACGCAGACCCTCAGCTAACAGGG
TTACCACATCCTTGTGGTCATCTAGCAGCTGTCGCACCAGCTGGCAATACT
GGGCTTCGTCTGCCTGGTCCTTGATCGGAGGGAAGTCTGTCAACTTCTGGA
AGGCCCGGATGTATAGCTCGTGCACATGCAGTATGGTAGGGTTGCAACCA
ATGATGAAAGGAAGACTACGGAAGCCCTTGATGCGGTGAGCGATCCTCAC
CGGTAACTCTTGCTGCAAGTAGCGACCACTTTTCAGAAGGTGGCTGCCATC
CTGTGAGCGACCAGAATAGAGCATCATGGTGGGAGTGAGGCGGACTGAG
GGCTTCTCTGCTACCACGTCAATAGCTGACTGGTTGTAAAAGGAGGTAAC
AGTCTTGGAGCGTTCCCTGGCCAGCTCTACATGGTGTGTATCGGTGGCTGA
TGTTGAGCGAACCCGGAGTGACAGTGAGGACCCCAAGAGGGGCCAAAGT
GAAGACCCGCTCCGAGGGCCGCTGCCCAGCACTGAAGTCAGTATCATTGT
CCCGCTTCTTTGTTGAACTAGGGTGATACTATGGGACTAAGGGCTCGACA
GGCAACGGTGACCCCAAAGCAGGAGTAGAGAGAGAGTGAGTGGACAGGG
TTTTCTCCGGATTTCAGGGATGTGGCTCCCTCGAGGATGCTGACATGCTCG
GCGCCGCCACAGCAGCTTCCATCGCGAGGCCAGACGCTGGGCCGGGGTCC
GAGCGGAACCGCGGGAGCAGTCGACCCGCCCGGCTGGCCGAGCCCGCCTT
CGCCCTCCCCCGCTGTAACATCGCGCGGGCTGTGTGTGCCCAGTGCCTCCT
GGGAGTTGTAGTTTGACCACAACTCGAGTACGTTCCTGTCCTTTAGGGTAT
CCCCTACCCCTTCAGATGAGTAGTTT
>LNC_001276
TGGAAGATAGATTTATGACCTTAGTTAGGGACTTGGCCATATACCTATGG
GAAGACACTCAGCATAAGAAACTACATCTTTTCTTCTAGCAACTGTGCATA
CCGACCGTCGAGATCAAAACAGGCAGGGAATTACTGGGTAGGCCTTAGGT
GTCCATGAGATCCTGGGATGGCATGAGGCTCCAGGTAGACACAGGAGACT
GCTCAGAACCTAGTTTCAATCTGGGATATTATTAGACATCAGTGAGACTCT
GACAGAAGAAAAGGTACCGGATACATTCTCCTGACAACATGACACCTCAA
TGGTAATTTAGAGAATGTACTAGTCCTCTGAAAATACCTGGGAAAAGCTT
TGGACCCTAGAAACATCATAGAAATGGAACAGGTTCCAGTGAGAAACTGG
AGCAGGATTGAGTCCAAGAGAGAACCAAGGAAAACATTCGAGTCCAAGG
AGGTCCTAAGGAAGACTTTAAGCCTTAATGAGAATCTAAGACAGACTTTA
GATCCGAAGGAAAATCTCTGGTAAGCTATAGCCATGAGTGAGAAGGAGG
GGAATGATTTCTTCCTCAGTGAGAATGTGGAAGACTGTTAGAACCACTGA
AAAACCACAGTTTGGATTTGGTGAGGCCTTAGGTTTACGTGAATACCTTTT
GAAAGCCTTGGGTCGCAGTAAGAACGCCAAAAGCCCTTCTATCCCGGGGA
GGTATTGCAGAAGACTTTAAATGCTGTGTGATCCTGGGGACCTTCTTAGAC
TGACAGAGAATCTAGGCAAAACCTTAGGTTCAACTTTGCAATATTGTAGG
ACACACATCGAACCCTGTCAAGGCCTGAGACCTTGTGTTTCTCAGGAAGG

TCTAACATGCCAGTGTAATCTTGCCAGAAGTTCACGACACACTACATGTCT
CTAAGATCCTTTCTATTTCACAAAGAACCTAGGGAAAACAGAGGTCTCAA
TAACACTTTGAAGAAGGCCTTAACCATATGGAGATCTTTTGGAATGTTGTA
CGTCGCATTTCGAACATGAAGAAAGCCTTCATTCCCATCCCTACATTCCAT
GAGAAATTGAGGAATATATTGGCCCACTTGATACCCCGTATAGGCTCTAG
CAAGATGCTGTGCAAGGAATTAGGCCACAGATACGACTTGGAAAGCCACA
GGGGTAACCTGGGAACCCTTGTGGGCCTTGCGCCATAGGCTTCATTGCGTC
TTTGGAGGGAGGTCTTAAAGCATACGTAGTGTTCCCATTTAAATTATCCAT
CCAGTCCTGCTTTTTGTATTGTGATGCTAATTCTGCCCCCAGACAGCAGAC
CACCAAGTACCCAGGTGATCTCATGGGAACCGTCTTCCTATATTAACCTGT
CAATAAATCTAGGGCATGTGATTGGAGACTAGAAGGGAAAGGTGAGACT
GGAGGTTTGACAGTGATAGCATTAGGAGAGAAAGAAGGGCAAAAGAGCA
AGGAGAGTGGCCTGTAGAGGAGGAGCTGAGATTGACCTGATTGCATTGGA
TTGTATGTTCTATATATGGTCTCATTTGCTTTGAAATTAGAGCAACAAAAT
GGCTCCAAACCTGGGGATTAGAACCCAAGCAACTTGAGGGAAAATTCACT
CCTCCCCACTATAGGCAAGTAGGGCTGGGCTGTAAAGTTGCCTCGGGGTG
AAAAATGATACACCTAGTGGGTCTACAAGTTTCTATAGATCTACTGAGAA
CCTCCCTCCATGCCCCATTCAGACCATGACAGTAACCCATCTCTGAGCTCA
GATTTAGCCCCAGTTCCAATGTCACAACATAGTTGGGGTTCGTTGAGCCTA
AAAGGAACAAGGACGCTGGGGTAGGTCTTCCTGTGAAACATCAGAGCTTA
GCATCAAGAGGTGAAAAAATGGTAGCCCACTTTGCAAAGGCTTCTCACGG
GCACAGGAAGCTGATCAGGTGTCTCCACCAAAGCAAGCCTTCCTGTGCTC
GCTTTCCTTTCTCTCCCATGTCTCAACAGGACACAGAACAGCCAGACCTGC
TGTACAGACGGCTTGCACTGCACACCGCCCCAGATTGCTGCATATGCAGC
CAGCAGCTGTTCAAATAAACAGACATGGCTCAGAAGGCTTGGGTCCTTCT
CCAATTAGCTCGTGCCTCCATATAAAAGATTTGGAGAGTACTGCGGTGTTC
TGAATGGAAAAGAGAGAGAGTAAGAGAAAGGCACTAGAACTCTGAAACA
GG
>LNC_001398
TTTCTGATCATAAAATATTTATTAAGGTATACTCTTAAGGAATATAGTGAA
TATAAAATGTAGGATCACAATGTGAAGCAATTTGAATCTGCTCAAAAGCC
AGCACAGCCCTCCTAAGCTGCAGATGGAGACCACTAGGAGGAATTACTGC
GGGCCAGAGGAGGAGGACAGAGCGGCTTTTCAGGAACGGTTACTACTGG
AGCTTCGGACACCTCTGTCATTGCAAGACCACACCACATTACACTAACGC
AGCAACAATGCATTCATATTGTTCTCATTAGTCATTAGTAGTCAATTTGCA
TAAAACACCAGAAAACAATCACCATGGAGATCACAAGTGAGTTAGAAAT
GGACCTGTTCTTGCAAAGCACGCACTGTGATGGGATAGACATGGAAAGGA
GTGTGCACATTACAGGAGCTGCACGCGTCCCAACAAGCCCCTCTGCCTTCT
TTCCTGGAAAAGCGAAGTACAAGGAACAGGCACAACTAATACTTGGGAG
GCAGTGTGTAGCTAAAATCTCATTTAACTAATAAGTCAATGATTTTTAAAA
TCCCCGTAAATCTTTTCTGTCCTGAGGTAGTTGCAAAATAAATCATAAACT
GGGTAATCAACTACAGTGGAGGCTGCAACTCTTCATTCATGAAAATTAGT
TGTTACTGAGAAGAACTACCTTTTGCTATCTAAAAGACAGTTGAGAATGG
GCCTCTTACTACACACACAGGAGGGTGCACAGGGAAAGAGATGCTGCTGC

TTCCCGACGAGAGACACGGTCCAGCATTGCTTTAACCCTCCCCACTCATTT
TTAGACTTGTTATTGTCCAAAAATCCACCAGGAACGACACAGAAGCACGC
AGATCACCGGCCTTTGCTGTAGCCGGGGAAGAAAAGGTCAAGGACGGTTT
GGAGGGTCTCATTTAGAGTCATGATGTAATTCTGCCCAAGGTCATGCCGG
CAAGCAGGGCAGGAGAAAACCTGGGCCTTAAAGGAGCGCTGTAAGCAAT
CCTTACAGACGTTGTGAAAGCATTCTGTTGTGACAGGCTGGTACACTAGTT
CTTGGCAGCACACACACATAAAAGACTGTTCCAATTTCTTCAGAAAATTC
GGTCCTTCTACAAGAGACGCCAGCACTTCATCCCACAGCTTCTGGTTCTGA
CAGTCTTCCTTGATCAGCCGTTGCTGCTGAGGCGTGAGCTGGAAAGCCTCT
ACGGCTTCTGTGGAATCTGATGTCTTCTGCACTTTGGAATCACTTGGACAC
TCATCATCGGAAGCTGGCCTTTTGGTGCCTTCGCTCGTCTGCTTCTTTGACT
GCCCCTTGGTTTTCTTCCCTTCCTTATCTGAGGGGTAGCCTGCTGGATACTG
CAATCGCAGACACAGTCTCCTTGATCGTTCGATTCCTTCTGAGGTCCAAGG
AGCAGGTTCGACATCATCTCTTCTTAAAAGATAACGCCAAACCAAGAAGC
CATGGCTGGATGAAATCTCTGGCCAATATTTCACTACCTTATAAATGCCAT
CATATCTGTTGCCTTCTTCTGGTGCATATTTGCTGATCTTCCTCCCTTTAAA
ACTGCGTATCACTCTGACTGGCTTACCAGCTCTCCAATTCCGAGACTCTGC
TCCAATTTTATCATCCAATGGAGCATCGCAGTTTAGGGCCAATGCCCTGTT
CATGTTTGTCAGTGTCTGGTCAGCCGACGGTGCACCAATTCTTTTATTACC
AGCAAGATTTTTACCACCGCTTCCAGTATATGTGAACTCATCACCTCGGTC
AACTTCGTCTTCGAATCCTCCAGCCAGCACAAGAGAATAAGCCCCGTCAT
TGCTGCGACCATGGATTCCACCAACATGAGGCCTATGAACCCCGGCTTCA
CTCACCTGAACTCTGAATCTCCAAGTTGACCCCACAGGAATACCAGGGAT
AGGTCCATAGTGATTGGATGGGACAATAGTACATTCTTTTGTACGACCAA
CACAAGCCATGCCCCTGCCCCAGTCTCTCCGGCTCTCCGTACTGGCTGAGG
GCATCTTCGCCTTCTTCTTACTCAGCTTGAGTTTCTCCCCAGCCTTTACCAC
CTCGCTGGAGTCAGTTTTACAGGAAGGACAATACCAGTACTCCTCTTCCGG
GACCTTATCCAAAGGCGGGCTCAGGCAGTAAATGTGATAGGCCATATTGC
ACTCGTCACACAGAAGCTGCATGTTGGGATCCCGCTTCTCTCCACACTTAT
GACAAGAACACATGTGGCATGTCTTATCTGGGTCTCCACCACACAGGTCA
CATTCAGGGTCATTTTTCCTTAAAAACTTTCCATCTGCAAAGGAAAGAGGA
TGGGCTCCAGGCTTCTCAATCTTGAAGATTTCGTCTACGAACATTACTCGG
CAGTCATTTAATGTTCCTTCAGAACCCCCCAGGAAAACTTTCACACGAACT
TCCTTTTTGGTCCTGGATATTGTCTTCAATGTGGTAATTTCGGCATCATACC
AAAATCCTCTTTTGCCTGGATTTTCTACATTGTAATTAACCATTACCACATC
ACCGACATTTAGTTCGTTCCATTTTAAAATGGTCCGTGCTCGTGGTCGAAG
TTCCTTAGCATTCATTTCTAGTGTACCACTCTCTGGATATCTTATATAGTCC
AAAGCCAGGGTCAATAAGACAAGTGCGAGCTGATGTAGATGGTTGACTGG
AAGATCCCCCTCTTGCTGTTTTCTTTACTTTAGGCGTATTATTAGAAGAGG
GTTTAACCTGAACATCATTCTGCTTAGATGTGCTAGGAAGACTGGAGTCA
GGACGAACTAGCAGCTGAATTATATCGTTCAGTCCAACGTCGTAGTCAAA
TAACGTATATCCATTTTCCAACTGCTTGCCCCGGTAGAACAGGCGCTGGCA
CTCGGGCCTTACGTCGAACAGCGCCCACACCCTCTCGCGCAGCTCCTCGAT
GGTGGCTTTGCGAGAAACGTCCTCGATGGTGCGCGTCTGGGAGCCGTCGA

TGGTGCGGACCTGGATCCACATCGCGGCACCGGGATAGGGGACCGACCCC
CTTTGTCTACCCCTGCGCTCGGGCCGCCGCGCCCCGCCCGGCCCCTGCCGC
CTCCTCTGAAGGATCCGGTTCTCTGCGGCGGCTCGGCGAACGCGGGCGAA
GACTGACGCTCTAGACCGTTCGACCGCCCACTCTCTCCGAGGTGGAAGGA
AACCGGAACCCGCCCGAGTTGCTAGCGCAGCCTGCGCCTCACAAATAGGA
AGAAGCCATGTCTCGGCGGCGCGGAGTGTTTACTTATAGAGATGCAGCTC
CCACATGGAGGTCACGGC
>LNC_001476
GCCAAACACGGTGAACAGCATGAGCAGGCCAGCCAGGCACACCAGCGTC
AGCGTCACCTGCAGGGAGTAAGGGGTGGCCCGGGTGCCGCCTCCGGGCGC
CTCGGTGCCGTTCCAGCTGCTATTGCCGGCATCCGGCTGCAGGGAGCCCAT
GGGCGCAAAGCTGCCCTCGGCCAGCGGCTGCTCCTGGCGGAACATGAACG
CGGGTGCTGGTTCTCCTGGGCCTGCTGCCAGCTGCTTTCCGGCCCCGCGTC
CGTGGGTCCTTCCTCTTCCTCCGGTGCGCGGCTGGTCCCGCCGCGCCTCAC
ATAGGGAAGAGGGCCGCAGGGAGCACGGGCCCAAGCTGGGGACCGCTAG
TGGTCCATGGACTGGAGCCCGCGAACTGGCACCGGTTCGTCTTTCCGATGC
TCCGGGTGGGTGCTTTCTCTCTCCCAGCTCCACAAACACAGTCAAGTTTTA
TTTCTAAGCGGCAGGATCGAGTGGGGCCAGCTGGTGGCTGGAGATTTATT
CCCAAGAGGCAGCCGAGGGGTGTCGTTGCGAGCTCGGGGTCCTAGGTCTG
TCTTCTTATACCCCCGAAGAGATTTCCGACTCCGTTGCTACCTCCCAGGCG
CGGTGGCGCGAGCTGGGCCGGACCTCCGGCGAAGGCGCGGACTCTGCCTC
TCCAGAAGCCCCAAGGGACTTCCAAAGTTGTGCGCCCTGCTGGGGCTGGG
ACGCTGCGCACAGAGTTCGGGGGCGGCGGCGCGTGGGGTAAAGGGTGTG
GGGGCCGCATGAAGCCGCCCCCCACCTCGGTCCTCAGAGCCAAGCTAGGT
GAGCGATCGGCTCTCCACGAGCCGCGGCGGGAGCCAAGGGTGGCCGCTA
GCCGCGAGCTGGGCGGGCGCTGAGCGCCGCAGACCCGGCGGGGTCGGAG
GACCACGTGAGTTTTAGCTGGACCGAAGCACTTGCGTCTGGGGGCGCTGG
GGTGGCTGGGTGGTGTGCGCCGGCTGCTGGGCGTCTGCACGGAAGCGGGA
GCCGCTGCTG
>LNC_001610
CATTTTGTTACTTGTATTTTTTCTGTGGATCAATTCTTCTTTTTCCTGCTGCA
ACTTTTCCAGTGCTATCTCCAAAGGGTTTATTTGCTCTTTAGCATCCTTTAT
TTCTCTGTTCAAAGACTGAACTTCAGTGGATAATTCCACAGTCTGCTCCTC
CATTTGCTGACGCCGTTGCAAATTGGTGGCTATCTGCAGTTTCTCTGATTT
CAGCTCATTTGTTTTACTTTTCAGGTGCTGAATTTGTTCCTGCTGGTCCTGT
ATAAGCTTACGGTTCAATTCAATCTTACTGGAAACTGTATCCAGTTTGTGT
TGTTTTTCTTGTTTTTCCTGGTTAACCTGCTGGACAGTCCGATCCAAGTCTA
CCCCCTGCAGCTTAGCTGCCTGCTGTGCAATTTTCCTTTCAACGTCTTTTAG
CTCCATCTGGAACCTCTCCATGATCGTGACGTCTGTCAGGCACACTTTAGC
ACTTTCCTCTTCGGGCATCACTGTACCCAAGAGTGTCTCCTGCTCCTCTAT
GTCATTCTTTAGGCGCTGTATGTCTCTATTGACACTCTGCAGTCTGTTTCTT
AATTCTGGTATTTCCTTTTCCTTCAAATCAATTATGCTTTGCCTCATGGGCA
CAAGCCCCAGCATTTCATCACGGCGCCGTTCTTTTTTTTTAGTTCTGATTC
TGTTGACTTGAGTTTATCTGGAGCAAGCCTCAGCTTGGACTGCAAGTCACT

GATGACTTCCTGTAATTCAGCTTCCGTCTGAAATACTCTCTGACAGACTGG
GCAACAGGACTGGTTTTCATCTGTCAGCTGAGTGATGAACTGGGAGTAAA
CTGCTGTGGCTCCAGCCAGCATGGCTCGCTGCTTTGAGGATTTTTCAATAT
CTTCTTTAAGTCTGTCTAAGTCACTTTCAAAATCTTGGCTACCACAAACAT
CAAACAGTTTATCTTCATAACTAGACAGCTGCTCTTCCTTTTTCTTTAGCTC
ATTATTTATATGATTTTTATTTTGTTCGGCTGAAGCTAGTTCTTTGTTCAGT
TTGGCAAGTCTGTCCCTGGTCTGATTAATCTCTTTGGATTTAGAATGAAGC
CAGTCTTCAAGCTGTTTTTTGTTAGGAAAATATCCCAACAGTGAAGTTAGT
TCATCACTGTGCCTGGACTTTATTTTTCTGATCTGTTCATCTTTGTCAGTTT
TGTCTTTGGTAAGCATCTCCATCTGTGTGCGGGTTGTTGTATGATGGTTTA
ACTGCTCCATCTCCTGATCCAGTTTCCGCAGGTTCCTGTCCAGGTCCGCTTT
CTCACTTTGGAGGTTTAGTATTTCTGCTTTTAGGGTTTCTATGCTGCTATTT
TTCTCAGCCTTGCTTAGTTCACGTTCCGCTTTTGTGAGCTCCTGGTCCAGCT
CCAGAATCCTGTCCGAGGAGCCCTCCAGCTGCTGCAGCTCATTCCTCACGT
TCCTCAGCTCAGTCTGCTTCTTCGTCAGGATCTCGGTCTTCAGCTCAATCAT
CCTCCCCAGCCCGCTCTTCTTGTCCCTCATCTCATCCATCTGTCTCTGCTTC
AGCGCTTCTTTGTCTGTAAGGTCGCTCAAGAGCTGGCTG
>LNC_001630
AAGTTTTATCCACCACACCTTTGGCTGTGATGTACAATGCTAGTTACGACA
GGTCCTTTGAGACTATTCTGTCCCCGTGAACTGTGAAACTAATGTACTGGA
GAGAGAGACTCTCAGTATAGCCCTGGCTGACCTTGAACTTGCATCAGTCTT
CCTGCCACGGCTGTCTAGGTGATGGGGTTACAGGTGTTCAAGACTAAATTT
CATTAAATTAGCAATTTTTGAACATGTGCAGAAGTATAAAGTATGAGTTCC
TACCATACAGCCACAAGCATGTTTATTACTTTGTTATAATTATGTCTTTTAT
CTATCCATCTATCCAT
>LNC_001653
ATACAAAGTCTTTATATTAACTCATTCCGACAGCCTCTGTCCTTCCTTGTCC
CTTCTCATCCTGGTCCTCATCAGGGTTACTGGGGTCAGGTCCCCCTCCACA
CCACCAGAGCTGTAGCTGACATTCAGGATGGGCGTGGCGCAGGGAGAGC
AGGGGAAGGTCCAGCTCCGGCTCACCTTCATCCCCGCTATTGGTGGGCTCT
GGAAACATGCGCTGGCCAGAAGCAGTGGCCAGCAGAGGCAAGGTAGGGT
GAAGGCTGACTCCATTGGTGCAGTCGTCCTGGGGCAGAAAAGTCATCACT
GGCTCCAGCTGCTTGCAGTCACTGAACGCTCCACTGATGTCCCACACGGA
GACAACTCCACTGGTGTTGCCACTCACTAGGAACTGCCCGCTCGGGTCCA
GATCAAAGTAGATGCGCTGATTGGTGGTCACTTCCCGACTCAGAGACCAC
AGGAGGTGGCCCGGCTGCCGGAGATCCCAGCACAGAAGTTCAGCATCCTG
AGGACAAGAACAGGCGTCTGGAGTCAGCCTCTCCTCTGACTCCACCTCGG
CCTCCCCGAATCCCTCGTACTTGGCACGTCTCAACACGTAACAAATGACAT
CCCACTCCTGACTACCCATGTGCTTTGGGCCCTGTCCAGTACCTTTCGGGC
TCCTGAGAAGAAGAGGTTGCCATCAGGGTGAAAGCAAAGGTGGGTGATA
CCTCCTTGATGGCCCCCCAGCAAGGCAAGAGGTGAACCATCATCCCAGGC
ATACAGGCCCAGGGTGCGGCCATAGGAGCCACAGGCATAGAGGGGCTGG
GCTGGACTAAAGGCTAGACAGGAGATGATGCCACTCTGGCCCTGCTTTTT
GGCAAATGTGGTTCGCACCTCACAGTCTCTGCCAGGCCTGGATGTGGAAA

AGACACGCACAGTCCGGTTGAAGCCACAAAAGAGTTGGGAACCATCCGG
GGAGAAACAGAGTGAGTGGGCCGCTGTCAACTCGTCCAGGTGGTTGTAAG
CGCGAAAGGAAGCCCGGAGTTCTCCAGTGAATGCATCCCAGATGTGAATT
GGGTTCTCCCGGCTGCTGCTGGCCACATAGGAGGTGTCTGGCTGGGTGGA
GGACATCAGAGAATACCAGCAGTAATCATAGATGGTGTCACCCTCTACCA
TTCGCAGGACAGGAACCATTTCGGCATAATCCACCTGCTCGCTTTCACTGT
ACAGTTCTGGGGGCAGGTTGTAAATCCGTAGGACATTGTCAGCACTATTG
GTCAAGATACAGGAACCGTCAGGGGCCCACTTGCAACCTTTCAAGAAGTT
CTCGGAGCGGGTGCTGAACTCGGACCAGGAGCCACTGAGGTATTGGGGTA
CCTGAGAGAAGCTGTAGCTCCAAAAGATGTCCCCCTCATCCACTGCAGCG
GGTTCCTCAGACACACCTTCCATGGCTTCCCCTGACTCCAACTCGGGCCGA
TTCGTTTCTTCTACAGGAAGGCTGGCATTTTCAGGAACTTCTTGCTCCTCG
ATTCGAGGACTCAACTCACTGGAAATATTAAATTCTACTTCCAGAGGAGC
TGAGTCCCCTTGTTGAAGCTCCTGGGACACAACCGAGCTGGCCACGGCAT
CTGTGGCTACCTGGAGTTGGTCATCTCCACCGCATGGCGGAGGCACCGGT
TCAGAGTCTGTATTTTTGTCCGCGGGGGAAGCTTGGAGCACTGGGGCTGG
GACCAGGTCAGAAGGGAGGCTATCCGGAGCTAGGCGTAGCTCCTCCGACG
TCTTCATTCTGCAGACTTAGCTAGCGGAAGGCAGAGACTAGGCGAGCCTC
GTTGAGCCAGCTACCAGCTGCCGAGGCTAGACTGCATTACCGTTCCCAGG
GGTCCCG
>LNC_001755
GTCAGAGGTGTTGGGTCATGTGATCAGGCCTGGCCCCTGGGTGGCGCTGC
TGAGCACCTCAGTGGCTCTGTAAAAGGCCAACTAGCTGGAAGCCGGAAGG
GTGGCCCTGCCTGCAGACTGAAGAGGGGCAGGGCAGGTGCCCAGTCCAG
AGAGGAGGGGCTAAAGATGACAAGCCGCACCCCACCTCCCCCCAACTCAC
ATGGGACTGGATCCACCCTGAGGAGGTACGGGTCAACATCCTGTTTCCGG
CAAGCCCTCCTGGAGGCCACACAGAGGGGTGTTTGTTCGCAGTGGGCGGC
GGGAGAGATGCTAATCGCGGTGGATCGCTGGTCCCAGCTGAAGGTCAGGA
GGTGGACCTGCCCTGACACATTTCCACGCCTGAGCCCTGTTAAAGTATGA
AGAAAGTCAAAGACTGGCTGCCCTCCAGGTTCTCCTCCCTGTCCAGTGGCT
GAGATGTGCTCCTCGGGTTGGACTCAGGCTGAGCAGCTCCTATAGGCCTA
AAGCCTGTAGCCCGGGGATGCCCAGGACAGCAGAACAGACTTTCAGATCC
GCCCTCTCCTCCTCGGGACTACCTCACCATCTACACTTGGATTAGTAATGG
GATGGTCAAGCCGAGTCTGCTCAGAGTCAGCCCTGACCTGCCAGTCCTCT
AGGCTCCTTCCTAGTTGGCTAACCACAGTCACCTCCTTCCACAAAGTTAGG
CAAAACCCTGATCGTCACCATTGCTCATCGAGTATCTATGCCAGAAAAAT
GGAGGGGGAGGGGGAGGAG
>LNC_001833
AGGGAGCGGTGAGGGAAACAGGAGTACTTCTCAACACCGGGGGACTTTA
ACAGTCTTTGAAAAGGAATAAGTTTGTCAGGCATGGCAGCATCTATAACC
TATCATTTGAGGATGAGACAAGAGTTTGAGGCCAGACCAGCCTGACAGGA
AACTATGAAAAGGCTTGCTAGGGAAGCTTAGAGATGACAGCAGCTGGGA
CAAAGTCCTTAAGTAGGACATATCCAAGACAGATGTGAGGAGAGGGGAG
GAGGGGTTGTAAAGGGCCGCTTCAAAGAATTCTGTACCATATTACTATTG

AGATAAAACAAACGAGTAGGTGCTAAACGGAATGTCACCAATATGAATGT
AGAAATACAGAATTTTTAAGTTTTAGTCTCATGGCTTCCGAATGCTGAGCC
ATTTTATGGCATTAAATCCAGGGGTAGTGGTGCATGAATCTAATCCATTTG
GGAGGCAGAGGCAGGGAGATTTCTGTGACTTTGAGGCCAGTGTGGTTCTA
CATAGTGAGTTCCAGGACAGCCAAAGAGAGAG
>LNC_001903
TCCCACTAGGCTTTATTCAGTTGAGCCAAAGAGGTTTACATCAGCCACTGA
GGTACAAACGAGGACCAGGAAGTCTGTGCGGAGGGCAGCCACGCTGGCC
CAGCCACCCGGGCAAGTGCATCTTCTAGGAGTCAGGAATGGGGTGCTAGA
ACGTGCCCCAAGGTAGAGGGTGCCCACGTGCAGGGTGGCCCAGGAACAG
AGGTCCCCAAAGCCTCTGTACCTTTCACCAAAGCTCGGTGGGCAGAAGTG
CAGCTTCCAGTCAGGCAGGAGGGGTATCCCCAGCCAAACTGGGACAGTGG
CAGTTGGAACCCGTGGATGCCAAGGATACACAGCAACACTCCTCCAGTCA
CAGGTGCCGTCTGCTTATCCTGTCCACGGGGCTGCCCCACTGGGCTAGCTA
CCCTCTCTGAGCAGCTCAGTGGAAAGGACTGAGAGCCTGCTGCAGGCAGG
CAGGGACAGCACCCTCAGCAAGGCCAGTTTCCTTTGGAATTGTGTGAGCT
GACACCAAGACACAGAAGCAGAGCAGGGCAGGGAGAGATGAGGTGGGC
ACAGGGCAAGGGCGTGAATGACGTCTCCGGGTCAGAAGCTAGTCAGTCTC
CCCTCTGGGCTGTGCCTCCTGCTGCCTCAGGGCCTCCAAAAAGGCTAATTC
CTTGGCTTCCTTCTTCTGATTCCGTGCTTCGTACTCCAACCTGTTCTTGATG
ATCCTTTGCACAATCAGGTCTGTAGTGAGGTCACTGCCACTGTCAATCTGA
CAGAAAATGCCTCTTCTCTTGGGCTCCTCGTAGGGGTCAGACCCATCCCTG
TCGGGTACAATTTCTGTCTTCCCATGACACACCAGGTCCACCTTGAAATGA
TTCAGGAGCTCTGCTGTGACGGAGTACGGTGCCCCAATCACCACTTCTGA
AACATACCGGCAGGCCAGCACACTGAGAGTCCGCTCATGCAGGTTCATGA
TGGGGTAGTTCTTGCCCTTGTACCGGTTTACTTCCTGGTCAAAGTGTAGGC
CGGCGATGACGTAGGGCCTCTTGGCTAGCTTGTGCACCTCCTGTAGGAAG
TCCACGTGCCCGATGTGGAACAGGTCAAAGGCACCAGCCACATAGATGAC
TGTCTCCCCGGGCTGGGGCTCCTTCCCAGAAGCAAACTGGATGATCTTCTG
GGATGTCTGTAGGAACTGGGACACCCCTGTCCAGGGGCTCTGCCCCCCAG
GGCACTGGGAGGAGACTTCTGAGGAAAGTGTGTCCCCGGCAGGTGTCGGG
TGAGGGGGCTTGCCAAAACTGTCAGCATATTCCCGGTACTCAGAGGACAT
CTCCTGGCTGCTATGGTGGGCCTTGGTCACCAGCAGCATGCGACCCACGA
GGTCTGTGGTGGACACACCCTGGGTGCGTTTGCACTCTCTGTACCTCCCAG
CCTGCTTCACTTCCTCGTAGGTATCTCGGCCATCTACTGTCAGCGTGATGT
CATTGCCATGAACGCAGAAGTCACAGTTGTGCTTGTCCAGTGTCTCCAGTG
TGGTGACGTAGGGAGCAGCCGGTACCACTTCATCCACCCACTTGATGGCC
TGTACCATCTTGTACCGCTCCTCCTGGGTAAACACCGGGGGCCCCTTATGC
TTGGCAATCTCCTCGTCAGTATGCACGCCCACGATGAGGTAGTCCCCCATG
GCCCGCGCCTGGCGTAGCTGGTTGGAGTGGCCGTAATGCACCATGTCATA
GCAGCCATCGCACCACACCCGCACGGTGCGCTGGCCGCCCGGACCCTTCA
GCCCAGCAGCGCCGCCAGCCCCGTGCCCGTTCCGGATCATGGCACCAGAG
GCCCCCCGCAAGTCCTGGCAGCTCCCGCGACACACAGCACCGTCGCCCCG
CCCTCGACGCTTGCGCGTGCTCACGCGGGTCTCACCCGCTAGGCGCAGCC

AATTGCGTGCACCACCCGGGGCCCCGCCCCTAGCCCGGCCACACCCCTCC
GCCCAACTTGAGCGACCAATGGCCTGTGTTGCAGGTGGCCGGGCTTTTGC
ATCTGACTCTCTACTGGTGAATGGAGCAGGCTTGTACCAAGAGTGTGCCT
GCGGCTCTCCTCACGCCTTAAAGCATTCCTAGGGGCTGGGTTCTTCAAAAG
TACCGCGATGTTGGCCCTCAGGGGCAGGCACCTCAGTGGTGCATGTGACA
GTCATTGGGACACCGAGGCAATAGAGAGATGTTTAATAAGACTTGCTCAG
ACCGGAGGCTTAGATCCTCCCTGTGCCGCATACCCAATACAGAAAAAGGT
TTCTATAAAGTGAACACTTTTAAGTAGTAAGTAGTAGAAAGAGGGAGGCC
CTGAGACTAGCCTGGAGAGTGAGGTAAGGCAGTGGTGGACTCCTCTGTGG
GCCAGGCCTGGGAAAGACGGGAGCTCTTCTTGCAAAGGCCTTGCTGCCAC
CTTTGTATAGGGGGGGATAGGATGGAGGTGTCCTTGAAGATCAAGCCCGT
AGTGGGGCGATCACAGCAGTGCAAGTGAGGCTTTCCACAAGGACAGATG
GCTAAAAACGACCGTCACCAGGAAGTGTCACCCCCAACCCCAGAGTTCTT
TCCACTAGATCTGGTGGGGGCACTCTAGCCTTCACTGTGACACAGGACCCT
AAACATGGGTCAAGCCATCTGCAAGGTGCCGACTGCTCTTTGTCAACACC
GGGGCTACGCCGCTTTCTTCCTTTTTGCACGCTTAGCACAGCCCCTGCCGA
ACCAGCCTCCTAGGATGGGGGTGGCTGAGGCAAGAGGGGCACTCTGGTCC
CCGGGTGGGGGCTCTTCTCTGCTCCTGCACAGTGACTTCCTACTGTGGCTA
CCTTCTTCGCCAGCACGGAGTGGAGTTGCCAAGGAGAAGATTGGGTCCTG
CCACCGGTTGTAGACAGGGATCTCCAGGCCCAGTTCGTCCATGAGGAGCC
GCATTACATCATCACATTTCCCATGAAGCTTCAGGGCAGCCCAGTCATCCT
TCGGGGTCCACTGCAGGTTCACAATGTAGAGTTTGGGCCGACGGCTTGGG
GGCTTCGTCATGCACCAGAGACGGGGGTACTTCTTCAGTACCTTCAAGCTG
GACCCTAAACACAGGATTGTGTCTGCTTTGCTAGCGGCCTCAGTCGCTGCC
TCCCAGTTCAGGGGCTGCCCTAGCGTCCCCCTCTCCCCAAAGTGCACAATG
GTGTCCCGAAGCTGAGTCCCACACTTGTGGCACGTCCGGCCTGTCAGGTGT
CGGTGAAGGGCGGTACGCTCCGTCACGTCAAACACTCGGACGTACTCTCT
GTTAGGGATGCAGGAGGTGCAGACTTCAATATACATATTCCCATGGAGCT
CAGAGATGGCGGTCCGTGGCAGCCCACTGCGCAGGTGGAGCCCGTCGCAG
TTCTGAGACACCACGTGTTGCACCAGCTTATGCTTGTGCAGCTGGGTGATG
CTCATGTGGGTGAGGGTAGGCTCTGCTTCGCTTAGGTCGGCAGCACTCAC
AGGCCTTCCTTTCTGGAGCAGTGTCCACACTCCATTAGGACCCCGGTAATC
TGGGATAGAGGCTGCCGTGCTGATCCCAGCGCCTGTGTAGACAACCAAGT
GTCTGGCACTTCGGACAGCTCCGGCTAGTTCGCGGACCTTCCTCCGCAACT
CCTCTGGGTCATCACACACCTCCTCCTGGCGGCGCTTGAGGCCCTCACGCC
GCCGGCTCCGACCCTGAAGCTCGGTCACCAGATCCTCACTCTCAGCTAGA
AGCCGGCCCTCCTCCGCGCTGCGCTCTGCAGCCGCCTTCCTCAGGATGCGT
GACACCTGACGGAGGCGCTCCCGCTGCTGCTCCTCCCGCAACCTCCGGAC
CCGCTCAGCCGCTTTGCGCTCCGAGCGGCTCAGACCGCCACCGGCTGCCA
TCG
>LNC_001919
TGGTTTTAATGCCCCTTTAAATATTTTTTCAAAACATGAATGACTTTCCCTA
TAGGTCCATATAATTCTAGCTAGAAAAAGTTTAATGATTAGAGCATATGG
ACAAAGAGCTGTTCTTGTGTTTCTTTTTCCGGTGCTAGGACTGAACCCAG

GCCCTTGCACATGGGAGGTGAGTGCTTTACCAGTGGCACGTCAACAGCAC
TTAGAGCTGGCCTTAAAAAAAACAAACTATGCAAAGTACTAAAAAGACCT
TTCAGATTTCACAAATGAGAACTTTAAAAATACATCCTGAAAACAATAAA
TTTCCCCCAGTGGCATCCAGCAAACTAGATGACAGATCAGTATTCTACAG
ATGCAGTAAACCACGAGTGGCTGAATGTTTGTAGACATTCAAACATTTAC
AGACAGTGGTGGTCTAATTGATGGCACTGGGAATAAGTTAGTTACTCCAC
ACACAGCCGAGGTGAAGAGCCTGGTGTACACATCATCTAATGACTGCCTG
TGCAGCAGCATGAAGCTCTTCAGTCATGGTTCTTGGCCCTGTGTCCTTCTT
CAGGTGGTCATCATCTGGTCTTCTGCTGGGCTTCCAGCTGACATGCTTGTT
TGCAGGGTGCAGTGTTGATTCTGTCAGAAGCACATTCTGGGCTCCGTCTGG
AGGACCACGCAAGAAATGGGATTGAGTTATCCTTTGATTCTTCAAAGGCA
TTTAGTTCACGGATGAGAAACTGTCTGTCCAATGGAACTACAGGTTGACC
AGATTCTGCTATGAGGACAGAGGCTGTGTATTTATATTTGTTGAATTCCAA
ATACTCCCTAATTAATTCATTAATTAGAAGGTTCTCATGAGACAACAATGG
TCGGGGTTCACGATCATCATCCAGAGCATTGAAAACTTCAGCACGGATCC
TTGCTTTTAAATGTCTTAACACTCCCCTTTTTCCAAGGTGTCCTTTAAAAC
AGCTTTCAGCTCAGTAACAGTC
>LNC_001950
AACCACATGGTGAACTCACAACCATCTGTAATGGGGTCTGGTGCCCTCTTC
TGGTGTGTCTGAAGACAGCTACATTGTACTCATATATATAAAATAAATAAT
TTTTTAAAAGTTTAAAAAATACTTTTAATGTGTGCATGTGTGTGTGCACGT
GTGCACAGGCACAGGTGTCTGTGTGTGCACACATGGGCATGCGTGTGTGT
GTGTGTGTGTGTGTGTGTGTGTGTGTGTGTGTGTACGCTTTCCTTCTACTGC
GTGGGTCCAGAGGTTGAGTTCAGGTTTTCAGGCTTGACAGAAATTGCCTTA
ATCAGCAGGGCAAGCCAATGTCCTTCTCCATCCATCTCTCCCCTTATTTCTT
TTCCCCTCCCCTCTTCCCCTTCCTTTCTGAAACTCTAATCTTTCCTGCCCTTC
CACGTCCTAGGACTACAGATGTGTGTGATGCCACTCTCAGTCTCTGTGGTG
CTGGGAATGGACCCAGGGTCCTGTGCAGGCTCAGCAGGGGCTCTACCAGA
GACTCCACTACATCCCGAGACTCCTCCCCCGTCCTTTAGTTTCTTAATATCT
TGCTTGTGAAATGATATTTTGCCTGAGTGTAGCTGTGGATCTATCCACTCC
TTTGAGAATATATTGGTTGTTCCTGAGTTTGGGCAATTATGAGTATAACTG
GTATAAATATTCTTGGGCAGGTTTTGTGTATTTGTAGGTTTTCAACTAATTT
GGGTAAATGTGATGGAACATTATGTATGGATTGTATAATAAGTATATACTT
ATTATACTTCGGATACTGCCAAACTTTTTAAGAATGGCTAATCATTTTGTA
TTCCAACAACAAAAAATAAAATTTACTCTCGCTTCCCATCCTTGCTAAGTT
TGTGTTGCCAGCATTTGTGTGTCCCTGCTTTTCTTTTTTTTTTTTTTTTTTT
GGTTCTTTT
>LNC_002042
CTCTCCCACACGCACCAGCACATGCACTCGCACAATCATGTCCTCCGTTTC
TGTTCCTCCTGAACAGCCACCTCAAACCCCACAGGTTTTCATTGTGACCAT
CCTTGAAACCTGAAAATTGGGAGATCCCATGCGAGACACTGGCACTCTTC
CCCCAACCCTGGGCAAGCATTCTCCTCATCCTCCTGGTGGGACAGGAGCTC
AGCTCTTCCAAGGCACCCAGATCTGGTGTGGTTTCCCTTCACACAACCCGG
GAACACCAATACCCAGAGCTGCTCTTTGAGGCTGGGACCCCTCGCTTCAG

GTCAACTCCTCTCACACAACAGAGGAGGCTTTGTAACCATGCTTAAGCGC
TCTCCAAAGGTTCCTGGCATAGGTACCGTCTGGTATGAGGAAGAGCGACA
GAGAGCAATTGAGCACCAAGTTCCCTAATGCCACCCTGAAGGAGGGTGCC
AAGCTCCAGTTCAGTCTGTACCAAGAAAAAGCAAGCCTAGCGCCACACAT
GGGGAAGGTGGGGATGGCAAGGTCTCAGCCTTGAGAATCTCACATCTCTA
CCCTCCAGCATAGATCCCATGAGGGACCCACTAGCACCTTGGCAATTGTA
AGGGCTCAGCCCAACTGGAGACACACCACACAAACAGTGGCCATTTGGAG
TTGGCCCAAATGCCTGTGTCGGTAACAGGGTTTGACTCCCGCATCTGACAC
TGACTGAAGGACACACAGCACAGCAGCTAAGGTCACGAGAGGTGCACTG
ACAGAAGGTGTTGTCTTCCAGAGGCACATGGACATTTCACACACTGCTCA
CAGGCAAGCTGGGACAGGAGAAGAGCACAGGCTGCCAGGGACTCAGCAG
CGTATCTAGGGCATGCCCTCTGGGAAGACAGGGCCAGGAATCAACGGGTA
AGGGCCTGTCCAATCACTGGGCTAGGATGTTCATATTCTCACATGACCTCC
TGTGTCTTCTAGACCAAGTTCCCTGAGGGGAAAAGCCCGACAGATAGAAG
TCAGGTCAGAAGAGGAAATCAAGAGAGCATCAGAGAGACTTGAGTGCCA
GCAGGGGAAGAGGAGACAGGGCCAGAGAGCAAGCCTTCTAGGTGGGTAA
GGCAAAGAGAGAGCTGTCTCCTGAGAGAAGGAACAGTGAGGAAGCTAGG
GACACAGGACTGAGACAGCTGATCTTCCCGAGGAAGGCTGCAGACCAGA
ATCTGGAGCAGACCGGACTTGAGAGAAGGCCTGGGGGGCCCTGATTGCCC
ATGGGAGAAACCAAGTTAGTTCTGGCCAGAGCTACAGACTGACACTGCGC
TGGTGCCGGCCTCCTCGGACTCCGCCGTCACTTCCCCGGCGTCCCCGGCTC
TGGGCTCCCAGTTCTTGGCCATCTAGACTGGCATGGTCCGAGAGGCCACG
CAAGTGCTCCACTGAGTCACACTTCTGCAGGTCAGAGGCTACAGTACGAA
GACTTAATAGGCTCAGCGTGTCCGTATCTGGGCCTGGGCCAGGCCCAAGG
CTGCTGCCTTCCTCCAGGCCTCCACCCTCTGCCCCTTCAATCCCACTGTCCC
CATCCTCAATGCCCTCAGGTCCAGCTTCAGCCCCAACAGGGGAGGATCGC
CGAGGGCCCAGGGGGTGAGGGGGCAAGCCCCGGCGCCTGGCATGAATCT
GCTCGCTGATCTGCTCCAGATTACGCAGAGCCACTGAGTATCGGGTCTTGG
CCTGTGCCACCTGCTGCTCCAGTTCTGTCACCTTGGCCTTGTGCTCCTCCAG
GATTTGGCTGAACTGGGCCTTGAGCTCAAAATAGGGGCGGCTCTTGCCAA
TGGCCCGCCGGAGGGTCTTCTGCAGGGCCTGGACCCTGGCTTCAGCCTGCT
GGCACAGCCTCGTCACACGCTGATGCTCCCGTTCACCTCGAAGACGCTCCT
CCTCTGCCTCGTTCACCTTACAGGTAGCATGGTTGAGCATCTCCTGCCAGG
TAGGGTCCAGTCGATTTTTGTCAGCCATGACACCCTGCTCAGCCACAAAG
ACCATCTCCCGGGCGGCATTGTGCATGCTCACAGCCCGCTCATACCGCAGT
GCTGCCTTCTGGGTCTCCTGCTGGGCCTCCTTAGCCAGTCGCCGAGCCTCA
TAGTAGGGCCGGGCCTTCTCAATACAGCTCCCCAAGTGAGAGCCCTGTGT
GTTAAGCTTCCTTGCTGACTCCTGCAGGATCCTCCGATAGGTAGTCCTGGC
CTCATCTAGCTGTAGCTCCACCTGGTTGATTTCCTCACTAGCCTGGTTCAG
ATGCTCCAGCTCCTCCTGTATCCTAGGATCCAGCTCTTCCTCCTCTCTAGG
GGACAATTTGGTCTCACTGCTGTTGCTTCCACCTCCTCCAGGCTCCTCTTCG
ACAGGGCTCCTAGGGACTTCGTCCTCTACCACTTCTGGCCGAAGTTCCCCC
TGTGGTGTCTCCCGGCCCCCTGGAGACTTTTTGAGGTCAGCCATGTTGGCT
GGAAGAGGGCAGAGCCCTATGCACAAGAGAGGGCTGACATGCTGGGACC

AGGGCCCCAGCTTGAGGCTTCCTGGGCTTTGGATGTAATAGAGAAGGGTC
TTCTAGTCTCAAAGGTTGAAAAGAAGGAGCACAGAGGAGCCTGGCATTCA
GTTAGAGGCTGAGATTCAAACTGCTGATGAGGTCCCTATAACTAAAGCCT
CGTCTGTGCAAAAGTTCTTCACACCCCACCACGGGGAGTCCACCAAGAGA
CCCCAACAGCAACAGCTCCAACTCAGGGAGCAAAGGGGGCCTGGATCCTG
GGCCGACACCTGTGAAGAGGAGCACCATTCGGGAGGGTGGAGGCAAGCG
CCTCCAGGCTGTGGTGGCAGATGGAGAGAAGGCTAAGTCCAAGCAGAGG
GCAGGAGATGCGCCTCTGGGGAGCTTCTATCCCCTAATCTTGCCTCTCCAG
ACCTGGGGGCCATCTGAGCCTCCAGGCTCCAACCGCATACTCTTCCGCGG
ACCCAACGTTTGAG
>LNC_002043
AGGGAAAGACGGGCAACAGAAGTGAACAGGCCCTTGCTGGGCCTTGTTG
GTGGATTCCAGTATCTCTACATAGTTGCTGAGCCAAAAGCCAGAACACGA
CAAAGACGCCTCCGGTGAGGGAGCAGTGTCTTACAGGTGAGAGCCACCAC
CGCGGATCAGAAGGCAGCTGTCTGACTACACATAGATGTCCCGACAGGCT
GGCGTAGGGCTGAGAACAC
>LNC_002112
CTCAGGAGTCAGTTTTAATAGTACACATTCACACATACACAGGGGTTTTGC
TTGTCTCTTAAGATATATAAATCACTGTGTGTGCATACACAATGTGGGAGA
TGGGAGTGCACATGCCAGTGTACCTTTAGCAATCAGAGGACAATCTGTGG
AGTCAAGCCTCCTTTCCATCTTTAAGTAGGTTCTAGGAATCCTACACAGGT
CAGCAGGCTTGTACTACAAGTACTTTTACCTGCTGACCCATCTCATCAGCC
CTTGTCTGTCTTACAAAAACTAGCCTGGAACAGGACAGGTGTATCCCCTTT
TCCTACACTTCCATATCTGTTCCAGCTGTGTGAATTCCTTAATATGCTCTAC
CTTATGGGTTTTCTACTGTTTTCTTTGCCTAGGATAATCCCATAAGCACGA
GGCTAACGCCTTCTATGTGTGACTTGAATCTTACATTATCAGACATGCCCT
TTCTCATACTGCAATATACACCATCCCATATCCCTGACCCCCACACCTCTG
CTGTATCCTGCTGTCCTTTGTTTGCTTTTCCTTCTTCCTGCTCCTCTTTTTGC
TTCTCTCCCATGTTAAATCCCGAGGGACCCACATGCCAAGCATACTCTACC
ACTCAGCTATGCCTCAGCCTAGCTTTTCTGCTCTAATGTACTTCATGGTTAT
ACTCAATAAATACCCAGACCAGGTTTACACAGAAGCATCCAGTTCCATGA
GGATTCTATTCCCTCCTGAAATCATACTATAACATCCCTAACAGTAAAATG
AGTACAGTTACAAAAGAACTGAAGTCATTACCAAATGTGAGGGTGGTCAA
CTCAAGTTTCAGAGGGAGAAAAGCAAATGAACGCAAACTCTGCTTTAGAA
GTCTGACCTGGAGCCTGCAGCAATACCGATCTCCTGACTGGCTTTGTGTAA
CCTAGGAAAGGAGGGGAACGTGGCCCCCAAAAGACCTTAAACATAAGAG
TAAACAAAGAAAAGCGATCTATTTACATGCTCTCTCTTCACTACTGAGAA
ATTATTGTGTTTCCAGCCTTTTGCTCTCTTTTTCTACATTATCTCTAATATCC
TGGAGTTTTCTGCAGTTCTGGACCAGAGACACCAAACTCTCCCTAAAGTTA
CTAGTTCCTTGATACCTGCCGATGTTTTCAAGCTCATCAAAGAAAGTCAAC
GTCTCTCTGAGTTTGGCTTTAACAGTTTTTTGCTCCTCTAATTCTGCAAGAA
GGGCCTGCTCAGTGCATAATTCAACCTTATATTTCTCCTGTAACTCTTTGAT
TTCCTGTTTGAGAAGCTGGAATTTTTCTTCACTGAAAGGATTCGTCTCCTG
ACACTTATCTTCAGGAAGCAGGATGTTTGGAGGAATTTGCAAAATCGATT

GTAAAATCAGCTGCTCCATTTACCAAAAAGGTTATCAAAACGTCCTTTCA
TGAAGCAAAGAAACTTCTCTGTGCATTTACGAGTCTGGATAGAGCTAATC
TCACAGTCTGGAATGCCCTCCAGCTTCTTTAAGATAACCTGTTCAACAGCC
TGCATCACTTCAAAGAGGTGGTCTTGAAATGCTATGTAGATCCTCAGCAA
GCAAGTCTGTGGGGTGAAGCCAAAGAACTGGGCCTCATAGGCCATCGGAT
CCACAGACATCCTGCTTTACCTTGAGCCTTCAGCTGTGAAAACCTCTTAAG
ACAATTTCTTGCTGTGCAGCCTATGGGTTGGCCTTAAACTCTTTCGTTTGTG
ACAGGGTCTCATGTAGCTGAAGCTGGCCTCAAACTTGGGATGTAGCCGAG
AATGGTCTTGAACTTTTGATCCTTCTGCCTCTACCACCAGAATGCTGGGAT
CCGAAGCGATCCCGGGGGTGATGTAGTCCGCCACACACCGGCTTCCCGCC
ACTTCCTTCAAACTACACTGAGCTGCTTGGAATAAGGCGGCGACACCGGC
TCCTCAGAATCAACTGCGACCTG
>LNC_002211
GTGAAGATAACAAAAAGTCTAAACAACATTTAGCCTTCTTTTAAAAACTT
CAGGCTATAACCACGGTCACAAACAATTATAGAATTCAAGGCGGCAAGGT
CAGAGGGTATTTGCAGACACATTATTTACTTGAGATGCTACTTGAAGAGTC
AATAATCAGTCTCGGATCGATCAACTCTTTCCAGAACACAGTGATCCGCTT
CTCCTGCGTCACAGCGTATTCCACCACCTCGGTTCCGTTTTCATTGTGATA
GACCAAATCAAACTTCCCAGGTTCTTTCAAAGCCGGTAGGGAGGAGAGGA
CTTCAAT
>LNC_002248
TTCCTCAGTAAATAAGCTTGACTTAGTATAAAGTACAATCTGGACATCAA
GTATTTCTGAAAACAAGCTTTATTTAAATAAGGATTTAAATACATTACATA
ACATTAAAACTGGAAGGGAAAAGAAAACCAAAAGACCAGTTTGTTCCTTC
ACATGGCACTGGGCAGTGGCTTGTATTGTGTTGAAGCCTTTATAGCTAGCC
ATAAGACCAACAGCACCAGCTTGGTATTTGTTCCCTTGTCGTAAGTTTAAC
TGATACCAGGCTGGCCTTACCCTTCATGTTTCAACATCCCTTGGCTAGGAG
AGATCTGCCTAAAAAAGACTGCCCTGGTGGTGAGCTAATGTCCATGACTT
CTCTGGAAAGGTAGCCCTTTCTGGATTCTGCCTACCTGGTCAGACACCAGG
GGTTCTTTTTACAGCCAGAGAGACTCAACTCTAATGATATAGCTGGGGCA
GTTACCCATACTCTCAGTCACCTGGGCTGTTCAAATGGTGACACTCTTCTA
GGGCTGGGGACTGTGTCAAGGGAGTCCCAAGGAACTTCTGGTCAGACATA
GCCTCCAGTGATTTGGGGGTTCTTGGCTTGGCTGAAATCCTGTTATTTATT
GCTTTGTTCCAGGGTGGACTGTCAGGGCTTACTGCTTAACCTGTTTAAAAT
GAGGGACTTCAAGACTACACAGCATGGCTCTTTTCAGTTTATTGCATGAAG
GAGTTACACTAGTCCAAGTTAAAAGCGGACCCCAAATGATTACATTATAC
AAGCTGTGAGGTTTTTAAACTTGTGACAAGGGACAGAAGGGAAATTCTAC
TCATTGCAAGGAAATCCTCACTTAAGCTTCAGAGAGCCACAAGCACTTAA
AACCCATGAACCTTCAGCTGATCGTCCTTAGCCAGTCCAATCTCTATCAGG
AACTGGCATATGTTCTTGCGCTGGTCACCCTGTAGCTGAATTACTTCTCCA
TATTCTGGATGCTCAATTACAGTACCATTGCAGGCAAATTTCTTCTTAAAC
GCCTTCACTAGTTTCTTTTTATCGTAATCATCAGCGATCCCTTGGACAGTTG
TAAGGGTCTTCCTGCCGTTTCTCTGTTGAATTCTTATATGGATATAATCCTC
AGTGCCAGCAGGAAGCAGGTCATCACCCTTACTTGCATCAGCAAAGGGGT

CGAAAGAGTGGAGGTTCTGGATAGCGGACATACGATACGATTCCTTTTCC
TCGGTGGAAACGGCCTGCGGAAGGCGGCTGCGGGAGAAGGCGGGCGGGG
GGGACGGAGCGTCGGGAAGCGAGGGGGCTCGAGGGGGAGGCAGCTGAGT
CCTCGGCGGCGGCTCAG
>LNC_002418
CTTGGCAAAGATTGCAGGTATTCAAAGCTTCAAACAGTGATAAATTGATT
TAATAAATAAAAAAAAAACCTTTAAGGGTAACACAGCTGTAAAACAACTT
TGGTCTTTAAATCACTGCAAAAAAAAGGATACAGACAAAAAGCATACCTT
TAATTGCTATGCAAAAAGGCCCCCAGGAGAGTTCCTTGCGCATAGCAACA
CTGGAGTTCTCTTACAATTTAGAATGGCTAATGGATATTTATATGATGTGG
GAACTAATTCTCAACAATGGCTTTCTGTAAATAATGCTCTGTTTAATAAAA
ATAAAAAGCGTTTCTCTCTTCCATCCACACAAGATCCCGACAGTGAAAAA
CACTGAAGAGCCCACCAATCCCACCGATGCTGTCTTACAGAGATGCTTGG
TAAAAAGGCCTTTTCTTTCCCCTCAAACACTAGATTTATGAAAAATTCAGC
ACTAAACATTCGTAATGAAGGGCAGCCAAAGTTCAAGGCAGGGCGATTTC
CTGGCTACTGCTCATCTCTGTACAGTTACCTTGGATTATCTCTGCTAGCCA
CTGGAGATGGCGAAACAAGACCGCCAGTGGGTTGTGGCAGTTAAGAGAG
TTTTGAAAACTCTCGTCTCTAAATAAACTGTCAATTAATTACAGTAAAAAG
GCAAAGGGTTGCCAGACATTTCCCCCCCCCCTCCAGTTCTCAACAACAGG
CAAATGCAGGCGCTTAGATTCACATGTATCCTCTGGTGGCCATATTTGAAT
ACCCATTGTAAGACAAGATAAACCTAGGCGATGATATACAGCTGAACTCT
TACTAACGCGACGATTACAGCAGAGTGAATGCACCGCTTTGTAAAGCCAA
GTACAATTTGGTCACTCTATTGACTACAACATCCCGAAAGAAAGTCTATTA
TGATACAGCCCCGTGACTTTTAATCAGATATTCTCAACGATTTAAACAGAC
CAGACTTGCAGTTTTCAGGTGAACTGTCACCTTTTGTAATTTGCAAAGCAT
GTGAAATCTGACAGAAGAGAAAGAAAAAAAAAAAGAAAAAAAGAAACT
CTAGCTCACCGATTAAAAAGAGAAATTTAAGTGCAGGCAACTTTTGCCAT
CAAACTTCGGCACTGAGTTATCTAAATACTCAGAACACGGAGGGCAGAAA
GGGAGGCAGCGTCTTTGCTGTGGAAGGAATCTTAAAGTCCAGTGAGAAGA
GTAAGACCAGAAAAACCCCCCCAACAAACAAAGAGAAAACCCCACCAAG
CTAAGCAGAGATCCATGAAGCACCAGGAAGGGTAAGTGAGAGATGAGGC
CCCCAGACAGATGGGGATGACCATAAAAGACAGCCATAAGGGAGGAGGG
GTGGGCACAACACTCTGAAAAGGAAGGAAAACGGGATCCAGCCATGAAG
GCCTGGGAAAACTGTTGCAGAAAGTGATAAGTGGCACCGCTCTCTACTAC
TGTATACAGTGGAGAGCAGACAATAAAGAGAAACCGAAGACAGGATATG
ACAGCAGAAATCAGAATCGGGGAGAGAAAAAGCACATCGTGGGACAAAA
CAGGTGCGTGGTGCCCACCGTACTGTTAGAGATTTAAATCGGATGGCACG
AGAACCAGGAGCCACTGCGGGTTTGATTTAAAGAGGGTGAACTGGCCCAA
TAATGCCCAGGCAGAAGGACTCTTGCCACTGCCACCCCTTGTACAGAGAA
GCTGAGTATTAGGAGCAAGAGTTGAGTTCGTCTTGTGTTTCCCTGGCTACC
GCTGATTGGGGGCTGTCTAGGGCACTCGGTGTCTCAAGTGGCAGGCAGCA
AAGAGAACATGACAGCTGGTTAAGACCCAAGACTGAACCCCGAGCAGAG
GGGCGTTGACCACGTATTAAAAGGTACAGCTTTAGTTCGAAAAAAAAAAA
AAGAAAAGAAAAAATTATTGACTATAAAGATTTTCACACCTGACTTTCCT

AATAGAGGACGAGAAAAACTGAAACTGTTCCAAGTCCACCTGCTTTTTTT
GCTCTCTCCATCAAAACTGAGTTTAAGCAACCAACAATGAGCAGAGTGTA
AATATTTCCAGGAACTAAAAGCATCAGGAGCTGTCAGGTTTGTATAAAGG
CGTTCAGCTCCTCCACAATACGCTAGTATTTGCGACAGCTACGAAATCGG
GGAGGAAACCTACTTTTCTTTGAGAAGCACTATCCTGGTACCGTGGTCAGC
TTTCTGAGAGGCCTCAGCAGGTCTTAAGTTTAGGTTTGCCACAGACAAACC
ATTTGGTCCCCACATGAAAGATGACAAAAAATTTGGCATACGAGAGGCAC
ATGGAAACATTCAGTGGACTAAGGAGAAATAACTGTTAGTAATAATGCAA
ATACAGATACGAATGCCTTTATTACACAGACAGGAAAAATCCCTGGTGGT
TTTTCAGAGGGCACTGGTGAGCAGTCATAAAAGCTTGGCACATTACCTAG
TTTTAAAGAGCTCGAATATTTGCATCAAATATAATGCCACCCAATAAGGA
CCTTTGTTTTGTTTTTTTTTTTAATGTATTCACTAAAAGCACAAAGTTAGCA
TCCGCTCTTGTATAGATGCCTCACTTTTGTCCGTACAGAAGATGCCTGTGC
CTACTTATACATGTGCACATACAAACACGCCCACAAACGAGGTAAAAAAC
AAGTGCTAATAATCTAAAAGTACTCCATGCGGTCACAACTGTCAGGACGT
TGGTTTTCTTCTAACTGGGTAGGGAGTGTGGTAGTTTTGTTTTACTTTTCAA
ACTTAGGGGAAGAGGAGGAGGAGGAGGAGGAGGAGGCGGCAGGAGGCA
GGAGGCAGGAGGCAGGAGGCAGGAGGCAGGATACAAGGAGGAAGGTGA
TGTGTGTTTAGCATGCAACAAAGAGACAACAGGCATAAAACCCCCTCCCC
GGGCCTTGCTAGGGCTGACGGAGACATATGTCGCCATGCAGGAGACGCAT
AGTTCAGATTTCAGTCTTGCTTTGTCATGCCTCCGATCAGAAAACATGGGA
AGAAACACTAGATTTTTTTTTTTTTTTGCCTTTAAACCAGAGGTCTGGAAT
ACATATGTTACAACCATAGCTATTTTATTTTTTTTTTGAAGAGTTTGAGCAC
AGTGCTTTGAGAATAAGTCTGGCTCCTTTCTGCAACTGACAAAATTGTGTC
ACAGGATCATGTTTAAAGGGGAGGAAAGGTACTGTATCTTGAAACACAAA
TCAAAGTAGCAGGACGGGACAGGGTGCAAAGGGTTGTGTGCAAAAAGGA
AAACCACATCTGTAAAAAAAAAAAGTATTCAAAACCTGGGTGCGCTGTATG
TCAAAGGGGGTGTGTGTGAACGATGGCAACGCCCAAGAATGAAGACAAC
AGGGCCATCAACATCATAGCACACAGAGGGGCCACACGGGGTTTGCCCAA
CCACATTCATTTTTGTACTTCAGTCTCTGTCACTAAGACCCAGAAGTGGCA
GTCTTGTTATTCAGCTAGCAGCATGAGTGGCAAAGCTGCTGGGAACCTGA
GTTTACTAAGGCTTGCTGGATACCCCTGTTTCTCGTAGGTATCTCTAGGTA
ACTAACCCGAAGGTTAGTGCTTGGCGAGTCTCTCTGGGAATCGATTTAAA
CTCAGTGTTCAGAGACTCAAGGATCTAACAAGCATCATTCTTGGAAGTTG
CCCCTGAAACTTTTGCCCCTAGTTGAGTAGAAACAATAGAAAAATGGATT
TCGATTGGTTACACAGCACATCATATATTTTTTATTACGTAAATTACAGCT
GCTTTAATATAAACTTAACTGTAATATTCATTTATGGTTCAATGACTTTTTT
CAGAGATGAAAGTCAGTCACTGTTGTGTGTTCCTTCCAGCTGTGAGTTGTG
AAGGCCATGGTGCTCAGACAACTGAATTCTTCCACTTCTGACTTTCAAGTT
ACAGTCCATCACTTCCCTTCAGTTATTCCTAGGAGGTTGTATAGTCCGCTG
GTTGGAAGCGACAAACTTCATATAAACTAGTCCAAGTAATTCTGCTAGAG
CTATGATTCCCAAACCTGAAATCAAAAGGGGGCCGTGCACTGGTTCACAA
GCCATGATGCAGAGACACATCCCGACCACAGCAAGGACATAGCCCAGCA
CTTGAGTGATCAATATGGCAATGGTGAAAGAGGTCATTCCAAAAGCTGTC

ATAAACACATTGTACTGAAGCAGTATTAATATTCCTGTGGAGATTACAAT
GAGGCCAAGACCAGAAGCGTTCTTCACGGGCTTCTCTCCCGGGATGAAAA
GGATGGCGCCAACAACCACGATGAGTGTGAGCGCCAGCCCGGCAACCAG
CAGCAGAATGATTCTCTTATTCGTATGGCTGGACTTGTATTTGAGTGTTAA
TATACCAAACTTTCCCCAGAACAGGAGTATAGCCAAAATTGGGAAAATAA
CAATGAGGATCTTTTCATTTGTAGAAAACCACGAAACTGGGCGGTTTTTCA
GCTCTATAACTGTTTTGCCTTCTCTGGAAAGCTCAGTCACTTCACAGGTGT
AGTTTCCCACCACAGCCTCATGGGTATCCATTGTCAAAGAGGCAATGCCTT
TGAGCAAGTCTGAGACCGAGATTTTTGCACTGGTAAAGTTTGTTCTCTAG
TAGTGCTATTTTTATTTCCATCATAGATGAAAATATACGATTTGTTCAACTT
CCACTTCACAAACATTTCATCGGTGCTTTGGGCTTCCACATTAAGGACTTT
GCAAGGGATGACCACAGTGTCATTGCATGACGTGAACTCTACAGATTTGA
CTTTACTAAGCAGGAGTTGAGCTGAACCGCAGCAGCAGGAGCCCAGCAAC
AGCGCCGCCGCCAAGGGCCACATCTCCGCGCCGCCGGGGGGTCGCCGCCG
CAGGTGTCTGGAGCAGCCGCCGCCTCCGCTGCCTTCAAGAGAACGACCAG
CAGCGCCGCAGCCGTGATAGGCAGGAACGGCCGCCCAGGCTGCCCGGGG
TACGCGCGTGCGCGTGCTCGCCGCCCCGCGCCCGCGCCCGCTCCCACGCC
CCGCTAGCCGCGCACGCGCACTCCGCCCCGCCTGGCCGCCTCTGCTCGTTG
ACGTGAAGCCAGCCTCACACCCAGCTCTGGGCAGGCTCTGGCGGGGAGCG
CCAAGCACTAGCCTGTTCCCGGGCTGTGCGAGCTGCACTCTCTGCTCACGG
CCGGTCTCTCCAGCTCTCTTCCGGCTTCGTGTCCTGGGCCGGCCTGAAAGA
CGGAGCGTGGAGCACACACGCTCACTGGAACTCACAGCACGCGCCCCATG
CGCAGCCA
>LNC_002528
AAACAGTTGACTCAGGGAGCATCAGATTTGTTTGTTTGTTATCCAGTTGGT
TGTTATAATTTGCTATACAATCAGCTGGGCGACTGTAGGTGGTCAGGTTGC
TGTCGCTGTTGCCGTTGCTGGCTGTGCAGCAGTTGATGGAGCAGTCATTGT
GACTGTTGCCTGTGTTGGGCCACGTGTCCGCCAGCCATGGCTGTGTGGCAG
GTTCACTGATGTTGAGGAGTCCCGGCCTCCCTCCACTGCTGACGGCTTCGC
CTCCTCTTTGATAAGTTACTGTTGGTGTGAAGGTAAAGGACGGGACTTTCC
GGATCCCCGCGTACGTGCTGCTGAGCCCGTTTCTCTTCTTGCGGTGTCGGT
AAAGCCAGATACTGAAGACCATGAGAATGATCCAACAGGCGGCCCCGAT
GCCCGCAATGAAGGC
>LNC_002643
CTCAGCATCGGGCTGGAAAGTGGGTATCAGGTATTTGGGGAAGCCTCAAG
GGGGCCAGTTCATTTCAGTTGACTTCCGGGGCACTGATTGCTTCTCTCTCA
GCCACTGGCTGTGGAGCTTGGCACAGCTGGCTCTACCCCTGCCTCTGCGAG
TGCCTCCGATAAGCCCCGGTCTAGATGAGGGGTCCTGGGTGGCCTTGTGT
GAAGAGGAACCAG
>LNC_002770
TCTAGATCTATTTACCATCTGTCTGTCATCTATCTATGTGATGCTTAGTTTA
ATGTGTCAAGTTGTCATAATCTAGAACTACCTGGACTTGGCCTATGAGCAT
GTATTTGGGGCAAGGATTGACTGTTAAATCATGTAGGAAGACCAGCTAGG
TAAAAGTAGGTGGTGACGTTCCTGGGGTTGTGTCCTGGACTATGTAAGTAT

AGAGAGGGAATCGTACCCAAGAGTCATGCGTGTTTTCATGGGTGGGTGTT
TAAGTTCCTCCTGCCTTGAATTCTTATCTCTGATGCACTGTAACCAGAAAC
TGTGGGTAAAATAAATCCTTTCTCCTCTAAGTTGTTGGGTTTTTTTTTTTTT
CATTTTGTTTTGTTTGTTTTTTTATTTTTGGGTTTTAGAGGATTAGTTTGTTT
TGGTTTCTGTTTTGTTTCAGTCTGTTGTTTTGTTTTGCTTTGTTTTCCTCAAG
ACACGGTTTCTCTGTGTAATAGCACTGGCTATTCAGGAACACTCTCTGTAG
ACCAGACTGGCCTTGAACTCACAGAGATCTGCCTGCCTCTGCCTCCCAAGT
ACTTGAACTAAAGGTGTGCGCCACCATGCTCAACTACATTGTGTAGTTTTT
TCTTGTTTGTTTGGTTTTGGGTTTTGTTTGTTTGTTTGGTTTTCGGTCAGTTA
TTTTATCACTGAACAGAAAGGAAATTAGAACACTATCATCTAAATACCCT
CTATCATCTGTCTGCCATCTACCTACAATCTTATCAATCATCTCTGTGTCTG
TTTATATATTCTCTATACTGTCTTTTACCTATAACTCTCACCTATACATCTC
TCATTAATGTACACATCCATCAATCTATTCAATCTATGTATCTCTTCCCAGT
GGCGTGTGTGTGTGTGTGTGTGTGTGTGTGTGTGTTTACTTTTGAGGTATA
TTGTCATTGTGTAGCTCAGGCTAGCCTCCCAACTCCTTGTCTTCCTGCCTCA
GCCTCTCCCATGCTGAAATTATAGGTATGCATCACCACACACGTCAACCTA
GGAAATTTCCCATCCAGCTACCTGATGATGGATTTTGTTGACTTTCATTGG
GTTTTTGAACTTTCACAAATACATATTAACAATAAATTTTTATCACAGTAT
TTTACTTATGTACTCCTTAGCTTAAAGAGTGACCCTGAGTTCTGTAACCTG
TAGGCTGTTCAGACTCTGGATCTGAGGCTTAAGGACTTTTAGGGTGTTCTT
GAAGGCAAAAGGAGTTGGGTGTTGTTATTGTTGGTTTGTTTTGGGGCAGG
GTCTTTCTTTATAATCCAGGCTGGTTCAGAACTCCCGGGCTGGCCTCAGAC
CCACAGAGATCCATCATCCATCCCTGCTTTCCAGGTCCTAGGATTAGTCTT
GTGCC
>LNC_002957
GGAGCAGGTGCCTCGGTGTCTGCAGCTTCTCATCAAGTCTGAAGCCTCCCC
ATCTCTGGGCCGAGGACTGGGCATTCGGGTGGTCTCAGAACCATCCTGCT
GGACCTGGAGTCCCAGCTCCTCTGGGCCCATATTGTTGGTACGCGCATGTA
CACTGCCTCTCTTATGGGCCCTCAGCCCTGCTTGCTCTGCTTGCGCTGCTCT
GC
>LNC_003007
CCCCAGTCTGGTTCCATAGCTGCCCCTGCTAGGCTATGACAGGGAGCCAC
CAGTTCCCACTCAGCTGAACTGAGGGAAGGGGAAGCTGGCTTAGACAGGA
TCCCAGGGGCCTCAGCTTACATCCCTGGGTGACCCAGTCTGTTCCCTGCTG
GGCTAGCAAGTTTGAGGGTCAGCTTGGTGTGTGCTCAAGGTGACTTCTGTG
GGGACAGGTTTTCTCAGGCTACAGGACAAGCTCAGTTTGGCCTGACAGCC
CAAACTCCCTCAGGCACCTGGCTGGTATGTGCTGAACCTGGGGGTTTTAGT
ACCTCTATCAGGGCCCAAGACCAGTCCCTCTGCCTGAGCCTGCTCCCCTAG
ACCAAGGGTACCTGGGGTAGCCTAGAACCAGGCGGGTTAATCAGAGGCCC
TAGGCTCTCTTCATAGGCCTCCCCCCTTTCTGCTTCCAAAATGCCACTCAT
ACACAAGCTAAGGCAGCCAGCTAGATGAAGTTGCTTCTCAGGTCTGTGGC
CTAGAGTCTAGGTTGAGTTTAGGTAGAGGCCAAGAGCTCTTTGAGGTGGA
AGGGACTTTGCCAGTCTCCTGACTCTCTGTGGAAACCAGTGGTTTTCCTGG
GGGTGCGGTGTGGGGCAGACTGTGCTTTATTTAGAGTAAAAACACCCACT

TTCTGGCTTGGCCCCTGGTTCAGCAGCTGGGAGAACGGAGGGGACCTGT
GCCTGCCTGCCCACTCCAATTCCCAGGCAGCAGTAGGTTCGGAAGGGCAG
GTTCACAGGGCTGCCTGGCAACTTCAGACAGCCCTGCCTCCTTCCCACCCC
TTGTGGACAACACAGCAGGATGAAGTGACCAGGGCTCCACCCCTGCTACG
CCTGCCTCTGCCACTTCACTACCCAACCGTGACATCAAAAGAACCCCCAC
AAGGCACGGTACAAGGCTGCTGAGTGCTCACCTCACCCTCCTCCCCTACCC
ACCTAGCTTATGAGAACCTTGGAGGACGCCAGTACTCACTGTGCCTCAGA
GTCCTTGAAAAGCAGCTAGCCGTGGCGTGGTGGCCAGCCAGGGACTGCTA
TGATGGGCAGTTAGTGTCCCTGTGCTGTGTGTGGCTCCCTCCCCAAACACA
GTGTCTACTTCAAGAGTGGAGGTGTGGGGGTGAGGGACCTGCCTTCAGCA
CCAGTTCAAATGAACTCTTCCTGAATGAGTCAAATTCCCAGGGTCTCCAGT
TGGCTTTCTCCTGCTGGGTGAGCTTCCCTACGAGGCTGTGGAAGGGTTTCA
GCAACAAGAAAGGACTAGAAGCCTGAGCGAGTACACTCTGCTGTCTCTGA
GCCATCTCTGGCCTCTGTCCGGTTTCTCACTGGGTCCCAGGCACTGATTCC
CTCAACGGGACCTTCTAGCACTTTGTCATCTACAGACGTCATTCAGCGGGA
TGTGGGGTTTCTTCCTAGTGGTTTTGTCCCTAATACCAGACACAGACACCT
GTGATGGCTGGAGGACAAGTAGACAACAATGCAGGGACGAAGCCCGAGG
CTACAGCAAGGTCCCCCAGGGAATGCTGGCATTGCTAAGATGAGACAGCC
TATTTAAGCAGCCTTCTTGAGCAGGAGGGCTTCCAGAATTTTCCATGGTAG
GAAGAGCTCAGCCAGGTCTGAGCCTCCAGCCTAAACCAATTTCTGGAGCT
CAGGAAGACAGAGCGAGGTGGTTCGGACGGACCCTGGCTAGTCCTTTTCT
CTCTCTGTGTAAATATGTGCATATATATGAGTGTGTCTACATACACACATG
GTTTCCATACATCGTAGTACATAATACTATGGTGTTATTGCATAGAAGAGG
CTGGCTTGACGGAGCCGGGCTAGGTGACGGTCTGGGAGCGCTGGCGACTG
CCGGGGGTCTGCTGGGCTGCAGGGCTTGGCGCGCTGCCCCTGAGCTGCTG
CTGCTCCTGTTCCCGGGCATGCTCCTCGTACCACTCCTGGATCTCCTCCTG
GTACATCTTCAGGCTCAGGTCACTTTTGGTTCTCGATCGCCGTCCCAGAAA
CACCATGGAGTTTTGCTGGTATTTCTCCATCTGAGAGAGGGTGTCGAGCAG
CCGGGTGATGTCAGAGGAGGTGCCTCGCGCCTCTCGATGAAGCTCCAGGA
TGGCCAGAAGCTCCTCCTTGGACATCTCCTTCTCGATGACGATGCCACTGT
CCACTTCCGATTCCTGGCTCTTGCGGCTGTAGTTCATGCGGAAGACGAAGG
CCTCCAGGATGAAGGCCACGATAATGGTCATCACCACCATGGTCACGATG
TAAAAGGTCATGAAG
>LNC_003069
TTTCTTAAAACTTTTGTCAGTGAAATGTGGTCACACCCATGTTTCTAGAAT
ACTGGTACTTTGGTTTGCAGAATGTAAGAAACTAAAGAAAAAGGGCAGGA
AGTTTGCAGGTGTTCATTTGATCGTTGATGGCCCTAGTTTTATACTATCATT
ATCATTGGTCTTTAGATTCTATACATTTATTGTATATGTTACTTTGTGTATG
TATGTGGGAGAGTACAGGTCCCATAGTGTGTGTGGATAGCACAACTTGGA
GGAGTCAGCTCTCTGCTTCTACTGAGTGGGCCCAGGGATTGAACCGAAGG
CCTCAAGCTTCTGCAGCAGACGCTTTACCAACTGAGCCATCTCCCCAGTCT
GCGTTTCATGTCTTACACCTGGCCGTCTTTGCTAGTTTCATTATTCTCAGTT
ACTTTCTTGTTGTTGTGACCAAAATACCCAAGAGACAAGTTCAAGGGGAA
AGGTTTATCAGAGCTCATAGTTTCAGAGGGTTCAGTCCTCCGTTCATAGGG

TTGGGGGGTACAGCAGAGGACTCAGTCCATTCATGTGGGGAGAGTACAGC
AGAGGGCTCAGTCCATTCATGTGTGGGGTACAACAGAGGGCTCAGTCCAT
TCATGTGTGGGGATGTTTACAGCAGAGGACTCAGTCCATTCATGTGTGGG
GATGTTTACAGCAGAGGGCTCAGTCCATTCATGTGTGGGGATGTTTACAG
CAGAGGGCTCAGTCCATTCATGTGTGGGGATGTTTACAAAAGAGGGCTCA
GTCCATTCATGTGTG
>LNC_003254
CCAGTTTTAGAGTAATTTAGCACATTTATATGTGAAAAATACAAAATTACT
CAATACATCTTTAATACACAAATACATATACAAACATTTTATTCTAATAAT
ATTCTTTATCACTTCAATTTAAACCATTCCATTATGAAAATTTCTTAATTGA
AGAGAGAATATTCCTTTAAAACTCTAAATGAAACAGAGCTTTCTCAACTA
ATTTATAGCCACACAGCCTTTAGAATACATGATTTTCCAGTTCACAAGTCT
TCCCTTTAAAACTGTTACGGCTTGTCATGGTTTTTAAGATAGAAGGCCCCA
GACAGCAGGGAGGACCCCAGCGTGCAGTGCTCAGAGCAGGTCTTCTCAGG
TGCCCACCCACCCTCTAAGTGCCACCAACCTCGCATGAGCACACTTGCCAC
ACTAACATTTCTTCTGAGTGCTCACTTGGCCATCTACCAAACTCAAACCAG
AGCCTGCACCACTGCAGGCGTGCAGGCTGGCACCCGCCGTCAGAAATGCT
CATCTCACGTAGGTGAGCTCCCGGACACTGGGAAGTATGTTAAACAGGGT
CCCCAAAGGAATCCCGTTCCTTGAATGGTCCACCTATTCCTTAACTGAACG
GACACGTAAGGCAGGCTGGTCCTGGACAGCAACTGGACTGCACGCACTAC
AGGGAACGCCCTTCACCCTGAAGAAGAGGCACACGTGTGGACGATGGAG
ACTTTCAAAGAACTGAAATGGCTCCTGCTCAGACTAGGCACTGAAACACC
TCTACAAAGAACTCGCCAGATTGTCTCTTCACTGTAGAATGTTCCATTAAA
CTGACTACAGACCACAGCAGCTAAGCAGCTCCATCCTCCTGAGAAACGCT
GTGCATCGAAATGAGGCTCTCGCACTGTTCACTGTCTTCTGAAACTCTCTG
AATTCGATGTTTTCTGCATATAAAGGCAAAAACTCTTCTGACTCCTACCAT
CACAGGATCCCAATTATTATAATGGCATAGGAGAGTAACAATAAAAAATG
AAAAAAATACAGGGATAGCGCCTGCAAAAAATAACCAAGAAAACTGCAC
CTTTTGCATGCACATGTCCGCTATGATGGAGAGAGGTATCGTAAGGCTTA
GTGCAAGTGTGCCTATCAACGATGAGGTAAGAAAGCAGCCCCATAACCAC
AGAAACTCGGACAGAACTGTTCCAATGAGGCCGTTGATAATGATACACAG
CAGTACCACTTTATTGGGAAACTCGAAGTCCTCAAAGCCAGTATAATGAA
GTAAAAAGAAGCCCGGCCACAAGAGCAGCAGATTAAACAGACCTACAAA
CCCAAAAAACATTGGAATATCCAATTTATCTTCCCTGTCTACCTTTCTCTTG
ATCATCACAATGTAGACAGCATAGAACATAGCTCCAGCAAGGGACCAAAT
GGAACCTATCGTGTCTCTTCCAGCAGACTTTTCAGATCCAGACAGGTTCAC
CAGCACCACGCCTCCAATGCTTAAAATTACAGCTAATAGTTTAGAGAGGG
TAAACCTGTCTCCGCTGTTACTCGGAAATACAGCTGCAAGGATTAAAGTA
AAAAGTCCAGAAGTTGAAGACAAAATATTCACTATGGCAACCTGTGTATC
TGAAAGTGCTTCTTGGTATGACAAATTTGCCAAAAACCACACAAAGCAAA
AAAAAAAGCTAATTTTTGCTACTTGGGTTGCAGTCAATTTCCCTACAGTCT
TCAGTATGGATTCTTGGTCTTTCACAGTGGGATAAGACATTCGAGACAGCT
TGGCCTCTAACGCATGGCTTGATGGGAGCTGTCGAATCTCCATGATATTGC
TAAACCTTACACGGGACTTCTTGGGAGGTTTTTCGGTCCCAATGTTTGTGC

TCTCAAGCTTCTCACTTGGAAGATCATGAAACTTGACAGGAACATACAGA
GGTTCACTAAAAGCAGCAGGCTTTCCTCTAAATCCTCTTGTACACTGTTGC
CTCCAAGGCTTCCAAATAATGAAGCCCAAAAGGTAGAGAACAAACATGG
ATGTTTTCGCAAAGGTGCTGAAGAATGGCTTGTTGTATTGCGTGAAGACAT
ATGAGGTGAGTTCTGAGGACGCCACCCATATCACATCAACCAGCAGGAGA
ATCACTATCCCCAGGGCCATTCGCCTGCGCTGACTGAAGCCACTGCTCTGG
GAATTCACACGGTTCATGATAAACACATAAACCATTTGCAGTCTTGTGGC
GAGGGCTTCTCTGAGATCCTCAAATGCAATGCGGGCAAAGCGAGCAGATA
CCTGTCTAAATGGAGGTGAAGAGCTCAGCCCACCTGGCCTTCCTGCCCCTC
GATGGAGTCGTGGCGGCACCATGAGCGGCCCCGGCCCGGCCCGCCGCCCA
GCGCCGCCGCCTCACACCGCACGCACAGACCCCAGCATCCTGCCGCACAG
CAGACCCAATCTCAGTCACCGCGCTACCCCTCGGCTTAGGGCCGCGGCTG
TCAGGCAGGCCAGCACTCGGCCCCACTGAGGGGAGTGACCCAGGCCACCC
GACCGACGGCGCTGGCAGCGAAAGTCCTGGAGGCCCGGATTGCGGCCGC
GGCCCAGGCCCCGCCCCCGGTCGGTCAGCCAGCCAGGCCACGCCCATTGC
TGGCCAAACAAGCCCATCCCACGGCTGGCCAACAGGCAGGCCCCGCCCCC
TGTCCGTCAGTCAAACAGGCTCCACCTCCCGACCAG
>LNC_003267
AATCTTACTGCTGTGCGGCCGTGGCATTCCTTCCTGCTATTAACACATGAA
ATGGTCAATCCAGTGATTGATTGACATGGCTTTCGTGGGGGTGGGGGGGC
TGGCAGAGAAAAGACTGAGACAAAATGGCAACTTAAACACATCAGCATA
CAATGGTTTTGAGAAAGATAGATGGTAGAGAGAAAAACATTATTCTAGAC
AAATGAAATAGCACTGAAAGCCCATGAGTCAAGCCACAGCCCAAACCGT
GTCCTACATAGGTTTCCTTTTTTGTTTTGTTTTTTTAATAAAACCTCTCCCC
AGTATCACTTTTAATCTTCCCCAGATGCCGCTTCATCTTCAGACCCTTCATC
TCCAGACTTCTCGAGTGGGGGGCTTGCAGATGTCTTCTTTTCTGGAGGGCT
TTCTGCTTCCTCGTCCTCTTCTTTGGGAGAAGGAGTTTTTTCCTTTGATGTC
TTTGAAGCCGTGGGGCGACCCACTTTCCCCTTGCCTTTCACTGGACTTGGT
GTCACTGTAGCCTTTAGTCTGGGTTTGGGCATTTTCTTTTCTTTCCTCTCAG
GTTTGGACTTCTTAACCATCTTTTTGTTTTTCTTTTTTGAACTGCCATAATC
ACTATCATCGTCGTCCTCCATTAGGAAATCTTCGTCGCTGCCAGAATTCTC
CTGGAATGGCGCCTCATCATCTTCTTCCGGCTCCTCCTCGCTGCCCACATC
TTCCAAGAGCATCTCTCTCTGCTTAGAAGCTGCTTTTGATGCTGCCTGCCG
CTGCTGGCGTACACTTTTATGGTCATCTTTTTCATCCTCACTGTCTTCTGC
>LNC_003383
CATTCTGCCACCTCCTCCTGAGCAATTCCCTGCTTACTTTTTCCTAGTTCTT
TAGGTACAGCGAAGGTGCATTCTCACTGGACAATTTGCACGCCTCAAAGG
TGATTCTGGCACCTCCTGGGCTGCTTACACGCACGCACGCACGCACGCAC
GCACGCACACCACTGCTCTCTCACCATTCTGCACTAGAATTGCCTGCTTAG
CAATCTTGACTTTTCCCTGCTGGAACTTGGATAACCTATTGTCTGAATCTC
CTAGGATTTGGCACACAACATACACATAGTAGATATTTGCTAGATGAGAA
AATAATTTGATTCTTACCTTTCCCTTCTGGTTTTTTCCAATTTGTCTTTTAAA
ACCATAATTTCTTTGTTGAGAGCAAGTTGCTGGCCTTCTGAGTCATGCAGC
TCCTTCTTCAGATTTTTAATCTCACTTGCATGAATTCCTTCTCTTTTAGAGA

TTTCTTCTTCATAAAAGATACTTTTCTTTTCCAAGTCAGTTTTCAGTTTAGT
TATCTCTTGCTGGTGTTCTATGCTGCATATTCCTGGTGAGTAACTAATTTGT
TTTTGCTTCAGTCCCTCCAATTCATTTTCCAGCTGTTTAGAATAATGTTCGC
TCTGCTCTCGGAGCTTCCGGTCCTTTGAGGCTTCAGCAATTAGAGCTTCAG
TGTGAACCTCCAGCTCTTTCTTGGCTCTTTCTGCTCTGCGAAGCTCCTGCCT
TAAGCTCTCAGCTTTTTGCATCACCAGGTCCACCTCTTCTTCCTTATCTCGG
ACATGGCGAGCAAGTTTCTGCTTTTGGGTGTGCAATTCTGTTAGTCGCTCA
TTGATCTCCATGAACTCCTGCATGGCCAGTTTCCTCTGACAGTGTGCGTCT
TTCAGCTCTTTGGATTGGTTTTTAATCGCTCACTAGCCTGGACTAGTTCCT
TATTTAGCTCTTCTCTTTCCTGCTGCAAAGTTTTGATTTGTTTTTCAAAAGC
TTTGATTTGCCTAAAAGCATCATCTAGTTCTCGTCTCACAGAATTAGCTTC
TTCAAGTTGTTGTTCCAAATGATTCACTTCTGCTACTTGTTTTCTTAGTTTT
TCAATCTCTTCTTTCAGACTTTTAATTTCTAAATCTTTGCTTGCCGTTAGTG
GACCATCAACAGTTGAATACTGCAGGGCTTGAACAGTCTGTGTTGACTCTT
GAAGTTTTCTAGTAAGTTCAAGCTTTTCCTGTTCTAGCCGTTTAATTCTTCT
TTCATAAGCTTCAGTTGCTAAGTTATTATCTAGAGTCCTCTGGACATTGAC
GTCAAGATCCAGTGAGGTGGGACCTGCTGTGACTCTTAAACAGCTCCGAT
CAGAAAGAACACAGCTACTAGTATATGTAAACCCAACAAATGGTAGATGA
TGGCCAGAAAATGCAGTATGTGTTGGTGGGGGCATTGTTTCAGAGTTTTTT
AAACAGTCGTCATCCACATCGAAATTTGACGTGTCTGTTGGACTACTAACT
TCTGGAATGTAAGGTGCTTCACAGTTTCGGATGTTATCCCAATCAATTCCA
CTGAAAAACGGATGTTTCTTAAAGTCTTCTATTCCATTCTGGCCAAGTCGA
TGTTCTCTGCTACAAATGAGTCTTCGAATAAGATCTTTAGCATTTTCAGAC
ACATCAGTCACTTGGGTTGGAAACTGAAACCTCTCTTTATGATTCATAATT
TTTCCATATGTCTCCACCAGAGATTCCGCATAGAATGGTGTTTCTCCGTAA
AGCATCTCATACATGCAGACTCCCAAGGACCACCAGTCACACTCTGGTCC
ATATCTGCCTTTCCCGTCCTCCATAGCCTGAAGGATTTCCGGGGAAATGTA
GTCTGGAGTTCCAACTGCCACTGAGGACTGGACCGTTCCATCTTCCATCAG
CTTCAGACAAGAACCAAAATCTGCTAAACGAATATGTCCATTCATATCCA
TCAATATATTGTCAGGTTTGATGTCTCTGTGAACATAGTGGAGCTGGTGAA
CTGAGTCAATGGCTATCACCATCTCAGCCAAGTAAAACCGAGCCATCTCTT
CTGGCAATCGATCTTCAAATTTGCTGAGCAGAGTAAGTAAATCCCCACCA
ACATAATAATCCATAACCAGGTATAAGTTATTGTCATCCTGAAAAGCATA
GTGCAGAGTGGTTATCCACTTACTGTCTCCATTTACCAACACATCCCTCTC
TTCACGAAAGCACGCTGTCTCAGCTCTTTTCAGCATTTCCCACTTGTTCAG
AATTTTCATGGCAAATACTTTATCTGCATTTTTCAGTTTAACTACAGCAAC
CTCACCAAAAGCTCCTCGACCAATCACCTTTAATATTTCGAAGTCTTCTCT
ATGTAAGCGCATTTGCTTCACTTTAGAAGTGAATGGTTTAGCCCATTCTAG
ATATTCAAGAATGTTCTTCTCTCTTCTCAATGGAGAATTATTGCATTCATC
ATAAAGACAGATGAGTATGTCCAGTAACGTCTCCACACTGAAGCACTGCC
CATTGGTCTGAGCTGGCCCATCTAAAATAAACTGTTCCAACTGCCTCAAAC
GCACTTCTCCAGACATGTTTGCTTCGATTTCTGCTGGTTTTCTTTTAAATGC
CCTTACGATGTCTACTGTTACTATCTGAACCTAAAATTTTAAAGATGTGGT
TTTTAAAAATAAACCACTTGTTATTTCAACAACTGTCATGCAATGCCGGTG

CTGAATTAAACATCCAATACACCAGTCACCTCACTTAACGGAAGCGTCTTC
AATTTCACCCATAAACATATATATTTTGAGGGTAAGTTTCCATAAAATATA
GATTTAATATTTTTAAGAGAATAATACAATTAAATTGTATATTTAAATAAA
ATAATAATTAAAAGTACAACGAACTAAAGAATCAACACTTCTTTCAAAAA
GAAAAAAAAAACCTCCTTCATTCAAAATTCAATTCGTGCAACATAATCCT
GAAACGAATCCAGTTGCATCATTAATGAATAAAGTCCCCTTGGCATCAGC
AATTCACTTCCCGGGAAGAAGAAAAATTGAAAAAGGAGGGGGGGAATGT
ATGGAGGGGGAGGGAAAGCCAAAAATCTGCTGCAAATGCTCCCAACCTCC
ACTCCGATAGCTCCTGGGCGAGTCCTCCGCGGCAAAGGGAGGGCAGCTCC
CGGAGCGGCTCTCCCGGCTCGGCTCGGAGCACGCCGAGCCTCGGCTCCGA
GCTCTTTCTTCCTAGGGCTCTGCAGCGAGTCCTGCTTCCAGCGGACTGCGG
CGAGGACGCGTCATCTTCTTCCTGGCTTCTCCCAGGGCCTGGCCGGCGGCG
CGGCCGGGATGAGGCGGGCTGCGGGCGGCTCTCGCGCCGGGCTCCGGGG
GCGGCGGCTACTTGGCCGCG
>LNC_003435
CTGGTTCATCGCTCCGTTTATTAGAGTAACCACATCTGGAGAGGCACAAA
GCACTGAAGTTTTACACGACTACCCAGCTCGCTCGCTTGCTCACTTAAAGA
ACAAGCTTTCAAGACACCCGAGTCAGCTCAACATAGACCCAGGCGGCACG
GGAGCTACTGCTTCAAGGCTGCACTGCTTGCTCCTGCGTCGTCCTCACGTG
GGAGCTGGCAAAAAAGGACTGAGATGTCTATGGTCACGATGCAAATGCTG
TGAGGGCAGTGAATCGATCCGCACAAAGTGCAAAGACGCAGTGTGCACG
GAGCTCCAGGACTCACTGGGCCATCTGCGCCTCCATGGCTTGCGCTGTCCA
CTCGGGGGCTGTCTGACTAATCTTCTCCAGGAGGTTATTTACTTGGAAACA
AAGTGATTGGATCTGCTTGTCCCACGTCGGCAGGGCTTCTCGTGTTTCAAA
ATGAACTATCCCATCGATCTGGTCAATAAATCCGTTCATGCGTCCTTCTGT
TATCATCTGAGATGCTATCTTTTCTGCCTTAGCTGCAGGGATCTCTAAAAG
AGCTCCGAGTTCCTCAAAGGTGATGTTGTTATATAATTTGCTTGCTGACAA
CAAATTATGCTCAATAACAGCTCTGTCCAAGATGCTGGAACCATCAGCTG
TGGTTGCTTTTTGGTGAGGCATCAGCATTGCAGCAAATTCTTGAAGCTGGT
TCCCTCTGATGATCCTGTCTAGGTACATTTTCTCCAGGATCCCATAAGCAG
CCAGTTGCTGGCACCTTTCATCCTTAAAAAGGGTAGCCAGCATCCGAGAG
CGCTGCTGTCCTGCCGACGCTAGGATGGTGCAGTGCAGTGCGTGCTTCAA
GGCCTCCAGTCTCTCACTTTCGTGCACTATTGTCTTGTAAGAGAGCTCATT
GTACCTTTGGGCGGCTTCAATGAACTTCCTTCTGTAGTCAAGGACACGTGC
ATAGCAAACCTTATAGTGTATCTGTAACTGTTCGTTTGTGGATTCATTCTG
AAGTAAGGAAGCTCGGTTTATGTAAGCCTCAGCCTGGACAGGGTCGTCAT
CCTCCAAGTATAGCCTCGCGATCTTCAGGTAGGTCTCCAGCTTATAGTCTA
CGTTGTACTGTTTTTGTCCTGTCTCCAGAGGAATCCCCACCAACACTTGGG
CTGCGTTTCTCCAGTCTTCTTCCTTCTCATAAATAGATGCAAGACGCTGTC
GTATGGAAGCCACCTGCTCCTCAAAGGAGATGACCCGAGGCTGGACCTTC
TCCAAGGTGAAATGGTAGACCTCCTTGGCTGTGCTGTCTGGCAGGTTGGG
GAGGTGTGTGCAGAAATCTGTTAGCAGTTGCCGTGAGATCACAAGGCTGA
CGTTCTCGTTCACCATCGCTTCCACAAAAGCTTTCAATGCTTCTAGTTGTTC
TGTCCCAGATAACTGAATGGCTTTTTCCAGAATCTGACGATATTTGCCCGC

CAGATCTTTGTGAGAGCCGCTTGAATTCATGAGCTGGGCCAAATCCTGTCG
CACGGCAGCCGCCATCTTTCTTCCAGCTCAGCCACGGCTGCGGGACCTCTC
GC
>LNC_003525
GTCCGCAAACTGTAATATAGAATTTAATTTTATCTTTATGAAGTATCTGAA
GTCAAAGACGATGATACATGCATTGGGCACATCCCACAGAAGACATTTAC
AATGGTTGACATTTCCTTTTTATTTATTCCTAGAAACTGCCCGTATTGCAG
AGTTTTCATGGATGAAATATGGAATTGAGCAGATTCCTTTTTCTACAAAAT
TATGGTATAAACTTGCTCGATTTTCTATCTTATTGCTAATAACCCTTACAG
ACGCACAGTTTGTAAAGTAAACCCCTTAAGGACTGAATGTGTAGAGCATG
CTGCAAGGTACAAGAAATAAGCTAAAACTGAGCTTTTACATAAACCTACA
TCTGGACCGACACAGTACTGAACACTGGCAAACATTTATCATGACACAGG
GAGAAATCAATAGAAGGCGATACAGCTCAGTCCTGAAAGGGTTATTATGT
TTCTAACCATCCATCCCAAGTAACAGTGCTTGAAAAGTAAATCAAAAGTG
ACAAGAAGTTCGCACATTTGTTCAGTACTCACCAGAGACACAGCTGCTTT
GGAAAACGATGTCTGTTAACTTGGTTAATAATCAATTAACATTTTCTTTCA
AACAGGAATCTCTTCCCACAAGAAGATTTTGATTAAATAACTAGTGGATT
AATTATTAAAAATTGCATTGGAATTCCTCTGTGGTTGAACACAGACCAGGT
TCTCTAGGCAAAAGCAGTACAGGCACCCGGCCATAGCTGGTACCCACCCC
AGGACCTGGAGACTAAGGCAGGAACTTTCAAGAATGCCCTGAGGGGCTAC
ATGGGTGAGACCCTTGTCTCCTGAAAACACTAACAAAAAGCCTTAGCAAA
GAAGCCTGAGGCCCTAGATTTGTGACCCCAGCACCACACAGAAAAGAAAC
AAAATTGTTAAGGTTAAGATGGTGGCAGTAAGAATGGCAGGCATTTTACT
GTACACAGGGAAGCCGCCGCAGTCTGAGTAGTCAGCAGCTCAAAGTCCCA
GTGTTCCGCTCTGCAACTGCACAAAGGACAGCCCACATTTTCAGTTTCTGG
CCTGCCTTCCAATCTCTTGACAAATTCAAGGAGCTGAAATACACCGTGGGT
CTCGGACTCTGCATTTAGACATGTTTTAAGAAGCTGTGTAGACTCACTAAA
CAGAATGGAAAAACACACCCACACACCACGACTGAAGAATTCCCGCTCTA
AGAAGGTAAGAGGATAAAGGGCCAGCATCTACAACAGCATCAACAGGGA
GGGAACATTCCACAGTAACCGGGACAAGACTCCTGTACATCACATACTGA
AACTGACTTACAAATCCACAGAAAGGACTTTTAATAAAAGCAGCTGACAA
AAGAACTATCAAGAACATGGTAAAACAGACGTATCTGTTCAGGCTACTCT
GTGGTAGGAGTCTGACATAAAACCATAGGAAAATGCCTTTATTGTTACAT
TCTTGAACTTTGTAAAAAAAACAAACAAACAAACAGGAAAACGACTTATA
GTCTAATTGTTTCAATTCTTACTAAAATAAACAAAAGAACCGAAATGAGA
TATAAGCATTTTAAAAAGTCAAAGTTCATTTTAATAAAGCCATGAACTTGA
AAACTTACTTTAAGCTACAAAGCTGGGTAAGGAATAGTTTGTTCTTCAAA
AGCATCTCATCTGTTCTCAGTTAACGACCTAACTAAACCCTAAATCTAGGC
CCTCAGAAAGCACAGCGGCACTGCAAACTGCTCCAGACCGCAGTGCCCTG
CTTCCATGGCAGCTGAGGAGAGGTGCTTGGAAAGAAGCAGGGCTGAAGG
GAACCAAGAACAGTTACTGCACAGATAGGTAAGGCCACAGTGAGCTCCGC
CCTGCACCACTGACACAGCCTGGAGGTGTGAGGTGGGGAGGCAGCGGGCT
GCAGCTGATCTCAAGAGAAATGGCTGTCCAGCGCAGCCCCAGCTTGGGGT
GAGTAAGAAGGCTGTCACCACCACATTTTACAGGTGGCCTTCCCCTTGAC

AAACAAGGAGGAGGCAGTTTAAAGGTGTTCGGAGTGGCATTTACAGTGGC
ACAGTCTATGAGACAGAGAGAGAAAGGCAGCAAAGCTGGAAAGCAATGG
TCCTGACGGGAGGCTAAGGGTCTACGTGAAAACTTTCAGACGCCCAGATA
GCCAGAAAGACAAACCGCCATTAGAAGTTAGTCTGCGTTTGTAAGGTACT
AGTGGCCGCTGTTTAAGGGACAGTAAACGCTTACCACAATCTAGAGGTAG
TTCTTACTCAAGGATGGTGGCATCACTAGACTGTGTCCTTTAACATCAATA
TGTGGTTCTTGTACTAAATGTAGCCCAATGAGTCTGCTGCAATTCCTAAGA
AACTGAACAAGGTTTTACCCAAAGTGCAGGGATGGATGGAAACAACAAA
ACTGGCAATATTAGCACACAGGCTGACACACACGCCTGAAGGTAAGCAAA
GCTTAACTTTTTAAAAAGGGGAAAATTGGACAGAATATTTCTATTGGGGA
AAAATAAACAAAGGGGCAAAGAGGAAAATGGAAATATAAGTTACATTTG
TATTTTTATAAACTTACAACTATCTGTCTCTAAGGTACTGTCTTCTGGCTAC
TTAGTTCAGTTTATTTAGTTATCATCTAATAAAAGCCATCTTATGGTCTTAA
GTTTAAAATTATAATGATGTTCTGGCTAGGCTAAAAAAAAGGAAAAAAAA
TACAATAAATCATCCTGCAGAGACTTTCCTCATTTTACCCTAAGGGCACCA
ACTTGGTAGCAAATAATTCTCATGATATTTTTTGCCATTTGCCAACAAGGT
AGGAAACACCCTGTTAAATGTAATCACTGGGAACATTCCAAGTAAAGATA
AAATGTTTAAAAATGTTTAACTTACACATAATACAAGTTATGTACAGACTT
GGGGAGGGGACCTGGACGACTCCTGGAACACAGATGAAGTGATGGACAG
GCAGAGGCGCACAGCTGTCAAATGGCGGTGTTCAGTGCCATCAACTCAGG
ATCACCGAACGGCCACCCCGACTGAGCTGTTAGCATCTCTTAGATTACCAC
TATAAATACATTTCTTTAAAAAGGGGAAAACACAGAAGTAACTACCAACA
GTATCTGAGAACAGACTAAGTTAACATACATTGCATGTATTGCAGGCAAG
GCAGAGGCATTTTTAAAGCTTTTGCAGACTTCATATAATCATAAAAAAAA
TATGCAGGCCTTTACAAAATTTGACTTGCTGAAATCAAAATAATTCCAACT
TATGAAAAAGTCATAAGACTTCAGCTTAAAAAAAGTTCCAGCCTTAGACC
AAAAGAACCTGGAAGTGTTCGGTACTCAGATTGAACAAGCAAGGTCAGGC
TAGAACCTGATTAGACTTTAGAGCCAACCCTCAACGGGTGGGGAGCGCAA
CCTGTTTGGTAACAAGGTGTTTGACATACTGCAGTATAAAAAAGGCATGG
TGAAAATGTACCTTTTACTAAAGCTTATGCAAGTCCCTTGGTCCATAAAAA
CTAGGTTAGGTTTGTGTCTTCTAGCTTAACCTGTACTCAGCCGCATGTCTA
TCTCTTGACCATTAAATCAAAACCAAACCAAAAACAACCAAAAGGGGTGT
GTGTGAGAGACAGCTGAAAACTGGATTTCCAGCAGCCTGTACATTGTGAT
GTTGTTCATTTCACTGTGGCTTCTGCATTTCACATCTGCACTTGCAGTTTTG
AATAGATCACCTGGTATGGAACGTTTCCCCAAGAAACCACAAAAGATTGT
TCACTTTATCCTTGCCTTTTTCTGTCAACTTTTTGCCGCATTCAAGTCAGTT
TAAGTCCCAGCAAAAAGACGGTAGTTAGGACACCACGGTTGCTGTGGACG
ACGTGACACTGGTAGAATTTGTGCTGGCATTTGTGTAATTTCCCTCGCTGT
TTGTGTTTGACTCATTGGTGGGCACTTGGCTTGAGTTGGCTCTGAGAATGG
CTCCCGCTGCGCTGCAGTGCGGCCTTGGCCCTGGTTCTGGGGTGTAGGTAA
AGGTAAGGCTGGTGGAGTAAATGATTCCATCATTACGGACCAAAGTTACT
GGAACCTGGACTGGCTGCCGGACCCATCGCCAACCTTCTCGGAATGCAGA
GATGTCTGGGACCACACAGAGCATGCTCTCTCCACATCTGTACATTGTTTC
GGCCTCTACATCCCCAAACCACACTCGTAAATTTGGAGTAAAGTTTTGTCC

TGTGAGTTCAAGCATGGCTACATCCCCGCCGCCATTCAACTGGAGACTTTC
TACGACAGGCACAGGAGTGACCGGGGCAAGGACAGGGCCCATTCCCTCAT
AGAACGTGTACTCAGCCTTGTCTGTGCTGATGATTGTCCAGGACGCTCCAT
CATTTATCATTTCTTTATTTTGTTCTTTTGGACATGGAGTGGCCTGAAATTG
GATTATTCTTTCTTGAGAAAGGCACAAGTACATTCTTTCTGTGTCCTTAAG
GTAAAATGCACATTTGTGGAGTTGTGATACAGGGTCGTCTGCATCCAGTA
ATGCTGTCTGCTTATCAACTTTCCTAATTATCAGTCTTGGGAGTGCCATGC
CAGTAACTGAGCACACCAGCTTGACAGTCTGTCCATAATGGATGTAGCCA
TCTCTAACTGTGAATTCCTCTCCTTCCGATTCATCATCATCCAAGAGATGA
ATGTAAAATGCTCCCCACTGCTGTGAACTAGCATGGAAATTCCCTCCTTCT
ACGTGCAGGTATCTGGTGCTAACGGTCTGGGACCGAAGTCGATTAAACAG
CGCCACCTTTGTCCCTGAGGCAATGCACAAGTCAGCATTTTTCAATGATTG
CTTCTTTTTGGAAGGTTTGGAGATGACCTTTATCCGCTTGCTAAGGAACAC
ACCGATGTCATCACTGTTGCCATAGAACATCTTTACAGACAACATGAAAT
GTTTCCTCTTGTCTGAATCAGATATGTACAATGTTTTGGCTGTGCAGTAGT
TCTTCCCTTCCAAGTTCAGCTGCTGCATTTCTTGGTCACTATTTCCTATTCC
AATAAATGCACAGGGTTGAGACTCTTGTTCAGAACAACCATCTCGTTCCAT
TTGTTCTTTTTTTTTCTTCCAACCACTGCCCATAAGATACACACAAGGAGG
AGGGCAAAAAAATCGTTTTTCATTTCCATATGACTTCTGTGCAACTTTTGC
ATGAAGAATGAGTACTGTTTGATCCCCTCGTTCTTTTAAATAATTTCGCAT
AGCTTCCCTAGTAAGTCGTTTAGGTGGAGGCCGCTCACCAAACTTCCTCCT
ATTCACATCAAACGCATATCCACTGGAGAATATCTGGAACTATTCTCCAAC
ACCAACAAGAAAAAAAGTACCTTTGAGCCTACTTATAACAGTTTTAAAAT
ATACCCTCCCATCAACCCTCAGGTAACCCAGGTGAAACGCTACAGCGGAT
GCTCTTGGCCTGGGCCGTCAGAAGCCAGTGATGGGAGAGCAATAGCCATC
AAGAAATCTAGATCCTTTTCCAGCGCTACTGTTACCAAGCAAAACAATCA
CGGGCCCAGAAAGATGGCAAAGAAAAGTGGGATCGTTTTAATCCCAAGA
GAAAGGCGGACGGGGGTGGGTAGGGGTGTGATCCCGAG
>LNC_003549
TACATAATTAACTCATTGTAATAAAAACAAATTTGTTATGGTTTCGAGACT
ACCAATCTTTTAAATTTGAAAATAAATGTTTCAAAGAATTTAAATCAAATG
ACATTGAAAAACTAAATTGCTTTAATAATTTCTGGCAATATGCTTAAATGG
GATAAGTGAAATGCAATTGATCACTCCTATTCATAATGTAAATGCTAGACT
CCTAGCCACGATCCTGAGAAACACACACTGTGCAGCCTACCTGGTCTATTC
TGGATGTTCCGGCTGCAGTGCACCAACTATCTTGGAGTGAATCTGAGCCCC
AAGCTGGCAACTGCAAACTAGATCTCACCTGTAAGGGTAAGAGTGTGTTA
CTATTGTTGTCTGTCCTGAACACATTATCTTCAATCCAAGATTTTGGAGTC
AGTGATGGAGTAGAGCGAGTGAATTTCGGTGGTGCTATAACATTACCCGA
AAACGGTTTCTTCAACGGAGATATCTGATTCATAGTCCCTGGAATTCCCAT
GTTTCCGCTTCTACGGTGATCTCTGCCATGCATTGCTCCCCAGGACATGCT
TGCAGTGCCCCAACCACTGCTCTGATGGTTGCTCCAAGGAGATTGTTTCAG
AAGAGGCTGGTGGTGGTTGTACGAGTTCCTCTGCTGCAGGAAGGCGGCTG
CGGCTGCCTGGTGCTGCTGCTGCAGCTGCGGGCTGACGGGTGACCTCCGG
CTCTGCGGCTGCTGCGGCGCTGCCGGCGGCGGCTGCTGTTGAGGTAAATT

CATGGCGGGCGGCGCGGGCACCGCGGGAGCAGAGAACGGGCCGCCGAAG
CCGCCGCCACCGCCGCCACCGCCGGCCGGCACGCTGAGCCCCGCGCAGCC
GTGCGGCGACACCGGCGAGAAGCTCGGGAAGAAGGCCGGGTTCATGGAC
GACGGCAGCCCGGGGTAGAAGCCGTTCTCCGAGTCCGGGCTGGGCGGCGG
CATGGCGCTCAGAGAGCCGCCCGCGCCGCCGCCGCCGCCCGGGGTGGCAG
CGGAGGAGCCCGGGGGCTGTGGCTGCGGCGGCTGCTGAGGCGGCTGCTGC
TGCTGGGATGGCTGCTGCTGGGGCGCCGGCGGAGGCTGGGGCGGCGGCG
GCG
>LNC_003726
TTTTTTTTTTTCGGAGCTGGGGACCGAACCCAGGGCCTTGCGCTTCCTAGG
CAAGCGCTCTACCACTGAGCTAAATCCCCAACCCCTGACTTTGGGATTTTT
ATGTGACTCCTATGTGTGCAAAATCTAAGTGTCTCTGGATTTATGAGTGTT
ATGCTTTTTCTGATGGGAGGAGAAGGTTGGGAGGATCTTGTAGGAGTTGT
GGAAGTGGAAAGCATAATCAGAATATATTGTTTGGACAAGATCAATTTTC
AATAAAATAAAGTTAAATAAAACAAAACAAAAACCTCTGTGAATTTGAAA
GAAAAAAAGGAAGGGCATATTTATAGGTATGAATGAAGAGAGAAAAGAA
GGGAGAATTATGTAATTATATCATAATCTCAAAAAAAGAAGTAATTAAAC
CCACTTTTATCGTCACAGTTTTAACATATTAAAAAGGGTTTGAATAGGCC
CTGTAATATTTTACAATGATCTTATCAACCTGTTGATTTAGGAAGAGATGT
CCATATGCTACCTTTCTATTGAACCTCTTATATTTCATGGCAGCTCCATTCT
GCAAGCATCGAGTGAGTTTGCTGTGTGAGAATGATCCCAGTCATAATTTTG
CACGCAATCTGCAGTTGTTTTTCTGATGATTTTGATGTTTTGAGATGATTAT
ACTTCTCAAAATTAACCCCACCTTCTTGTTTCAGATTCCTCATTGGCACAG
TATATGCAGGCATGATGTAGCTACAGGGGCAATGGTTGGAGTACCCTGGC
TGTGGTTTGGAGCATTTAATGCATAAAACTACTTTGTCTTCATGTTATTGCT
TAATCTATAATTGAGCAGCATTTCTGTATTGCTTTAATAGTTACCTTGGCA
GTGTGTGTGAGCTTGCACATACACACACTCACACACACACACTGACACAC
ATACTCAACATACCCAAATGTAGTTTCCTTTATTAATCACTCTATGAAAGA
CCACATTACTTGATGTTATCTTATGTACATACACACAGTGGATATTCTCAA
TAGAAGTAGAAACCAAAGTGTAATTAACTCTCTAGAATTTAAGAAGGAAA
TGCCCTGCCCCCTAAAATTACTCTAATTCATAATACATTCTTTTCTTGGACC
CTGATTGTCATCCTCATGTTATTTTTGCCTTATAAAACATTCTTTTCATCAT
ATAACTCTTCTTTAAAACAAAAAAAAAATCATGCTGAGCATTTGGGTCAC
TATCCTCTGAGAAAATTAGACATCACTATTTTTTATCAGAACAGCAGAGA
CTCTTTGCAAAAGACCCCTGGATTAAGCCTCGTGACTTATAACACTCCTTC
ATAGGAGAAGGTTGTTGAAAGGGTCATTGGGACTTCATCTGTCATCCTGG
CTGCTCTACTCTGCATCCTTAGCCACTGTCTGTAATTCTACCATATTTGCAC
ATGGCTTTATAGTGTTGAAGTCTCTAAGGAGTTGGAATATATGAATTGAAT
CCAGTGAATTTATGGGAGAGGACACCACGTATGAAGCTATGAAGAAGTTA
TCTCCATGTTGGGGACAAACCTTTTTAGTAGACTGTCTGCTTTGGGGTTAC
TCACATTTCTGGAAGTAACTCATCATTGATGTCTAAGTGACCCCAACTAGT
TCTCCAACGTGGACTTTGTTGGTATCTATTTTGGTTTGTTACTAGGCTGCAA
AAATGAAGGAATGGATGTTTGTTTACTCCTTTCCCAGAGAAAGAAGTCCT
GTATCCCTTGCTTGCAGGCAGAAGATTGTAAGCTGATGAGTTGACTAGTGT

GAATTATAGAAGTTTCTTTTCAAAATAATCCCTCTGTAAATCATGCTGTAG
GTCACACAATGTATCCAGTGTTCATACCAAGATGATCTTTTATGCCAATTG
CAAGTGTCCTTTGCTACTTCTGCTAACTTTAGTTTTTGCATGTTTTAAAAAA
ATACAAAGATCTAGGGTTTTGTTTGGTTTTGTTTTTAATCTCATTGCTCAAT
AGCTTCTCATGGCCCAACTACTGAAAGTTCATAAATAGGTCAAGTGGGGC
ATCACTGAGACTTAGTCCGTCTTTTCATACGCTAACACAGTACTATAATGT
GAATATAGTTCCATGCGCTTATGAAGTGTAGCTTGTTCCTGGGTGATATTA
GAAAGAACTTGTAATTAGGAAAATACACAGAACAATGTGGTTGAATGCTA
TGAAAACTAATAATCTTCTAACCCTACATAAGAAACAAATACTAAAGTAA
AATAAATTCATTCATATTCATTAAATATAGTTTTAAAAAGATTCTTATATT
TTGCCGGGCTGATTTAATGTTACCAGCAAAATGTCCCTTTTTGCAACTTCA
AAGATCTTTTCAAGTTCATATGTGTTCTGGGATCACAGAGCCACTAAAGG
AAATTGGGCTTTTGCCTAGTAGTTATGGAATAACTTTCCCTTGTTCCAAAA
TATTATGGAACTAAGAGGATATTGTATTGGTTTTCTGCCCAGAAAAATGAT
GCCTCATTCCCCGATTATGTAGTTAGCTGTTCTCCATTCTGTAATCTCCACC
ACTGTATGTGGCTGTCATGCTGTGTTTTGCAACTGAGGGTGACTGTGCCTC
CAGGGCAATATCTGCTTCCTCTGGAGACATTTTGGGTTGTTCTATGTAGCC
TGCTGTTATTGGCTTCTAGTAAAAGAGGCATGGATGTTACTGCGCATTCTA
GTGTTGTAACTAGGCCCCTGCAACAAAGAATTATTGAGCTCCAAATGTCA
ATAGAGCGAAAGCTGAGAAACCCTGTTTTACACTATTTCATATATTTTATG
TTTAGTACCTTGGAAGATTGACACTGTATGAGGCTTAGTCTTGTATTTCCC
AAAGTTTCTCAAAACACAAAGCTTATTCTATATATACTGTATGGTGAATGA
CTCTCAAATGGAAGTTTGCCTTTGATTATTAAATTTTTATGTTTTTTACTCG
ATATCATTAGTGAAGTTTTGAAAAGACTAAACTTTTATATTTGGCACATAA
ATGATTCTCACAATTAAAGAAAGAAAGCATTCTGCATATTTTTTTCAGAAT
GCTTGTTAGTTTACTCTTCATACCCACCAGAATGAATTTTCAAATTATATA
TTCTGTACAGAAATATCAACATAAATGTGCTTTCATGAAGCATGCATCTGC
TATCAGTGAGGCTGACTGAAATTTAGACATATGCTGGGTGGCAGCAGATA
AGTTGGGAATTAGTTGTGATAGGTAATCCACAAAATAGCTAGACCACATG
CCAGTAATCAAGAATTCACTGTAATTACTTTAATCTGTAATCAGCTCAAGG
CAGTGCACAAAAGACTGGCAGAGGAATTCTGCGACCAGAGAGGAAGTTG
GTTAATTATTCCAAGACCATGTCTTTGTTCTTGTATTGATTTTCAATTGTGT
GACCTTTTTATTCATTCATTTATATATTTATTCATTCAATACAATGCGTTAA
TTACTCTAGGAACATATTAATTACAGAAGGAACAATAAACAATAAATATA
TCCCCTGACCTCTCAATACATCCAAGCTCAGAGTGAAAGAGGGGCATGCA
AATTAGCTCATATGCTGCATCTTCATTCTGTTTGTCCCCTGAAAATGGAGA
AGATGGAGGTTCAAAGTTAATAGGCAGTAATTATGAGGGAGTTCTTAGTT
TTTATACTGGAATGAAAATAATTATTGGAAAGCATACAGACTTATGCGGG
GATGCACATCTGTTGTTGCGAAGGCAGACAGCTTAGTTTATTCATGAAACT
AAAGAACTTTAGTTTCAGCTGAAATTTTTGCTTCTTGAAAGAATATTCTGG
TCCCGCCAGGGAGGGCCCAGGAGCAGCAGGACCCCTGCCTGAGACACCG
CCGGAACCTGAAGGAAACAGACTGGATAAACAGTTCTATGCACCCAAATC
CCGTGGGAGGGAGAGCTAAACCTTCAGAGAGGCAGACAAGCCTGGGAAA
CCAGAAGAGACTGCTCTCTGCACACACATCTCGGACGCCAGAGGAAAAG

CCAAAGACCATCTGGAACCC
>LNC_003733
TGGAGACAGAAGATTGTGTCTACAGAAGCACAAGTTGTAACATTTCACAA
CTTCTAAAAGGAATGTCAACAATTACAACGATCATGCATACCATGGTCGA
TAATCACATTTTAGAAGCATTTTCAACCATTTCTAAAGAAATGCTTATAAC
ATTGTTATATATAGAACTACTTTCAATAAACTGCAAAACATTGATCGACTT
TTCCAGTATGAGCTACAGTGTCAACACAAAAGGGAGGCATAAATGTTTAA
TTTATGAAATCAGAATGGAATATTTACTGTAAAGAAAAATTAAAAAGCTT
TCAAATAAAGGCCATTATTGAACCAACATGAAGAGCACAACTTGAACTTG
ACTTCATTCATCTTTAAAGCTGTCCTCTGAGGAGCTTCAGTTCTAAGCAGT
GCACTCAGTACTGTGAATATTCCTTGGACTGAAGTTTGGCAATAATGCGGT
CCAACTGTCTCTTTGCTTCACACTGTGGGAAATTGCTCATGTCATAATATT
GAGGGGCAATTTTCACCAACCATTCTGGCTTGATATCTGTACAGGTTCGGA
TATAATTCTTTGTTGTGAGCACAAACTCATTATAAAGCACCCATTCAGGCT
TGTGATCAAGCACAGTAGAGGGATGCAACTGGACCACCTGGTTATCTTTC
ACAGTTAAGTAATGCCCTGTTCGTTCTAAATGTGCCACCTGCATAAAATAC
CCAGTAACCAATGCTTTTCTTATATTAATATAATAGTCCCTGCTTGTGAAA
TCAGTACTTCGACGAGGCAAATTAAATCTGTCCATAATTCTTGATAGCTGC
TGTCGTACATTGTCTGCAGACATCAGGGACCGGTAGTTAATGAAGTTGTC
ATAACACCACTGAACAGATTCATGATTTTGTTTAAAAGCGTGGTAAACATT
CAGCAGTGTCAGATGATCTCCATCTATGTGGGCAAATCTCATCTTGGCCTC
ATCTGCAGCTTTCTTGGCCTCCGTGGGGCGAACAAAACACTGTGGGACTG
ACAACATGGCAGTAATAGATAGGACCTCATTAGAACAGTTGTAGTCACAA
CTTGCAATAACCATTTTAGCGAGCTGGGGATCTAAAGGAAATTCTGCCAT
CATGGATCCCAATTCAGTCAGATCTCCATCATCATTTAAAGCGGCCAAATA
ATTCAGAAGTTCTAGCGCTCTCATCAGAGTTTCGGGAGCTGGTGGGTCCAT
AAAATCAAAATGTACCAAGTCATCAATACCAAGCTTCTTCAATTGCAATA
CAACTGATCCTAAATTAGAACGCAGAATCTCAGGATAGGTGTTATCCTGC
ATTTCTGTTTTATAAGCTTTCTCTGTGTACAGCCTGAAGCATTTCCCAGGTC
TGGTACGTCCAGCTCGACCAGCCCTTTGCTGAGCTGAGGCTTTACTAATTG
CTGTCACCAAAAGAGACTCAACTCTGATTCTAGGGTTATATACCTTTTGCT
TTGCAAATCCAGGGTCAATAACAAACACCACACCATCTATTGTCAAAGAG
GTCTCTGCAATATTAGTTGACACAACCACCTTTCTTCCAATTGCTCCATTCT
GTTTTTTTGGAGGTGGTGGCTCAAAAATGCGCTGTTGCTGCTGGGGTGGAA
GTGTAGAATATAATGGAATGATTTTAATATCACCAACTTCAGGACCCAAA
TCATCAACTTCACGCTTTATTCTCTTACAGGCCTCATCAATTTCCTCTTGAC
CAGTCAAGAAAAGAAGAAGGTCTCCTTCCTCCTCTTCACACATATGTATTT
GGATCACCGTTCGAATTGCTGCTTCAAGATAATCTCTCTCTGGTTCGGGAG
TATAAAAAATCTCAACAGGATGTGTTCGGCCAGGTATAGTTAAGAGAGGA
CAGTTATCAAAGTAAATCTGGAATTTTCCAGCATCTAGGGTAGCACTCATA
ACTATAACCTTTAAATCTGATCTCTGTCTCACAACTTCCTTTAGAACACCC
ATAAGAATATCCGTAGCTAATGTTCTTTCATGGGCCTCATCAAGAATTATC
ACACCATAACGCTCCAGGAGGGGATCATTCATAGCTTCACGAAGGAGCAT
CCCGTCAGTCATATACTTAAGAATGGTTTTTGCACTACTGCAGTCTTCAAA

TCGAATGGAGTATCCAACTTCCTGGCCTAACATCACGTCCATCTCATCAGC
AACTCTCTGAGCCACACTCATTGCAGCCACTCTCCTGGGCTGTGTACAGGC
AACTCCCCTTTTGGGTCCTGGCAAGGATCGCATATACTCCACACACCACTG
TGGTATCTGTGTTGTTTTACCAGACCCAGTCTCACCAACCAGTACAAATGA
CTGATGTCTAACAAGAATATCTGTAAATCTATCCTTGTATTCCCAAACAGG
GAGCTGAAGACGTTTCTTTAGAATATCATAATATCGAGGAGTATGGGGCA
GGTTGGTAAATGGATTAATACACTGTGGAAGTGATGTGTGTCCTGCATGTC
CGGTGTGTGTTGAATGGGCAGAATGTGTAGAATGTGTTGAATGAGCGGAG
TGGGTTGAATGAGCGGAATGAGAAGCTTTTAAAGGTGGCAATCCAGCACT
GATAAGCATAGCATTGGTGGAAGCTCTCAATTCCTTTTCCTTTTCTTTCTCC
CTCTCCCGTTCTCTGTCTCCTCTATCACGTTCTCGGTCTCGATCTTTAGATC
GGTCCTCTCTATCCCGATCTCGTTCCCGGTCTTTCCCATCGGTCCCCGCACG
CTTCTTGCCGGAGGGGTAATCCTCCCCCAGGTCCAACCGATGCCTCTTGGA
CATCTTCGCACTCCCAGAGCGGACGCTTAGGAAGGAAGGAAGAAGAGAG
ACTCCAGCAGCCGGATCCACACAGAAGAGGCCCGCTGCTCAACTCGCAGG
GACCCCCACCCCTCCCGCCACCACAGACCGCTCCGTGCGGCCCGAACCAG
GAGCTAAAATGGCG
>LNC_003783
GTTTGAAGGGCTGATAGAGAAGGCCTTGAGAAAAACAAGAGCAGGCTTG
TAGGAGAGCAAACTTTGCTTCTGTCTTCAAAGGAGATGAGCTAGAGCCTT
TAGAAGTCTGAGGAAAGCTGTGGGGCACACCACACACTCTCTTTGCTCAG
GGCTTGACCTGCTCCCTAGTGTTAGCTCTTTTTTTTTTTTTTTTTTTTTTTT
TTTTTGGTTCTTTTTTTCGGAGCTGGGGACCGAACCCAGGGCCTTGCGCTT
CCTAGGCAAGTGCTCTACCACTGAGCTAAATCCCCAACCCGGCCTCAGTG
GTTTTAAAGAGGCCTTGAGTCCATTGTTTAGTTCCCTTCTCCATTCCCCCAC
CTTCCTGCCCAGTTGCCAGTCTCACAGGATTTGAGAAGCTTTACTTGAAGC
TGGCGTGGCAAATGCCTGTAATTCTAGAATTTGGAAGGTAGAGGCTAGAG
GGTTAGGAGTTCAAGACCGTGCTCAATGGCATGCATAATGGTTCAAGGCT
GGCCTTGACTTCAGGAAATGCTATCTCCGGCCAGCCCTCTAACAAACGTTA
ATTCACTCTTGCCCTTCTTTGGGGCTTAGACTTGATGGTTCTGAAGGGAAG
GCAGTTTGTGTCAGATACACCAGCTGGGACTGTGCTGCTGGTGTTTGGGAT
CTGTGTCAGGTGTTTCCAGGGTTCCGGCCTCTTATCCAAAAAATCGAAACT
AGGGCCTCACATGGATTTGCACTAGGAGCAAGGTTTGGAGCAAAACAGTA
GTGTAGATTGTGGAAGGATACACTATCAAAGATTAACTGCAGGCTGAGTG
AGTTGGGTACTCAAGGACCCAAGGCTAGGGTCTCAAGGTGTCTAAAGAAA
GGAAGGGTGGGTTACCAAATGAGCAGAGTTTGAGGTCCTCTATTTTTTTTT
TTTTTTTTTTTTTTGGTTCTTTTTTTCGGAGCTGGGGACTGAACCCAGGGCC
TTGCGCTTCCTAGGCAAGCGCTCTACCACTGAGCCAAATCCCCAACCCCG
AGGTCCTCTATTTTTAAAAAGTTTTAAAATGTTTCAAGGCAGGTCTCTAAT
GTCCTTGCTGTCCTGGAACTAGGTTGGTTTTGAACTAACAGAGATGATCCC
ATTTCTGCCTCCCGAGTGCTAATTAAAGGTGTGTGTCACCACGCCTGCCTT
GGTGTTTCCTGTTTATAGGTTTTACATTTTCTTTTTTCTTTTTTTTTTTTCTTT
TTTCGGAGCTGGGGACCGAACCCAGGTTTTACATTTTCTATTGCATATGTC
ACTTCCCAAATTACATCTCATTTAGTCATCTGTGTGCCCAATCATGAGTA

AGCATAAGATGGTTTATTAATTTCCCCTAAAAGTTACTTAGAAGACAGTTT
GCATTACTGCATACATACTCAGAACCTGTTAAGGCCAGGACAGTCCTTCTA
TACCTTCATACCTATAAAACATGGTATACCTTTAAGAGGTATACTTTCTAG
ATTTATTGTCACAGGCAGCCACGTTTGTTCAGCTGTGGGAATGTGCGTAGC
CTGTTGCAGTTGAAAGTTTGAAAGCGGTTCTGATGTGTTAGGAGAGGAGT
AAGTGCATAGTGTATTTGGTATAGAGTCCAATATGAGAAGATAAGCAGAA
GACAAACCAAGTAGGGTCTCAGGTTAAACTCTTAGGCTTTCCTGCCTCAG
GCTGCTGGAGTTGCACTCTTACTTATTCATTCGTTCGTTCATTTGTTCTTGC
ATTCATTCTTTGGCTTGGGACAAGGTCACAGCTGGGTGGTAGTGATGCGTA
TCTTTAATACCAGCGCTCAGGAGGCAAGTGGTTCTCTGTTTGAGGCCAGCC
TGGTCTACAGATGGAATTCTAGAACATCCGGGGCTACATAGAAAAACCTG
TTCTCAAAAAAAACAACCCCCACTAAAACAAAACAAAACAAACCCAAAA
CTAAAGGAGTGGGTATGTGTGTGTGTCTTATCTGGCTAGAATTGAATTTAG
AGATACTCCTGCCCCACTCCTGCCCCCCAAGTGTATGGATTAAGGTGTAAT
TTACTGCATCCACGGGGTGTGTCCTTACAGCCAGACTTTTCTCCTGAGGCC
TGAGGAAGACTGGGAGTATGAATCTCATGACATGGAATGAATTTCCTGCT
TTGTTTTCTTGGCTTTAGTATAGCTAAGCAGACCCACTATTCTGCACCACT
CATTAGTTAGGATGCTAACACAAGCTCAGTGGTTGAAAAGGCATTTTCAC
AGAAGTCATGGTGCTCCCATGGCTTTTATTAGGTCATTCAATAAGCCAGAT
TCTTTTTTTTTTTTTTTTTAATTTAAAATAAGCCAGATTTTTTTTCATCTACT
CTGTTCACTACAGCTCGTTGGCATTTTTGGGCAGCTAGCTTCCTTCAAAAA
CAAAATGTCTGCAGAGCCTTGAGCTGAATATCATACAGAGTATTTGCTCA
AGACAGGGTCTACGTCTCATTAAATTGCTGAGAAAGGGGGTTGGGGATTT
AGCTTAGTGGTCCCCAGCTCCGAAAAAAAAAAAATTGCTGAGAAAGGGAA
GTGTGTTTAGTCCTATAAATTATGTCGAATCTGTGTGTCCTTCGCAAAGAG
GGCTAGTGTCGATCACTGACTAGCCTAGCCTGTAAACATGGCAAGACCTG
GTCCCTGAACACTATTCCCACTTCTGATTTCTACTATCTTCCCAAGGACTCC
TGGTTCTTCAGCTTCCTTTGGGACAGCTCACTTGCCCTCTTTTAATGTTATT
TTTTATTTTTTAAATATGTGTGTGTTTTGATTATATATATCTACATATCACA
TGTGGGCTTGGATCACTGCAGAGGCCAGAAAAGGGTGTCCGATGCCCTGG
AACTAGAGTTAATGACTGATGTGAGCCATTGGGTGCTTGAAATGAAACTT
GAGTCCTCTAGAACAGGCAGAGCTGCTGCTTTTTATTTAATTTTTAGCTTA
AAGCTTTGTTTCTCACATTTTTCTAGCCTCAAATGCTTCTAAACTTATGCAG
TACATACCTGATAACTGCAACGTTGCTCCAAAGAGCCCTTGCCCTCTCTGA
CACAGGAATGCCTTGCTCTAGGTTATTCTTCTGTTAAATTTATTGAGTAGT
TGGGTTCCATGTGTGAGGCACCAGGAAATTGCCCCAGGCATTTCTGCTTTT
TCTGTAAGGTTCTGATCCCCTGGAAGACTAAGCATTCATTTTTGAGACTAA
GTTTCAGGTAGACAAAGCTTGCCTTGAACCTCACTATATATAGGGCTTATC
TTGAATACTCGAGTTCCTTCCACCTTTTCAGTTAGCTTTTGTACACGTTTTT
TACATTTCATTTATGTTTAGGAATGCTCTGCAAGTACACCCTGCCAGACAA
GAGAATCGTATCCCTTTATAGATTGAACTCAGGACCTCTGGAAGAGCAGC
CAGTTCTCTTAACCCCTGAGCCATCTCTCCAGCCCTTGTACACACATTTAA
TATTCTGTTTCATCTGGCCAATTAGTGTTTGCCTTTGTCTTATCTGTTCTAC
CAGCTTCATCCTGAAATAATTTTTTTGACAGCTTGACAAATTCTACCCATA

CCTTACTACCACTGTTGGCTCAGCAGGACACACAGGATAAGCTGACAAGT
GGAACGGCTTTTTCCAGATTCCTCTAGAAACCGAGGAGTCACTGAGAGCA
TGGTGGCATGTAATGAGCTTGGAAGTCCCAGACTGCCCTGACTCACAGCC
AGGTTCTGTTGAAGTCTCCCAGTGCAGAGCAAGCAGACTGGGCAGCTGAA
GGATTATAATCAGTATTGTCAAGACTTGGGAGCCCTACAGAGAAAGCCCA
ACAGAATGGAGAATGGGCTATTTTAATCTCTACATTGGAATTAAAATAGC
ATTTATGGCCCCAAATCCACAATTAATCCAATGATTGGGCTAATTAATATG
AACCTGAGAGTAAATATAACAAAAGGACTGGAAAACCTGCAATCAGTGT
ACTATCTTTCAGAATGCTTTTACATACAATTTACATAGTACTGGACCTCGA
GTTCTAAGTAAAAAAAAATTTTTTTAAAGATTTATTTTTATGAGTCCACTG
TAGCTGTCTTCAGACACACCAGAAAAGGGTATCAGACCCCATTACAGATG
ATTATGAGCCACAATGTGGTTGCTGGGATTTGAACTCAGGACCTCTGGAA
GAGCAGTCAGTGCTCTTAACCAGTAAGTCATCTCTTCAGCCCATAAGTAA
AAATTTTGAGAGGAACTAATACAGCAGAGGCAAGATAAAGAATTAATAT
AGGCTGGATTAACCAGTAAGTTTCAGAGAAATTACCATATATGACTTCCCT
AAAGGTCAGTTTGGTTTTGTAAAGATTATAATATTGATTCCTTGTTATTTG
CCACAAGCTACTCTCAGGGTTAGTCCAGCTTAAACTGATGCTATCAGAACT
TTATGTGCAGGAGAAATTCTAGAATTTTTTTTTTTTTTGGGTTCTTTTTTTT
TGGAGCTGGGTACCGAACCCAGGGCCTTGCGTTTTCTAGGCAAGCGCTCT
ACCACTGAGCTAAATTCCCCAACCCCGTAAACTGTCTTTTAGTTCATTGAG
GAAATAATCACAGTACCAGTTTTATGGTTCACACGCGTGGCATATGCAAC
TTTTAATAAACCAAAGAAAAAGTCCCAAATATACAAGTGAAAAAAATCCA
AACAGTTGACACAGGCCTAACTGATAGTTACTTTATGTACAGCTGTATGTA
TAAGTTCAAAAAAGCTATCCTATTTGTATGTACAAAAGTTTATACACAGTT
CTGTACATAAGGGTCTATACATTTTATTTTCTCAGAACCCTTAGGTGTCAC
CTCTAGAAGACACCAACACTTCATTCACATATTTTATAAAAGAAAGACTTC
CAGGACTGACAATCTTGTGTCCCTTGTATTTGAACCATGTAGGCTCCATTT
GATTATTTAACAGCCTTTTGTCATAATGTAGATCATGGTAGGTATGTGGAT
CCCCTTAACATTCTCACACAGCGTGCTCCAATGTAAAAAGCCCATTACAA
AGATGGGGAATGAAAGTCATGGCCAGGATGCCACCGCAGGTCCAAGTAT
GGGTGGGCCAGCCTGCCCTGTTCACTGTCTATATTCCAGTTCATCTACACT
GATGACTTTGGTGGGCATGGAGGGCAGAGGCTGCTGTAGCACATCTGAGC
CAAACCACTTGGCAAGACCCACGGGGGAGCTGCTCCTTTGGCTGGTGCGA
TGCTCCAGCTGGGAGTGCATATGGGGCAGGCCTGACCGGCTGGGTACATT
CGGAGGAGTCTGAACGTTGATAGCTGCTGCCTGGGAACTAGAGCCTGGAG
GATGCAGAACTGAGCGCTGTAGCTGCTGCTGCATCACTGCCAAATGCAGA
GGGGTCCCAGGACGAGGGTTTAGGAGAGGGTGACTAGCAGCAGAGACCA
AAGGGTACAAGGGTTGACCTAGGAGTGGGCCAGATAGTCCCTGTAAAGG
AGCTATGTCTATCCCAGGAGGAAGCACACCAGCTTGGAGCAAACCTGGAA
GATGCTGTGGATGCACCCCTTGGGCTATCAACCTCTGGACTAATCCTGGGT
GAAGCTGATGTGCAGGTCGAACAATAGGAACATGAGGGACGAGGGGAAC
TTGGTG
>LNC_003882
TTCTCCTCTGTGGTCAGGCTGAACACCACCTTTCCCAGGACCACGGTGCCG

CTGTTCACACCAGGCTGCAGCGTACTCAGTGGCTTGAGCTGCAGGGTCAC
CTTCTGCCCGGTGGCTGACTGGTAGCGCCCGTCGCCACAAGGGCCCAGAT
GGGCCGGGCGCAAGTTTCCCAGCATGCTCTGAAGCTTCTTGGGTTTTGTCT
TCCCCTTGCTCGCCAGGAGGTTGGTCAGCCTACTGAGGAATTCCAGGAGC
TGCTGTTCTCGTTGTTGGGGCTCTGGCCATGCAGGGTCCAGCGTGGCAGCC
TGAGAGAAGCCCTCTAGGGCCTCCCCATAACTCTCTTCATATTTATGTAAC
GTCGCCCTGTTGAGATGAAGGTCAGGGTTGCTGGATGCTTTCCTGTCGACC
TTCTCTGCTTGAGCATAGGCACTGAGGGCTTGCTGGGAGATCTTAGGGTTC
TGGCCAGTATTGAAATAAAGAGAAAGATATGCATTCCCCAGGATATACCA
GGAGCGGCCATCAAGGACGTCCATCTGCACAGCCAACTTGGCCTGTCGGA
CACTATCCATGACGTGGCGAGAATGTTCATCTCCGGAGTCGGTCTGCAACT
GGCGGAGCACCATGGACAAGTTCTGCAGAGAGACCTTGTTCTTGCAGTGG
GTGAGGGCTCCTGAGAAGCAGGTGTGGGCAGCCGCAACATCTCCTTTCTT
CCAGTACACCTCACCCAACTGGTTCCAGGCTTCCACCAGTTCAGGCTCCAG
CTTCACGGCCTTTGAGAGCAGCACCTCGGCCTCCGGGCTGTAATCAGGAG
TCACATTCAGTGCCTTCCCCTTGAGCATTAGAGCCTGTGCCTCAACCTGGA
CAGAACCAAGTACTTCCTCCATCTGCCGCAGGGTCTTCTCCATCTCTTCCT
GAACATCCTGCTGCTTCTGTCCTGCATCTTCCACACTGTGTGTCTCAAAGT
AACTGTCTCGAAAACAGTAGAGCTGATCCACCAGTTCCTGCAATTTCTGCA
AGACCTGCTTCGCTTCCTCCTCTTCATCAGCCATCATTCCAGCCGCTCTCA
GCTTCTCCTCCCCC
>LNC_004034
GGAGGAGGAAGAGGAGGAGGAAGAGGAGGAGGAAGAGGAGGAGCTTTC
CCCTGCTTCCAGTTAAGCTCTCGATGGTCTCAGGGACATGCTGGTTACCAC
GAGGGCCAGCCTTGTACAGGACACCACTGTCAGAGCTGTGTGGTGTTCAA
GGCACTCCTACCACACAGTGTGGTGCGTTACTAACGGCTGACAACTGCTG
GCCTTGGGATGGAAAGGGGTCTCAAGAACACCAAGTGACATTTCCAGAAC
CTCCGAGAAGCTTGCCTGCATAGACTGTCGTATTTAGGGGAACACCACAT
GGCCTTTCCGTCCTGCACTATTACTAGCTGAGACAGATTTATTCCCTTTGT
ACCAGGAAACTTTAGCCATGGATGTGAGTCACGGAGGCTTATTCCTGAAC
TGAATATCACCTTCTGCAATCAAACCAGAACGGCATGTTTAATGAGAAT
GAACACCGTTCTCATTCTCTCATTCTTTAACGTTACACAGAATTAGAGAT
TGCTGTGAATTTTTTTAATTTGAAATCCGGATTAAAGTGAAAGCAGTGGG
AGTGAAGCTTTACAAATTTACATTACTATGTCATTGACATGGCTTTTACAC
TGATTGGATACAGAAAGAAAGAGACTAACATTAGAATTAAGGCAGTGAC
AACATGTGCAAACGCAGCACACCCCCTGACAGGCCTCAGTGGAAAAGCAC
TCTGGTCAGTTGGTGTCTGACGTGGCTGCATGCAGGTCTCATTGCTCAGAA
AGATAGGCCCTGAAGAGCTTCATTCCTTAAAGGGAAAAAAATCCCCGTCA
CTGGCCAACGATGGCCAGGAGCAGCTTCCTGTAATCTCCACTGGTGTCACT
TGCAATCATCGTGCTTAGAGTCTTCTGATACATCTGGGTGAACATCTGTTT
TATCTGAACGAGGTCAATCTCACTCCGAGTGACCACAATCCTGACCAGGG
TGGAGTCATCTGTTCCAGCGCCTTTCATGGAATAGTAGAGTCGCTCGGCGA
AGAAGGCAGGGCGGTTGAGGGCGCACTGCAATATGGTCTTCAAACCGCTT
TCAACGTATCCGGAAAATTCCCGGCTCACACTGCTTAGCAAATCTCGATTA

GCCATCCTGGAATAAGCCTCCATGGTGGCTCTCAGCTGAGGGAAGCTCCT
TGTGGCGAGGATCATGTTGAAACAGGATTCGTCTGTCCCTAGCCTCCCCTC
CCCAGCCTGATAGAGGCGCTGGGCATCTTCCTGGGCCATCTGGTGGTTTAT
ACTTGGGCTCTCGTCACGGTTTCCCTGGCACATGGACACGAGGAGCCGTTC
GAAATGTCCTGAGGTATCCGATCTAATGTCCCTCTCGAGCTCGCGTCCAAA
TTCTAATTGATAGCATCTAACAATGTCTCGGATTTCTTGATTTGTTCTTGTG
CACAAAATCTCTATCAACACACGCTCTTGAGTTCCTGCTCCCTGTATCGCC
TTCCGCAAACTCCAGGCATCATAGTATGTAGGAGGCATGAACAGGGCAAG
GATTAATTCTTCCATGTTTCCACTCAACTCTGACTTGAGATCTTTAATTAAA
TCCTTGCCATACATGGTCTTAAAAGCTGCTTTAATTTGTTGCCTCTGGTCAT
TGGAACGGTTAGACACGACATCCACAATCGCTTGCTCATCAGTCCCAAAC
CCCTTCATTGCTTTGCGGAGAATCTCTGCATCTCTCATAGCATCGAAGTTG
GAGGCTGGGCGGATTGTTCCTTGGGTGCCCTGAGTCATCGCAGCAGGCTG
GCCAGGGTAGGGAGACTGTCCTCCGGGGTACTGAGACGGCATCTGCCCTC
CAGGAAAGCCACCTGGGACTGGAACCTGGGCTGGTCCACCGCCGTAAGAC
TGTGCAGGTGGCTGTGGATAGCCAGAAAAGCCTGCCCCACCAGGTGGGGC
CCCAAAGCCTTGACCTCCAGGATAGGATGGAGGACCCCCTGGATTTAGGG
TTCCAGGATAGCCCCCAGGGGCTGGATAACCTCCAGGTGAGGGGTAGCCT
CCAGCTCCTGGGTAGCCACCACTTGGAGCTGGTGGGTAAGCACCTCCTCC
CATTGGTGGAAAGCCACTAGGGTACGGATACTGACCCGAAGTGGGGAAA
GAGGACTCCTGACCTGCAGGAGGATATCCAGGGAAAGGTGGGTAGCCTGT
CGGGGGGTAGCCTGGGTATGACATTCTTGCGTCTTTTCACTTGTCCCCCGG
TTTAGCCATTCCCCTCTGCATCTTGGTTACCTGGACGTCTGGTAAGGGTCA
AGGACTAGGAAGGAAAATCTAGACTGTTACTTCCGAGAATAGTTTCCAAG
AATTTTAAGCCGTACCTTACGTATCAGGCTTGTGAATTCTGTCCTAGGTGC
CATTAAGCACAGCAAAAATTCCTCAAGATCGCCTAAATTTGCTGTGTCACC
AAAAAGTCTAGTCACTAGCCAAACCCAGCTTTCGGAGCGATTAGTCGGCC
CACCAAACCCGCCCTCTGGTCTCAAGCCCTCCACCTGCGACCCACCTGACC
GAAAGCGGGGTGCCACTGCTCCCTGGCATCCCACTCTAAGCCTAGCAGAG
TGATTTACCAACAGGCCTCCCCAGTCCTTCCGCACGCGGCGAAAGCATGC
CCAACCCACCCCAGCACCCTATACGCGTCCGAGTCTCAG
>LNC_005121
CATTTATTCCTCAATTTTTGTGCAAGAGTGTAAAGATGCATTTCAAAGAAC
CTGATGACTAGAGAGCGTCAGCCATGGCCTTCAAGCTGGCCTGGCTTACA
TTTAGGTTAGGAAAAGGGATGAGGGGAGATAACTTGCCTAGTTCACATGA
ATGTTTCTGGATTAACAGTTGTTTACAAATTCCAAACTACATTTTACTAGA
GTCAAAGGAAGACTTCAAAAAGCCACATACAGTACAATTTAAAACCAATG
GACATCCTGCAAATGTTAGTGCAAAGTAATAACGTAGACTCTCCGATACA
AGATTGTGCTCATGTTTCCCAGGTTTGGTCAGTTACTCGTTATAGTTACAA
TCAATTAGCTCATCAACAGCAACACCTTGTGTCCTATGTCCTATGTACCAA
GAGACTGTCGTAAGGTTCTAGTGAAGTGCTGCTCACTTTCACTAACAAAG
CAAATTTCTTTAGTTTAACAGTCATGTTCCTGGAATGGTGCACATGTAAGT
TTCATGAAAATGCCTTTAAGCTTACGCCTCTATCTTGTTTAATAAAATTAC
TCTCACATCTCTTTTAGTATGTAAACTGGAATTGTTTCATGATTTGAAATG

CAACAAATATAAATGAAAACATTTCAGGGATGAGGAAAAATCCACCACTA
ATCTTAGCTAGACCAAGTTTTTAAATCAAAAAGCCTATTCACACAAAATTA
TATGTAATGACTGTAGTAATCAGTTTATTACACAGATTAATCATTCTTGAA
CGTACAAGCTCCAGAGGAGCAACTGGGTCTTTAAATATACACAAGTGTTT
CCATCAAATGAATGTTCACCCTTACATCGAAATAGGCCTAAAGCAGTTAA
AAACATAAGAAAAATAAGAGCTATTAGCTATGTATTAACTGAGAAACCAC
ATACAAACCAAAGATTATGGAAAGGGGCTAAAAGGAGGAAGGCTGAAAG
GGCAGGGAGATATGAAAGTGTTGGGAACAAAGTCCATCACACTTTATGTA
CTAACAGCTCCAGTCCATTTAAATGTTGATCAGTTAAACTTAGACCTTAAA
ATACAGCATGTAGTTTACTCTGCTTGGTCATAACTTTCTCCTAAGCTGTAG
GTCTTTCAGGATGAAAGGAATACAGAAACAGCTTCACGCTCTTCACCATG
CTGTTCACGACTGTTAGGGGATTAGATGGAGTCCACTTAAGGACCATCTCC
TATGGGTCACTGGAGCTTTTTCCTCCCATTCAAGCACACGCTGTGTACCCA
CTACCCATCACATCTTGCCATTATCCAAAGTTAAGTCAATAAGGAAAGCTT
TTAGTCTCACACCTAGGTTACGCCCGGAGAACTGGCTTCTCACACACACAC
TTCTCTGCTAGGAGGCAATGTTGGTACAGAAGTACAGTATTTCTAGTTTGT
TGTTCCAAGTATGTACAACACGCAGCACTGCTGTGTCAGGTAAACATGTG
CACAGGGGAAGATGAGGCGTGGGCTCATAGAAAGCAGTCAAATGGAAAT
CAAAAGGACTTTCTCTCTTTCAGAGTTCCCCTAGCTTTATTTGTAGAGGTT
ATCCAATAATTTCTTTAATTACATCGCATACTTCTGAGTCCATTCCCGAGC
TATTCTGTTGTACTTTTCTCTATCTGTTTTGTAGATCCGAGCAATCTCAGGC
ACTAAAGGATCATCTGGATTGGGATCACACAACAGAGAACAGATGGACA
AAAGTACTTTTGAAATAGTTAGTGCTGGAGACCACTGTGACCGTAGAATA
TCAAGACAAATGCTGCCATTACTGTTAATATTTGGATGATAAATTCTTGTT
GTAAATGCAACCTTAGGCGGTTTGAAGGGGTAATCTGTTGGGAAATGAAT
AGTCAAGAAAAATACTCCACCCTGATAGGGGCTGTCATTTGGCCCCATTA
TTGTAGCCTGCCAATGAAACACTGAAAGAAAGAAAAAGTCACCATATTAA
TTAACAATATTTGCATTACACCTACACCGTAGAACCTCTCATTCCCAGTAC
CGAATCAAAGCTCATTTATAAATAGTTTATTTATAAATAGTATGATAACAC
CTAGTTTCTATGGGCAGGATGAAGATTAAACCATTAAAAATAAGAGACAG
ACAAGTCAGCAGGGTGTGGCAGTGCCTACCTTTAATCTGAAAGCCGCTGG
AAGCAGAGGCAGGTAGATCTCTGAATTAGAGTCCAGCCAGAGTTTGAGTT
CTAGTTACAGAGAAAGCCTATCTCTAAAGCCCCCCCAAAC
>LNC_005300
TGTGTGTCGGCCCGAGACCTCGGTGTCAGCCCGAGATCTCGTTAATAAAA
CTACCTCTTGCTGTTACATCAAGATCAGCTTCTCGAGTTTCCTGGGGCACG
CTGCCATCCCGAGACTGGAGAGAGAGGCTCCCCGAATCGGGGGCAGCTCT
TTCAATACGCTTTACAATACCTTGGGAAACAAAGCCACAAGCTAAAATTT
ACAGAAGACATTTCTCCCCTTTCCAAAAGCCTCTGTTCTTTCCACCAAGGT
CAATGGAGTCCCAGTTCCCAGGTCCAGGCCTAAGGGCCTTGAGGGCTGGA
CCATAACTCTGTATTCATACCAAACTCAAGTCTCAGGAGGCGGCAGAGGT
GCCGGTAGCAGAAGGGCCTGGGGCTGGGTGAATGGATCCATTTGGAGTCT
ACGGCTTCCACTCTCTTGAGCTGTATGTGGCCGATCCAAGGCAGAATGCC
AACTACATTCACCCCGTGGGAGTAGAGAGCATCATCG

>LNC_005382
CTGGGCCTCCCTTCCCTGACGTGGGACCTATCAGGCTGGCTGCTGGCCATG
TCCTGTGGCCAAAGGATGTTGCTCCTCTGGAGGTGGCCTTCTTTATCAGCT
CTTCCTCGCTGGCCCTGGAGGGCTGGCATCCTTCACACGTTCATTAGCTTG
GTAATAGCCGGCGATCACATAGCTGTTGTCTTTGCACCATGAGTCAATTAG
GGTGAGCGCCACCTCCAGCATGGGCGTCAGAGCCAGCGTGCCGTGGAAGA
GCGGGATGCAGTCCACGAAGAGAGTGTGGTTGCCCGCGCCGGGAGGATGT
TCCTTGCGCGGCCTCTGCCTCTCTGCCACCAGAAGCCCGTTGACGGCGCAG
TGCGGGTACTTGGCGCCGTGTAGCACCATCTTGCAGTAGGCCTGGGTGGT
CAGCTTCACGCCAGGCATGCTGAGCCTAGCTGGTCGCGTCGCGACCAACG
CACCACAGTACGAAACACGACCCCACTGCACGCTAAGATGAGCTCCCACC
ACCAGCTACAGCCTCCCGCCTCAGTCCGCCCGCCGGAAAGCGTAGTCGCT
>LNC_005494
CAGATAGATACACAAAAGAGTCTTCATGCTCCAAGTTCTCCAGGAATATC
TGAAGAAGCTTCTTTTGTTCCTCCAGGGCTCTCCCTTCTCTCTGCTCTACCC
AGCGGGAAAGGGTGCGGAGGGCAGCAGCCCGGGTTGGAACCTGGGGATC
ACAGGCAGATAAGAGAACCTCCCGGAATTGTTCTGTGGTTATATTTCCCG
GTGGCTGGCTTGTGGCAGTGCAGGGCTCACTGGAACCTTGGCCTGTTCTG
>LNC_005519
GCTAAACTAACATAAAAAGGGCTGGAGTCGGGGAAAGGGGGGGGGATCC
CCAAAAGAACTGTCAAAGGGCTGTTTCTGTACAGTGAGGGCTTGGTAAGA
AAAAGCCAACTGTTTGTAAAAAGAAATGAGGTTGGTCTGCCTCCGGGCAC
AAGGGGGAGGGGATGCCGCAGCAGTGACCACGTCGGTGGAGGGGAGGTG
TGGACGGTCGGGTCGGGTCAGGCTGGCTGGGTGACGTAGCTGCTCTTCTCT
CCCAAGAACCATGCAAAGAAGTGGGCATGGGGCATGCGTTAAAACCACA
GGGTGGGGAAGCCACAGGTTGCAAGTCTCAGTAGGGGACAGGGACAGCA
TAAAAGGGGGTAAGGAACGACAGCAGGACCTTGGAGGACCAACTCTCCT
ACAGGGGTATACAGGGAGGGAAGGTCAGGGCCCTTCCAGAGTCACAGAA
GCAGAGAAGGGGAGGTGTGAGCTACAATACGGAGAACCTCTTTCTTCCTC
TCCCCGCCCCTCCCCCCCATCTTGATCCCCACCCACGAGGAAGCTGGAGAC
ATCTGGAAATAATACAGAGTAGCAAGTGGGCAGACTGACTGACCAGGAG
AATATTACCATACAGAGACTGGTAAGAAAAAAGCAATTAAGTTTCCAAAG
GTCTTTTTTTCCCTGTTGTTGTTTCCTTTAAAAACCTGAACTACAAGCACTA
ACCCGACAGCTGACTGGTTCAGACAGTACTACACGCCAGGGGCTGGGGGG
GGGTTGTGTGTGGGGGAAGGTAGGGAGAGCGGGCAAGTGGGGGCAGGTG
AGGAGGGAGGGCCGGTGAAAGACTTCATAAATAAGAGCGGGTCCCAGAC
CCGCAATTTGTCAACGTTTCTTAAATAGGTGCGTTATTTAAATCTTATGTA
CAACAAGAACCACTTTGCATAGCGATGGTGAGGACACAGGACGATGGGG
TGACGTGACTGGGTATTCCTGCCCCAATGGTAGGGGTGAGATCTCCGCTC
GGTGCACCTCCAGGAAGAAGACTCCTCCTTCTGGCCCGAGTCAACAGCAT
CAGGCTGGATGAATGCTCCCTCCCCACTCCCCACGGTATAACACAACAAA
GGCAAAGAAACTCCACACAGGGATTCCCGCCCACCCCTCCCTGCCCTGCC
TGCCCGCTGTTCCTCCTCTTGTTGCCACCAAGGCCGCTTTTGATCAAGGTC
AAAGGTTCCAATATGAAGCTCTAAGAATTAGGCGGGGGGTCTGGGCGTGC

TGAGGATTTCCCCACCCTTCCTCCCTCGGCCCCCCTAAGACTCCCCCATCT
ATCCCGCAGCCAGGTGCTGGGACCACCCCGGGACCCACTGCTCTACGGCT
ATTAGTCAAGAGTCATTTTTTTTTGTTTGTTTTGTTTCTTTTTTGTTTTTGTT
TTGTGCGACGCCATCCTGTGCACACAGGGGGCTTCACCAGGCTTCTGTGTA
GCGAACATCAACCTCCTTTAGATTAGGAAGCTTGGCCTTCAGATCCTCATA
GGTGTCAGCCGAGAGCTTGGTGCTGTTCATATTTAAACTGCAAAGACTCTT
CATGGAGCTGAGGGCCAGCAAGCCCGCGTCGGTGACTGGGGTCTCACACA
GGTTCAGCACCTGGAGCATAGTGAGGTGTTCCGACAGGAGCCGCAGGCCG
GCGTCTCCAAACTGCGTGGACCACAGGTTGAGCTGCTTGAGAGAAGGCAG
TTTGATGAGGTGCTCGGCGCAGGCACTGGTGACGTTGGTGAAGGCCAAGC
TGAGGTTCTCCAGGTTTCCAAAGGAGCCGGAGCTCAGTAAGCGGGCCAGG
TCCGCGTCCGTGGACTTGGAGGGCAGTGTTAGCCTGGTGGGTCCGCCTTTC
CTTTGCAGCTCCTTCAACTCCCGCTGCCGGTCGCAAAGCTGTTCATACATG
CGCTTGCCCACGTCTGTGCTCTCCAGCGTGGAGATGATCTGTAGCTGGGTG
TCGTTGTTGTCGATGATGTTGTACTCCAGCATCAAACACAGTATCTCTGCC
ATGGTCTGGTTCCCTCTCAGTGCCAGGAGCATGCAGGGGCCCAGTTTGTTT
TCAGCCAGTGAGAGCAGAAACTTTTTGGTCTTGTAACAGGAGCCCATGAG
GATATGCACCATGCTGGCAAACAGTTCCCCGTCATCATCACAGGCCACCC
CTCCAGGACTGTAGAGCTGGAACCAGCCATCCTTGCCTGCCCGCACGCTC
AGCAGACACGAGTGGACTTCCTGTCTTGGATCTTCTGCAAACCTCTGAATC
ACGTACTTCAGCTCCTTTGTGAACTTCTCGTGCGCGAGAGCTTCTTTGGGG
AATGGAATGGGTTGAAGCAAGCTGGCCAGAGACGGTCTCCAGTCATCATA
GTCGGACAGCAGCTTGAGGATCTCCACATGACAGCCGAGCGTGCGGCCTG
CCTGGGGGCCCAGCTCGATGCTCATGGTGCCACACGCCGGGCTGACCATG
AGACAGTCAATAAGGTTGGGCTCGCTGTCATTGCCCGTGCTGGCTGTCAG
CGCCGGCTTGGCAGTGCTGGTGCGCTCCACCTCTACGTGGACCTCGCTGGA
GGCCACGACCACCGACTTGAGCCGTGGCTCAGACAATGTGGCTTCCTGAG
ACACCAGGTCCGGGCTGGGGTGCAGCAGGCGCAGAGGTAAGGTGGGCTT
CCGGTCGCACGGCTGCTGGCAGCCGTTCCGGATCTCTTTCAGGCTGGGGG
AGTTGTCGCGGCTGTTGATGAACTCTTCATAGCAGGAATATATGGCAGCC
AGGCACACAGCCACGAGGTTGGGCAGGACCCGGGGGTGTGGGCAGTGCC
CTGTGGGGCCATGCATGCTTTGAATGAACTTCTTGACCACCTCCATGCCTG
CGTTGAGCTCATTCAGGGCGAGGATGTACTCCTGGGTGAAGAGCCTCAGC
CTTGGGCATTTCCCGAAGTCCATATTATGCTTAAGGATCCTCTGAACCACT
GGCGTGAACACCTCGATGACCACCATAGACCGGGGCCGCTCTCTGCAGTG
CTTGCAGAAGAACTCGCAGAGGTCTGGTGGTGGGTGGTTGTTCTCCAGCA
GAGGCGCAATTGCCACAATGATGTTCTCGTGCTCCTGAGTGGTCAGGTTTG
TGTTCTCCGAGAGCAGTTTTGAGACGATTTCCAGTGGAGCCTGGTTGATGG
AGTCATCCTGCAGCGGGGACGTGAGAGCCATGTCGACCAGTGTCCGTATC
TCCTTCAAGACAACCTCCCAGCGGCTCGGGTTACTCAGGACTTTCTTGTAT
TTGTAAATCTTTTTCTTCCACTGCAGAGAATGGAACCACTGATCTCGCAGG
TAGCTGTTGGCAGCCTGCAGCAGAACGGTCCCTCCAGGGATTGTGAGCTG
TAAACAGTACTTCGGGGCGTTCTCCCAGGACAGTGGCTGAACGTCTTCAA
TGGCGCTGTAGGAGACCGAGTTTTCCATGTACCCAGACGGCGTGGCGGAT

GCCAAGCTGTTGTCGGCCAGCGTTAGGTGGTGCGGCTCCCAACGCCTCAG
GAATTTGGAGGTGAGGATCTTGCTGAGAAAGGTCCGAGGGTGCCGGATGA
CACAGACTTGAATGTCGCCCTCCTGCAGCAGCTTGTACCTCATCCCGTTGC
AAAGCAGCAGGGCCCGGCGGCAAGGCACGGCGCCCATCACCTTGGTGCCC
TCGGGGGCCGACACGTCGCTCCCTAGCAGCGGCTTGGTCTCCTCGATCTGC
CGGGGGTCGCCGCCGCCCGAGCTGCTCGTCACATCCATGGCCGCGCCGGG
GCGGGGGCCGCGCGCGCGATCGCGGCTGGCGTGGGGCCGGGCCGGCGTG
GCCCCCGCGGAGGGCCCGCACCCCCCCACCGCCGGGAAGGTGGGGGGGC
GTGGGCTGGGGGCGCCCCGCGGGGACGGACGGGAGCGCGCGGGGGCGGG
CGGGTGCGCGCGGGGCGCTTCCTGGGCTGCTTCCTTCTATGCTGGGGCGCA
TGCCGGCGCGAACGGGGTTAGCGACTGCGGCGAGGGCGGCGGGCGATCG
CGGGGCGCAGAGAGGATCGGGTCCTCGGGGGGGCGGGGGCGGCGCCGGG
GACGGCGGGCGCGGGCGTGTCTCGGCCTCGCGCGGGCGCGCTGTGCGGTG
CGTGTGTCCGCGTCCGTCCGCGAGTGTGTGCTGCGGGCGGGGGCCGCGCC
GGCTGCCGGGGAGATTGCGGGAGGCTGGCGCGCGCGATTGGTCCCCG
>LNC_005537
CTTGACGGGGGATGGGCAGACGGGGGATGGGCAAACGGAGGAGCTGGGG
TCAGCTGGGGAGCTGGGGTCAGCTGGAGAGCTGGGGTCAGCTGGGAAACT
GGGACAAGAATCCAATCCAGGTAGCCGTAGCCTCCGGCGCGCGGGCGGC
GCGGGCTGGTCGCTCCCGGGTTCGGCGTGGGGCCGGACGCGCTTGCGGCT
GCTGCTCCGGGTGAACTCAGGAGCCACGAGCCCACCAGGGCTCGGGGTGA
GACAGGTCGACTTGGCTCGCGGAATCGCAAGGCCCGGACGACGGCACGC
ATAGAAATCGGTAACACGACTCTGCGCCATGGCGCCAAAAAGC
>LNC_005561
CCGCCCTCATGTTTTTCCTGAAAATGTTCCCTTCTTGCTGACCCTGGAGTCT
TGCCCTGCAATTACAGCCTTTTTCATGTTATCTTCCTCCCAAGGCGACAGC
TCCCATCCTGGCGGAGGAGACAAAGCAAGGGGGCCTTGACTTTGCTTTCT
GGCCTTTGTTGCAGAACGCAGGATTTCCTTATAGTTTGTCTGGGCTTCCCA
CCGAGCGTGTTTACCAGTCTCCAGTGCCAGATTCATGACCCTGAAGAATA
GAGACCAAGTTCCTATGTGGCTGAAAACACGGCAGGTCCAGGGCAGGTTG
GTGCTGTGGGCCAATCCTGTGTCCCATTAGACAGCTGTGTGTTCTGTTGGG
AAAACAGGCCCAAATCAAAGCACTCACTGGGCTCCTTTCGTCCAGAAGGA
GACCAGAACAGGGCAGTGTCTCAGAGAGGAAAGAACTGTGATGCTGTGC
AAAATCCCCACCTTGTCTGTGTTTTAATAGCCATGTCTGATCATTTATTTCT
TCG
>LNC_005600
CGGCATTAAGTGCATGGAGGCGTCGGTAAATCCTTCTGTTGTGTTAGTTGG
GATCTCCACCAAGGTCAAAATGAATGCTTGTTCATCTTCTCCATCTTGCTG
AACATTAGCAAACAGATTCACTGTATCATCTTGAGGACTGATGTTTTCGTG
TTGGGCTTCTGGAATATTAGATGGTACACTCTCTGGCACATGAAGCATGG
GCACAACGCTGCCTTCAGGGCTTTCTCTGAGGGCTTCTTCAGCAACACTGT
CACCAAGACTCTGACCTGACTCAAGTGTGTGCTGCCCTATCTCAGACGAA
GCAACTGCAGCGCCCAACGTTTGAAGCCCTTGCAAGTCGTCCTCGTGCTCT
TCAGTTCTTGCAGGTAGGATTGCTTCTTGAGAAACCCTGGCCCCTTCGACA

CCATGAACGCACAGCAGCTGCTCCTGCTCCTTGGTCTCTGCTGCTGGAACT
GGCTCAAGGCTTTTGTCGTCTAATTCTACAGCTGTGGAATCATTTTCCTCC
GTTTCTCTTTGAATTTCCACTTCTGTCCCTTTAGTCATGCAAAGACTGGCAA
CTTCCTGATGACCATCAGGCTCACTGATCTCTAAAATTCTCTCAGAACCTG
CTTTTGGTGTAACAAGCATACTCTCCATTCTCAAGGGGGTACTCATTGTAC
AAGCTGTATCCCTGGTTGACAGAGGCTCCTCCATGGCTTCTCCTAGCACGG
CAGCTTCTCTGATGCTTTGTTCCTCCAGTACAATCTTGTTGTCTTCAAATGA
CACATGCGACATACTAATGACAGGTGAGCACACCTCCTGATAGTCATGAA
CAGTTTTCTGATTTACATTATCTGGGATAAAAGGAACATGATTCTTATCCT
TTGAATGATCATCAGGAAATACACATACTCCTACATCACCCTGTCCAGGA
ATGATCTCTGACTGCAGTACTATGTCTCCCATTGTAAGTAGAGTTATGGCG
GCTTCAGTGCTCCCATCACCGGGACCTGGTTCTTCTTGGGGCTGTTCACCT
ATGACCACTTCAAATGAGGACGGGTACACAGCCTTCTCTGGTTGCTCGTCC
CGAGCTGCTATGTCCACATGTGAAAGCTGATGGTCAACCACAGTCAAACA
TCCTAGGTCTGCACCGTCCATGGTGATCTCAAGCTCTTCCATTGTTTCCTCG
ATCTGAGCAGAAACTGGTTCAGGGGATCTGAGACCAACAGGTACAAACAC
TGGTGCTTTGTTTAATTCTTCTGGGGCAAGATGACAGGTTTCTTCCTCATA
GTCAGACTCAAAATCCTCAGCATCATCTTCATCGTCTTTCTGAGAAGCCCG
AAGAGTCACCAACACGGACTTGGAAGCTCTAGGTTCCTTCTTAGCAGTCTT
ACCTCGAATTCGTTTTGATCCGCGTGCTTTGGTCACACTTGCTTTCATCTTC
CGATGCGCTCTTTTCCCACTATGATCTATCTCACACTCAGAGGCAGAAGGG
ACAGTTGTTCTTCGAGGAGGAACAGTTTTGGAATAGCTTGATTCTTTCAAT
AGTTGTGGACGGCTAATTTGTTTACTGAGACTCTGCTCTTCGAGGCTCGAA
GAAAATGTAGGCTCCTGGGCAATCTCTTCCCTGGCACCTCCTGATGGTCCA
GCTTTTAGCTGAGCGTCATTTCTGTCAGAGCCCGACTCTTCAGAATGTACG
CAATCTTTCCTCATGGAAACCTCCAGAGGCATTTCTG
>LNC_005698
GAAACTCATGATTGACTTCAGGTAGAGCAAAATGGATCATAGCTAGCCGA
CTGGAAAAAAATACACTTAGAATCTTTTACTTGCCCTTTCTAGAAATTGTG
GGATGAATTCAGCTGCCCTTTGTCCTCACCAAATTGTCTCATGCTGATTGA
AAAATAAATAGCTTGTTCTAGCCAGGGTTTTGAATATCAAGACAGTTTTGG
GGATTCCTTTTCCCATCAGTACCTTTTAAACATATTGTGTTTACCAAAATC
ACAAATGGAATCCACATTGCCCGAGTGAAGACTTGGCAGCAGCAGCAGGC
CAAGCAAATTCCAGAAACACAAACACGCCTGCAGCCTGATGGAGAGCCA
CAGGCAGAAGACAGCACAAGACAGCAGGACCTGGGCAGAGTTGGATTTG
CAGTCTCCTTAGGAACATGGCCTCGTAAAGCATGTCACTCCTGGCTGGAG
AATGGGTGAAGTAAATTGCCATCAAGCCCAACAGTGGGCTGAGGACAAA
AACCATGATCACCTTGTTCCCTTTCCTTCAGTAATGAAAAATTCAGACTTC
TCATGGAGACTAGGCGTATCAGTTCTTTTGCTGTCGCTGTGCCCAAATGGC
TTACAAGGAACAATGTAAGAGAGGAAGGATTTATTATTGTATGTATCTCA
AAATGCTCATTCCTTCTTGGAGTTGGGATCAAGATAGCTGGTCATATTTCA
CCTAAAGTTTGGAGTCAATGAACAAGAAGTAGAACTTGGCTATAAAATTT
CCATACCAGCACCCATTGATCCACTCTGCCCAGTGAGGATTCACCCCTTAC
ATGCGTCACAAACTGGACATTATCTGGGAATTAAACATTCAAACACATGA

GCCCATAAAGGATATTTAATATTGAAACCACAATAGGAGAATTCAGCTAA
TATGTTTGCAGTGATATTTAAGTATCACAAAGAAGAGAGAAGAGAACATT
TCTGAGAAAGAGAACAGGTGTATGATGTATGACGGTATTCTGTACAAGGG
CAGACATACATGCTGCATATATTCCAAGAAAGAATATTGTTTTTAAACAGT
GACCAAAATATGGGAACTTACAAATACAGAATCTATAGGGCGTATAGATT
CTCAAATTCAGGGCGGAATTGAAGACAAAAATATAGGGTGAAATCTGCCC
ACAGGTTTCATCCTAAGATAGAAGTTCTTTTGTGATACACCTCTGTCATGA
GCACATGATGTCAGAGACATAATATGAACACCTTAGACCTCTCCATGTTG
CCAGAATTCCTTGAATAACAGTAAATTCATGAGATGATTTCTTTAAAAATA
AAACTGACAGTTTCTCAAGTAGTCCAGCTTTATTTGATAGCTGCTCAAGGC
AAATGTAAGGCCCTTTTCCCCAATATTACTTACTAACTCTCAGATCACAAG
CTATTATCATGCAGCGAAGACATCTGCCCGCCCATCCATCCATCCATCCAT
CCATCCATCTATCTACCTATCTATCTACCCACTTACCTACCTACCTACCTAC
CTACCTACCTACCTACCTACCTACCTACCTACATATGTTACATAATGGTTA
TAGAGTTTTAATCATTATCCATCAGAGTTCAAAATGTTTGAAAGAGGCCCA
AAAGACTGAAGGTGAAAGAGATGTCAAGAGAGTTTCCGTAGGCATATGAT
AGTTGTTCCAGCCTAAAGTTCTGCTGCTAGAGTCTGGTTGCTATGTCACAG
ATGACTGCAGGCTTTGAGATGTGGAACAGGCTGCAGCAGTGAAAGGCAG
AACTAGATTGTGATGATTGAACAGGCAAAGGGGTGAATGGCAGGATCAC
ACTGGTAAACAGAAGGATAGACAGAAGATAGTTTGAGTAGGTCACATCAT
TTGTAGGGACTGAGATAAAAGCTCTGAGGCAGTGGTGTTTCTCTCACTGCT
AGCCCCTTGAGATACACACTAGGTGGAAAAAAGGTAGGTTGGTAAGACCA
CTGCCATCACAGTACTAAGTAAGTGACCACTTCTTAATAAGGTCAGGATG
GAGGTTTTGCACTTTTCTTCTGTCTGGTGTGTCTGATAAAGTTTCAGACCTT
GTTCCTCCTCATAGCATATTAATATACACATAACCTCTACAACCCCAATTG
ATTTTGACATATTTATAGGATGGCTGCCTTTCTAATTATGGGAAAAAGTTA
TTTCTCTGTATATGCCATGCAGGTGTTGCTAAGACAGGTGTACCATTCCAC
TGCCCTTCAAGGTGCAATGTCATCCTTCTTTCTGCAGATGCAGTGGAAACC
TTCTTATCAAGTGGAGTATCTTAGGGGACAGCATGGGTTGAAAAGCCCTT
GTTTTAGACATTGTAGGGTAACCTTGGACAGAGCAAAGCCTTGTCTCTGCA
CTTGTAAATGACCACCTGCTGGGTACATCACCTAGCCAAGCTGACACACC
CAGTTATCTACCATAGTAAATAACTGTGGAAGCTCCAGATATGTTTTCATT
ACAGAAGAGATACTATGCTGTCCTCTAAAGAAGTACATCTCAAATGATGT
GTAATGTATTTCATGCTCTTTATTAAATGAAGAGCAAATGCATGGGGACTG
TGCTGAGATTTTTGACTTGGCACTTGGTGCCGTCATCTCCATTACTGCTCA
GTGTCATAAAGGATTTCAAAAGAAATGAAGAAAAATTCCTGTTGTTTCTC
GCAAAAGAAAAATTCTTCATTAATGTCATTTGCCATCATTCTCATTCTCCA
ATGCAATCTTCTCTGCCAATATGATTATTATTTATTATAGATGTTTAGTGAC
ACAAGTGACAGAAGGTGTTTATTTCATATAAATGATGCAGATGAGTCCAT
GCTTCTGTATAGATGAGAAGCTGATCATTATTAGCTTTTTTTTAAATCACC
CATATATTATCTTGGAATTGTATCCTCATGGAATTATTTTTTCTTCACAAGA
TAGAATATGCTCATCAATTTTAATTGACACAGAACTAGAACCTACTTTTCC
TTTCAATCATGTTTCTCTTTGCCTTGGTTTTGGTGGGACAGAGGTACAGAC
CTTTTTCTGATGTTCTTGTGCTTTGGGGCTTGGAGTACAAAGGAATCTGTG

TTATCAGCTTAGCTCGTCACGTTCTCCGTATCCCTCCCTCCCAGCTGTTTGG
CATATTTGAAAGCCATTGCATTGTGTTCTGGCTACTGGGGACACCCTGTGT
CTGTTGACTGGATGTGGCTGTTCATTCTGCCCAGAATTCTGATTTACTGAT
CAATCAATTCTGGGTTGCTGGCCATGGGTGGGTTGTACCTCTGTAGTCACA
AATGTCTTTCCTTTCCTTCCCGTTCTGTTTCTCAGTGTTTCCAGACACAAGA
AAGCATTTGGCAAAGGCTGTGAAAGCCCTCCTTTGTGCTCTGGTATATTCC
CCTGAACCTCCACCTTGAGGTCTGGGGCACATCCACAGACGTACACCTGTT
CCTTGCAAACAGAGTTAAGCTAAAACCGCGTGTCAGCTATAGAATACCAA
CCAGGCATGGTTTTCAGACTTTATGTTGACTTTCCTGGGTGCTTTTGGTTAG
TTGAGGCTCCTTTTATTAAGAATGAGAAAAACCAATTTAAATCAGAATAG
CTGGAATTATGGAATTTGCTGACTCTCATAAGAGAAAGCTCTAAATTTGTG
CATGATCAGGACACTATAATAGGCATAATTTAAAATTGAATGCCTTCTTTT
ATTTTTCCTGGAAGACATGATTTCGTGGATCATGCAGCTTTCCTCCATCAA
ACATGGCAATAATTTGAGTATAAGAAAGTAATTTGATAAATAGTTTAAAA
TCCATTCTAACTAAAATAAACAGGTAGAAAGCATAGAGCACACAATTGTG
CCTTCTAGGGAAGCCAGCTTCTGAAGATGAGGTGTAAAAGTGATAATAGC
CCCTAGGATTAGAACACTCTTGATTTCCATTAATAGCATGCTGTGGGAAAG
GTAAGCACTCTACAGAGTTCTAAACAATGACCAGTATTAACAGAATTGTG
ATTATTCAAGTATTTTCTTTAGGCCTCTTGACAATGCCATTCAGAAATCAA
TTCATAAAGGTACTGTGTATGTATCAGCAAGTTACCGTTCTAATATAGAAA
GGGCATCTTTTGTTCCCACGTGTACATTATTAAAAATAGGCCCTTGAAAAT
CACAGCAACTTTGCCACCTGCAGAATATGAGGTAAAATTTCATAAATAGT
TCTCTGGCATGTGCAAAACTCCACTGTATGTGCATGCTTGTATTTTTGCCA
TGCCGACAACATATGGTATAATTATTATTTTTTGTCTGGTGTGTGTGTGTGT
CTCTCTCTCTTTCTCTGTGTGTGTGTGTGTGTGTGTGTGTGTGTGTGTGTGA
CATAGAGTTCATAAAAATTTATAAAGTGCTTCAAGTAGAACTGGTAAAGA
GATAGTAGTTTCTTGTACTTTCCTCTAGTTTTAAGTTCTGATTTTCAGTATT
TTGTGAAGTTAGTATTTTACTTGCTCACACATTTTGAATTATTGTTCTGCAC
TGGACATGCCACGATGGTTTGTGGACAGAATATAATATCTTGTTTTGATTT
CTTTCTAAGAATGTGTAAACTTTTGTTTATTTTCGTCTCACAGGGCTTGTGC
TAATTATCTTTGACTAATTTAATTGATCTATTCTTTCTTCTTCGAGAGCAAA
CAGATACTATGCATTAAAATCTAGATATAAAATCCTAATGGTGTGTAATTT
GACATTTTACTTAACCTTAATTCTCTTCATATAAAAGTTTAAAATCCTAGTT
CTTTTTCTAATAAATACCAAGTTATAGAGTGTCTAGGCTATATTACTGGCT
TTTCTGACTAACCTCTCTGACAAAGGCCTCATATATGTACTTCCCCTTCCA
GTTCCGCAGCTGAGAAGTCTAGGCCAGGTGTCCTCCAGGGCCAAGAGGGG
AGTGTTACCGCACTTCTCACACCCTTTGGTGGCCTAGCTCCCAGGCTGTGC
TGACATCTTCAGGTGCCGTTCCCTTCCATGTTGACCCTGGTCATGTTCAGT
GCCAGCCTAGCTCCGCACTCTTAGGGCTGTGTTCCCAGTGGCCACATTGCC
GTGATTCCTATATTTCCAAATAAGACATTCTGAGGTCTTGATGGTGTAGGG
CACCCATGTTATGTTGGAATTTTCTGAGTGTGTGGGATCTACATCTCTTCA
TAGCAGCTGCCATTGAGTGAATGTGGTTTATTTTATTGAACAAATATTAGC
TCCATGTCTGTGCAGTCCTGTAATAGGATGAGCTGGGCAGTGTTCACAGTT
AGATTAAGGCCCTCCCTCCCTTACAGCAAGTTAACTTCTTGGTGGTAAAGA

GGTGCTGACATTCTGGACATTCTGGGTATCCAGGTCTAGAGACTGATGAG
CAAATCTACTCTACTTCTACTCCTAACAGGAGTCTGATTTCATTCTCTGTCC
CTAGATTTTTCAGCCCAGGAAGGTCCGGTTCCTTGACAAGTCGCTTTGGTG
ACTCTAGTGGAAGTGCCTGCCCATGAATATGACAGCTTCTTGCCTACATTA
AAGTGAACTCCGACCTTCACTTGATACACATGAGCACTTCCCAATAGATTT
GACTAAGGATGTTCACAGCCCTTCTTGTACATGAACCTTTGGGGTAAAATG
CAATTTCTCGTGACTGAAGAGTGGGCTTAGTGTTAAGAGCACTTCCCGCTC
TTCCAGAGAATCCAGGTTCAATTGCTAGCACCTATATGTGGCAGTTCACAT
GCATCTATAACTTGTTCTGACTTCCACAGCACCAGGCCTGTATGTGGTACG
CAGAAGTGTGTGCATGTAAAACACCCATACACATAAAATATTATAATTAA
AAGTGGCTCCCTGGATAAAGGTAGTGATTAATAACAAATTTTAAAAATAG
TAGTTGAGGTTATATTTTATTTCATTTTCTTTTTGACTCTTCAATATTCCAA
AATCTAACAGTAGAAGATACCTGGCTTCTATGAGTGACCCAGGTGAAATG
AACAAAACATTGCATGGATTAACTTGTGCCTATGATCAGACACAAGGAGA
GACAGAAGCTCTGGCTGGGACTGCTTTACACAGACCGTACTTACTCCTAA
AATTTCAGAATGAGATAATTACAGATGGAGATTGACTTCACTGAAGCAGG
GATCTGCAAGTGCAAATCCTCATTTGGCATCAGGAGCTGCCTGCTTAGCCT
TTGGAATCTGTGGAGGAATGCTGTGTGTGTTCTGGCCCAACACTCCTTCCT
CAGAGGCCTACAAAATACATTCAAGAGAAATTACTATAAAAACTGTATTT
TTCCAAAAATCTTTGGACTTTTTTTGCAAGCTTCTTAGAAATAGGCCTTAA
ACCTTCTGTAAGTACGTTTGTTCTTAAATATCATCGAGGTTGTTATTTGAGT
AGATACAGGTTTTTTGCTATAACATTACACACTTTGACCATTTACATGAAA
ATATGGGAAGCTGGAGTTTGCAGGAAACTCAGCCACATGCCTGTCTCAGA
ATCAGCTTGAGAGTGACAAGGATGGGGATTGTGCTTGAATTCCCTTGTAC
CCAATTTATTATTTTAGTACAGCTATGTTTTCACCATTTGGGGCTACTGATA
ATTCATAATTTGGAAATAGGTCAAACTACAGCCATTAATCATATTCTTGTT
TGGTATGTGGAAACCAAACAAACCGCTTGACCTCAAAGTGGAATTATTAT
TACTAAGGACTGGGGAACATTTAAAGTGGGACAAAGAGTGGAAGAAGGG
TGGTAAGTAAACAGTGCATACTGTGTGCGTTTGTCATGATAGCATACTAA
GCTTGTAAGTCTTTTTAAAATGCCATTGACCCAGTAAGTTGGGAAATATCA
ATTAGCATTTCTAATGCGCTTCTAAATAGAAGATAAAGTGCTTAGAATTAG
TGAGATGGGAGCAAAGATGTATGGTTTTAACTATAATAAAAATGAGGTAG
TTACTTAAATTACAGCACGTTTACTGACAAAGGCAAATTAAAGGAGTAAT
GGTTGGTTTGTTTCACAGTGCCATGCAGAGGCCTATCATGGTAGCAGGAG
GGTGATGCACCTGGTTACATTGTATTCACAGTCAGGAAGCCGGAAGAGGT
GAATACTGGTGCTCAGCTCACAGCCCATTGGATGGTTCTGCCCACTCACTC
GGAGGGTAAATCTTGTTACCTCAGTGAACCTGAAGTAGACAATCCCTCAT
AATCATGCTGCAGGCCATTCTTCCAGGTGACTCTAGACTCAGTCATGTTGG
CAATCTGTGCACATGTAACAACATAATGACACTTGTGTTATTTCTCATAGA
ACATTGTTCTCAGTCTGGATGCCACTTCATTGAGGTCAGGATTGTAGACGC
AGGGTGATTTTATTCATTTGTTCCCTAACACTGAAACAGGCTTCAGGGAGC
TGGTTAGTTTCAGCACAGCAAATAGATGAGAACGTTTAAAAGGTATCGGT
AGAAGTAGGTTCCCTTGGCATTAAGAACACCTCTTTCCTCTGATGATGCGG
GTGGCTGCTTGTCTATCCAGGCACCATTCTTTACACACTCAATGATACTGA

CTTCAGCAGAGGGGTCAAGTGGACGGTGGGTCAGGAACTGCTCATGGGAC
AGTATTCTGGTGGACAGTTAGGCTCCTATGCCATTTCCACTTGTTCAGAAT
TTTTCAGAAGATAGGCGATGTAGCTTTCTGTGAGCAGGTAGACAGTGACA
AGACTGTGTGGGGACTGTGCCGTTCACCTGGGGTTTGAGGAAACAGTGGT
TCCCCTTTTTGATTCAGGTCATCATACAGAAGAACCCAGGCTTAGACTAGA
TTTAATCCTGGACTCTGCCCCTCAGCAGTATAAAGAAAATTATCTTAGGGG
TCTCTGCCTGACTGCAAAGAACAACTTTCCAGGGAGAATAAAACATGTGC
CACAGAGGAAATTTTACCTTCCTAGTAGTTACCTTTAACAACGAAAATAA
AATTAAGCAAACCTATCTTTGTTGTCTATTAACATTTTATAAGATCCAATA
TATTTCAATAAAATCGTTTGAAGCATACATTAAATTGATTTCTCTTCCTGG
GAAATACACAGTTGTTGGTAATATCTTAGGTGTATTCTATCTTACTCAGAT
TATCCTGACTTTAAGTGCTGAAAGCCAAACATGACTGGGGCTGCCGTATT
GGTAACACATTATAATAGAAGTTAAACATTCTGAAATTGGATATAATCTT
GACTGGTTCAGCAGTTCAGTTCAGGCTTGTTCCTGAGCTGGTCCAACTGAC
CGGTGTCATAATTGTGTATAAGGGGCCTTGGTAGAAAGTGTGGGTCCATT
CTAGATCAGAGCTTCAAACCTGAATAATGAGCCTGTCAAATTTGGGTGTG
CACGCTACATCTTTGAAGATGTACCTGGGAAGAATGGTTCTGGAAGTAAT
CGAATTGGATTGTGTTTTGAGTTCCCTGGTAATTCACGAACAGTTGGACCA
GACAGCACACGGGATAGAGCAGCCTGGGATGACATCATTTGGGGGAACCT
CTTCCTGTTTGTTATTTTACATTATACGTCTATTGCTAACACTACTAAGAAC
ACTTTCTCCATATACACTGAAACAGACCCCCGGGTTTCTATACAGTTCTAA
TGTATTTGGTTCTCTAAACTTAGCTTTAGAACTGTGCATTTGTTACTGGTAT
GTCTGTCTAGATTCAGTCCATCCAGGGTGAGAGGCTACCGTGTGTCTTCTT
TAGTTGATGTATTCAGAATCGGCTCCTTCCTCTAAATGATTTCTTTACTACT
TGATGCATTGTATTTTATGCCCAAAAAATAGGTCAATATCTTTTGTACTGA
CAATGAATCTAAGATTTTGTCTAGTTGTGAGAATTTGGACAGTAGTTAATA
AGTGGATTATATCACTATCTTATAGAGAAACTCAACACCAGCCCGTATGG
AATCCTGAAAAACTCTTTGGTGATATTCTGAAATACAGTGTCTCTCATGCT
TAAATATTTACTACTAAGTCTTACTGTATCGAAGAGAAGGGAGCAGCCCT
TCACTGCTGGGGAAAGGTGATATGTGTAAAGGGATATGAGTAGGACACTG
TTGGGGCAGCTGGTCACCGTAAGGTTTTCTCATGGTGAAAGATGAAAGTC
AACAGGATTTCAGAGCCTCAATTTACATACTGTGCCCCTCAATCCAGTCTC
TAGTTTTATAGATTAGCAGATACAGACAGGAGGCGGTTACAATGGTAGTT
TTCAAACCAACTATGTCACCTCGGCTGAAGTATAACCAGCTAGGGAGCAC
GCTGCTCCGATCTTCTCTGACCTTGTAGCCACCACATTTTCTTTTGCCTTTT
AGCCTGGCGTGTCTCCACTTGTGATCGTTCTCAATGTGTACTCCATAATCT
CTATAATATTGTCCTTTGACTGTTTTACCCTGGTACCTGACTGTGTATGTGT
GGCCAGACAGTATTTTTACAGATTTAACTAACATATGACCTAAATCTAGG
AATCCGTATTAAAAATGTAGATTCAAACCTCTACCTATAATCATTAATGGG
GTTATAATTTTTTCTCTGAAAGAAATTAGCTGTTAATCACAAAGATGCTTG
AGTGTCTGGATGTAGCGCTTGTGATTGTAACTTACGAGTGAAAAGCTGTCC
AAAGCTGACCAGGAAGGAAGGAGGGTGACGTGTGCACACAGGGATTTAT
TTTGCTTTTCCATTCTTCGTTGTTTACTGAGAAGAGTCCAGGTGACCTTTTA
TCATTGTGAGAGCTGGGTCATCTGCACAGTGAGCTACTCGGACCCAGAAC

GGAAGGAATGTCACCGCCTCTATGGACTTACAGATATTCAGTGTCTTCTTG
ACATTTGCATAGTGCATCAGATACAGTGTGTTGGCTTTACGCTCGTGGAAG
TTAACATCAACTTGTTGGAGACAACTCTCTGTGAAGATCTGTGAGGGATTG
TTCTGATGAGATTGAGATGAAAAGACCCACCTTCAGTGTGAGGCTGGAAT
TCTGGACTATAAAAACAGAAAGGTAGTCTGAGCCCTCAAATTCAGTTTTCT
CTGCCTTCTAGGGGTGAATTCAATGTGTCCAGCTGCCTCTTTCCCCTGCTTC
TGTCTCCTGCCAAGTAGTGATAGTCTACACCTTATAACTCTGAACCAAAAT
AAACTTTTTTTCTGAAGGTCCTTGTATTCTTTTGCTGCAACAATGAGACAA
GTAACTAAGAGTTGTTATTATAGTGCTAATAACCAATACAGTCCTGATAAT
ATGAAAATGGGTTTTAAAGGTTGTTAGTGGCTACTCGGTGTGAGAGGTAT
ATACAGTGAAAAGTAACTCCTAAGGTAAGTACTCGGACTTTCAGATATTA
GATAATTTCTATTTTAAAATTAAAGGCATTATTCACTAACATTCATTTCTA
ATAAGCCTAAGGCATTCCCCTGTGCCTACTTACACGCACTGGCAGTTTGTT
ACAGAGTGCTAATTCTGGAAACATTTCACCTCATGTAGCAAGGTACCCTG
GATTTACTTAGTTATGATTCCAGTTAATAATATTTGCATTAATTCTTTGAGA
ATTCCATATGATAATATTTTGATTATATTTAACTTCCCTTCCCCTAAATCCT
CCCAAATCCACCCCAACCAACTTCCTGTCTCCTCCCCCAACATTTGCTACC
AAAATATTTGTGACTGTGTAGCTGCCTTGCAGCACGGGTGCCCTGAAGCA
TGTGTACCCTGGAAAATCATTGCCCTGCAGAGTGGGTGCCCTCCAGCGTG
GGCAATTCTTGTGGAAGTTTTTGGTGTGGTCAGATTTGAACATTTGAATCC
CAAGTTTTTAAAGTTCAGTTGAGATCCCAAAGTGCCCTGTAAATGCTAATT
TATTTCTAAAACAAACAAAACAAATTATAAAGGATATTTAATGTTTTTATT
CTTCAGATTTCTACACTTCATTGTTTACTCAGATGTAAAATATAGATTAAA
AGGTAGCTATAATCTTAGTTACTTTTAAGAACATCAATTTTATAGAGCGTT
CTTACTATCCAGTAACAGATAATGCTATCGGAGAGGCATTATTTAATATTA
TTAACTTTAGAATCTTTTGAGATATTTTATAGTGATCCAACTTGTTTTTAAG
AAATATGAGCATCTTGTGTGTTTGAATGTGGGCATGCATGTATGTATAACA
TATCCTTGTTACATGCATATGTAAGTTATTTCAAATATGTGAATACAGACA
TTTGTATGTATGTGTGTATGTGTGAAATTTTTTAGTTTTCCAACATGCATGT
ATATGTGTACATGCAAGCATATGTGTGTTTGTCATACGTGAATGCATATGT
GTGTCACTTGTATATGTGTGTCAATGGACATGTATATGAATGGGTGCGTAC
TTTTGGTTCTTCTGTGTGTATCCGTGTATGTCCATTCATACATATGTGTTTG
CATTTGAGTATTTGTGAATGTCTGTGTGCTTTCCCACATGTAGTTCCCTCAC
ATGCATGTATGTGTTTGAACATATATGAGTGTTTTTGAAACATGTTTTGTG
TGTGTGTGTGTGTCTGCACATCAGAATATTTACACTTACTAAAATCAGTTA
TGTTCTAGATGAACTACCTCTACTAAATAGAAAAAAATTGTTTTTCACTTT
TATAATTTATTTAAAAATAAAAATCCATTTTCAACTTTCCCAGTAGATGCT
ATTGTAAATTGAAGGGTTGAACTTAGTTGTCAAAGAGTAACGATTTATATT
TAAATACCTCTTCACAGTGGTTTAAAACTGTATCAATTAACTCAGGGTGTT
ATGTGGCTGAGGGCTGGTTTTTGTTGCTTAATTTTAATCAGTGTTGCCTCCC
TGTCCTGGTTCCTGAGAAAGCTTTCTAAAGCTCTACCATCCTCATGGATTC
TCGAGTGTCTGCTGTCGCCTTGCTCCTCCTTCCTTACCTTCCCCTCTTTTAC
TTCCCCCAATTTGGAGCTCTGACTGTACCTGCAATACATATGGCTTTTTAA
GTCAGGGAAGGGATTGAAAATAGGGATCACCCAGGCACCAAGCCTGGGG

CCATTTCTCCATCCAGACACCCTTGCTTTGACTGCAGATCCTAAATGAACT
CCACTTACCCAAATGCAAGTAACCGTATTTGTGCGGTGCCACTAAATCTGA
GTCAGTTGTTACTCTAGAAAAGTCCATTTTTGTAGGATTCTCATGATCACC
TGGGTTTTATAATACACAGATCAAGAAAGACTTCGTGGGTGTGATGTGGC
CCAAGCCCTACCCTACCCTTTCCCAGCTCTAAAGACTAAATTGACTACTAG
AAAATACAAATGTCACTTCCTTATTTGATCAGACACAATACAAAATGCAG
AGAGTAAGTGGCTATGAAATGCTCAGCCCAAAGTGGGGTTCGTGCTACAT
ATGTTATGTCCACTCCACTCACAGCTAAGAAAGCCTTGTAGAAGACAGGG
TGGAAATATTGCAAGATACAAAGGTCGTTGGCAATTGCTTGCTGCTAAGC
AAAGTTCTGTGGACACGACAGGAACAGTGTGTACATGAACTCATAGTGGG
TATGATTGCATCCACAACACTACATGGTCAAATCAGATAAGACTTAGCAT
GGATGGAGGAGGGTCTTACAAAGCCCACGTCTAGCTAAGAAACTATTGGT
ACTTGATGTCTGATGAGGGGAGAAAGAGTCAATTTTCTTCAAAGATTTGA
CCACAGTAAGTTGCTTATGAACCAATGGATGACCCTACTTATTGTCCTGAC
AGCACTAATTTATCTCAGTGAATTAAAAAAAATGAAGACGTGAATCTGAG
ATATGGTGGGGTTTCTTGGAGGAATCGAAGGAAAGAGTAAAGAGTGGAT
ATGGTCAAAACACTTGGTATACTTTGAAAGTCTCTAAGAATAAAATCATA
TTTCAGAAGGTACAATATAGTCATATGGTATGTATGTATATATGTATGTAT
GTGTATATGTACCTGTCTACCTATTATCTATATTTATCATCTATCTGGCATA
TATAAATATAACATGTGACTGAATTGATAATATGTAATTTTAATCACATTA
TTTTCATAATAATAATTGGATGATTTTACTACTGCTTCCTCTTTTACCTTAA
TTTCAAGTAGGATTAGCTTGCTATTGTATTTCCTTGACTCTGAGGAAATTTT
ACTGCAAGATTCACCATTACAGTCTACCTCAAAAACAGTGATGGGAAAGA
AGGGATACAATTTGAAGATGTAAATCTTCAGACATAATCTAGCATCAAGC
CACATAGGAAAACATTCTCTCTTAGGTAGAGTCAGTACAATATTTAAGGC
TTATATTTCCTATTTAGCAAAGAGTATTTGAAAAAAATCCCAGAGTTCAGG
ATGGTCATGAGTTGAGTGAACCTGCCCTTCATCCTAAAGCATAAGGTGTA
CAGAGATTGTTGGAGTTGCTCTCTCTGGCAGCTGAGCCGTGTTACCTCTAA
TCCCAGTGGTACTTTCTGTATATGAATACATATCCCAGCCCTTATCTTGAG
TGCTTCTAAATCATGGACAGTCTGGACTTGTCATTTCATTTAATTGCCATG
TGACCCAGGTACCCACTGAGGTGTCAGAACCAGTGTTCCAGTGCGTACAA
CATCTGTCAGAGAAACGGTGTACTTCTGTTCTTCAAAGGATACATAGGGG
AATCCAATCATGTAGATTTAGGAAAGGGGAGAGATTCCATAAAACAAAG
AGCTCAGTCTAGGGACTATAGCTTAGTGCAGGTAGGGGAAATATGTTATC
TGGAAAATGATAATCACTGCTGTTCTTTTGAACTCCTGGAAAGGGCAAAT
CCTCTAGGAAGGGAGCATGGGGATACAACAGAGATGATATGCAAAGGAT
GTTTGGTGTTCTTAAAGATTAGCTGTGTACAAACCTGATAGTCCCAGCATA
GTAGGAAACTTAAGTCTGGAAGTTAGTGTTAGGACCCACATGGTCAAGCT
ACAGGATGAAGCAGTGCGAGCTTGCCATAGAGGAGTGGCTGCTGTAGTGG
GGAAGTAAAGAGCTGGAAACACAAGGAGATCAGGACTCCACATCAAATT
GTTCTCAGAACTGAATTATTCTTGAAAGATGAATGAAAACTCCAAGATCT
ACCTGTGGACACAGAAATAAAAAAGCAAACAACACAAAAGTTAAAAAAA
AAAGAAAAAAAAAGAAACTCAAAGTCAGTCTTAGCTAGGGTTTTACCACT
GTGAGAGACACCATGACCAATGCAAATCTTATAAGGACAACATTTAATTG

GGGCTGGCTGACAGGTTCAGAGGTTCAGTCCATTATCATCAAGGTGGGAA
CATGGCAGCATCCAGGCAGGCATGGTGCAGGAGGAACTGAGAGTTCTACA
TCTTCATCTGAAGGCTGCTAGTAGAATACTGGCTTCTGGGCAGCTAGGCTG
AATGTATTTAAGCCCAGGCCCACAGTGACACTCTACTCAACAGGGCCACA
CCCTCTAACAGTGCCACTGCAAACAGTGCCACCTCTAACAGTGCCATCGC
AAAGTCTGATCTGAGGGGGCAGGAGGAAGCCAAAACAAATTGAATTTCAT
GTACCAAAAGATCTGACTGGTCTGTAAAAATAATCATTCTGAAAAATGAC
CCTTTTTGTTACTTACAACTCTACAGCTGGTCAGGGTGGAGATGTTCACCA
TGGGTGGATGTTCAGTATGGCTCAGTCCAGCCGTGCTACCTACAAGGCAT
CCTTCCTCGCTTTGCCTTTACATGCTAGTTAGTGGCGATTATTTATGATTCG
CAAAGAACTTCCACTTACATCACCCTACCAATGTTGGAATAACCCTCTCCT
ATTGGACAAGACGAATTTTATGTCAGTAGCTTGCTATACTATACAAGTAAT
AGTTTATAGAAATAGGTAATAAACCAACCCATTGCTCTGAGTTTAGCATTT
TCATTTCATCCCTGGCATAACCGTACTTTGTTATGGGCTAGAGTGTGATCA
GGTAGTAGTACATTTATACACTTCTTAGTCAACATGGGGCACCCACCTTAA
ACACTTATTTCATTGTGGCCTCAGGTATTTTAATTCAAATTCTTTGTTCTTT
TTCCTTACTGGTTTCCCCCATGATGTTGAGACCAACAATCTAGTGCACATT
GTAGAATGTTTCTAAGCTGAAAGAGGGCAGCTTAAGCAGAAGCCTGAGGT
TTGCTGTTTTCCTTATCGCCATGTCTTACTAAGAAAATTGGCACCCTGTGA
ATAAAGGTGGCTTAACTAAATTTAAACACACAAAACAAATTAAAATGTCG
GATGCTGCTGGTTCCCTGACTATTCCGAGATGATCCTCTTCCCTTCGCTAT
GCAAGCATTAGACGCTGTGCCAACTCCACTTGCTGCCCCTTACCACCTTTC
CCCAAGAGTCTCTGTCTGGAGCTGAAGGCTTCTGAAAATGACCCATTTTGG
AAAATGGACATTTTACCATTGGAACAGGTGTTTCGTGTGGTGGTGGTGGT
GGTTGTGGTGTTGGTGTGTGTCTCTATGTGTGTGTGTCTCTATGTGTGTGTG
TTGTGTTGGTTAGGGTAACCCTACACACAAAGCCATACACACACGTCATA
TAGTTGTCAGCTGCCTGGGTTTTCATGCGTGGCTACTCTTGGTAAACTCAA
ATTGGGTGCAACAATGAATGTGTTGATTCAAACAAATCTACTCCAGGACA
TTTTTAAAAATTAATTAATTTATTTACTTTACATCCTGACCACAGGTTCCAT
CTTACCTTCCTTCCTCCATATTTCTCAGCACTTTAAAGAAAGTTATTGCCTA
GATTATTATAACGTAAGGGGAAATAAAAGAATGTCAACAAAAATGATATC
GTGTACCTCTGTTTTGAGGTGCAGGGACTTTCTTTTGGGGCTTCCCTTCTTT
GTTTGGATTTTAATTTACATAATTTTTTGCTTCACAGGGATAAACGTTTGG
GTGCATCCTTTCTTGTTTTAGTTTGGGTCTCAGCGGTACTTCTCACTTCTCA
GAATGAGGGCAGTGTGGTTTCACAGAGGTAGTCGGTGGCATCTATACACA
GTAGGAGGCAGAAAACTCTCATGTTTTCTTTTTCATCATTACGCTTTGTGA
TGAACTAAGTGCTTTCTATCAATACAGGAGCACAACTCTGCATTTCTAAGG
TTTCACACGATAAATCTACCCCCTAAAGTTATTTTCTGTGAAACTTTCTGG
AAGATGGGATTAACTTAATGTCAACCTACTCTTCACTTTTTTTTCTAAGTTC
TCTATAAAGTGTGAGTTTAAAAGTTTCTCATTAAAAAAAACACTTCATTCT
GTTCTTGGTACAATCCAGATCATTTTATTTGTTGACCTGAAGAAGGGTGAC
CCCTCCAAACTTTTGAAAATGAAATATCACACTTTTAAAGGATCCCTGGAT
CGAAGAGGAAGGCC
>LNC_005763

CTTCTTTTCACAGTAGAAATAAATTATGTACAATGTAAGTTCCTGCCTTGT
ATTATGCTGTTTTGATAAATATGCCATATACATTCATTGAACCGACTGACC
TGACAGATTGATACCTCATGGATTACGTGGTTGCTTTTGTTCTGCACAGCC
CCGCTAGCCTGAGCACAGAACAGAGTTTGGGGTTGGACAGACAGAGTGTA
CATTGTATGGCGAATACCCATCCTGCAAGAGGAGGCTTCCCGTGTGCAGG
CTGAGGCTGTCTGCACAGGAAGCCTGAGGGCTCTTTCCCTCTCTCCTCTGA
CCTTTCAGCCGAGTGTATTAAACACATACAAACGTGCAGCAGGCATGAAA
GTAAACAGAAAGGAACAACTGCTTGAATTCTGCTTTCCTCATCAACCCCCT
CAACACCTATTTGGGGGACAGAGACATGGGCTAGGTTCAAGAAGCTATTC
ATTACGTGAAAGCAGAGGAATCTCCCATCAGGGGACACTCATTACCAACA
GCAGCCGTGCTTTAGATGTTACACAGGACTAACTTAGAAGTCATTGGACTT
ATCCTGAAATCTTCCATCTGACTTTTTGCCTCTATTTATAATCCCAAATCCA
GGGGCCATTTAAGCTGCTGTGTAGAGGCAGCTAATGGACAAGAGAGCATT
GCTACCTACAGAGGAGACAGAGTAGTACAGATTCAAGTCAATATTGATTT
ACCTCCAAGTTAGCTGAGAATGGGAGAAATTGACCTACAACCCCATACAA
GGAAAACTATTAACTCTCAATCTTCAAGGTAAAGACTCATAGATTAAAAG
CCCAAACCACCCTTGGTGCTGTTGCCAGGCAGCAATACTCTGCAAACGCC
ATCAACATCAAGAGAGCAGCGTCTTCACCTGCTCGGGTGACCTGTGAAGG
ATGCTCTCCGGGTCTTTGTACGCGTTGACTGGATTCATCACGGCTGCATGC
ACCTCCCCGTAGGCTCTGCAGACGAGCTCCGTAGACTGCTTTATAATCTGC
TCCTTCACGGTGGCACTGAGGAGGGAGTTCAGCTGTGGCATCAGCAGGTG
ATCTGGGGCTGAGAGGTAGCGGTCGAACTGAGCCATTGCAGCCTTCAGCG
CCACAGAATCTAGGTTGGGCATACTGGCTAAGGAGCCCTGATCAGGCCTA
TGCTGCTGTATCGTGTTGTAGATGTAACTCAAGCCCACTCTAGTTAAAACG
TAAGAGGCTTGCTCATTGATAAGAGTGTCCAGATGGGCTTCGATCTGAAA
CTGAAGCATTTCCAGGCGCCTGTCTGTGAATTCAAACAGAGCCAGTGTCG
TCTTCATCATGTACAGTGAATTGACCATGAACGTGGCCATGTCAGCGGTGC
CTAAATTGCTGGCTGACACTGTGCACATCTGGAGGAGAGGATCCAGGACA
CATGACAAAACCTGCACAAAGTCAGCTTGCCGAGCATCCAAGGGGACCAC
TGAAGAATCATGTGAGGCCAGAACGTCCCGCAGCAAAGTGAGGGTCTGAC
TCAGGGCAGAACTGGGTCCGAGGTCAGGTGGTGGGAGCTCGATCTTGTCC
ATCAGTTTATTCGCATGGAGACTCAAGCTACTGAAGAATATTTTTTTGCTT
AGCAAGTGCATTTCTTCAATTGTTGTCAGTAGAGTAGCTGCACTATTTCCA
ACAATGCCACTGATGGTATGGTGGTAAAACTTGAGGAGGTTAGAAATTTT
ATATAATAACACTGCCCCAGGTTCAGCAAGTATTACTTGTTCAATTCGAAC
CTTTAGAGGCCGACACACACCCTCGGTGATATGCCCAACAACTTCTTGAAT
ATTTTCTTTAACACCCTGTGCAGTTACATGCTTTAAGAGAGCTTCCAGATG
TTCCTTCTCAGAGGCAGTAGCTTGATGGAGCCAGGCCAGCATGTCTCCCAC
ATACCTCAGCGGGTCATGGGAGTGCATTTCAATGGGTCTAGGTGTACCTCC
TGGCCCTCCTCTTGTAAGAGCATCAATAAACCCACGAACCACAGTGCTTC
GCCTGGCCGTTCCAAATTCATCTAAGGTATACTTGTACAAGACAGGCCTGT
CCTGCAGGGCTTCCATGGCCTGAGTTAACACCGCAGACACATCACAGGAC
TCCTGAGTCAGTGCTCTGCACTCGCTTTGAGCCCACCGGTAAAGTCTTTCA
TAAGCAGTTTCTTGAAGCAAGGCCATCTGTTCCATAATCTCCAAACCTGCC

GTCTGCTGGTTGGTTCTCAAAAGAACTTTGACGTCATTATGAATTTGTTTC
ACTCTTCCCAAAGCCTTGAAAAAGTCCTCAGTAACGGGGCCTCCTCTTGTA
CCTCGGAGGAGAGTCATTTCATCAGAAGTCAACTGGAACTTGGATAAAAA
GGCATCTGCAACTTGTGCTCTAATTTCTAGTCTTTGGTTTTCCGCTTGAAGC
TTAGTGGTCTTTACTATTAAATCTTGAGTCTGTTCCTTTGCTGCCTGTAAGC
GGCTTGTCATGTCCTGACAGCAGCTGCTCATTGCTTGTACGTCTTCATTGA
TGCTTTCGAGTTCCTCCTTTACATCTTTGAAAATGCTTACAAATTCCTCATT
GATGGCTAAGCTTCTACGTTCAATATCACCGCGTAGATTCCGTCGAGTCCG
CAAACTATTTTCAACAAAAAAGGCTGAGAGGGCCTTGAGAGCTTCCAACA
TCTCCTTGTCATTTTCAAGCCGCGTTTCCAGGATCTTGTGCAGCTTGCGCG
ACAGCGGGTTGTTGGGCTGCGCTGGGGTCGCGCCAGCCCCATTGCTGAGG
CCATTGGCGGCCCCGGAGGCGGGGACCGCCGCCACCTCCCCGCTGGTGTC
AGCCATCGCGGACGGACTGG
>LNC_005818
TCGGAGCCACCTTTAGCAACAGCATTTTTTACGACCACAGAAGCAAAATA
AATTCTAGCTTGTCCTCTGGTGGATGGCTCTCTTTCACTTCGAGGGCATTTT
CTCAGCCATGTGCTTCTGCTGACTCTCAGCGTGTCTAGTCAAGTCTTCTAT
GTCCACCAGCATGGCGTCTTGTTCTGTGTGCAGCTCATCTCGAATGAGGAG
CAGCTGGACCAACTCTTCATTCAAACTCTCAATCTGGGAGTGAAGGTCATT
GACGATCACTTGTAGCTGCCCGATAGTCATGTCTCTCAGGTCGGTGGGCTT
TAAGCTTCTTTTCATCAAGCATTCGCTTATGTGAGGCATGTGAGGCAGAAG
GTCAGCGACGGGAGACTTTTTCCTGTTCTGTTTTTCCACTTCTACTTGCATT
TTGGCCATTGGTTTGGCCATGGCCAGGGCCATTTTGGCTTCAGCTTGTAGC
TTCTTTTGCCTTGTAAGGAAGTCCATGTCATCCAGGGATTCAGATTCCTCC
TCGGTGGTGTCTCTATCAGAGTAGGAAGAACTCTGCTTGCTCATGGGGGA
CAAGGGCGTGTCCAGGCTGGTCTCAGTCTTACTGTCGTCGGCATCGCTGTC
CTTGTCACTGCCGCTGTCGTTGACAAAGCATATCTGGAGGTTCATCCCACT
TTGTAGTCTCGAGGAAAGGCTTGGCTTCCCGCTTTTGCTGCAGCTGGTATA
GATTCCGGGGCCATCGTCAAAGAAGCTTCCGAGTGCCAACTTCTGTCTGAT
AGACTCTCTCTCATTCTTCTGTGCAGAGGTGGAGTTGGTCCTCGCCTGACC
CTGATTTCGCACCTCCTGCTCCCGGAACTGCAGCCCAGCCAGATGCTTTTC
CAACGCCTCCCAGTCCATTGGGGGCGCAGGGGGTTCTTCCGGGACGCGCA
TCCCGCCGTCCGGAGGCTGAAGGCAGAGCCCCCCAGATGCCCGGTTCCGG
CCTCCGCGCCGGTTTCCTGGAGGCTGGGTCTTCTGGATGGGTCGCCGGGG
AACCCGGGAGGCGTTCGGCCGGCCGGCTATCACCAGGTTGCCATTGTGCC
TGAGGTCTTGGGGGTCGTGCGGGTGGAGGTTGCCGTTAGGCATCGGGGAA
GGCATGGTGGTGGCGGCGCTCCTGTTCCCGCTGCCGCTGCCGCCGATCCTG
GCGCTTACGTCCCCCGGCTCCCGGGCCGGGCAGCGGTCATCCCGATACCC
TCGATCGTCCTCCTCTTCCTCCTCCTCCCCGTCCTCCTCCGGAGCCCACTCA
TCAATCACCTTCTTCTGGTAGACCGGGAAGGGCTCTTCATAGTCTTCCAGA
GCAGACACCAAGTCCAGGCTGCCCCCCGGCGACGATGAGGATTTGCACTC
GGAGCAAGGGGTCACTTTGGTGGAGTTGGACTGTGAGCTGGCGCGGCTGC
TGCTGCTGCTGCTGCCGGCGTCACTGCCTAGATCCATCCCATCCTCTCGGT
AATCCTCAAGGCCTCTTGGCTGGGCCCTTTACCCAAGCGAAGCCTCCCGG

AGGGCCGCGCGGACCTGATACCTCCCAGTCCACGCGGAAGTTGCCAAGTG
ACCCTGAGAAGGAGCTCCTGACATCCTCAAAAACAGATAGACAGGCCGA
GTAGGAGCTCAAGTGACCAGTGGTCAGGCCCTGTCATTTCCTTTCCACCTT
CTCCCTTGCCGCTTCCTCTCCCTTCACCGCGCCCTGGTATCCTCTTGCAAGT
TCAGGCAGTGGGTGGAACCTGTTGCTTTTGCTCACGTTTCCTTCCCCTTCCC
AGCGGCATTGAGGATGACCTCAGCGAGGTGTGTGTATGGCTAGGAGAGGG
AGTGTGAGCAGGGACTGTTATCTAACGTGGAGGTCAGGAAGTGGGGTGGA
GATCTATCCTGAAGGTGGGGCCAGACATCCCTTCCTACCGAACGGACCCA
GGAGTTTGTAAAGGAGAGAGAAAGGCGGTCCTGAGCGGTGCTGCCCCTAG
TACCTTGGCTGGCTTTGCTTACTTGATTATTTCTTCCAGGGAATTCAATTA
AAAAAAAATCTGTGGGAAAACTCCCAAGACCATACACGGGCTACCAGAA
CTTGCCGATCCTTTTTTAGGAAGAAGTTTCAATGAAGTGAAACTATTCCCT
TGGATAATGTGGATGGACTACAGAATACCTTTTAAGGACTAAACATTTCCT
CTGAACAGTCTTGCCCTGTAAGAAGTTAGCTAGGAAATGAAATGCCTCCT
CTGCGGTGAGAACGGAAGAGTGCTAAACTCCACAATGAAATGGAGAGTTT
CCTTGGACTACCAGATTTAGGCCTGTAACTAATTTATTCCAGCAGGGCTAG
TCTGGATCAAACCCCAGCCCTTGCTTGGCACCCTCCAAATGGAGGAGCAG
AGGCATGCTTGGGATTCCTGGGTAGGGAGTGGCTGCCACCCGGCCAGCAG
GGCTCTTGTAGGGGACTTGGTGCAGAAACACTTTGTCTCAGTGATTTCCTC
TTTGGTAAGTTCCTGGCCAGGAAAGGTTATCACACCTTTTTAACTTGTCTG
GAAGCAGCAATCCTGTGAATAACAGGAAAAGAGGCCTCTGGTCTGTCCAG
CGCTAGAGTCGAAAGGGACTGGTTAGGACTTAAAATACAATTCATATGAA
CATCTGTAAACAACCTGCTTGGTGCCTCAAATATGTTTTGATGTGAGGTTG
CAGTCGAGTACTGGCTGCCACTGAGAGGTATGGAGGTGGCCCTGTGTTCT
GTGTCTTAGATGGGAAATATTGCTCAGAAGATCACCATGGGACAATGACA
AGTCTACCTAAAGGGCTGGAGAAGTGGCTTAGTGAGTAAAGTGTCTTCTG
TAGAAGCCAGGATCTGGGTGCTACCTGTAACCCCAACATGCTAGAAATGG
CCGCAGAGACGGGAGGATCCCAGATAGAGGGAATCAGGCTACCCCAAGT
TGCAAGCTCTAGGTTCAGAGAGAGAGAGAGACCCTATCTCAAGAAAAAG
ATCTCAACATAAGCTGAAGGCGAATCTGGCCTCCCACGTTTCTGCACGGG
CAGGTATACCTACATGCGTATGTATACACACGTGCATACAGTACACAAGC
ATACACAGAGCTACATACTCTGTGGCAGATGCAGGTAAACAAATATGGAT
CAGCTCGGGTGACAGGAACACCATAGTGACGGCCCAGTTTTAAGCACCA
GGACCTCCCGAGCCACCCACCCGCACAGCTGGCACACTAACGCCTCAGCT
GCTTGGTGATCAGGAAATGTAAGACGAAGCCTAAACGAAACAGTTGGCA
AGGTAAGTGCACTGCCAGGAATGCATGGCGTTTCAAAACCAACTACAAAC
ATCTCGGCATTATCTGTACTTTCACATTGTTTCCTGACCAGACGATCTGGA
TCCGATGGGAAGTGCCAAGTCTCATTTTCCTCCACGAACCATGGTAAGTGT
TTGGCCAGCCTGGTTACCATTCTGTTTCCATGCTTCCCTCCAGTCCCCTGCC
AAGAACACTCCCACTTCGGAACAGGAAGACCTACCAACTTCAGGGCTGCA
GTAGTGAGCGGCTAGGGCCAGCAGGATGCTGAAAATGATTTGCAAAATGA
TCCAAAAGTCCAGACTTCTTCCTCTTTAGGGAAGCTTCTCTCCAGTTAATT
TGCTGCTTTCTTCCTTAGATCTATGCCCCAAAACCGCTGTGAATCAGACAT
CAGAAATGTAGGGCAAGAGGAGAGGCCTATTTCAAGAACCTTCCCTGTCA

ATCCAGTGTTGTCATTTCTGCCTAGATTAAGAGTCCCTTTTGAGCACCAAC
TGCCCTCACACCAAGTACTACACTCTGAAGGGTCCTGACCATTGCAGACA
GCCAAGCCTAGCCTCCCTAGGAATGCAGCTCTCTCTCCCTGACCACCCGAA
CTCCACAAGGGTTCCATCACTACCTCCAACTTCTCTGAACAACTTTTTCAT
TCTTTATTGTAAATCTCAGCCAACAAATCACTTCCTCCTGGAAGGTTTGGG
TGCTTCCTGCTCCCCTGGGTCAGAGGACAGTCCTTTGATCCCATGGCATCC
ACTGTACTGTGAGATATACTATCAGTGCTGCTGTTTAATCAGCCTCCCTAG
TAGCCTCATAGCAAGAATTACATTTTGTATTAGAACTCCGGTAACACATCT
ATATAAAAACAAATACGTTTCGTGAAACCCTTATTTTGTGAGTATCCTATT
CTAGGGTGCTAGAGGTTTAGTTCAGTGGTTAGAAGGATGATCTAGCATAC
CAGAGGCCCTGAGTTCGATCCCCCAAACAGCCTGAAACCAGACAGTGATA
CAGGCTCAGACAATCCAAAGGAGGAAGCCTGAGTTTCAGGCCTTCAGGCT
TATCCTTGGCCTCAACCATCTGGGTTCCAGTCTCAAAAATCTTTGTTCTAA
CTCATGTGAAACAAAGTGTAGCTGTATCTTTATACCTTACTATGGTGATTT
GAAAATCCTTGAGTGGAGTGGGATTCTGGGGTGGGAAAGAAAATACTCTG
AACAGTGAGGCTATGAAAGGAGTAAAGGCTTGACAGACCTTTGTCTCAGT
TATTAATACGTTCTAACTTTCATTTGGTCCTCAAGATTTCTGTGAGGAAAC
TATTTCCTACCCATTTGCACGGGAAGAGGTCAAGGATGGCGATATTAAGT
AATTTGCCCAATGTCACAAACTTGTAAAGTAGGCGCTGAGCCAGGACTTG
ACACTGGGTAGTTTGGGTTTAGAATTTCTATTCTAATTCTCTCATCTACAA
GGTCAGTAGATCTTGGCCCCATGTGAGTCAACTTACTCGGGGGGGGGGGG
GGGGGATGTCAGAAAGGGTCTCCTTGCAGAATCCTCAGGCTAGCTCTGTC
TCCTACTTGGAATCCCTGTGTGAACAGTATTTATGTCTGTGCTTGGCCTCTT
AGTTGTAAAAGGAAGAGTATAGGGGGGCTGGAGAGATGTGTTTCCTTTTC
AGCATCCACATGGTTGCTCACAGTATCTTAAACTCCAGACACCCACTTCTG
GCCTTCAAGAGCATGTTATGTCTGAGGTACACATTTATATGTGCAGGCAA
AACATTTATACAAATAAACAAATAAAAAAAATGTTGATGGGTGGGGCTGG
AAAAATGGCTCAGTGGTTAAGAGCTCTGGGTGTTCTTCTGGAGGTCCTGA
GTTCAATTTCCAGCACCCACATAGTAGCTCAGGGTCATTTATAACGGGGTC
TGATGCCCTCTTCTGTCATGAAGGCATGCATGCAAATAGAGCACTTGTATA
CATAATAAAGTAATCTTTTTTTTCCTTAAATGCTGCCAGGTAGTGATCCCA
GCAGGCAGATCTCTGAGTTTGGGGCCATCCTGGTCTATGGAGCAAGTTCC
AGGACAGCCAGAGCTACACAGAGAAACCCTGCCTTGAAAAACAAACAAA
CACGTTTTAAAGGAAAAGTATAGACATTTCAGGAAAGCAAATATGTTGCT
GCCGCTAAGGCTAATGTGTCAAGTCCCCCTCCCCCCCCCGCCCCAGGCAA
AATCCCTTTAAGGTCGGCTTGGGTATTTATTTGTCTCAGGGAGGAAACTGG
ATCCACAATAAAACAGTGGCTGAGATGGCCATGTCCCTCAGGGCTAGTGC
TGTCTCCGGGATTTTGATTTGTGAAGGCAGTGTCTGAGAAAGCACCTGCTT
GAAATCCATGTGTTTTAATGGGTGTGACTGGGGAAGGATTATTTGCCAAA
GATTCTCTAAGCTAGGTCTGAACTAATTAGCCCTTACACTGAACTTCCTTT
ACTTTGTGACTGGTGTACTGACAGGGAGTTGGCTGGCGCCCGCGGTTTATT
CCATCTTTATGGTGTGAAGTCCATGCAAGTCACACACTGGACACAGATGTT
CTGCTGCTTCATCACCTGTGTGTAGCTCTGGGGACATTGAACAGCTCTGTA
GGACGAAGTGTCCTAGATGTAGAACATCTGGGCCTTGTTTCCAGCTCAGTC

TCTAAACAACTTTATGACCACAGTGTGCCGATGAAAGGGGGTGGACTGTC
TGTCGAAGGCATTTGGCAAGTAGCAGGGGTTGGTTAAATGTCTGGAAGAA
AACCAGCTTCTTCCAGTCTAGAGACCCTATGGATTTCATCCTAGAAAAAT
TGGAAGTGGGATGCAGAAATGTCTCTCAGTCTTTCAATCACTTCCCTTATC
AAAAGAAGCCATGTTTCTGCCCCAGCCCTACCCCTTTGCCTCTCATTGTGA
CAGGCAGTAGGCTGGAGAGCTGGCCCTAAAGTCATGACAGCAGCAGGAG
AGCTGGCCCCCAGGAGAGTGGGCCTTGCACCTCACCCATGCAGCACAGGT
GAGCTGGCCATGAGAACACAGAGAGCAGATTGCTGACTGGGTCCTTGCTG
GCTGCAGCATTAGGTGAATCAGCCCCTGTACAGCGGTCAAGTTGACCAAC
TCAGCTACCTCCCAGCTCAGATCCAGGGCTCTGAGTTGACCTACCCCAACA
TCTACCCCCACGTATGATCTGCTGGAGCACCAAAAGGGTCAGTTGTGAAG
ATCCAAAGCTGCAGGATCTCCATGACACTGGGCAACGACAGGTTATCTAA
GAGGAGCCCCAGTGAAAATGCAGTATTGATGATATAGCAGAAGTCAGAG
GCCTCCAAACAAGCCAGTGACTCATTGCAATGAACATTTGCAAGTAAAGA
TGTGTGGACAAAAGGGTATACAGTGTGACACACCACGGCTTCTACAACAA
GAATTTTTTTTTATTCTATTTATTTTCCTTGTTGGGGGGATTGCAAGAGC
AAAGGATGATTTGAAGGGAACAGGGAGATGAGTGGGATTGGGGTACAAT
ATGTGAAATTCACA
>LNC_005933
AAATGATCATCCCAATGGCGGGTATTACCAAGGAGGATGCAAAGTAGAGT
GCAAGGAGTTCAGGGGAAAAATAGTCCTTGTTATTTCTTTTCCTTTCACA
TCAAGTGTTAAACTTCGCAACTGCGAGCCGACTTCGGTTTTCGATTCACAC
TCGTATACTCCGGCATCCTGCAGCTGTGCCTTGCGGATGGTGTACGAGCCA
TTAACAGACTTTAGCACCATGTCTCCTGTCTTGGCTTTCTTTTTCAGAATTA
TCCATATTTCGGGCACACTTCCACAAGTACAGGAGATAATGACAGTGTCT
CCCTCTTTGACGCTCTTAGATGGGAAGACTGTAAGCTGTATGTCCTTCGAA
GAGCCTTGGATAATCAGTTCAACTGATTTTTTGCTAATTCCAGCCTCATTA
ATCCCTTCACATACATAAATGCCGGAATCTTCCATTTTTGTAGCCATGAAG
GAGAGTGTTGTATTTTGAGAAAGAGGCTGCAGTTCCCCGTTCTTTAGCTGT
CTGCTCCACAGGATTTTGGGAGTTGGAAAACCATCACTTGAGCAGGTCAG
GTTCACAGGACTGCCCTCCTCTGGGACAGGAGAGGGGCTAACCCAGATGG
TGGGTTCTTTCGGAGCAACGTTGACATAAAGAGTCTGTGTACTCTGCCTTT
GTTTGGGTTCAGACTCCATTTGACTGCTGTGTAACTTAGCGAGGCAAACAA
GAGCTTTCCCGGTGTCTTCAGCGGTGGGGATGAAGGTCATTTCCAAACTTT
TGGTCTCTAGAGACTTTGTGCCAATTTCCTCCCTTAAAAATTTATTCAACA
GTGTTGTCTCCCCCTTCAGTAGTTCAATCTCCAGATGGTCAAAGGGGTACA
CATTAGGGACCGTGCAGTTGACAGTGACAGGTCTCCCGTGTACAAGTGGT
CCACTTATTTCAATTTCTGGGTCTTCAGGGAATGAGTAGACCTCCACTTGG
ATTGTCTTCTCCAGTTTCCTTCGCTGACAGGTCACAGTGCACAGGTAAGAG
TGCTCATCCTCAACACCCACAGGGCTCAGCGTCAGTGTGGATGTAGCCCCT
TCATCCCTCACCTCCCCATTGAGGGGACTGTCTGTCTGGGTTCTCCAAGAA
AAGGAGGGAGAGTCACAGCCAACAGCAGCACATGTCAGAACAACGGAAT
CCCCAACCTGTGCAGCCACCTGAGATCCAGGGGAGATGTCAACAGTAAAT
GGTTTCTCTTGAACAATTAATTCCACTTCTGTTTTGTCTCTCCCAACCAGAT

TCACTCCTTCACACACATAGATTCCAGAATCTTCCATCCTCATAGCAATTA
AGGTGAGGGTGGCATTTCCCGAGAGGAGCTGTAGAACTCCATTATCTAAT
TTCTTGCTCCAGAAAATCTCAGGAGCTGGTAGACCCTCGCTGGCACATGTC
ATCGTCACAGCAGCACCCTCATGTAGCCTTGTGGAGGGATGTACAGAGAT
CTCTGTATTCTTGGGGGAGGTGTAGACTTGTAGTTCTCTGACAGTCTCCCT
TTCTTTGGGAATAGAATCAGTTTGGTCAATGTATAATTTAGCTCGGCAAAC
AAGAGCCTTTTCAATATCCTCAATGACAGGAGTAAAGATGACTTCCAAAC
TCTTGGTTTCCAGAGACTTCTTTGCCATCTCGTCTACAAAATCCTGTTTCTT
CATGAGACGGTCACCCTTGAACAGTTCTATCTCCAGTCTGTCAATTGGGTA
AACATCAGGAGCCAAACACTTGACCATGACCGGCTTCCCAACCTCCAGGG
GGCCACTGAATTGAATCTCTGGATCCTTAGGGAATGAGTAGATGTCCACTT
GGATTCCTCTTTCCAGTTTCCCAGAGTTACATGTTGCTGTGCACAGGTAAG
AGTGTTCATTCTCAAAGCTGACAGGGTCCATGGTCAGAACGGACTTGGCC
CCCTCTGTCTTCACCTTCCCATTTAGTGGACTGTCTATCTGGGTTCTCCAAG
AGAATGATGGCGACTCACAGCCTGTGGTGCTGCAAGTCAGGAGCATGGAG
TCTCCAATCTGAGCGAGCGTTTTGTATTCAGGGGAGATCTCGATTTTAAA
>LNC_006145
CGTGACTACTGCCAATGGCAACGCAAAGTCGAGAAAGCGCTGGGAGCCC
AGAGCCTGTACCTGATGCTGCTCATGCTTCCTGTCTCGGAGCCAAGCTTGC
ATCTCTCCAGCTGGCTGGCCACGATCTGGCGTTCAGCCTCCAGCTCTCGGG
TCAGCCTTTCAAACTGTAATTCCTGTTCTTTGACGGAGGCAAGGATAGCAG
AGGTTGTTTCTGTCTCAGAGCCATCCCCATTGGAGGTGTTTAAGCCTGGGC
TCAAGGAGCTGTTCTTCTCAGAGGCTGACGGAGGCTGGTCTGGGACAGGC
ATAGCTCCGAACGGCGCGGCGCCCGACTGCTTCCTGGCGAACATGCACCC
GCCGCCAAAGACAGCTCCTCAGTCCGGGGAAAGGCGTGCGCGCCGCCGCC
CGGCTTCAGAGCAAGGTCCTGACCTTGCCCAGCTGCAGCATCCTCCGCTTT
TGTTGTCGGAGCGCGGCCGCGGGACAAGTGATGCTGGCGGGGAGCAGGG
GCGAGCGCGGCAGGCGAGAGGCGGCTCCCGACGCGAGTGCGCAGCGCCC
GGCTCGGCCGCTCCTCCGAGCGCGGCGAGCGTAGCGCCCCCTGCCCGACT
TGGCGGGCGCCGCGGGATGCTCGGGGTTCCGCGGGGCTCCCCGATCTCGG
GCCGGGCTACCGAGCTGCCGCGCCCGCCGCTCCGCTCGGCCGATTGCCGC
CGCGGGCGCGAGCCTGGGCCGCCCTGGCGCCCGGCGCCGAGCGCTCTCGA
GCTGCGCCCTGC
>LNC_006189
TGTGTGTGTGTGTTGGTGCGGTGTTTGTTGTTTGGTTGGTTTAAACTTAGGT
CTCTACTATATAACTGGTTGGCTATGAAGACCAGGTAGAGCTCAAACTAG
ACAATCCTCCTGTCTCAGCCACACTCTGCCCTCACACGCCATCAGTACTGG
GTCTAATTCAGAATGTTAGAGGGGAAAATAAAAATAAAATGGGAGGAGA
ACGAGAAATTCCCATTACACCGATGGAACTGTCCCCACAGTAATTTTGCC
ACTTTAGTCCTCTTAAGGTGGTCATCATCAAGAGAAAACCAGTGTCCATGT
GGTTTGAATAGCAATCCTTTTGGAATATAATTTTGAACACAAGTCAGCTCC
AAAATTGGTTGCACAAAAACTACTTCCAGCCAAAACCCAAAGATGCAGCC
GTTCCCTGTACATCAAACTGCTAACGCCGTTGGTCTTTTGAAATTGTTTATT
GAGTATTATACAGTGAGACAGACCCAGTTACATAATGTCTTACCATGCCC

ATGGCGCTGCATGAAATGAAAAACTCTAATATTTCAGTCCAGACAGAGGA
GGAGGTGAAATTACAGCATGGGGCTCAGTTTTCCTGTAACCTGGAGCTGG
TCAGCCCTCCCTAAATGCATTCTTCTCCTTTGAGCACATCTTTCCTAACGGT
CGAAGAGCCAGGCAGCAGTTGCTCTTTCCTCGTTGACAGAAATAATTGAT
GTCTTTTGAATCCTATGATTCTGGCAGATTTTTGCAATCATATGCACAGCC
TTCTGAGCAAACAGAGTCTTGCCCAAGAGGGGTTTGGCAAGACTATAACA
AAGAAGTATCTGAAGAGCTGCACAAGCCTCCTCCCTCCCTTCCCCGGGCTT
ATTAGTTTCAGATGATTTGATAAGCATGTGCCTAGGAGTAACTGCGCTCTT
CCAAGCTTGTCTTTAGACGGTGCGAGGCCTTTGGTTGAGAAGCACCCGGC
TGTACCAATTACATCCTGGTTTCTCAACAGTCATCGTTGAAACATTTGGCA
TTAACACATAACAATAGGTCCTTCTGTTAACTGTAACTATAAATAAGACA
AGTATTGTACAAAGACCGGAATTCTGCCCCCATGAGGTAGAAAAGAGAAA
AGGCTACCACAGGTCACCCCACAGGGAAGCTGTAGCAAACATATATCTTA
AACATACAAGAATTCCACACGTAGATCACTGTTACATCCCTCAGAGATTTT
GTGATGGCTATTTCTTCTCAGGGGTGTCAAGAAAAACAAATGACCAAGAA
GAGGATTTCCTCCCGTTTGAGCCAGCCTGGCCTACAGAGGGAGACACTGG
GGTGGGGGCGGGGCAGACTAAGAAACCCTGTCTCACGAGTGTGGTGTCTA
ATCTTCATTGTCAACCGGATTAGGTTTAGACTCACCATGAAAAACCACCTT
TCGGGTGTCTGTGAGGATATTTCTAGGAAGGTCTTACTACGGTGCAGAGA
CCCGCTGTGAACGTGGGTGGCAACATTCCATGGGCTGCGGCTGCTTCTGA
GGGGAAATGCAAAGCCATTACCTCTCAGCTCCCTGATTTCAGAGCCACTG
TGACTGCCTGCCCCATGCTTCTGCCACAAGTCTTTCCCACCAGGATGGACA
GTATACTTCACACTGTGGCCCGAGTGAACTCTTCCTCTCTTCAGCTGCTCC
TCGGCTAGTGACAGCAATGAGAAAAATAGCTAACCTATAAATTCATCTCC
AATGTGCAGTTTAGTCACATGAGATTTTTTTCTAAGCATGTTAAGTGTTTC
ATCTTTTCTTCTAAATTTAAAAGTAATATAGGTTCATTTTATAATGTCTGCA
TTTTTATTTTTGCAAAAATGAAAAGTTTTAGAGTCCCTTGGAGGAACAATT
TTAATGAAAAAACACTCTTTTTTTAATGTATAGAAAGACTTGTTTCCAAGT
TGAGTGTATCACCTCAATTCATGGAGAAAAAGCTAAAATAGTTTGTTGAT
ATAAAATTTGGGCGGATGATTTACTTACAGTGTACAGGGGCTAACTCTGC
ATAAAATAGCTACAGATGGAGACTAGGAAGTAAACGGTCTATCTCAGGCA
ATGAACCCTGTCTTAGCCGAATTTAATCGCTGTGAAGAGACACCATGACC
ATGGCCGCTCCTATGAAGAGAAGCATTAAATTGGGGATGCCATACTGTTC
AGAGGTTTAGTTCACTATCCTCCTGTGGGGGGTATGGGAGCTTGCTGGCTG
ACCTGGTGCTGGAGAAGGAGCTGAGGGTCTACATCCAGACCCACAGGCAG
CAGGAAAAGAGAGTGACACAGGGCCTGGCTTGAGCTTCTGAAACCTCCCT
GCCCACTCCCAGTGACACATTTCCTCCAATAAGGCTACACTTACTCCAACA
AGACCACAAGTCCTAATCCTTTAAACAGTGCTGTGCCCTAGAAATCTGTG
GGGGACCATTCAAATTCAAATCACCTCAAGCTTACAGAACAGAGACCCAT
CAAGCACCTGTTTCATAGTTCTCATTCAATTTGGAATTGGCCTTGCCTCTG
AAATATACACAGAGGAGTAGGATATGGAGAGACTCAGAAGGTTCAGAGT
CTGTTAAGAAGTCAGAGCCAGAAATGGTGACCCACACCTTTGATCTCAGA
ACTGGGGAGGCAGAGGCAAGAATATCTCTTGAGTTCAAGACCAGTCTGGT
CTACATAGTGAGTTCCAGGACGACAAGCCCTACTTAGAGAGACTCACCTT

AAAGAAAGAAAAGAAGAGGAAAAGGAAGAGGAGAAAGAAAGAGGAGG
AGGAAAAGGAGGAAGAGGAGGAGAAGAGGAGGAAGAGGAATAGGAGGA
AGGGGAGGAGGAAGAAGAGGAGGAAAAGGAAGAGAAGAAGTTTTTCTAT
AGCTATGGTCTTGGCAAACTTTGCTTTGGCAAAGTTTCTACAAATTCAAAG
AGAAAAGATCAGGGGCAGGAGACTGATTCTTTTTAGAGTCAATTTCCAAA
TATCATTTCTTAATTGGGGCCTGGCTACATTGAATAAGAAAATTTAAATAA
CTTACTCAGAGTAAAAAAAAAAACAAAAAACAAACCAAAAACCTTCCTTT
TCCAACAGACCCCAACAGCAAACATGTCATCCCTTCCTGTTGAATAACCA
GAGGAAAGGGGGTGAGGAAAGGAGATCAGAAGAGACCAGACTGGGTTGA
GGTAGATTTCAGGCCACATAGCATAGTTCCCCACTGGAAGGCAGCTTGAC
AGGTCACTGGAGAAAGAGGATCTGGTAGACATGTGACTTCCTCTTCCTTCT
GAATAGTAAACCAATCAAGAAAGAAAATGGCTGGGCTGGAGTCAGAGGC
TAGAGTAAGGTCTGGGCACATTCCTAGAAGTGAGGAATGTCAGCTAACAT
AGACTGACATAGAAAGCTCTGGACAGCAGAGTAGTATGTTTGGCCTTTTG
TTTTAGAAGGACCATACAGCTAGCCATGGTAGCACATGCATATCACAGTA
GCACCTAGAAACCTGAAGCTGTAAGAGTGCCACAATTTTAAAGCCAGCAT
GGGTCATATTATGAGTTCCATGCCAGTCTGGAATACAAAATGAGACCAGA
TAGATAGATAGATAGATAGATAGATAGATAGATAGATAGATAATTGATAG
ATAGATGATAGATAATTGATAAATGATAGATGATAGACAGATTGATAGAT
GATAAACAGACAGAGAGATGATAGATGATAGATAGATAATTGATAGGTA
GATGATAGACAGATAAATAGATGATAGACAGATTGATAGATGATAGATA
AGTGATAGATAGATGATAAACAAATAGATGATAGATAGATAGAATATAA
ATGATAGATAGATGGTAGATAGATGGATAGATAATTTTTTGAGAAAAAAA
AAAGACTGCTATACAAAAAACTTGTATGGGTGCAAAGTGAAACCCTGAAA
TCAGAAGAATGGGGTCCAAGCAAGGATGGACTCCAAGCATGGGAGCATT
CAGGTTCAGCATGTGTTTTAGAGTAAGCACGTCCAAGGAGCACCAGATGA
GCCTTATTCATCAGGGGTGTCAGCCCTTAACCAAACAGATGGGTGAGGAA
AGACATCAGTGGACCTCCTTAGTTACATTACTCTTCTTGGAAAGACATCAG
TGGACCTCATTAGTCACATTTCTCTTCTTGTTTCTTTTTTGTTTCGTTTTGAC
TTGGTTTCTTATGTACCCCTTGTTGACTTTAAATTAACTGTGTAGCTTAGTC
CTTCTGCCTACACTTCCCCAAGCATGGATCATAGGACTGTGCCACCGTGCA
TGTATTTTTGTGGTGCTGGGGACTGAACCCTGGGGACTGAACCCAGGGCTT
CATGCAAGTTAGACACAGGCTCTGCCAAGTAAGCTACTCCCCAGTCCCTA
GATTTGTAAACTCCAGCTAAGCAGTGCTCTGTCTTGGAAGTGTAAACCTGA
GCTTCTCGGTCCTACCAGCATTAGACCAACACGTGGGTCAAATTGTACTCC
CATGTCAAAGTTAAAGACCATTTTTTCTTTTTTTTCTTTTTTTTCTTTTTTCT
TTTTTTCGGAGCTGGGGACCGAACCCAGGGCCTTCCGCTTGCTAGGCAAG
CGCTCTACCACTGAGCTAAATCCCCAACCCCCATTTTTTCTTTTAAAATTTG
TGTTTACTTTCTACATATTATGTCTCAGTAAATTCTCCAACCCAAATAAGC
CCGTGCAAAGAAAACACAACTCAGTTAATATGAATAGAAGCTGCGTGCCT
AGATTGGGCAGATCTACCACTACGCTACTCTATTCTTCAGCTATGAGATTC
CCCTATAATTTGCAGGGTATCTCCAGGCCATTCCCCTTCCACCTCCTCCTCT
GTCATCCCCTTCTCCCTTCCTCTCTCCCCAACTCTTCAGCTCTACCTTCCCT
TTCCCTGCCCAATCACTGGCTCTTGCCTCTATTTTATAAGTTGAAATGGGG

AGAAGGTCCCAATGAAGTCACCTGAGTATGTGTTTCACTCCTCGTCCTAGG
CAACTCCTCTTGGGGAAGAGTAATTAGCATCGAAATACAAACAGCACCAG
GGCACCCCACAATACTGTATTACTTATTGTGCAATACCTGCCTCAGTTTGG
ATGTGTGAAGGTCAGAAGACGTCACTCTAGAGCTGCTTCTTGCTACCTCAG
ATCTAGTGATGAAATTCAGGCCGTCAAGCCTGTGCACAAAAACCTCTACC
AGTGTTTGTCTGACAGCCTCTGTGGTTGTTTCAATATAAACAGCCCCCGCA
GGCTCGTAGGGAATTGTACTATGAGGAGGTGTGGCCTTGTTGGAGTAGGC
GTGGCCTTATTGGAGGAATGTCATTGGGAACAAGCTTTGAGGTTTCAGAT
GCTCAAGCCAGGCTGGTTTAATCTCACTCCTTTCCTACTGCCTGCTGATCC
AGATGTAGAACTCTCCGCTCCTTCTCCAGCGCCATGCCTGCCTACACAGCG
CCATGCTTCCTGCTGTGAAGATAATGGACTGATCTCTGAACGATGCACACC
TTGAGCTTGTGAGGTGGTCTGTGTCGCTTTCCTCCACAACCCAGCTGAACA
ACAGCACCTCTCAGTCACTTCTCTCCTCACCCGAGCTCATGGTACTCCAGA
GAATCAGGACTTCCTGGAAAGAACAAAACGGAAGAGAAAAATTGTGTGT
GTGTGTGTGTGTGTGTGTGTGTGTGTCCGTACGCACGTGTGTGCATGTGTG
TGTGCACGTGTGTGTTATTTAACTAACATTTTGACACTGAACTATAGACCT
TACCCATAGAAGCAGCAGGATCAGT
>LNC_006205
GTTAGGATCTCTTTATTTCCAAATTGTAATATATTCACATTCAAACTAAGG
GGAAGGGGAAGGGTTCATGTGATTGCCTGTTTCCCAGGGACTCTGAGAAA
GTCTCAACTCCTCAGAGATTCCAGAAAGTAACTGTCCTTTAACAACAGGG
TGGTGACCCGAGACCTGAGGACAAGTGAACTTGATCCCACAAAGTCCTAC
TGTCCCTCTAACAAGTTGATGAGTCCATTGGCGAGAGAGTCAAGATGCTC
CTGTCATTGGTATAGAACGGATCCGACGCACTCCACAGCCTGCTGATATG
GTTGGGACTACACCGTCCTATGACTCGGTCCAGCACTCTTCCAACAGATGC
GCACGGGGTCTCTCCAGGGAAACAGCATATGGAGTCCAAGCACTTGACAC
CTGAAGGGGCTTCAGCAAGCTGTGCCTTTTGTGTACTTGTAGGATGAAGCT
GTCGATCAAACACTTTCTTCACAACATCAACAAAGAGACAAGAAACAACC
ATAAGGAGTATGGCAAACCAAGCTGACCCACTTGACAGGAGCTGAATGA
AGACAGAGTACATATTCTGGGAGCCCAAAAATGGCCAGAGAATCCCACCA
TAAAACAAAGAGAATATAAAGTAAAATATAATAGATCCCCA
>LNC_006223
ACTTGGTGCAATAGTTGCATGCGGCCGGCATGGAGGTATTGGGTAGGGGA
CAGGTTTGTGTGGATTGTATGTTGAAGGAGGTGGCTTGGATGGCCCGAGA
ATCCGCGCTGCTATTCCTTTGCCTTCATTGGTAATTTCTTCACCATCCTTCT
TCAGAGGAGTTCCAGGGAAAATCCCATTTATACGACTGGGTTTTCCTTTGG
GATAGGGGTTCCCCGGGATGGTTCCACATGAAGAAATCATGGCGTGAGGG
GCTTTGCAGCCTGCCTTGGAAGCCTGCTCCAGGACTCTCTTGCACCGTTTC
TTCTCAGACATGTTGATTCTGAATAAATCTTCAATGGGACGAGAGTTCTGT
GCATCCACAATGTTTGAGGCCAAGAGCTCCAGAGCAGCCAGCTTGTCCTT
ACTAAAGGTGAAACAGCTCAGGATGCTCACCACTTCTGCTGGGTGGACGG
CCACCATTTTGTGCTGTAGAGCTTTCATGGCTTTCAGCTGTGGCTCCGCCC
AGGAGAAGTACCTCAGGAGATCAACCACCTGTTCACTGGAGAAGTACCCA
TGAACATATTCAATTGCTTTTAATTTATATTCTGTCAAAACAGCCTTTCGA

ATTTCATCCAGCACAGTTTCAAAGGATTTTTTGTCCATCTTGAGTACTATTC
CGACGTGACGTCTAACAGTTCTACTTGCAATTACGAAACGCTCATCCCAG
GTTAGACTCGTGGATTATAATATACACCAGAAGGAGAAAGGAGGCGAGG
TAGAGAGTATTGCAAACTGCACCAAAACAAAAACAAAAACAAAAATCCC
AGAACGGAAACTTGAAAAAAGTTTTATTAAAATCAGACTTCAGTAGCTT
CTTCGCACAAAGCTTCAGCTGCTCAGAGCAGAGCCGGCTTGTGCGTTTGC
AGAAATCAGGGCTCTGGAGGGGGCCCAGGGCCGGGCACTCCCGGGAAGG
CCGCCGAGACACGCTCCGCTCACCGCAGCGATCCGGGACACGTGGCCCCA
CCGGCGCTCAGCCCAGCCGCTCCGCCGGATCTCGGGGTCCCGCAGCTCCG
GCAGCCGGCTGCCCCGCAGGTCCTTTGTTACCGGTTTTCGTAGCTTCCGGG
AGCGGGCCGGCGGCCGGGGCGCAATAGGAGCCGGGCGAAGGGGGATACA
GCCTCACGAAGGGCGGCTCTCGAGCACCGCAGCGCTCACGCGGCCAGACG
CCCAGCGGGAGCACCCCTAAATGCCCCACCAGCTCCTGGGGACCTTCGGT
AGTGCTTGTCG
>LNC_006414
CTTTATGATGTTTGTTTCTTTTAAATCGTACCACTGGCTGCTTAGGACCGTC
ATGTGCACTTGTGTCCAATTTTTCTGGATAGGCACACACTCTCAGAGTTTT
TGAATTTGAACCAACGGCATATAAACTACCACTTGGGTGAAACGCCACTG
CTCTAACAGCCTGTGTGTCTTCTAGAGTATTAATACAAACAAACTGCTTCT
TTGACTTGTCATCCTCTTCCCCTTTTGACCTTGATAGACTCCCTGATGAATC
TCCAGGTGATGGCTTAACAACAGAATGTTCTGATGAGTTCTGGCTTCCACA
AGGTGATTCTTCAAGGAAAGGGATGTGATTTGTTGATCCAGGATTACGAG
GAGTACTTGTGTGAATATTAGAAGTATCATGTGTTAACCTCTGACTGGAAT
CTTGGGGTGGTGAACTGAAACTAGTGACAGAGGAATTATTTGACCCACTG
TTTTTGCTCCCGAGGCATTGTTGGTTGAGTACTGGCACCTCATTGCCAAGA
CTGTCCATT
>LNC_006418
GGATGTCCAGAGGACTTGTGGCTGCCCCTGTAGTAATCTCTCAGGATCAGT
CGTTCGCATCCCGTGGCTCTCGGTAGCCATCAAGAGCCCACAGAAGGCAG
AGTTTTGCTTTTCACTTGTGAAGGAACGAGGAGGCCCCGTCAAGGTAGAA
AAGGAAACCAAGCATCAGCCCCTTTATGGGATGAACTCACATGGACATAA
TCTACGCTGGCCTTGACTCATTCGCGATGTGCTCCGGTTTCCAGAAGAATC
CCACACCATAGGTCTCCCCAAAGTTCCGCGGAATCACCAGGAGAGGGCGG
CCGCGGACCTGCCTTTTGCCTTCCCAAACTGCAGAGCCCAATGCGAGGGT
AAACAGCAGCCACAGCAAGTTCTATCCACGGCCCAAGTAAATCACAAATA
TATAAAAAGAGACTTCAAAAGGCAGTGGTATCAAAGCGGCAGTTAAAGA
GAAGGAGACATCCAAAGTCACAGCTGCGTCGACGGAGGCTGCTTCAGCAG
CTAGGATTCGGAAGCATTTAGGGATGAGTTTTTTTCCCCCCCACCCCCAAA
GTATTTCTCCCAGTCCTTTTCCTTTGATATACATGAGCCTCTCGGCTTTCCT
TTGTTCTTTTTCCACGCGTCAGGTTTTCATAAAGTTTTTCGAGTTGATGAGC
TTTCCCCGGAAAAGACGTTAGATTCGCAGCACTAGCAGCTGCGGATTTGG
ATCTGCAATGCTCTGCAGCCTTAACCTGGTTCAGCTCCAAGAGCAAGCAG
AGAGTGTCCCTCCACCAGCTTCTTCCACAGGACCCCACCACCTTCCGCACA
GAAAATATTTGGATCTCTGGTGTGACCCGTGTGCCCTGTCCTTTCTCCTCT

ACTCTGGAGCTGTCACTGTTTCAGGCTCGGTTCCTGCACCAGGGGACTTTT
ATAAGAGTCAAATCACAGTGAGGGCAGATCTTGATAAGTCATGATGGGGC
AGTGGGTGCAAGGAAGACTGAAGGACACCTCCCTCTCTTCTCTCTGTTTGG
TCCATATATCCCGCCTTTATAAGGAGGCAACCAAGGTCAGGAAATAGGGG
CCCAGATAAGGAGTACAGATGTCTTGAAGGGTTGCCTTTGTCTTAACTGTC
CCCGGAACAAAGCGTGTAACTCCTGAAAAGAATGACGCGTCTTACAGTGT
GTGCTCACGAAATGCACTTTAGGGAATCCTAAGTAATTATTTCCCGCACAC
TTCAGGCCAACACAAGTTAAAAGGTTCTCCCTTCTGACAAGTGTAATTAA
AACAACAACAGCAGCAGCAGTGGGGCTTCATTTGTTTAAATTTGTTTAAA
ATTTTTTAATTAAGGTTTTTGACAGGACATTTATATCATTTTTCTCTAAGGT
TCAGGGAACACCCAGGAAGAAGGAACAGAAAGCATTCAGGGCGGGAAGA
TGAGGGAGGGGAGGGGCTGTGAATCACTCTTCAGGGCAGAAGAGCGCCA
CTGCAAAGGTGCAGGCGGCTGCAAGCCTGGGATATGCACAGCACTGTGCT
TATTAACAGCCAACCATGCTCAGGGAAAGGCTCATGGTGACTTGCTGTCT
CTGCCAACTCCTACCTACTGGTGGATTCTGGGGAGGGAATAGTCATTGTCT
TCAGTACCCGAGGTGAGCCAACTGGGCTCCAGGGGATAGTTCTGCAGACG
CGCAGATAGCCCTGGTTAAAACCAGTGGTTCACAAAACAATACAAGTGAG
CTCGTAAGAGACTTGTAGGG
>LNC_006468
GCTCAAGATTTTGAAAGGTGAGTGAGTGTATGTGAGAGTGATGGATGTTG
GTAGAAAAGAGCTTTTCTCGGTTGTTCCAGATGTCGATTCGCAGTATTCAG
AACTACATAATAATTCAGTGAATGTAGCTGGCGGCAGTTTGATTTCATGA
AGCAGAGTTTGCCCTTTAGGCACAAATTCCTCTTTCCTCTCATCTGTGCTTC
TGAAAATTACATCTGTGATAAGTGTGGCTCAATGATGAATGCTTGGGGTTT
TCTATCGATGAAAGATAAGTGGGCTCCTGCAGGTTCGCCATGTGGTAGGA
TACGTTTTCATCTGCTTAGATCTTTTATGGGCCTGCAGACTCTTATCATAGC
AATAGTATGTTCATATCTAGAACACTCATATTAGAATTTTCTGATTTTTTCC
CTTTTATGTTCGACGCCGGCATTATGAGAAAGTAGTTTTCTACATGCCTGG
AGTATTGCTTAAATAAGATATTTAAATGTTACAATTTTTTGTTTGGTGCCA
AGCACAATATTAGCCTAGAGGAGTTCGCCCTTGAGGTCAGATACATCTGC
CATTTTGAATGGATGCTCCAGGGACCTGTCTTATATTTTGTTTCTCTCAGG
AACACCTTGTTAAATTTGGTGATCATGTCCAAAGTCAGCAATGCATGCTGA
GTTTTGTGAGCTCTTTGGCTATATATTAGGCTGCATATTTTAAAGCTGTCA
CTGTTCTCATTGAATCTTTTGGTGTTATATTGATTTATTGTGTTTCTCTGTTT
ATGTATCCATGTGAGTTACACATGGCTACCTGTGCTTTTAGCAGTCTTATA
AACACAGAGAAAACAGTGCCTTTAAACGGGGTGCCTAGGTGAAAATCCA
GTAGATAACTTAAGGAAGACGGATTCCCTCTAATTAACTTATGGTCTGGG
CCCTTTTAAATTAATTTTTTAAAAATGCAATTTAAGGCCATTTCATACTGCT
TTGAAAGCTTAAGGCTATTTTTTTTTCCTTTTGCCCTTGTTAGGACTGCAAG
TGTTACTTTTGCAAAATGTGTTTCTTTTCTCAAGATCAAAACTTATTTGTGT
ATAGTCTATTTTCTCAGAATTCTTAAGTTACAGCGACCATAATGTTGAATA
CATATGATCCAGACACAGTATGCACCACAGCTTTGAATACAGAGGAGACA
GTTACAGCTATTGTGAGCAGACCCGTTTGTTCATTCCTAGTGGGTCACTGA
TGAATTGATTTGGCTGTAAACCTGCAGAAACACATTAACATTTTAGTTAAA

TTCAACAAACACTGAGCATTTGCCATGATCAAATCCTTGGGCACCTCTGGG
TTCGAAATAAACTGTGGCACATTTCTGCTCCTGGTGGAGCCAACAGATTAC
TAATGTATAAGGCCATAGCAAAAATAAACAAGACATAACACAGGGACCG
TATAAAGCACAGTCTGTGTGAGCATTCGGAGATGTGCAGAGCCTGAGAGC
AAGAAGGAAAAATAAAATCATGAAGGAATCTGTGTCTGAGATTATGAAG
GAACCCAAGTCCAACATTATCGTTGAGAGAAAGGACTGAGATGGCCTGGA
TGTTTTTAAGGTGGTCCGAGGTTGGGAGGTGGGAGAGATAAACTCTGGAA
TAGCAGGCCTTGTATTTATACGTGAGGAAATGCTTTGGTCAACCTGGGATG
GACAGAGAGTATGCAGCACAGATATCTGGAAAGAAAAGGCACAGTGGGC
GGGCCATTGGCTGCGTAGAATCCATTATGCCCACCAGCTAATGGGATACG
AAGGGTCAGGGAGGCTGGAGTTCAAAGTTAACTCCAGGATTTGGAGGCTG
AATGTCTGGAACTTTAACAGAAATTAAGAAATCAGGAAGGGTACCTGTTT
TGGAGAGGAACACGGAGCAATTCAGTGGAAAAGGCATTGTGGGTGCTCTC
TTAACCACTGCTCTAGTAAGCACATCTCATTTGATGATTAAACATGTGCTC
AATACTAGTGTCTGTAAGTCTTCTAGTGATTATGGGAAGGTCACAGATTTC
TTGGGCACCCGTGTTTATTGTATGTAAAGACAAACTCAAGTAGCGAGTCCT
ACACCTTCATGCTCTAGGTTACTCTGTGTGTCATATTGTGAAATAAACTTA
CTTTCCTCTTCCCGACCATCTCTCCTTCTCTTCTTGCATTGCATGGTGGGTA
ACCTGTGACGGTGTTTTGCTATTTTCCATGATGTCTGAACAGGAGCCTAGA
CCTACTCAGAAACCTTTGAATACTTTCTGTCTCCTCCCTGGGGATTTGAGT
CTCGCGTTCCAGCTCCTTGGTATCTCTCAGGTGTGTCCACCACATTCTTGGT
TCCCAGCTAAGGCTGTGTAGGGCTCCCCCATTCTCACTGGAATCACTGTAA
TGTTTCCTAGCAGTGGTTCTTTGTTGGCCCAGATCACTGCTACTGTTTCAG
AGTTAACACGAGCACTCAGCACCTGTGTCGGAGTGGCTCCCTAAAACACA
GCATCCTCTCTGAAGTGTATGTGACACTTGAACATGTCACAGCACCAGGA
TCTGCTCTCTGTTCTGTCCCATAGTCCTCTGTTAACTCTAAGCATTGTCAGA
TGCTAGCTGAAGCTTCCATGGTAGCAGGAACCAATGTTTGCACCGTGAGT
CTCTCAGTTTTTTCTCCTTCTGTGGGCTACTCTAGCGAACTCTTAAGACCCT
CTATTGCTATAGTCTTTTTTCTCATTCTCGTAATACCTTTTTTTTATGGTTAT
GACTGTGACTTTCTCATTATATTTACTGAACTAGGAGTATTAACTATGAGT
AGTTAGTATGATGCTACTTATTCTGTAGGGATGTTGAATGAATTAATAAGT
AAACAAAGACCAATAGAGCCTAGATTTTATTCTCAGACAAACTCATTGCT
TAGTAAAAAGTTTTACAAGTAACAAATTTAAAAAGCCACCAATAGATGAT
TTCAATATAGTGTAATATAAGGCATTAAAACGTATGTTCCAGTAGGAAGT
AATTAAAGGCTGGTTAATATCAAAGCCTGTCTTTTGACTCTTTAAACTGAA
CCAACTTTTTCCTTTTTTCTTTTTTCCTTCTTTTTTTTAGTTTTATAGGGACC
TAAAAGAAAAGTCAGATGGGATGCATTGTGTTTCTGAACATCCATTTATC
AGGTTTATGAAATTTGGGGACTTTGTTATTTTTTTCTTTTCAGTTATGAAAT
TCTCAAAATTCATGAAATTTATGAAATTCAGTTATGGATTTCTCTATGTCT
AATAGTACTAATTTTTACCTCTTTAAGTTTTCTCTTTTATAACGAACTCGCT
AAAAGCTAATCACAAAAATATGTTTTCTTTAACCTTTAAAACAATTCAGGA
GGAAGACATTTAAAGAACTTGAACCTATAACTTTATGGCCTCTCAGCTATT
AAATACTATTGTAGGATATTTTGCTTCATAATCTAGAATCAAAAGAATGTT
GAAAATAATAGTGACTCCTGATACTAGAGTTACAAATTACAGCATGATAT

AGCTTTCATAGTCCCCATTAAGCGCTCACTCCGACTCAAGGATGGCCTCTA
TTGAACTGTTTCACATTTTTAAAAAATAAATATAATTGCAGCATTTCTCTTT
TCCCTTTCCTTCTTCCAAACCTTCCCATACACCTCTCCCACTTCTCTTTTAA
ATTCATGATCACTTTTTTACTAATTGCTATTACATGTATACATACATACATA
CAACACACACACACACACACACACACACTTTGCATATCACCTACTCAGTCTCT
ATAATGTTGCTTGAATGTATGTTCTCAGACCTGATTGGTTGTGTCCCTCAG
AGCACTGGGGAGCATGACTGAGAAGTTGAGGAGCAGGTAGGAAACCTCT
TGCTGAAGCAGTAGGCATATTTACTGCTCTGTTCCAACCTCGGATAGACAT
TAAAATTTTTTTTTCCTAATACAGGAATGGTTTTGAAATCTGTTGTTTTACT
TGATACATATAGTCAAGCAAAGCTACATTGTAGACAATGACGAAAACTTG
TGGGAACAGAAAGTGTCTCTTTCTTTTGTCATGGCTACTCTCCTTACCTCC
ACATCACATTAACTGTGGTGGCATCCTCAAAGACACTTGTGAGCTGAGCC
CCCATGCCTTTGGCAGGCTGTGTGTCCTGTGCTCCATCCCATTTGTTTTCCA
TCAACCCCGAGTCCCGCAGAATGGTACCCATCTAGGGACAGAATCATAAA
CCAGAACTAAGGAGGGGAAGAGGAGGGAGTGGATTGGGGGCTCTGAGGG
CAGAGATTTCACACTTGTTACTGTCAGGGGGAAGTTGGGGCTGTTGAGGG
TGTCATGGGTTTCACCTCACCGTGCCAATGACTGGCATGCCAAGATGTCTG
AGTCAGGTCGGGGTTTTATAGATTTTGGCCAAAAGGCAGTTTCAATTGAA
AAGTAAATGTTTGAAGGTGCTGACCATTGCCCTGGCATCCTCTTGCATTCT
TTAGGACCTAACTTTCTCTCCCACCCTTAAGTAGGCTTAGATCAGGGGCAA
AATTCAGGTCACTACCATAGTAACCACAGACACGGAGCACAAAGAAGAG
CTGTTTGCCCTGATTTCTTTAAGATAGAGGGGAAAGGGGAGAACAGGGAA
CAGAGAGGACAGTGGAGAAGCCTGTCGCTGGGAGACAGGCAGTGTAGCC
TTGACTCAAGAAGTGGTTTGTTTCTTAAAGTCCGATGTTACGATGTTATTT
GATCTAGGGATTTTACTGCATCTTTGAAACAGTTTAAGAAGTTGGTGCCAT
TGATTCAAAATGCTATTTTAAAACCGACTTTCAAAATGACATGTTTTTAGG
GCAACCATAACTGTCAATTTGAAGGATGGGATTCTGTACAAAAGAGCTAG
TAAAATACCCTTAGCCATATGTGAAACAGGTTTAGGGCAATTTGTCATAA
ATAAATGAATTTGTCAAACGCATGTACATGTACACGCATGCACATTTTATT
TATTTATTTATTTTGCCTCAGAGGGGGAAAATCCAGATTCTTAGATAATGA
CATATTGGTAATTTAATAGCACATCTTATGTATAAGAAGCTGATGCATTAA
TACTTTTGTTAGTAAGAAGCTATAGTTTTAAAAGAGATATTAATTATTCCT
GTATGCCTGAAGGGAAGAATATTTGTTTTCCTGGAGGTAATCAAGACTTG
CTGGACTTGGGTAAGTTTAGGAGGCTTAGCGAACAGTTGCTGTGAAAGCC
GTTCTATATTCTGTCACTGAGAACATAGGCAACCCTGTCTTCCATGATGGC
CGTCTCATTTCCTCCCTGCCTGTGCAAATGTTGGGTTTATAATGGGGATAG
CTCTACGTGTAGATAATGTTTGTGGCCACGGGATACTTTCTCTCTGTCTTTT
TGTCTGTTCACATTATACAGAAAAGTTTTCATATATATATATATATATATATG
TATATATATATATGTATATAATGTTAATGTTAAAGCTGGAATGTGCATAGT
GTGTAGTAAACTATTTTCATTTTTCTCATTTAACTGTTTATTTTTTAAGTGT
ATCTGGCATAATCCAGGTATTTTTAAAAGAATTTCTCAAAAAACAGAATT
ATGAACTAGAATATTTAGTAATTAAATAATATGCTTTTGAGAAATGTCTAC
AAGTCCATCTACTTGGCATCTCTGTCTCTTTCTCTCTCTGTCTCTCTCTCTCT
CCCTCCTCTCTCTTTCTCTGTGTCTCTGTCTCTCTCTCTCTGTCGCTGTCTCT

CTCTCTGTGTCTCTCTGTCTCTCTCTCTCTCCCTCTCCCTCCCTCCTCTCTCT
TTTGTTTAAAGATAAGGTTTTCTCTGTGTATATAGCCCTGACTGGCTGTCCT
GTCTCTGTAGACCAGGTTGGCTTCAAACTCAGCAATTGCCTGCCTCTGCCT
CCAGAATGCGGATTAAAGATGTGGGCCTCTATGGCCTGGCTCTGTCCCTCT
TCTTAGATGCCATTCCTGCCCCTTCCCTTGTTTCCCTGGCAAAGGCTTCACT
TATTATTTTTTCCTTCATAATTCTGTTTATAAGAATTATGCTATTCTCTTTCC
TCTGTACTTTCCCTTAAGTACTTTCTCTGTCTTCCCATAATTCCTACAATAC
AGCTAAACTTTCTCAATAAATGCCTCAATGTAGTCGATGCCTTGCTCTCCT
GCAGTTATTTCCAATCCCTTCATGAACAGAGACTGCCTTCTGGTTCTTTCTC
TATCCCTATCTCTCAGCAGAGTAACTCACGGAGAGTAAATCACAGTTGCA
TAAATAAAATGTCTCCTCTGTATCTTTAGCCTCGTGCCACTTATATCATGA
AGAACAGTTAGAAGTATGAGTTTCCGTTTTTGCAGCACTTCTAGTGTTAAA
TGTCTTTGAAATTCTATTCTTTTTTTTTATTTTTGTGTCTCTGATGGCTTTAA
AGAAAAAATAAACTCAATACATTATGCTTTAATGATCTTCAAGGTTAGGG
GCCTAAATTTGAGGAATATTAGATAAATTCAAGCGTTTGAGCCCCCAAAT
ACGTGGTTCAGGAGAAAGCTTGAGTGGTGATTTCTGCAGCCACCTTTTAGC
ATCCTTGAAGGTTAGGTGTGCAATCCTGCATGTTGGGTGGGTTTCAAGTGG
GATCGCTTTATTGTGCATTCTTATGCTGTGCGACTACTCTAGTGTGAAACA
GATACTTGATTCCAGAGAGATGCTGCCATTGTAAACAAGTTACTGTTTATT
CTGTTTTCTTTCTCGCTGCTGCTTTTAGAGACATCTGAGAACCATTGAGCC
CTCAGTTTTCCTATGTGAAAACTGGCTTTGAGCACGATCTCTCCTGTACCT
CTGGGTCAGTGATACTCGAAAAGCATTTCACTGAACCATAACCTTACTCA
GTGAAAGAGAAGGGAAGAATTTAAGAGTAAATAAGATGCCCACTGATGC
AAAGGTGCTGCGAGGTGTGATAGTCAGCAAACATTGCCTTTATTGACTTGT
GAAGTATGTATGGTATGTGGTTCTTTCAGATATTCCCTGCAGTAGCTTGTG
ACAGGTAAGTGATATGTGATAACTGTTCCCTTCCTCTCTGAATTTTCTTTTA
GGGGAGGTGGGTATGGGTGTGTAGGTGTATGTGTATAGGTGTTTTGTTAA
ACATACGATTTGGATCGTTTTAGTCATCTGTAACAACCACAAATACTTCAG
TCAAGTGTGTAATCTTCCCGCGTTAATGCTTCAGAGTTTGTTATATGAAAT
CATTCGCTCTCCTTAGCGCTAATTCACAGCAAATTATATCATCCTTTTCAG
GTTGATTTCATAGAGTGTTAAGTGCTATTTAAATCCTTAGCAGATATGAAG
CCCATTTATTTCGCCCTAATCACTTAAAATAGCATGAGCTTATCAAGAGTT
TTTTTTTCTCTCTATTTTATGTATATGCCAGTCTGTTCTTGACTCACAAGCA
TGCTTCTGCCTTTAGGTCAGTGGGCACTTGTCCCAGCCATGTTTTCTTTTTC
CCCCCTTTCCCTAGTTTCTCTCAGGAACAAATGGCAGCACAGTTAGGCATT
AGAAACCTGTAGCTGAAGGTGCTAACCTAAACATCAGGGCCAGGATATGA
AGGAGACTTAATAGACTAATTAATTTGTCTGCTGCACCCTGACCTGGGAAT
TACACACAACAGGCTATGCTTAGGAACCCAGAGCCTCTGATCCAGAGTAG
GGAAAAGGCGACTCTTGTGTGCTTTCTCTACCAACATACAATGAATACTG
GGTTCTTTTTAGAAAATAGGTATTGAGAGTGGAGCCTGATTCTGTTAGATA
CCTCTGCAAGTACAATGCTGTATGGTTTTCGTTGTTATTTCAGTTCCCCTGT
ATTACTGTTTATGGTTTTAACAAGTAAAACCATCATGTTTATGATCCTAAT
ATGGGTAAGGTGCTGACCTCCAGGGGACCACAATGATTACCATCATTCCG
TGGTTTTTTTTACCTATTCTGATATGGTAGAATCACTGTAGCTCAGAAAAG

ACGTGAACATGAAGCTCAATTGACACTTCAAAATGTGTTTATACTATGATC
TTCTCACTACGAGATCTCGGGATTCACACTTCTCTCGTCAAGAGGATTGGT
TTTTCTTTCCTCTTATGCTCTCCACCTCATTTCTCCCTCAGAAAAATACTTT
AAAAACTATAATTTAAAGCAGTCTGAGTGCCGTAAGAATTCATGCCACTG
GATGGCAGCTGAATTAAGCAAGTCTGTTCCCATCAGCCTGCCATCCTGTGG
ATTCTGTATCTCCCTGCGCTGACTAAATCCATTCCTGTTTGAATTTTATTTA
GAATGGTGCCTTAACATTTCAGTAGCTTTGGGCATTGGAAATGCTCACTAA
ACCTCAGACATTTCTTTTTTTTTTCGGAGCTGGGGACTGAACCCAGGGCCT
TGCGCTTGCTAGGCAAGCACTCTACCACTGAGCTAAATCCCCAACCCCCA
GACATTTCTTGAGCTAGGCACTCTTCCACGTATGGTCGATATGGTGGTGAG
GACTGGCATTTTCTGATCTTGCTAGGGCTTGCATAGTGGAAGCCACCCACA
AAGGAGAGGTGTAATGTAGGGGACACTAGAGGAGAAAAGCGAGGTTAAA
TAGATCTGAAGTCTCGGTCGTGAGATAATAGCCTCGGAGTACATGTTGAT
CTCACACAGTTGTTGGTGGTACCTTTACTTGAAGAAATATGGAAGAATTCA
TATCCTTCTCACTTCAGGATGGGAATAGTAGATAAATGGGGTATCTGGCTT
AAAGAGTAAAAGCAATGTACCTTTATGAAGGTTCCATGTTGCTTCCAGTA
GTTACCAACAGGGTACAGGCTTCACATTTCCCTCTCTGCTGTTTTTAAGAT
GCAAGGTTGCGGGGGCTGGGGATTTAGCTCAGTGGTA
>LNC_006628
GCCAATTGTTTCTTTAATAGATCCGGTTTTTTTTTTTTGACATAATAACACA
TCAATTTAAATACAATCACATAAAGCATGGTGGAAAAAAATTTGCTTCAT
AATTAGAAAAACTCACAATAAAACTTGCCAAGGAAAACCAAAAACAAGC
CATTTTGAAAATAAATACAGTCCATGCCAAAACAGTATAAAATGAAGCCA
AACTTCCTCAAACAGCGAAGGTTTCTTATCCCAGAATCGTCTCACTTTTCG
AGGTTCCTAAGGTTGGGGAGTCAAATTCAAGGGTTGTTGAGTTGTTCTGAT
TTGGCTCCTTTGGTGTCTGTTTCTCACCACTATTGTCCTTGGATTTGTCTT
CTTCCTTAACATCTGTCCTCTTGTCCTCCGCCTCCTTCTTGTCTTCCTCAATC
CTGGCTTTTTTGGCTCCCTTCTTATGATCAGCGACATTCCTACGACCTCCTT
CACCTTCATCCTCAGAATCTGAGAACTCTTCATCACAAGCTATCCGCTTGT
CTGATGCTCGAATAGAAATTCTCTTGTCAGGGTCTTCTCCATCCTCATCAC
CACTGTCTTCATGAACAGCATCTTCTGGAATAGCTTGCATTTGAACACCAG
GCGCATGTGGTAACATTCGCAGATTTTCAAATAAACGCTGTTTTATCTTTT
CCATATATTCTGGAGTGTTCTGGTTTGTCATGTTTGAGGGACTAATATGCA
GTTTGAAGTCTGGTCCAAAATACTCGAAGTAATCATTATATGGCAACTCAT
TGGGAATTTCACAATCAAGGGCAACTGCAGTCTCATACGTCCAACATCGA
GCAACATTCCGGATTGTGTAGCCACCTCCGCCAAGCATCAGCAACGGCAA
GTTAAAAGTTTTCACTACTTCTACACATTTAGCGTGACCTTTGACAGTTAG
GTTGAAGCAGCCCAGCCTGTCACCAGAGAGGGAGTCAGCGCCACACTGCA
GCACCACCGCGCTCGGCTGGTACATCTCCATCACTTTTGAGATTATAGGCT
TAAATATCTGTCCATATGACTCGTCATCTATACCGTCCCTCATGGGAAAGT
TGACAGCGTAGTACTTTCCTTTTCCAGCACCAATATCCCTCAAGTCTCCTG
TTCCAGGAAAGTATTCTCCGTATTTATGGAATGAGACGGTCATCACGCGAT
CTGTTGTATAAAAAGCTTCCTCAACACCATCACCATGGTGGATGTCTATGT
CAATATATAAGACTCTCTGATGATATTTCAGTAGCTCGAGGACAGCAAGC

ACAATGTCATTAACGTAGCAGAATCCTGAGGCCTCTGACTTCTTGGCGTGG
TGCAGTCCTCCCGCCCAGTTGACAGCCATGTCAGTCTGTTGACGGTTCAGC
TTCACGGCCCCAGCAACTGAGCCACCGGTGGAGAGCTGACAAAACTCAAA
GAGTCCATCGAACACTGGACAGTCTTCTCCGACATTAAATCTCTGCATCTG
CTTACTATACTCTGACATGTTATCTGGCCTTATTGATCGTAGAAACTTGAT
ATACTCATCACTGTGGTATTTTGTCATTTCTTCAGCAGTGGCCTTATGAGG
CCTATATATTTCCATTTTTCGGTATAAACCATAATTTAGAAGCAAGTTATG
AGTCATCCGGATCCTATGGGGCTTCATGGGATGACCCTGGCCGTAATAAT
AATTCCCGATATCACCGTCATAATAGTAGCACACTTTCTTCTTGCCGCCTC
CTTGACTGTACGCCATGGGCTCCGGCCGCCGCCTCCTGGCTGCGGCTCCTC
CTCCGGCTGCTCTCGCCCGCCGCGGCCCGGCGGCCAGACAAGAGGGCTGA
GGGAGACGGCGGGCGCGGCGGCCCTCGCAGCAAGGACAGCCCGGAGCGG
GACGGCGAGCGGGTGCCGGGAGGGCCCGAGATCGCAGAGCGGGGTTGGC
AGCGGCGCCGACGCGCGACACTCGGCAGGGGTAGGCCTCCGGGCGGCGG
GGCTGCGAGTTGGGAGGAACCACTGCGGCCGCGCGGAGGGGGAAACACA
GCTACGGGCCGGAGGAGAGACCGGGCCGGAGTGGATGCCACCGGTCGAG
GCTGCAGCAGTGCTCCGTATGTCTTATCACACACACAGTCCGGAGGTGCG
CACAAGCCGACCTTGGCCCTGTGCTCAGAGTCCCGAACGCTCTGCCACCA
CGTCCCCCTCTCGGTCTGGC
>LNC_006659
AAAGTGTTTAATCAAAGTCTAGGTTTGGGTTGCTAGCAGGTGTCTGAAAC
CTACCCATATCAGATTCTTGCTGAGTTGAAATTCGGTGCTGGTAATCCCTC
ACATGCCGGTACACAGCAGGAAAAATGCCCAGATTGTGCATTATCTGTTA
AACATGGGTTTAAGCCAATAGGAAGTCTTTATTAGCTGGCTGGTGGTGAC
ACTGAGTGTCCAGGCTCCCGGTGTAGCCCGAGCCTTTCTCAGGGTGAGGT
GTTAAACCCCAAAAACCAAGTTCTGGGTTGACATACTTCAGCCAACAAGA
ACAGTTATCCAGAAGCAGAGCTATAGAAGCCAGGAAGCAATATTAGTCCA
TTTAGAAATTCTTCCAGAACTATAGATTTTGATGGACAAGGCCTTTGTTTT
CATTTTTGCTTGGGAGGAGGAGAGGGTGCGCTGGCCTTATGCTGAGTTTTA
CATCTGATCAGCACTTACACCGTGGAGTCAGTTGTGCTAAGGTCTCAGAG
GCCTCCTGAGTGATTGTTAACCATCTTGTCAATAACACGAGTATTATCAGA
GGTGGCTAAGAATGGCACATAAGGGAGGTGGAGAGATGGCTCAGCGGTT
AAGGGCACTGACTGCTCTTCCAGAGGTCCTGAGTTCAATTCCCAGCAACC
ACATGGTGGCTCACAACCATCTGTAATGGGATCTGATGCCTTCTTCTGGTG
GGTCTGAAGA
>LNC_006688
CAGCATCAGGAAGGACACTGAGCGGAACCTGTCATGTAGGACTGGTGTAT
TTAGGATGTGTGTAGTGTGGCACATTTATTGCAAGTCCCTAGCCTGAGTGG
AGAGGGCAGACACTGTGCTTCCCCCAGTGCCTGTCAGAGCTGCCCGTCTG
TCCATGCTGGCCTTCATGCCGACCCAGCCCGCAGCAGCCCTGTTTCTTCCG
GCTCCCACCGAGCATCAGACCCTCCCTTCAGCGGGTACCACAGCACCCGC
AGCTCCTTCTCCCTCACGGCTTCACTCTCCTCTACATGGGTCTGGTCCTAA
GATACAAGGGACTCCACATATCTCTCCATCATCTCTAACACAGAGATCTCT
GCCCTGCCTCTGGCCCCGTTCACCTCTGGGCTTGGAAAGGACCCTGTGGCA

TCCATTCTCCACGGTTGCTTTTATTCTATTTGCTTTTTAAAAGTGGAGGTG
GAAATTTAAAAAAAAAAAACTGTGGGAAAATGTAAAAGCAAGAAATAAC
AACAGCTGGTGACTTCAATGGTGATGCTCCACTGTCGATGTCTGGCCACCC
TAGACAGTCCTGTCTGTCACATGTGTGACAGGAGATCCTGTGCCTGAGTTT
CCTCCCCATCCCTGAGATCACTGTGCTTACAATCTGGGTTTCCCCACCTTC
CTCCTAAAGGCCACCCCACTATCTTTACCCTTTGCTCCCACCATCTCCCAG
TCACAACGCCCTTCCTGGACCTTCTGTCTGCTCCTTGGCCTCCTTCCCCTGC
CCCTCACTCTCCCAGTCAGCGGTCTCCTCATCCTCAACAGGTTCCCGGAGG
CTGACAGCCAGCACTAGGGCCTGCAACAGACCAAAGAGGCAGGCAAAAT
GCAGGGGGTGGGCAGTAAGCAGGCTCAGCACAGCATGGAGCCCGTGTAG
GGCAGCCAGGAAACACAGTAGCCCGTGTAGCCAGAGGCCCACAGGTCCC
CGAAGGCCCAAAGCATCCAAGATGGCTCGGAGTGGCCTCGAGCACAGAG
CCAGCAAGGCTGAGGCAAGCAGCTCCAGCAGGTGCAGGGTCAAGTTGGT
AGAGATCTGCAGATACTCCTCTGGGTCCTGCAGGTAATGTGAGGGAGCCC
CTGGTTCCCCCCGGAGCCACCGTAGCAGCTTCTGACGTCGACCAGGTTTCT
CCAGTAGGGCTTCTGGCCCAGTGGTGCCTAACCTCCCTTGGGGGGACACT
CCAAGGTTCTTCATGCCACCTGAATCCACAGGATCCAAGTCCAGTTTGGG
AACGGCTCCTCCAGTCTCCAGTCTTGCAGACTCTTGAATGGCAGAAGCAG
CCTTGTCCTTCTTGAGGGAATCCATTCTTTTACTCCCCAGCTGATACACTCT
GGTGTCCCTGCTAGACTCTGGAGCAGCCTGGGGGGGCCTCCGTGTTCCTG
AATCCTTGGTCTTCAGAGGTCCCACATCTCCCATGGTCTCTGGAGCACTGT
CCCAGTCACTCCCTGAGTTCTCCGGGGAGCTGTCCACCCATCTGGGTTCTG
GGGCAGGCAAGTCAGTCTTTGTTGATTTCTTTCGACCCCAAGGTCGCGGCA
GCCAGCCACCAAGCCGTCGAAGGAACATAATTGAGGAACTGTGTGCAATG
AGCTCCCCAAATCTGTGACCGCTTCCTGACTCTGCTTAAGACTCTCAGGAT
CTCCCCCGAAGCCAGAATCAGTTCCGGTTCAGAAGAGGTATCTGCTTGGT
GATGTCGTCCTACTCTGAATTCAGAAAGGACTCACACAGCCCCAAGCGTG
GTAAATGGTCAGCTTCCGTGTCAGTCAAGACCACATCCCAACGTGCCCCA
GAAAGGGGAGAGGTGGTGCTCCATTGAAGAAGGAAAGAAGTGGTGGCCC
AGGGGTCCTTCGGTCCTGGATTTGAGGAACCGTAAACCGCGGGAGCTCAA
GGAACAGGAGGAAGTGTCTGTTGAGAGGAAGACATCGGGATGGAAGTGT
CCAGCGAACTCCAGTCAGTTACATGCATCCCCTTAACTTAGGAATCTATCG
TTCCACAAAGTGGCCCTACTGTCCCGTGAGGGCGCAGCTGAGACGCGGCC
TCCGCAAGGTCAGAACAGAGATGTCCTTGGAGATCGCAGTCGCAAGACAC
AGTGCAGGCCTACCTGGGAGACCCAGGCGTCGGGGTGCTACGCGCAGGG
AGTAGCTGTAGGGCCC
>LNC_006689
GGCTCCCTCCTTACCCCATCTCTGGGAAAGTGGCTCAGGCAGCCCCTCATG
CTCCACACGGCATGTGTAATTCTGCTCCTTCCCAAGAGGCACCACCACAG
ATGCCCACTTCTGGAAGGTTCCATCCCCTGCAGGCCTGGTCTCCACAAGCT
CCATGTCCTCGGTCAGGTCCTCCCCATTCAACTGCCAGGTCAGGGTGATGT
CAGCAGGGTAGAAGCCCAGGGCCCAGCACCTCAGGGTGACATCACCTTCA
GGTCTGGGGTGAAGGGTCACATGTGCCTTTGGGGGATCTGAGCCCAGCAG
CGCCTCCTTCCCGTGCTCCAGGTATCTGCGGAGCCACTCCACGCACGTGCC

CTCCAGGTAGGCC
>LNC_006737
ATTTTTATTTGGTGCCTCTTTGAAATGGCTTCAGATCACACATTGTCACAG
AACCAGCCCCATTGGATTGTCCCAATCCTTGGACGCAGAGCCCGAGGCAG
GCACAGTGGCTTTGATTGACCACTTGTGGCCCTGAGCACACAAGTCCCTCC
ACAGGACAAGTGCCTTTGCGCGGCTGCTGAGAGATTTGCGGACTTCAGAC
TGAAGAGCGAGGACAAGGCTCTTCTTGGGCTTGGCTGGGGTTGGGTTCTG
CTCTGGATGGGATCTCAGGGGTCACCAGAGAAGCCACTCTGAGTGACAAG
CCCCATGTCGTGTATGGCCCTCAGGAAAAAAAATGAGCACCAGGCTGAAT
CTGGCCACATTCCTGGTCTCTGCCCACGGTGACAGGAAACAGGGTCAGAT
ATGGGGTCACTGTGAACTTGGAAACCTGCTCTGGCAGGAAGTGGGGGAGT
CGGGAGAGTTGGGTCCCACTCCTCAAGCATGAGGAGAGCCAGTTACCACA
TGGATGAGCAGGTGCCCGCCTGTACAACTGGCCACAGCCACTGGACGGTG
AGAGGGGAGCCTGAACTGCCCTGTGCTCTCCCCAGCACGTGCGTGTGACT
GCTCTGTGAAGTGTCTGCACAGAAAGACGCCAGGCTAGAGCAAAGTCTGC
AGGTCTTAGACAGTAACTCTAGGTCATCAGCCTTCTGCCGAGACCCAGCT
ATGGTCCAGCTATGGTCCCGCCAAGCATGGAGGAAAAAGGAACCGTGAC
AGGCTAGCCAGTTCAAGTAGGAGAGGAAGGGCAGGAAGTCTACTGTAAA
TGTTTAAAAAAATATACTCTTTGGCTTCCTTTTATTTCTTGACGATTACATA
AAATGTGAACTACACAGGGTCGTATCCTAGCCAGACCCTGGGTGCTGGCT
GGATGCTCCCTGCACTGTGTCAAAGTTCTGCATCGCCTCTGTCCGCCCTCT
TAGCTGGCAGAGGCCCTATTTGGAGAAAGAGGTCATGAAGCTATTCTCAA
TGCACAGGACGATATAGCTGTTGTGGCAGCTCTCGGCCTTCTGTTCCAGGA
GCCTGCCCGACAGCAGGGAAGAGGCCTGACCTGTAACCCCAGTAGCTTCA
GTCCTCCACGTCTCACAGTAGCTCTCCATCAGCCTGCGCCCACTGGGGTCT
GAGCCATGCCATACGCTCTTCTGTGGCCAGGCTGGGTGTCTCAGGACGTCT
CTGCCGTCGAAAGAAAAGATGCGGGCCCCAGAGTGCAGTTGACCCTGGGA
GCCGGAGAACAGGGTGTCCCAGCTGGGAGATAGTACCTCATCCTTCAGGT
TGACGATGGGCACAGAGCTGCGGTCAGCACGGCGTACAATGCTGTAGAGG
TCCTGCAGCCTAGAGGACAGGAAGGCCCGAAAGGTGCCAGACAGCCCCA
CGGCTCGGGCTTGCTGGAAGCACTGGAAGTCAGCCCCACGGATACCACGC
ATGCCTCCAGACAGAGGGGTGTTCAGTGCCACCAGGTGGAGCACTGGGTG
AAAGTCCTGGTGAGTATGGGCAGGTGAGGGGGAGGGGTGGGCTGGCGGC
CGGTGTTCATGAGAACTGTGGTGGTGGTGGTGGTGGTGGTGGTGGTGTGG
AACTCCCGGGTAAGGCTGGCGGTCCGGCAGACGTGGTGGGTTGGCCAAGA
TGTCATCTGCTCGCCAGGGTCGTGCCGTGGGATAGGAGTGCTCCCTCCGG
GTATATGAACTGCCCTCATGAAGCTGTACCAATGGAGGTTGCAAGGCAGC
TACCTCGTTGTCCGTCCCGTGCGGGAGGGCTGTCCGGGCCTCCAGCAGCA
CCTTACGGAAGCCATTTCTAACCCGTACGTAGAGCTCTTCCCTCTCGGCCA
CAAAGATGAGCCAGCCCTCTGGCACCTCACGTATCTTGTCCAACATGGTCT
GGTATGTAGCCCAGATCCTTACCCCAGCAGAGGCACCCATGGCTCCTGGG
GGACCTGGGGGGCCAGGTGGGCCTGGAGGACCAGGAACACTGACAGTCT
GTCTATGAGGGCCAGGGAATGAGGGAGGTCCTGGAGGTCCTGGAGGTCCT
GGGGGGCCCTGGCGCCCCTCATATCCAATGCCAGGAGGTCCCTGCGGGCC

AGGAGGGCCAGGCGGACCCCGAATGCTCTCTCCTTTTGGACCCGGAATTC
CAGGGTATCCAGGTGGGCCAGGTGGTCCAGGTACACTTGAGCTGAAGAAT
CCACCACCAGGAGCCCCAGGCTCTCCCCTCTCTCCCTTCCGTCCAGCATCC
CCTCGGTCTCCCTTGTCCCCCTTCATTTCGGCTTCCAGGTGGAAGAGGTCA
ATGGGGAACTGCCCTGGTGGCCCGGGTGGGCCCACCTCTCCTTTGTCACCC
TTTGGTCCTGGAGGCCCCTGCACACCTTGCTGTCCTGGTAGTCCAGGTCGG
CCAGACTCCACAAATGCATTGCTGTCATAGACGGGCACTCCGGGAGGACC
TGGGGGGCCTGGAGGCCCAGGGGGGCCAGGCAGTCCCCTCATGCTGAACC
CAAGGCTGGCCTCTCCAGGCTCCCCTTTCTCTCCCTTTAAGCCATTTGTTCC
GGGACGACCCGGCCGTCCAGGGAAGCCAATTTCACCCTTGTACCCAGGCC
GTCCATAAGGACCCGGGGGTCCTCGGAAGCCTGGTTCTCCCTTGGCTCCCT
TCTGGGCCTGGCCCAGGGCTGTGCCATCAGGGCTAAAGATACTGCCTGGT
TCTCCCTTCTCACCCTTGGGCCCTGGAAGACCCTGCTCGCCTTTGGAACCC
AGGTCACCCTTCGGTCCAGCAGGTCCAGGAAAGCCTGCGTACCCTGGCTT
GCCCTCAGGTCCCGGCGTGCTTACTACAGCTCTATCCTCATTTGACACATA
GATCACAGGCCCTGGGGGGCCTGGAGGGCCAGGGAGGCCTGGCCGACCT
ACTCCATCTTTTCCTGGGTTTCCCTTTTCTCCCTGGGTCCCTTTCTCTCCTTT
GGGTCCCGGGGCCCAGCTACACCCTCTCTTCCTGGGAGACCAGGGATGCC
CTGGACTCCGTCTGCTCCAACTTCTCCCTTGGCCCCAGGTGGCCCAGTCAC
TCCAGGATCCCCCTTGATTCCCGGCAAGCCCGGAAGTCCCTGCAGCCCATC
GGAGCTTCGGGCTGTTGACCAGAGAGGTGTTCCAGAACCTTCCATGTCAT
CAAATCCAGCAGCAAATCCTGGTCCTGGTGGCCCTGGAGGCCCTGGAGGT
CCTGGGGGTCCAACTGGCCCAGGGAGTCCCGGCAAGCCAGACTTCCCAGG
CATACCAATGGGCCCAAGGTCTCCTTTGCTCCCCTTGGGTCCTGGGCTGCC
TGCATCGCCAACACTGCCTTTCTGGCCGGCCACTCCTGGTGGGCCCTCTTT
GCCTGGAGGTCCTGGGGGACCAGGAAAACCAGGGGGCCCTTCCTTCCCAG
GCACACCAGGAAGGCCTGCAGGTCCTGGTGCGTAGGAACTGTTGACTCCA
AAGCGCCCTGGTTCACCAGGCAGTCCTGGGACACCAGGGGGACCTGGGGG
ACCAGGGAAGCCTCGTGGGCCTCTGAGGCTCTCTAGGTCTCCGCTGAAAC
CACCAGATCCCTCCATGTCAATGAAGGTCAGCTTGTCCTGTCTGAAGGAG
GGTCCTGGTGGTCCCGGAGGTCCTGGAGGTCCTCGAGGCCCAATACCAGG
ATCTCCCTTCTCACCCTTAGGGCCCACATCTCCTGGGGTCCCCGGGAAGCC
TTGAGGTCCAGTGTCACCCGGCCTCCCGTCTTCACCAGGGTCGCCTGGTTC
ACCATCCCTTCCCGGGATGCCATCCTTCCCTGGTGGTCCCTGAGGTCCCGG
GGGTCCTTGTGCTCCAGGGACAGGTTGTGAGCTGGGGCTCTGTACCGCTG
GACCGGCAGGACCCTGGGGCCCAGCTGGGCCAGGTGGGCCTTGAGCACCT
GGCTCTCCCTTTTGTCCTTTGAGACCTCCCTTTATCAAGCCACCTCCAGGGT
TCTGGACATCCCCGTCCCACACACCGCTTGAATCTGTGACAGGAAGGGTA
TCAGCTCCTTTAGAATCTACCGTGGCTTCTTCCTCCTTTTCTTCTGTCCTAG
AATCTTCGGTGGCACTGCCTCCAGCCAAGGGCGGGGAAGTGACAGGAGGT
GGCTGGGGGAGCCCGGGTCGTAGATATGTCTCCTGCCTGTGTAAATTGCT
GCTTTCTTCGAGGCCACTTCCAAAATCTCCAGATGCCCGGTCGTCATCATC
ATCTTCTTCATCCAGGCAGTGCACAGGGCTCACCCGGGGGGTTTTGCGTAC
TCTCAGCTCTGAGATCATTCCCTGGAACTTGTCAGGGTCCGCTGCTCCAGC

CTGACCCACAAAGAGGCCAGCGCCACGCTCTAGCTCCAGACCGTGTGGGG
ACCGAGCGAACGGCACTCTCTGGAACTCTTCACAGTCTACGTAGAGAGCC
ACGGAGCTTCCATCCACGCTGAGCGCGAAGTGTGTCCACTGGCCAACAAA
CGCAGGTAGGCGGAAGCTGGCTCCCGTCTGGGTCTGGCTGGCCCCGGGCT
CCGTGTAGAGCAATGAGATGTTCTGTTGTCCATCTCGGACCTCTGAGAGCT
TCACGCCTAGCGAGACCACCACCTGGGCGGCATCTGTGATAGCAAACAGC
ACCCCTGCGGCCTCTGTGGTTGGCCGGACTTCGAACAGCAGCGAAAAGTC
CCGGAAGAAGAGTTTAGGGAAGTGATACTGGGCCATCTGACTACTTTTGG
AGTATGGTCCAAAGACGTAGGCCAGCCCGACATGAGGGTCTTCGACTTGG
GAGATCTTCTGGGGTAGGGGGTCTCCGAGGAGCTGCAGGAGCCCCACATC
TTCAGCAACATTCTCTGGCTCTGCCCAGGAGACGCGTGCCACCAGCAGCA
AGACCAAACTGGTGAGCATGTCCAGGAGGTGCCACCTGGGCGCCATCTCC
CCAGGCCAGGCCGTCTGCGCCCGGATGCTGCACCCAGCTCCTCCGTGAGC
CGCTTGCCCACAGCGCTCCCGACCGCTGCTGCCGGCTAGGGCAGG
>LNC_006836
GCATATCAATACTTTAATTCCTATGGATCAGATTGCTCTGGCCTCTAAGGC
CTGGCAGGTGGTGTGTTGCCCAAGGTACAAAAGGAGAACACAGGGCCAG
CCCCTCAAGACAGAATACAGGGAGAGCAGAGTCCTGACTTAGATGCTGGT
GGGGAGAGCTTCAGTAGCCATCATCAGCCCAGGTGACCTCATAATCAGGA
TACTTGGCTTTGATCTTCTCAGTTGAAACAGAGTGCTGGGCACGGCCGTAA
CCCATAGAATAGCCATACACGTGTATCTTCCTGTCCTGGCTCTGATGGGAG
ATGCGCCCGCCGCCTAGGCACTCGCAGTCATAGCCATTCTTTTGCAGTTCG
CCTGACACTTTGTCATAGATGTCCGCGTGGTATTCAGCCCATTTGTAGCCA
CGCACGATTTCCTTGCACTCCCTCGCCGGATCCCCGGAAGGCTCTGCTAAG
TGGACTCGAATCAGCACGTACTTGAAGATACCATCTGAATCAATGTCCAC
ATCAGGAATCTGACCGAGGTCCGCCGCCATGTTCGCC
>LNC_006871
TAACAATGAATTAATTCCAACTATTCGAAATCTTCTGTTGACTTAACATTT
ATTGAATCAGCGCTGACGTAGATGTCAGACTCCCTTCTAGCTGCAGAACC
GAGTCACAGTTTGGCTTGCAATGTACTAAGGAAGGGAAGTGACTTCTAAC
CCCAAGGCAAGGTGGACGACAGCCCATGAAGTACACAGATTTCCTGACCA
ACATCTGAACGGGTGTCTGTGATGCCAGTTTAGTTCTGATCTCTCCCTAAC
AGGCAGAAAGCACATCTCCCAGGCCTGTGCAAACCCGTCTTGGGGATGAA
AGGGAAGGAGAGGGGAGCATGAGAAAGCCCAGCTTCTTTTGGCACAGTG
TGGCCCAGTACTCAGAGCGGGAGCACACGGCCTCTACCCGTCTGCTCACG
GCTGGCTGCCACGGGCCCCTTAGAAGATGCCTGTATTGCTGCCACAAGGG
CCTCGGAAGTATGAGATTGCAGCATTTGTCAAAAAAGCTAAACAGGTTTA
GGAAACATACAAGGTGAAACCATGGGCGATGGCCTATGTACAGGACTTGT
GCTTGAAGCTCTGGTGTGCCTCCCTGGGCAGCCGCTCAGGCACCAACGCC
CATGCCTGATTAATTACTGATGTACAGCGCAGACCAAACCACAGACACGA
TTTTTTTTGTTTTTTGTTTTTTTTTTCCAGAAGAGGGCAATAAAGGCACAGA
AACCGCCAAAATAGATTTCCCATAGAATAGTGTGGCTGATCAAAATCGCT
TAACACTAGTAAAACCTGGATCAGTAGAGCAGACGATACAAATGAGTTTT
AAAAAATGACATTCACTGAATTCCTGGTCTGTGCACTCGATGTGAACCATC

CTCAGAGACGGAGGACGGCTGAGGCTCGGTGAGCAGGTGTGAAGGAAGG
ACGCACGTGCACCCTGCCACGCACTGCTCGCCCTGGGCAATGCATGGTGG
AGCCTGCCCCAGTGTGCAGGCTCAACATATACCATGGTGGCCCACACCTA
CATGACTTCCCCATCAAACTGCACACATGGAGAGTGATAAACTGTGTCCA
CACTAATAAATAGCTCTGCTTTTGCTTTTGAGCTCAGCTTAAGTTTTGTCA
AGGAAGCCATGTCACAGAGGGAATGTCACGCTACTCTCAGCAGCTGCTCT
TGTTTATTAGAGTCTGAAGATGTCATATCACATCAAATATAAATATATATT
ACATCTACATGTCCTTCTCAGCTCTTCCTTAGGAGACTGTATCTCCGAGAC
CCTCAGTGCAGGTGGCCTCCTGAGCGAGCACGCTCACACAGAGACTTGGA
AGAGGCACACTGTTCTTTCCAGAAATGAAAGCGACGGACAGGGAACTCAT
GAACCGAGATCTAGATGTTCCCTAAAAGATTCTGTTGTGGTTTTTAAAAAT
TTAAAACACCTGAAAAACATGGTCCCTTTTGTAGTACTACTCTACGTGGGG
ACAGGAAATAAAATAGGCAACTCAGAAAGGCCGAGAGGAGCGAGGACCT
GCTGTTAGCTTGTTAGTCTGCTAGGAAGAGCCGTGAGGAGACATCTGTAC
AGCCTTAGACAAAACGGTGTTCAGCTTTCAAGGAAGCTGCTACACACTCT
TAGAGATCTGTTTCCTGGCTACAGTCTGCTGCCTTGGTGGGCCTGGTCTTG
GTCACTTTCTGTCATCGATGGTTTTAATAAACTCCACGGCCGCAGTGAACT
GCATCCACCAGTAGGACTCCTCTCCAGACAGACAGCTGGCATAGAAGCTG
CTGATGTACTGCACGGTGGACAGCAGGCAGGGTGGGTTTGCCTTTATCAA
CACAAACACCAACACGGGGACAAAGTCATCTGCCCCGGGGACAGAGTCCT
CATTGGCCAGACTCAGGAGGTTCATGATAGTGGAGCACATTCTCAGGATG
CACTGCACTTTGTCCCGGGGCGTCTTGTAAGCACTAATAGTCCTGATTTCT
GACTGTGCAGATGGCCACGGTGCCTCACGAAGATAGACCTCTGGGATCTG
CAGAGCTCTGTGGTTTGCAGTCACTACTTTAGATAATCTCTGGATGTGTTC
ATGCAGAACCTGGTCACGCAGTATGTCCCCATCCTGGTTAGGGTAGAAAG
CAAGCTTGAAGATCCGATTCATCACGCTCCGCTCGATGGCCAGCTGGGCA
TCCTGCAGCTGCTCCTCGCTGGCATTCTGCCATATGACATCCTGCGCCATC
GCACCATACAGGAACTGTAGGAAATCCTCCACTTGAGCCGTCTTATCATC
AGCAGCCGTAAGTTTCTGAAAGTCTTGAATGAACTCCCTGATCTTCTTTTC
TTTGCTCTCAAGCAGTAACCGCACACAGACGGTGGTAAAATACCGATTGG
CCACCTCTTTGTCCCGCAAAACTCTTTGCAAGAGTCTTTCGAGGTGAGCCT
GTGTGGTCTGCAGTCCTTGCCGACAGCGGGTGAGATACGCAATATACGGA
GCTCTTTTTCTGTAGTCTTCAGCAATGGATGCCAGCAACTTCCTACAAGTC
CTATTGTCAAAGCGGCAGACACAGCGCATTGTTTCTTGAAGCTGAGCCATT
AAGCTCTTATCTTGTAAGTTAATTGCTTCAGCTATTTGAACTTTTAAGAAG
CACACAATTTCATTATCTTCCGGGTCTGTGTGCTCTGGCAGTCCGTTCCTTG
TTGAATGAGTCAGCATGGGGAAGGCCACAGAGTCTGCAGAGCAAAGTGC
GAGCCTCAGCTTCTTTTTCACATCTCTGTAAGAGAGGGCAGAGTCCTGAGG
GTGAGCTGCTTGGCTCGCAGAGTCTGGGAGGTCGTCAGCTGACATGTTCT
GCAGCTCCTCACGAGGAGAATCGTGAGCACTTTCACCATCACCCATGACC
TCTGTACTCTCATCATTTGCCAGTGCTCCTTCACTGGGGCTGGTCCGTTTAA
TGGCATTTCGGTATTTGTCCAGAATGTCTTCCGCAACCTGAGCTTGTGCAC
TGAGCCTAGGGCTGGGCTCGTCTGTGAGTGTTGAGAATCTGTCAGGCCCC
AGATCATCTTTGTCTTTTTCTTGTTTTTCTTTCTTTCTAAATGGTATAGGAG

CTGGAAATTAAATAAAGTCAGTACTGTGACATATAACATACTACACTCCT
CGGAAGTTAGCTGGTGTGCCAACATTTCTAAGTGCAAGGAGGCTGAAACT
TTAAAAGAAATCCCAAGCGTCAACTGATATTCTTAAAATAGCATCAAAGG
GTCTTAAGAAGCTTTCAGAATCTGCTGTGGAGTCATTTGCCATAGAAAGA
ATGATTTAATGAGGATCCACTTAGAGTAAGT
>LNC_006875
CATGATTCCAGCTTCGGCCTCCGCCACAGTCTTTTCTTCCTAAAATGGCGT
CGCTGGATCTGGACAAGTATATGGAGATAGCGCGGCAGTGCAAGTACCTT
CTCGTGAACAACCTGAAGGTGAGCCGGGCCGGAGGCCGTCCAGCTTGCGA
GGCAAGCGCCCTCCGGCTGTGAGGCTCCCTACGGGCGCCCGATCCTGTCG
CGGCGGAGGGCACTCCGCGGTGTGAGAGAACCGGGTCGGACGCGGCGTG
ACGCGGAGAACCAGGGGAACCCGAATTCCCCGAATGCTGTCCAGTTTTCT
GAGGGGAGATCCGGGGAATGTCACGATCCCCTGGTCCCCACGGGACTCCG
AGCTTACAGACTCATTCTAATTCGCTCGGTCTCCCGGAGACGGAGCCCAG
CGGCTAGGGTAGTGCTCCCGGGATGGGTCCTCCCTGCAGAGGTAACAGGA
GCTGCTGTCTGCTCCAGGGACGCGCGCGGCCCGTGTGTGCTTAAGTTCCTG
AGAGGTGGGAAGCCGGTGGGCGGGAATCGATGCTACCACAATGGGAGAG
AATGACAAGTCGCCTCTGCTTCACCACTCCCCTAAATTGCTTCCTAGGAGT
GGAATTTTTTCCTGATAGATTGGATCCTATTTCTTAGCACGTCGCTGAGCT
ATTTAAATAAGCAGTTGCTTATCAAGAAGTTCTGAGCTATCCCCCTTGTAA
CTCAAGGTGTCATTTTCTTCGCTTTCCAAAGTAGGTCTCACTCAGTTAAAG
TAGCCTGGCGGCGGTGGCACACGTCTTTAATTTCAAGAGTCAGCAGGCGT
ATCTAAACTACAGATGAGTTCCAGGACAGCCAAGGCTATACATAGAAACC
CTGTCTCAAAACCCCACCTTCCCGAAAGAAAAAGACAAAATAAAACGAGT
GTTAAAGGAATAACATATTTTGCTTCTGCTAAATATAGTGCTTAGGTTAGG
TCTCCACTGATGTTAATCCCATTGATGGTAGTTAGTGACTATTTTATTCTAT
TAACTTTGATTTATTATGTAGTAAGAGCATAAATGCTCGATCATCTGAATT
TCTTTTATGCAGTTTTTTATATTTTTTTCTTTTCTTTTTTTCGGAGCTGGGAA
CCGAACCCAGGGTTTATAAAGCTAAATCCCCAACCCCAGTTTTTATAATTT
TAAGCTATGGACTTACAGTTCTTTTCAAGAAAAGATTACATTTTTGCTTGT
GAATTATATTTCTCTTGGGAAGATTTTGCTTTGTGTTTGTTATTTTTTCCCTT
TTGGTAAACAAGGCTGATATTTAAATCAGTCAACAAGCATGCAGAATGTT
TAAGACCTTATATGGTAAGTGAAAGTACCTAGCCTGTACTGGTTGTATGTG
TTACATGGATGACATTCATGTATAGCCCCTGAATCTTTCAGTCATTTAGTT
TCTTCTTTCTGTTCCCTCCAAAGTTCTTTTGGTTTTGTTTTCTTTCTGAGACA
GGATCTTAAGATAGTTCTTGCTGGGGCTGGGGAGATGGTTCAGTGGTTAA
GAACACTGGCTGCTCTTCCAGAGGTCCTGAAGTTCTATCCTCAACAAGGA
CATGATAGTTTGTAACATCCTGTAAACTCCAGTTCCAAGAGATCTGATGCC
CTCTTTTGGCCTTTACAGGCACCAGGCAAACACATGTTTCACAGACATACA
TTCAGGCAAAACAAGTCATGTACATAAAATAAAAATAAACCTTAAAAAAA
AAAAAATCCAGCCCACTATATAACTATGGCCTGATCCTCTTGCTGCTGCTT
TCCAAAAGCTTCAACCTAACCAACATGCTCAGCTGCACTGTGTTTTTCTTT
CCTCTTTAAAAACAATTTATTTGTAATCAGTATTTGTAGATGTAGCTGTGT
ACACACAGTACCCTTGGAGGCCAGGCCGCTGTGAGCTCCAGATATGGTCC

TCTGCACAGGCTTAACTGCTGAGCAATCTCCGGCCCCACTCCCCACCCTTT
TTTTTGTGTGTATGGGTACAGAACATCATGTAGCCCAAGCTGCCCTTGACC
CCCCAATTCTCCTATGGTTTATTTTATTGTGGTATAAGTGTTGAACTGAATT
TTTGAGATTTGTTATTTATGTATATGTATGTGTGCCTATGTGAATTTATGTG
CACCATGTGTATGCTGGTGCTGGCAGAGAGCATTCTATCCCTTGGAATTGG
ATTTGCCTATCTCCTGGAATTGGGTTTGTAGGTAGAGATATATATATATCT
ATATCTATCTATACACACACACACACACATATATATGTGTATATATATATA
TATATATATATATATATATATATATATATATATTAGTAAGCTTTTATAACT
GGAGCCACTCCAGTTCCTGTTGTGTGCTTTTAAATTTACTTTATTTTTGGTT
TCTCTATGTAGCCCCAGCCCCACTTTACTGACCAGGCTGGCCTAGATGGCT
GACTTATATCTACTGCTGTCTGTGTGACTGCACACCCCTCCCATCCCTCCC
ATCCCCCAGAGGCAACGTTAGATCTCTCTGGAACTGCAGGTATAAGCAGT
TGTAAACATCTGACATGGTAAACACCTCACATGGGTGCTGGTAAGAAACT
CCGGTCCTCTGCAAGAGCAGTATATGCTCTTAACCCGAGTAATTTCCCCAG
CCTACACCAAAAAAAAGTTAGTTTTAGTAGTTAATCATAAAGTTAGGGGA
GGGAAAAGTCTAAAAAGGATTTTTTTTCTGTCCTGGAAATAGTTTTAATT
AATTTGCTTTATATATTTGCCTAGAATACTTTCTATCTCAAAATAGATATTT
ATGTGCATACATAATGTACATGCAAGTGTATCCGTGTATACATCTATGTGT
GTACAAGGGTATATTTCAGCCTGGGAGGAATCTTAATCTGATCACACATCT
GAGGAAGTGGGCCAGAAAAAGCACTTAACTGAAGAAATGTCAGGCACTG
ATCTAGCTCAGAGCTTCTTATCACCCCAGGTCACCTGTGCCACCAACCACT
TAGTGACCAGCCCTTTCATATATAAACTAAAGCTTCAGAGAACCTTTATGT
CTTAACATGGCCAACCCTGCCTGTGCCAAGAACTATGGTTTATAGCCCAGT
GCAGTCATCCAGCAGACTCAGATCTGTGTTGGAATTCTACATTTTGTAGAA
TTAAGTGATCTAGTCGAAAGTTATGTCACAGAATTCAAAGGTGTTTTAACA
ACATGAGATAAGTCGCTATACCATTATAGTACCTTTGCTTCAATTTCAAAC
AAAGGCTATTGGAAAAAAACTTTAATAACAGACTCCATGTGCTTTTATGG
GCAACCAATTAGCAGATTAGTGAATCATTTAACTTTCTTTACTCAATATTC
CACTCAGAAAGATAAATCCAAAAATGCAACCTCGTACACTGACCAACAGA
ACTAAGTACAGATGAAGTCAATGGTGTGTCCCATTAGCATGCTGCGGTGT
ATGTCAGTAAAACAAGCCCTCCCCGCCCAGCCCTGGCCCCTGGCAAACAG
ATAAATGATTGTGCACTGCGTGATGATACCTTTAGGTGAGAACTTTGGTTC
ATGCAGTTGGCTGCCACAGATGTTGCACCCAAAACCCGCAGCCCTGGCAC
CCAAAGTCAGTCAGCGGTGGTAGCGCTTGGTGTCAGCCATGCTCCTTCCAT
AAAACCAGGTGAATACTGTGGTCAGGCATGCTGGTAGCAAAGACTAAGCC
CGTGTCATTGTGCCCATCCAGTCCATTCAGCCAGGACGCTTCGCCTGCCAG
GAAGCTGGAGCCTCTCTTTGATTTCCTGTACTCTGGCAGAGGCCAGCGGCT
AATTAGGCTCTCTGTCCTCAAGTCCATGATGTATAGGTAGTGGTTGTCAAA
CAGCAGAGCGAAGACATCTCCAAAGCCAAGCAAGGCCAAGTTTGCTACCT
CAGGTGTCTTGATGACCCTGAGGATATCATAACTGGCAAAGTCCCACTGG
TACAGACCCAGGGCTGAACTACAGACAATGTATTTTCCATCAAAATGAAG
TCTTGGCTGTAGGCAGATACTTCTATCCTCAGAGACAGACAATGTCTTTAA
GCACTTACAGTTGATTTCTCTCCCAATTGGCCAAATCTTGATCTCGTACTT
GTCTGCACTTAAAAGGATGTAGTCCCCAGGGCTGTGCAAGAGAGACTTGA

CTTTGCACTGCTGCAGAACCACCTTGGTGACCCATTCTGTGTGCCCAGTGA
GTGTGTTCAGGCATGTCCCAGCAGATAAAGCCCATACTTTCACAGCGAAG
TCCGCAGAGCCACTCACCAAAATATCCAGTTCATCACTGTAGTCCACACTG
AACACCGCCCCTGTGTGCCCCCGGAAGTGCTGGGTCCTGGCTCCGGAACT
CCATTCCCAGCAGGCCACAGTGTTGTCAAAGGAGCCTGTCACAAGCTTCT
GTTCATCGAACTTCACGGCTGCACAAGTGTGGGTCTGGATGCCGTAAACA
CACTGCCCTGTGCTTACATCCCACAGCTTTGCAGACAAATCATCTGACCCT
GTACAGAGAAGTCCATCCTTGTAGTAAAGTGCATACACTCTGGCACTATG
TCCAATTAATGATGAGGTCTCAAAGGCTTCATGGTCCTCCAGTTGCTTCAT
CCTCAAAATAGCCTTCAAGTAAACCTTCTTCCAGTGCAATGCGTCCTGAAC
AGAATCATCTATTTGCCAGCCCAAATTTTTACATGCAGTCTGCCACACCTC
TGTGCAGGCACTTATCACCTTATTCCACTGCTTAGAGACCAGGCAGCATGT
GAGCAAAGTCTGAGGATCGAGCCATTTTAACAAATAAAAACTGAGCTCCA
GGGGAAGGAGTTTGAGGAAGTCCCGCTTGAGGAGAGTCTCCAGGTTATTG
GAGAGATGCCTGAGCTGGACTGCCCCACTCAGACTAATCAGGTGGTCCAG
AGTTTCATTTTTCTGAGCGAGCGCCCCGGGGCCCGGGACCTCGCGCCGGG
CTCACACCGACTAGGCGCGCTCGGGCCGCCAGAGCGTCGCAGCGGCCGGG
TCCGCTCCGCAGCCATGGCGCCTGGACCTGCGGCCGCTGCTCTGGGTCCGC
AGCCTCACAGGGGAGCGGCTTCCGGTGCTG
>LNC_006882
CCTCCTCACCACGGAGGCGGACCTGGAGGGATCCCGATTTAGCTCTCGCG
AGAAGATACTGTTCACTTCACGCGAGATTACGCAAGCCCGGGAGTTGGGT
TGGAAGGGAAGCGTAGTGGCCGCGCCACCTAAACTGCGCATCAGTTTCCC
TCTGAAGAAGAAAGCCACCTTCGTCACATGACAAGGGAACCCCAGAGTAC
TGTGGGAGATGATGCGAGGACTAGGCAAGACGAAGGGCCTTATGGGAAA
CTCCCTGTACATCTAAGCCTCGAGCCAGACCAGCTGGATCGAGCCAACGT
TAAAATTATCATCCATAGATACATCTATGTTACATCCTACTTTACTCTCTTG
TAGTGAGATAGTAACCAGATTAGCAAATAATTCTCCAGTTTACAAAGATC
AGGATCAAAGTGAAAACGTTTGGCAAACCCACCTACGTCATCTAAAATCC
CAAATGCCTCAAAGGAGGAAAAGATGAGAATTTACAAGTTTTGGAATTGT
AACTCAATTTTTTAGAGGACTTATGGCAAACTGTAAGGGAAATACATGCC
TGCGTTGGATGAGGTACAGGGCTGTGGAAACTGCCCGTTTTCTATATCATC
TGTATGGATGGATTCCACATGAAGGATACAAATTTCATTCTCTTTAAAGCC
ACCATCTACTGTTGAGAGCTCTGGTGACTTCACTCCCCTTCGCTCTGATGT
CAGTGTCTCTGCAGCAGATTGTAATGCATCGCCTTCCTCCTCTTTGACAGG
AGGATCTAGGTTTACAGGACTATCTTCTGAAATTTCAATGATCTCACAGTC
TTTATCAGAATTTTCTTCTTCATTCTTAACATCTGTATCTGAAGATTGACAC
AAGTCAGTGTGTTGGTTATTTTTCATAGAAAGATCAATTTCCAAATACTTT
GACAAAGCTTCTGTGCATTTTTCCACAATGTGAACCATCTGAAGGTAACTG
GCAGCAGTCAAGTATTTCAAAAGCTCTTTTCTTTTTACTTCAAGTGCTCCA
GTATAGCAGGACAACAGCAACTTCCTGCCAACTTCTGCACTCTGCAGAAT
GGTAATTCTGATGTGTTTTGACTGTGTGAGTAAAAACTGATCTCTCATAAA
AGTGGAGCAAGCAGCCAAAATCACCTTGTGCCCCTGGAACTCAGTATCGT
TAATATAAATCGATACATCACAAAATAAATTCTGCTGTCTCAAGAGATTC

ATTTTCTGTAGGACCACATCTCCTTGCTGTTCAAACTGGAAGTGTAGAACC
TCAGACTCAGCAGCCATGGTGACAATCTTTTATAGATTGATCTTGGTCCGT
TTTTCATACACCCAGCTCCTTAATGTTAAATTGCTCAACATTGTACAGATA
AAACACGGGAACACCCATTTGCCTATCTATTACATTCACGCTTCCAGCTCC
ATTTCCCTACTCCGTTAGCTCCGTTGAGCCCAAAACAGAGACTAACGTGAC
TCCTCCTCTCCCAGGGAATAATTTCCTCCTGATTCTGTGTTCACCCAGTGG
GCAAAGCGGCCCACACAGCAGGAGGAGCTGACGGGAATCTAGACCGCCT
GCCTG
>LNC_007228
TTCTTGTGTGCCTTGAAGACTTTGCCCTCCACCACCACGCACACATCACAC
AGGACTCCGTGCTGCCTCTGCTCATTGAGCTCTCGAAGCAGATGCCGGTA
GTGAGAAGTGATTTCCATATCTTCCTTTCGGTTGTTCATCTGGGAGTCTTG
CGGCTATGTCTTCTATAGACTCTGTGACCCCATGGGGCGCGGGCGCGGGC
TGGCGCCGTGGCCGCGGACCGGTGATCGCGCGGCCACTGGCGAGAGCTGC
GTCCGGGAGGGTCCGAGCTGCGCGCTCGCTCCGGTGGCTTGGTCCGCCCG
CCGGTCCGTTCGTCCGTCCGTCAGGCCGGC
>LNC_007234
TGGTGATCCACTGTGTGCTCCTGAGAAGGGGCTGGCAGCTGCTGTGCAGC
CTTCAGGCTATCGTGGTCTGCTGCTTTGACTCCCACGTCATTTTGATCCAA
GGACCAGGCAGGTGTGGACTTCACGGAGTGGAAAAGAAGCCAGGACCAG
TCAGCCCCATACACTAATGAGTTAGGCATCGTGTGGGAGGTTAGAACAGT
TATGTCCTGCTTCTCCTCGATGGCCTTCTGGCAGTTGAGGATCTTGAAGCC
GTTGTGCATGCAGGCCGCCAGGAGTAGATGGTGGTGGACCGG
>LNC_007286
TGGAGCTGCATAACGTGATACTGTAACTATGCCTCTGGGCAGAACTCGAG
CTGAGAGTCTAGTTAGAAGCCCAGGCTCTGGAGGGAGTTCAGAGCCCAGC
TCTCCAGTTCCTGGTGATGTGACCTTAAGACAGCTGCTTCACCCCTCCTCA
GGACCTGGAAAGTGAGAACGTGCTCCCTACCAGGTTGGGATGGGGTGGGG
AGGCTACCTCAGGGAAGGACATCTCAATCCCTTCCAGGACTAGCAAAAGG
ATCTCAGCTGATACCCTAGGGCAGAAGAAGGTTAATGAGGGAAAAGGAC
AATAAACGTGTTTGTCCTACTTTTATATGACACAGACACCTTCAGATGGAA
GATTCAAAGACCCAGGACAAGGCTGTGGATAGACGCTGCAATGCATGGA
GCCACACCAGTGGTAGTTATTCTGGAAAGTGAAATCTGGAAGATGGCATC
TGACTAAGGAGCCCCAGGTCCCATGGGTCAGCTGGCAGAGTCCAGAGCTT
GTTTCCACTGCTCTCTCCTCATGACCTGAGTGGTCTTTGGCCTCTCACTCTG
GCCCCCAGACTTACAGCCAAGACTTTCCCTGGAAAACCCCAAGCATCGTC
ACCTTCTTCCTCCTGATTCTGTGGGGGGGAGACAGAATTAGAGCTTGCCTC
ACGGTACGAAGTTGTGGCCACAGCTGCAGAAGAATGACTTTCAACTTCTA
GGAATCGACCCTGACTATCAGATAGGGCAAGGGTTGAGTCACTGGCAGAG
GCCACATTAGTGGAGATTTTGATCAGGTGTTTGCGTGTGTGTGAGTGTGTG
TGTGTGTGTGTGTGTGTGTGTGTGTGTATGCGCGCACACGCGTGCTCGTGC
CTCTGGTTCTACTTCCTCCTTGGGAGGCCCAGACCCTTCAGTGCTGTTTCTG
TGCTGAGATGGGAAGGGAGCACTCACCCCGCAGGTCCCAATCCAGGCCAA
CTGGGATGTGCTAAATTAGGTCCTAAAAAATCACAGCTGTGAGAACACGG

AATTTTGCTTTACAGATGATAAAAGTATTCAGAGAAATCAAGAGACTTGT
CTTATTCTCCTTAAAAAATAGTTGGGCCTTAAGCTCAGGGCTCCCAAAAGA
TCACCACACGACCCCCAATAAATGCTCTTTCCTAAGTCTGGAGTCTCGGCC
ACTGTCCTGAGGCTCAAATGATCATAGATAAAAGGGGGGAGGGCACAGTT
GGAACTGAAGTCAAAACCCATGTTTGAGCAGGTCCTGCCGGATGTAGTGG
TCATTCTTTGTGCTTCTGGATGGCTTTCACCATCTCTTTTCTAAGACCCAAC
TCCTCTGTGTGAACCGCTGGGGTCCATATCTGTCTCCTCTCTCTGATCTCTT
GATAACAGTTTGTTAGACTGGAAATGGCCATCTGTGCCAATTCAGCTTCCG
TCTCCTGTGCCCTGGGCATTGGGATACAAAGGGGCTGGGCTGCCTTCCTGG
CTGAGTATTTAAAGTGAGACCTAGGGCTTGGCCAGATTAAAATGGGACAG
CAGAACGAAGTCCAGGTCACAGAGAGGATGAAGTAGGCTTATAAGGGGA
AGGGGAAGGGTGAGAGACCATGGCATAGGCAACCATGGAGGAGAGGAAG
AGAGCTTAGGAAAGAGAGACAGGAAGATAGGATAAGGAACAGACAAAC
AAGCAGGCAGGTGTGTATTCCTGAACTAGGGAAAGATGAAGAGGAGATC
AAGGTATAGGGGCAGAGAAGAAATAAAATAAGGAGGGAGGGATAGACA
AAGAGACAAACACCATTAGTCTTTACTAGCCAATGACCCCTAAATCAGCA
ACAGCGACAGACTCTGGATTGAATATTTGAAAAAGAAAATCCCCCATCTG
TAGTGTACATATTCAACTTCTTTCTCTCCGTCATTTTGTTTGTTTTGTTTCTG
AGACAGGGCTTATCTGTATAGCCCTGGCTATCCTGAAACTCACTGTGTAGA
CCAGGCTGGCCTTGAACTCACAGAGATTTTACCTGCTTCTGCCTCCCAAGT
GTTGGAATTAAAGACATGTGCCACCATGGCCTGGCCATTATTTTCTAAACC
ACACAGCCTAACTACACTGCGCATGGAACCCACAGTGTATTGGGTGTGAG
CAGTCATCTTGAGGTGATTTGACAGTGTTCAGAAGGCTGGATGTGTGTCTG
TAATCCCAGCACTGTGGAGACCCGGAAAGGAGAGATAGTTCAGAATAGCT
AAGCTACATCTCAGGGTGGTGGTAGGACTTGGCATCCTCCACAAGGTGCT
GGTTTTGCAAACATGTAGAATGCGAGCAGCTATAGAGGCTTACACTATGG
TTCTGGGAAGACCCTGGGTCAAGCAGTGAGAATCCCTGCATGGGTCATAA
GCCGACAACTGTGCAGGTGTAACCTGTGTTTCCCAAGGAAAGCTGCCCTTT
TGCCTGATGGATTTTATCCCTCAAACCTTACGGTTAAATAACTCCTTTCTCC
CTTAAATTGTGTCTTGGTGGGGTTGATGGGATGTGTATGCCCCAACCCTGA
TCTCCAGAATCTTAGGCCCTGCCTCTGCCTGAGGCTGTCATTGGCCACAGG
GCTGTGCTCAGCTCTGGCTGGGGCGTACTAACAGTCCTGGAAGGGGCAGT
GCCCCAGAACTACCTCAGCCACTCAGTCTAGAATCAATGCAGTTCCATCC
GACCTCCCTCACTCCCAGAATTTTCTGGAGGGCTTAAACTCAAATGGCTCT
TTGGTCCATTCTCAGCTCCTCAGCACACTAGAGTTGCCCTCCTCCCAGCCC
CTGCTTCCTGCTAACACTTCTTGGGATCAACTCCTCAAACCAGCCAGCCAC
ACGTCCAGTTTTGTTCTGGAACAACTTCATCAATGACAGGGACACAATGG
ACAGGTGTTGTCAGAGGTACCACACAGATGAAAAATGTCAAGGTTCAGGC
TAGTTTCAAAGAGACAAGGG
>LNC_007300
TGTAAGTCAAATAAACCCTTTCCTTCCCCAGCTTGCTGTTTGACCAGTTGT
TTAATCAAAGCAACAGAAGCCAAATTAGGACAGTCCCTTTTCACAGTTGT
TGAAGTATGGCAATCAAAGTAATAAAACTGCTCCATAAAGTTTATTTTTAA
AATAACAATCACAAGAGAAGTTGGCAAAAATTTGGAGAAAATGGAATAA

ACATATACTGTTGGCTCTGTAAGTCAAATAAACCCTTTC
>LNC_007527
CGGTTTTGAAGACATGGTTTGTCTGACAAGACCTTTCGCAAGCTGAACACC
AAGATCGGGAAAGTCCTGTGGCTTTCGCTGGCGCTGCTGCCGATGACGCG
AGTCGCTCCCTTGCTGCTGGATCTGGGTGCCTGTCATTTGCCCTTTATCACC
TGAACAGTTTTCCGACCATAGTGATAATAACTGTATGCGGACGCAGCTGT
AGTGAATGCTGTACAACACCATAGTATCTGAAGATAAATGCTGTCAGCAT
AATTGAAAACTGGAGCTGCCAGAGAAGCTGCCACCAAAATTAACTGGACT
GCTGTATTGACCTTGCTGATGAATGTTGGTTTTAACCTAGCAGTGGCATAG
CAAGGATTGAAGTACTTAGCTAGTGTTCGCGGTGTTGGCAGAGTTCGGTA
TCTGACATAAAACACAGCAGCGATCAACATTACATCTCTTGAAATTATCAT
GTAAGTGAGTGGGACTGGAATAAGATCTGCATAGGTCAGGCTAATATATA
AGATGCTGATAAGAACCTTATCAGCAAGTGGATCAAGAGCACTTCCCAAA
GCTGATTTTTGATTGGCCCAGTTTCGAGCAATAAATCCATCCAACAAATCC
GTTAGCCCAGCTAAAGCAAAAACACCCAGTGCAACATTAAAATCTTCTTC
AAGAATCAGATAGCCCAACACCGGGGCCAGGCCAATTCTTGTCATTGACA
ATAAATTTGGGATTGTCCATGGATTTTCATACGAGCTGGTTGCGCTCGCCC
GGACCCACCGGGCACTAGGGGCCTGCGCGGCGGCATCTCCTCCGGCTGCG
GGCTCCGGGGCTGCCTTCCCGGCGCCGGAGCAGTGGGTCCGCGGGCTGGT
GCCTGGCAGCCGCAAGGCGAACGCGGCCGGACGCAGCCGCCAGCGCTCG
GCCAGGCAGCCCAGGCAGCAGGCCGCGGGTGGTAGCAGGGCCCTGCGGG
AGCCGCCCCTGCCGAGCCGCGCCCCTGGAGGCCGGACGGCCACGCGAAG
GGACCCCCACGCGCCGCGTGCCACGCGCCAAGCCAGCATGGCCTTGCAGC
CTTCTCGGAGTCCAAGAATCGAGAACTCAGAAGCCACTGGCACCAGCCGC
CAGCTTCTCTATAGAAATCGAGCGGTTCCAAGGCCGTACGGATAGCTCCA
TACTAATTTCCACACACTGGCACTTTCCGCTTATAC
>LNC_007633
AAGTTTTAGAAAGCACCAAAATCCTGTCCTTGACACTTTTAGATACACATT
TTGGGTTTTTATTGCTGATTACAGACTATGAAATGTGCATTGCAAGTCAAC
AAGAGATTCTTTTCATATTTCCAATAAAAGCTTAAAGAAACAGACAACAA
ACACAAAGACAAGAGAAATACAAGCTTCAAAACAAAACAACAGAAATAA
GTCTCCACAGTCACGGTCTGACAAAAATTTGAAATAACCCAAACCGTGCA
AATGCTCCACAGAGACCATGGCCATACATGGGGTTGTAAATACTTGTCCC
ATTAAGGACAAACCAGACAAGTTATCAGTTATATTGGTGATCAAGACAAA
CCTCATTGGCTTACAGCAGTATTTACTGTCCTCTGTGAGAGTCTGACAAGT
TCAAGAGGTTGGAAACCTGTGATGCTTCAGCAGGGCCGTGCTCAGAAGTC
CACATGGTTCCTAAGAAACCATCCTCAGCCCCCCATGGGTCAGGAAGATG
CAGTTCACAACAGGGTCACACTGGCTGCCTGCAGGTAACCTTGGAGGTAC
TGCAAGGCTTCTGCATTGAGGCTGGTGCTCACCATCGAGGGAACCCTTCCC
GGACAGGCAGTAGACAGTCTGTGAAGTGCCTGTGCCAGATGGACCCTGGG
GTTGTTCACCGTCTGACCCACAGGGTCATACTCCTTTTTACCAGCAAACGC
CAGCTGTGAGAAGGCAGTCTGGTACCCTGGGGTGTCTTCTATGTCAATGA
AGTGCTCTTCGTCAGGGATGCTGTCATCTTCAGGTAACTCAAACAGGCCA
ATGAGAGACTGTAACAACGGAGTCCTGGATGGGAAGGCACAGACAAAAC

CAAAGCAAATTACTTTTAAGGGCCAACAATGGAGGCTTATACCTGAAATC
TCAGATCTCATGAAGTTGAGACATGAAGACTGCTGTGGGTTCCAGACCAG
CTGATGCTACTCAGTGACAATCTGTCTCAAAATACAATGTCTAGGGTTCTG
CATTACTGAACAAAATGCCTGCCATGCAAGGTTTCCTAGAATCCATAATCC
TAGAATCAGCAAGTTCCAGGTTCAGTGAGATCAAGCCTCAAAAAAGTAAA
GTGCGACTGAGGAAGACTTGTGACAATGAGCCCACCCGCCCACACATGTC
ACATGCCAAAACACATGTGAACACAAACATGTATGCATACAAAAAGACA
AAGCCCTAAGAGACATTCTGTGACAGGGGGCACACCTGCCATCACAGGAG
GTAAGTCTGTATCAGGTCAGGCTCTCAGAGGGAATGGATGGATGGAAGGA
TGAAGGATACGTAAATCCTCCCCTTCAAATCAAGAGTAAAGACAGTGCAG
TGTGGGTGCATGTCTGCAATCCCAGTACTGGGAGGCAGAGGCAGGCACAG
GTCATGTCTAGCCTGTACTACATAGCAAGTTACAGGACAGTCACAACTGT
AGAGAAACAAAACAAGGGCTGGAGAGATGGCTCAGCGGTTAAGAGCACC
GACTGCTCTTCCAG
>LNC_007651
TATAAATCCTTGAAGGACTTCTTGCTTTGTTTTCAGCTGGGAAGATAAAGA
GGCATTGCCTCTACACTGATAAATTTTTGGTACCAAACTTCTTAAAAACCA
GATGGTTGAAAAAAATTTCCCAAAAATTACTCAACATGCTGCTCAGACAG
GAGTGCTAACAGAGAGCATGCACACGTCACCCAGCGTCAGCGAAGCCTGT
GCCTGCCACTCCGTTCACAGCACAGCTCGTTGCAGGCCAGTCTCTTCGACT
CTGGACAGCGAAGGAAAACACGGCTGTGCCTGCACACCAGTCTGCAGTTA
GAGGGAGCCCAGGGGAGACCCACTGCCCACTGTGGCCAGGAGGCTGTGCT
CTGTAGACAGTTCAGTGGTGGGGCGCCAGCCGAGCCCAGCGCCCAGCTAC
TCCATGCAGCAGCGCTCTCAAGAACTCAGTGCCACTAGCGAGACTTAACA
GCGATGAGGACCACAATGAGGACGACGATGACGGCCACAAGTATCAAGA
TCACAAGCATCTTCCTGTTCTTCTTCTGGTACTGCTCTGCCTTGTGCAGTTG
CTTCAAGCCATCTTCGGTCTTAACACAGGATTGTTCAACATTATAGTCAAT
TCGATCAAGGACTGTGCCCTGCTCCACGATCATGGCGCCCAAGTCCCTGA
AGATTTCGTTGAGGTCTGAAATGGACTGTACAATCTGACGGATCTCTCGCT
CCCTCTCCTCCACCACCAGTGTGTTCTGCTCCACCAGCACCAGCTGGTCAT
CCGTGAAACCCTGACCATACAGAGTAGCATCATCTCCATCATCCATTAGTG
GTACCGGGGTGTCGAAGAAATGCTGCGATCTTTCCTCTCGGTTTTTCATGC
GTTTCAGGTAGTCAGACTGTGCATGCCGGAAGCTGGTAGACAGCTCCTGC
AGGGCCTGTGCCAGGGAGGCCACCACATTCCGCAGTAGCCGCTCTTCCTG
CTCCGAGCACGTCCTTCGTGCCCGGCTGGGCAGGGCCTGCACAGCACGCT
GGCACCTATGGAAGAGCTGTGTGACCTCCTGGGTAGTGATCTCGATGGCA
TGTTCCTCCTCGCTGCTGTCGTCCAGCGTGGGTCTGTTCAAATGCTTATCAT
GAAGGCTGGCTAACTCCCTCATCTTCTGTTTAATGCGGCCAACATCATACT
GTATTTCGTCTACTCCGTCCACCCACTTTGGAGGGGACCGCTTTGTCACGC
CAATTGCTGCTTCTGGATCTAGGCTGATGCCTGACACCAGCGCCATGCGGT
CATCAGCCAGTTGCTCGGCTAACAGCTGCCGGTTTTGGATGGAATTATTCC
GCAACAACAAGAAAGCGTCGGTTAAACGCCTGGTGGCCATGTCTCACCCT
TTGTGACCACCCTCTCAGGGTCCCCCACCCCCTTATAGTCCTGATTTTTCAC
TTTCTTTCTCTTGGTCTGGCCTAGCTGGTCCACTCTTGCATAGAAACCTCTC

TTGACAAGGCCTTAGGCTTTTAAGGCACAATTTTGCGCTCCACCAAAGCGC
AAAGACCCCTCCCCAAATAACCACCTAGGCTTTCCAATTGTCTGACTCCCC
CTACCCAAGGCCTGGCTCAGATCTCAAAGCACTTGAATTCCCCCAATCCTT
GCGTAGAAATCTAACGTCCTGGAGACCCCTCCCCAATCCAACCTTGGATCT
ACAAGCGTCTAACGCGCCAATCCGGCTAGGGGATGGAAAAGACCCCTACC
CACTCATTTCCTAAGACCTCCCCAAAGGTGCTGGCGCCCTAACCCTAATGT
GTCTGGCATGTGGCTCTAACTTGGGAGTTTGGGTTAACTCTCCTCACAAAA
GGCCTGCTTCAAACAGGCCAGCCCAGGACTACAAAAAGCGTCTACAGCTC
CCATATGGCGACTGAGTCTATTATGGCTCAGGTCGACCTAACCCCTTTCTT
TCCTGGGTGGCCTGGTTCTCCACTGCCAGCAGGCTGTAGGCCTTCAATAAG
GACCTCACGGCCTGCCTCCCCGGCCTCCTCACTCCTCTTCAATCCTCTTCAC
CGGCAACCCTCCATCCCGGGATCTGGCTGCAACCCTGCCCAAGCCGGCTT
CCG
>LNC_007717
CAGTGCGTTTAAGACTAAAGACAGAGAGCACACATAGTCCTTCTTTTGTCT
TTAACCATGACTGGATCTCATATGACATTGGAAAAGCATACAAAAGCCTG
GTTAATTATCAAGTTGACAGCATGTGATATCTTCTGGAGGAACTTTAACAC
TTACTCCTTTCTTTTTCACTCACCTCTCCACAGTCTGACTCTGACAATGACT
CAGATGACAAAGGTTTATCCTCATACATCTTTCTCTTTTTGCTGAGTCCTTT
AGTGTCCTTGCTGTCTGAGTCTGAATCCGACACGGAGGTCTCCGGAGACTT
ATGGCAATGGCTCTTCTTTTTCTTCCTTCTTTTTGTTTGCTTTTTATCCTCCT
CCTCAGAATCTGAAGAACTGCTGGAAGAATCAGAGCTCGATGAAGAAGA
AGATGAATACTTGACCAAGCACAAAACAATTAAAGCTTGTGAGATGCAGG
AATTGGTTTCTTTTACTAAGTCTCTCAAGCACTTAGTGAAATCAATCTAGT
GTCTAAGAAACAGATGCCATATTCACAGGATTTAGAAACTAAAATATCAA
AGCCATTTCCCT
>LNC_007798
CTAGTATCAATGGTAGGTTTTCTGTGTTCTATGTACAAGAGTTAGGGCTGA
GTAGAACGGGGGTTAATTTACAACTGGTACAATTATTTAGTAGCTAGAAG
TGCTCAAGTCTCTTTGGGGTTCCAGTGTTCGGATCAGTGTATTCTGTCTGA
AATGGGCGGCCATGTTTCCCACTCATTATTTCTCTTCATTTACTTTCTCTCT
TCCAGAAATTTGTTGAGATCTTCTCCAACCAAATGCTTTTAGTTTCCTTTGC
TGAGAGGTCTCAGTTTTCCTGGACTGTATTCTTGGACTTCAGTTTCAGTGA
GGCCTCCCCAAACCTCTCTATCTGCCACCTCGATGGGCACTCCTTACAACT
GTGTGAGAAGATACAATTTCATTATATTTTCTTTCCATTCAGGCAGATAGG
CTAAGCTTGTAGCCACTTCTTTCTGGATAGGCCAGGCCCAGGCCTCAAAG
AACGGAGCCAGATTCTTCTGCACTTGCTGGGAGAACATCTTGACCCAGAG
ATTCATTTTGTTGACGTTGTCTGTGGGCGAGTTCGTTGTCTGGTTTCTGTAC
TCAGTGAAGAGACGAATAAATGGCTCCCAACCAAAGGCCTCTTGAAGCTG
TAGGTACGTTTCCAACGCAGTCCAGGCATTCCAGTTTTTCACATTGGGTCC
CTTGCTTAAATACATTCGGACTCTCTTCTCCCGAACAGGGGGCCATAGCGC
AATGTTGGCACGGCTTCGAGGTATGCCCAGAACTGTTTCATGCACATAAA
CACACCACAGGTTGCATGTGGCTTCCGTAGTATGTGGGGGGAACTCCCAC
TCCTGCCTCTGCTGGTTGCGGCCCAGCTCATGCACAGGACCCCACAGTCCC

TTGGTCCGGATGAGCTTCTCACTAATGAGCTCCTGCACTGACTCCAGATGG
CACATGATGGGGTACCCTGCATGCATCCAGCCTACTGAGATCTGAACATC
AGCCACTATCCTCTGTGGCAGCCGCAAAGGGAAGGGCTCGCCTCCCAACT
TAGCCACAGCCTGCATCACCTCATCCCAGAGGCGGAGCAGTGGCTCAGGG
TTCTCCAGAGTTCGAAGATTTGCAGTAGGCACAGTCAAGATGAGGTTGTC
AGTGGCCAGTTCTCCCCACGGACCTGGATATTCCAGGAGACGCCTCTTCCA
CTCTTCCTTGGATGTCTCCCCTAGCTTGTAGAATGGGGCACGAACGGCTCC
CTTCACTGTGAGGGGCACAGTACCCAGTTTGCTATTCTGAGGCACAATGAT
ATAGAGGAGACCACCCCAGAGGCAGTTGATTGATTTCATGGGTTTATCCA
AGCAACACCGGTTAATCACAAGGGGGCCTCGGAAGAGCTTGCTGGCCCTG
GTCAGGTCATCAGTGTGACAGCCAATTTGTATCTTCAGATCAGCAGAGGC
AGCATCTTCAGGCAAGGAAACATCTATGACTTGCCTTCCAGGTATGTAGA
GTCCAGTGCTCATCCAGCAGTATCGGGTGCCTGGATTGGTGCAGTTGACAT
CCACGGTGATGGGAGACTCTGAGGGGCGTAGGTAGCAGCTGCTGTACACA
TCTTCAATTTCTGGGATTAGCAGTGAGAGGTCACTTCCAGAGTGGGCCAG
ACCTGTGGCCAGGGAAAGCATGGCCCCCCTACAACAGTCATTGATGACCG
GGTTCTCTCTGGTTGCCACTGGAAGCCGGTAGCGACTCAGCAGCTTCCGCA
GGAGTCTGTGCACGGACATGTAAGCAGGGATCTCCTCTGCAGGAATCTGC
AGGAAAGCTGCCCCATCTGGGCCCAGCTTGGCTAGCCAGCCCTTCTCCAC
ATTTCCTCTTTTCCTGCCCATTATCACCTGGAACTCTGCCAGGGTAGAGCG
GAAGTGATAGGTCCTTGTTCCTGCTTTTGGGATATGGAAAGGTCCTGGATT
GAGGCTTTGGCTTGTGATGCTGATACCAAAGGCATTGAGGAGCAGGTTTC
CTGGGAATCGAGCCAGTGGGGACACTCCTGGGTTCTTAAAAGCCCACCAC
CAGGCTTGGGTTCCAACAAACAGCCCACCACCCTCAGCTACAAACTCCTG
TAGTTCCTTAACTCCCACATCACTTGTGGGTTCGAAGCAATAGACACTTGC
GTCACTGGTCAGTTGGGGCTCAATGCTGGTGTCTATACCCCCTACTGCAAG
CAGACCACTTAGTGTTCTCAGCTCGGTCTGTACCACAATCTTGCCTCTGCG
GCCGCCATCTAGCCAGCGGACAGCATTGAGCAGAAAGGGGCCGAGTTTAC
CCACAGTGAATAATACCTTATGACCAGTTACAACCACCCGTCCCCGGCCA
TAGCGGGCAGCAGCTATCACACAGCCATGGTGGGTATCTAGCCCCAAAGG
AAAAGCCAAAGACCCATGCACTAGTAGCTGGGATGGGAAGCAGTCCGAG
TTGGTGATATCCAGGTCTGAGATGCCATGTAGAAGCTCATCTCTGTCTTCT
GAGAGGTCATCTTCACAACGAACTAAGATGGGTATCTTAGGCATCTTCTTG
GAGACTTTATAGCAATTCGTGTCCGCTTTGTTGTCAGTGAAGTACACACCA
GCCACACTGGTCACCAGATTCCCAGGAAAGGAGAACAGTACTCTATCATC
TTCACCCAGGTTATCCCAATCCCAGGCTTGGCCTCCTATGAGCAAGCCCCC
GCCACGTTTCACAAACTGGAGTAGCTTATCCGTCATGGTTTCATTGTAGGC
ATCGATGCAGTAAACCCCCAGAGAGTCATTCACTTCTGGTTCAATCTTTGA
CTCTATTCCACAGCTCTCGAGGATTTTCACCAATGGTGCTAAAGATGAGTG
CACACCAATGGTTGACTCAGAAGAGGAACGAAGCCATCCAACAGCATTGA
CAAGAAAGACAAAGAGCTGGCTTTCCACCAAGAAGTCCTCATGAGATGCT
ACCACCATGCGACCTCTGCCATAGGAAGAGGCAGCAATGAGGACCTGGCC
TACATCATTCACCATTATAGGAAAAGAAGCCTCTCCAATAAGAAGCAGTT
CACATGGAACGGAATCATCAGGGATATCCCAACTTGTTACTCCATTCATA

AGCGCCTCAAAGGTAGCAGATGGAGTTGCCATGGTTCTGTTGGTTCCAGG
CAGCTGTCTTGGCGCTTCTAGGACCGCACCAGGAGGGCTGAACAGGGGCT
TCTCTGTGGAGCACCAAGGCGCCCCCGAGCAGCCACCGAAAGCTTGGAGC
TTAACGGGGAGGAGATCCG
>LNC_007828
CATCCCTCCCCCTTCTTCCTTCCCTCCCCTCTTTTCATTCATCAAATTCTCCC
ACTGTTCATTTGGCTCCAATTTCCAAAACGTGCTTAACCACAGAAAGCTCA
ACAGACCACTGTGGCAGGACATAGCAGGAAGTCAGAGCTACAGGAACCA
CTCTGCAGGAACCACTGTGATCCATGAAGGGGCAGCCACACCCTGGAGCA
CATCTCTGTAGAAGCTCGTGCTTAGAGAGTTTCCAGTCGTCGTTGAGATCC
ATTTCTTGAAATGATTCGTGGGACCTTGGGCCATTTCGAATTTCCAGGAGG
TCAATGTTGAATATCAGTGTGCTCTCTGGGGGTATTTTACCTTTTCCTTCTT
TGCCATAGCCCAGGGCAGGAGGAATGGTGAGCTTTCGCTTCTCTCCCACA
CACATTCCCTTCAAGCCTTGGTCCCAGCCTTTGAGAACCTCCAGTATGCCC
AGGGTGAACCAGACGGGCTGGCCATTGTTATGTTTGTGAGTGGAATGAAA
CAGGGAGCCATCCTTTTCTAAATAGCCTTCGTAGTGGACCAACATCAGATC
CCCTCCCTTGGTCTTGCGATGGCAGATGAATGGTTTCTGAAGAACTTCGAT
CTTCACTTCTGGCTCGGGGATCAGAGCTCCACTCAAAACCGTGACCCACA
GTGTCAAGTTCGCATTCCACAAGAAAAACCTCATGTTACCAAAGCAGAGG
AAAGGCCTCCGACTTCATAGAGTTAAAAATGTGCCGAAAGCAGACGACAG
CTTAGGACAAGTTCAGGACTCCCCCAACAGACACCCTCCGAGGAAGACGT
GGCACTTTACTACGAACTTT
>LNC_007922
GCTTGTTACATAGTCTGATTGTATCTAAATTCTTACCTCACTGCTCTTCTTT
TTGATGATTGACTTTGCTCTTCCTGAATGTTTTCACCTTCAAGTATTAGTCC
GTTAGTTACACAGGGCAGTTCCTATCTGGTGGCAGTTGGTGCTGAATTTAA
TGAAACTGGAAATGTTGACATCTGTATCATGTTTAATCCTCCGACCCAGGA
GCACAATCTATTTATTTTATTTATTCATACAGTTCCCATTAAAATGCTCCGC
TCCATTTTGATGGCGTTACCCCAGCTGTGGTGTTGAGTGGCTGTCATGATT
GACACCTGTCTTTGTGTATTATAACTCACCATAGCTGATAGCTGGGAGGCT
TGGTGCAGGCAGGCAGCCCCTTAATGGATTTGATTTGTTTTGTTTTGTGAC
CTAGTTTTTCTTTAAGGGTTGTTAGTTCAATTTTCTAGAACACAGTGTATTG
TTTGACAGCGTTGCAGGGTAAATTCTTACTCTTCTTACCCCATTCCTTACCT
TGCCATCCCTATCTGCTGTGGCCATCTTGGATTACTTTGTGTAGTTAAAAA
TAAACATTACTTTAAATCACTATTATCACATTATTATTTATTAATAAACAG
GAATAAAATTATCCATGGAGTTTTTAAACCACAGTCTCAGAAAAGGCTAC
AATCGGAGTCAGAGTTTGTTTTTTTCTGACCCTG
>LNC_008004
ATGTCTCTTCCCCCAGATCCACTCCTCCATTTCCCTTCAGGAAAGAACAGT
CTTCCAAGTGATATCAACTGAACTCAGTATAACAAGACACAGTAAGACTA
GGCTTACACCCTCCTGTCAATGCTGGACAAGGCACAGGAGGAGGAAAAG
GGTCCCAAGAACAGGCAAGAGTCAGAGATGCCTCCACTTGCATTGTCAGG
AATCTCACAGAAACCCCAAGCTAAGGAATCACAGCATGTATGTAGAAGGC
CTGGTGCAGAACCTATGCAGGCTCCATGGTTGCTGCTTCAGTCTATGTGAG

CCCCTCTGAGCCCTGCTTAGTTGATTCTTTGTTCACATGGTATTTTGGACTC
CTTTGGTTCCTACAATTCTTCCTCCTCCACTTGGAGTTCTTGAGTGTTTGGC
TGTGGGTCTCAAGGGTCTCTGTATCTGTCCCTGTCAGCTCAGGGAGAAGCC
TCTCTGATGCCAATTTGGTTAGGCATTGATCTATGAATACAGAGAATTTTC
TTTGTCAGTTGTGTTTGGTTCCACCCTAGGTCTCAGCCATCTAGTTTCTGGA
TCCTGGCCATCTAGATAGTGTCGAGCATGAGTGTTCCCTCTGATGGTGTGG
ACCTTAGGTTAGACCAGTCATTGGTTGGCCACTCCTACAAGCTCTGGTCCA
ACCATCTTGCAGACAGGGCAGGTTGTGGGCCGTAGATTTTGTGGCTTGGTT
GGTATCCCAGTCCTACTACTATGAGCCTTGCTTGGTTACAGAAGATTGCTG
ATTCAGTAAAACCTACTCTCAACACAGTTCTATGATCTTTATTGCTGGATG
ACAATAAATTGAGTAATCAGTCTTCTAAGCGTAGACTCCATTTTCAGAGGC
TTACTTTTCATTTCTTCCTCTGAAATGCTGTATCTGCCCTGGGAAGTTCCTG
TACCTTTTGTGAACACTCCTTTTACCTAAAACTATTGAACTGTTCATCACA
GAATTTTCCTCATCCTTCGACCCATTCTAAAGCTATGCTGTCTCCATCCCAT
TATTGCAAAAAACACTTATGTGAACTTCTGCACAACTCTTCCCTCTGTGTC
ACTTAGAGGGTACTTGCCATTTTAGTTATCTATGTATGTGTAGAGACTTAC
TCTCAGTCGCTGCCGGATTCCTGCATAGGAAAACAATGGCTGCATTCCTTG
GGTTCTTAGGGGTTTGTTTTTCCTAGATTTCAAGCAAATTACAAATTACAT
TTCTTATGTTTGTTAATTAGCACAGCTCCCCGATAATGATGATCACCTTGA
ATTAAGCAGCATCACTGCCTAAATTTGTGTCCTGGACCTGACCTGACCTGT
ATTTGAGAGCAGTATTTAGTGTCCTTGGGTATTGTCACACCTGCTACTATT
AATCACATATTGTTATTTGACTATCAAACTTGAGAAGATATGGCGGTTATT
GTCAGAGAGCTGTCTCTGCTTATTCTTTGTGTTCAAGGTCCTTCAAGGATG
TTGATATTATCAGATAAATGACATGATTGGGCTTGCTTTAAAAAAAGGTTG
TGCAAAAGAATTTAATCTGCATTGCTTAATTGACTGTTTTCCAGTGTTATC
CGATCCAAATTTTTGAGTGAAAATATTTAGATTCTAGAGTTCCTGGCTGTT
TTCTATGGCCCCGTATTGTACACCTAAAACCGTTCGAGTACCTCACACATA
TTCATGCTATTGTTTGCAGTTAGTAATTGGGTAGAGCGTCTGCACTGGTTT
ATAAGCACAATCATTTTTTATGTGATGGCGAATAATGTATGACTAAATATG
TGAGTTTTAGGCCAGCTTTGGATTATAACACACACACACACACACATACTC
ACTCATGCACATATATACATACGCACAGATGCACAAACACCACTGGTATA
TACTTTCACTGTTTTGAGAAAAAAGAAGTAAAATTCTGATACCTTTGTTCT
AAATGGCTTTAGGATCATTTTAAAATATGAGAAATATGAGGATACATATT
GCAGGAAGACAACATAATCTACAAGCATACATCAAGATATAGTGATGTGA
TTGCAAGAACTAGATTTCCAAACCAACTGGCCATTGAGGTCTATGCTAGG
TCACCATATATTCTACCAGGGCTGGGCTTTCAATCTCACTGTAATTACAAG
TGAAGGCAAAAACTTATGCAGCCTGTTGTCAGCCTTACTGACTTCTGGTGC
CGAGTAAATGAAAATAAACGGGCTCTCAAGAAGGGGGCATACTCTTCTGG
AAGTAAGAAATTGCTTCCATGACTCTTTAAAGGGGGGATATTGTTTTAGAT
CTAGGATTGATTCTTACTATTAATACAAACCAGCAGTAGCTACTTTAAATT
GGAAAACTTGAAGAGTTAATTAACAGTGTTTATGGTAGATTTAGTGATTTT
TACATTTAGTAGATAATATGCATGTGCCTTCCACATAGACAGTATGTGATA
ATTCTGTCAGATTTCACAGAATTGACATTCCTAGATTCCAATATCTGCTCT
CAATTCAACATGAGCTCATTGTTTTATCCCCACACGCATCAGGAGTCTTCA

CCTTAACCTGGCACTTAAAATAGTCACTAGTTTTATCCTGTTGTTGTATCTT
TTTCAAAATTATTATGGTAATAGTGCAAGCTTATGGAATGTTGGTGATAAT
TCAAAGCATTCACGCAATATGTAGTAAAAAATCAGGACAATTAACACTTC
ATCTCCTTAAACATTTTTTAAACTATGTCATTACATAAATATATGACATGG
GTGTATACACACACACACACACACACACACACACACACACACCTGGCTGA
ATTTGTTTTGTTGTTAGTATGTGTAAGAATACCAATCAAAGAAAAAGAGG
CTATCAATTTGAGATTTAGGAAGACATGGGAGAGGTGGAGGAAAGAAAG
GAAATAGGAACTTTTAATTGAACATTTTATTTCGATTAAAAACATTAAAAA
TGCTTTGACTGATACCAAACATTAAAGAAAGGTTTTGACTGTACCAAAAT
GGAGTATTCTGCAAAACAGTGGTTTAACTCATTCATAATAGCTATCATGGT
TTTTCTGAATAACTATACAAGAGTGTTAAGAAGACAAAAATGGTGTTAGG
GATCTAGAGAAATTCCTTTCACTTGGTCGATCAGCACACAGATACCCAGG
AGTATCAGCTTGTACCCTGATGTTGCTTTGACCACTGCGTGCCATCTCCAA
CCCCATACGTTCCTGTGTTCTAACTCAGCAACTTGGTTTCTCATAGTTTTGC
CTAGGCATTCTTTGCATCCACCTGCCTTTACTCTGCAAACCACCAGTGCTT
TCTGTGGTCCCTTCCAGCTCTAGCCAATGTCTGGTTAAGTCGATGAATGCT
TCTCGGTTCACATTGCTCAAATGACTGTTAATTCTGGGCTTCTCTGCATTCT
TCTTCATTCTTCTTCTCTTTGTTCCACCTGAAACTCGTCTGGATTTTACATG
TAACCTACCCTAAGCACACTGTCACTTATGTACTTGTATGCATTTTCTTTTG
TCCTCATTACAATTTGTTTTGAACACACACACACACACACACACACACACA
CACACTACTTTCATTTTTTTTTATACTCCAGTCACTATCCCCTTCCTGGTT
CACCTTCTGACTGATCCTCATTTCATGC
>LNC_008030
TGGTCCCCTTTCCTCTTTTGGGTGGCCCACTATCCATCAAGGCACTAAGTA
CACATCAAATCCAAAGAGTGATAATGACCTGTATGGTGTTTGACAGCTTA
ATATCCACCTTGTAGGAGAATGTTTGTTATATTATAATTTGTTGTTCACTAT
CATTTAAATCGGCAAAGTAAGACAGACTTCAGAGTATTTAAGGAAACTCA
CATTTGTGTGTTTGATGGGAGGAAGAGTGTGTAAATAAGAGACCACCATT
GGGTCCTCTCAGAGGTAAACCAGTCCTGTAGGCAATTCAGAAGATTTTAG
GCTGGATTTTTATAATGTTCTCCACCCAAGAACAAATTTCAAGGAACTCCA
TGAGCCGTTCTACTGGAGTCAAGAGTTCTACTAAGCACTGTTCACAGAAG
TGCACACCCAAAGCAGTAGGAAAGGCCATAGAATAAGAGGCAAGACATT
ACTCCTGTGGTTTCTTTCTTTACTAAGGGCCTAGAAAATGGCCTATGAAAA
ACCTTTTACTAGAACAACTGTGTATGCTCTGTTTCCTTCCTTCCTTCTTCCT
TTTTCTTCCTTCCTTCCTTCCTAACTCCCTCCCTCCAGCCCTTCCTTCCTTCT
GTCCATGAAACAAGCTAGCACTGTGGTACACTAACTGACCTTTAAACCTT
ACATTCAAATGATCCTCCTGCCTCAACTCCCAAGCTTCCTGCCAGGAGTAG
TTAGGATTCCAGGTGTGCAATATGACGCTCAGCTGCTTTGCAACCAAAAG
CCTTCCTCGGAGGTCTTCCAAAGCTTCATGTTCATGTAAGATCTTAGCATA
TGCTCTTTCCTTCGCCAAAACCTTCTCGAGCTCAGTTTTCTGGCTTAAAGA
GTCCTTTACTTCCCTGAGAAATGGGCTCAGGGTTCATTGTGCTTTACAATA
CTTACTAGGTGTGTAGATGATCATTTTTATGTATCACCATTTGGTTAATATT
GGTGTGTACTAGGAGGCAGAACCTTCCGAAGCTAGACCTCAAGCTGATTT
TACTCAAAGGAACAGTTCGTAGGTATTGTGCTATGTCAGCTTACATGTTGA

GTGTGTATTGGATGGTTGCTGAGTGAGTAACTGACTGGCAAGAATTATTA
CTGAGGAAACAACATTGATCCAAGACATGGTGCTGGACAGAAAGCAATCC
TCTGCATTGACAAGGTCAATAGTCCTTTGACAGGGACTAGATGAGCCCCT
CATTAGGAATTTCCATCTTGAAAACTACTGGAGTGATTGTTGTTGCAATGT
TTTAAACATTGTTATGTATGTAAAAGCATTCATTCATTAATCCTGAACAAA
GCTGTGGTATGTTTGTTCCTCTTAGCTCTTCCCTGCTAAAATTCTTAGTTTT
CTGCATCTCTACTCTCATCACAATAGGGTTTTCTTTTCTTTCTTTCTTTTTTC
TTGCATCTGGTTCTTCCTGGGTCTCATGTGAAAGTTGTTACACCTTTCTGAC
CAAGCTATGAGTTATCATATGAGGC
>LNC_008031
CACCTGGAGCATGGAGGCTGATATTAACTTTATTTGTTTTGATAAAATTTA
AAATTTTGCAAGTAAATTTCTGTAACTGTAATTTTCTATCTTGCCATATATT
ATTGATGGTAGATGTTTTGGCCTGATGTTTCACCTTAAGTATAACATGATC
GATAATATATTTTCCCTTCCCAAATGACTTCTAACTTAACTTTCAGAAATA
CCACAATTGAAAGGAACTTGAGTCACCAACACATGACTCCTTATGTGAAA
AAAATTGTCCCAGGAAACCTTTGGTTTGGGGAAAACAATGTACCAATTCC
ATTAACTAGATATACTTCTTGAAATGTATTACTTTTTATGTCAACTTAAGA
AGTTTTTACAAAAATTTCAGCTCTTTTCATGTTTCTTGGGAAAATGTGGAT
GAGTTGGTGTAATTTAATTTTTCGGCAATATATAGGAGCCAAGAGATTTGA
AGTAAAGAGAACGCCAAAGCTCTCATATGGGAAATTTGAGATATTTGGAG
GAACATTAGGAAGTAGAATTTTGAAGTTGCTTTATTGGTGGTTTTTGTAGT
TCCTTTATCTAAATCTCAGGCAACTGCTTTACTTTTTAGACTGGAAAATAT
GTAAGACATTGTGCCAACCAATCTGCTTGATTAAATCAGATACCCCCAAA
GCCAATCTCACAAGATTCCTGTGGAAATTGCTGTCACTCCACTCACATGCT
ATTTTCCTTTGCTTCTTCAAAAACTAACAATAATAATACCACATTTTTAGA
GCCTTTAGGGGACTCAGCAAATAGCATCCATTTTCTGTGGCTGCAATCTAA
AGCAAATGCATTAAGAAGAAAATGCTAAGGCAAATGGGAACCAAAATCC
CACCATCATTGCTTACAAATCTCACGTTGAAAGGCATGGTCCACACTTAGG
GGTCTTAAAGACCGAGAACTGTCTTTCTTGTCAGGGACAGGTACCCACTTT
CAGAGAGCCTTTTCAGAGTGTCAATCTAATTAAATCATCATTGGCGAGGA
GCAGTGTTAATCTAATAGTGGATGGTGTGGAGGGTTCTTCCTAAAGAAAG
TAAGCTTAGGGTTAGGGATCAAGCTGTGCATCCATCATTATATCCTCTAAG
TTTAAAGACTAGATCATTGATGTAGATCACCTATCAAATACTAACATATCT
GCCCATTGTGATCCCTTTCTTCATGACATCGGTGTGAACTATGAATCACCA
AAGAGCACATGCCTGGCTGGACTCAGTAGCATAGCAACTTCCATTGATAA
ACTTGAAATTCATGATCCTAACATTCTGCTCGCTCCCACCCCCACCACTGT
TCTCACACCAAGTGGATCAGTGTCTTCTGTTGGAGTGATGTTTTGTACACT
CTGTGAAGACATGTGTCTGTCCTTCCTTGCCTGCCTAAGGGACCTTCTGAC
TGGTTAAAATAAAAGGCCTACAGATAAATATTAAAAGGCAGAATAGTAA
GATGAGACATCCAGCAGAGAGTAAATTGGAGGAAGAATCAGAGGTCAGA
GATTTGACACCTTGACTTGGAGGAAAGCGGAGATGTGAAACTGAGGAGA
GGGAACCAGCCACATGACGGACCATAGAATCATACAAGAGGGTTTACAG
GTTAGGAGCTAGCTGGAGAGCAAGCCTAAGCCAAGGCCAAAAACATTGT
AAAGAAATATAAGCCTCCTTGTCATTATTGGGGATGTGGGATAGGCATAG

TAAGGCTCAGAGTTACAGTTTTCACTATTGATATTAAACTGTACTGTTTAC
GATTAAGAAATATTACACCTTGAGCATGAGATTATATTGTACCATACTTAA
TCAACTGTAGCTATTGGTGTGCTCCAGAGAGAAGTTCCTTAGTCTGAAAA
ACATACCAGCCTTTAGTTGATTAAAGTTAAGGTTGTACAGTCTCTAACTAA
GACTCTTCCCCCATCCCTGGGTAGGTAGCCTCCATAGTACTAGGGCTATTA
TGCTGGGGCGAGAACACTGTGCTCTTACACCTTCAACACCAGCAATAAAA
TACTGTGTTGTTCACCCTAACTGTTTAAGTCTCCTTTGTTTATCACACAAAC
GTTTTATTTCCATGTAAAGTAGATCCCCCATCATATTCTTATGCTTTCTACT
GTTTTTCTGAATTTTCATACTCTCATCTGCCCTCTGACATTTATTATAAATG
TCATTATGTTTTGCTATTTTATTTTATCTTATTTTATTTTGGCATTGGAAATG
GAAGAGACTCATATAGCCCAGTCGCATAAGCCCCTACGCCACAGACCCAG
TTTAAGTTGTTCCTTGCCTGGGTTTCTTTTCTGATTCTTATGATAATGTGCT
CGATAATATGCTCTGAGACTGTCTAAGCTCCCAGGGGAGAAAGAGACCAT
TTAAGCTCACATTTCCAGGTTACTGTCCATCACAATAAGGGAGTCACAAC
AGCAGGGGCTTGAGATCACAACCAGAATGAGACAGGACCAGGGACAAAG
AGCAGTAACTTCATGCATGCCAGTACTCTCAGTGCAGTGTCTCCGGCCTAG
GGAATGATGCCTCCTGAAGTGCCAGATCTTCCAACCACAATTAACACAAT
CCAAACAATCTCCAGAGGCATGCCTGGAAGCCTGATTTAGACAACCTTGT
CCGGACAATCCTTAATTCAGGTTCTATGTTCAGGTGGCTGTAGATTGTGTC
AACTTGATAATTTGCAACAACCATCTCAGCCCTCTGGGAAATATTCATCAA
TGCTCCAGTTCGCTGCCTACTAGAATTTCTGTTTGTAGAAAAACTCTACGG
ACCTGTGTTTTCTGTTGGTTGGTTGGTTGATTGGTTGGTCAACTGATTGATT
AAATTTTGTTATTTTATTTTATTTTGACATATTAGCAATATGTAGCCCAGAC
TAACCTTGGAATGATACTTCTCAGACTGCCCACGAATCTGATCTTATTGCT
TCAGGTTTCTAACTCTGGAACTTAAGAGATGAGCCTCATATGATAAACACT
AATTTAAATGCAAATTCAGTGTACATCTATCGCTTAAGCTATTTTTCTATTC
CCTCAACTATTATTCATAGCTCTCTTTGTAGATCAAGGGACTTCAGTTCTG
GGACAGTAAGCCCCCAAGTTCCTAAGCTGTAAACGACAAGTGTCATGAGT
TAGTTTTCAGTCAGCTAGAACTCAGATGCACCCTTGATTCTTGTGTAATCT
GTAGCCTCAGCTTTTGTTAAGACCAAAACTAATCATCCAATGAGATAATGT
ATCCAGAAGGTTTAGCCTGATTGTGAAGTACTGTTTTTAGTAATGCTGGCT
ACTCTCTAGCTTTCTAATGAGTGCTCAGTTAATGCCTTAATGGAAAAAAGG
AGGACAGGATAAGGGGTGGTTTCACCACCAAGGATCTTATTTATTTGTATT
TATTTTATATGTACAAGCACTTACTTCTTTGAGAACCATGTGTATGTAGGA
GCCCATGGAGACTAGAGGAGAGCATACATCTACCTGGAACTGGAGTTACA
GGTGTTCGTGAGCTACTATGTGAGTACTAGGACCTGAACCCATGTCCTCTA
CAAGGGCAACGAATGCTCTGAACCACTGAGCCAGCATCAAGGATTTTCAT
TCTTTCATGCCCTCACAGAGTAGCCATGTAGCTCTCAAAGTAGGATATGAA
GATTCCTAAGACAAATCTCTTTTCATAATAGTATGAAATGTATTTTATTTTT
ACCACTGTCATTCTCCCATCAGTATATAAGGGAGTGTTCCAAGGGCTTTGT
GGCACTGACACTGCATCAAATCAAAAGGACAGATTAGAGAACACAAGTCT
TCCATTATCATAGATAATTAAATGTATGAATAATTCCATTATTTTTGAATA
TTATACCATATTCTTACCTTTTATGAAACATATAATTTGCATGATCACAATT
GGCTAATAATCATCATTTTAGTGAATTGATTTATAATTTTAAAATGTGGAA

TTTCGGTATCCAAAACTATGAGCCCTATAGCACATAAGCCAAAGTCTTTAG
TGCCTTGAAATGTAAGGAAATGGTGAGGGTAAACTATTTTAGGATCATTG
ATGTAACTGACTCCCTGGGTTTTGTTAGTACCAGGGGATAGGCAAACACT
GATACAGAATACTATGAGTTGAACTACACTGGAAGACAGTTAGATTTGTG
TCCCTGACATGATCCATCATTCTCACATTTCCTAAATTTACCTTCTAAGAA
AGAACCATAGTGAATTTAATGAAGTGTTATGTATACAGAATAAGTACAAC
TCATAGAATCATAAGATTCACACTGGCAACAATTAAGAGATTCTCTGGAA
TAATGCTATGACCCTACCCAGGATTCCACTGAGCCTTTGATTATGACGATG
ATGATGACGGCAATGATGATGCAAAGGCTTTTTGGAAATTTATATACCTA
AGTAATTAAATGCACTTTGAGGTATGTTAGATATGTTAGATATGTTAGGTA
TGTTAATATGTTAGATAATTCATGCATTACATTTTTCCCATGTAGAAACTG
AAATGCAATAAACTTAAACTTTTAACTAATAGCACATTTATTAAAATCTGT
CTAACCTGCTTTGGAGCCCCTACCTCTCTATGACAGGAATCAAAGTCTTCA
CCCAGCCTGGCCGTCTAGGGTGTCATACCGCTACCCCAGGTTAACTCTGTT
GACAATGTTCTTAATGGCTATTCTAAATATTAGCAATCAGGTTCAGCTTCT
TAAGACCCAAATACATGCTATACACTTTATTTCAGGCATAAATCTACAAA
ACATTGATCTCATAAGTTATGATCTATAGAACAAATCATCTCTCCGTTTGT
GTTAATATTTTGCATTAAGTGACTAACTGCCCACTGTGTTATGCAAGATTA
GGTTAAAACAGTAATATATAATGCTTTGCAATTTATTTTCTTCATAGATTT
GGACCTGCCTCACAGGAAATTTCTAGGGCTGATGTCATCTTAGAGTTTTAC
TGGCTCCAAAGGATTCACTTCCAATTAACGCTGCTAGGTCTCCCAAAGATT
GCCTTAGCTTTCTAGAGTTACTAAAGGGTCTCCTCTATCTGGCACAGTAAA
TGACCCAAGGTAAAAGCTGCTTTATTATATTTTATGACTCAGTTTTAGAAG
TCATATCCTCTTATCTCGGCATTTTTCCCATGGGCTATAAAGATCAGACCT
CTATTCAATGTGCACATGTGTAAGTCTGGACATGAAAAGGATCACTGAGC
TCATGTTGGAGGTGGCTAGTACAGTGTACTTTGGTACTCAGAAAATGTCA
GTTCTGGCTGAAGTTCAAGGAACAATCAATTCTCGCTTTTTAACTTGATCT
TTTTCTTACTTCAGATACTTTTCTTCCTCTTCTCCATTCATGTCTTCCTCCTA
CTCCCCAACCCAACATCTTTTTTAAAATTAATTCTATTTTCAGGATCCAACT
ATTTTTACTATTGAAATATATATATTATAAATTCTTTGAGAATCCCATGCA
TGCCTACAATATATTTTGATCATATTCAGCCTCCTAACCTCCCTTAACTTCT
TCCAGATCCACCTCCCACCTTCCACCCCCTCCCATCCTCAAGCCTTCTCTTT
TCTTAACCCTTACTATGCTGCTCCTGAATCCAGGAATGCAGAGCCAACCAC
AGAAGCATGCTGCTCTCTAGGTGACACCATTAAAGAAAACAAAACCTCCC
TATCTCAAGGTATCAATAACTTCTCAGCAGGAGGCATGGCCTGGTGAGCC
CCTCTCCTCCTTGCCTAACTGTTGACCAGCATAATCTTATGTCAGCATTTTG
GAGGCAGCCATAGCTGCTATGAGCTCACTAGTAGAGCAACCCTGATAGGG
TTTACCCGCCTATTGCTCCTGTAGGCCAATGACTGCCTTCACCATCTTTCCT
CCGTCTATTCCATAATGGTTCCCGAGCCTTGGGATAAGGGATTTCATTTGC
TTAGCCTATGTCTCACCATCAAAGTGTCCATAGAACATTTGTTTGTCTTGC
TTTTGTTTGTTTTAATTGAGATTTGTTGAGGAATTATAAGAAATGAAGCCA
TAGAGAAAAAAAGCTTGGATTTAGAAACACATGAGCCTAGACTTTGAGTT
GTTCAGGTTAATTTTCTATAAAATGGGATCATTTTTACTCACACACTTGGG
GCAGACTCAAATGAGTCATTCTCGTATGAAAACTGTTGAAGAAATGTTCTT

CCTATATACTGTGTAATGGTTTGTATATGACTGGCCAAGGGATTGGCACTA
TTTGGAGGTGTGGTCTTGTTGGAGTGGGTGTGTCACAGTGGGCATGGGCTT
TAAGACCCTCATCCTAGCTGCCTAGAAGCCAGTATTCTGCTAGCAACCTCC
AGATGAAGATGTAGAGCTCTCAGCTTCTTCTGTCTGGATGCTACCATGCTC
CCACCTTGATGATAATGGACTGACCTTTGAACCTGTGAGCCAGCCCCAATT
AAATGTTGTCCTTTATAAGGCTTGCTTTGGTCATGGTGTCTGTTCACAGCA
GTAAAAGCCTAACTAAAACATCCTGCTACTCAAAATTAAATTATTTTTGTG
TAGTTCCCACCAAAAGCGAAACTAATTAGATTAAGTTCATTTGCAGAAAG
TTTACTCCCTTCTGCCTAGACGTTTATTTTTGTTTGTTTTAATGTTAATTTAA
ATATAATTTCGAACTTTTCTCTCACCTTTCCTCCCTCCAGTCTATTATGTTC
CCACCCCTCCCTCTCAAATCCATTGCCTTTTTCTCCCTGAATATTATTGTTA
TATATATATATATATTATATATGAATAAATGTGTAAACACAACCTACTCGT
TCAGTATCGTGCTGCTTGAATATGTACGTTTTGAACGCTGAGCACTTGGTA
TCAGATAGCCAGTTAGGGAGTTCGTCCCCGAGAGAGGCCAGTTCCCCCAT
CTCAGCAGTCATCAGTTGCCGGTAGCTCTGAGATTTCTTTCCAGGGCCAGC
GAATCTATTGATGCTGTCATTGTTCTAGTGTTTTATAGGCAGTTATATTGA
GGCGTCATAGAGGTAGTTTCCTTCGTTTCTAGGAGACACAATCTTACAGGT
TTCTTACTCCTCTGGAGTTACGTTGTAGATGTATTCACTGGGTCTGGGCAC
CCAACAATTGCTTTATCTCCATAGTCTGACAAGTTGTAGTTTTGCGTAATA
ATGTCCGTCTGATTCAAAGAGAAGTTTATTTGATGAGATATACTTGTGGGT
ATAAGGATTGAGTTAAGAGTGATATTATGCTACACTCACAGATCAGAGAG
GCTTCCTCTGCTAGCAGATGGGAGTGGGCGCAGGGTGCAGAGCCGATGCT
GGCTGTGGAACCAGACATTATGCTGAGAGAGAAGATCAATCACAAGTCTC
CGTACAGTCCCTTCCTTGGAGATCAGGGAACCCTGTGTAGAATGCGTTGGT
GGTCTATAGGAGCCAGAAGGGCTGGAAGATATCGGAAGAACGTGGCCTA
CTGGATCAACTAAGCCAGGATCATGGACTCACAGAGACTGAGGCTGCATG
GGTCTGTACCGGGCCCTCTACATATATGTTATGGTTGTCACCCTGATGCTT
TTCTGGGACTCCTAACAGTGAAAGTGGGTGTATCTCTGACTCTTTGCCTGT
CCTTGAGATGCTTTTCCTCGTGTTGGGTTGCCTTGTCCAGCCTTCATAGCA
GGGCTTTTACCTTGTGTTTTGTATCTTGTTTTGTCCTGCTTGGCTGGTATCT
CTTGGTTTTCTGAACTAAACAGAGAGGGAGTGGATTTGGTTGATAGAGA
>LNC_008032
TGAAGCCGCACAAGGCAGGCCAGGTAGAGGAAGAGAATCCAAAGACAAG
AGGAACAGAGTCAGAGACAACACCCATTTCAATTGTTAGAGAACCCACAT
GCGGACCCAGCTGCACATATGCTACATATGTATAGGGGGCTTGTGTCCAG
CCCATGCATGCTCATTGGTTAGTGATTCAGTATCTGTGAGCCCCCATGGGC
CCAGGTTTTTTGACTCTATAGCTCTTCTTGTGGTGTCCTTGACCCCTATGGC
TTCTGCAACCCTTCCCCCTACTCTTCGACAAGACTCTCTGAACTTCACCTA
AAGTTTGACTATGAACCTCTGCATGTGTTTCCATCAGCTGCTGGATGAACC
CTCTCTGGAGACAGTTATGCTAGGCTCCTATCTGCAAGCATAGCAGGTATC
GTTAAAAGTGTCAGGGAGCAGCCCTCCCCCATGGGATGGGTCTCAAGTTG
GGCAGTCATTGGCTGAACATTCCCCTCAGTCTCTGTTACATCTTTATACCA
GTACATCTAATAGGTGGGGCGAGCTTTTGGTCAAAGGATTTGTGGGTGGG
TTGGTGCCCCCCTTCCCACTGGAAGTCCCACTTGGCTATAAAGATGGTCAC

TTCAGGCTTCATATCTTCTGCTGCTGGGAGCCTCAGCTGGGGTTACCCCCA
TAAACTCCCTGGCACCTTCCCTATCCCAGTCACCAGCTTGTCCCAGAGATG
TCAGCCCATTCACTGCTGTTCTCTCTCCCAGTCCTCCCCACCCACACACAC
TTGAACATACTTTTTGCTTTCCCAAGCCCTCTTCCACACAGTTCCTTCCTTC
CATCTACCTCTGATGCCTGTTTTATTTCCCTCTGGGCCCTCCTTGTTATCTA
GTTTTTTGGGGTCTGTAAATTATAGCATGGTTACCTTGTACTTTCTGATTAA
TATCCACTTATAAGTAAAGATACCATGGGTGTCTTTCTGGGTCTGGCTGGG
TTACCTCACTCAGGATAATAGTTTCTAGTTCAATCCATTTGCCTTCAAATTT
CATGAGTAGTATGCTATTGTGGAAATGATATTTTCTGCAGTTGAGGGACAT
CTAGGTTATTGCCATTTTCTGGCTATCACAATTAAAGTTGCTGTGAACATA
GTTGAGCAACTATCCTTGGGGTATAGTGGAGATCCTTTGGGTACACTCCTA
GGAGTGGTATAGCTGGGTCTTGAGGTAGAATTATTCCCAATTCTCTGAGA
AACTGCCAAAGTGATTTCCAACATGATTGTATAAGCTTCCACTCTCATCAG
CAATGGTGGAGTGTTCCCACTGCTCTACATCCTCTTAAATCAGCATGCGAA
ACAAATTCTCTAATTTAACGGGCATACTGTGCTTTAATAACATTAAAGGTA
AGGTGGTATCATCTTGTAGTTACAAAAAGTAGCAGGAAAGAAATGCCACC
GTGGTGGATATGTAAAAGATTCCCTACAGAGTACTTTCAAGGTCAAAATC
TAGGACAATTTGACCACCAAATTAAATATAAAGAAATTAACTAGAACCCA
TTAAAAGAAGTTCCAACATCTGTAGTAACCTTCATAAATGTTTAAGTAATT
AAGTAAATCAGGGATCTTGAATGATAGCTGTTAAGAGTACGTGCTACTCT
TGGAGAAGACCCAAGTTTGATTCCCAGAACCTACATTAAGGAGCTTACAA
CCATCTCTGTAACTCTAGGAGATTCAACATTATCTTCTGACACTACAGAGT
TATTCATATATAGACATCATATGAGTAGAAACACAAAATGTCACACATGC
ACACACACACAGAGAGAGACAGAGAGACAGAGACAGAGACAGAGAGAC
AGAGACAGAGACAGAGAATCAAGTAAAACATCATGGAGAAATGGTTTAG
GAATATAAGGCAAATCTATATATAAGCAAACTCCTGTTATAGATACGTTA
CCTTTAGGAATGGGTTGCACATAGCACATGCCTTAGAAAGTCAGTGTGAC
AAAGAAAAACTGACAGTAAAGTAGAGAAAACAGAAGAATGGACCCTAGC
CAGATGAATGAGGCTAACTCCATCAGTGGCAAAGCATGCAAGAGTAGGC
ACTCGTGCTTTCCCATGATTGCTGCTGCATGCATGCATTCCTAAATTGGCT
CTGTTTATATGGTGTCACGTGGATGTGTGTTTTCAGGGCTGACCATCAGGT
ATTGGATAGCCAATAATCGTGCTTGTCTCTGGAAGACATTTTCCTCTGCTC
TCAGCATTCCTTAGTTGCCTGTAGTTCTTTGTGTAGAGTTGAAGCCTCTTGT
GTTTTTACCAATCAACTTTGGCATGTCTACTGGTATTGTCCTTGTTCTTCTC
ATGTCTGGACAGTCATGTTAGTGAGAGTTGATGGGTACTGTTTCTGGCATT
ATTAGGAGACACAAACTACAGAAATCTACCTGACCCTCTGGACTTTATAA
TCTTTCTGCCCCTCCTTCTGCAATGTCCCTGGTCAGTAGGCATGAAAGTGC
TTGGTAGATGTATCTACCGGAAGAATTGCTTATGGTTTTCTATAGTAGTCT
GGAGCCATTGCAAAGGGAAGTTTCCTGGATGTGGAGTAAGGACTACACTT
CTCTTGGGTAATAAGAACAAATACTCAGAATGCAGTTAAAGATTATGATC
GAATAGTAAAATGGTTGGTTGCAAGTTTTCCTCCATGATCCATGAATTCAT
TAGGTCAAGGTGAAATGATAATTGACATTGATGGAAAAGAATTATGCTTT
GCACTAATATTGTTCACTGAAAAGTTCCTGCCTCTGGGAATAGAATCATTC
TCAGGTGAGTTTTTTGAAAGGTTCATTCTCTTGAGAATTAAGAAATTCCAA

AGGAATTTAAAATTTAACAAATTGTAATTGCCACTTTTATTCTCCAGATGT
AATTGTCTTGGAGGGCTACTGCGGAATATGTTCACCGTTTCTAATTCTCTC
TGAACTCTGGCTGGCCGGTTCAACTCAGTTGTTCTGGCTCAAATTCCTCCC
CTAGCTGACTGACTCAATGTGGCTTCTCTTAGCTTCTCACCAGATTCCTTTA
CTTGGCCTCAAACTAACTGTGTCAATTTGTCCTAATCTTCTGTCTCCTTATT
CTCTGACTTCAAGTGCCTCTGTTGATCTGCACTGACATGCATGACCTCACA
ACAAGCTCAACTCCACTGCACTCACTACACTGACTCCCAACTGACCTTCTC
TCTCACACACACACTGTGCTTCTTTCTTATATTGCTTCTCTTTCCTGTGATT
TTCTCTGTGGTGAGTTGGGTGTGTTGTATCTCTGACTCATTCTGTCAAATCT
TACTCTTATTCATCACTTTCTCTGCTCCTCAATTAGACATCACTTTCAAGCA
TGGCTGCTTTCTTCTATAAACTAAATTTACCTACAGTGTTTGGTGTTAAAG
GACTGTACTAAGGGTATATTTATATTCCATTCAGAGGAATTAAAGGTGTGT
GCGTGTCTACATTCCAGCCAGATAAGATGGACCTAAGTCTTTGGATGTAAT
CCTTTGCCAGAGTAGCCATGTTGCTGGATTAAAATTCCTCTACAAAAATGG
AAAAATGCATTTGAGAATCTCAATGAAATACAGATATTGAATGATGTAAT
ATGAATATAAATAGGGTTTATATGTGTATCAAATTATGAGATATTTTATAC
TAACTTGCTTAACTTTTGACTACTATTTGGGGACATTTTGTAGGATACAAT
GATATTTTTCATTTGCTTAATATAAAATGATTTGCAAATGTCACTTTTTTCC
CTGCTCCATCAATATGCATTTGTACATTTCCCCTTTGCCTAGCCAGTGTTTA
CCTGAATTCCACATCCTTAGGCTTAGTAATTCACTTCTTGTATCATACCTTG
ATATATATATATATATATATATATATATATATATATATATATATATTATCTT
CTATCATAAGATTTATGAGGAGTATTGTGCCTATACTCCACAGAGAATAC
AGAATAGCCTGAAATACATGCAGTCTTTTAAACTCAATAATTTATCAATAG
CTAGGAAAGACTGCCTGGCAGAGAGAAAACTGCTCTCTGAAAGTCACAGA
CATTGCCTGAGTGCATCTTTCCCACCTAAGTTCATCAAATAAGAGAAGCAT
CAGGTATGGCCAACAATGACCCTGAGACCTGTATTAACATCTAAATAAAC
ATTAATATATACCTATGACTAAGATCCAATAACAAAATTCCTGACTGCCCC
GTGTTGTCTGGTGAATATGACCCATCTCTCACTTTTATGAAGACTGCTTCC
TCCTGTGATAGGTCTAATATTTAGGAGACCCAAAATTATGAAAGGATTCTT
TTTGAAGGATATTAGTAAGCTATTGATTTGAAACATCTAAGTATGTTCTTC
TATAGATTTCTTCTTATGAAAGGTATAGAAATTCAGGTGGAGATAATGAT
GCTCAATTATACTTATTTAGTGTTCATAATAATTTACTTTGTTGTACAAATG
TAGCAATATTAACATTTGGAATCAAAGAGGAAAAGTTAAGCTTCTTGCCC
AGTAGCTTAACCTAAGCCTTTTCATCATTGACATGATTATGGGCTAATTAA
ATTTAAATCATTCCACAATTTTCTAACTTGTTTTGTTTGCCTAATATTTTAC
AAGTACGGGATCTATATTATTTTTGTAGGTTACCATTGAATTTCTATAGCA
AATTAATAAGGCATCAGCTGACAGTTTACTAGAAGTTGGTTATAGGAAGT
GTTCACTACAATTGCCAGATTTTGACTGGATTACTTGGTTTATTGTCTCTTA
ACTTTTTGAGTTGTTTGTGGATTCTAGAAACTAATCTCCTCTCAGATATGC
AGGATCTAGTTCTGTGGCCTATTGTTTCTCTCTGTCAGCTTTTTCCTTTGCT
GCGCGGAAGCTTTTTAATTTCATACAATCTCATTAGTCAGTTCCTACAATT
ATTTCTGTAGCTATTGGAGTCTTTTGAGAAAGCCCTTGCCTATGTATTTGTT
TTGTCCTCTAACAGCTTCGAAATTATCATTGAAAAGATAAACCACAACAA
ATGATGGCGAGGACAGTGGGTACGGGGGACATAGGAAATACGTATTATA

CACTCCTGGTGGAAATGTACACTAGAATAGCCACCATGTAAATCAGTGTA
GAAGTTCCTCAAAAACTAAAGACAAAATATAATCCATCTGTATTATGAAA
AAAAATCAGTATTGGACGTGATTTCATTGTACTTTGGTTCCAATTATTCTT
GATTTAAAAAAAATAAAACAGCTGTTTCTTCTTGTTGCTTTGAGCCTTCAC
ACTCGTGCTCTCCTCTCTCATCTTCCTTTGTGTAGTTACCGTGTAAACTGGG
TTATCTTCTTATCCTACTACCTGAGGACACCTGAGTCTGTAATCATTGTGTC
TGGTTTTTCTTTCTACCAAATAGGGAATGGTTTAATCCATTATTTCTTCAAA
CATATTATCTCTTTGTCTTTCTTTCTCTGTCTTTGTTTCTCATCCTCTGGAAT
CTTAATTACGTGTATGTTGAACCAGTTGGGACTATTCCAGATTTCTGTATC
GTTGTTCCCTTTTGAAAATCATATTTCTTCTTAAACTAAAATTGGTATCAGT
ATATTGTCAGGGTGAGGTTGCTTGATGTTTCTGCCCTATGTAGTCTTGTAT
ACATTTGTTGCAATGATTTTCTACTGTAGTTTTCAGTTATCTTACATTTTCA
TTCCTGTCTACACATTCATTAAGACAAGGTTTGGTTTTGACTCTTTATACCA
GTTGATTTACTTCCAGGTCCAGCATTCAGATTGCCTTAGAATGAATTTGTA
TTTACTATATTTTTCTTGTCATGAATTCAAGTCATTAATCATTTATGTACTT
AGCATTTTGGCTGAAACTGGTCATTTTAAGAATTATACTGCACTGTTCTGT
CATTTTGAGAATTGTTGTGGGTTTGGTATGTTCTGATAGGTAATTAGATTG
CTGAACCCCAAACTGTGTTTGTCTCCACCTGGTGCGTAGCAGTGACGTCAC
TGTCTAGACTGCTGTAGCTTAACTTCGTACCTTGAGGATCTCATCTGAGCC
TGCATGGTGGGTTTCAGCCAAGGAAGTCTATGGAGCTGACGTGCAGATTT
AGAGGCATTCCTGAGAGAAGGCTCACAGCATACTGCATTCTAAAGTGAGC
AGAAGGCAGAGCCCTATCCTTACACTCTAAAGTGGTACCTTAGCATGCAC
AGGTATTTGGGTAGAATTGGGGCTTTCACTCTAATACCAGTCTAAATAAAT
CATCCTTGCTTTAATATCTGTATATGAAAGTTTATTTAGAAATTAAAATTC
ACGGTGATATATTAGGTACTATATATGCCTGTATATTCACGTCCTCTCCTT
AAAAATGCAATTATTTTCATAATCTAACACATTTCACTAAGTCAGTCATGT
TTGTGTACTTGATAATGTTTGTTGTTACCATAAAGCAAATCTCTTTTACTGT
ATTTCATACAGGTCTACTCTTATAACAACGATATTAGTCTTCCATTGACAT
ATGAGCAGCTATGTCCCTGAATTTTATTATAATTGATTTTGAGTTGACTCA
CTCATGCTTTTGCAAAAGGCAGTGACTATCTGGAAATAATTAAAGATAAT
CTTTTCTATGTCACATAATCATTTCATACATTTCTACTTTGGGCATACATTC
CTTGGCTCGCTACTTCATTTGAAATGTTTCACACATTTACCTTTTCACTCCC
ACCACAAGATTGTCCTTGCACACCACACAGCCTTGAGTTGTCCCTGCCTTC
GCTGCACCGTCACTAGGACTGCCCAGGGTAGACGTGCAGTCGCGCCCAGC
TGTTCCCAAGCTCACACCCTTTCTTTTCTCAGCAGCTCCTACTGTCACTCAA
AGTAGCCTCTAAAATCTCCTTTTCTATAACACATGCTCCATCGCACCTGAT
GCCAGAAGATGGTTCAGGGGAGAAAACATTGGGTTGTTAAACAGGTAAA
AATGTAAACAGAAAAGCCTCTGCTTAAATTCTCTGAGAAGGAATCCTGAA
CATTAAACGTGCATCTCGCCTGAGATATTCACCTAACCGGAATATCAAGA
ATAGATATGCACCTAACCGGAATATCAAGAATAGATATTCACCTAACCGG
AATATCAAGAACACCTTACCGGGGTTGGGGATTTAGCTCAGTGGTAGAGC
GCTTGCCTAAGAAGCACAAGGCCCTGGGTTCGGTCCCCAGCTCCGAAAAA
AAGAACCAAAAAAAAAAAAAAAAAAAAAAAAGAACACCTTACCATATGGG
CTAACGATGGTTATGATCTGAGAGGAAATTCTTTATCGTGTAAAGGAAAT

ATGCTGTAATTACTGGTTGAAGATTTAATGAATACGCATTAGATTTCTCAA
ATGTAATTTGAACTATCACAATTTCTTATTTAATATGTTGATACCACATAG
TATATCCAGCTATTTTATAAAATTAAGCAATCAGTCCTTGGAAGATAGCTT
ATTTTTTAATCATGTATTTTTTTTTTAACCAAGGTGCTGTGTTTTTATTTGG
AAATCTTTTATTTAAGCTTCTGTATTTACAAACCCAGGTCCCTACATTTGG
CACCAGCCTTATTTTTTAATCATTTAAATTTATGTTTACATATTACAGCAAG
GGTTTGTGCATACAAACCCCTAGAAAGCAAACTCTATTTTTTCTCCTGTCT
GTTTATCAGTCCTTTGTCGTGGAGGGATGAGTGAATATACTAAGAAGTAA
ACAGAACAATGACAACAAAAGAAAAACTACTCACATGGAGAAAGAAAAA
AAACTAAGAGATTTCCATGTATGGTCTGTACTGAGCAAAATAAAGAAGAA
AACTTTACACGTATACTCATCTATAACCATGAACATTATATATCATTTAAC
TGGTATGTGTGCCAAGAATCACAGTTCATCTTTCAAACACCACTTTTCTGT
AGTTATTCATGTGTATGAAATCTCTGTAAACACTATCCTATGGAGGCAACC
TAACTTTGTTTTTATTCATCTTGCAGTCTTGCAGATTTCTAGATACATAAAA
ATTTTGCCTACTAGAATTTCCAATTATTCTAAATAAATGTTTGCTAATTTGA
TTGAAACAGATCATTTTAAGGGAGACATTATAGAGACATCGTGTACTGTTT
TGTCATTGAAAGTGTTGTTAACACTTTTGTTTGTTCTGACAGGTAATTTATT
CATTTCAGTACAAGCTATATGCCACTGTTTTTTTTTCATGGTCAAGCTGCTG
GTTTTATACTCCGGTACCTTGAAAGTCTAGATTATAAGTTTTACACAACAC
CTTGAAAAGAATGTTTTCTTTTTATTAAAATTTTTTATTAGATGTATTTTTT
TACTTACATTTCAAATGTTATTCCCATTCCTGGTTTCCTGTCCATAAGCCCC
CATTCCCTCTCCTCCCCCTCCCCCATATGGGTATTCTTCCTACACATCCCCC
TTACTGCCCCCCCAATATTCCCCTGTACTGGGGGTTCAGCCTTGGCAGGAC
CAAGGGCTTCCCCTTACACCGGTGCCCCAACAAGGCTATTCTCTGCTACAC
ATGCAGTTGGAGCCCTGGGTCAGTCCATGTATAGTCTTT
>LNC_008033
TGTTGTTGTTGTTGTTGTTGTTGTTGTTGTTGTTTTTGTTGTTGAAATTACTG
TTAAGGGCATGTTTAAAAGTGTGGCATGAGATTTCTAAAACTAAGAGAAA
AAATGCACGGTAACATCTTATTTGTTCTTCCTCTATGGTTTCCAAGAGTTA
ATACAGAAATGGCTAGTGTATTCTACTAATTAAGACAGTTGATCCAAGAC
AGTAATGGAGCTTAAGATTCATGGACCCACATTTTCTGGGAACCTGAGAA
AGAAGTCCTAGAAACATGTTTGCCTATGTATAGGTTTTTATAAAATTTGGG
AAAGATACACTAACAGAAGCCTAACGAGACTGCTGTGGGCTTTCCCTCTC
CTTTCCTCTCTCTCCAGATGGAGCAATCGTAGGAATGTGAACACTTCCGCC
AAGGAGGAGTTAACCTTGGACGCTGTGTCTTAGACACAGACATTCCCTGG
ATCCTGTCTCTAATATGTGTCAATGCGCCTCATATTATCTCAACAACACCT
CTGTTCTTGAACATTTAGCTTTTCTTGAATCCACGTTTATTTCATTGTTTGC
CTAGAAAAAATGGTCATCAAGAAGCCAGTTGATAGATTGCACCGTTGATA
CACTTATTTAATATATCTTCTAGTCACAGGACATATACTTCTAAAGAAAAA
GAAAGCTGACCCACTTCACACACGCAATAATTGGTTGTATTGGTTTCTGTA
TGTAAGGCTGGCTTCTCATCTCATCCTTGGCATAGTGAGCTGAATGGTGCT
AGAGGGGGAGGTATTCTCTGCTCATCTGAATGGCTGCCGTTTTTAACTGCC
ATGCCCTTGAATCTTTAAGTGTGATAGAAGATGGTCTAATAGCTAACTAG
AAACAGATGCATTCCTACACAGCTTAATTAAGCTAATAAATATTAACCAG

TGACAAATTGGTTTGTCCTAAAAGATAATAATTTTGCTATGAAAATGATTT
CTTTTATAATCTTATGGCTATAATGGTAAGGATTGAATAGGACCATGAAAT
GCTCACCAAATGACTATTTTACCATCCAGAAATCTCCAAGAACGCATACTT
TCTTTTTACAAATGGTGTGGTTAGGCCTTGCTCTGGTATTATTAATTCTGAT
TGTGAAACTCTCAGAAACCTAAACATCATTTTTAAACAAATAATATAACA
TTTTTTGCATTCATATTCAGAGACTATGGGCATTTAGAGAACAGCACAAAT
AACAACTATAAAGTTGTCTAAAAGGAAATGAAAAGGTAAATATTATTTTA
ATAATGCATATGATTTTTCTTGGTTTTACACTTCCTATAAAGTTTCCAAAAT
GCCTACTTATATATGTGGTAAATTTGGTTAGACTACTACATTGAATTTTAG
TTTTTCCTTATTGCTGGAATGTAAAAGAAACTATTTCATTGGATCAGCTTT
ATTTCCATCTGTTCCTTCTGTGCTGAGAAAATAGAAAACTAGCAAAATATA
GCAGAGGTATCCATGTCCTCTGGCAGTAACAGATTTTTCTGTCAAAGAATG
GCCTGTTTCCTGAAAGAATAAAATTTGACTAAATGTCAAAAGAAGCCTTTT
TCCTTAAAACAACTTACTTTTTGCTTGACAAGAAATGATCGTTTAATTTCC
TGTACTGTGAATGTGAATCCTCTTACCTCAATGAATTCATATTTCATTTGA
GTCTGGCTGTGTCTACATGACACCAAGACAAATGAGATGTCCCAAAGATA
GAAGCCATCTCTGCCATCACTGGACACAAACAACCCACGTGTTCCTGTGA
GTATCTATAACTGATGATCTAGAGCTCACATTTCCAGGGAGGAAACATTA
TTATTTATTATTATTATTATTATTTCCTAATAATGATGGTATTGTAGCAATA
TTGAGGGGTATCTATTCTTACACTCATCAGATATAGCCAGTGTTGGGGAAG
AAGAGATGTTCATTCCATTTTCCTTTGAAAGCATCGTATCAGACACATGTA
TCCTTGATATGGATTATAATCAGAACTTTATAATATTATTTGACTTAGTAG
TGGTAAGCTATTCAAAGACAGCTGGTAAATGAGGGTGTTACTCTGACCGA
ACCTAGACAATGGCCTTGCATTGGAGATAACTAACAGATTAGAGTTAAAA
CGGGTGTCATTCCACCCTTTGTTTCTATGGATACTATCAGATGTTCTCCTTC
AGAGCTCATGAAAGTAAAGACACTGGGAGAATCAGGGTTTAAGGTGTGCT
TCTTGGCTGGCTGCTGAATCGCAGCTGATCTGGGCACATTCCTGCCTAATT
CATCTTGTGACAGATGCCAGAAGTAGCAAGATAGCTGCTTGGCTTTAGTTT
TGCCATCTGTTGTGAAACTAATTGGTCTTGCTGCTTTAATCAATGAACCAA
AAGGATTCTAGAATTTTCTGGGTCTCAGGGGATTTGGACTCCACTCATCCA
GACTTCATATTTATTTTAACAAACAAGAAAAAAAATTTTTTTACAAAGAAT
AATTACTTGCTCTGATTTATGTAAATGTAAAATGCTACAATAATAGTGTAG
ATTTAAAATATTTTACATGTACTGAAAATTTAACTCGGGAAATGATCATAT
TTCTGTGAAGTATTCTATTGTTTCTCAATACATCCTGGCCATATTTTCCCTC
ACTCTACTCTTCCAAGTCCCTCCCCCTCTATCTCTCCTGTCCTCCAGATTTG
ATCCTCCTCCATTTTCCTTCAGAAAAGATCAGGGCTCACAGGGATATCAGT
TAAATACACCATAACAAGATGCATTAAGACTAGGTAAAACATATCAAGAC
TGGACAAGGCATCCCAGTAGGAGGAAAAGGGTCCCAATTGCAGGAGGAA
GAATCAGAAAAACCCCAACTCCCACTCTTCAGAGTCCCCCAAGAACACCA
AGCTAACAATCATATCATACATGCAGAGGACCTAGTGCAGACCCATGCAA
GTCCCTGATTGCCATTTCAATCTCCATGGGCCCCCAAAAGCCCTGTTCAGT
TGGTGTCTGTGGGCTGTGTTCTGGTACACTCAACCGGTCTGGAGTTGCTAT
CTGTGTGTTACACTTGATGCTAGGTTTACAATGTCGGTGAGGTGGAGTCCC
GAGGTTCTTTGTCTCTTGTAGCCTCTGTGTAGGAAAAGAAACGGACAAAT

AAAACTGGAACAAGTAAATGAAGAATGCCAGGTGATAGAGTCAGGGTAT
AATTGTAGATCAGTAGACTGGGTTATGATGGTGCTGAAACTGGCATTTCA
AAAAGTAGTGAAAGGAAGGGAAAGATATTGACGTGATGGGTGAGATGGA
CTGTATACAGCTACATCCTCAAAAAGAGAGAGAGAAAGAGAGGGAGAGA
GACAGAGAGACAGAGAACAGAGAGAGATTGTTTCAAAGATAATAACTAG
TGTTTCTTCAGATCAATTATGTTAAGACCTATGATTGTGACCCGTTGTGTTT
TTCAAATTTCCCTGTTGACTCTCTTATTCAACCAGGGCTTAGAACCTTGGC
TTTATATGCCTAGAAGTATTTAAAATACCTAAAAATTTCATTTATATAATT
TTTATCAATTAGGATACTAAGTCCTATAGACTTAGCTCAATGAATGCTTGT
TTGTTTATTAGTGTTTCGTTGCTTTTTTTTTCAAGATGGGGTTTTCTCTGTG
TAACCCTGGTCTGTCTTGGCATTCACTCTGTAGACCAGTCTGGCTTCGAAC
TCAGAGATCTGCCTGCCCCTGCCCTCTAAGTGCTGGGATTAAAGGAGTGA
GCCACCACTGCTGAGTTTTCCATCATTCCTAACTAGCAAACTTACTTTTGT
CTGATGACCAATAAGAGAAGAATTCATTTCTTTAGTATTTTAGCTGCATAT
GATCAACCCAAACTACTTTCTCTCACGTCCCTTGTGACTCTTCTGCTTGAC
ATCCATCAGTCTTGGTGGTATTTATACCTTTCTCTTTTCTAGTGTTAATTTT
ATCCCAATTTTAACCGCACACACAAAAATATTTGAAACCTAAACTTGCCTG
TTACCATGGAGTGTTTTAGTTTGTGCTTTGATGGGAAATCTTTTGGTAGGT
AGGCTTTCCTTTTAGATGCCTTAGAAGCCAGAGGTTGCCTGGAGAACTTGC
TTTCCAGAAGACATTCTCTTTAATGGTGCTTTCCAATATCTGTGGTATAAA
TGCTCTAACTGCAAATGACTGAAGTTGTTAATGCAACATCACTGTAGTCAC
ACATCTTTATGAATATTTCCTCCACACAAGACATAAATATCCTTGGGTGTA
AATGATAGGCAAGTGCAGTTCTGTGTGTGTGAATCCTGAGCTTGTCTATAT
TCTGTTTTGTTTTTTAATGCCATTTGGATCATTATATAATAAAATGGAAATC
GTTTTTAAAATGGATCTATTTTCTAGCTCTCAAAATGTCCTATAGTCGAAT
GGGTGGTGCTTAAAGGCATCTTATACTGACTTCAGGAGGCTAAAACATCT
GACTCACTTTGTGGATCCAAGTGAAGGAGGCAAAATTTCAAGGACCAGTG
GTTTCTGACCCCTGTGGGAGTTAGCTCATCAGGGTAGGAAGAAGCAAGTC
TGATAGGCAGAGATGACCCAGGGGACAAATGAACTGCTGACCCTGCAATC
TAAGAAACAAAGCAGAGAGGCTTCTCCATAAATGAATTAGAAAAGGTGA
TTTTTCTTCCTTGCAGTGTAAAGCACATTTAAATAGATACATTTAAGTTGA
CCCTAATCTAAAGAATGGCACTTCAGTTTAAATGCACAGAGGTTCTTTATG
AATATTTTGACATTGCTTTATTTTGATTTGCATGAGGCTCCGTCTTCCGCTA
TAAACCCAACTGCTTTTTATGAGAGGTGTTTCTTTGACTGATGTTGTAGGT
TGCTTCTAACTTAGAGAGCGGTATGGAAGCAAAACCATCTTTTTAGTTACT
CTTGGTTATCTGTTCTACTGGTTATCACTATTGGTAGAAATGACCTCAAGA
CGGAAGTGAGTGTACCTTAAACACATCAAAGTAAGCATCATTGTACTCTG
GTTAAAAAGGAAAAACAATAGAACAGCTAAATATGTGTATACACTGATCA
TGAAATAAAATACCTAACTTATGAAGGTAAAGTTTTCTCACTGAGAAGGA
ATTTCTTGAGTCTGTATTAATATGATGAACATAGAAGCTGAAAGAAATTGT
AGAAATCAGACCACATGAAGAAGCAGTTTTGGAGGCGATGATATAGTCAG
CTGTAAAGACATTAGAGCAGGAAACAGCAGTTCTACCAAGGCACAGAAG
CAGGAAGACTGGGCAGGAGGTAAATGAGTAGGCTGAATCAAAGCTAAGT
TGAAGTTCCAGGAACAGTCAAGAGTTCTGTGTTTGTTATTTCTTGATAGCA

TAGATATCTAGTGATAAATTGGTGTGGGTAAGTTGTCATCAAAGCGTTAA
ATGCCCATCATTTTACAGAGTCCATGTGAAGAACTTATACCCTTTTCTTGC
TCTCAAGTGATCAATAGTTCCTTACTTAGTTTCCATGACAGTAATTTGATA
TACCTATATGTCCCTACAGTCACTCTTCACCCTAATCACTGTGCAGAGGCA
TATTTTGTACTTCCAACATATAAGTCACTCATGAGCCTGTGACACATGGGT
GATGCCAGGGAAATGGTATGATTTACGTGTCCACCAAGAGCGCTAACATT
TTCTGTCCTCAAGCTAAAGACAAAAGTTGCATGCAAACAGAGTCTGGGGC
ATAGCACAGTCTGAAAACCCACTGATTTGCACAATACATAGTAGTTTAGG
ACAAGACAAAGCTTAATCTCACACTGTTCAACTGACTACAGTGGTTGATG
TTAATCTCAGGGCATATCTTCCACAGGTGGTAAGTGCCTCTCTGACATGTT
TGGAAGACCAGACAATTGCTGTAAAGTCTAGGTAGGACATGAGAATGGG
AAGAGACTGGATTCCAGTTACTAGAAATGTGTTTGTGTAGAAAACAGAAC
GGATTCTGAAAAATAATGGATGTTATGTTGCTGTTCCGGAAATGATCACC
ATTCTTCTTAAGTCCTTGCTCAATAAGAATAAGCTTCCAAATGAACTGGAT
TTATTGTATGAGTCTACAGGTAACACTACTGTGCACTTCATTTTGAAGTTT
TTATTGAAGTCTATCTGGGTTGGAAAAGAAGTGCCCTCTGTTCTTTAAAAC
ACTTTTTAATATGTAAGAATTTGCTGTAGTCTTTAAAATCCCAAGTAAACA
ATGTACCCTAGTAACTAGAAATTATGTATAAGTCTGTTAAGATGCTTATAT
CGGTCGTTCTTGTTGTAAAATAGACAAATTAAATTGGTCGTTCCAGCTACA
AATTTTTAGCTCAGGGACATCAAGATTTTTATGCTACCATTCTCCCCGATG
AAGTAAGTCAATCAAGCTATCGTATTCTCTCAACCTTGAGAATCCTTAGTG
TAAGAGTGACCTTGGAGACATTGTCTCCCTTTAATCTGAGTTAGATGCATT
TCTAAAACTGAGAAGCTATCAAGAGAATCCAGAGATTTGGGCCTTGGGGT
CGAAGCATATCATCCTTGTCTTATGCACTAAGTGCAAACACTACTAAGGTT
TGCATGGATACATTACAAAGCATCTCTGGTCACTGGGTGTTTCATTCCAGC
ACAAAAGAGCAATTTCATAAAATAAAAAAACCAATTAACAAAAGGTTTCT
GGGAATTCTTTAGGTATATGATGAGCTCTGCTTTCATTCTCCTTTGAAGTTT
TGAGGACTTCATATTGGGTTAAGAATTGTTCAGTAATGTCGTACGTAGTGA
AGGGTAACTTGATTTGAACAGTAATGCAGAAGGAAAAGGTCAAATGTGTG
ACCTTTATCCGGAATGTTTTCTTTCTACTACCTCTTATTTTGAACATTTTAG
GTATGAACATTTCTAAAACATGTCGTTAAATTTGCTTGTGTTTTACTTTTTA
TTACTTCTGATATTAATGTGGCTACAATCTTTTATAAATCACTTTTTGATCT
TCATTTTAAACTGCTTCCTTGAAGATGTGAAAATCCACATCCATTTGTATG
ATGACCTCTGAATGAGTTGGATTTTTACGAATTATATTACCATCAATCATC
TTTATGAATTTTGACATGTTTTACAAATAGAGAGATATTGAGTAGAAATAA
ATCTCTGATTAATTTTGATTTAGAAAATACTAATATCTTTTCTAACTTAGAC
CTATTTTTTTTCTATGCTATAAGCATGAAAGGTGCCAATAACCACCTGGTG
TTGTTTGTTTCTCTTCTTGTCGGATGCCCATTTGAGTGAAAATCAGTGCATC
TCGATGGCGATTTCTAATCACAGTTCTTGCATATGCTATTTGTTTGACCTTT
TAACATCATTATAAGTACTTGAATCAGCTACCTAGAACGTAAGAACAAAA
AAAATCATCTGAAAAAGTGACCTAGCTGTCCTAACATGCTCTAGCATTGTT
AAGATTTCTGGAAAGTTAAACTGAAAAGACAGGGATTTCAAAACAAAAG
AAAAAACATGGGAAGATAGAGGAGAAATATTTGAACAAAGGCATACATC
AGCTGTGACCTTTAATCTTGAATATTAACTTGATAAGGTTGAAGGAAGAT

GTCAGAGAGGTTACTATATACACCTGTGGGTATTTTCAAGACAGTCTCAG
AGAATTAATTAAGGAGATGTTGCATGAAATAATAAAAATGACACCATCCC
ACGCTAGCGCCGGCTACTCTCTGACCCCCGCACCCGCAGTCCCTTCACGTC
CATGGCTAGCCCCATTTTGTGACTGCCTATACAGCAGTTCTCTTGTGCAGA
TTCTGCTCACATCGCCAGCCATCTGCCCTCTGTGGTTAGCAGTCACAAATC
GCTGTAACCCGGTAAAATCAAGACTCTAGAGGTTTATAATTTATCAATCA
GATTTATATCAGTAAATTATCACCCCACAAAACGTACACACAATGAACTC
AGAGACAATTGATATTGATATAAACTGCCCACCTAGATAAAACAAATTGT
CCTGTAATAATCCATCCCTTATAAGCCATTCATAGCTTCCTGTGGCTAACG
TCACGTGGAATCTGGATTGTGTTTTTCTTCCTCCATCTTCCTTCCTTCCTCT
ATTTCTCCGTGTCTCTGTCCCATCTCTAAAATCCCCAGCCCGCCTTCCTTTT
CCGCTGCCCAATCACAAGCCTTATCTTGTCACCTACATATAGACAACAACC
CACAAGGAGGAAAGTTCTTCACTGACTGCTAGTCATCCCGTCCCACAGTC
GAGGGCCTTAAGGTAAACAGAGAAAAGAGAAAGCTAGCCAGTACTTTCC
ATTAATTTCTTTCTCCTGTTTCCTGCTTATCCTGAGGAAGCTCCTTTGTTCC
TCCCTTCCATGATAAACCAAAACCTCTGTACCTGTAAGCCAAAACAAATC
CTGCCTGTGCTTTGGTTTGAGAAGTTGTTCATCTTAGGAACATTGTCACCA
ATCAAAAGTCTGACACAGAGAAAAAGTTGGCTAATCTCTGTTCAGGGATA
GAAACCACCTCTTAGGGACACGTCCTTTCTCCAAAGAGATTAAAAGTAAA
TGTGAAACTGAAGCGAAATTCAACCATAGCATCATCACAACCATCAGGAA
ACTTATTAACAGTCGCTGCTGGGCTGCACCACTAGAATTTCTGATTCAATA
TGTCAGAGTTAGCTTAGAAATTTTATTCCTTAAAAGATCTTTGGTGAACTA
AAAGCTGCTGAACCAAGACACATTTTGAGAAGCAAGAATTATGCTTTTCT
CTTTTATTTTCTTTTTTTTATTAATGCCCCTTGGCCATTGCTGTGGGCCCAC
TATTCAGGAAGCATACATAAATACATTGTATCACAGGGTCAGAATGTATT
AAATAATAATATATTCACAAGGTTTGATATTTTCAGCAGCAGCAATTTGCA
AGATAATACCACCTACTACAGTTATCTGCCCAAGGTAAATCTTTTATTCCA
ATCTTAATTATTTAATATTAGCTTCCACCTGGATAACACCCAGTAAATACA
TCTGTATATCTCTATGCACATATATGATCTGTAGAAGGAATACACATAAAA
GAGGTTGCAGCAGTGTTTAAAGGTTTGAGAAGTTGGGTGAGCGTCTGTGT
CGATGAGATAAAGAACCAGGCCAGTGCTGGGAGCTACTGCTGTAGCTTCT
TGAGTCAGGGTTTGAGAGCAAAGCTATAGACTCCAGCTGCAAGTTAAAAC
ACCAAAGGAGAAGACCCGAGCAGAAAAGGCAGGCTTGAGTCCAAGTCCA
ACTGCATGAACAGGTAGACAGGC
>LNC_008035
CAATATGTTGCTTTTTTATGGAAATAATAGATAACAACCACAGAGTGTTTT
TAATGTACTTACTCCTTACTAGCCACTTTGATATGTCTTATAAAGACCACA
AAGTAAGCACAGCCACTGTCTTATTTTGTAAATGAAGAAATGGAGAAAAA
ATCAGATATCGCAGAACTGTAAAACAATATGTCTGTGTGTGAATAAGTAT
CCTTGTCTCTGAGATTAGTGATTGCCACATCTATTTCTATATCCTTCCTTCA
TTTGCTCCAGTGTCAAAGGCAGATATCAACTAACATGCTCCTTATGGATTG
ATCTAGTAATTTTCTATAAAGGTATACCTCATTCTTCATTTAATATTATATA
ATATTGCTATCAAGCAATATTAAAATTATAGAATTTACTTTTTTCAAATGA
CGTTGGCTAGTTACATCGCAACTATCCTTTTAAAATTCCTCAGGATTGCTG

AATATATTACAACCTACATTCATAAGGAGAAGCTCACACTTGTTCCACACT
TAAGAAAAAAAGCTTAGCAATAATCATCGGCCAGTTTCTGTTACAGACGG
CTGGTTCTGAATGCTTGTAGGATCTGAGGTTAGATATTAACTACACAAGCA
TCCTAACATCATGTCTCTCATTATATTATCGTATATTCATTGTCATTTGGGT
CCTATTACCAAAACAAACAAAGGATATAAAGTTTTACAGAGTGAGTGCAT
ATTCAGAACACAATTATTGGGGTTGTGCTTTTGGCTTAACAGATGTCATAA
AAAATCCTTGTTTGTGTAGCTTCATTGCTTCACAACACTTAACCTTTAGTA
GGTAAGTTTTCAAATTGCTCTGGGCAATCCTGGAAAGATACGATGTACAT
ATCCCTACATTCTGAAATGAGGAGCACAAAGTAGTTAGTTTTAGGACATG
ACGTCTCTTGCTGAAGTTATGCATCCAACTTATTACAATACATAATGCAAA
TCCAACATTATCAGGAGCACTAGGCACTCTTGCTGGATGTTTTTGCTTAGG
AAGTAAAAGTAATGTGTTCTGACTCTATTTAATTTTCCA
>LNC_008039
CACACACACACACACACACACACACACACACACACACACACAAACATCCTGC
CTAGCCTCTCCACTCTCTGGAGCCTCAAACCAGTTCCTGAATGCTGCCTGA
TTGGTGGCTCAGTATCTGAGAGATCGAAGGGGTCTAAGTTATTTAAGCCA
CAGGGGTCCCTGGCTTCAGTCCAGGATATAAGTCTCTACATCTCTCTCAGT
CAGTTGCTTGTTGGGCCTCTCAGAGGACAGGCATGCTCAGATCCTGTCTGT
AAGCACACCATAGATCAGTAATAATGTCAGGCCTTAGAGTCTCCCCTTGA
AAGAGATCCCAATTCGGGGCAGTCATTGGAACTCCTTTCCCTCAGACTGTT
CTCCATTCTTGTCCCTGCAGTCCCTTTAGATAGGAGCAATTCTATGTTAGA
GTTTTTGTCTGTGGGATGGCAACTCTATCCCTCCACTTGATGTCCTGTCTTT
CTACTAGAGGTGAATTTTAAAATCTCACTCTCCCCCCTGTTGGGTATTTCA
TCTAAGGTTCCTCCATTTGAGTCCTGAGACATTCTCACTTCCCAGTTCTCTG
GAACTTTCTAAGGGTCCCCCCCACCTCCCACCCCTGAAGTTCCCTATTTCC
ACTCATTCTGCTGGTTCTCAGGGATTCTCTCCAGATCCCCCCAACCATTAC
TGATCTTGTTCCCCTTTTTCCCCTCCTGCTTCTCTCATCTACCAGATCTCTCC
CTCCATTTGACTCCTGTGATTGCTTTCTTCTCCCTCCCAAGTGGAATTGAGG
CATCTTCACTTGGACCCTTCTGCTTGTTAATCTTCTTGGATTCTGTGGATTG
TATCCTAGGTACTCTGTCCTTTTTCCACTAATATCCACTTATTAGTGAGTAC
TTTTGGGTCTGAGTTCCATCTTGAAGACCCAGCTATACCAACAAATAAAGT
TTAAGTTTCAATTGTTTTCTTTGTATCAAGATGTAGACAGTGAGCTGTATA
AAAGTACCAGATAGGATCATCCTAACATTAATCTTAACAAAAGAATTAAG
AGTTAAGGAGAATTCATGACCATGGACTGAGTAATATATTTGTATAGTGT
ATGATTTGAATTGGTTTAAAAGTCTCAGGGAAAAATGAGACTCGTAGCAT
ATTAAAATTATAAAGATGTTATTATTTAGAATAAAAGAAATCATCCCATA
CCTCTATCTTATTGCCCAAAATAAAGATATTAGCATTCAGAAATTTAAAAT
AAAAAATAACTTTAATTAGCAGGGATACTAAAATAAATTGCCATGAAATA
AAGACAATACTATGTTATCTGAAGTTAGTATTCTAAATGTGAAACAATTTC
CTTTTGATAAATTAATTCAGTGTTTATTTACCTGTAGCAGTGTATCTCCTGG
AGTTGAATGATTAAAATGTATGCAATTAATTATTGCTTTTATATTCCCAAC
AATTTCTTCAGTCACTATACTAGAACAAAAACATGTCATCTTGCATTTTCA
AGGTCTCTATTTTATGAACCAATGAAAGTGACCTGGCTCCTGGATGGACAT
CTGGAATGAGGAACCAATAACTCAACCAAAACATAGGAAGTTTATGACAT

TTAGTTTGAGTTTTCTGATACTTGTGGCACAAGTCATGCTGGTTTATTAAT
ATAGAATACACTAAATTCCAATCTTCTGTTGGGAGAAGCAAGCACTGTTG
ATGATCTGGCAAGAACAGAAGTTTCGAGAATACATCAAAACGTTCTTTGT
CACTATTTCAGCTCAGCCAAATTTTGAGTATTTCTCCATTACCTCACTCAGT
TCTCTTTCTGTTTTAAACCTGAGAAACTTTTCATCATCTTGAGATAAAAAC
TAGGAACACAAATCCGTCTTAACCTTATCTAAAATGGAACATATTCAAGA
AAGCAAATCAGTTTCAAAAATATACCAAAAATTCATGCCAAATCCAACTT
AAGATATTTTTGAATTATATACTGTTTACAAGTATATAGGCCATCAATCCG
GGAATGTATGTAATTATAAACTGACTCATCAAAATGTTCCAGCTCCACTTA
TTTAGGACTGGAATTCGTTTACTAAATATTCACAAACTGTTATTCGGAGCT
TATATTTTGGCAGGCACCAAGCTGGTGAGTACATATGGATTAATAAAAGC
AAAATGAAGACTGCTCTACAGCTGAGGTCTTCAATTTGTGCTGCATCTTTT
GCGACCTTATTTCAAAAACTATCAAGAAAAGTCAATAACAGATGAAAAAA
ACTAATGTATTGGGAAGAAAATGTACCAGGAGCCCTTGGTCCTCAGCATC
ATGCACGGAGTTTGAGTAGAGCCAGTCTTTGTACATGGACATCTTCTTAAG
AGCATATGTGCCAGAACCACGCTAGCAATGACACAAGGGCTCAGGAGTCT
TGACTACATCTAAAGAATTTATTATTAGTGTACAATAGTTAAGAGTACAG
AAGCCTAGAGTAAGAAGGTTTGGATCCCACTAATAAAAGTATCCTGTGCT
AGATGTCAATCTAAGAAAAAAATACGATGTGCAATTTATACCTCAGGCCCT
TATTTTGCAAAATTCATATTGTAGCATATTTTTTCAGAATGGTATGTGGAT
GATTTGTTCCAATGCCTATAAAATGTTTAGAATCAATATGAGCTTAATAAA
TAATTGTTACTATAAAGCAACTATATTATATATTAATATTTAACTTAGTTA
ACATTTACTAAAAATAATAGTATTTAATCCAGTCCATTTTCTAAAATACAA
ATGCGAAGCCAAAGGACACCTTTTATCTCTTAGGTCTCAAGCAATGTTGAT
AGAGCAAGGATTTCTGATTAAGTAACTCCAAGAGAACTGCAACAAACGCT
TTCAGAAAAAAAAGTGTACAGAGCTGTTGAGATTCTACAATTCATGGGTT
GTGACAAGAGTTTCCAATTAGTAAACTCTAGAGTTTGATGTAGAAAGTAA
TGAGAAATGAGCTTGCAGAATGAGAAGTCAGCCATGCCACTCACTGTAAC
ATGCTGAATTCTAATGCATTAGCCATCGTAAAGAACAAACCTGTATGAAA
TGCTTCCTATATATTAGGCAGTTTTCAAACCACTTTGAATACATGTTTTTCA
TTTCTCAAATAACTCTCTAAGATAGACATTTTCAAGGAAGGATACCACAGT
CCAGAGATGTCACATCACAGCAGAAGCCAGAATCTATCTCTTGTCAATAC
TCTAAAACAAATTTAGGGGAGCTTACATACTGTGTTGTAAATGCTCTTAGA
ACAGGAATAGTGGGGTTATATTTATTTAGTAATTTTTCAGGGAGTGAACAT
GTCAGTAACATTTTTGGAACATAGTTAGGCAGTGAAGATATCTAGCTAAA
GGAGAAAGATACAGATACTGCAACTTCAGAGGCTCCTGGATTTTGAGGGG
AAAGGATTTAGGCCTTCAAACACAGTAACAATGGTGAAATAGAAGACGG
GTGATCCAGGAACTAAATAAGTGACAAAACCACCAATATTTATGATCACA
TGTGAAAAAGAAGAGTGTTGCTTTATTTTCTCATTTCTGATGACTGGTACT
ATTTGTTAAAATAGGAACTGGAATAAAAAGTTACTAGAAGTAGAAGGTAA
AGCCGATATTTGCAATATATTTGACATACCTACCTGGAAAGCATCTCTCTA
ACGTGGTCCTAGAGTCAGAACTTACTGTATAGAAACTTACAGAATATAGC
ACTGTTTAATAAAATGATTAAAGACAAAAATAACATAGAGACATAGCACT
ATCTAAGTTTGAGGACAGGCCCGAGATTCCACCAAATAAACCAAGTCTGT

AATCAGAGCTGTATTACTGTGTTGAGCTCGCCTCTGACAGATGCTGCTGAG
AGTTTCAGGAAAATGAAGCCTGGAAGGTTCCCATTGCTTTTGGCAAACAT
GGAGGTCACCTTCATTCTGGATCACTTCAGTGTAGACATTACAAGTACCCA
GATGTCCCCCAGAATAATTGCTAGAATTAGGAAATATTCAAAGAGTGGAT
ATACTCTACTCCTTCAAGAAACATGCTGGGTGGCAAAAATGAAATGAACC
CTAAGGGATGGGTGCTTTGTTGAGCTGTGTTTGGGCTCTGTGGTAGAATAG
ACTGAAACTTTAAATTCTTTATTTGTGCCAGATAAGCATGTTATTTTACCT
ACACAATATTTTCTAGCAAGTCAAAAACCTTTGACTGATTTATTGAATAAA
ATAATTACTTGTTTTCTGCATCATGATAAAGCTGGGGCTATCGTTTAAGCA
ACCTTAAGTATGAGATCACATTTTCCAAGAATATAGGGATGGAGGACTAA
TGGAGTTTATGTAAAACATTCCATTTAGTGCGGTACAGGAATAAATAAAT
ATACTGCTTTTAGAGCACCTCAGAAGGGAATGGTACTGAGTACCAATCTT
AGAAACTGAAGAGTTCATTGAGTGCCATTCCCAAACTCACTGTCCTCATTT
GCTTGGAGTTGATGTTTTTCTGCCTCTCTGTTTCCTCCTACACCTTTGAGTT
GGATTAGTAATCTTTTTACCATGTGACAAAATACATGAGATAAACACTTAT
TTTGGATCATACTTTTAGATGTTTCAGGCTGTCGTTGAGTGGTTCTATCACT
GGAGGCTCCATCACTGTAGACTTGGCTATATCCTCATGGGGTGTGTGTGTG
TGTGTGTGTGTGTGTGTGTGTGTGTATGAAGCACTTACTATCTGACACCAT
TAAAGCAAAGGGAGATAAATGTGAATGTCTGTATATGAGACCATTCAAAA
GGATTCTCCTAATATTTTCTGCAAATAGGCTTCACCGTCTGCTGGCCAATT
CACCCATGTACTTATCAGTGGGGTAGGAAGTTAGCACCATCAAAATCTTA
TCATTGAAAGCCTTCAACAGAAACACTGGCTGAGAACACAGACCTCACAC
ACAACCATGTCTGCAGAATGAGTTAGAACCAAAACAAAAGGAGATTGAT
GATTAGGAAGAAATCTTGTAATATGAGAGCAAATCCTTTGTAAAATTGTTT
AATGTGGTTGAAGTGGAAAAGTGATACTTAGAGAGTGAAGATGGAGACTT
GGAGATTGTCTCCAACTTTTGAGTAAGAGTTCAGGAGACACAGTATGCAA
AACAGAAAGAATAGAAGCAAAGGAAGGTGTCTGAGTTACTTCATTGTGCC
TGTGAAGGGTCACCATGAACAAGGCAAGTTATCAATTGAGCTTAGTTGCG
GGCTTCCTTACAGTTTCAGTGAAACCATGACCATTATGATTGGAGGCATGA
CAACAGGTAGGCAGGCATGGCACTAAGATGGGACAAGATCCACCAGCAT
GAAGCAGAGAAATAGAGAGCTATTATAATGGTATGAACTCTTTTCAATGG
TCAAAGCCCACTCCTAGTGACTCACCTCCTCCTAAGGACATACCTCTTAGT
ACTCCCACACATCTAAGACATTGTGGGCCAAACATTTAAGTATATGAGCTT
ATTAGGAAGCACTATTGTTTGAGCAATATATAAACAAAAGATAGTCTTTTT
TCATATTTGTTTCTCCAGGAATGGGGGGCACTTGAGCATGATGAAGAAAG
AAGATTTTATTGGCATATGTATTGAGAGGAAACAATGTGGAGCAAGGCCG
ATTGGCTGATGTCAGGACTGGAAATGAAGTGAGAGATGGGACACAGTATG
GAATTAGATAGAACTAGATTCTGTATTATGCTAAATGAGAGATAATAGAC
AATGCATCATATCTCCTATAATCTTGGTGCTGGGGCCTCACTGGTCAACAA
GCTTCAGGTTGAGTAACCAACTCTCTCTGAAAATGGACAAAACAAAACAA
GGCAATTTCCACCAAGAAAACTGGTACAGGGAGATGAAAAAAATAACAA
ACATCAACATATATATGTATATATATATGTGTGTGTGTATGTGCGTATATA
TGTGTGTCTATGTACATAAAATCATATGTATCCACACACAAAATGTGATGA
ACATAAAAAATTAAAAATCCATAAATGGCAGATAGCATTAAGGAGGGAG

TAAGAGGAAGCATGGAGTCAAACTCAGGCTTGTGATTCTGGAAAAGTTGC
AGAGTAGATGAATATATAACTACAGAGATTAGAATTTGATCCATATGAAA
ATTGATTCAGGCAGTTCTAGAAACCAAAAATTATCACATTCAAGAAGTGT
ATAGAAAAGCAGCATGTTGGCTGGAAAATGACTCAGCAGTTAAGAGCAC
ATTCTACTTTTGCAGAGGACTCTAGTTTGTTCCCAGGTTCCATGTTGAGCA
GCCCACAATCCTCTGTCATTCTAGCCCCAGGGGACTCTGATGCCTCTGGCT
TCTGCAGGAACCTGCAGTTAAGAGCACATATCTAACCACAGGAGTATACT
TGTATGTAAAATTAAGTAATAAAATTAAATAAATAAAAATGTAAAGAAAC
GTACCAATTTCACAGCATTGTGTGAATAAGTAATAAGATAATATTTATGA
GATTGTTATAAATAATATTTTACTTTAAATCATTTAGATTAGGCACTAGAA
TTGGGGATTGTTCATTAAATTAAGTTTAAAATGTCTAATTTGTATTTTAAG
AAGATTTTCTTATTTTACTTTTCTCTATGTGATTCCACTTAAAGAACAGAGT
GTAGAGAAAGTGGTGTCTGGTAACATACTGTGTGTGAGGGCTTGCCAGAT
GGCAAGTCTGATGTTCAAGCATAGTTGGCCATAACTCTGCAGCTCACAATT
GCATTGGATATGCAGTTACAAGGGAGGAGATTTGTTTTGTACAGAAATAG
CAACAGTCATATAGGGAGCTCTAGTCCCCTTAGAAGAACAATAAGATTAT
AACCATGGTGGACATTTGAGGAATGTTTATATTAACAGACATAAAATAAT
AGGGTTATATTAGCATGGTAGACTGTTGGTATATTCCTTTCCAAAGGCTAA
TAGATCAGAAAGCCAATAAAAGTCGGCCCTCAGTGTTAGTTCCAAGAGGT
TCAATCCCAATTCAGTCAAGAAATTAAAATTCTTCTCTACTAGACTTTCTT
AGTATTTTTAATAAGGTCAAAGTAGTGTAGTATACTACTACTACTACTACT
ACTACTACTACTACTACTACTACTGATAATAATAATGATAATGATAATGAT
GATGATGATAATTATAATAATAACCCAGTCTTCTTAAGTTATTCTTTTAAT
GAACATATATGCCATAATATATGTATACACACACACATATATATATCACTA
TACATAGCTCTAAAAATATTTCTATTTTACTAAAGAAAATAAATCAAATAA
CAAGTATTCTGTCTTCATGCTGCCTTTGTCTGTCTTCTTTCTTTTAACCAAA
GGTTATAGAAGTTTGACAGTTTTCTACTTTTTCATTCTATGAGGGGCTATTT
TAAGGTGTTTCTCTTTGACCATGGCTGTGATGTTCTAGGCTATATTTAGAG
TAAACTCTGAAATGCAAATGTTCTCTAAGTGGCACCAACATTGCTCTTTGA
GATGTTTGTAATGAATCACTTTTCTGTAACATGTAAAATAATCCAAAGGTC
TTTGTAGAAAGCTCTGTAATATTCATCGTTTTGAAAGCTCCGTTGACTATG
TCCCAACTGAATTTATAGTATGCCATGTGCTTCAGTGATGGGAAATTTTAA
CTAATAACTTTTTTAGGCAATTTCTGTAGTTTCAGTTCTCAGAAACAGTAT
ATGAAATACTTGAACATTATCAGCTTTAAAGAGTTGGTTCAATTATCTTGT
CTATACCCAGCATGTAAAAATCATATCTAAATCCTCCTGAGGCTTACTCCA
CCACAGAATCCCTATTTACTTTAATTTGGTAGAACAATGGATTTACCAATG
AAGGTGTGAAATATGCCACTTGAATTGGAACAGGAGCACATCAACGGCAT
GGCAGAATCTGATTCATTACTAATTCACGAATTTCAGGAGTGATTTCTGAA
AACATGTATAATATTTTAAAAAACATTTGTTGAGTGAATGAAAGAACGCG
TCTCCTTTCTCTATGGCTCTAATTTTAATTGAATCATAGTGTATTATTCAAA
GATCTTCAATGAAATAGAAACAATTAGCAATTATGACTGGAGTCATAAGG
GATTTAGTGAATTGCATGAGGAGGCCGACTAGTTCCAAACTGCACAGTGG
ACGTCGGTGATATGGAGCGTGGAATGGCTGGTGGTGTCTTCCAACCCAAA
GGCTAACAGACTAGAAACAACAATAAAAGCTGGTTCTACACCTTAGTGAC

AAGAATGCCAGTACAAAAACAGTCAGAAAATTATAATTGTCCTTTACTAA
GGGCAGACCTAATTCTGCTAAAGACCTAAGCCGATTGGGCAAGGCCCACT
CATATCAGAAAGGGGCCTCTACCTCACTGGGTACGCAGATTTAAATGTAA
GGCCATCCAAAGCCACTTTGCAAGATACAACTATAATATGTTTGATTAAGT
ACCTTGGTACCCTGTGGTCCAGTCAGCTTGACACATAAAATTATCCAACAC
ACACACACACACACACACACACACACACACAATTAACAGATATTGTACAGAT
AGCTATTTTAGCCAGAGAGTATCTTTTGAAAAAATATTGAAAACAGTTAA
AACAATTTTTTTTGCATATATTAGATCTTGCTCTAATACACTCTGGTAGACT
TTTTGGCAGTGTGAGCGCAGCTTAGACCATCCATCCATGAAGCAACTAATT
TTACACAAGGTATTTGTTTTCCCCAAAATTAGGCATAAGTGTTGCCCAGTT
TGTTTTAACATGCATTTTCATTTATAATTAATCCAAATGAACACTTTCCCAC
TATGAATAGGTACCTGGTTGTGTCTAATTTAAACACTATGTTAAATACCTT
AGCAATGCACTATGTATGCTTGCTATGATTCAAAATAGTATGAGGCCTTTC
CAAAGCCAGGCTGCTAAGACAATTATCCATCAGCAATAAACAGTTTGGCT
GCCAATGGCAGAGGCAGCTCAGGAGTGTTCTTAGAATAGAGGAAATTTTT
TCATTTGGAGAATTGCCTTTGTTCTTTTTCCGAAAAGGAAGCTTTGTTCTA
GGTAATAATCATTAAGGCTTTTTAGAGAACTGCTGTATTTGGAAATGTAGA
GGTAGCATTTTAACTAGTACATGGTAAGAAAATGGTATCCATTTA
>LNC_008050
CGGATTCAGATAACATCTATTCCCAGGAGGAAATAGAAAAGGGACACTGG
GAGAGTAAATGGACAAAGGTCAAACCCACCAAAATAGTCATGTTAGACTT
ATGCTTGATGAGAATACAGAAGGTAATTTGAGTTATTAGTAAAGCTCTAT
ATTCTGGTTTCCCCAGAAGAGTTTGACTTTCATGAAGCTGAGAACCACCCC
ATAAGCACTTACCGACTGTTTGCTCCTCCTCCGGCCACAAACCGTTTCTGA
GGGTATTTGTTTTTAAGTTGTTTTAAGGTCATATTTTTATGGGCCACAACCC
AGACGTCACCACCTCTTCCACCTTCTCCACCTAAACGAGGGTAGCCCATTC
CACCACTTCCTCCCTTGGTGAAGATTCTTAGATTATCAATGAAATTTCCAT
ACTTTCTCAACAACGCGCAACCACAGCGCACCATGAGCTCCAAGGGGCAG
GACTCCCGCCCGCAGAATCCGGAC
>LNC_008129
GTGAAGAGGCATTGTCCATCAAGCTTTTGAGCTCTGCCTCAGCTGCTTTGC
CATTATAAAGGCGAAAGTGATTGCAGGCCTTAAGGTTGAGGGCGATGGTG
CTATCAGGAATTTGCTGAAGGTAAACAGCCAGGACTTCTTGAGACACATC
ATAGTAATCCAATTTGTAGTAGCAGAGGGCCACATACACATTGAGGGCCA
GGTATTCCCTGTTATCCAGCAGTATCCGCTTGTATATATCAATAGCTTCTT
GGTAATGAGATCGCATGTAGTGGATTGAGGCCAAGCTAAGCTGGTCTTCT
TTGATATCCTGAAGATTCTGATGAAAGTTCATTAATTTCTTCTCATCATTA
AACTTGTGAGCCAGATGGAATAGTAGGCGGTTTTGGAGTCGGCTTTTTGG
AGCTTTAAACCCAGCAGCTTCTGCTTGTTTATACATTCCAAGGAAAAAGTA
GGTGCAGGCCAAGTTTACCCACACTTCCGGGTTGCAATTCTCCTCCTTCGC
CGCGTTTTCGTATTCCTCCAAAGCTCTCTTGTAGTCACCCAGGTGAAAAGC
ACAGTAGCCAATCCATAAGTTAGTGTCCTCTTCTTGCTCCCCAACATGGCG
TTTGAACTCCAATAGGGTAATAGCGCCAGTGAAATCTCTTTGCGAAAGTA
GATCCTCTAGTTTTGGAATCTTCC

>LNC_008149
CTCCACGACATGCTTGGATGAAGAACATTTTTGGTTTGTTCTGTAGACTTG
GACAGTTAGCATTGTCAAAAAGTCGAAAAACCTCTTGGAGCTGAAGCAGT
TTGCCATCCACCCCGTAGATGCCACCTTCCACACCATGTGAGAGGAGTGCT
ACTATGCAGGAGTCTGTGACCCGGTGTGCAGGTAACTGTGCAAAATTCTG
AAGTTTCTCTTGCATTTCCTAGAAGACAGAGACACCTGCTAGTACTGTCC
>LNC_008231
GTATCGATAATAGTGGGCTTTAGAGTGGTCAAAACTGCTGAAGGGGAGGC
CAAGGTTGCTCAGGGCTGGCTCTTCACCACTGGGCTGGGAGACTCTGTCC
AAGCCTCGGGACTGATAGAATTCCCGAATCCGTTTCTCTTCACTGTCTTGC
AAGCCTGGTACTAGCTTATACACTATGTCCTGCATGACCCGATCCAGTTTG
AGATTGAGCAACGGCTGCGTCTCGTGGATCTTGATGTTACACATGGGGCA
GTACTTGCTGGTCTGCAGGTACTTCACGATACAACTTTTGCAGAAGGTGTG
GAGGCACTCTGTGATAGTGGTGGCGTCCACGAAGTAGCCCGCGCATAGGC
AGCAAACGATGTGTTCATTCAGGTCTTTGATCTTCACCCGGACCTCCTCCT
CGTTCCGTAGTGGGTCCATCTTGTACACTGACTGGAGCTGGTTCCGAAGCC
TCATCGCGAT
>LNC_008251
CGGATTTCCATTATTTGTCCAAATGGAGAAAAAGTCTGACGCATTAGTTGT
TCTGTCAGTCCTGACGTCACTCCTCCACAGTACACGGTACAGTTGCCGGGA
CTAGACTGACTCACAACCTCATCGTATGACAGCTGTTTGGTGTTTGACTCA
TATGTACTCTTTGGAGCTGGAGGCTTTCGGGTTGCCCAGTTAGTTCTGATT
TGTCTTCCACCAAGCCACTGGCCACCCATCTGCTGAATGGCATTTTCTGCA
TCCCATTTGTTGAAAAAGGAGACAAAGCCATATCCCTTAGACTTCCCGGT
AGCCATGTCTTTTACCACACGGGCATCTGAAATTCTTCCAAATGGTGCAAA
AGCTGCTTTGATGTCTTCAGTTGTGATTTCTGGACTGAGGTCACCAACAAA
CACATGGAAATGATCTTGTGAACGCTGTGTGCTGACAACGGTACTACTGC
TTGTATCTTTCTTTTGACTGCTAGGGGTTGTTGCCCAATTCACTTTCACTTC
CTTACCCATTATCTTCCGCCCATTCATAGCAGCCAGGGCTGCAGCTGCGTG
GCGATGCTCATGGAACTCCACGAAACAGTACGGGTC
>LNC_008683
ACCACCTCCACATGGCTCTGCTTGGCGTTCTCGAGCAGATGTCTGCAGGAT
TTCAGCGAGCGCCCGCAACCTCAGGCTCAGGTGATTCAGAGAGTGGGAGT
CCTGGGTAGCAATGCACAGAACCATCTTGGCACTGGGGTGGCTCTTGACC
ACCTTGGCCAGTTCCACTTCATTGTCGAAGCTTAGCAGTCTCACCCCGTGC
TTGGCAGCATACTTGATCTGGGCAACTTGCTTACAGGGGTTGGCACAG
>LNC_008689
GGGGTGGGTGGGAAGGTAGAAACAGAACCAATCTAAAAATGGCTGATAT
TACCCAAGCCTGAAAAGGGGCCCCAGAAGACACAGTCGCCCCTGCTGAAA
AGCTTAACAAGGCCATCCTAGCAGAGCTCCTAAGCAGGCACTTGCAGTTC
AGGATTTAGTCTTCCGGTTGTTTTCTTTTCACCTGGACCCTACGTGGTTCAA
AACTCAGACCTAGGGCAGGACGGACAAGGGAGCTCTGGGAAGAAGAGTT
CTCCCAGTACCACTGCCCTGTGCGCTGGTCCCTATCTAGTCCCTGGGGACG
TTATATTTAATATATTTTATATAAATACACAGGAATAGAAAATATAAAATC

TGAGAAATTGAGGGAAAGTAAGGGACTTGGAGAGAAGATGGAGCTGCAG
ACCTTGCTCAGGCCATCTTGACCTCTTCTTTGACCCCTTTGGCTTCTGGCTT
CTCCTCCTTGGTTTTCTCCTCTTCTGTGACTTCTTTTTCTCCTCGGGATCTT
TTTCTTTTTCATCTTCTGTTTTGACTCTTTTGGCTTTTTTGAAGTTAGAAGA
GTTCTTGCGACCTCCTTCTCCCTCCTCATCAGAATCAGAGAATTCCTCCTC
ACAGGCAATGCGTTTGTCAGAGGAGCAGATGGAAATTCGTTTGTCAGGGT
CTTCCTCATCCTCATCACCGCTCTCTTCTGGGATGGCATCCTCTGGGATGG
CCTGCATTTGGACCCCAGGGGCATGGGGCAGCATTCTCAAGTTCTCAAAG
AGCCGCTGCTTGATCTTCTCCAGGTATTCATTAGTGTTCTGGTTAGTCATAT
TGGAAGGGCTGATGTGAAGCTTGAAATCTGGTCCAAAGTATTCAAAGTAG
TCATTGTATGGTAGCTCATTAGGGATCTCTGTGTCCAGGGCCACAGCTGTC
TCGTAAGTCCAGCACCGAGCGACATTACGGATGGTATAGCCACCTCCTCC
CAACATTAGCATCGGCAAGTTGAAACTCTTCACGAACTCCACACACTTGG
CATGTCCTTTGGAAGGAAAAGGGATGTTGGTTTACATGGAACCCAGCAGA
CCCTGCCCACCTATGCGTGTGCTACGTGGTCTCACCTTTGATGGTCAGATT
GAAGCAACCTAGCCGATCCCCAGACAGGGAGTCTGAGCCGCACTGTAGGA
CCACTGCACTAGGCTGGAACATCTCCATTACTTTGGACATGACCTAAAAA
GATACAACATCTTCGTCCTTACGATCAACCTCCAAGAACAACATGTGAGG
TCCAGAGGTACAAAGTCCCAGCATTTTCTTCCTTTTCATGTACTTCCCTGAT
ACTGTGTGGTAGACAAGAGGTTTCTGATTTGAAAGCATCTGCAAAGATTA
TCACAGCCCCAACGACTGAGCTTTGAATGGCTTCCCGTCAACTCCTTCCTC
TTTTCCTTTTTTAACTGCCAACTTCCCAAGCTGCTTCCTTTCAGAAGCTTCT
CAACCTCTAGCTGCATCTGCTCTACTCCTTTGTCCCTATAGATTTAATGTAT
TTTTGTATATTTTATTCTCAAACCTAAGCATATGCACTGTTACTCAGGAGC
CTGTAAGTGGAGCTTCTGTCATGTTACCTTTCTCTAAGCACATGTGGGCAT
TTGTCTGTGACCACACTAAATACTTGATTAACAGTCTGTCTGCCACTTCAC
TCTATGTGGCATATATTCCTTTTGTCCTGTGTCATCCCCTCAGTCTTAACCT
CTTTCTTCACATAGGTACTTCCTCATTTAAAAAAAAAAAAAATCCAGCTCAA
GGCCTGGCATGGTGTCACAATTTAGTCACAGCACTCAGGAGGCAGAGGCA
GGTAGATCTCTGTGATCCGAGGACAGTCTAGTCTATAGTGAGTTCCAGGC
CAGGATTACATTGGTGAGAATCTCTGCCGTCCACACTACCTATCCCAGGCA
CCCCTGTAAAAATACAGGCTCCAGTCAGCCTACTGAGGACTCTAACAGCA
AGCCTGTCTGTACAACATGCCACACGGCCTTTCCAGAGTCTCTGGATAGAC
TAAACAGCTAGGGGCTCTACCCTATAAAGACTAG
>LNC_008766
GGAAGATCCGCCAGACTGGCCAGAATCCCAGAGTATTTATATGCTTGGGT
CCATCAGGACTTCTAGGCAGCTGCCCGGCAATAACCCCCACCTGGCTTCCC
CTATCTGGTGTGGCAGGTGACACTGAGGATCTGGGTTCAGCCCACAGCCC
GGGCAGACAGCCGGACATGACCTTGAGCCAGGCCCCACCCGAAAAGAGG
CCAGACCCGGAATGGGAAGCATTGGCAACTGGACCCTGAGAGTCCCTCTG
CCAGGCAGCTAGGGCTGAGTGCTGAGGGATTCCAGGAGTTCTGCCACAAG
GGGACAACTCCAAGTGGGTGTCAGACCGGGGGCTCCACAGGAACGCGAA
GCTTCCCTGGCCTTCCCATATCTCTGGTCTGCTGTCAGAAGCCCAAGCCTG
GCAGCAACAGGGGACAGGGCCACCAAGCCTAGAGAAGATGGCTCTCTAG

AGCTGTAGCTACCGCTGTCGCTGCCGCCCAAGCCCGGTTCGGCGAGGTGC
TTCCTCTCCGCCACTCCTCAATGGCGGTGACTGGAGCGCTAGACTCGACCA
ATCAAGTGGCTATATGCCTGGGCCTGGCCAGCCGAGCACCGAGCCGTCGA
TGGGGCGCCCCCTAGCGTCGGCAGCCGTTCCTACAGGGCTGCTGGTGCCT
CCCAGGACCATTTTACAAGCTGGCCTGAAACCCCCTATGGAGCTAAAACT
CATCTTGGACTCCTGGTCCGTCTGACTCCACCTCTGGAGGGCTGGAATTGC
CATGCGAACCCGTCAGGACCAGCCTTCCCGGCGCTTGATGCTATTTGAAAT
GCTCTGTGTAGTTGTCTATTCCTGCATTAGCTTTCCTAACCCTGACTCCAAA
GCAGATGCTAATGTTTCCTCAGCAAACGCTGTGTCAGCTCCAGGTAATGGT
ACACACGTGCAATCCCTGCACTTGAGCGGTGGAGACAGGGGAGGTAGGC
GTTCAAGACCCTCCTCAGTTCTATAGCTAGTTTGAGAGCAGCCTGTATTAC
ATGAGACCCTACCAGGAGGGAAGGGAGGG
>LNC_008768
GTCTCTGTCTACTGTCTGTGTACTTTTTGAGTACTGCGGATCAAACCCAGG
GCAAAAAGCACACTGGACTACTGAGCCCCAGCCCCAGCACCCTACAACAG
AGCAATCCTGCCTGTTGGGGCATCAGGATTTCAGTGTGTTGGGAGAGGGT
TCTTCCTAAGAGTCTTGCACTAATCTGAAGGGCCTCTGTGCCCTACCAGGG
GACTACTTCCTCCTACTTACCTCAGACACTGCCCTGCAGTGTCAGCATTCA
CGCTGAAGAGCTGTGCTATCCTTTCTCAAAGCATATGGAAGAGGATGTTA
ACTCCTTAGAAGGGAAAATTCTGGGGCCTTTCAGAAACAAGAATCAGCCA
GAGCACCCAGGAGATCCAGAGGGTCACAGAAGAACAAGGGCTTCCTGGG
ACTGCCCCCATGAAACACCAAGGCTGTTTTGACCACTCTAGCTGCCTCAG
CCCTCAGTTGACCTGCAACTGGAACTGTAGAGGCCACTAAAGGAAACGGG
CTCAGAAAAAGTCATGAAGTTCCATGGGTTCAGGGCCTAGGGGGGTCCCT
TTACAGGGTGTTGCCCCTGTCATGGGTCAAGGCACTTTGACAGCAATCTGG
TCATACCAGCCTGGTTGGTTGGGCTTCATACCCACATCAGGCACACCAAG
GGGAAAGGGCTTGTCATGGTTCTGCAGTGTCTCTGTGGTCTCCCACTGGGA
CTGAGAGGGACACTCCTCCTGGGAAAAGGGGACCTGCTCATCCTTTGGAG
ACCCTGAAGGGTTAGCATCTGGGTCTCCCACCGTGGTGCTGGCTTGGGAA
GAACACTGAGAGCTGTGGTCAGAGCTGCTACAGACGTTCACGATGCAGGT
GACGTTGACATGGGTCCCGTGGCTGCCATGGGAAGAATCTGAACTCCTGG
AGCCGGCACAGGCTTCCTGAGACCCTTGGGCCTCCGCTGTGACTCTTGCTT
GGGGATGACCCCCAGGGGGCGCTCTCCTGTCCCCAGCGCTGGCTGAGCTC
TCCAGGGAGCTGCTGCTGGAACTGGGTGCTGTGGTCAATAGGTGCTGCTG
TTCAAGGCCTATTGCATCCTGGGATTTGTCATCAGGCAGATGAGGCACCAT
GGTTTCTCGTTGTAGGCAGGAGGGCTTCTTTTTCCTCTGCACCAGGATGAA
GCAGTTCGCCAGTCCTAACATCAGCAGACCCAGAGTTGTCAGTCCAACGA
TCAGACCAATTGGAAGAGAGATGCCACCCGTGATGCTTGGTTCAATGATG
GGGGTTGAACCCAAGGACACAGGGATGTGTGGAGTTTGGCTAGGCCCTGG
CTCTTGATCCATGGGCTGGGATCTTGTGGGCTCTGGCTGAGATACGTAGAT
TGTCCTTGGAACAGCGCTTGGAGTTGGGGACTCGGATGCACAGACTGCAT
CTGTGCTTGCATTTCCAGGAATAGCCAGGATGCTACAAATGCGGTGGGGC
CTGCACACATCTGTGGATGATGTGGTGTCAGAGAACGTCCCTGGGGCACA
GGCACTGCATATCACGTTTCCATTTGAGGTTCTTGAACGGGCCACTCCGAA

GCCAGGGCCACACTTGCTCAGCTTCATGCACTGTCGACAGTTCCCAGAAT
GCAATTTCAAGGCACAGTAACTGTCAGCGTTGCAAGCACACACTCGGTTC
TGTTTTTTAGTGCAGTTGTGGGTCTCCACCTGGTCATCACTACAGGAAGAA
CTGCAGCTCAGGCATGTATGCAGATGGTTCCAGACCTGGGTAAACATGCC
TGCCGCACAGTCCGCACACACGGTGTCTGAAGTCTTGTTGCAGAAGTGTTT
TGCATACTGGCCAGGGGGACACTTAGCACAGCACATCTGAGCCTTCTTGT
CATAGTACTCCTGTGAGATCTGGCACTGGTTCCCAGGTTCTGGCTTGTAGG
GTGTCAAGACAACCTTGGCGGGCACTGTGTGCCCGGTGGCCCACAGCTGC
AGTTCGACGACCAGCGCGACCCAGAGGGCGGCGGGCGCCATAGGGGCGG
CGGTAGCTCCCGCTCTTGCGCCTGGAGCTCTCGTGCCTCTGCTGCGCTCTA
GCTGGTGACTGAAAG
>LNC_008960
CAGCCTTGGCTATCTCAGTTACAATCGTTGTACCTGGGTGGTGACTCAGAA
TCTTTCTTAAGCAAAGATATAAGCTCCTCACACAGCACTTCTTTTTATTTCT
TTATTTTGGGGAAGAAGGTTGTTTGGCTTGTATATCTTGAACCACATACAC
AGTGCATTTATAACATGGTTGTCTTTGTTTCACCATCAAAAGAGTTTCAAA
GTTCTCTAAGTCCGTTTAAACTTTTACAACTTTACTATTTGATTATACTTAA
AAAAAAAAAAAGTAGAGAAAGACTGATCGTTTCTTTGTAAGAAAACATG
ATTCCCATTTGATTGCTCCACAGCCTAAGTATAACAGAAACACAGCACGC
CTAATCTTTTTGATATTCAGATAGTTTTGATTATTTTACTATGACAGGTAAT
CTCCTTTACACATGATTCTTTGAATGAATCTCCCATAACTTCAAGAGAAAC
TCCTAGAATACCTAGGTACCAGAAACATGGCACCAGTTTACAACCATACA
GTCCCATCTCGGGCTCATATTGGTTCCTTAGCAGTGTAGGGAAGAGGATGT
TCTCACCAGTCTTCCTTGCTGTCAGTTTCATAGGGAGGAACGGAGACAGAT
GTGCTCTCGGACTTTGTTCCCTTACACTTCGGCAGGCTGTCTGCTTTCTTTC
TGGGTCCTGGAGGAGTCGGGGTCTTGGCTGAGTCTCCAGCAGTAGCTCTCT
TTCTTTCCAGGCAAAGGCATGAAGGCTGCACATCTTTCCCATTTTTGTTTA
AGCAAATCCCCCAAAGAATAGAAGTAGAAAGTCTACTTATGTCAGCCATG
CTGTTTTAATGTCTTGGTGGTAAAACAGAAGACTGGGCCATGTTCTTTAGG
TTACGGAGCGTTTCTGACCATTCCATTGACCCTATCTGAGGTATAGTAGTT
AGGAGTAAACTTTTGGCTTTATTGGCATTTTCTTTCATGGTCTTTAAAACCC
CATCCACTGACACTGCTTCTTCATGCTCCTTCCAGCAATCATAATCGGTTG
CCATGGCAATGCTTGCGTAGCACAATCCGGCCTCCTTTGCAAGCACCACCT
CTGGAACAGTGGTCATATTGATAACATCTGCGCCCCAAGTACGGAATATA
AAGCTTTCTGCCCGGGAGCTAAACCGAGGCCCCTCAATTGTGACTATCGTC
CCCTTTGAATGGCACCGGAGTCCAAGCTTCTTAGCCATTTCTATGAGGACC
TCTCTTGTTTTGGGGCAAAATGGTTCAGCCATTGGGATGTGGCACACTCCT
CTGGCACTGCAATGACTTCCATCATAGAAGGTCTGAGGCCTCAGGGATGT
CCTGTCAATGAACTGATCGATGATGACCATGTCACCAGGCTGGATCTCCTC
TCTCAAGGACCCGCAAGCTGTGGTCACAATGACATGTGTACAGCCCTCTTC
CTTCAAGGCCCAGATGTTCGCCTGGTAGTTGACTTTTGAAGGCATGATGGT
GTGATGTCTGCCGTGTCTGGCAAGAAGCACACAGTCGACGTTCTTTATCTT
CCCCAAAATTAAGGCATCAGATGGCTTGCCAAATGGAGTATCCACATATT
TTTCTGTTCTTCCTTCTAAAATTTCAGGGTCATCCAAGCCTGTTCCACCAAT

TATTCCAATCTTCACTGCCGTGCAGGCGGAGCCCGAGGCCATGTCTGCACC
GGGGAAGTCCACGGCGGGCCGCAGCGCTGGACCAAGGGGAGACTGCGGG
GCTGGCACGGCGCGAGAGTGAGCGGTGCAGGGGCCCGTGGGCTCTGGCTC
CTCCTCTTCCTCCCAGGGAGCGCGGGATTGGCTGCCCTCCTGGCACCCTCC
TCCTCAAATCAGGTTCCCGCCTCCCGCCCCTTTTGCAGACTTTTCG
>LNC_008964
CATCCATATTTCTTCCCTTTTTTTAAGCTCCTTGTATTTTTGATTCATTTCA
CCTTGGTGTTCCTCTAAATCCATGTCAAGTTGTCTAATCTCTTCACTAAACT
GATTTATTTTTTCCTTTATATCTGTTAACTGTCTCTCCATGCTGGCTATTTCC
TGATTATCTTCTTTAACCTGCTTAAGCAATCTCTCCCTTTCCTCCATTGGCG
ATCCCATGCTTTTGTCTTCTGCAATCATTTGATCTCGATGGGACTCTAACTC
ATAAAGTTTTTCATATAGCAATACAGCCTCTTGTTTTACCTGGGAGTGTGC
TATTTCAGTTTCTAGGCTCTCCTTTTTAATGTT
>LNC_009039
GCCGCGCCTCTCGTCGGTCTCCGGGCTTTCGCCCTGGGGGCAGCCCGCACG
CACCTCTGCCTTCCCTGCGCGTGCTGGGCTGCTCCCCGCCTCCTTCCGGGC
TCCGAGTGTACCGCAGGGAAGATGCTTCTAGGCGTCTGGTTTTCCTGTGGT
CTCCTTGTTGCTGTCATCCAGAGCCAGTGGTTGAAGATTCTGTCTACATGC
CCTGGGGCCTGACTTTGAATGATGGTCTTTGGATGACAGGCCCAACACCT
GGGCCTTTTTCTTCTGCAGCAGACTCGCTCCTTCCATAAACAATGGACTGA
GGTTCCTCGGATTTTGCTCCAAGAATGACTTTTCAGGAAAGAACTGGATCC
AGGCCTCCCTCGAGCTGGTTCTCTTGTTC
>LNC_009054
TGCACCCTCCGGGGGACTGCGGACTCCGGCCTCAGGGAGCCCAGTGGCTT
GGAAGACCCGGTTATCAGAGGGCGACTCAGAGGAGCTTCTTATCAGAATA
GGATCCACACCATGCCAGTTCTTATTAGCATCCTTAGCGCTCTCCACGAAG
TTGGAATTACTGTCTGGTTGGAGGTCCGGGTGACATACTCCAGGAGGCAG
GCTCCCCAGGTACTGGGCCCCCTCTGCTAGTCCTGGCTGGATAGCAGCAA
ACATCTGGACTAGGAAGCGCAAGGCCCTGGGTTCGGTCCCCAGCTGGCCT
AGAGGCGCCCGCTCCTGCGGGGTTTGCTATGGTTCCTCGGGGCCTGCTCGC
TGTAGAGGCTGGGGGTGGGTCCTGGACCTGCGCGTGCACGCGCGGTGGCG
CGGCTGCCTGGTCCTGGCACAGCC
>LNC_009128
AGCTGGTATACTTCCCATTGCATGCTGTTGGCCCAGCATTACCTGTGTAAC
CACAGGTAATGAAGAGAGGTCAGGGCAGCTGTGGTGGGGAATGCAGCTTT
GCCTTTTGAGGTCCCGAGAGATTCTCTGTAGAACCTACCAGCCTGTAGCTG
GTCAGGGTCACCGCTGTGTGCTGGAATAATTTCAAATCTTGCTTCCACGGT
GTGGAGAGGCTGACCAGTATATAGTCCTGATGACTCCAAATGGCTCTGCA
CATGGCTGCTAGCTAATCCTGTGGGGGCTGGGACTATGTGGGACTACGTG
GCAGGTGAGCATGGTGCTGGGGACACACTCCAGCGCTGGTTGTCCGAAAT
CCTCATCAGGGCTCAGGTCTGGAGCCATGTCCACCTCATCAAGGACAAGG
ATTCCCTGGTGCCTTCAGTCCTTGGCAGCAAGGACTTGGTTGAGACTGTCT
TTAGTAATAACAGAGAGTGACACCTATTTATCTCCTAAATGTTTATTACAT
AGGCTGTACTCAGAGACACTCCGGGGGCTTGAAGGGACTCACGCCAGAGC

TGACAGCCACACTCAGCATGGACAGGGTTAAGCCAATGCCCGCTTCTCCT
TGTAGGTCGCCATCATCTTCTTGATTTCTGCAATTGCTTTTCCTGGATTCAG
ACCCTTGGGGCAGGTCTGTGTACAGTTCATGATGGTGTGGCAGCGGTAGA
GAGAGAAGGGGTCCTGCAGCTTGGCCAGGCGCTCCTCTGTGAATTCATCT
CTGGAGTCGATCATCCAGCGATAAGCCTGCATGAGAACTGCAGGCCCCAG
GTACTTGTCTCCGTTCCACCAGTAACTGGGACAGCTGGTGCTGCAGCAGG
CACACAGGATGCACTCATACAATCCGTCCAGCTTCTCCCGGTCCTCGATGG
ATTGCAGATACTGTTGCTTGCCCTCCTGGGACTCATCCTTCTTCTTCAGATA
GGGCTCAATGGATTTGTATTGTGCATAGAAGTTACTCAAGTCAGGGACTA
GATCCTTGATCACATACATATGCGGAAGAGGGTATATTTTCGAGACTTTGC
CAAGGTCTGTGTCGATCCTGCGCGTGCATGCCAGCGTGTTGCCTCCGTTGA
TGTTCATGGCACAAGAGCCACAGATCCCTTCTCTGCATGATCTTCGGAAGG
TCAAAGTAGAATCGATTTCATTCTTAATCTTGATTAGAGCATCCAGCACCA
TCGGTCCACACTTATTCAGATCCACCTTGTATGTCTGCATTCGAGGTTTAT
CTCCAGCCTTGTCCGGGTCCCATCGGTAAATGGCGAAGGTTTTGATTCTGG
GAGCTGCAGCAGCAGCTGTCTGTGCCTCTCGGCAGGCCTGAAACTGCAGG
CCGACTCTGCCGAGAGCCGTAGCCGAAAAGCCGCGCTTCAAGGAGACCCC
GACCACCGCCGCCATTTTGGCTCCTGACGTCAGCCCCACCCCTTACCCTCC
GGCATCCCGTCGAACACCGCGGTGGGAAGCCGGCCTCTCCCCTTGAGGTC
CGCTAGCAGCCTAAGAGCCAACGAGTGGAAGGCAGCTTCCGGTATCCCGA
GTAAACGGACAGGAAGTGCGAGTGTGCCGTAGCGGCCAGTAGGGCGCAT
GCCCACTGGGCTTCCTATGTTCCTAAGGTGACCTCTGGAGTCGGA
>LNC_009165
ACACAGGAGAGCACCTGGGATCTCTCCGCTGCACACAGGAGAGCACCTGG
GATCTCTCCGCTGCACACAGGAGAGCACCTGGGATCTCTACGCTGCACAC
AGGAGAGCACCTGGGATCTCTACGCTGCACACAGGAGAGCACCTGGGATC
TCTACGCTGCACACAGGAGAGCTCCTGGGATCTCTACGCTGCACACAGGA
GAGCACCTGTGATCTCTACGCTGCACACAGGAGAGCACCTGGGATCTCTA
CGCTGCACACAGGAGAGCATCTGAGATCTCTATGATGCAGGATTGCCACC
CCTTGTAAAAATCTCCCTGACCCAAACAAGCAGCCGCTGAGACCCATGTG
GACAGAGGAGTCTCATCTAATAAAAAAAAAAAAAAAGTTTTCTCCCATGG
AAGACACAGCCTTCTCCTGCTTACATTTGCTCTCTGAGGTCGAGAGGGCAC
CAGCCATTGCTCGGAGTGGTGGGAAGGACAGGTGGCCCAACCCCAGCTGG
GAGATCTTCTCAGCTTCCTTATACACCACCTGAAAAACACGATGTGGCCAT
TAGCCAGTCTGTGCAAGAGCCAGGGAGCCCAGCGGGCTGTGGTCCAACTT
AAGTGCCTGACACGGGGGTCTCTGGGAAGAGCAGCAGCCAGTCTGACCCT
CTGCAGTGAAGGACAGCTCAGCTGCCACCTCAGTCCTGCTGGCCAGGCTG
CCCACGTGGCTGTTCACATGGGAGACCAGGTCACACCTGTGCAGCAGTGG
CCTGTGTGTGCACTGTCAGCGTGAGGGAGAAAAGCAGGCCGGGAGAGGA
CAGTGAGCGACAGCAAGGCTGTGGAGCGCAAATGGCTGGATCTTGGCACT
TAGTGTGAATCTTAAAGGCCCGTATGTGGGGGAAGGCAGGTCATATTGGG
ACCTCTCGTCCTTCCTCTCGGTTTCCTGGCCAGAGCAGCTGTAGGTCAGCT
CAACTCAGCTCAGCTCTGCCATTGGTCCCTACTACAACATGCTGCCCAAAA
TAAGTGGTGGATTGAAACCTCAGACATTAGGAGCCAAAACAAACCCGTTC

TACTTTAACTTCAGGTTGATCCTGTCCCAAGTACTCTGTCGCAGTGACACG
AGGCTCAGGCTAGCTCACTATTAGGTTAGGTAGGTACACCACAGTCCTAG
GCAGAGATACTGCCATAACACACGGTGGAGACTTAGATGCACAGGCAAA
CATTGGCAGCCTGCTTGGCCGGCCTTGTCATCTCTAACCTGTGTTGCGGCC
ATGGTCAGCTACTGCTATTGGAGGAGCAGCCAGACTAAAGCCTACCCTTG
CCCAGCTTCTCCTTCCTACAGGGTCCTGCTGACATAGGCTCTGGGGCAGCT
AACTGAGCCCTGGGCTATGGCCCTGGCCCTTCCTTGTCCAAGGGAAGAAG
AAATCTGTCCTTGGTCCAGACCACTGACAGAGGATAAGAGGACTCAACAC
TCCTGGGCTTCAGACTGTGCCTTCACCGCTGCACTTCCTGGTTCCTGGGCC
TGGGCTGCCCACCTTCGTGTCGATCCACAGACCAGCTCTTCCGGCCCTCTC
TGCTCGTCTGTGTGCTCTACTCTGGCAGGATGGCCCATGTCTCCCCAAGGA
TCTGCTGAGGACAGAGGTGCAGTCCACAGAGGAGCCTGGACTCCACATGC
CCTTGACCAAGGAGGGGATGAGGGGCAGAACAGGGAAGACGACGTCACA
GCAACTGAGGAAGGTCAAGGGCAGCTCACCGCACTGGAGAAGCCGCGGT
GCCCAGGTGGAGGAGGACGCAGACTTACTGTCTTGGGATTATTAATGGCT
GCAGGATGCCCAAACTCCGTGATCATCTTCCAGGTCACGTGATTTAGGAG
GCGCACCTGGGAAGGAATGGTGAGAATGACTTGCGGGCAGCTCGCAGCCT
GCCGGCATCCTGCCGCTCATGGAACTGGCCTACAGCTTATGGAGTTGGTG
GCCTGCAGTGCCCCAAGCCACTCCCTACCTTTCCATCGTAGCTCCCAATGG
CCAGGAACTGACTGCTGGGACTCCACGCCACAGACTTGATGCCCAGGGAC
CACTCATAGGCACAGTATGCTGACAGCAGGCGGCCGTCCAAGGAGTATAG
CAGAACCTTGTACTGCAGGTGAGACATTTAGACGTGTCACGGGCCCCTGC
GAGCAGCGCTTCCGCACCCAGCAGCTCACTGCACTGAGAGCACACAAGGT
ACCTGCAGGTGAACTCTCTTGAAAAAGGGTGCTGCTGGCCAAGTGCTATA
CCTCCAAGCAGGTGTCCCACGCTGCCAGCACACAGCCATTCGGGGCCCAC
TCGATCCCTGTGAGATCCTGGGTGTCTGTGTCAAAGTGCTTCCGGACAAGA
GGAGACATGACAGTCTGAGAACATTTCCTTAGATAGGTGGTGTGCTTATTT
TATAACATGCTCTTGGCTCCTGTGTCATTTTATGTGCAGCAACAGACAAGG
TATCAGAGTGCTGTCCTGTGAGCGCACACAGTGTGCCAGCCCCTCTGGGC
ACGGCCTCGTCCCCTCATGCCATCCCCGTGGCACCTGTGCTGTTCTCCCCA
GCCTTGCCTGGCAAGCCTCCCCAGTCGGCCCCTTCAGGTGTTCTCTAACAC
AGACACTGAAACGCATTTGTGGGCCTGAAGCTGTGGGTATGGATGGTGGA
TGCTCCGTGCCACAGCTCTGTGGCAATCTCCTCCCAGGGAGGAATTCCCTT
ACGCAAAGAAGAGGGTTCAAAGTCCATGTCAACATGCCAACTTCCAATGA
CCCCTGCCCCCTGGCCATAGAAACCCCATCATTCAGTCTACCCCAGGGCTT
CGACAGCCTCTGGGATCATCAGGAACTCTGGTTTTACCCCACTTCCCAGCC
AGCAGCAGAAAATGGCCCTACCCGGAGGAGCTGCCAGTCACTGCACACG
AAGATGCTCACGTAATCTCTGCAGTCTCGCCTCTCCGCCAAGGCCAGGTA
GCGGCCATCTCTGGTGAAAGTCAGTCCCTGCTGGCAGGCTTTGGGGTACTT
GATGTAAGATACAGACTTGGTGCACAGGGACCAGACGGTTATTCGCAGGT
GGAACTCCGTTGTATTCAGAATGTGGCGGCCGTCTGGACTCCAGCAGGAA
GCGACCAGCCCTGCAGAGCCCTCGTCGATCTTGCAGTGCCATTCTGGCTGC
TCCAGTGACCAGACCTGCACAATCCCCCGTCTGTACATGGCACACAGAAT
GAAGAGGGAGTCTGCT

>LNC_009166
ACACAGGAGAGCACCTGGGATCTCTCCGCTGCACACAGGAGAGCACCTGG
GATCTCTCCGCTGCACACAGGAGAGCACCTGGGATCTCTACGCTGCACAC
AGGAGAGCACCTGGGATCTCTACGCTGCACACAGGAGAGCACCTGTGATC
TCTACGCTGCACACAGGAGAGCACCTGGGATCTCTACGCTGCACACAGGA
GAGCATCTGAGATCTCTATGATGCAGGATTGCCACCCCTTGTAAAAATCTC
CCTGACCCAAACAAGCAGCCGCTGAGACCCATGTGGACAGAGGAGTCTCA
TCTAATAAAAAAAAAAAAAAAAGTTTTCTCCCATGGAAGACACAGCCTTCT
CCTGCTTACATTTGCTCTCTGAGGTCGAGAGGGCACCAGCCATTGCTCGGA
GTGGTGGGAAGGACAGGTGGCCCAACCCCAGCTGGGAGATCTTCTCAGCT
TCCTTATACACCACTGTCTTGGGATTATTAATGGCTGCAGGATGCCCAAAC
TCCGTGATCATCTTCCAGGTCACGTGATTTAGGAGGCGCACCTGGGAAGG
AATGGTGAGAATGACTTGCGGGCAGCTCGCAGCCTGCCGGCATCCTGCCG
CTCATGGAACTGGCCTACAGCTTATGGAGTTGGTGGCCTGCAGTGCCCCA
AGCCACTCCCTACCTTTCCATCGTAGCTCCCAATGGCCAGGAACTGACTGC
TGGGACTCCACGCCACAGACTTGATGCCCAGGGACCACTCATAGGCACAG
TATGCTGACAGCAGGCGGCCGTCCAAGGAGTATAGCAGAACCTTGTACTG
CAGGTGAGACATTTAGACGTGTCACGGGCCCCTGCGAGCAGCGCTTCCGC
ACCCAGCAGCTCACTGCACTGAGAGCACACAAGGTACCTGCAGGTGAACT
CTCTTGAAAAAGGGTGCTGCTGGCCAAGTGCTATACCTCCAAGCAGGTGT
CCCACGCTGCCAGCACACAGCCATTCGGGGCCCACTCGATCCCTGTGAGA
TCCTGGGTGTCTGTGTCAAAGTGCTTCCGGACAAGAGGAGACATGACAGT
CTGAGAACATTTCCTTAGATAGGTGGTGTGCTTATTTTATAACATGCTCTT
GGCTCCTGTGTCATTTTATGTGCAGCAACAGACAAGGTATCAGAGTGCTGT
CCTGTGAGCGCACACAGTGTGCCAGCCCCTCTGGGCACGGCCTCGTCCCCT
CATGCCATCCCCGTGGCACCTGTGCTGTTCTCCCCAGCCTTGCCTGGCAAG
CCTCCCCAGTCGGCCCCTTCAGGTGTTCTCTAACACAGACACTGAAACGCA
TTTGTGGGCCTGAAGCTGTGGGTATGGATGGTGGATGCTCCGTGCCACAG
CTCTGTGGCAATCTCCTCCCAGGGAGGAATTCCCTTACGCAAAGAAGAGG
GTTCAAAGTCCATGTCAACATGCCAACTTCCAATGACCCCTGCCCCCTGGC
CATAGAAACCCCATCATTCAGTCTACCCCAGGGCTTCGACAGCCTCTGGG
ATCATCAGGAACTCTGGTTTTACCCCACTTCCCAGCCAGCAGCAGAAAAT
GGCCCTACCCGGAGGAGCTGCCAGTCACTGCACACGAAGATGCTCACGTA
ATCTCTGCAGTCTCGCCTCTCCGCCAAGGCCAGGTAGCGGCCATCTCTGGT
GAAAGTCAGTCCTGTGGGGAGAGAGGGTAAACTGGAACCAGAGTGTGGG
AAAGCACCAGAGCTCGGCAGTGCGTTTTTAAAAGGGACAGGGAAGAGGA
CCGATCAAGAGAAGGGGACACTGTGACATGGGAAGCACTGTCATATGAC
AAGCACAGCCCTTAGCCCTCTTGCAGACAAGGAGAGGATTTATATAGAAT
CAGACCCAAGACCTGATCCTTAAC
>LNC_009192
ATAGGCATTAACGTTTTATATTTTTAAGTGACCAAATCTAATAAAATGAAA
AACAAAACAAAACAAAACAAACAAAACAGGTTTACTAAACAGGTTCAAA
AGACAACACAGGAGATAAGCAGTAAAACCAAAGCGTGAGTGGCACTGTC
CATGTCAACCTCCAATCTCCTCCAGATCACATTTTGGTTTTTGCTTTCTGTT

TAAGGTTTTTAAAAAGTGAAGCCCAGGAGTCCGGCAGGCCACCCTGTGCT
CAGCAGCATGTCCGTCTCGCCTGCATCAAGCTCTGGCCCCTGTGTCCCTCA
GCAGCAACTGCAGGTTGTGGCTTGTGAACCTCACTCCTGTGCTATGCTTCT
CAGCACGTATTTGACCGTGTAGGCAAAGGCCGCAAACCCAACTATAACAA
ACAAGTTCGCCAAAGCGCCGCCCAGGAGTCCATGATGCCTGTTGGCCTGT
GGGAGCCCAGCAAGGTTTGGGCCAGCAGCAGGTGCATGCCCATTGAGGA
GCTGGATCCCATTCTGGCCACGGTGCATCTCCCCATCAGGAACCACGTCTG
GCAAAGGAAGATTCTGGGGCCTGTTACTTAGTTCATCTTGTGCTTTCTGTT
TCTGTTTAATTTCCTCCACAACTTCAGGAAATAATTCACAGAAGACTTTAT
CTTTTAAATTAAACACTAGACTCTGTGCTGCCAGCTGTTTTTCGTGAAGT
CCGATGTCTCTATGCTCCCCAGGGTGGGGCCTTTCTCCACCATAAAGCTCA
GGAGCCCTGTTAGAATGGTGGAGACGGACCACGCTGGATTCCATGTATCT
GGATGGAAATCTGTGATGGAGAGACACAGCCTTGTGTTGCACTTAAATCT
TCCATTGGGGGTTATCATGTAAATACTAGGAGGCTTAAATGGAAATTCTCT
GGGAAAAATTAGTTTTCCATGATAATAGCCACCTACGCCGAAAGAAAAGT
AAAATATCCTTGTCAGTAAGCCTCGTCAACATCCAGTGCAACTGCTTCAAC
TCCCACCAACCTTACCTAGTTTCTAGCTGGCATCCCAAGCCCGTGGTCTCC
CACTACCCTCTCCCTATGCTGGCTCACAGGCTCAGCCAACTACTGTCTTTA
TTACTCTCCAGGGCTTGCGCTCTCGTCCAACACTTCAGCCTGTCCCCCAGT
ATCTGACTCTCCATCTTTTACACCTATACCCAAGGAGTCTTTAATTACCAC
TTGTTCCACTAAAGCTATAAAACAACCCTAATCTTCATGTTCCTCCCTTTCT
GAGAACTGAGTCATTAGAAGACAACTTGCTACAAATCCCTGCCTCCTCCA
CGACTTCATCTGGTCCCTAGCAAATGCATTCTGAGCACCAGCATTTGTCTG
CGGGGATGAAAAATAAGAAGCACCCACTTGCATTTCAAAGAAAAGGACC
ATAAGAATAGATGTCAGGCAAGAGCCTGGTGAAAC
>LNC_009316
CTTCCAGTTTTGTTAGAGCAGCCATAAAGCCGAGGAGGATGATCTTCAGC
CTGTGTTTCCAGCAATGGTGAAGTCTGGTAGTCTCCTCTTCCTCCAAGAGA
GTTCCAGAATTCATCTGGTTCCTTGCCTTCTTGAATTCTTGTGGTTTTGCAT
TTGAGGACATCAGCCACATACTCTGCTCCTTTCTCCTCCTCCTGGCTAGCC
CCTTTTCCTATCCAGATGAAGCCATTGTTTCGTGGCAGTTTCAGGACAAAA
GTGTCATTAGAATTCAATGAATTTGCATCCACATCAACCTCCACGATTCTA
GTGATAGATGCCAGGTTCCTCCGGACTTGAAAGAGGCGTG
>LNC_009333
GTTCCGGCGAGGCCGCGCGGCTGTCCTCTCAGTTCGTCCTCCTGTTAGGGA
ACCCGAACCCGGATCCGGGCGAGTAGCCGACTTCTTCTCCCCGCCTAGGG
ATACCCACAGAGCGGCCGGGAGGGAACGCTGCTCCTGGAGTGGTGGGCA
GCTGTTGGGAGCTGCTCAGGCTGGAGCCACGGGATTTTAAAGGACCTGTC
TCTGTCATTTTTACTTTTAGGATTTTGGCCAAATTGGGCGAGGGTACAATA
TTACCACTTACCCCTTCTCACCGAGGAAGAGCGGGAGAAAGGGTATGGCA
CAGTCACAAGGCTGGGTGCGAAGATGCTTCAAGGCCTTTTGCAAAGGCTT
CTTTGTGGCGGTGCCTGTGGCAGTCACTTTCTTGGATCGGGTGGTCTGTGT
GGCTCGAGTGGAGGGATCGTCGATGCAGCCTTCTTTGAATCCTGGGGGAA
GCCAGTCCTCAGATGTGGTACTTCTGAACCACTGGAAAGTGAGGAATTTT

GAAGTACAGCGTGGTGACATTGTGTCATTGGTGTAAGTAATTCAGAAACT
TACTGAATTTGTGTTTGTGTTTTTCTGGACTTTGGTGATGATTTACTGCTGA
TACTACTTGTCCAATTATGATATCAGTGAGTGTATCTTAAATATAATCAGT
ATTGATATACAGCAATTGTTAAGGTTCATGCAGCATAAGTACCTTATCAGC
GAGAGCCAAGGAATGCTACAGGGATCACAACACCCAACAGATCTCATGTA
AGAGGCATGGAGTGGTAGGACAGCTTAG
>LNC_009388
CACAAAAATATATTTAATAACTTGTTATATAAAATCAAACACAAAATTTTA
TCAATAATTCAAAGTCATTTCTATCAGGGAGTGCTTATATTTAGGAAAAGA
ACTAAAATATATACATATTCACAACATTTGTAAGCACAACTGAAGAGCTA
TGATGAAACTCAAGAATTCTAGATTTACAGCTGGAAGTTCCATAAGTTTAT
CTGTAATAAAGTGTTCAGCACTAATTATGAACAACTATTAAGCCCAAAGC
AAGAAAAATAGGAAGGAATGACCTGTTTCTAAACCTTTATGACAAATACT
TCTCAAAGATAAAAAAGGCAATATGAAATTTACTTAAGTGACCAAGATTC
CTCATTGGATCCCTGACTGTCACTTTTGGCACGTAGCCCAGCCTGATGGAA
GACAGTATTCCCTGTGGTCAGGAAGCCCGTGCTGTGTCTCACCAGAGCCTT
GCTGACTGTACTCATGTGCTCTGGCTCAAAGGAAGGAAGGAAGCAGTATT
AGCAACAGTTTCGCACCAACTGAGGCCCGTATTTATTATATTCCCGTATTC
CTGACTCTATTCCTGAAAGGCTTTATCTCCCTCCTTCAAAGGTGTCCCTCA
CATAGGGAGGAGGTGGGAGTAGATGACCACACACACCATATACACGTTTG
TCCCCATAAAGGCTCCATGCCCTGTTTTATTCTGAACTATACCCATGGCA
ATGGTCACACCATCTCATTCCAGAAAACACCTGACACCACAAGGAGCCGC
TGTTCACAGTGTGCTCTCGTCCGCTTCCTTTTCGGAACAAGAAGGGTGTGT
CCACTCCTGTTTAATGCCATTTATGACAGTCTCTTCCTGTACTCCTTTCCAT
CCACAGTCTCCAAAGCAGTTTCCTAACAGCATTTGGTAGTATATGCCATAT
TGGCTTTTTAAGTGATAAAACTTTCTTGGTTCAATAAAAGAAAAAGTTGAT
AATATATGTAAAACTAGAATTTTGTAATTCCAAATAGTAAGTTGCCTTTAT
TAACCAAAGAACTTGTTTGCAGTCATTTATCTGAAAATATTTTAAAGCTCT
TACAAAACCTGGCTTGTGGAAAGTTAACTTAAAAACTTCTTGCCATGGTGC
TGGGCATACCACTGCTGCCTCTGCTTGCGGTTTTCCAGCTCGGTGACAAGA
GCCTGTCTGTAGGCCTCATACAACTTGACAATCTGCTCCAGCTCCTTCATG
AAGAGCAGCTTCAGCATTCCCTGGTACGTGTCCAGGTCAGCCAGCTGGAA
GAGCTCCCACTTCAGAATGTGGTCGTATTTCACAGGGGCTCGGGCATAAA
CCTGAAGCCAGTCAGGAATTTCTGCAGTATGGTAGGGGTTTGCAGGGACC
AGGAGAGACTGATTTCTTTCAGTTTGCTGTGGCTGATACGTGATGGGTGGA
GGCTGATCATACCGAGGCACACTGGGAGCACAGGGGTGCCTGTACTGAGC
CCTTTTATTTGTGTGATCCACATAATAGGTTCCAAATTCTGATGATTCCACT
CGCTCCCACCCAGGAGGAAGTCCTTCTCGTTCAAGAGGATGACTCCAATG
AGTTGTGTTTGTGTTATGATCTATGTAGTATTTTCTCCCTCTCATTGTCCAG
TCCACAGACCAGCCAGGAGGAAGGGGTAAATCTTCAGATCCATGGTTAGT
TAAATTTCCTAAAGATGTAGCAGTGACTCTCCCAATACCTGATGTGTGCCT
CCCCTGATTCTGTGGCATTCTTTGGAAGAGATCATGGTTGTATTCATAATA
TCTATAGTCTTCTTGTGCACGATCTCCAAGTGGCCTCCTTCTCTGACCATCA
AAAAAGTTATCTGAATAAAAATATCGGGAACCAGAGTCTCCATTTTCAAC

AGCAAAATTAACTTCTGTTAAAAATGACTGTGATGAGCCGTATTCCCGAG
GGACATCCGCTAGGCTCCTGACAAGGTAAGAAGACGGTGCGGACAGTCTG
TTGCTTTCTCTTCTCATTACTTCATGAGGCGTCCTTTGAACTGGAGTTCTCA
GGAAACTCTGGTTTCTTGAAACTACGCCATCTCCAGAAGCTGAGAAAGCA
CTAGCGCTTGACTCTGGAAGACAGAGGTCAGTCCGTCTTGGAATTGTTGG
ACCGTGCCGAATGAATGAAGGCATGAGATTCCGCAGCAGGGGCGACGTCT
CCTTCTTCACGTACTTGCCCTGAACCTCGGCCGGCTTAGACACCTCGTTTTT
GGTTTTCTTGCGGGACAGCATCCTCGCGGACGTCGCCCGCCGCTGCCCCAG
CAGCCTCGCTAGGGCCGCGAGCCCGCCGCCCGCCGCCGCCTCCCGGGCCG
GCGCCGCCTTCGCCTCGGGTCTCCGCCGCCCGCGCCCGCCCGCAGGCTCCG
CCGCATGTTGGCGGCGCTAAGCGCGCCCGCCGCCGCTCACTCGCAGGGCA
GCTCCTCCCGGGAAGTCAGCCGGCTCAGCGCCGCCGCCCGAGGCCGCCTT
GGGACAGGGCCGCGGCCGCCCACTAGGGCCATGGTCCGCCGCCGGGAGC
CGCTACGGCTGCGGCCACCGCCTACGTCCGGTAAACTTTCCCCGCGCCGCT
GGGAATGCTGGGAAAGTCGACGCGCGGGCCGCCCCTCCCCCGCCGGGCGC
GGACGCGGCTGCGTGCGGCGACTCTCGACCGGCGGCTAACGGGCCGGGTG
TGGCGACCACCCTGGCTCCTCAGCGCGGCTACCCCGGCCCGCTCCCGCCG
CTGAGGAGCCGGAGTGCAGGCGGTCGGGAGCCCACTGGGCTGCGAGCCG
CTCTCGCCTCGGACTTGTCGACCGCCCTGTTGGAGGCAGAACTGCCCACA
GCCGCAGCCGCGAGTGGTTGCTGAGGGAAGGCGGCCCTCGGGTGTGCAGG
GTTGATGGGGACGCGGGACCGGACCACAGCCAGGGCGGCACCTAGTAAC
GCGTATGCCTCTTTTGGTGACAGCGTAAAAACCGAGTATCCGTTTCTTCTG
TCCTCTTCTCTCTTCCCAACCCTTGGTACTCCCACCCCTTTTATGCCTCCTC
CCATCCGCTTTTTCGGATTAAAAAGACAAAGAAGATGGAGTTTCTACAAC
TCCTAAGATAAATTTGACGTTAATGTATTTATCTCATCATTTTAATATTTAT
ACTTAATAACCACGTGCTTTTTCTTGCAATTAAGTCTTAGAATCTTGAGGC
CTTTCAGCACCAGAGTTCAGAGCTGGGAGTTTTCTATCTAATTCATTGGAT
GCTACTTTGTTCTCTTTAACTCACTCAGGTACTCGTGTTTGGAAAATAAGA
TGAGACTCACTCCTCTTGACCAAAGTAAG
>LNC_009601
GTTTGCTTATTAAACATACATTCTCAAACTGACACTTTGCCATGAATAAAG
CAACAAAAATAAGGCAATACCATAAGATGTGCTTTACATACAAACATCAT
CCTGGAAGAGTAGGAAGTCTGAATCCATGTACAACAAAAAAAGCCAGGA
ACAAAAAGCACTGTATTCTGAACACACACAAATGTGTATGTATGTAAAGA
CAGCACACACACACAATACAGTTTACTTGGGTTCTTTATGAGCATAATTCCTT
AATCATCCTTTGGAAGAATGAATCTTAAGAAATAAGAAATCTGAACTGCA
TTTATTTTCACAATATCAATGTTAAACTTAGGAGAAGCAACTTCATTGACT
AAATGTTAAATATTTCAAGAGTAAAAGGCAACCTTAATTTTTAGTAACCCT
CTAAACAGGGTCTGGAAAACAGTAGGTTTTCCTCTCTTGGTATGAACAAA
GGATGGTAAGCAAGCATCATTGAGAGTGTGGACACATAGAAAACCATCTT
GGGAACCGTGCTCTTCACAAGCCTAAGAAACCACCAGAAATAATGAGTAG
ACCCCATGCAACCCAAATGCAAAGCTAACTCTATACATGTTACAATTTTCA
AACTCTTGCTTCTAAGACAATTTTAGTACACCTAATAAGTACAGCAGGTCA
TTTAGAAAAACAGAAACAAAATATATACCCAGTCTTTGCAATTCAAATTTT

TTTTAACATGAACAATATTCAAATATAAAATTGCACTAATTTTTCTAAAT
ATGATTTATGCAAATAGGTTTCTTAAACAAGCCAAGCCTTAAGGATATG
ATGTGTACAAACTACCAGTAGTGTATATTCCAAACATTCTTCCTATATTAT
GATACAACATAACTGTTCTCTGAGTGAAGGTTTATGTTAAGTTTAAGGTGT
AAACACTTTAGAAGCAACTTAGTTAAATTATTGTTCAGTTAAAAGTAACAT
CATCTCATAACAGGCTCCTGAATTTTCAAACTTACATGTAAAAATACATTT
ACATCTTAAATTTCTAGAAAAGGCACGTTTTATGTAGAAAAGCAGCAAAT
TTTTAAACCCACAATTTACTGAAAAAGCCATTACTTTTTATTAGAAACAAT
AATGCTTCTCAAGATGTTGGCAACAAGGCATAGTATAGCACACATTTCAT
ACAGTGTATTTCTCCCTTTAATTACATTCCAGAAGTGGGTGGGACACCTCC
TTGGCCATGAGAAGCTGACGTATTTGATGGACTATGTAAGCTGGTCTCCTT
GTACACAAACCAAGCATTTCCTCCCCAAAGTATCATATTCAGAAAGCCAA
AGATCACGGATACATTCAGGGACCCCATACTAGTCACAGAGACAAAGTAA
CATGACACTTCTGACTTACAAGGATCAAGTTCTTCAACAATTCTGTGTCCA
GTAGCTACCTTAATATCTGTAAGAGCTTTAGCCCAGGCCGAGGAACTCAC
CAACCACAAAAAAGTAGCAACAAGAGTAACAATAAAGTCGATCATGGGC
AGTTTGCGACTATCCCGGTAGAGGTTGGTGTAACCAACATAGAGGAGAAG
GGCAGCAATGCAGTACAGGAACACCAAGACTGCAAAGGTGACATAGAAC
TGTGCGGAAGAGGAGTAATCACCTATGAGGACATGCTTTTCCCAATTCAC
CTTACACACATTGACATTTGGAGCTGAATGAAATGATGCCTGATTCAACCT
GAATGGATAACCAAAAGTAGCTGTAATCGTCTTATTTTCACCAGGTGCAG
AAAGACAATTCACTTGAATTTCTGTTTTGCCCTTAAACCCACCACAGGTGG
CAAAAGCAAAGATAGAAGCAAACCACTCGAGGATCTTGATGAAGCCGAG
CGGCTCCTTGAGCGGGTTGAGGTTGATCTGGAAGGTGGACATCCTCTGAG
GGAAAGGAGGGAAAAGAGTCAGGACGCGGGGCGGAGGAGGGGAGCAGC
GACACCCGAGCACTTCGGCCGAGAGAGCGGGAAGGCGGGGCCGGCGAGC
CTAGGGGCGGGGCTGAGGGCGGGGTCTGCCGGGTTGGGAGGAAGGCCCT
TCGGGCCGGGGCGGCCTAGCCTTTGGGGCGGAGCGGGGCGGCGGTTGAG
GCCGCACTGAAGAGACGGAGAGGGTGAGCTGAGGCCTGGCTGAGACCGG
GCTGGGAAAGGCTGGCTCCTCCCCGGGCCGCACCTGACTCAGCCGGCTGA
ACCGCTGGCGAATCAAGTAGGCTTTGGAAGCCATGCGTGTGGCGCCCCTT
TCCCCAAAGCCTATCTCTTTCTCTACAGCGCACGCGCGCTCCCTAGGGCAC
CCCCCCAGTCTGGCTTCCGGCCGTCGCGAGCGCGCGGGGGTGGGGGTGGG
GTCGGAGGGACCTCCCCCCCTGCGCCTGCGCAGCGCGGACCTCCACCTGG
GCGGCGTTCTGGTTGCTATGCCCTTTTGTGGCTGTTACAACCGGCCCCCAC
AACGCCCTTCTATCACCTTCTTCACCGTGCTCTGAACTTTCTGGACAAATG
TATGAAGTATGAACCCAGACAAAGCCTCACTTCAAAGGTCGTGTTAAAGA
GCCTTTAAAGTAATAGTTATTGCAAAACTACAGCGGTCTGGCGAACTTGC
CTTATGTGAGGGGGTACTTTGAAAGTATGATTCACCAAACATGTCCGCGG
ACATTTCAAAGTTGCTGGGAGTCAGAGTAGGAGAAAGACCACCCATTTTC
ATGACTTGACAGGTCTCACAGGGCAACTCATCTCTCCACTCCTATCTCCGT
CTGGCGAGGCCTGTATTTCGGGCGTTTTTTGGGGGGGGTCGAGTTAACATC
TTTCATGATTTGTCTTAGGAAACCCTCCCTCCTGCTACCAGAATGGGGGTG
GGGACAATCAGACATCTCTTCTCTGACTTCCTTGTTACCTTGCATATTTTGG

AGGGCTGGGATAGCGGCCTGTCTTCATGCTTGGTGAGATGATCAACCCAA
CAGCGAAGGAAGGAAGATCTTTGCCACAGAGGATCCCATTTCCAGCTTCC
AGGAACTTATTTGTGTTTATGAAATGTCATTGGATTATTCTATCAGAACAA
CTACAACTAAAAGTAGTGACC
>LNC_009611
CAGGTTGAAAATAGACTCTTTTCTTTCTTTCTTTCTTTCTTCCTTTCTTTCCT
CCCTCCCTCCCTTCCTTCCTTCTTTCTTTCCTTCTTTTCTTTTTTCCTTGTTTA
TTTCCAGTATCCAGCTAGGCTAATAATGTCAGGCCTAGCATTTTATTGTAT
TACAAAGATAATTGCTGCCTGCCTTGGGTCTGAGAATTCTTCCTCTTTCAG
TGGTTTTCTCTGACTCTTAGCAAATGTGAA
>LNC_009779
GGAGAGGAAACCACTGGAGAGCTCAGGCTGGCAAGAATGTCTCCAGAAC
TGACTATGGGGTGGTGAATTGGCAAGAGAGTGATCTTGGAGATCAGGAGA
GGTAGAGCTGGCATTTGTGAGTGAGGCGTTGGCTAGGAAAAACACACAGT
CATGGATCTCAGACTTTGTACCAGGATAGGAACAGACACCGCCAATGCCA
ATACATAGCTGACTTTAGCATGGGTTCATTTTTAAAGCTACATGGCTACAA
TAACTCATAACATCCAAAGGGTGTGAATAGTCCAATGGACATAGTAAGGT
TTTTCAAAATTTGGCTTAACATGTGTACCTCAGCTATACTATAAAGAGTGT
GTAAAAAACAAATCTAGATATAATACCAGTAAGGAAAATATATATTATTT
ATTTGTTTTGATGAGCATTCATTTTTTTCTATGTCTTTTACATGTTTTGGTAT
GTCTTGACAGAAATTCATATTAAAAAATGTTCATACAACAACTTTTGGTCT
CAATTTCTCTTCTAGTTTTCTTTATGTGCTATGAAAAACATTAGAACTAAA
AGCAACTTGGGAAAGAAAGAGTATATTTAGCTTACACATTTAAGGCAAAC
CCCATCATTTAGGGAAAGGTTGAAGTGGGCTCTTAAAACAGTAACAATGG
AGGCAGGCTGCTTGGGGACTCAATCTGGTTTGCTCAGGCTCATACTTAACA
ACATTATAATACATCATGAGTGAACCTTCAGAGGGATTGGTGTCACCAAT
AATAGACTGAGTCTTTCTAAATCAATTAGCAAAGCAGTCTCTTACAGACA
GGATGTTGAAGACAGATTATTTCATTGAGATGTCTTTTTTGGATGAATCTA
AGTCATGTCAAGTTGACAAGGCTAGGGAGGTCATATACTTCCTCTATGTCC
ACGAATGTACACTCATCTGTTTTATAAATGGTAGGATTTTTCAGGAATACA
TGTTTAATAAATTTAGAAGAAAACTATGAATAACTTTATAGAACTGTTAAC
CTACTTAGAAAATCTCATCATTAGCAATTTTCTGAAGAACCAACACAAGA
CAAGGGAATTATGGCATTTGAACACACACACACACACACACACACACACA
CACACATGACTTGATAATAAAGAGACAAAGTTCTGATTTTTCTCTAAGAA
GACACCACTGGATTATATAGAGCTGATTATTATATTTTATAAGCACAGTCA
AATAAATTGGTTTTCCTGAAACTCGTAAAGTTACAGTCTGAACTTACTTAT
CAAATAGTTTACATACATGCTAGTTGCGTATCTGTGAGAATTGGCTACTGA
AAAATGTCTAGGTTTCTTTTGGAGATCCTATCTGACATCAGTGTTGTTAGT
GAGCTAATGATCATTAAGCCAATTTCAGCAATTATAATATTTGAGATTTCA
GAGTACTCAGGATATGTACCAGAGACAAAAATAAGTTTAATTTATTTAAT
ATTTGTATGAAACAAGTTTTAAAAAGTTTTATTTTTTTTACATTTATGGATA
CATATATATGGATATGGACAGATGAGAGCCGTTGCTAAAAAAGTTTAAAA
GTAGGCATTGGATTTTTCACAGTTGAAGTTACAAACTGTTGCTATCCACTA
AGTGTGTTTGCTACAAACTGAACTAGAGCCCAGTGAAAAGCTGCAAGCAC

TGTCAACTACCAAGTGATCTCTCCACTTCTTATATAGTATACTTTCAATTAT
GACCTGAGGCATTATTAAATCTTTCACTGAAAATAGCATGAGCATCTTTCT
TACCAGAGGAATCTTTGCTTAAATTTGTTTTTGACAAACTGTGTAAGAAAA
GCTATTAACGTTTACACAGTCCCCAAAATAGCCGAAAAGAAACAACACAA
GAAAGAGCTTAAACACCACGCATGTGCGCACACGCAAACACAGACACAC
CCTGTTTCCCTCCCTTATACATTCAACCAATGTTGTGTTGAGACCATTGAG
TGCAATCATTGATTGTAAAGTTTAAATTCACAGAGGATGGAAGGAAGATG
GAGGAAGACAAAGATGATCCAGATTCTGTGTGGCATTAAATAGCCCACAG
GTAGCTATGAATATCTTATAAGGGATGGATAATTACAGGATAATTTGTCTT
ATCTAGTTCCAGAGGGAGAAGACTTCATCATGGCTAGGAAGTATCAAAGC
AGG
>LNC_009798
CAAAAGTCAGGATGTGTAAACCAAAGAATCAATAACATGACTAGCTAACT
TGGCCAGATGCTCTTGTCAGCTACTGGTCATTTGTTTCCTTTCTGCTTTAA
CCATATAACATGGAGAAGTAAATTATCAGGTCAAGTTCTTGGGTATTCTTT
CATCCTTGAAATCATTATGGTTGTCTTGCCCCAGGCATCAGCATTCAGGGC
TTTTCTGCTTTGGGGAAAGGTCTCAGTTCACCTTGCTAATATGAAAAAGAC
TAACCCATCAGACCTGGTTATGAGATCATGTCCTGAGCATCACCATTAGGT
CTTTGTCGCACAGCCAGGGGAGGCAGCGGCTTCCCATAGAAATCATTATG
ACGTGTTTCACCAAGAGGCTCAAGTTTTGGAAGTCTAGTGACTTTGGGGGT
TCCCCAGCCAACAGGAGGGGGAGGTGGGGGCGGCGTGGGACTCTTAGGA
GCAGACTCGTCTGTATTGACTGGTTCTACCCGATGACTCCTTTTCATTCTTA
ATTTCTTGGTTTTCTTCTTACTGTTATCACCTGGTTCTGACACTCGTTGGTC
TAGCTCATCCTCGTTGTCCAGTTGCTGTGACAGTGCCTCCTTGTAACTATTT
ACCACATCCCTGTGAGGGTTCCTGAGGCAGCCACCTGCCTCATTCTGCGAC
TCTGCTCCCTCCTGTTCCCCTTTATGTTCTGGGTGGCCAACATCACTGTCCT
TCTCCGCAGTCTTCTTCTTTTTCTCTTTTTCCTGTTTCTTTTCATTCTCAATG
ATGACAGCTGCTATGGGTTGGAAAGGATTTGTAAGAACTGGTCCTGAGTC
CATCTCTGCCAGACCACTGGTCC
>LNC_009828
AGTGCCCCCAGTCCCACCCATCCGGGAGGGAGCAAGCCAAGTGTGGGCAA
AGGGACCCATGTCCCGAGTGGCCTGCCATCCAGGGCAGGTGGCTTGAAAA
GGCAGCACATGGTTCAGTACACCACCTCATACACAGTGGCCTTCTTGTCCC
CTGAGCCTGTCACAATGTACTTGTTGTTCCTGGAGATGTCGCAGCTCAACA
CGGAAGAAGACTCTTTGGACTGGAAAATGCTGGCCCCATAGGGTGTCCTC
CAGGCATTGAGCAGGTTATCCTTGCCTGTGCTCACAAACCAGCGTCCGCA
GGAAGCGAACTTGAGCGACAGCACGCAGCTCTCGTGCAGGCGGAGCTGGT
ACTTCTCAGGCTTGCGCACATGCAGGACCTCCACGTGGCTGCTTTCCATCC
CCACGGCCAGCCAGTCCTGACTGGGACAGTGGCCCAACGAGAAGATCTGG
GAACTGAAGTCATGTTGCTGCAGCTGGCGGCCCTCGCGCAGATCCCAGCA
GCGCACGGTGTTGTCCAGGCCCCCAGTCCACAGCCGGGTCCCGTAGTCTG
ATATGTCAATGCAGCTGGCCCCGTCCGTGTGGCCCTGGAACTGTCTGACCA
TGGCCTGGTTCTGCAGGTCCCAGACCACGATGTTACCGTCGCTGCAGCAG
GAGAAGCAGACCTTGGCATCCGGGCTGATGGCCAGCGCGTAACAGGCGG

GGGCGGACGAGGTCAGCTCTGCTTTGATGCGTGGTGTGGGTGCTGCCAGG
TCCCAAATGGACAGGGTACTGGCCTCGCCACCCACGATCAGGCTCTGCCC
GTCAGGCAGCAGCTTGCAGGAGCGAATGTAGTTGTCCCGGTTCTTCCGCA
GCAAGAAGCAGGGACAGGAGGCATTGGTACTCC
>LNC_009848
GGCTGAAAACGAGTCTTTATTGTCCCCACTACTTGAGGGGGATAAATCGG
GAGGAGTGGGTGGCACCAATGCCGGGCCGGCCGTGCTTCACAGGCTTGTA
GGTGATGGAGAACTCGCCCAGGTAGTGGCCGATCATCTCGGGTTTGATCT
CCACCTGGTTGAAGGTCTTGCCGTTGTACACACCCACCATGCTGCCGACCA
TCTCGGGCAGAATGATCATGTCCCTAAGGTGGGTCTTCACGACCTCCGGCT
TCTCCATGGGTGGCGCCTCCTTCTTGGCCTTCCTCAAGCGCTTGAGCAGTG
AGTGCTGCTTCCTCCGCAGGCCTCGGTTCAGGCGCCGTCTCTGCCGGGCGC
TGTACAACTGCATCAGCTGCTCATAGGACATGTCCAGCAGCTGGTCGAGG
TCCACGCCACGGTAGGTGAACTTGCGGAAGGTTCGCTTTTTCTTCTGCTCC
ACTTCGGCCTGCGTGAGCGCGAGTCGGGGTTAGCGGGAGCTCGCCGTTCT
TTCCCGGGAGCCCCGGAGGCGGACTGCGCGAGCTGCACAGCTGCCCGTTA
AGGCTACCCGCTCCCGGCTACGAAGGCCCGACGAGGCGATACACAGCCCC
GCGGCCGCCCGCAGCCTTCCAGGCCCTTCCGCACCCGCCATCCGCAGTCTC
CGAGGACCCGAGCACATCGCTCCATCCCCGAGCAGGGACTCCCGAGTCCT
GCGAAAGGGCCCCGGGAGGATGGCGACGGATACACTGGCGCAGAGAGCG
AAGAAGACACATACTCACCATCTTGGCGGCTGCTGGGAAAAGGAAGCGGT
GTCAGCGGCTGCGCAGTTATCGCGAGCTCTGCTGGGAGGCCCCGCCTCCC
GCCGCACAGGAGCTTCCGCCCTCGGAGACTCGCTATTGCCCCCTGCTGTCC
GGAGGGCATAGCGACGAGCGCAGCTAACCTCGGAGGGGTGTGGCTCCCTT
TGGGGTTTTTGTGTCTGTCTTTCAGTCCCTATTTCTCCTTCGCTCGGTCCTTT
CGGATGTGTGTCTCTTTCTCCCGTTTCTGTGTGTTTTTCCTTGTCTTCTTTGT
CTTTGTGTTTCTGTCTCATGTACAAGGTCTCAAGCAGCGATTCTCAATCCG
TGGGTCACAACCTCTTTGGAGGCGCCATAACAGATATTTTACGGTTCCTAA
TAGTAGCAAAATTACGGTTATGAAGTAGCCACGAAATCTATACATACATT
TTTTGGGAGGGGGGCGCAGACGGGTTTCTCTGTGTAGCCCTGGCTGTCCTG
GAACTCACTCTGTAGACCAGGCTGTCCAGGAAATCAGAGACCCTCCTGTC
TCTGCCTCCTGGACGCTGGGATCAAAGGCAAGGCCACCACCATCTGGATG
ATTTTGTATTTTTATTTCAAAATAGTTTTGAGTCAGGGTCTTACTAAGCACC
CAGCCTGTGAAACCAGGCTGTCCTCTAACTCAGAGAAAGATCTGCCTGTC
ACTGCCTCCTGAGTGTCCACCACGCCCGGCTTATCTTGTCTGCTTTACTTGT
GGGATGTGTGAGCCTGCCACCTCAAAAGCGTGGGGGTTAGAGGACAACGT
GGAGGGATCAGTCCTCTCTCTTCAGCAAGGTCTTCAAGCATGGCAGCAAA
ATCCCTGTCCCAGTGAG
>LNC_009963
CAGCTTGCAAGAGGTAACGTCGCTTAACAGAACAAGCTTGCAGAGTCCCT
TGTGCCAATCCGTGAGGCGCAGGCTGCTGCGTGACCAGGCTCAGAACTGT
GTGTACAGAGGAAAGAGGAGGACCCTAGCACGGCGGGGCACGAAGACAG
CTCTCCGAGAAGAGAGGCAATCAATGCGGCTCTCTCATGCGTCCCGAGCT
GTGCCTTCCTGGCAGGAGGAGCCTTAGCCTGGCTTCCACACCTCGCCCAG

GGCTTCCTCCAGCTTCTGCCTGTAGGCTGTATAGCGCCGCTTCAGGCTCTG
TAGCTGTTCGTCCTCTTCCTTGTCCAAGATGCGCAAGAAGTTCTGCAGCTC
CGGGAGGCTGAAGGCTTCCCACTCTCCAATTTCGTGTTCTCGAAGAACAA
AACTAAGCGTGTCGGTTCTGGGCCCTGCTACCAAACGCAGGTACAGCGGG
TGTTCCCGGTCGGAGAGCTTGCAGGCATACACTTGGTCTTCCCTGTGACAA
CGCTTATAAAGCGCAAACTTGGTGGGACTCTCGGTCACCTGAAACTTCTTG
AGCAGCGCGTCGATCACGTCTCCCACGGTGTTTGTGCTGCTGATATGAAGC
GTGTTCATGCAGCCACTGCTGCTGGGGTTGGCAGGGGTCTGGGCAGGTTT
GCAGAGTTCCATCTGCACCTTAATGAAGCCAGTGTAAATCCCGTTTGAATT
TAAGGTCATCTTCAGCTTATCTGTGACGGCTAAATTGTACTTATGCACTTT
CTCTTTGATTTCCTCTTTGCTGAGGTAATTGTGTGTTTCCTTCTCCTTCTCCA
CATCCGGCTGACCCGAACGGGACTTGCCCGGAGGCGCTCTCCTGAAGAAG
GAGGTCCTGGCGGTGAAGAAAAAATCCTCCGCGTCCTCCTCGAGGCTGCT
GTAGCCGCTGCTCATGCTGCTGGTCCGGTTGCGCTCAGGGGGCCTCCCCGG
GGCGCAGGCGGCCAGGCCGGGGTGCGGCGCCCGACCGCCCCGTGAGGTTC
CCGGCGCGCGGCCCCGAGCCGCCGGCCGGCCGTCCAGCTGCAGCTGCGGC
CGCCGCTCAAGCCGCCCTCCAGTCCCCTCCCG
>LNC_010063
TGCGGAGTCTCAGTCTTTAATCTCAGCAGTGCTCACACACATGAAACCAC
ACACTCTCGGACTTTCAGATCTTGTTGAAGGCTGCAATGTCGACACTCTGC
ACATGCTCCTCAAACTTGGTGATCTCCTCTTCGAGCAAGTCGGTGCCCACT
TTGTCATCCTCTACCACACACTGGATCTGCAGCTTCCGGATACCATAGCCG
ACAGGCACGAGCTTGGAGGCCCCCCAAACCAGTCCGTCCAATTGGATGGA
ACGCACACAAGTCTCTAGCTGGGCCATGTCTGTCTCATCATCCCAAGGTTT
AACATCCAAGAGGATGGAGGATTTGGCCACCAGTGTGGGCTTCTTGGCCT
TCTTCTCTGCGTACTGGCGTAGCCTCTCTTCCCGTAGTCGGGCAGCCTCCTT
ATCTTCCTCTTCCTCATCACTGCCGAACAGGTCAATGTCATTGTCCTCGTC
GTCCTCTGCTGGTGTGGCTCCTTTCTTGGCTGGGGGCTCCACTTGACGCAT
AGGAGAGACATGTTGGGTCTGTGGGGCTGTAGCTCGGTGAGTAGGTGAAC
TCTTCTCCAGAGTGCTCAGCCGGACCTCCAACTTGGAAATGGCCTGCTGCA
AATCTTGCACCACGCCTCGAAGGTTCTGGTTCTCTACTTCCAGACTGGCAA
TCCGCACAATGAGGTCACTGTGGTCTCCACCAGGTCCACTGGAGGCTCCA
GGGCCTGAACTCTGCCGAGAGCCAGCGGTCACAGGCCCATTCATCTGCTC
GTAGAATCTCCTTTCTGCATCGTCATATTTAAACTTGTCAAACCAGATTTT
CTCGTGCATGAGAAAGTTTGTAGCCATTTTTCTGCTCAAGGACCGCACTTC
ACCGAGGAGGTATCGCTAGCGGCCAAGAACCGGATCTCCCGACACGCCGC
ACCCCTCCACAGCCCGAAAAGTGGA
>LNC_010192
TTCTGTCTGTAAATCCACACATACAGTTCTTTCCACGTCTTATATTTCAAAA
CAAATAATGCTGGTTGTGCTTCTCATTTCATTCAATGGTACTACTCTCTAG
ATCAGAGGAGCCCCCTTACAATTCTCCACAGGTAGTGATGACTGGGGTGT
TCAGTGCGACTAAGCAGGAGTTATATGGTTGCTGCCATGATGCTTGATCTT
ACCCAAGGTAGGGTTTCGGGGCATGGTTGCCAAGATGGCTAACAACAGAC
AGTGATTGAAGTGATGGAACAATAACCTACAATTCTCCCTCTCTGTCACTG

TTTCTCTGTCACTCTCTGTCTCTCTTCCCTCCTTCCCTCCCTCCTCTCTCAGA
CCTGAATGAACATTGGAACTAAAGTAAATGTACAGTGAAAAGAAGCCAG
GTGTGGTGCTGCATGTCTTTTATTGCATCACTTGAGTGGTGGAGGTGGGTA
GAACCCTGTGAGCAGGCTGCAGACCTGATCCAAGTACTGATTTCCAGGGC
TTATGTGCAAAGACCCCTGACTCAACTAAAGAAATGAAGAGGAAAATAA
AGAAACAGATACAATTAACT
>LNC_010205
TAGAAAAGGAATAAGAGAGATACATCTCTTGAAATAGTATAGAGAATTTG
ATACTTTATCACATGAGAATTAAAACTGAGCCACATAGCCTTCAGCAGAA
GAAATGAGGGAGAAGTGGGTATATACCTTTTGCAGCACAGTCTCTGGAAT
AAAACAAACAGGGACATTTAAATAAATATATAGCAGTGTTTAAGACTTCA
TTTCTCTTCCAATATTTGCAATTACATAGTCAAACATTATATCTTGGAGTC
ATAGATCACTGTGCTATCTAGGATTGCACGATGAGGATGGCATAGTGGCA
GTATAGCAAAATAACTGATAGGAAAAGCAGAGCAGTCACCCCATTAGGAT
TCATATGAATTGCATTTCCTCCCTGCTGGGAACTAAGCAAAAATGGGTGA
AACTGTACAAGTACCTCTGTAGTAATTTGGTGTCTAGGGGTTATGTCCAGA
GGATATACAGCTGGAGCACATGGTGTTCAATTTATTTTTAGTTTGTCTGAT
TTAAAATGTGATTGCTGTTAGTAAACATTTTTGTGAAGATTGCTTGAAATT
TATTTGCTCTTTATTAGAAATTGTAGAATCATATTTATAATTTGCAGTCCTG
GAGGTACTGGTGGTTGTACATGGGGAACAACTAAGTCAGCAAATGATTAA
GTCATACAATGCTGATGAAAAGAACATGGGATACCACAGTTACAGCTGCC
TAAGAACTGATAACCACAACCTAAGGGAAGAGTCTCTTCCCTCAACCTTG
ACTCCTTTAAGACATTGGCTTCAGCATCAGACAGGCCTTGACAAGTGTGAT
CCTGGGTAAGGGCATAGAACTAGTGTTCCAGTTATTCTTTTGTCCAGGTCT
TTCAGACAGCCTAGGGCAGTCCTGGCTCCGCACATTAATATGTACTTCATA
GTATTCATAAGACTTTCCAGCTGGATGTGACTTTTGAATTTTCTTTCATAA
GCAGGGAAGTGTTAGATCACAGTCCTGTCTAACTGGCTGCTTTCCTCAAAC
CTGCCATCCCCTTTGTAACCTCAATATAATTGATAAGCAACATATTCTGTG
GCCTTGTAG
>LNC_010277
AAACACATGTGAAAGTCAGAGGAAAAGGTCATGTGTAGGTTCTCACCCCT
TACCTTGTCCTGGGTAACCTCTATTGCTACTACCACTGAGTAGTCAAGGCT
ACCTTGATCAGAAGCATCCAAGACTTATCTTCTTTTCTCTTTCCACCTTACC
ATGTGACCTGTGGTATCTACATCAACCATGACCATACTTATTTCATATGGA
TTAGTTTTTGATATGGTGGCTGTTTGCGGCAAGGTCTTTTCCTTAATGAAT
AAAGATTTCAGGGCACCAATCACTGGACATATACTTCCAGGTTAGACTTC
CAGGTTGGAGGGAGAAAGAGAGGATGAGAAAAGGGACCTGTGGATTTTT
GGATTGTGGGAGAACTTG
>LNC_010282
CCTCCAAAGCGTTTTGCCACTTCTCTAGCTCTGTCCTCTGTAGCACTCTAA
GCTCTTATTGACTCTAATTCCCTGATGCTTTTCTTGGTGATCATCCCATGGT
TCTGGAATCTCCAATATGCTGGGGTCTTCACCTACAATCAGGTTTCACCAG
TAGTCTCTCATAGACTCTCTTCATGATGCTAAGCCTCAACTTCTTTTCATGG
TCCCTTCATTCCTGAGACCTCAACTGTTACTAAGGCTGTTCCTTCAACAGT

GGCCATCCCTACACTCTCACAGTGCCAAGCCTCATGTGATCTCATGACCCC
TTCATACCTTTAAAGCCAATACTACCTTGGTGATTCTTACACATTACAAGT
CCAGCTGCAGCAGGAGATACAACCTTGAACATCTCTGGAACACAGCTTCT
TTGTACTCCAATTAGATGGAAACAGCATGGAAATCTCGGGCAAGTTTCTTC
ACAAGGGAGTGTATGCTTTGAATCAGCATCCTAGATATGGTGCAGTTTCTC
CCACAGACAGGATCTATGGGTCCAGGAATCAAGAGGTGGAAAAGCGCAT
TCACTACCACTCCTAGTAGCCCACTAAATGTTGTCTCTGCAACCCTAAATT
CTGCTGGGCAAGAAGTTTGGAAAGAATGCTAGCAAGTAACTGCAAGCAGT
TGCATAGAAAAAGGAAACTTTCCTTCTTTCACTGACCTTATGTATGCCTCC
AGCAGAAAATATTTCCCAGATTAAAGTGTGTACCACAACATCTGGATCTA
TGGTGTTCTTTACTTGAAACTTGATCTATCTCAGGCTGACCTTGATCAGAG
ATCTGATTTCCTCTCTCTCCTGGGATTAAAGGCATGTACCACCTTTCTCTGA
CTTTTCTAAGCTTTTCATGGGGACTATGTTTCAAGATTCATATCAAAAGCA
TGTGTTTTTCAGCCTCAAGATCTGAAAATCCACTATAAGATATGGGCAATA
TATCTTGATATTGACAGACATATCTTCAGCAGAATATATTTTTGAAAAACT
TTAAGCACTAAAATTTCAATAACTGGAAACCGATTACACATTAAGTCAAA
AATAGGTTATCATTGTGTTATTGTCATCTGCAATACTCCCTATCAGTGTTTT
TATACAACAAACTTATATTCAATACACTTCCAGACTAGAATGTAATCATTT
TTGCTTGATTGACTGCTGTATTTTCTATGATGCGAAACTTGAATGTAACAA
CATGTGAGTGCCAGGGAGCACTGGTAACTATATGTGGATTCCTAGACATT
CTTTATTTGTCATTAGATATATTTGATTCTGAAATTTTAAAATACATTCCAG
TTGCATTAGCTTTATTATATATGACTTTTATTTCATCCTACTTCAAAT
>LNC_010302
GTGCCAGCCAGAGGAAGAACTTATCTCTCAAAAAGAACCACACAGTTCTG
ACTCATCAGTCTGCTTTGCAGGAGAGACTGGAGCCCAGAAGGTCCTCTAA
GACCCTCCCTGAGTGCTAAGTTCTTAGCCAGTCCTACAGAATTCTGCCAGA
ACTGCACGTGATCTTTGGGGAGGTGTTAGAAGATATAGGTGGCACAGGAT
GCCTTGAGGGGGATTCCATCCTCACAGCCCCGAGGAAG
>LNC_010338
TTGCAGCAAGACTAGTCACCTCCCCTCATATTAAGGCTGGATGAGGCAGC
CCAGTAGAAGAAAAGGGTCCCAAAAGCAGGCAAAAGAGTTAGAGACAAC
CCCACTCCTGATGTTAGAAGTCCCACAAGAAGACCAAGCTACATAACCAT
AGCATATCATACAGAGGGCCTAGGTCAGATCCATGCAGACAAGTTTAAAT
TCTGTAGTAGCTACATCCAAAAAAGATTACCTTTAATCATGAAACTTACTT
TATACAATATTCCTCTATATTATGAAATGGAGATTTAATTTTGATATTTTGT
AATGTTTAATACAAGGTTAGCAATATGATGAATAACAGCACATACCTCGA
CTTAGAGTGTTAACATTTGAAAATTCTTAGTAACTCCATAGGGCTGTTAGA
AACCATACTGGAGAGTCAGATATGGGATTTTTGAAAGAAATCTGTACTTA
GGTTTTAAAAATGGAATCAAATTGAGTTTCCGAGCTTCTTACTATTTATTT
ATTTATTTATATGGTATTATAATATCAGAAGAATTCCTACATTTAATGGAA
AGGCCTTTGACTTTGTTACTGCAGTGGGATGAGATAAAGGACATACTAGG
AGAATATTACATTTTGATTGACAGTTGCAGAAGTTTCAGCCTTGGTTGGTT
GGTTTCCTTGCTTCTGGGACTATGGGAAATCAGAACACTGTGGCATGGGCT
GTGGCAGAGGAAACTGCTCACTTAATGACATGGGGTGGGGTGGGGGACA

AATAGAGTGAGAAGGAAGAGTGGAGTTCACCTAGAGTAGTGCTTCTCAAC
CTGTGGGTCACGACAATTTTATGGGTCAACTGACCCTATCACAGGGGTTGT
AGATCAGCTATCCTGCGTTTCAGATATTTACACTAAAATTCATAACCGTAG
TAAAATTAGTTACAAAGTAGCAATGAGAATAATTTTATAACTGGGAGTCA
CCGCAATGTGAGGAGCTGTATTAAAGGGTGGCAGCATTAGGAAGGTTGAA
AACCAGTGATCTTGAGCCTACTCTTTACTGCTGTAACTTCCTCCCTACTTCC
TGATGTTTCTCCATCTTCCAACAGTCCTAGATCACAGACACTGAGCTTAAC
TACCACCTTCAGATCTTAGCTTCATATCAGAATAAGCCAGGCACTGTTAAA
TGTATTTATTTATTTATTTATTTATTTATTTATTTATTTATTTATTTACGTGT
CTATGTGCATGTGCATGGACATGTGCACGACACAGCATGTTTGACATTCCA
TGCAGGCTCACAGACTCAACTCTGGTCGTAGGACACTGAGCTGCAAGCAC
CCTTCTTCACTGAGAGCTTCCCGGTGGCTGCAAAGGTTTGCCCCCTTTATT
TCTCAATTTAAATTAAAG
>LNC_010342
CTGGAGTAAAATAACAAAATCGAGTGAAAATTTTCCTCAAAACCAAATCT
TTTGGGTATGTTTACAAAGACCAGTCAATGGCAACAAACATTGATGAATA
ACAGTCTCATTACAAAAATCTGTACAATTTAAAACAACAGGTATTCCTAA
AACATCTGGGCAAAGTGTGCCTGGGGCTGGTTGGGCACAATGAATGCACA
GGCATCCTCCACCTATGTATTTACAAAGTGCACGTCCATGAGCAGAAGAA
AACGGGTCAGGATTTTCTGAGTTTGACTCAGCTAAAGTCTTTGCATCTCAA
AATCCTTTACGAAAGGAAACAGTAGAAAGTCCGAGTCTTTCCAATGTTAC
TTTTTTCTCTTCTGCTTCTTTTGGTCAACCTCCATCTTGAGCTTAACTTCCTC
TGCTGTAAGAGCGCCCAGCTTCTTGGCCTTCGCCTTCTTAACCTTTTCCTTG
ATGGCAGCCACATCAATCTTCGTTTCAGTAGAAGCTTCCTTTGGCTTCACA
GCCGGCTTTTCCTTAGGTGGAATAAATGCTTTGTTTCGTTCTTCCTGCCTCT
TTGTAAGAGCTTCTGCTTGTTTAGCCTTGATTGCTTCCATTTTCTGCCGCTT
CTTCTGATTCGCCTTCAAAAAGTATTCCCCACTAGCCAGTTCTTTGTCAAC
CTTGGTTCTCACGCTTCTGTTTCTGCTGCCGAGAGTCACTTTTTCGAGCTTC
TTTGGCAGGCGTGTCTACAGAAG
>LNC_010359
GTGCAGCAGTGAACAGGGTCCGTTGAAGGGCACTTTCTAAGTGAGCGGCT
GAGATGACAGGTCCAGGGCAGAGGAGCTTCTACAACAGGTGATGCTAATA
AAAGAATTTACAAAGTCCATTGAGCGGAAAGTGCTGGTGAGCTAAAGAAC
CCCAGGACACCCATTTGTCCCGGTGGTGATCAGCTGATACTCCTTGCTTTT
TCTTGAGGGTTCTGGGCTGCCATCTTTCATTAGAAAAAAATAAGATAAAG
ACGCTCCTAGGTTTGAGAACACTTCCCACAGACAGAGGTGTAACTTCCAT
AGCCACTCCTACTATTTTGATACAAAACCCTGGCGCTGGCAAGTCGAAAG
GTGTTTGTGACAGTACCGCTTGCTGAACTCAGACTCTAACGTGTACCCCAC
CTTGAAACTGTGTCTGCAGGGCAGTTTTAGAACTGGGATGCGTAAGGTGC
GTCTGCACTCCATGTTGGTTTTAATGGCTCTCCCTTTTGAAGAGCGGCGGC
ACACAGGGACTGGGGCATCAAAGGAGATGTCTAAACTGTGAGTCCAGAG
AACGTGGGCGGCCTGCTGCAGTCTGTTACAAGCTGAGGTCTACTTGGATG
TTTGCAGGGACGCTCCTTAGCTGGGAGGTACAGAGGATCTCGTGTCCCAC
CACCCACTGTGTCGCCTGCTTTGCGTGAAGGCATGGAGGTAGGAAGACCT

ACTGCAGCTGATGGCTGTCGTCACTCTGACGTCACCTGCTCTCAGGAGCAG
CCTTCAGACTGAAGACCAGGACGTACGGTCCGAGGCCTGGCAGGAGCTGC
TCACTGTTACTTGGGAGCAACTGAGGCCAACTTCACTATTTCACTGATGAC
GTTTAGAATCCGAGTGGAGTGAATGATGCCACTTAGCCACAATTTTGTAA
CTTTTGGTTTAAAACTTAAAATTTTTTTCCCTCTAAAAGTAGCCACAGCAG
AGGTGTCCATTCCATGCCTGCTGGCCTCTCTGTATCACCAAAGTTGCTGGG
GTCACATGAGGTAAAGGAATAAACTCAGTGGCTGTCACCCCAACATGGCC
TCTGGAATTCTGAAAGTGACTTCTAGCTCTGTCTTAAAAGTTAAAGCTGGC
AGAGGCTGTGTAGCACTAAACTCACTCACGGCCTAGACAGGCTGGAACCA
GGCCCTCTACTCTGCTGTCCTGCCAGCACTTAGCCCTCTGTGGAACTGGAC
TTTAAGGAATAGAATGTCTGTGAGAGCAGGGGAGGTGGGATGAGGCAGC
CGGTCCTTCCCACCACCAGGAAACACTAATATTTTCAATGACAATGTCAGC
TAATTGCAGCCAAGCAGGATGACTTGTGTGTGGCCTTGATACTCGAGAAC
CGTCCAGACAGGCACTTCTAAGTGCAGCCCTTCAGCCCACTGATCCAAGA
GCCCGATCTGCTAGCTCATCTCCCCCTGGCGGTTCCTTCTCCATAGTGCTTC
TGGGCTCCCTGTGGTTAGAGCCACTGAAAAGCTGAGGGCATCTTAGGGCA
GAGCTTGGGTGCCAAGTCGCACATTTACCACACCACAATGGATGTTGTGA
CCGTACTTTCTCTCCAGCTCTTCTCAGGGCTGTGTGTGTTTGATGGTGAGG
AGTTCAGTGTGAAATGCTAGAGAGCGCTTTTGATTTGGGGGCTAGCTACA
TACCGAAAGGCTTTATCTCTCTGCCCTAAAGTATTGCCGTTTATCAGAAGA
GTTTTACAGATTCAGACTTGACCACATGGGAACTCTTATACTGTGCCCAGC
CAAGATGAAGGGACAAGGAGAGGACAAAGACCTCAGGAGTGGTGGTTGT
CATTTGAGGAGAGACAGGGTAAGCCACTTCTGACGGGGGCAGATTCACAG
TACGGAAGTCAGGTGACTGGGTTATTTCAAACAAGACTTGAAACAGCAA
GGCCAGAGCTCCCCCAGGCCTGGGCTCTTACTCCGACTACTTCATCGGAA
AAGGGTGTGCAAGGATCCAACTGCAGAGGCTGGGCACAGCTGCTACAGA
GGATGCTTTGAGAACCTTCTCATTCTGATGCTGCCATGATTGCTGAACATG
AGGCGCAGGTCAAACCTGTATCCAGGGTCAAAGACCCCCGGAGTGCGACA
GGTTGGCCCGGAACAGGACTGGAGCATCATCAGCCGATAGTTCATCTTTC
CCAAAAGCTCGGGGTCTATACTCTTAGCAATGTTGGTGATCTGGTCTGGAT
CTGCAGTTATGTTATAGACTTCTACAAATACCTCCTGGTCGTCGAACTCAC
AGTACTGTAGGTTCCACATGGATGACAGTGTCCTCACACAAGCATATGTA
TTGTTGTACGCGTCCTCACACACACAGTCCGGGAAACATTGAGACACTCC
AGGACTCAAAGAAGGGCAGGTCGGGTCGGTGACATTACGGCCTTCTCCTT
GATATTCCACCAGGACATCTGACCTCCAGGTTAAGTTACCGTCCCCTTTCA
GGATGGGCAGCAGGGATGTCCCGTCCATCTGTGTCTTGTTCAGGTCATAGC
CAGCGAGGTCCAGGATGGTGGGACCCAAGTCAATATTGGAGACCAGCATC
TTGCTTGTCTGGTTTGGTTTGATCCCAGGCCCTCGAACCAACAGTGGAACT
TTGATATCAAACTCATAGAGTTGTCGTTTGTCTATTGGCAAAGAGAACTGC
CCTGTATGGTAGCCATTGTCTGAGGTATAGAAGATGTACGTGTTGTCCAGC
TCACCAGTCGAGTCCAATCTCTTCACCAGTTTTTCCACAAGATCGTCAACT
GAGAGCAGAGTTTGCCACCTCCTTCTGAAGGCGTCATCTAAGAACTTTATT
GAGGAATTCGTCATGGGAGTCTTGGCTTGCCTAATTAACCAGTGCTTGTTC
GTCCCATGGATGTTGAAGTTCTTGTTTCTCGGTGCGATGACATTGGGGAAA

GCCTTCTGGTACTGAGGTGCGGCTGTCCACGGTGAGTGGGGCGCTGGAGT
GGAGATCATCATGAAGAATGGCTCAGAGTTGGACTTGTAGTCCAGGAAGT
CCAGGGAGAGATTGGCCAGAACGTCTGTCAGGTAGTCCACGCTATAGTTC
TCGCCATGTCTGCGTGCCTTCCCGTTGATAGAGAGGGTGTAGTTATAGTAT
TTAGAATTCTTTTCCAGGGCATACCAGTAACTCCAGCCCAGAGGCACGTG
CTCCAGTCCACCCGCATCTGGGGCTCCATACTCATTTAAATACTTCCCAGC
AAAAAAGGTTTGATAGCCGCACACCAACTTAAGGATCGCTGGGAAGGTGT
ATGGCTCCTGGATCTTCTGCCAGGACTTGCTACTGCAGTTCCCCTCCAAAG
TGTTGTTGACGACGTGATGGTTGTGTGGGTACTTCCCAGTCAGGATGCTGG
CTCTGCTGGGGCAGCAGAGAGCACTGGGCACATAAGCGCTGGAGAAAGT
CATCCCCTTCTCTCCGATGAGGGCCTTGGTTTTCTTTAGTGGGGTCATGCC
GCCGAGCTCCGCGTCCTGGTCGTCCGTGAGCAGCAGCAACACGTTGGGCC
TGCGAGCCGCCCCCACGAGCCCCAGGCAGCCGCTCAGCAGCGGCAGTAGC
AGCAGCGCGGGCAGGCGGCTGGGACAGCCCGGCCCGGGCCCAGCTGCGG
ACGGGAGCCGCATGGCCGCCACGCTCCGCGGGAAACCGAGAGCTGCGCA
GAGACACCGGACTCCAGTGAGCTGAAGACCATTCTTCGCGAGCTAAAGTC
CAGGGTTGGCATCCAGTCGGCAAAGTTACTCCGGCAGCTGAAGCAGAAAG
ACAGGCTTTTGCATAAAGTTCAGAAGAACTGCGACATTGTGACCGCCTGC
TTGCAGGCTGTGTCACAGAAGAGAAGTAAGTTCAGGGGTACGGGGTGTCT
GTCATCCCTTGCCACTTTGGCTAGCAGTGAGCCTGCAGCAGGCGCTGGTTT
GCACTGCTTTGAAAAGTCCAGGAACCAACCAAACAGCGCAACTTTTTGTT
TTTAAATTTAAAATGAATGCTTGATGTCTAATGTTAATCCCCAGCTTTATT
ACTCACGAGTTGAGGTCACATTTCACTCTGACAATGGTTACTTTTTTTT
>LNC_010408
GGAAGACATGAAAACTAACGTCTCTCTGATGTGTAATGGTAGCTCTGTGA
TTAAGCCGATTACAGGAACCAAGATATTCAGAATAATCAGCTGTGAATAG
ATAGACTGAATGTGGAGTAGAGTGACGTATCCGATTCCAAAGGTTAGCTG
CAGAACGATGAGTGCCATCCACAGTGAGGGCCCTTTCCGAGGAAGCAACT
CAATTCCAAATGCTGACGTGTTGTAGGCACCGTAGAAGTCAATGTGCAAA
GCAGCATAGTAGTTCACCAACACTGAAGTTGCAGCTAATAGAAAGGCTGA
GCTGTACATGTAAAACTTGAAAAGTGATCGCTGTGACAAGATCAGAACAA
TACTGGATACAACTATACCGAGGAGCCGGAGAAAGATCTGAAGCCCGATG
CAGAACCGGCTTTGGCTGGAATCGTAGTAGGAGTTGAAGATGGCGTCGAT
GATGTAGAGGCAGGGCACCCGGAGCGCCACTTCGAGCGCCGCCCAGACCT
GCTGCTGGGCCATCCGCACCTGCTGCTGCGGGGGCCCCACCGCCGCCATG
AGCCGCTGCCACACCGGGGCTGGGCGGGCCGGGCAGGGCGGCGCGGGGG
AGGCCCGGGGCCGCGGCCGGGGCGGGCGCTGAGAAGAGTGGCTCCGCCC
CCGCCCGCCTCCTGTCCTGTGTCCCGGCCCGCCCCGCCGCCCGCTCGCGTC
CCTCGGTGGAGCAGGGGGCGGCGGCAGCGGCTCCCGGGAG
>LNC_010515
CAGAACCGACCGGAAGAAAGAGGGTAGAATCAAACACAAGTGCTGCCCA
TGTGATCCTATAGTTCCGTGAAAGAGTCCAACAGAACGCGCTGTGGTAGA
GACTTCTATGGCAATACCTTGTTTAAATCTTGTATTCAGGCAAAATAAAAA
GCATTTGGGCTCTTTCTTTTGTATACATGCAATTTTAAAAAAGGTGTTTTCA

TTCTGCAAAGAGTTTTGGTTGGTTTACCTTTGATCTCAGCACTCAGGAGGC
AGAGGTAGGCAGATCTCTATGAGTTCAAGGCCCGCCTGGTCTATGGAGAG
AGTTCCAGGACAGCCAAGGCTGAGAACCTGCCCCGCAGGCTGGGGGTCGC
GGCGACTCCTCCGAGGCCCGAGGGAGGCCAGGACGCTCCCAGAGGTGAA
GTGCGGACCGCCTGGAGCGGGCCGCACGCACATGGGCCCGGCCCCGCGGC
GCGGCGCCTTCGGACTCGGGCGCAGCGGGCGGGAGGCTCGGGACCCGCTC
GGGACCGGCTCGGGGCCGCGGGCGAGATCGCTCCAAAATGGCGGCCGCG
AGTGC
>LNC_010536
GTCTTTTTCTGGTCATTTGGGTCAACACTGATGACGTGATTAATGATGATG
GGTTCTGGTGGCATGAGCAGGGCATGCAGCCGCTGAGGGATCTCTGAGAA
CTTCATCCGCTGAGATTCAAAGATCTGCTGGAGGTACTTGTCACAGATAAC
AAACTCTCGCTCGTGAGGGTCCTGGAGCTTATGTGTTTTAATATATTGCCA
CAGTGCCTGGATGATCACTGGACGTGTCTGGGTGTGGATGCCCAAGAGGC
GAGCCAGGCGAGGGTCTAATTTAAACTGGGGGGGCTGGTAATCCAGCATC
AGCAGGACAGTGCACCGTACATTCACATCTCCTGGTCGCTTCACCTGGAA
GCCGTCTGTCTCCTGGGTAGTGGCGGTCCTGTGCCATTCTACCAGATGGTT
GTCTGGCCCATAGAGGTCTTTGTCCAGTTCGATCACCAAGGACTTAAAAA
AGGAAGAGAACTTTCTCTTTTGCTTGGTGGCATCATATTTGGACAAGGCTG
AATCCTCCAGGAGCCGTCCTTCTACCCGGAGCTCCCAGGAAGCCACAGTC
CCTTCCCCGTCCTCGGCATCCGACTTAGCCGGATTGAATGTGTTAGAAATG
AAAATCCGCAGCTTCCGTTTTTGCTTGATGGGCCGCTTCAAGGCCTCCTGG
ATATCCAGGCGTTTCCTCATAATAGTCTGGTCCAGTTTCCTTTCAAAAGCC
AGGAGATCCATGTAGGCCTGTGATTCCGGTACCAATTCCCGAATCCTCTGA
GGTAGGATTTTGTCAGCCATCTTCTTTTTCTTTGCATTGTGATTTCGATTTT
GGACCGCCTGCTGCTGGACCTGCTGGATCTGTTGAGGTGCAGGTCTCTTGC
GGGATTGGTCCATCCCTGACTGGGCCAGGCCAGGTCGGACTGAAGGGTTC
CCCCCATAGCCAGGAGGTCCCATGGAAGGTCCCTGAGGTGTCATTCGGCT
GCCTGGCAGCATACCTGGTCTCGGATAGGCCGCCCCGGGCATCGGAGAGC
GGTACAGCCCTTGACCCGGCGCCGGGCCCATTCGCACGGGAGGCCCGGGA
GTTCCGCCCGGGCCCAGAGCAGCCGCCACGCCCGCTCCTCCTGAGGCTCC
CGCGCCGCCGCTCGGAGCCACGGACTGGAAACCCGCCCGGGCCGCCATCT
TCCCGGAGCCGCCGCTGCAGCTGCACAAAGAACCGGAACCGGAACTCCCC
CCGCCCCCCGCGCGCCCCCCGAGCGCCTGGGCTGTTTGTGGGCCCGGCCC
GCCGGCGGGCACGGGCGGCTCGGGGCAGCGAGACTACGGGCTAGAGGGG
GCGACTAGCGGGAAAGGCGTGCTGACCACTCCCTCCCGCCTTCACGGGCC
AGGAGGGAGCACCATAGAGAGGCGGTGTGCTGATAAAGAAGTGCCGGGA
GCGAGCGCCCCCGGGCGGTTGGAGGACGTCAAGGCCTTGCAAAGAGCCC
AGCCCACCCCGCAGGCGGCTGGAGTGATGCTTCACCTCGGCCCAGTGCGT
TTACCTCTCCAAAGCATTCTGGTTGGTTTGCTTTTTGTTTTCCTACGGATCA
GACTCCGGATGCTAAAGAGGGAAACAGGCTGTTTGTTCGTTTTACAAACG
GAAAAGCTGAACTCAAAGGAGGTGAATGTCGTGGCAAAGACAAATCGAG
ATAAGAGAAAAGCCATCCTGGATTCGGGTCTCCTGGCTGGTAGCCAGGGC
CTCATCTTATAAATAATGGCTTAAGGGCTCCCCGGCACGGGAACTTAGCC

CTTCACCTCAGACCTTTAACTTCAGATTTTAATGAATTCTTTTACCTTGAAT
CTTCTCCAAAGAGACCCGGATATCTGTTGCTTTAGATTACTTTGCTGGTCC
ACTGCCTAGGGAACAGAGGATTCCTGAATTTGTTCTTTTCCAGGTGGATCA
GTGTTTTTACCTGATCAAGTATGTAGGGTCTCGAATAGCC
>LNC_010576
GGCAGACCGAAGAAGCTGGGTTTGTCATCCTCTGGGAGCTTCTCAATGAC
AGCACGATAGTCCAAAATGCTGCACGAGTTTGGGAGAGAGATGGAATATG
GGAAAATGCTTTTCTTATTCCTTTGGTTTAGTACATCAATTATTGAAGAAT
TAAAAAACTGCTTCAAGTAGGACTGAAGAACTCTAAGATCAAAATAGTTA
TCTATACGTCCTCCATAAATAGAATTTTCAAGTAAGCCATGTACAAATTCC
CACTGTACATCTTTGGAACCATCGAAGAGCCTGTCAATGACGTG
>LNC_010674
GTCACATCTTAAAAAACCACATAGACTAACCAGTTCTGTCACTGAAGCAA
TCAGGGATGTCAACCAAAGAAAGAATGATCAACCATTGTCCCCCGAAAAC
AGCACAAATGAACCTGGCAAGAAATGACAGTGGACTCGGAATCTGCATGT
CAGCAATAGGACAGAGTTTTATTTAAAAGGCTAGTGGCTCAAATGCTATT
GCTTGAGGAGCTCTGGCAGGTATAAAACGTAGTAACATCTGAGGTTTAAC
CACATGTGCAGGAGCTGAAGGCCAGATCACAGGACTAGTTCATCCTTGGT
CAAGAGAAACTACAAGCGCATGCTGCAGGAGTGACCTAATGGACTCATTT
GGACCATATTCCTACTCTCTGAGGATCACAGGAACACCTGGGTCAAAATG
TTCTTTTTACACCCAAAATTCTGGCTTAATTAACTCTCCCTTCCTAAAATTA
TACTCAGTTCAGAAAACAATGTCTTGAAAAACTCAAGAACTTAGGCTACT
AAAAAAATAAAAGGGAATTTCACTGTGGTAGACAATATTAGTAAAGACCA
TGTGTATTTTCCTGTTGCTAATGAAGACATGATACATTCATGACGATATGT
GTTAAATTCTACACAGATGCTCACCAGGGAAAAAACACTTCAACTTCTAT
CTTTCTCCGTCAGGTGGGTCTCAGCACAAACCAAGAGTTCCTCTAACAGCC
CTGTCGGCTGCTCTGGAAGAGGAACAGAAAGCCTAGATCACAGAGCTACT
GAATGTGACCATTGAGCAAAAGCATTGTAGAGCACATTCAATGAGTTACC
TCAGTCCCCAACCTGGTCCAGTTTAAAATGTTTGTAGATCGTTTATGGACA
ACCAATAATGTATTATTTTCTCAAGTGGCAGTTTGCATAGCTCAAGCAAAC
CAGACGGAATGTAAGGAGCTCTCTTGAAATCTCGACCAAGACACTAAATT
ATATAAGGAACCTTAAATGATCAGTGAGCCATCTCCATGGTGGTGCCCGC
CTTTACTCTCAGTCCTCAGGAGGCAGAGGCAGGCAGGTCTCTGTGAGTTC
AACACTAACCTGGTCTACAAGCAAACTCCAGGCCAGGCAGAGCTATACTG
TGAGACTCATAGACCAACAAAAGGAACCTAGTTTAATCTTTACTAAAATA
GTAGACTAAAGATAGCACATCTAAAGCCAGGTGTGGTGGGAACACGCCTT
TAATTTCAGCGCTTGGGAAGCAGAGGCAATTGATCTCTGAGTTCAAGGCC
AGCCTGGCCTACAGAGCTTCAGGATAGCCAGGACTATACAGAGAAACCCT
GCCTCAAAAAAGAAAAACAAAAGGGGGGGAAAAAAAAGACATAGAGAG
CACATCTATTGTGAGAGCAGTTTCACTGTTTCCCGTGGAAAAACATTTTTT
TACTGATAGAATTTAAGAATTAAACCAATAAAAAAACCAACCAAACAAAC
AAGCAAATAAACAAGCATGTCTAGGGATTACCTCAGCGGCAGAGCACTTG
CTTAGTATGTGCTAAGCCCCGGGTTTGATTCCAGTACTGTAAAATATATCT
ATTTATATATAATATATATAAAACGAAAACAAACATATACACCTTAAAAT

AATATAAAACTTGAAATAACATCAGGCATTATGGAATGGGAAACAATTTT
GACACCACCCCCACCTCCTACAAAACAAAGGAAAACAACAGGTTGGGGG
TATGATGACAAGCAGAACTCTGGATGATTAACATTACACAGACCCTACAT
CACAGAACACCACAGAGTGAGTTATACATTAGTAAGATCGTCCTGACGGC
AGTGCAAGTCCTAGATCAAAAATGAGTTAAAAGTTTGCGGTGTAAATAAC
CCACCTGTGAAGAGATGAAGGGGAAGCGGAAAGGCTGCATGCACGGGTC
TATTACAGACATGATATTCAGCAGCAGTAAGAACTCTATCCCAGTCCCCA
GACCTTGTTCCCACTGCTTAAGACTGAGAGGAACAAACTCACTTGTAACT
ATAGCATCTCGCATACTGGAGTGACAAGCATGTTTAATCTGATGCTATATG
TTAATAAAAATACCGAGCATACATGAATCAAACAACACAACACAACATAC
TAACTCATGAACCTTAAAACTGGACCAAAAATAAGTTTAAAGCCAAGTAG
CTGTGATAATTATCACACATGACATAATCCATAGAAAAAAGGGCCAAGAA
AAGAATCTTCATATTGTACTCTTGCTTGATGTTGTCCTAGGAAAAACAGTG
ATAATTCTACAAGTGTGACATAAACAACTAAAAAGTCCCCTTCTGGTTTCA
AATGAATCTTCCTTGGCTAATTTTTCTCCTCTCTTCAAACAAACCTTTATAT
ACAGGACCAATTTAAATCCCTCCTCCCCTTCAAACATACAAGCATCCAGG
AACATTGTACAATGCAAAGTGCCTTAAGAAAGGATTCTGTAGTGCCAGTG
GTCTAACTGATCTGAGGAAGCTGGGATGCTGTTAACACTGATGGGCCAGT
CAAGCAATGGTGGTCTGCTTCTCCTCTGGTGTTTCCTCCAGCAACTGCATG
ATCAGTTCATGGAGGGGTTTGCAGATCTTTGCATAGCCCCCTCCTGTGAGA
TGAAGAAAATCAAACATGTCGTGGCAGGAGATGGCACCGTCCGAGTGCAC
GAAGCCCCCATCTATATCCAGGAGCTGCACATTGGCAAGCTTCGGCAGGG
AAACCTTGAGAAGCTGGTTCACCTTGGCATTCTTTTGTCTTAAAGGGTTGG
GCTTCTCACCTCGAGGTAACAGACCCAGTACAATGATCTTGGCCTGTGGCT
GCCTCGTGTTTATAAGCTGTACGATAGCCTCAATTCCACCTGCTACTTCTT
CTGCTGTATTTTCATGGTTGTTTGTTCCTACCCAGACAACAATGACCTTAG
GCTTAATGTTCTCCAGCTCTCCGTTCTTTAGTCTCCATAAAACATGTCGTGT
TGTGTCTCCCCCAATTCCAAAATTAAGAGCATGAAGTGGGGAAAACAGCT
CCCGCCATATCTCGTACTGCTGCATCAACTGTACCATGGAGTCTCCCACAA
ACAGCACGTCCGGCTCTTTGTCTTTGCAGTCCAGAACAAACCTATTGTGCT
GAGACATCCACCTGTCATCTCCTTGAATATCTTCTGCTGCATGTGGAATAG
CTGCTGGGTTTGAGTCTCCTTGGCTCATTCTACACAGAGGCAGAGGGTCCG
TACGGGCCCTCAGCGGGTGCGGCGTCGGCTGGTTACGCTTCAGTCGCTCG
CTTGATCCCGACGGCCGCGTTCCGGCGCGCGTCCCCTCCTCGGGCCCCAGC
ACCAACCAGCAGCGCCTCCACTTCCGGTCCCCCTGCCACTCCGGCAAATG
GCTTCCGGAAGTCCCGCCTTTTGCTGCGGGGATAGCTGCCGGGTGGGTGC
ACCTCACTTTCCCTCGCAGTCCGTGCTCTTTGCTTGGCTCACAGGTCTTCAA
CCATCCCTGTCAAGTTACCGACTCTTTCTCTCCCTGACTTGAGCCTCCATCT
TTCTTGTAACTGCCTTCCTGTCCTGATAGGATGATGGAAACCATCCGGAGA
CGGCTTAGGGACAGTGTAGCTTGACCTCACGCTGACGCCATTCCCTTCTCA
GCCACTCAACCTGATTTCCTCCACCGATGGGATAAACCAGTTTGCAGAAA
ATGGGGAGAAATAGGTCCGAACTTAAAAGAGACAGTCCCCTACATTTACC
AGCAGAATTTCTACTTCTATGATGAAATCTGAAATTGTGGCTCACCGCCTC
TAAGGTAATAAATACCACTGGGGGAATGACAGAACCCCCAGCCTTTACCT

TAAAAATTCGTCCAGTTTGGGGCTGGGGATTTAGCTCAGTGGTAGAGCGC
TTACCTAGGAAGCGCAAGGCCCTGGGT
>LNC_010697
GATTGTAAATGTCCCACCCTGGAACTCGTGAGGCTGAAGTTTACCCTCTCT
TGCTTTGGAGGCTAAAGAAACAACATCACTAGCAATGGTTTCCAGTCCTTT
TATGTGTGCATTAAACACAATAGGGGTGATAAGTCCTGCAGGGGTACTGA
CAGCAACGCTGACATCAACCACATGATTTTGTCGTATAACTGTGTCCATCC
AAGATGAGTTTGCTTCAGGAACTTTCAGGCAGGCCAAAGCTGAAGCTTTT
ATGATGAAGTCATTGACGGAGATTTTTCCTTTACCTTCAAGCATCTTATTA
AGTTCCTTCCGCACCAACAGCACCTCTCCCATATTTACATCAACAGAAAGG
TAATAATGAGGTATAGTCTGCTTCGACTGCATGAGCCTCTGCGCAATCACT
CGACGAATGTTGCTGATGGGGATGTCTATGAAGACACCTGCAGGAGTTGG
TGCCACTCTTGGACCCGGGGGAGCAGCTGCTGCAGCGGCAGGAGCAGCCT
TAGTAGGCACAAAAGAGTCAATGTCCTTCTTGATGATTCTGCCTTCTGGTC
CCGTCCCTTTTACACTGGCCCACTTCTAGAAAAAAATTGGTCAAATTGATT
CATCTTCCCCCATGGCAGCCTTTTAAAGGATTCCTGTTGTGTGCAGTTCTTC
CCAATTCCTTTCCTTCGTTATGAAAACAGAGTTGGCTAGAACTGTCTCTCC
TCACCAGGAGCTCTAGAACAGAGGTGGCAGGCAGGGA
>LNC_010816
CACATTTTCAAAAGCATATTTTAATTTTTTAAAATTTTAATTCAAGTACAA
GTCAGCTAGTTAACATAAACAGAACATAAATGCACATAATTTGCAGCCAT
GTACAAAAGTGCAACAAGAGCAACACTCGCATGGCCCGGCAAAGCTCGTT
AGCTCTCAGGCTGCCTAGACTGACTCATTGGTGTAATGTACTTCACATCCA
CTGGGGTACATTCAGAAAGCTTTGCATGACTCATCACAACTAGTCCTTTAG
TTTCTAAACAGATAGCATAAGAATCCAGGTATAATTATGGAGAAACAAGG
TTATAGGGGAGAAGATATGTGAGGGGCAGAAAGCACTCTTTATTTCATAT
GGTAAGATTCTGTCCTATTTACGCGGACGTATGGATCTTAGGAATGTTGAA
AGTCATGGTAAAGGAGGATCACGGGCTGTGTATCAGCCCTCAGGAGTCCC
TACTGTAAGGGCAGTTTGGACAGGAGAGCCTGTGAACTGCGAAGTAGGAA
GAAAGTCCTCAGAGATAGGGGGAGACCAATTTCTACCACTTACATTCTTC
AAATGAAATCATTATTTCACGGTGATTTTTAAAAGCCAAAAATTTTTAGAC
TTTAGTAAATGAACAGTTATTCTTCTGAACACCACAGATGTGCTTATCCTA
ACAAGGCTACTGCAAGGAGACATCCAGGTTTATGTGACAAAAAGGTCAGG
CTGTGTTGTGTGACCTAACAGTGCTGTCATGGTATCAGAATTACAAGCCTG
CTTGAACCGCTTTAATCAATCAAGTTGGTTCTACATAGTTGTACAGTGAGA
ACCGGAACAGAGTTTGTGAGCATGGAGAGGCAGGCCTGTAACACCAGCA
CTTAGGAGGCAAAGGCAGAGAACCAGGAGACAAGGTCTTCTTAGGTAAC
AAGAGCCCCTATCACCACAAAAACAAAACAAAATTTGAAGAAAAAGGCA
AACCAATGCCAGGACAGAGGTCTCGGGGAGGCCGCTCCCTGGTAACTTAA
AAGCACTGTATGGTACCTACTAAAGCTTGCGATTGATTCCCCAGCTGGAGT
GGTGTCCGAGTTCAAGCACTTGAGCACAGCAGTCTGCTGCTGTGAGGGCT
GCTTCTAGAAGCTACTACTCCAGCCCGGCTGAGGAAGTTCAATTTTCTGAG
AAGAAAGACTGCAACAAAAGTATACAGCAAGGTCCACCCGAGCTCTTTAC
ATTTAAAAAAAGATTATATGATTAAAACAAGTTTTTTTAAAAAAAACAA

TGCTCTGAGTATGTATTTTTTTAAAAAAAATTACAATTAAAATCTTCAAAG
AAATACTTCATAGACAAATTGTGTGACACATAAATGACTTTCTGTTCTGGA
TTGTCTGTCATACAGTAAGCCATGGTAGACCAATGCTCAGGGAAAACACA
AGGCCGGCCTCACTGCTCCCTGCCAGCGGTTGCTTTTCAAGTAAACACTTG
AGCCTCTAAAACCCCCTTCCTGCAGGTCACAGGCTAACTGCAAAAGCTGA
GTGCAATGCACTAGACATTAATGTAGGTTTACTTGCCAAAACCAGCCTA
AGACATGCTCATCCCTGAAGGACACTTATCATGGATTTACTGTGACGACG
ACATAAGCTGTCCCTTGGCTGCTGGTTTTCACTTCACTCTTCATCATCTGAG
ATCTGTGTGACCCAGAGAACATGTCCTACATTCAACACAATAGAGTGTTA
TGGATCTTCACAGAGCAAATGGCGCTGCATTAGGCAGTATCAATGGCACC
TGAAGAGGAAACACTATAGCCTGAGACCTTCTCTACAGATTTAAGCTAAA
CCCCCAGGTAGGGATTAATCTTTTGGTATGTTCCATGTAACAGATGTCAAA
GCTGCAATAATTCCAAAGGTAACGATCCATACCTGCCTTTGGTCTCCAAAT
AGATTGGAATATTCTTAATTCCAATTGAGCACCTCAAAAACATCCAGTTAC
TGATTACAAGGAAGTCAAGGTGAGACATTTAAGAGGGAATTAAACTTCAT
TGGTGACAGCAGAGAAAGCGCTCAGTGGCATGCGGCTTTTCCATAATACT
GTGGTGTCACACAGCACATGTACAATACTGTAAGATCTCCAAAACATACC
TAAAGTCCAGAAAAATATGAACACTTCTTCTGCCTAATACATAGCAATGTT
AAAGTAATATGAATTCTATTCTTACCCTTTCAAAAGGCCACAAAGTGCATT
TCCAAAGTCATTCTGAAGAGAAAGCCATTGCACAAAATGTAAAGTTAAGT
AAGGTACACTGATGAGTACCACAGTAGAGTTTAATTGTAGAAGTTCTATA
TACCAGTATAGCAACATCCATAGGAAAATCTACAGTATTGCAGCAAACAC
CTTTATAATGGCCACATGTATATTAAAGTATTCAAAGATGGTGCTGACTCA
ATAGTAACATTTAAAAACTATCTTGTTTACAAGGAACTAAAACTAACCTG
GCTCTGAGAGAGCAGTTAGGGAGCTCGGGCAGAGCGCTTGCCCAGTGCAG
CACACTCACCTATAGGCTCTCTGCCAACTAAGATTTGTTGTATCTGATGGC
TGAGGGTGTCTTTTTGTAAGATTTATATGTTCCTAAATATTAGTATCACTTC
AAGGAGCCTGTAAAGGGCAAAATTGAAAATTACCATTTCCTCTACCAACC
AAGAATAACCTATACAGATGTGGAAACAATTCAAAGGACGAAATTCTTAA
TGTGAAAGTAAGCAGAAAAAAATTCAAGTCATTAGTTTCTATATTTAGAGT
AAATAGTATGTAGGTATGAGACTGTGATAATCACATTATGAATCAAAATT
GCACCCATTCCTGCATCACATAATTGGAGAAGGTATGAGGATGTGCTCTA
CGTCACTGAGTCAGTCAGTATCCAATAGCTTATTTTATAGTCCAAAACCAT
TAACTCCGTGCATCTGATGTGTTAAAGTCCACATTATGATGAATACCTTTT
AGCTGTGACCAAAACATTTTAATGTTGAAAATGTGAAGTATTTAGACACT
ATTTCACTGGTTGTCAAAACACAGTGATTACTGGTGTCAAATAACTTAAAA
CACTGTAACAGGCAGGCCCTTCTAGGGAACTGGGATATCGAGATCTACTT
GTTCTTTTACAGTACTGCAGGAGAAGTCCGGTTAGTCATCTGTAATTGTCA
CTGTCATTTTTGGAAAATTACAGTACAGTATATACTGAGGAATGATTTTGT
CTAAACAACCAAAAATGCTCATGGAATTAACACTATATTCTGACTGTCAT
AAACCAGTAAAACAAATCTGGAGCTGGTGAATAGTAGAGAAAATGGCAA
TTTTGAGATAGGAATGCTTTTCAAAGCATTCTCTAAGAGGCAGCCCAGGG
TGTCCTCACTGAAAGATCAATGTGGTGTGGTAGTACCTTACTTCCTAAAAC
CAAACTTCCACTCAGCCACAGTAGTTACATGTTTCCCCACTACTGTCTTCT

GACCACCATGCTGGCTTCAATATTCCATGGTCCACTGTGATCGTTTGGTCT
TCCTGCTCTGCAGCACGGCAGCCCCTGCTGCTATCCGTTGAGTAGGTAGAC
CACCAGTTCGTAGGTGGCCATCATAATGGCTGTGTTTGGAATCTGTCTCAC
CAGATGAGTTGTTAGGCCACGGTAAAGAGACCCATAGCCTTCTTCTTGAA
CAATCAAAGACAGTGTCTGAAAAAAAGATCTGTATTTTGTCCCTTCTTCAC
GTAGTCTTGTTCTTACAACTTCGTGTGGATATGCGACGGTTGTGGCACAGG
TTTTTGATGTGGCAGCAGCTAGCATCATTCTCACAAAATCCGATGCTTCCT
TCACAGACTCCTCGTCAGTCTCCATCATAGAAGCAGTCTTACACTCCAGTA
GCTTTTGCTTTATACTTTCATAAATAACAAAATGGATAACTGTCTCTGATA
TGCCAGCATATGAGGCAGACATGCCCCTATAAAATCCTCTCAGTCCGTCTG
TCTGATATACTTTACGAACACATTCAAAAGCACCCATTCGTTTCTCCCCAC
GATTCCTTGCATCAAGCTGCAGCCGAGTCTTTATAAGCCAAATGGGGTTTG
TTGCAGTGATCGCAGTAAAACGAGGTTTAAGTTAGTCCATATTTCTACAGG
AAGTGACCTGCGGATGGGGAGCATGAGATGACACGATCTCACTCTTTTCT
CGGGAGAAATGTACAGTAAATGCCTGCCATTGCAGCTGAAATCATGTGTA
CTTGGGTAGAGTCAGGATCAAAAACACCGTTCAACTTTTCCTTGCAGTTTG
AATAGGCAGCAAAGTATATTGCTCTGGAGGGAGCCACTCCCACCAAATTG
GGGCCTAATCCTCTAAATAATGAGCGAGGGCCCTCTTTTTCCAGGATAACC
TTTAGACAATGAAGAGGTCCAGGGGACACTACTCGGTTGACACTGGCTCC
AGCCATGGTGTTCAGCTGAACTTCAGAAATATATAGTGTCACAGAAGATG
ACTGCAGTCGTGTTTTGACAACTTCCAGTGGACATGTCAGAATAGCTCCCA
CTGTACCACCACATCCCCCGGCAAACAGATGCACCAGCGTGTCCCTCTGG
CTCATTCTCTCTTCTGCATGTCCTCAGGGAGCAGAGGCGGGACGCGGACC
CAGCAGGCCGCCACGGCCAGCGGCGAGGGCCAAGAAGAGGGACGTCGGG
GAGTGAGCGGAGCCGCCGCGGCGGATGGCCACCCGCAAACCCACCCCGCT
GGGTTCCTGCTCCAGCGAGCTCCGCCGGACTGCGCAGCGCGAGTCGT
>LNC_010820
TAAATGGCTGGGCCAGGAGATGGTGGGAGTGCAGACATGCCTGAAGCCA
CAGTGACCTAGTGAAATGCTCTAGGAAAAGGAATCAGCTGCCCAAACTAC
AAGCTTTTCTGGGGCAACAAGTTGAAATGGCCATTACCTGTCTGTCCTGCT
TAATCTCTGTTAGCTTACTGTCTGTTAGCTGTAATCTCTAACAGCTAATAC
AGGCAGCAATAAAACATCTCTTCCTAATACAAAATGAAAATAATTCCCTT
TAAAACTTGGGCATACTGGCTCTATGGGTTTCCACACTTCGGAGTTATGTT
TAAGGCTTATGCTGAAGCTAATGCTGGTATCTGCCTCGATTTAAATGTAGC
TTAACAACCACTCTACACTTAAAATTGGAGCAAATTATTACTTTCAAAAAC
AAAGGAGTGAAATAAGTCTGCAGAATCCTAATTCTGGACTAATCACTTGT
TCTCTTTTTAGTAGAGAATACATCCTCTTCCATCCAACATAGGCTGTGCAG
TTGTTCACAGTCCAGGACTGTTAGGAAGTTAAAAGCTCTTCCTGGCAAGC
ACGAACCATTTCAGAAGTTAACTTTTACAGAATAAGGAGAGTACCACATA
TTATTACCACACACCATAGCTTCAGTATCCAGATATTCTATCCTTATACTA
AGTGCATCATTGTCTGCATTTGAGGAGTTTGAGGAGACAGTTTATAGGTCA
CATAATACAAAAAGGCACAGCATTTCTGACCACCCCCAGGTAGGCCACCG
GATGAACTGAATCACACTACGTGCTGGTGAAGCAGGCTACCCAGGAATTA
GCACTAGGAAAGCTGTCCTATGGACAGCAACGCTGCATCTTAGAGGAGTG

TAAGCGTTGAGTGCGACATTCAAATGGCAAGTAAGACACTTCCATGGTTT
GAGGATGGGATGACTACTGACACCTCTTCTTACTCAATCTATTAGTCCACC
AAGGGCTTCAGGGAAAGTTAACTACATGCATACCTCAGAAATACTGAGTT
CTGTCCATAACCACAGCAATAAACAAGTCATGAATTTTAAGTTTACTAGTG
TAGTTATACTATACAGTAGTCTATTAACGGTGCAATATATTAGTCTTTTTTT
ACAAAATGGTAATTTTAATAAACAACAGCATCAACAAACTTCCTTAACTA
AAGACACCAACTATCCCTTTTTTGTTTTTAGACAGGGCCTAACTAAGTAGA
CCAGGCTGGCCTGAAACTCAAAAGCTCCTCCCATTTCAGCCCCCAGGCAT
CACCATTCCCAGGCAGACTTTAATTTGTAAAGATGTAAAACCTTCACAGC
ACAGTATGAGTAATGTTTGTTATGTTCAGCTAGCAACTTGGGAGCAACATC
AACCTGATGCCAAAAATACATTCTTTCACACCACTTTGAAAATAAGTTTAC
TGAAACCTTAGAAGCTTTTCGAAAAACCAAGGGCCATCAAAAAAAGTTAA
TTCCATAAGCTAAACATGTCAACGGGGAAGTCCTACAGGTAGAGTAGTTC
TGCTAGAAAATAGGTGCCCACCAGATCTTACATTTACTTTACAATTGCCAT
GTAATAGCCTTGAGTGCCTAGAAAACCCACCAATACCCAGGAACACTGGA
AAGTAAGGGTGAAAAAACAATCTCAGTCTGCTCTTAAGATGCAGTCATAC
ATATTTTCTGATTATGTCACAAAAGCAAAGACTAGAAGAACTATGCTAGA
GAAGCAAATCCAATGGAAATCAGTGAAAACCTATCACTGGAGAATACACT
GCCATTGCAAAAATACTGACTTACTGTTGGGCAGTAAATGACTAATTATTC
AGGTCACTCATACTTACAAGTCTCTCTGGATCTGATCGATGAGCAAACCAT
CGATCTTTTTGTTATTTACCAAATACCAAGCCATTCCACCAAAGTGTCTTG
TTGAACGTAAAATCTCATATTTTCCATGTCTGTCTATATCTTCGTAGGTCTG
GTGATGAACCTTGATATACTCAGTGGAATCTGGCTCAAACTGGGCAACAC
AGAGATTGAGGACATCAGCATGCCGGTCTTGGCTGTACATGGTCTTAAGG
TTCTCATCTTTGAAAATTACTGGTGGCAAAACTCTACGACCTTCTTTCGGG
AAATAAATTTGTATCACCCTGTCCCGTTCTTCCCATGAAGCTTTGCGTAGT
GTGCCACTTGGTTCTCTAACAACAATAAAACGTTCCTGAAACAGAAAGCA
GCCAGAGATGGATAAGTTGTGAAGACTTCCGTTTAGCTCTTGCGATAGCA
AACACTATGAACGTGGCAGGATCTACAATCACCTTAGAGACAATGTGAGA
GAGGCATTATCTAGATTATGCTGAGACAGGCAGCCCCACCCTAATGTGGG
CAGCACCCTT
>LNC_010869
TGCTTAGCCATCTCCTCAGGGCCCACCCAGACCCTTCAGAGAACTGAAAA
TGAGGTCGGTTACCGAGGTTCTGGGGGGTGAGTCAGAACCACTGCCTGAG
CTGCTCTTCTTTGTTCTCTGGGCCAGGGATGTGCCAAATCGGAGGGCTTCA
TACACTGGGTCCACTTTATTGCCACAAGCAATGGGCATAACTTCTTTAATG
CATCCAAACTGTTCGTGAATGAGCAAGGGGGAGCTGTAGTAACGCCGGAA
TCCTTTTGGCAACTTGCCCATGCACCGCTGGGGTGCCAGGCCGTCTGCACA
CTTGTGATGAATGCTCATCTTGCAGGCTCCACAGCGTAGTCCGTGCTTAGC
ATGTGTTCCCACGATCATGTGGTTGCAGACATCACAGAAGGTGGGCTTCTT
AAAGACATGCTCCTGAAAGGCGTGGACCTTGCTGTTGCTGCCGGGGTGCA
GGCCAGCCTTGGTGGGCATGGATGCTGGGCTTCCAGGGATGGGTATTGGG
CTGGGGCCTGGGCTAGCCTTGGCCAGCAGGTCTGCTTGTGGTTTCACATCA
CTGTTGGTCCTTGGGAAGAAGTTGTCGGCACTTTTGCTCCGCAAGCTCTTG

GTCTTGAAAGAAAGTGACCGTTTTAGTTTCTGGAGCTTGGATTCCAGGCTG
CTGGTGGACGCAGGAGAGGGCGGCTGCTCTGTGCCGGTTGCCTCCCGGGT
CAGTCCGTCTCCGCTGTCCTCGCGGGCGCCACTTGGAGGGATCATCTTGGC
CGCGGGAACTGTGTTGGGCTTCTGGGGGAACCGCAGGTTCGCTCGACTTC
TTGCTCGCTCGCTCAGTGCCGTGTCCCCGGGATGTCCATCGTGCCAGGCGG
CGGCGTGCGCCCTGGGGCCTCGCCTGGCCAGGTCCAGCCCACCTCCCGGG
CCTCCGCCCCGCCCCGCGCAGCGCCTGTGGCCACCTCGCTGGAGATTGCG
CGCCTCTAGTCGTGACTGCCGCCCACCGCAGCGGGCGGGTGGGCCCGGGC
TGGCCCCTCCGAG
>LNC_010886
GAAGAACTGGAGTGTCTGCTCCCTTCCACCTCCCCTGAGGCTAGTGTACAG
CTAGCAGAGGCAGGTCCCTCTGAAGGGCCCAGAATCTTCCATCCTAGAAA
TCAAGTGGCCAAGTATACTGCCTCTCTCATCCAGTGTAAGGATTAGAATG
AGGAAGGGCATTGGGATTCGGGGTGCTCCTCAGTACCAGTGTGGCCGCTT
GTGGACACAGCCTCTTGTCCCCCACCCCATCTTCATATGGACATTGTGTCG
GGCCTGCAGCCTCTCCCTCCCGGGAAGGTGCCAGTGCTTCACAGACACAT
CTAGATCCATCTCCTGCCTCTATAGAAGCCGGACATTGCTCCAGTGTTCTG
TTCCTTTCAATCCAGGACTACATGTGAACTCTAGCAAAGCCCATGCCTCGT
TAGAGGCTGCAGCCTAGAGAGCTACACAGCTAAGGAACACCAAATGTCAC
CAGGGGCCTTCTCACAACCCAGGCTCTGGATCTGGGGCAGCCTGGACTTG
GCCGGCTGGCCCCTCAGTCTCTGCCATGCAATCCAGGCTGGCTGTGTCTGC
TGGTTCCTCGGTCTGTGCTTCCAGCAGTTCTGCAGACAGCCCTTCTTCTGG
TGAAGCTGCAGACGTGAGGCCATCAGGGAGAGTCAGCCGGGGCAGGTGG
TCCACCTGGGCCTGGGAGCCTGGACTGTCGGGGAAGGAGATCTCGAACTC
TGACCCTGACCACGTGGCCTCACCACCCCGCTCATGAGAACTGCTGCTGG
AACCAATGTCCTCTGGTCCTAAGGCTGAAACAGCTGTAGGGGTCAGCAAT
GAGGAAGATGACAAGCTGGGGCACACACTTGTATTGCTGCTGTCCAGGAG
GGACATGCCTGACACATCCAGGAAGCCTGGGTCCATGACCCGCTGGACTG
GAGGTGGAGGCTGAAGCGGGATCTCCATGGAATGTAGGCATCCCCCATAG
TCAGCAGTGCCATGGCCAGGACTCCCAGGCCCAGGGAGCTGCTCCTCCAT
AAAGCCACCAGGGACCTGTGGTAAGGCCTCCATGTAGTCACTCTGCCTGG
AACCCAGCTCTGGGCCAGAAGAGTCCTGAGGGATGGGCCAAGGTGGTGG
GGCACCAAGAGGAAAGGCATTAGGTTGTGGGGTCTTAGCTGGAGTGCCAG
GTGATGAGGCACTGGGTGGCTTCTTGTACTGGCTGGAGGGCTGCGTTGGG
ATCCGGTGCAGGTACCCGTAACCAATACCACACCCCAGACTGCCCTCTCC
ACCCCAGGCTGCATTGGGGGTCACAGTCACCTCCCGGCAGGAGTCGGATT
CAGAGTTATAAACCATCAGCTTCAGGGGCTTTCCCTCATGGGACTCAATG
AGAGTGAAGAAGTCTTCCGACTCCTGGAGGATCTGGTCAGAGCCAACAAT
GTAGTCTGTGTAAGGGCGCAGGCCGGCCAAGGCAGCGGGTGAAGAGGGC
TCCACATCCAGCACATGCCACACGTGCTCGCTGGCCCTGCGGAAGCTGCA
GAAGCGCACGCTAGCTCCCAGCAGGCCCTGGCCGCCCCACATGTTGCTGG
GCACCACCTCCACCTCTCGCACCCTCATGGTCTTCATGTTGAACACCTCCA
GCTTCACCGGCTTCTCCACATTAGCCTTCAGCAGTGCCTTCAGCGTGTCAT
TCTCCTTGTTCAGCCTTGAGTGCCCGATGGTGATGATGAAGTCAAAGTAGG

GCTCCAGGCCCGCCTGCTGGGCTGGCGAGTTCTCTTGTACAGAGGGGGCC
CCGGTTCTTGGACGCGCTGGGCCTCGGAAGGCATGCAGGAAGGTCACTGC
AGGCCTTGGGGCTATGCAACCCTC
>LNC_010922
CAAGAATCGACAAATGGGATCTCATAAAACTGCAAAGCTTCTGTAAGGCA
AAGGACACTGTGGTTAGGACAAAACGGCAACCAACAGATTGGGAAAAGA
TCTTTACCAATCCTACAACAGATAGAGGCCTTATATCCAAAATATACAAA
GAACTCAAGAAGTTAGACCGCAGGGAGACAAATAACCCTATTAAAAAT
GGGGTTCAGAGCTAAACAAACAATTCACAGCTGAGGAATGCCGAATGACT
GAGAAACACCTAAAGAAATGTTCAACATCTTTAGTCATAAGGGAAATGCA
AATCAAAACAACCCTGAGATTTCACCTCACACCAGTGAG
>LNC_010923
GTCTTGACCAAAGCACATGACAGAAACAACTCAAGGAAGGTAAGGTTTAT
TATTTTGGCTCACGGTTCCAGAGGATACAATCTATAATGGCTGAGGAGGC
AGCAGTGGAAGTATGGGACAGAGGTAATTCATACATCCTGACTGGATAAG
ACCAGCAGGAGTATATAATCCTCACCCATCGATATCCTTAAGGTTTCACAG
CCTTCAAAATAGAGCCTTAAGTTGAAAGACCAACAGCTCAAAACAAGCCT
CCAGGTACATTTCAGATTCAAGCCTCAACACACACCTTAATATATTTCAAC
GAAGCTACACATTCACAATTTAAATACAGTTTTTCATCAGGGAGACAGAT
ATAATTCATAAGAGTGTTTAGACTACAAATCAGATACAATTTAGTCAAAA
TACCATAAAGAGTACTTTAAGTAATCAGGAAAGAGATTTTAACATTATTG
CTTACTCTTATGAATTTTAACCAAATTGATAATTCTATAATCTTAGTTTATT
TTCTATAGACTTAAATTATACTGCTTGCCAGGTAATGCAAGTATTGATGCA
ACTTTCTGAAATTAATTGAACTGCAAACAAATTATAAACTTAGCCAGCAC
ACACATTTTGACTCCACTGCTTCAGAAGTAGTACTATAAAAAGTAACCACT
GTATAGGGAAAAACAAAGAAAAGTCTTTCCTTAAAAATTTTCAAGAATTA
TAGTAACAAATGTTCATGGAAAGGCCAATCCAATTTTTTCTATATACCACT
TTGCTTTCCCATTTAAGGGAAAAAGGAGTTATCAGAATTTAAAAAAAAAA
ATCCTCTGTCATTTCCTGTTCATAATAATTTATACCAAAATATACTGTACTG
CAGTTATACTTATGGGTCCTTTCCCTCCCTTCAAGTGTAAGGGCAACAACT
ATAGTTCTAGTGAAGAGACTTGAATAATCATATTATGCAACTCAAAAGAA
GATAGAGGCACAAAAATTTAACAGACTTTGGCTTTGAAGAACCTCATGAT
AACCCTAAAATGGCAGATTAAAAACAACAAAACGATTGCAGCAGCAAGC
AACTACATCACCACACACATTAAGAACTCTAAGTTAGACAAGGTTTCTATT
TCTCTTCTAATCAATCAGCTATATCTTTTTGTTTCTCCTGCTTTTTGGTTGTA
GGTAGTTCAAGACTTGCCCACTGCATGGGCTCTGCCTTTCTCATAGTGATC
TCAATCTTCGTTGCAGTCATAGTTACGTAACTTCGTTTAACATCAATCACA
CCCCATAGTTTCACATTTTGATGAAATTCTTTCTCTCCTTCAAATACAATGT
GTACATTTAACAATGTACTATTTGCTTCTACTTGACTGAGTTCTGGCAGAG
AATTTTTAGCATACACTGAGATGGTAACTTCACCCCCAGTCTGATGCCAGT
CGTGTCTACATGGAACAACTTTTTTCCCAGCATCTTTTTTAGTCCATACATG
TTTCCCTCTTGTACATCCCTCTTGGGCTAAGAATGTATTAAAATCTGAAGT
TTTTCTTCTACAGCAGCTCCAATATTTCATCCCCTCGTGGAAAATGGGTAC
TCCAGAATGATATACACAAACTTCTTCTAGACTCTGTAGACCTTGGTATGT

CTTTGAACACCCTCCATTTTTACAAGAAGTCCCAATCTTAATTTCATCACT
GTCTTCTTCTTTTTTATCTTCTTCATTCCCAGATGACAGTTTAAGTTTATCA
AGTGCTTGTTTTAGGGAAGCAGATATTTTTAATTCCAAGTTTGTCATTGGC
TCATCTGGGCTTGGCCTTTTTATTGCTTCTACTGGTTTAGGGGCTTGAATGA
TGTGCTCCTGAAATTTGGGTTTTAATTCAGATAGTTCCTTCTTCTCAGTAGT
CTTGACTTCGGGCTTGACTGGTTCCGGTGGCTTCTCACTATTATGCCTACCT
TTTGTACATCCTACAATACTTAAGAAGTCAGAAAAATCCGTTGTTCTTCTC
TTACAGCAAGACCAACCCTTTAGGGCATCATGAAAGACAGGAACGCCTGG
GTGGTATGTGCAAGCATCATCCGCATTGGCCTCGGGGTCGAAACGCTGGC
CACAGCCGCGGTTGTAGCACAGGAGCGCCATTCCGTTTTCCCGGCGAAAC
ACACACCACCCAGGAGTAGAAGTGAATCACCACGGCAATCACCTCACCGG
CACTACTGTTCAGGCCGCAGCTAGCTTCCGGAAGCCGCCGGTACTGCTTCA
GGAATCTTCCCGAACGTTCCGGTTGCACAGGCGCAGAAGAGACGGGAAG
AGGGGGGCCAGCGCCGTGCGGTGTACCATAGATTTCCTGCCGGACTCCTA
GAGGTGAGCACAGTGGAGGTGGACCCGGACAGCTATAGATGAGATACAC
TCGGGCGGAAGCGGAAGACTGGAAGAGCCGCGCTCGCTTTGTGCTCGCCC
CTCCCCCAATTCTCCACCGGTGCTGTGTGCTGCGCCGCACGCCGAATTCTC
CGCGCAGCCGCAGTCGCTTGGCAGAGGGTGAGAGAAGGGTGAGAACCGC
CCCCTTTCCGGTCCCTCGGCCCAGATCTG
>LNC_010927
CTGGTGGGGCTCGGAGGTAGCTTGCTGCCCTGGGAGTTGAGCTGGTGGAA
AGCATTCTTGCCAGCACCCCAGGCTGTGGAAAGGAAGGCTTCTTAGAACG
GGAGAACAGTTTGGACACCCGGGCTTAGGGCATGGTGGGCAAAGTGCCAT
CCCTGAGACAGGGCCCCACACCTCACAGGAAGGTAGTGACACAGGACCA
CCACGCAGCGGTTCCAGATTTAATGGAAGGTGCACGGTGCGCTGCTTCGC
ACCCTGAGGCCGAGCTGCTGCAGCGCCAGGTATGGAGCGTCTGCTTTTCG
CAACGGAACAGAAGAACTGAGCATACAACTAACAAGAAAAAGGCCCTCC
TGCCTGCCTCTGAGACCGGGGGATCAGGTCACCTCTGACCCCCTGAGCCT
GGGGGCCTCCCCACCTCCACTGCCCTTCTGAAGTCGATTCCTCCAGAAGGA
GCCTAGCTAGGACAGCCCCAAATGGGGGCAAACAGGGAGCGGGATGCTC
AGAGTCCCCTTCTCAGAGCCCCCCTCCCAGTCATTTTAGGAACAATAAATA
CTGTGTGACAGTAGCAGGAAGGGCGTCCATGGGCTGTATGTGTGGCAGCC
CAAGCAGGCTGGCCAGGTGCCTGCCCTCATTCTGTGCGTAGGATGATATG
GATGCCTGAATCCGTGAACACCGGCCCACTCATTTCCCCGGTCCGTAGAG
CAAACGATGCATCTTCGAACGGTTTCTGCATCTGACCTCTGCTGAAGGCAC
CCAGGTCTCCCCTGGCTTTGGCAGAGCTGCAGTCACTGAACTGTGAGGCC
AGAGATTCAAAGTCCTCCTCTCCTGACTTAATCTTCTGGATGTAGCCATTG
ATCAGCTCCAAGGCCTCCTCTTTGCTCCTGGTGATCTTTTCCTGCCGCCAG
GATGAGGGCCTCCGAGATTGGCTGTGCTTCACCAGCAGGTGTGAGCAGCG
CACCCTGGCAGGCTCACCCTGGCCATTCTTGCTGCCGCCTCCAACGGTGCT
GCCTCCGCTGGGCCGCTCCCACTGGCTGGCGTTGGTGATGTGATTGAAGTA
GTAGACACGGCCTGAGCTGCGACTCATACGCTTCTCCCACCCGGACGGCA
GCTTCTCCTCGTCCGCCATCTTCCCTCCTGCCGCAGCGCCTGCTCCGCC
>LNC_010933

CAAAATCTTTTGTAAAAAAGAGTCCTTCAATATTCCCTGTACCCCAGCGTC
CTTCCCCAGCAACCAGGTCATGTGAGTGTGATTCCCACAGAGGCCAGTGC
TTCAATGGCCCAGGCAGGACAGAGGCTGTGGTCCCCATACATGGTCCGCA
TGAAGTTCCGCACGAAGTCGGGAAAGCGCTGTTCCAAGATACTGCTGCGC
GCGGCGCTCAGGAGCTGCAGCTGATAGGCAATGTTGTGCACGGTGAGGTG
GTGCAGCGCTGCGGTGTTGTCACTGTGCAGCAGGGTGTGCAAGAAGGCGC
GGCTGTGCGTCTGGCAGGTGGCACAGGGACACTCGGGGTTTATGGGGCTG
AAGTCCTTGGCGTACTGCTGCTTCTTCAACTGCAGGTTCCCAG
>LNC_010947
CTTTCATATTAGAGCACAACTGGAAGGCTCAAACAGTTTACTGTGCAGAC
AAAGGAGCCTACCTTTATCTTCTTAACCAGCTTATTCTCTTCTTCATCATCC
GTTTCTGGAAATTCATATATTTTAATTTTATGTTCTTGGATTTCTTTCATTA
TCTGCTTTTTAAACTGTTGGCATTCCTCTGGTGTAAGTGTGTCTGCTTTGGC
AATTAAAGGAATGATATTCACTTTTTCATGCAAACGTTTCATAAACTCAAT
ATCCAATGGTTTAAGTCCATGTCCTGAAGGAGCAATGAAGTATAAACAAC
ACTGCACCCTGTTATCAGGCATCTGTCGTCTGTTCACTCGAGATTCTGCAT
TTAAGTAATCTTCAAATTTACTATCAATGTAGTCGATAACAGGCTGCCAGC
AATTACTATTATCCACTGCATCTCCAAATCCCGGAGTATCAACTATCGTCA
GCAGCAACTGAACGCCACCTTCTTTGATTAAAACTTTGGATTGCTCCACCT
GAACAGTCTTTTTGATTCTATGAGAAGGTCCTGGATACTCTGGAGAATACA
AATCTGTGAGGAATAATGAGTTGATTAATGTCGACTTTCCCAGTCCAGATT
CACCTACTACCATAAGAGTGAATTCAAATCCTCTTTTCACCGATTTTCTGT
ACACTTGATTTGGGAGGTTGGCAAATCCCACATAGCCCTCAAGGTTCTTCG
GTTGAGCTACTTTGTAATGGAAACATTTCCATCATTCCATGGTGCTGCAGT
TGACGCTCCTCTCCTCAGCAGCAGCGGATCTCGCACTGACCGACATCCCCT
CCCCCTCCGCGACCAGCCCCGGCGCGTCGCAGCCGGCGGATTCTCCAAAA
CCTCCTACGCCGATTCCGCAGCGTAGCCGCCCAGCGTCACTGCCCCACTTA
CGCACACGGCAGCAGGCGGACTGCCCCACTGGCTCTCGCCCGCCCTGAGG
CTTCCGGGCCGAGCCGAGGCGGCTACGCTCTCGCGAGATTACCCACCTCC
GCGACCTGCCGCCCTGCTTCGTTCGCTCTGAGCGTGTTCTCCCTGGCGCCG
CCGCCGATTTGCTGTGCCTCCTCAACCGCCATGTTTACATTGGCCGCGTCT
GGGCACTTCCGTTGTCGTGGACAGTAGGGTCTTCAGTATTTTTTTTTCTCG
ACACTACCTCTACGGTTAACTGCGCTACTTTTGTCACTGAAAGTCCTGGGG
ATTTAGTGGGAGCGGTGACTGGGGCGCGAGGGCGGGGTCTTGCCCGAACT
AAAGATGGCCGCCGCCAGTGCCTCCCCGCGCAATTTCACCTCAGGTTCTG
GAGCAGCGGGAGCCGCGGGGTTGGCCCCCGCTTCTCCTTACGGAATTGGC
GTAAGGCTGGGAGGCGCTGGTTAAAGAGCCAGCCAGCTCTTGGAAAATTG
CCCTCAGGGTGGGGGCCCCCGGTTAGGCGGTTCTGCTGTGGAGCTGACGT
CTTTGTGAAAGAGAAGAGACAGGCAGGTGTCTGTTCTCCTAGTTCTCCTGG
CCACCCCATTTCCTCACTTCCGTGACTTTGTGGGTGGAAAGTGAGGTTAGG
CCCTGCTTGCCTGGACACTTCCTTCTTGTCATGTCTGGTGGTCTGGCGCAG
TCGAGAGACCGACTGCTGCAGTTTCATCACACTTGTTGACTCACCTTGGTC
CCTGCTCGCCCTCTGGTTGAGGAAATGAATGGACGCATGAGAAAGAAACT
CAGTAACAGTTGAGGATATCGGAAGGCGGGAGGTGTCGTCTTATACGGTC

CTTAGAACCTTCCCTCCCTAGTGAGAGTGTTCTTAAGTCGCTATGAATGAT
CCTTCTTTCGTCCCTGCTTGAAACTGATGGTTCTGTAAAGTTGGTGCACAA
GTTAAGACATTTTGTATAAAGTTACTGTGAGCCCTAAAGAAATGTTCGTAG
CAGCACAGCTCTGTCAGTGAAACAGTATCTACTAATGGAATCCCACATCG
TTTAAAAAGTTGGTACACAGACTACTATGTGAATTCAGTGAAGTACAGCT
ATCAGCATTTATGGAGAACGCAATACTGAGGGGGAAGCAGGTTGCAAAA
ATTATCTAGTATAAGTAATGTTTTTAGAAAGGTTTTATTTTTTAAATGAGA
AGCTGTGAAATCAAAGTGCAAAGGTCTCATAAATGATACATATCAAGTTT
AAAATAATGGCTACTTTTACTGAGGACCAGAGGGAGTGTAACAGGAAGCG
TTCCATTGTATCTTTTTATTTTCTGTCCAAGTAATCTGAAGAAAATGCGAC
AGCCAAAAGTTAAAATATGTTAAAATTAAGTATAGGGCACAAGGGTGTTC
AATTTCCTGTGTGCTTGAAATACTTCCAAGTAGCCTTTTTTAAAGAAAGAA
ATTTTATCTAAAAATCCAATTATATTGTATCTTGATTTATGCTGTTCCTACA
CCTACATTTTTACATTTATCAATGTATCCCACCTTAGCTGCCAACCAATTAT
TGTGTTAAAGTTCTTTCATTAAGTTTCTGTCTCAAAAAAGGCAATATAGCC
TGCCATTGGGCCGGTAGGGCACTACCTTTATCCCAGCATTCAGGAGACAG
AGGCAGGCAGATCTCTGAGTTCGAGGTCAGCCTGGTCTACAGAGTGAATT
CCAGGACAACCAGAGCTATACTGAGGGGGGAGGGGGTCGAATAAAAGAG
CAGTATAGAAAATACAAGGTCTAGGAAGTTAGAGTGTTTGGGTTCAAATC
CCAGCTCACATTATTAGGCACGTACGTGGCCAAGGACAGAAAAATTTCTT
AACTTTCTATGATCAAGTTTCTTCCTCTACAAGTGTCTGAATTCAGTACAC
TAATGCATGCCCATTGCCTAGTAGAAGCTTACTGATATTTCTTACATGTAG
TTAATTTATCATGATGAATTTATAGTGCAAAATTAAAATATTTTTATATAA
TTTAATAGTCTATAAAGAAAATATCTATCTTTGTAAGTAAATTTCTTTGAA
GACAAGCTTTTAGTAGGATCTGAATCTTAAGAGATTAGTGAATTGTGAGC
TTTGTCATTTCCTGGAAATGGATAAATTCTCAATATAAATAATGTCAAAGG
TACATTTGTGCTGTCATACTAAGTTCACCCCAAATAATAACGTCAGTGTGT
TCTATTGAAAATGCATGGTAAATTACAGGAAATGAGTTTATACCATGTAG
AGTTTTCTCAGAAAGAGATTCAGTTTCCAATTATGCTCATTTTATGATCTG
TTGTGTGTCCTTGTTAACAAAATAATGCCTAGACGTTAACCTAATTCAGAG
AGAGTCTTGAAAAAGAAATTTCTGGGCTTAGCATGCAGGAAATTTCCAAT
ATATATTTACAATGTACACATCCAGTAAACTACCAGCTTCATCAAGTGCTT
ATTTCCCTAGTCAGCTCTTCTTTCCACTTTCAACAACAACCTCCTCAGAATT
TCTGCATTATTTTCAAGTCATCTTTCACTTATTTTCAGAAAAAAAGAAAAAA
ATATATAAACCAATGGATTGTTTCCTCATTCACAAGCCTGCCTGCACAGGC
ATCCGACTTGGCACCAGGAACTGGATACTCCCTTACCCCCTACCATCTCCG
AAGCACATATTTCTGTCTCTTCAGGAACTTCTCTCAACAGATATTTTATTCC
ACTCAATAAGCTTCAGGATTTTATCCCTATCACTCTACCTTAGGTCATCAG
TACTCAGTTTTTCGATAGAGTGGACCTTTTTTTCTTTATTAAATCAACTTCT
CTGCAGTATTAGATAGTAGTAATCATGGTTTTCTGAATATATATCATTTGA
TTTGTAAAACTACACTTTCCTGGTTTTCCTCCTAGATCTCTACCTTTTTGTT
TGTTTGTTTGTTTGTTTGTTTGTTTGTTTTAATCTTTCACTCTAGGATCATCT
GCCTATCGCTTAACACCTAGAAGTTGTTCAAATTGATCTGTATTTTCACTTT
ATATATAGGACTTGTTCCTATTTTCACTTTGTGTGTGTGTGTGTGTGTGTGT

GTGTGCGTGCGCGCGCACTATGTATTATATGTTTATTAATCATGTGTGTGC
ATGACACCCAAAGAGGCCAGAAGAGGCCATCACATCCCTGAGACTGTAGT
GCCAAAGATTAGGAATTGCCATGAGTGCTGAGAATGGAAAGTAGGTCCTC
TGGAAGATCAGCCTGAAACCGTAACTACTGAGGCATCTCACCAGATCCCT
GTCTCATGATTTAAAAAGTCATTTATTAGTAGTATATTACTGTGTATATGT
GCATATAACATATGTGCAGAAGTCAGAGGACTTTGAGGAATCTGTTCTAT
CATTTCACCATTGATTCCATCCCTGAAACTCATAAGGCTTCCCAGCAAGTG
TTTTCATCCCCTGCACCATCCCGAATCCCATTTCACCTTTAACAGTCCTCTT
GTGTCTTGTTGGCTTCTGAGATCATATGTGTGCTGATGACTCCTAAGTATA
AGTTTCAAGTCTACTTCTCCCTTTTAAGAATAAAGTTCACATACCTTTAAA
ATGTCTATCCTACATAGTCCAAGGTATTTCAAAATAACATCTGAAACTAAA
CTTCTCATTTCATTTTGTCTATGTTTGCTTCAGATCTCTAGATGGTATCATC
ATCATTCCAGTGACTATTGCTTCATGATTTATAACTCATTCTTCTATATCCA
TAGTGACCATTCTAAGCCTTAACTAGATCAGTCTGTTTAGTCTTTATACCA
GAAGGTGTACATGATATATTTCCTCATTTCTGGCCTGCTCACTGTTTCTCTC
TCTCTAGACTCTAGCAAGACTTGCTGACCCTCTGTCTGTAATACTCTAACA
TTCCATTTCCTTTTCTGCACTCATGCTTTTTTTATTTTTCCTTTCAGTATCAG
TTTGACCATTATTTCTCTAAAAGTCTTTTTCTGATCTCCCATGATTGGGTGA
CATGCCCTCTAAACTTCTATAGTTTCTTGTATTTACTTTCAAATTGTGCTAA
TAGTACATTTTTGTAAATATTTGTCTCCCTTCCAGTGGAGTACAAACTCTTT
AAGAGCAGGGATTCTTGGTAACATAGATAGCCCCACATAGGGTACAGTGT
TCACTACTTAATTCGGCAGCTATTTCCTGAGGGATAATTGTGTAGTAGGCA
CTAATATAGTACTACATGTTGAATGAGGAATCCCAACAGTGAGTAGGGAA
CTGTGTCTCTGACAATGTGGGAGCTGGAGGTCAATATTTTAACATACTAAT
ACTGACTTTTAAAGGCAATATTAAACTGATTATGAGGCTTTATTCTCTATT
AAATCTGTTTCTCATAAAAATACACTAGTATTTTTCAGTATGATAATTGTA
ACACGTTAAATGCAATAAATAAATAAATAATTAGCTGTTTCTTATCAAGCT
CGACATTTTTAGCTGCCAAAATATAAAGCAGTGATGTCCCAAACTAATTTT
TATTGCTTTGAAGATACAGATATTTTTCATAAGCTGTGCTTTGTGTTGACA
TAGATTATTATATTTTAATGTATTTTAGATATTTGTGAATTGAATTAGAAA
ATAATCTAAACTTTCTCGGTTTTAATTTCAAATAAAGTAGCTCGGGGTTGG
GGATTTAGCTCAGTGGTAGAGCGCTTGACTAGCAAGCGCAAGGCCCCGGG
TTCGGTCCCCAGCTCCGGAAAAAAAAGAAAAAAAAAAAAAAAACAAATC
AAATGAAGTAGCTCATGTATTCCACATAGGCAGTATACCTTAGCATCCTCA
GTGACTTGAGTAATAGCAAGGTGCTGTGTGAGGAAACATGTGAGCCCTGC
TGCAGAGGCATAGGTAATGCATGCCAGGTGTCTGTTGTTCACCAGTGACTT
CCACTTTACCAGCACTCCAAGACAACTACTTAGGACACTTTGAAATGCTGT
GCTTCTCTGTGTAGTCTAGGCTGATCTTCGACTAACAATCCTCCTGCCTCA
TTCTTGCTAGTGCTGAAATGGCACATGTGCTTCCCCACACCTAGTGCATAG
GAATTCTCTCCCTCTCTCTCCCCCCTCTACCTTCTTTTCCCTTGCCCTCCCA
AGACTCTATCCCTCTGGAACCAAGAGAAAAGAAAGGAAAATAGAAAAAG
AGACGTCCTTAAGCTGCTTTTGTCAGTCTGTTTTATCACAGCATCATCAAC
AACAAAAAACTAATGTGTGTACCGTCCAGACAATCACTTTAAGTTTTCATG
AAATCTGAGTGTAGATTAAATAATTGTAATTAAATAGCATAGTGCCAGAA

GAAAGTCGGGGTGAGCTCAGACAAGCTTATTAAAATAATTGAAAATTTGT
GGTGTTTGACAAACAAAAGGGAATAGATACTGATTTATCCCTATATGGAT
TAAGAATCTGAAGTACAAGATGGGACATTTTTGAGGTTAATGAACAAAAA
ATAGAGAAAAATAAAACCCCATACTTTAAAGATGAAACATTGTAGAAGA
AAAGCCAAAATACACAGCAAGAAGAGAGAGGGAAGGAAAAGCAGGGAG
ATTTCAGCTTCATCCCAGAGAGTTGAAAAAATGGAAAAAATATCAGAACA
ACCTCCATTCTTAACCTCTACCTATTTTTCCCACCAGCCAAGAAGAGAGTG
GGTAAAATCATAGCACAGAAATAGGATGTCCCAGAAGTCCAAATTTATCT
CAATTTATCACTGAAGTCTCAAGGAATGTTCACATCCCAGCCTTTTTTACA
AACGTATTTCTCATACATATGAGAATCCCTCTGTTCCTTGAGGGCCCCACA
TCCCTGGCAATAGACTGGCAGTGCAGTTGCCTCTGGGACAGGAACGAGAA
TAAAGCACTTACCTTGTTTTTCATTATGATTTGTTTGACATCAGTACCTTGT
TTTAGAGCTATCAAGGAAAAAAATCCAAAGTTTATAAAATCTATTGCAAG
TGGAAAAGGCCATTGCTGCCAACTCCCTTTGGCATTTCCTACCACTGAATC
AGAAATTGACTGAAATAGGAAATAGGGACACATGGTTCTATCTATGGGAA
ACCAGCTGCCTGCAGCAACTAGGCTCAATATATTGACTTTGGCAATGGCT
GAACACTTCCTGCTAAATAATACAAAAGTGAACTGAACTTAACTAAGTTT
ATAAGGATGTAATAGGTTAGCAGTTGACCATCATTTTATAAGCAGTAGGC
ACTAGCCCTGTTCTTTTCTAGCTGACCTATTACATCCCTATAAACCATGATT
AGTGACTGATGAGTTTTCTGAAGGTATACCTGAGAGAATAAGGGGAAATT
TAAAGTGGAAGCAAATGCCTTGACATTTTTGTTAGTCTAGTCTGTTTCTTTT
AAGTGAGCAATTGCATTACAGTACCATGGAGACTGAGACAAGTCAAACTG
TTAGTAACAATATTGTGTCACTGCTGCCCTTTTTTGTGAGGCTCCTAACAA
CTTCAGAGACTGTGTTGTGTGTATGTGTAAGCACATACATATATGTGTTTT
CATGTGAGTCCACATACATGTGTGTATATGTACAAAGACCAGAAGTCAAT
AGGTGTCTTCCTCAGTCTTTTCTCCATCTTATTTTGTTGAGGCTGAATCAAG
TGGCTCCACTGGCAATGAGAAGAAGCCCCAGAGACTCTCTGTCTCAGCAT
CTCCAGCACTGAGATTGCAGGCATATGGACCATGCCTCATTTTTGTTTTTG
TTTTCTTGAGACAGGGTTTCTCTGTGGAACCCGGACTGTTCTGGAGTTCAC
TCTGTAGACCAGACTGGCCTCAAACTCACAGAGATCTGTATGCCTCTTGCT
GGGATTAAAGGTGTATTGCCATTACCACCTGGCCCAAATCTGTGTTTTTAG
TGTGGGTTCCTCACATTTTCATGACAAGCACTTTACCAACTAAGCCATCTC
TCCAGCCCCCCAACTTAGAGACTTACTGTATCATTAGCCATTTTGTGTTGA
TTGGACTCTACTCTAGAGAGGTACTTAAGCACCTGACAAAAAGTAAGAGG
CAAGTAATAGTATTCATGATTGCTACCCATAATGTGATTTCATTCCATGAA
TCAAATGATTCCATGAATCAAATGACTTCTCTTGTTTATCTTAATACTTTTT
CGTATTTTATTTGTTCCAGAAAATTTGTAGTAAGTAATAACATTATGCATT
GAACACTAAAGATTGAAGAGACTTCATGAAATGAAAATAATGACCATCTT
TTTCATCCAAAATGATAGTAATATCACAGTTGACAGTAGTGCTTGCTTATC
TATTCAAGTTCCTGACCCCAAAGGCTCTCTCTATGTGTGTCCTTCACTTAGT
CTCAGAGTTAGAAGGTTAGAGGACCTCATACTCTCAATCAGCTACCCACC
AAGTGCAGAACTGCCTTTTCATAGCTCATATCCTGCCTACTATTGAAGTTA
TGAGTGTGAGCATTTAAGGGAAGCAGCTGTATCGCAGATGATGATATTAC
TGTAGCTCCAGAGTGCACATATGCCACGGGTGTAACTCCCTTTCCCTTTCA

TGATTCTCAACACACAAACGAACACTTCTGGAATTAGTTTCTGATAAAAGT
ATTCATTTAATTGTTATTGTCTCAAAATAATCTTGGGCTTTGAGCTAAATA
TAAGTGATTTAAATTTTTCCCAGTGGCCACTAGCTTTAAAATCTAGGTGTC
TGCAAATTGTTGAGTAGATCTCTAGATGCTGAGATAGATTAAATCTTAACT
ATCTGATATAGAACTTAGTGTCTCATACAGGGCCTAATATAGCACTCAAGT
TTATTGGGAATAATTGCATATATTTTGAGGTTCTTATGCGACTTAGTAAAC
TAAATAAAATGGAAATGGCAATATCAAAAAATACAAAGAACAAAAAAGT
AGAACATGAATATTACAAATAAGGAGATTATCTTTGGGTTTGCTTCTTAAA
TTGTTCCCAACTTCCTTTGCACATACATGCAAAAACATTAGAGAAAAAAT
AAACACAAACAAGAGAATAGTCCTTTGGTAAATAGTCTTCCTGTGAATTTT
GCATGACAACTCTCTCTTCTCTTCTCTTCTCTTCTCTTTTCTCTTTCTGTTCT
GTGACACTGAAATGAAAGGCTCAGTTGATCACATTGGGAGTGTGCTAACC
TGCCTTACTTTAGTCAGGTCTTCTAGGCCATGCTTCTCCTGATCACTTAGTT
GAGTCAGTTCTGACTCAGCTCCCATGGTTTACTAATCTGATAGTAAAGAGA
CTTACCAGGCCAAAATGGGAAGAAAACAAATGAGAAAGATCAGAAAAGG
AAAATCCTCTTTAGACCCCTGACAGACTCTCCTCTGCTTTTATTCTCAACAT
GCAAAAATATCCAGCAAAGCAGCCTTGATTTTACAAGTAAATTGTTAAAG
GAAAAGAAATAGGCCAACCATTCCAGCTACCTTGGCTATGAGTTCTGGGT
GAGTCACTTCTTTAGGAAATGAAAAGAATGTCAAGTATGTGAAGGTTGCT
GATGAGGCTGCAGGCCGTAATTTCAGGTTTCCTTCCTGCTTCTTGAGGAAG
CACACACATCAAATTTACTGAGGGTTGGGAAAACAGAGTGGTCAAGAGG
GACTCGGTGAAAGGGTAGATGCCTTTTAGCTACTGGGTTAGTCCAGGTTC
ATTTAATTATGTGACCCAGAAGGAGTGTCTGCCTGGTAGCGCAGGCCTAT
AATACTAGTTATGTGGAAGCTGTCACACGAGGATGTCTGAACAGCTTAGC
GGGAATTTGTCTCCAAAAATAATCAAAATAAAAGTCCTAGAAGGGACAGA
GAAGTGGCTCAGTGGCAAGACAGCTTGCCTGGCATATGCAATCTCTAAGT
GCAAGCCTTAGTATTACAGAACAACATATTTAATAAGCTCAAAATGATCT
ACTAAAGGGAGACGTGTTTTCCCATCAATCCAAGAAAGAAATATTTGAGA
AAATTTGAGGGGAAGAAATTATTGCTTTTACTTTTACTATTTTATTGTGAA
AAAGTAGAAAATGATTCAGTGGAAAATATTTTGACCTGTAGTTTTTTGGCA
AAATGTGTTACAACAAAGCACAAAACAAAACAAAACAAAACAAAAAGAC
CAAAGATGTGGCCATAGAAATGTCTCAGCAGGTAAGAGTGCAAGTCAGAT
AACTGATCCATATAAATGGTGGGGATTGGGGGGTGAAACTCTACAAATTG
TCTTCTAATTTCCATTCATGTGCTGTGATACGTAATTAGCAATATATACTA
AGTACACATACATTCTAGTCAAAACAAAAACCCTAAAAGTATAAATAATT
ATTTTTCATAAACCTCCCAAAATCCTTGGTTTCCCCAAGGTTCAGAGTAAA
CAATCTGATTAAATAAACAGCAACAAGGCAGGGCAATACAAGCAGTGAG
AGATGGATAGGCCAGAGGTGCAGAGAGAGGGACTACCTCAGCTTCTTAAC
ACTGGGCTCAGCAAAGCAGGTGACACAGCCTTTAGTGACTTTCACAGTCT
GGGAGGGTTGCCATGATGAAATATCATACCACAATGTGAATGTTGTCCAT
GAATCTATTTTTTACAGTTGTAGAAGCTAAAAGGCCAAGATGAATTGTGTT
AATAAGCTAGCTTTTCCTGAAGCTTCTCGCCTTGGTTTACAGATGCCTGAC
TTTTCCGTGTCTCTTTGCTCTGCGTGTGCACGTTGTTGATGTCTTTGTACAT
CCAAACATCTTTGTCTTATTAGGATACCAGTCAGACAGGGTTAGGACCCCT

TTAACAACATGTTTCTTAATAACTTAATCTCCATTTTAAAGGCTCTGTCTTG
AAATACATTCTCACCCTGAAGCACCAGGTAAATTAAAAGATTGTAGTTCTT
GAGGGACACAACTCATGCTGTAACATTCTCCATTCAAGTTAACAAAGGCC
GGTCAGTACAAGTACTTACCAATTTTCTAGAAATTTGTCCTAGAAAAAAAT
ATCTAGTACACATGCCAAAATATGAGTACATACTTAAGAATGTTCACTAC
CCCCTTGAGTCAAAGAGCAAAAAATAATAAAGCGACCAATTATTCATTAA
CAACATGTTTATATCATTAAATTAGAATCCAACTGAGGAAATAGAACAGT
GAGTCTATATACACTGCTAAGTCAAATTTAAAATTGCAGAACACTATATA
ATGTACACTATGATTTAAAATTAAATATTCGAACATACCTAGCTAAACGTC
ACCCTGTCAAGAATGACTACCTCCAAGGTATAAACTTTGGGATGGAGCCT
TGTACTTTTTATCTGTCCATTTGTGTAGTTGGAATACCTCAATAGACATGTT
GTTTTGAATTAAAAGTGAAATTGATAATTCTTAAATTCTCCTCTACACACT
TAGGTAGATAGGATTCCAGTCTCTAAAGTTTGACAATACATTTTCAAAGCT
TCATTTATTGAGCATCAAGTAGCTCCCCATCAACATCTTTGAGCACTTTCC
CAGATAGTATACTTGAATTCTTGGAAGAACTTCTCTAGGCAAGTGTTATTA
TTGCTGCTCTATCGTAGTACACCGTGGAAAGCACATCGTAGGAGAGTCAG
GTGATTTTCAAAATTTGCAGAGAATAAACCACAAAGTCAACTCTTGTTGG
CTTTGTACTGTTGACTTACAAAATTACTTCTGTTTTATATACAGGATATAG
AAAACTATCCAAAGTTTGACATTATCTTAGAATATCATTTACTGGTCTCTA
TTCCCTCAGCAAACATCACTTCTAAATCTAAAAGTATGGAAGTACTTAAAT
CTATGTGGCAGCCACTAGTCGGATGCATTTGTTAGAGCCATAAGGAGCTC
AACAGTAATTGGTGAGTAGAAAAAGTCCATTGTCTTGAGTTCCTGTGGCA
CTAAAATAAAGAGTACGAGGAGAGATGTTTTGTGTACCCCTTATTGTAGG
CAGGACAGACTTTCCTTTGGAATACTAACATAGTAGTAGTGAGGTCTGTA
ACCTCACTTATCAAAAGAAAGTGGCTTTGACGGATTGTGAACGTTAATGA
CCATCTTTGTTTAAATGTACACATACTCATTGTTCCCAGTGAACACTGAGG
ACTTTGTACAGTGGCAGGAAGAGTACTTGAAGAGAACCTGAAGCAGCAG
AACCCTTGGTCCCTTTATTTCCTGAGATCTTATCTCTCTCCTACAGCACTTT
GCCTCCAGTTTATCCCAAAGTGTGACTTCAGGAATTCAGCATATCTGTCAA
ACCCTTATGCCCCTGTCCTGATACTACTTTCGTTCATTCAAGTTCCCAATCT
TTGTTCAAAATCAATGGTTCCCAAAGCACAGCCCTTTTGATTCTGTCAACA
GACTATTGTTTGATGTGAAGGCAGTTGAAGAATATTAGGTAGCCCACCCA
AACACACAACCTGATTACTGGAGGAGAAACAGAACTAGATAGCCATGGA
GGGCATAAAGACAAACTTCCCAACGTTGGCTGAGATATGATTCTGCAAAT
AATGGGAGCCTTCACAGTCATAAGTTAATGGGAGCTTATTTCACCCCTCAT
GAATATGGTATGGACACTCTAAGCTGCCTGGTATCTCCTGTAAGAGGAGC
CCCTGGGTGATATGAAAGGGAATATAATGGTTACTACTTGACGACAATCC
AAAATTGGAGGGCTCACCTGTGATCCAGATCTAAGGCTGGAAGATACAAA
TTTCTGACCTGGATCTTGGCATGGAGATCTTAAGGCATAGTGGCCATAAA
AAGCTTAGGCCCAGGCAATGTAGTACACACCTTTAATCCTAGGAGACTGA
GGCAAGGAGATCTCTGAGTTCAAGGTCAGCCTAGGACAAGCAAGTCCCAG
ATCCAGGCTTTAATCTGGGGACCTTCTGCTGGAGATCCACATAAGGACATT
GGAAGAAGGAAGATTCACTCTTTTTTGCCTGCTTGCCAGCACATCTGTTAG
AACCTACTTCTATAGGAGACCAACTGAAACAACTAGCTTCATGGGACCGA

GAAACTACTAGATTCTTGGACTTCCCCTCCACAGCTGCCCATTGTTGGGTT
ACTTGGACTACAGACAGTAAGTCATCATAATAAATTCCCTTAATATATAG
AGTCGTTTCATAAGTTCTGTGACTAGGGAACCCTGACTAATACAGGTAAT
GACTGAATAACTGGAAGAGAGGTTTAAGAAGGGGAGAGGGATGAGAGGT
TGGAGGAGCTGAAGAGACAACTAACACTAAGGATGTTTGAAAAGGCTGA
TTGGAAACCTACTATTTTATAAGCTTCCTAAAAAGTAGAACTTGATGTTAA
GACTCCATTGATGAAAATGTCACAGGACATGGGGATATCAAGTTGATACT
GACTTAGAAATCCCACCCACCCATCCCTCACAGTCCCCCACCCCCAAGCCC
TCCTCCACTGGCTAGCATTCATCATACTAGAAGGTGCCATGCAGGCTGCTG
AAGAAGAAAAGCCATAAATGTGTTATTCAGCTGTGAACCCTTTCAGCTAT
AACAACAACCAACATGGTGAGATATACCATGGACAAAATAGTAGCACTTA
TTAAGGCCTGCTCCACAGAAGGAAATACATACCTAATGCTGTAAATCTGG
CCAAGAACCTGTTAAAAGCTCAGAAGCCCCAGCATGGAATCTACTATTTT
CTTGTTAAATGGGCATAGTATCAAACTGCCTCTAAATTCCTCTCTCTGTAC
CCATGGATTAGTGCTGCTCTCAGGACTCAGAAATTTTTGTGCAGTGGAAA
GCAGTTCTTGCAGAAACTCACAACTGGTCGAAGCACAGACAATAAAAGCC
TACGAAGTGCTCAGCTGTAGGTGGGTCATCTATGTCATAATCTCTTCCCTG
AGACTCAGGGTCTATTAAGACAAAAGGGAATGGAAAGACCGTAAAGCCA
TAGGTTGGAGAGGACTGGAGCTAAGCAGTGTTATTTGGGTATGGAAGCAC
TGTTGCACTGAAAAACCTCACAGCAGCTGTAACTGGCTGCTGCCCAGGAC
TGCACATCAAGCCAGTCAGCATTCCAGCATGAAGAGGGAAGGGACGCAC
AAGCCCCCACCTCTAGTGGAGAGCTATCTACGTTTGATGACTTTTAGGACA
ATTGTTTTTCCTTAAAGGAAAGAAAGACGGTCAAACAAGTTCCAGCAGAT
GCCCCCACATTCATGAATATATAGGTAGCACACAGTGGTCATGATGGTAA
AAGAGAGAGAAAAGACATAAAATTGACATGGCTGCTAGGGCTGGAGAGA
TGGCTCAGTGGTTAAGAGCACTGACTGTACTTCCAGAAGTCCTGAGTTCA
ATTCCCAGCAACCACATGGTGGCTCACAACCATCTGTAATGGGATCTGAT
GTCCTCCTCAATGTGTCTAAAGACTCTACAGTGTATTCATATAAATAAAAA
TTAATAGAAAAGTTTAAAAATTGACAGGGATGAAGGAGCAGGGCGTGAA
TACAAACAAAATACACTGTGTGCATGTATGACATTCGCAAAGAATTAATT
AAAATGTTTTAAAAAAGACAAGCCCCTCATGCTTAAATGTTTTCCCAGCTA
TCTATGCAGGAGTCTGACCTGCTTTTAAAGGCAACATGAAGGGGGGAAGA
CAGAACAAGACATCATTAGAAGGAGTTGTTAGTCACTTCTTAGCCATCTG
ACTTTGAACACATCAGCTCCTGTCTCACAGAACTGTCATGAATATGGAATG
AGATTACACAAGTAAAGAGCTTTACATAGTCTCTCTCTCTCTCTCTCTCTCT
CTCTCTCTC
>LNC_010954
CACAGTTAAAAGCTCTTCCCCTAGTCCTCTGTCCCATGGGCAAGGCTAGCT
GGCTAGATAGTGGGTGTATTCCCCACTGGCATGAGGGAATACACACAGGT
CAGGGGCCTGAGCCATCCCATGATGGGCAGAGGAGGACAAGGCCCTGTA
CAATGGTCCCTCTCTGCCCCCTTTAGCCTAGCACCCCACCCTGGGGACATT
TAGAATGGTGTCAAGTCATAGCAGAGTAGCGTGGCCTGGGTCATCATGAT
CCACACTGTTGAGGATGGCCGTCTGGTCTTCTGGAAGCTGACGGTGGCAC
AAGTCTGAAAGCCGGGCAAAGGGATCAGGACGAGAGTGTCTCTTCTTCTT

TCTGAGGCAGACAGCTTCGTCGGGCCACAGAACCTGAGAGTTGGGAAGCT
GCTGATCCTGGGAGGCAGAACCGTCGGATCGTTTCTTTGACTTCGAAGAG
CCCTTGGATGACTTTTTGGGCTTCTTTATCCGTTGGTATCTCAGAGCCTCCA
GCCTCTCAGGCACAGCAGCGTTCCCG
>LNC_010988
CAGGAGGCAAGGGAATAGCGTGGGCTGGGGTACCCCAGAGTCCCGTTGG
ACCTTCTGCACACAGAAGCACTCAGTCAAACCATCCAGTTTGGGGACCTT
GGAAGAGCGTAGGAGAGACAGACACTAGGACAGACTCCAGAGGCTTTGT
TTTATTGTGCAGTTCTCTTCCTTGGTGAGGTGCCGTAAGCTGTGGTGGTAT
TACACATGGTGATGGGAGAGGTAGAGCTGCCCGTGGGGAAGAAGATGCTT
GGAAAAACTATCTTCCAAAAAGTGTGCACACTAGACCTGCCCCTGAGACT
AGGAGGTCCTGTAAGGGGCAGGTTGCAAGACTCTGGTTGTCTGGGGAATC
AGGGACAGCAAGAGGGGCTGGCAGAGAGGGGCAAGGGAAAGAACAAAA
AATCTCCATTAGCAAGTTGGGTCCAACTGGTTGGTTCTTGGACCCCCCTTT
CCTGCACTGATTTAGATCCTGGAGTCAGAGTTCTGGCTCCTTGGAGGCAGA
GAAGGGGGACTGGTCTGCTACCCTCACTGTAGGCAGTGTGGCCTTTGGAA
ACAGCAGGATCATGTTTTAGAGGGGGGGGGTGATCTGTCCCTGACCTCCT
ACCCCTTTCCCCAACATTCCCTGGCTGGACCTGCATCTTTATTGAGTGTGC
CCTGGCCCTTCTTCGGTGATAGAAGGGGGCTGAGAATGACCTGGTACAAT
CTAAGTTCTCATCCTTATTGTTTGGTCCACAAAGGCTCAGCAGCGCTCTCC
TATCTTGAGGCCCCTCCCCCTCCCTGCCTACTGGGCGATACTTTCAACAGA
GTATCACTATACCAGGCACAGACACAGTCCAACTTCCCTCATCCATATCTC
TTTCAGGCATCTGGCTTAGAAGGCAAAGAGGAACTCACCCGGGGGGGTGA
TATCCTGCCTCTTCACTGCCCATCATACACCCTGGTGTTCCATCGGGTCTCC
AGGACAAACTAATCAGAAGCAGCCCCGGAATACAGAGTACAAAATGGCA
GCTCGTGGTGTGCACTGAGGGTGAAGTGGCGGGGGTGAGGGTGGGGGTG
ATGGCATGCAGCACTGGGGTTCCCTAGGAGGGACAGGGTCCCCATTTCTC
CCCGCGTTTTGAGATATTTGAAGGTCTTGGGGAGGGAGTCAGCTGACCGG
GAGAAGCAAGACCTCTTCAGCAGACCTTGAGGTTTCCTGTTTCTCCTTGGG
CCCAACCAGTCACAGAGAAATGAAGTCCGTGGCTTGGAGGAAGGAGAGG
GAAAGCAGGAGCAGCAGCAGCCAGGAAGTGTTTTGAACCAGCAGGCTTA
TGCCACCACACTTGACCAGCTTGTCTCTGATCACATTGATAGTTTTATTGG
AGCTTGTGGGATTCTGGCCCGAGACTCGAAGTTCACACATGTAGTCGCCCT
CATCCTTGGTGGTGAAGTTGGCTAGAGTAAGGACCTTGATAAAGCGGTCA
CTGAAAAGGTTGACGCGGGAGCGGTAAGTGTGCTCGGGAACCCCCAGGGT
GCCTGACAGCACGTGCTTCTTCTTCTCTCGGGTCAGGCTGAACTCATGCTG
GATGGGCAAGTTGGTGTTATTCTCATGACGGCAGTCCAGTCGAAGGTTCT
GGTTCACCAGGCAGGCTGTCAGGCTGATCACCCTCTGTCCTCGGGACATCT
GCAAGACTGAAAGCAGGAGAGTGATGCTGATGACTGGGTTCATGGTGCCA
ATAGCTCCGTCCTGGGTCTGGGCTCCCCTAGCTTGCAGCCTCCTCTTGGTG
CACCAACGCCTCTGGTTGCTGCACCCAGCGCGAAGCCCGCAGTTTT
>LNC_011005
CCATTGTTGAAGTCTGTCACCTTTCCAGAAGCTCCTGCCTTCAAGGATGGA
GAAGACATCCTGGCCCTCAGGAAGTCCCCACCATACCCTTTCCTGTTTCTT

AGGTGGTCTCTAGCCTCTACCAGGATGTCCAGCTGGTCCCCTACACAGTAT
GTTCTTGAGGGTTGAGGATGGTGGCTATGCTGTGTGTAGCACTTGTGGTGT
TGTTGAGGTGGGCGAAGGGTCTGGGAGGGATCTGTTTGTTTAGTTTCTCTA
GGATCTCCCTGATTCTCAGCTCTGTCTCTGTTGGTGAAACTGATGGATTGA
GAGGCACTTTAGGGCATGAGGATTTCATGATCAAGTCCCATTGATTGA
>LNC_011017
ATAAAAAGTACTTTTTCTTTTGGTTTTAGGATCTTTGTCTGTAGACAGAGTT
GAGGTTCTGGGTCGGCACAGAGCTGTTTGCCATCTTTCAGCTCCTGCTGAA
TGAAGTTCCGGTAGCGCTCGGGATCATTTTCTGCCAAATCATCGAGCATGC
TCCAGAACTGGGAGATGTGGGTGAGAAGACCCTTTGAGGAAGCCTTCATG
ATGGTGATGACTCAGGCCTTT
>LNC_011093
CAGGGTTTCCTGGAAATCCCGCTTGTCATCAATGTCATCAACCTTGTATGA
CCCTGAGAGGCTCAGGTAATAATAGTAGTCCATGCTGGTGATACCAAGGC
TTTGCTTCTGCTCTGGAGAGGCGCCCTCGATGAGCTGGTAGAATATGTGGA
AGCTCCGTTCTCCCGGGTTCCTCATCACCACTCTGGACTTTTCCAGAAGGA
AGTTGGAGATCTTGCCACCATCTGGTTCCCCACCTGGACTGAACTGGATTT
CAAAGTATTTTCCAAATCGGCTGGAGTTGTTGTTGCGGACTGTCTTGGCAT
TCCCGAAGGCCTCCAGGAGTGGGTTGGACTGAAGGATGATGTCCTTGACA
TGCTGGACCTTGGGGCCTCCTCCGGACACTCTGGAGACGTAGCTCATGAT
GTACTTGGCAGCTACTGTCTTTCCAGCACCACTTTCACCACTGATGATGAC
ACACTGGTTCTCTCTGTCGATGATCATGTTTCTGTACATACTGTCTGCAAG
GGCATAGATATGAGGTGGGTTCTCATACTGGGCCGCTCCTTGGTACATTTC
AATCTCTTTTTCTCCAAAATATGGCATTTGCTTGAAAGGGTTGACAGAGAT
TAAGACAGACCCTATATATGTAAAAATATAGTCGTCCATGTATCTCTTCTT
CAGGTTCTCTACGATGGAGCTCTCTGTGATCTTGGAGAGTAGTACCATGTC
GTCCACACCACTGTGCTTGACATTGTGGCTCTGCCAATGGTACCGGTAGGC
GCCTTTGCTGCCCATGGTGACTCCCGCCGCGGTCGCGCCCCCGCCGGGTCC
GGCTGCCTAGGCACTAGGGTTGGAACGCGGTCTCCTGGGCGAACTTCAAA
AGTTGTTCCCCTTCGCCAAAAAACAATCTCCAGACACCCAAGCACTCACA
GGAGCCAATGGAAACCCAGAAGGGACTCCATCCAGGCGGGATCCAAGAC
TCTGGGTGAAGGCAAAACTTAGCAGACCCGGTGCACACTCCGACCTGGCG
ATCCCCAACTGTCTAGAGATGCACCCTGATGCGCTCTGCAGGACTGCCTGT
CCAGCTGCCTTGCCTGGACCTCCTTTCCCTTAACACTTAATCCAAACTCTTC
TCTGGCTCAGGCTGGGCTCGGGCCGGGCAATCTGTCCATAAATGGAGAAT
TCAGCACAGGCCTGACCAATCAGAGTTTGTTTTCGCAGCGTCACTCCTCAG
AGAAGCCGAGGTAGCCAACAAGCGTGAAACTACAAGAGTTTCCAAAGGC
CTCTGGATCTTGTAGTCCTGCGGCCTCGCTACCCTGCAGAGCACGCTGCTG
TCCCATCGGTCCCCAAGGAGCCACCCCCAAACCGAAAGGATGAAAACACT
TGGGCATTGCTACTATACTTCTCATTATTTTCCGATGCTGGGGATCAAGCT
GACAGTACTCGAGATGAAAGTCAAGCATTCTACCACTGACCGACACACCC
TCCTCACTGCAGCGTCCAGAAGGAGGGAATACTTCTATGAATATCACTCC
ATCTCCTAATGCAGAGGTAAGCTCTTGGACCCACTCTAGGTAGAAAACAG
ATTTATTTATATGTATGGGTGCCTGTGGAGGCCGGGAAAGGGAGCTAAAG

CCCTGGAGCTGGAGTCCCAGGCAGTCGTGGGCCACCTGACCTGGAAGCTG
GGATCCGAACTCAGGTCTCTGGGAGAGCAGCAAAGCTTTTAACTGCTGAG
CCTTCTCCCCAGCCTCTCTGTGGCTACATCTTGCTCTTTCTTTTT
>LNC_011103
GACGGACCTCTCTCGACCTCCCACTGCTGACCTCTAGCACAGCACTCCTGA
GCTCACCGGACAGTTCTAAGTGCTTTCTGTTGGGAATGAAACATGGAATG
CCTTGTCGTTCAGCTTCCTTCCTGTCCGCCTGCCAGGTCTGCTGCTGACTCT
GCTCTACGAGGGCTACTTCCTCCAGGACTGCCACCTGTCCCACCAGATACT
GCGTCACTGGTGCTGGCTGAAGGGTCTTCACCACTATGGCAGACCTCTGTG
GCTTCAAACTCCAGGACTTCTGATGGCGTCAAAGGTTCCAAACGAACGCT
GTCTTGCTCACTAGCATCAGAATTCACAGATTCAGGTTCCTCCCCCTGCCT
CAGACACTGACCAAGGCCAACACCTTCCGGTGTTAGCTGTAAATCCTGAC
TAATCTCTGAAGACAACTCAGCTTCAGTGGGACTGTGCTCTTTACTCTGAT
CATCTGTGCTTTCTGAACTTTGGTCCCTCTTCAGCTCCATTTCCTGACAGGT
GTGATGTGCTTGCTCTTCCTTTTCCATATTATCTGTTCCAACCTCTGGACCA
CGACTCAAGACGTCATTAGAGGTGCGTACGTCTTCTGTGTCTGAAGCGGTC
ACATTTCTTTCACGGTTTGCTGCTGCTGCAGCTTCACATACACTTGCATCA
CGCCCAGTGTCGTCTTCTGATGGAGGAGCTTTATCCTGCTTACTTTCAGAA
TGTGATGCTCCTGGTTCTCTTTCAGCTGTACTATGTCTTGCTGTTTCCTTAG
ACACAGATTCTTGTCCCTTAGTGGGAGCTTCAGTTTTT
>LNC_011121
CCGTTTAGTTTTGTTAGTTTGCTTTAAGGGATTTCGATGACTTACTTCCGCA
TCTTTTGGAGGCATGTTCTCCCCATATAAAATCCTGGCTGCAGCAGCCTGT
ACTTTGGGATCGCAGCTGCTCTTCCTACTCTGTAAATTCCCAAGTGAACTG
GAGAATGATTCTTGGGCAGTTAGTACTGTAGATGCTGATAAACTCTTTTTG
TCCTCCTCTGAGAGCAGCTGCTTTTTCTGGAGGTGTAGGTTACGGATGGCA
GTCTCCAGCATCTGCAGCGGCACTGCCCCCGTCTCTACAGCTTTATGAAGA
AGCTGTTCGCTTTTCTTAAGATTGCCTTGAGACAGTTCAAACTGTGCGAAA
GAGACATGCACGAAAGCAAACTTCTTGCAGTTCTCTCGGGCCATTTGGAA
GTAGTCGTGAGCATCGTCAGGTTCTTGAATAGCCTTTAATTCTGCAAGTCT
CACTTGTATTCGAGCAAAGCTCTCATTCTGGCCATATTTATCTGGAGGAAG
TGCTTCAATGGCTTGACTATACCGACCAATCAGCTTATTTAAAAGAGCATC
GTTTAGAGGTGGGCTGTTTTTCTCTAGTTTCAGCAAGAAATTCAACCAGTT
CTCTGGTGTGTTCTCAACCATCATAATTT
>LNC_011165
GGCCGATGTCCTATCCAGATTAGGACCTTGTTCTTGAACAAGTTCATTAAA
CAACTGTTGTAGACTGAGAATGAGAGTCTTAGCACACTGAATCTTATCAA
TCTGCCTGGTCTTACTCAGGGTTTCCTTAATAATATCACCATAGTCATTGT
AATACTTCATGTAGTGTTTAAAGATGTCTGCAGCTGCATGCATGTCAACAA
TATCGTAAATGATAAGTTTGCTAAAGGCAGCAAGTAGGTTCCTTCTTTTAT
GTAAGGCTTCAATTTTATTAGCTTCATCTTCTTCATCGCCCTCCATGCTTTG
GTTTTCCTCGTCTTGGTCAATAAAAACATGATCCATCACAAAACTGAGGA
GTTCAGACTGCAGTCCCGTGTCTGGATTAAACACCAAAGGCTGAAGGCCT
TCTCTGCCACCTGTCATTAACTGGTGGCTAAAAATCATTAAAAGATCACAG

AGTAGCATGAAAGCCTGTTCCTTAACTGGAGTATTAACATTAGACAGGCA
CTGCTGGCAAACAGCCAAAAAAGATTTCACTGTTTTCCTCAACACCAACA
AATCCTCTTTGGAAGGAGAACCATCAGTAATTTTCACCAGCTGCCAAAGA
ATTGAGTAATGGGAACACTGCAGTGCTTGTACGACTATCTGTTCTGGCATA
GCTCCATGTTCAATTCCAGTCTTCAATAGTCTGTAGCAATTACCAAACAGA
TCCCATTTAGTGAGATCATGAGCATTGTGAAAAGAAGTTAAACGCTTTAA
GGTGGAAAGAACATTGTAGATATCATCATCATCAGCTTCTTCACCCTCTTG
CAATAGGTCTTCTACAGAATGATTGAATCGATCTACAAATTCATCAATGA
GCTGGCTCCGAGCAATATCGACTCGATTTTGAATGGTGTACTCTTCACTGC
ATAAAATGCTGTAGGTTTTACTGCAGGCTTCTAGGACATCTGACTCTACAT
GTTTCTCTACAACAAACTTAATCTGTTTTAATAAAGCATCCAGGTGCTTTT
CCATCCGACCTGTGCTGTAGATTTCCAGATCAAAATACTGTGGAATCTGCA
GCAAGTTCGCTACCTTCTCTGCATCTGCAGAATACTTTGATAGTAACATAG
GAAGTGTAATGATAAAATGTTCAGTTAATTTATTTCTATCATCAATTTGAG
TTTTCCTTTCTTTGGCTGTTAGCACTCTCTTGCCGGTTCCCCTTCCCACTGG
AGGATGTGCCTCAGCTGCTTGACGAATTGTACAGACCATTAGCTCTATAA
GAGCGCTCTCCTGACGGTCAGACATGGCTTCCTCTCCTTGAACAGGTTCTT
CTAATAGTAACTCTGTCATACATTCCCAGTCTTTCAACAGTTCTTGAGAGC
TCTCCCACAAGCTGTCCACCAAGTAGGCTGCATGTTCATGTAACTCACTTT
CAAGAAAGAAAAGAACAAGCATCCTAATGAGGTTCCCATTTGGACTGTTT
CTTCCTCTTCTCTTTGCTAATGCTTC
>LNC_011175
ACAGCATTCAACCAACCCTTCCTGTGATATCTGGCCCCTGCCAACTGTCCC
CACCCTCTGTAGCCAACGGCTTTTGGACTGGGCTGGAACCATAGCATGAA
ATCGTCTGGAGGCTAGGTGTGCACCAGGGGGGCTGTGCACACAGAGGAG
AGTCAGGAACAGTGACAGGGCGAGGTCACGAAGTGCTGGTCCAGAGGCC
CTCAGAAGTAGCAAATGTGCTCAGCCACCTGTCCTCCTGTCCCACAGGAA
GCGGTTCCTTAGAGCCCAGGCCGGGGAGGAAGGAAGCGCAGGGGTGACG
GGTGGCAACCCTGCAGTCCATGTGCCCTGCCCTGTTCCCAGGCCAGACAC
CTAGCTGTCTTCCCCATTCTCCCCGGGGCCCCGGATGAGGCGCTTGTGGCC
CCGCTTGCCTCCAGGGCCTTTGGGCTTGGCGAATGCCTGCTTGAAGGCGTG
CTGCTTGGGATGCACCTCATCATAGAGGAACTCGGCGGTGAGCTCCGCCC
CATCGCCGATCTCAATCTTCTTAGCGATCTCCAGCTGGAAGTCCTGCTCCA
CAGAGTCAAGGAGGCGGCTCTTGCAGCTGGAGTAGAGCATGCGCTCCTTA
ATGCTGCACTTGTACCCCGGCATGGAGTAGATGAACACCACAGATTCGAG
GGAGTCACCCTCATGGGTATGCTTATACAGGAAGAAGTGGTAGCGGGCAG
CATCTCGGGGTACCCGTGAGGGCAGCTGGGCCACATTTGTGGGTTCTGTGT
GTACCAGCTCGATGGTCTCCCGTTCCAGGTCCAGCTTCTGAGTAGGAGCA
GGAGAGGTGTGAGGGCGCCAAGGCACCCAGTAGGAAGCAGGATTGGTTG
GCGGGCAAGGGTGTTTGGGGTTCCTCTTTCTGGGATCCCCTTCTCAAGGCT
GGACCTCAGGCTGCAGGGGTTATTCTAGTATTTTTTTTAAAAATATTTAT
TTATTTCACATATATGAGTGCACTGTCGCTGTCTTCAGACACACCAGAAGA
GGGCATCGGATCCCATTACAGATGGTTGTGAGCCACCATGTG
>LNC_011394

GTCTTCCTCATTGAAAGCAAAGGGGTCTTCCAACTCAGTTTCCAGGTAGTG
GAGAGGAACCCTTGCCCCAGGTGGAGAGAGGGACATGCTATCCAGTCCAT
TGGGCATCTTCAAGGCCAGGGTAGGTACCGTCAGGCTAAATGCCATGGCG
TCCATCTGGTGCCTGACCTTCACCTCATCTACCGGGTCATTCTTTTTCTTCC
TCTCTTTTTTCGGGATAAAGCCGAGTACCTGCAAGTGGCTGTTGCAGTACC
TGAGGTTAACATTTTCTTCCTTGCTCCACTCCTCTAGGCATGGTTCTGACTT
TCAGGCATTGTAATCGGAGCCACATGTGCTCACCAGAAATC
>LNC_011395
CACAGACACACACACCACGCAGTAAATTACACTACAACTGTTAAAAACTT
TCATCAAAATAAACAAAGAAGGTCTAAGTAATAAATTAATTTTCTTTCTTT
TTTTTTTCTTTTTTCCGGAGCTAGGGACTGAACCCAGGGCTTTGCGGTTGCT
AGGCAAGCGCTCTACCACTGAGCTAAATCCCCAACCCCTATAAATTAATTT
TCATAAGCTGTGGTAGTAGCATTATTGATTGGGCTACCACAGGAGGATTC
ATTAAATGTCTTAGATCCTGGGAGAGGAAGGCGCTTTTGTAGGAAAATAT
AAACCTGGTAGAGGTCATAGAAAGCATGATTACTGCTCAACCTAGGAACT
CAAGTCCCTGATGGTGACTTTCAGTTCAGGATCAAGGACTCACTGGCAGG
CTCTCACTACAGCCTCCGGCAGCAACCAGTAACATTTGAGTCTGCTTTACA
ATGGAGCCAGCCCTCAGTTACTGAGCTCTCAGGAACTGGCCAAAAGTTTT
ATAAACTTTCTGATTTAACTGTGGGGTTGAACCTGTAGTTACTATAAATTT
TTATCCTCAGCTTACAAACGAAGAAACAATGTTTAAAAATGTCCAACTCG
GGGGCTGGGGATTTAGCTCAGTGGTAGAGCGCTTACCTAGCAAGCGCAAG
GCCCTGGGTTCGGTCCCCAGCTCCGAAAAAAAGAAAGAAAAAAAAATGT
CCAACTCTTCCAAAGTTAAACAAGATCTGATCTGGCAAGAAATGCTTTAG
TAAGTCAGGGA
>LNC_011403
GGACATATTTCTGCTATGTTATCATAAATTAAACCAGCACAGCTGCAGTCT
AAAGGGCTTTTAGTGTCAAGAAGTTCACACGGCACCCCCCAGCCCCACCC
CCGAGCATCTGGAAGGTACCGTCGAAGGCAACGTCACAATAGTGAGCATT
GTCCTTCTGACTCTGCACTCCTATGCCCTCCATTTCCAGGAAGACCCAAGT
GTTACTTAACCCCACAGAATGCAGGGCAGATGAGATATTTTAATGAAGCA
TGAATAAATGAATAAAATAACTAGGCAGATTCCTTCTGCTGCCATCACAC
CCGGATTCTGGAACCAGAATGAAGTGTGCCACATCAGAAGTCACTCCTGT
AGCTTGTGATGCCTCCCTTCAGGGATGGGGCCAGCCAATAAGAAGACACG
GTGCTCAGAACTGGGTGAGAAGCTTCCGGGACTTGAGGCTCTTCAAGGCA
CACAAAAAGAAAGAAGAAGTCCAGCATGCTGGGCTCTCCCATTACCAGAA
CAGAGAAACCACCAAGTGAGCTCGAAGGCCTGATTTAAAACACACTAGG
GGATTCCAGGCAAGCCGTTTCTCTTGGGATTTCTGCACGGCCAGCGGAAT
GAAGAAGCATAAGGCGAAGAAAATGGAAAAGCTGACTCATGAGAGGTTA
TGTCACTTGTCCAAGATCACACAAGAAGATACAGTAATAGCAAAATCAAC
AATCCTCAGAGCTGTCCCTGGCTTCCTGCTTACAGCTTTGGCCTCCTGATG
ATTCACGGCCCCTGGTTATATCTTAATCATGCCCTCTGTGACCCAGGGGCA
TCTGATCAGTCTTTTAAAAAAGGACAACTTCATTCTTCCAAGAGGTCTTTC
TGTAAACACCTCCACACAGCGTCTTGGCAATTTAGATGGTAGCTGTTTTTA
AGGGCAGACATATTCTGTAACCATTTTTTTTCTTATCTTCAAACTCTCATGA

AATTACCATTGAACTTGAATATGGTAAGACTGTCTTCCCCCAGGATATGGG
AAGGATACCTGGGAAGGGACAGCATTCTGGGAAGACCAATTGCTCAGTAA
CTCTTAATAAGGATTCTTGACATCTGACACCTCTGTCCATCCAGTAGAGCC
TGTGGACTCCTTCCCACAGTTGTGTTTTTAGAAGCTTAAATTAAATCCCCA
GGATTACACAGAATCCCAGGGAAACCAATTATGTTGAAATATAACTTTTC
AAAACATCAAAATGTCAGATTTTTGATGCAGTCATATATGTATTTATTAGT
AATTATATAACAAGATATAATGGCAAGTTTAGCAATTATCGTAATTCTAA
AATTGTGAGGGGAGAGTAGTATTTGAAACTATCTTCAGCTTCTTCAATGTG
ATATAAAAAACCATCTGTGACTTTATTACGGATGAATCACAGCCACTGTG
GTATGCTGCCTACAGGCGTAGTTGGAGAAAAATGACCCCTCAGTCTTGAG
GTTAGTGAAAATAACTATGCCTTTTGAAAAATCAAGTTCACAGAATGAAT
TGTATCTAAGGACCCGATATTAAGAACCCTCACTTAGAAAATGATATACT
ATTCTACCTGATAAATTGTCTCATTTATGAGTTATTAAGGATAGAAATCTG
CATGGAAGGCTTAGCTGAACTTCCAGATCATTGTACCTAAGCATTCCACTC
AAGTTTTGATTATTTTCATATGCAAAGGTACAATTAAGCAAGCGTTGATCC
CATGCCAGGAAACTGGCAAACTTGACTCCAGCCATGTATGTGCTTCCATTT
TCCTGGTGTATTAAATCTCAGAAAGGCTGGGATGGGTGGGATGCCTTGGC
CGAGGCAG
>LNC_011404
AATGGATAGATGTACCACATTTTCTGTATCCATTCGTCTGTTGAAGGGCAT
CTGGGTTCTTTCCAGCTTCTGGCTATTATAAATAAGGCTGCTATGAACATA
GTGGAGCATGTAAAACACACATTTCTAATGGTTTTAGGAACTGAAACATG
GCTCAGTAGCAAAATTACAGCTACCAAGTAGCAACGAAAGTGAATCCATG
GTTGGGAGGCTCAGGACCATCAGAAGTGCGTGGTTTTGATGGTCCTGACC
CACAAATTGAGATCTGCAGATGTAAGAGGAGGTGAGTTCAGCTTCTCAGT
GCAGAGGCAAGCGACTTCCACGGAGCCAACAGTACCAAGCTTGTCCTGCA
CCTCCATAGGGTGATGCAGCAGAGTCCTCTGGTGAGACATCGTGCACATG
CAGTTGTCCTTAGTACACTAAAATGGGATTCTAGAAAGTTCTATAAACAGT
ACCACACAGTTACTTCTCTGCTTTGCATGGAGCCACAGCGGTGACCACTCT
ATTAGACTTGAGTGTATTGGGTAGGGGAATGCTTCCTCTCAGCCCTCCCGT
ACTGCTGGCTATTCTCACGCTTTCCAAGGTTTTGTATATTCCTCGTCAGCCC
TCTACCACAAATCCCCTGTCATACTTAGGAATTCTTTGTTTTGACATCCTCT
TGTCGAAGTTATAGTTATTCAAGGCGAGGGTCTGCCTTCCCTCTCTGCAGT
CACCCAGCAGCAGCACCGTCAGGGGAGCTAACAGAAAGTCATATTCGTGA
GTAAGCCATCCCATGCAGAATGATATCCCATCCGGGAACCTCTCCACCAA
GGAGCTGCAAGAACACCTTGTGATGTGGAACCACAGACTGCGACCTATGC
CACACTTCCTACCGGCAGTAGTTTTAGTCTTACACAACTATGACTCATGAG
AACTACAAACTACCCTGCTCCATCTGTGTTAATCATAAATATGCCACAGTT
TCACTGTTCTCATTTTTATTTTGCAATAAGATTGCAAAAGCGTTCATGAGT
TCATGATTTCCCTATGTGGCCGAGGATGGCCTTGAAACTTGATCTTCCCTA
CCTCTGTTCTGGAATTGCAGATATGTGCCACCACACACCTTTGTGACCCTG
AATGGTTACAGCTTTTCAAATATCTCTCCTGGAAATAAGAATATACCCTTA
AGTTTGTTAATCTCTGTCTGTTCTTTAAAAAAAAAAAGTCCCAGGTACTGC
TCTTCCAGAGGTCCTGAGTTCAAATCCCAGCAACCACATGATGGTTCACA

ACCGTCTGTACTGAGATCTGATGCTCACTTCTGGTGTGGCTGAAGACAGCT
ATAGTGTGTGTGTGTGCGCGTGCGTGCGTGTGTGTGTGTGTGTGTGTGT
GTGTAAAATAAACCTTTAAAAAAGAAAAAAAAAAAAACCTAGGTTAGGAG
GTTGGAGGGACAGCTCAGTGGTTAAGTGTATTTACTGCTCTTCTGGAAGCC
CCAAGTGAATTCAGTTCTCATATCCACATCAGCTGGTCACACTGTCTAAAG
TTCCTGTTCACAGGGATCTGATGCATCTGGCTTCCTTGGGCGCTGCCCCCC
TGAGCATGCACCCCTCTCCCCCAACATACACAGTTTAAAATAATAATAAA
AAGGGAATCCTTAAAAAAATCAGGGGGCTGGAGCAATGGCTCAGCAGTT
AAGAGCACTGACTGCTCTTCCAGAGGACCTGAGTTCAAATCCCAGCAACC
ACATGGTGGCTCTCAACCACCTGAATAGGATCTGATGCCCTCTTCTGGTGT
GCATGAAGATACTGACAGTGTACTCACATACATAAAATAAATAAATATTT
AAAAAAATCATTAGATGATAACTGGTTTGAACATGAAGATACATTTATTTT
GGGAGTAGTTTTTTACAAAGATAAAACTATTAACATTAAAGTGATTTTCCC
CCACAGAAATCGTAAGTTCTTATGAAGTACGAACATTCTCAGGACGTTAA
GGCACGCAGGAAACACAATGGGTGGCTGTCTCCTTGGCTTAGAGCCTGGC
TTGCTTCTTCAAAGATTCCTTCACCTTCTTGATAAAAGAAGAGTCCCCAGC
TCCCACAGGCCAGTTGTCATGATCTGGTATCGTGAAGTTGCGAGTGTACA
GAGCCTGGCCT
>LNC_011423
TGGAAGTTCACACTCTGTACTTCACACTGAAACACCTCTTTGTTCCTTGCT
GCAAAAGGCACTTCCACTTTAAAGCCATTCCTGACAAACACAGCATTGGC
CACGGTCACAATGTCTTTATTCTTTTTGGAGACAATAGCCTTGTTGATCTTC
TTCAGCACTTTTCCGACTCCGTTCACATTGTATCGCATCACCGTTGAGAGC
TGCTTCTTCGTCCTGCCGTCAGCCCCCAGCTGCAGCATGCCCAAGATGGAC
GCAATCCCGTGCGGAGAAATGACAACGTTCTCATGAGGCTGTGATTTGAT
GATCTGATTGAAAACCTGGATCCCTGTGTCAGAGCCTAGTTCCTCGAGTGA
CAGAGAGTTGAGCTGGGAGTACACAGAGGATAAAGTCACTGTGGTCAAG
ATGAAGAAGGGAAAATGCCAATTCATGGTTCCTTCCAACAAGGACAGCCA
AGGAGAAGGCTGCTCCGGCTTGCTGGTGGCCTTTCCTGGCACAAGGGAGT
TGAGTAAGGGTGCTGACTTCTCCAGCTGGGGCTTAAATCCAGATCCGTCCT
CGCTTGGATCCCCAGGTGGCGGCCGGAGCCGGGAAGGAGGTCGGCGCGG
CGGTGGGGGACAGGCGGTGGTGACGCTTGCAGCCCGCGCTCCGGTGCAAC
CACTGCACTAGCCGGCCAGGCAGCGCGGGGGCTTTA
>LNC_011619
CTCCTTTATTGCCACAGGAAGCAAGTGCACTCATCTCTTCAACAGTTTTAA
GAATCACAAAAATGCAGAATTCACACAATAAAGAATAACACAGACTTAG
GGAGAAATGGTCCCTTGGGTTATATTGGCTGTAATACTGAGGTAACCAAT
TGACCTTGTTCAGTGGAAATATCTTAAACAAAATTAAAAATAAAAACCCA
ACTAGTTATAACATCTAATTTATATTTACCAAATAAAAATATATATTATTT
GCTTTAATGTATAGCAAGTTTAATACATTAGTGTTCACAAGATAAAAACTG
TATTCTACTGAAAATTTGTGTGTGTGTGTGTGTGTGTGTGTGTGTGTGT
ACGCGTGCGCGCTCTACATGAGTGTGCGTGCACACTTCTTTATCTCTAAGC
TTGGAAGTCAAAAAAGTATGCTACTTAAGGAGACTTAATTCTGGATTTAA
AAATGCAGGTTCTAGGTCCAGGAGAAACAAATATTAGCTGGAAGACCACT

GCATCTTTTAAAACTTGCGTGTTAAAATCTACAAACTTGAACTGTATGGCA
TAGTTTTTTTTTTTTTAACAAAAAGGACCACAAAATTTCTCAAAATAGATT
ATGGAACACAGGAAACAGTTCATGAGAGTACAGGGGCACTAGCCAGCAC
TGTGGCTAAGATTAGAACTAAACACTCTATTTAAATGGTATTGCACTAGCT
CTACACACAGTGGATAGTGAGACCACAGTAATCAACCTCACAGTAGCAAC
AGAGGCACGTGGTCCCTAAGATACTTCTTCAGTGGGCAAGAATCTATGGT
CCAATAGAAACATTTAGGAAGGAAAAAGCCTTGTAATAGTAGAAACGCTG
TTTACTGGTGTTTTTACTGTAAAACTCTTACAGATCAAATTGAAATGTGAT
TATCTATGAGACAGTGCTTAGAGCTTACGGTGCAGACATTTCATCGAACAT
CTGGCCAACAGATTTCAGTATAACAGATTAGGAACTCATATCTATGACAG
CATGATCTAAAACAGAACTAAAAACACACACACGCCATGTCAATTCACCA
ACATCATCATCTTTATCACATTTAGGGTTAACTTACTATGCCTTAATTTGTT
TAACACATTCTGAAATATCAATGTTTAAGTCAAGGCTTTACACAGTAACCC
ATTTATTTCAAGTCCTCTGTCCAACTTCTGTTACATCTTGAGCAGCTGTCAT
AAATGTAGCCGACCGCATCTTGGGAATGCTACTACCAATTTTTAATTTGTG
CATTGATTAAACCAATGAAAGTGACATTATTTAGTTGACAGCTACCTATTT
CCTATTGATCAACATCAATGTTATCAAGTAGCAAGCACATGGGTCTTCTCC
TTGGTGTAAACTTTGTATTTATCTTCTTTCTCCAGTCACCTTGCAAATAACT
ACATTGCCCAGGCTTTCTTTCTAGGACTGTGAAAGTTCTGAATGCTGACAA
GTAACATTTACGGTAAATACACTTAAAGTCGACAACAGAATTTCAAGTAA
AATAAGTGGGATCCACGTCTATTAGACTGCAAGGTATCAACACCTAGATT
TGCTTCTATAAATTACAGACACATTGTACATTCTTAAATAGTGAATGCCTA
CGGTATTGAGCCTTTCCACACAAGCAACTTACACAGGAAGGTAACCGGTC
GGTGCCCGTTACTTTGCAATGCTATCTCGGTTCCCTCTTCTGTGGCTGTTGC
TTTGTCCTGGTGCCCACAGACGACTGTGTGAGTTCTTACGCTTTGGCTATG
GTCTGGGAGCTGAATGAGGCAACACTGGTTATTAAGGCTTAATCTACGAT
GGAGCATATCCTCGGATCTGGTGTCATTTCCAGAGAGCAGGGGGAGAATC
TCAGCTATCTTGAGTCTTCGGGCTCTGTTTTAATAACTTTCTTTTCCAAGGT
GAATGCAGAGTGAGGATAGTCTTTTAGAATCCCAAGGCTCTCTGATGAGT
GGGACTTCACCTCGTTGGAGTAAAGTCTACTGAGCTCCCCATCCACCACA
AAATGATGGGGCTGTCTGTTCTGCACACTAGGAATTCGGAGATTCAAAGG
CCTTTCTCCTTGTCCATCGGAGCCATCAGAAGGAAGGCCGCCATCAGGGC
TCTGCTCTCCAGAGCTCTGCCTGTAGAGCCCGGCCATGAGTCTCCTCTCCT
GCTGCAGCCTCTCATAGCCGGCCTGGGCACACAGGAGCTCCATTTGCTTTG
CCATCCCCTTCTCAGCCTGTGAACTGAGACCTGCAAGCTCTTCACTTTTGG
CTCTGGCCTGCTGGAAGAGTGTTGGCACAGATTCCTGGAAGTCCGGAAAT
CCATCCTCCATTTTAATTCTCTTTGGAGACAATTCATCTCTTTCACCACATT
TTAACCTGGTGGTCCTGTAAGTGTATTTGTAGGTAACTTCTCGAGAAACCT
GCCTGGCCAGGGCGAAAAGCTCATCTCTTCTCGTCAGCAGGGCATTGTCCT
TCACACACAGCTGAGCAGCTGCCTCGTTAACCGTGAGCTCATGCAATGTG
AGGTGTTTACCATCCTTTCTTTTGGAGTCAAATCGCCCATAGATGGCGCTG
TACTTTCTGATCTCCTCCTCCTTGTGTGGATCCTCGTCGCTCATCTCGAAGA
TGTGACCAATCATTTTGGCCAACTTCTTATTGTTCTTCAGCAGCTCTTTCAC
TTCATTCAAGTCACTTTTGGGCAGCGTGGGGGCCATCCGCTCCACACACTC

GGCCACAGAGAGCGCGGCGGCAGCATCCAGGGCTTCACTGCTTTCCTTTG
GGGAGGCTGGGGAGCCCAGGTCTGCTGGGGAGAGACTATGCTCGCTTTCA
GTGGTGTGGTGTCCTTGCCAGAGTCTGGATTCACTGACACTCTGCAGTGCT
AGACTTCCCACCTCTGACTTGCCCTGACCCAAGCTCTGAACACAGGTGGTG
GCAGCGCATTTAGGGACCTTTAAATGTGGCTCTCGGGCACTGCTACTCCTT
TCATAACTATTGCAGGATATTCCCAGCCATGTTGGTGATCCCTCTGGTAAT
TTATAGATGGGTATGCTACTGACAGGAAGGGAAGTCAATGGCTGGTTGAA
AAGGCCAGGATTTGTAACCCAATCTCTCAAAGCCTTCTGAAGCCGTCTAA
CATGAAGGGGCTTGCTTGCCATGCCCACCAGTGCCATGATTTCCAAAAATT
CTTCTTCTCCAGCTTCGCACAGTTGCTGGACATCATCACCACCTTGTTGGA
TAAAGGCATCAAAGTAAGAAAGCAGATTAGCTTTCTGCAATATTCGATAC
AGCTGCAGCTCTCCCAGGGTCCTAGGTAAGGCTGTGGCCATTACTGTGGA
TGGGCTTAACGCGGCCCGCGGCTGCGGAGCGATCCGCGACAACGGCTCTG
CCGGGCTCCTGCGGCGGCTGCGGCGGCCGGGACGCCCCCGCGCCCATCGA
GGGGGGCAGGGACGTGGTGGGCGTGCGCTCTGTCCCCCGCCCGCCCCGAC
GGCGCTCGCTTGGCCGCGCGGGCGGAGGCTCGGGTGGAGGCGGGCGGCG
GCGGCCCGGGGCACCCTCCCTCCGCCTCTACCGCCGCCCCTCCCTCGGCCT
CTCCCTCTCCCTCCCCCGCCACCTCCGCCGCCCCCGCCCCGCCCCGCGCGC
TGTCACATCGGGAGATTCCGCTCTTGCTCCATTCGCCGCTCCGGCTCCCGC
CCACGCGCGGCCGAGTGACGCAGATCCCAGCGTGGGCGGCCGCGGGGCG
GGGGTGGGGGCCGGCGGGGCTGAGGCTCGGCGCGGTCCCGAGGGTGGCC
CATTCCCACGGCAGCTGAGAGCGCGCGGGTGGGGGCGC
>LNC_011643
TAAGAAGCTATGATTTACTAGTTCCCAGAAGTTAAGCTTTGGGTGGCAAG
TACCAAAAAGTTTCAGTTTTCCCAACCAAAACTGATGGATTTTACTTAGAT
GTTTGAATTTATCCAATAAACCTAAATATCCTTACTTAAAGCTGAGCAGAG
CCTACTTTAAGTTTCCGAGGCATCCTGAGGTGACAAAAACCTTGCCAAGA
AGATGTACTGCTATCTCTTGGTTTCTCCTGATGGAATGACTTGGACCAGTG
GATGGAATTCGCCTTCAAAACTGTGTCCCTCTCATGCAGGGCTTGCCATCC
ACCTGTTGTATGCAGCAGTATAAGTTAGCATGTATTAAAACAGTGCTTTAC
CAACACTGATGGAATTCATTTCTAAAGAGCAGCTCCTTAATTTTATTATCA
CGCACCTAGAAATTAAATACTGTCATGTGATACTGGCAATGGTTACCAGA
TGAGCTCAAATCCAAAACTGTATACTTTTCAAAGGTACCTGCTGCCTAAGC
TCCCAGCCTCTTGTCTTTCCCCCAGTGTATGGAATGTTGGAGAAGAAACTA
GCAAAATCTGTAAGTAGCAAATCACAGCGCCAACACATGAAGGAAGTCA
GTCCTCAAAAGATTTCTGCAGCTCCCAATTGCTACTCAATGGATCACACTC
TTGGAAAACTACTAACCCTGACCAAATCCACAAGACAACACCACCACAGA
AGCAGAGGAGGGAATCCATGCACAGCCTTTTCAGGGTCTCTTGGGGCTAT
ATGATACAGTGATGATCAAAGTTAGACTGACACCCGGCGGGGAGGGCACC
ACGGGACACACAGCTCACAGAAGACCAGAGAGCTCCATACTCCTAGCTAA
CAATCCACACTTCAGAGTTTCTTTTGGCTCACTGACTGTTAAAGCAACCTG
TCTTCAGACTGACTGACCACAAGGTGAAGTGTGGACAGGGATAGTTCAGA
AAACAGCAGTTTACAAATACTTCACTCAAATCACAAACTTTTGACATAAA
AAAACATAAATCACATAATGTACGAATATTGCTTTTGATCTTTTTTATATA

TACTTGGTTCAATGCATTTACCTATCAATTCTTACTTGGGAGAAGAACACA
GTCATGAGATTGACATTGCTGGGGAAATGTCAGCAATCCCTTTGGTCCCA
AAGCTGAAAACAGCTGAGAAAAGCAATATCTAGAAAAAGGAAACAAGAA
ACAGAGGCCACCAGAGGATCATTGGAACGTCCTGTTTGCTACTTTGAGAA
AATGTGACATTGGTATTATAAAATAGTCGTTTTCATATAAAAGATGATTTT
CTTGCAGCATCAATCATATCTCCCTTCAGAAATTATGTTTGCAGGGATCCG
GTTTTGTGGGTCTTTGTTTTGTTTTTAAACAGGTCTAATTTGAAAACAAAT
GAAAATGTGATTTTGTTCCTCCAAAAGTAAAGAAGAGACCTTTCAGGTTTC
AGCTGGTGCTTCAAGGCCAAGAAGGTGAAGCACGTCAGTGGCCTTCACGT
GCAGGGCAGAGCACTCAGGGGACCACGAGTCCTTCCAGGAGCCTTGCTGT
TTCACATCACTGGAAAGCCAAAGCACTTCACAGCCAACCGGGAGGACATG
ATACCATCACAGTCATGTGACCTGCAGGGTTCTTAATTCCCAGCACCCATA
GCTCAAGCTGAGCCCTATCCTTCAGCTTCCTCGATGGGTTCTGGTGGTGCA
TGGAGGGAAGCGGACGGCAGGACTGTACTGGTTCCTCGAGATACCCGGTC
TTGCTCTGTGTTTGTAGACAGGGAGGTCATAGAGATGGGCTGGAGCAAAG
TGGTGGTCATGCCAGTGGTGACGGTCAGGCTGTGACCAGAGCCCTTGAGG
GTAAGATCTGGTGTAGGAAGAAGTGGTTTGAGGATACTCACGAGGCAGAA
AGCAGAGCCACTTTTAGGAATAAAATTCACCTTGTTTTTGTCGGGACAGTA
AAAGGCTGGAATCTCATGAAGTTGCTTTGGCGAGCATTCCTCAAAGACTTT
GACCCTTTCACAGCCCTCTGGAGGTTCCCTTTTGAACATGTAACTGTTGTT
AGGTTTTGGTGAGGTTGCATATTTTGGCAAAGGAGAGTTGTTTCCATTGTC
TTTATCTCTGGGATCGGCAAGCAGGTCGTCAGCTGAACAGGGACTCTCCTC
ACTGCCTTCATGAGAGATGGTGAGGAAGGATGTCGGGGACACAGACTGA
GAGCGGCTGCTGTTGTTGCTTCCCTTGTCTGCACCCCCAGGAGTCTGTGTG
TCAATGCTGCGTGTGCTGCTGCTTCTCTGTGCGAGGGGTGGAGGCGCACG
ATGACCATCTGGGATATCTTGTGGAATCAGCTGTTCTTCCTTCTCCCCTGTC
TCTTTGATTATGATCTCGATCTCCTGGTTCAGCCCTTCCACACTGTTCCGGA
ACCGGGAGCCTGAGGACTTGGTGATGGGGCCAGCGGGAATGAGCGTGCTC
TTTGGAGCAGGAGCCTGGCTCTGGTTAATAGCTGCATGGTTGCCATGGAA
TGGAGACTGCCTTTCTTTATCTCGATGGTGACGGCTGCTGTGTTTGCTTCTC
TGCAGTTGCTGGCGTAATTTTGCAATCTCTTTAAGCTGTTCTGTACTGCCCC
AGGATGATGAGCGTTTATGAGACCCTTTCTTCTTTTCAGCATACTCTTCAG
CCCACGCACTCTCGGTCTGTGTGGCCTTGTCCCTCATGCAGGGTGTGGCTT
GCCCACGACTGTCTCGGGGCCAGTGCCCAGCAAGGTATGGAGCTGCAAGG
GTATCCAGGGAGGAAGTACGCCGGATAATGCTGGAGGGGCTGGAGGAAG
GTGGGCGTGTTTTGTCACCTGCTTTGCACACCGGGGCGCTGGGGCTCCTCT
TCTCCGCCGAGGGCCGACCCTGCTCTGGGGACCTCCTCCGGTGGCGAACG
CGGGCGGGGTGTGGGGCCGCACTCACCTCGCTCGGCCACAGGTGGGTGGG
CGACGAACTGGGCGACGACACGGTGCCCAGTAGGGGCGGCGGCGGCTGC
GGTCGCGGAGGACTGCTTCGGGCTCCAGGCGCCGTGCCGCGCGTGGGGCT
GGTGCCGCGCGTGGACGTCACGGACGCGCCCGTCTGCGTGGTCACCGAGG
TGCC
>LNC_011903
TGAAGTTTCTGATGCATGCATGTATCCTCCGATGTTGATCATGCAAAGTAT

TTTGATGCCAGCAATCCTACCTGTATCATGAAGCTCTGTTGATGTCAGTGA
TGTATTCTGTGTCCAGAAATAATGAAGGAGTACATTTTTCATATTGGTGAT
GGAGTCTCTGATGATGGCTAAGATATGTGTTGCTGTTCGAGCACCTGATAA
ATTCCAAGGTCCTTATTGATTCCTGAATTCATATTGAGGAAATTCTGTTGA
TGTTCCTGATGCATGCATATAGGTCCAGATGCTATATGTGCAATATATGAT
GATGCCTATGATAATATCTGAAACATAAACCTATGTAGATGTAAATGCTG
CATGATATGAGCTGCAATCATGATGGAGTCCCTTATTCCTATCGTTGATGG
CATATCATAGTTAAGGCTATGATCTGTGTTGCTGTTTGAGAATCTGGTGAA
TTTG
>LNC_011920
GTGGAAAACACATTTAATGATTGGTGTTAAATGCTGACCTTTCAGTCAGG
GTACTTAAGAACCTATCAGGAACCGCCTAAAATGTCTACATATTGGATAG
GTTTACAGAAACATGTCTAAATCCAAGACTATCCAGCAAAGTTGAAGTTG
CTCTTGAGAGGAGAATCTCATTTTATGATCTTTGATTATATTCTTTCTCCTG
CTGGATAGTTTCCTTCGAAGGAAGAGTATTCTTTTCTTCAGTATTAGTCTTC
TTCAACTTTGATTTGTCAAATGTTTCAACTTCTGATAAGTCTGGTTTATCAC
TCATCTTGACTGCTCGTTCCAAAGCTTCTATCAGGCCAGCAATTCTGTCCG
CAGCTACCTCCGGACCAGGGTGGTGTTCGCGGGTGCAGCCACTAGCTGAT
AACTCTTCAACTGGTCTGCCCCTACTCTCTGCC
>LNC_011971
CGAATTCTTTGGGATGCCCACCAGATGATTCTGGCTGGAATGGTTAGAGA
GCAGATCCTTCATTTCATCATAACTTCCAAGAGTGTTCTGGACTCGGTTGG
CAAGGGCGTCCCCTTTGTTTGTCACTGCTGCTCCAAGTCCCAATCCCTGAA
AAAGTCGAATAGATCCATAGCGGCGGGGCGGCGGCGGCTGGCGGCCGTCT
GTCCCTGCTCGCTGCCTGTCGGCGCGGCTCTGAGCTGCGCGGCTCTCTGGC
TGCGGTCGGCGGCGCGGCTCTCTGGCCCTCCTGCCCGGTTAAAAGTGCTG
GGGCCGCATTAGCGGCAGCGGCGGGAGCCCCGGGTACCTGGTCCTGCGCT
CCGGAGCAGTGGCGGCGGCGGCGGCGGAAGCGATGGCACCGGGCAGGCA
AGAGCCTGACCGCCCGCTCTGGTCCACTCGCTCCGCGGCTGCCCGCGTGCC
CAGGGCGTCCGGGCTGCGGTGGCGGCCGCTTGGAGCTGGGGCCGGCTGGG
CCGCCCGCCTGGCAGGCTGCGGGCGGGTGGCGACGGCAGAGGCGGGGGC
GAGCGAGGCACTGGCAGTGGCGCGGCGCGGCAGCCGGGGCGGCAGCGGC
GGTGGCGGCGGCTGCACAGGCG
>LNC_012086
TTCTCCTTCAGTTGTTAGCAGATGTTACAGATTCCAAGCAAGATGAAACAA
AAAGAAAAACACAGTCACTATGTTCTCTAATATCAAAATAGAGCAATTTA
TTCAAGACACTGAATATGATCTGTTGATTCTCTCTTGAAACAAATGAGCTA
CAACACACAAGGTGATTCTTTTTTTTTTCTTCTCGAAGAGCGTGTGGGGTG
AATGGGAGTGGGGGTGGGGGAGAGGACAGAGGGGGAGGGGAGTTTTGAT
GGTGCTTAACAGAATGTGGAAAATGTTGAAAGAGGACCATGTCTGGCTGT
ACGTCTGACACAGACAATAAAAACTGCACAAGCCAGAATTCAAAACTTCA
GTGAGTTGCAGAACAGACATTTATGCATTTGACAAGAAACAGAATGGAAA
GATATAACAAAGGTTTATAAAGGGAAAAGCAACAAGGAACAAAGCAGAT
CCTTACATGTGACAAAGTGCACTTCACTTGGCTCCATGGTCTATTTCTTAG

TCAGACAGAACAAAAAGTCAAGTCCTATGGTGTTGAGTGGGGTCAGAGAG
TTGAACTTTGTAATGTCCATCTGGCCCTTCGGTCCTCTACAGGCAGCATCC
TGGATGTGCTAGGAATGGTATATGGTTACTTCAAACTCAAGGAAAATTAC
CTGGAAAGGCTGCTATGTGACTACACTGCTTTCAAGGTTGAATTTGGTCAC
AAGACTAACCATTCCTCACAATGTTTTTAGGAGTTATAAACAATGTTAGGT
AACAGCATAACACATGGGCCACCCCTTGATCACAAAGATGATGCACCTGC
CCTGTAACAGCAGGCAAATGATGGCGTCCAAATGCTGGCCGCTCCAGAAA
CAGCTGCAGACTGAGCTGTCTTATGGGGATGAACTTCAGGGGTGTGGGCT
GCATTCTTCTGCTTGGTCCTTGTGTGGCCAGCACTAAGTTCCTGTCCATGTT
TAGCTGTAAGGCCAGGGAAATGAATGGTCTTCAGGTTTATGTAGATTGAA
CCTTTTTGTCCTAGTCTTACAGCCATTGACTCCAAAATAATAATTCTTATGT
GGCTTAGATTCATCTAGGGGGGGTGTAGCCTGGACACCCAGAGATTGTCT
GATGAACCCCATATGTGCCACCTACCCATTACCACCATATAGCAATGTAG
AACTTGTCAAATGAAAGTCATACCTTTACAATAGGCTGACATCTAACAGC
TGATCTTCTGTAGAGATGAACAGAAATGTATTTGTGGAACAATGATAGGA
TTTATCTGGACTTCCAAAATTACATCCTAAAGCTAAAAGCAGTCTCAGGGC
AGGGAACTTCATATCCACTGACACAGAGACATCTGCTATGATCAAATTTC
CACACAACTTGGGCTTACTTTGCTAAGCATTTAGGTGGCAGAACCCAAATT
CACCACTCAACTCAATACACAACACTTTAGATTTTGAGGGCTCACATAATT
TACATGGCAAAGTGGAGGCCAATACATGTTTTGGTGCTAACATTTGTTGTG
TAGGGAGCACCTGACCTTCCTTGCTGGTTGATTTTTGGCTTCATATATGTA
TACTTTTCTCTAAGTAACACTTGCCTCAGCTGCAGACTGGACCCTTCATAC
TGCCTCTTGCAGCCCCCTTCTGGTCTGTCCCCTATTACTAATTAAAGAAAC
AAGTCAGTGTCAAGTTATGACAAGAAATGAACAAAGGCCACCTGATTCAA
TGATGGCAAACTTTACCAGGCACACTATGCCTTATTTTGTTTTTAAAAAAT
AGCCGAAATTTCTTTCCTATTTTTTTCTCATTTTTTAAAAGAAAGCTTTGTC
CTAATCTTCTGTCAGAGAAATTGGAAAGTGGGCAGAAATGATCAGTGACA
TTCTACCTGGGTCATAATTCATTTTTACAATTGAAAGTCTCCCTTGAGACT
CACTGTCTTCACAAGACTGAGTGACACTGAGGCCTTTGGGTCAGTTTTGCA
AGATTCACCAAGCATGCTCTTGATAAAAGTGCCCATCTGTAGCTACACTGT
TGTGAGAAAGATGTAGCTGGCAGGTGCCATGAGCCACACCTGGTCAGGGT
GGCTTCTTCACTATGTTCCTATTGGCTGCTGAAGGAAATGTGTACCAAACA
GTTCAGCATTCTGCTTGATGCCTAACCAGTGGGCTCTTTCTTCCCCACCAG
CTGCCAAAACAATCCCCTTGTTCTTCCATAGGACGCTTCCCAATCCACGGC
TTGCCTAACTGCCTTTCGTGAAGCTGAAGGGAAAGGGGTAATTAGACACC
TCAGACTCTGCCAGTGTCTTCTTAGTCTTTTAAATATGAATTTCACAAAAC
TCTCTATGTCTGATGGCCATTTTAGGATTTTAAAGTCATTTTAACATAAAA
ATATTTTTTTCAAAAAATACTCCAGGTTCTTTCTCTTATAAGTAAGTCTTTT
TTTTTCTGTAAAAAGTCCCCATCCCTGCCTTCTTACATAAAGCACTTACTAT
GCTCTGAAAGTGCCTTTGGGACCTAATGTGAAGCAACTATGAGAAAGGGA
CAGAGTAAGCAATAGGTAGGAGTGGGAGAGAGGGAAGGAGAGTGCTGTC
CAACAAAGATCCACTGTGTGCATGTGGCCATATCTATGCAGGATTTCATTA
GAAGTGAGACCACACCAGAGCAAGGACATCAGAGGAAGGGGTGGGAAAC
AACAAGGGAGGACAGAAGGAATAAGCCAAAAACAGTTTTCATCTGAGGT

GGGAAGCCCCCAGACCACAGAATTTTAGCAGATAGAAAAACCACCAAAG
AAGGTTCTAGAAAATACAGCGTTGGGATTACAAAGTCACACATTTCATTG
GTACAAACTGGTCTTTTGGAGCAACTGTAGTATCCAAATTGATAGGAGGG
AAACACCATAAGGGACAGGTTTTTCCTCCCTTTTGGTTTATCACAGCATTT
TTTCTCTTTCATTTTTTAACCTTTTCATTATTTTTTGGCACAGCTTTGTGTTC
TTCCTTCCCTGTTCAGTCATTAGAGCCTGTTCTGGATGGAAACCAACCATG
TACCATGTAGCTGATGGCTTGCTTCATCCTTCCCCAGCTGAGCATTCTAGG
TAGAAACCATATTCCTGTGATACAACCATGCGTATGACTTTGTATTTGTTT
GTGTTTTAAACTTCTGGATGGAGATTAGGGGAGGGGGGGAAATAATCCTG
TGGCCAGGGACAGGTGATTTCCAAGTAGACTCGAACAAGTCAAGTTCACA
CAGTTTGGAGCCCATCATAGGAAAGGGTGCTGCAATCCCACACATTCACT
GCTGCTCCATTAGAATGGTTTTGTTTTCTACATTTTGTGTTTACTTTCGGAG
ATCTAAAACACAGACAGAGCCATCAGATGCACTGGCACCCACTTTGTCTC
CTCGAGCATTCCAGCACACCTCAAATATGCCGCCTGTTCCTCGGTAGCTGT
GGACGAGACTTCCACTCTGAGTATTCCAGATATGGACACACTTGTCAAAG
GATCCACTGGCCAAATACTTTCCGTCAGGGCTGAACGCTACACTATAGAC
AGGCTCCTGATGCTTGGTCAGTGTGTGGATGCAAACGCCTCGCTCCACATC
CCACAGTCGAACCGTAGAGTCAAATGAAGCACTTGCTAACATGATGTTGG
AGTTCGGGTTGCTGGTGGCTGGTCCTGTGGGACTCCACTTGATGGTATATA
TCTCTTTGCTGTGAGCTTGGAGGTCATGGACACATGCATCCTGCTTCATAC
TCCAGATCTTTAATGTCATGTCATCAGAGCAGGAGGCTAGCAACATTCCA
GAAGGATCCCATTTGATAGCATTGACCTCATTAGTATGTCCTTGGAAGGTT
TTGACTGGGCGATCACAGCCAAGCCTGCACACATGAATGCACATGTCTGT
GCTGCACGAGGCAAAGGTAGTGTTGTTCTGCCAGTCCACATCAAGGGCAG
GTGCTGAATGAAAAGGAAACTGTTGTTTGGCTTCTCCTGTGTGGGCGTCCC
AAATTATTGTTGTCTTGTCTACACCAGCACTCAAAATATAATTCCCCTTTTT
GTTCCACTTCAAGGCAAAGATGGGGCCTTTGTGTTGACCCAAGGTGCTGG
CTAGGTTACCATCTTCTGTCCATATTCTTGCAAAACCATCATAGGAACCTG
TTGCCAACAGTGTCCCATCGCTATTCCAGTCCAATGAGGTGACATCCTTAT
TACTGGGAACATCATGTCCACCTTCACGGATGCAGTGCCTCAGCACAAGC
TGTGTGGAGCCCCCGTTGCTGTTCTCATTAAGGTTCCATATCCTCGCAGTA
GAGTCTCCAGATCCAGAAGCAAGTAGGTCACTAACAGGATTCCAGGCACA
AATGAACACTTCAGACTCATGGCCTCGAAGGACTGTGGCTTTACTCGGTG
GAATTTCAACGTCTCCATCTATTTCCATTGGTTTTGAGTGATTATTTATTGC
ATGGGCTCCATTTTCTTCCCCATTCACGGTGGCTTCTCCATTCTTTGGTGGG
TTTTGCTGAGCAGCAGCTGCTGGTGTTGTGGCCGCTGTGCTTGTGGCTGTG
GCTGCGGCTGCTGCTGCTGCAGCTGCTGCATTGGCTTGCTGTTGGGCGAGC
TTCTCCCGAAAAGCTTGCTGTCTTGTCTGTACCACATCGGGCATCACAGCA
TCAATTAACGATAGGGACTCAATGGGACGCCCATCAAATACCGTGCCATC
CTCGTTGATGCTGATCTCCGCCTCCACATATTGCAGTCCTTTCTGAAGAAT
GGAGATTAGGGCAGCCGGTGGCACTAGTGTACCATTGATGTTGGACTGGC
TAATGTGGCTTTCGATCCCAAACGTGAAGGCGGAATGGGAAAAACCTGAT
TCCTGGAGGTAGCGATATACCAGAAAGTTCACCTCGTCGCTGGTAATGCT
CATCTTAGCCTCACCTTGCGGCGATGAGGTCTTCGTCTCGATCAAGTGTGA

GCCACCCTCGTCACAGTGAGTGCATGTGTAGACCTCTGGAGCAGTGGGCT
ACCCAGCATCCAATCACAGAACTTGAGAGGTGTGCACAAAGTCCAGGACC
ACCCTCGCTGCTAGCCGTGCAACTTGTGGCCGCGCAAAGTGGCCGCGCAC
TCAGCGTACTGGCGGGTCCACCGTCACCAACCCCGGGCCGCGCGGAGGCC
GACGAGGGCCAAGCGCCCCGGAGCAGCCGCGCGCTGCCCCAGGCCCGCC
ACCTAGTCCACGAGAAGAGCGGGCGCTGGAAAGTTTCGGTGGCGAATGGC
GGCCGGAGGGCGCTGCAGCGGCGGCGGGGCGGGGGCGCTGGCGGCTTGT
CGCTCCGGAGCGTCGAGACTGGGGGGGCTGGGCTGGGAGCGGCGGCGGC
GGGAGAGGGCGGGACGGCGGGAGGGACCTACACGGCCAGTGACCTCCG
>LNC_012218
ATAATTTCCTAGATATCAATCGCTCAGAAGTATCTTTGTTGTCTGTAACAT
AAAGTGCTACAGTCCTTCAAATAAAATTATAGACTAATTGAAAATATATA
TTATACATTTGCAGTTATTTATAATCATTCCATAATTTGTAGTGTGTTAATG
GAGTGGAAGAGATAAAAGGCAGTCCACATTCATATTTTAAGATAAATTTA
CTTCAGAAGTCAGTTTTAATCCTTGTTCATGTTCTAAGTATAGCCATTGTG
ATAAGACCTAAGATATAAGCAGCCACTAATTTTTGATTCACACTTAAGTG
GAGGTAGAGAGGCACCAGCGATTCCATATCCCCTAGAGTAGGCAGCATCA
GGGCCGCAATACATACCACTCCCAGGAAGACAGTTAGCAAACTAGGTCCT
GAAGAGACTGCTCTGTTTTCTGGTTGACAGGACTGCTCAAAAAATACACT
CTCGGTTAAGTGCTCCTTTATTCTCTTCTCCTCTTCTTCCTTTTGTTGTTCTT
TCGCAGATGGGAGAAGAGTAGCAATAACCTCTTTCCTTCCTAAGGCTTTCC
TCTGGCTTTGTTCCGAAACCTGGAGACGGAATTTGTCTATCACACCATAGT
GACAGGATCGAACATCTCTATGACGAATGACAATATCCACACAGCACTGT
GGCTTCACTGCACCAGCAGCATCAATGACAACATACTTATTTGGAGTCGT
ACACAAAACTTTGAACTTTAAGGCAAACTCATAGGGATTGTACAGAGTCA
ACACTTGCTTATGTGTTGACTGATCATCTGCATAAAATATTAGCTCCGTGG
GAAACACAAAGACAGGAAGATTCCCTTCCACTAACTCTGGTTGTCTTTTTT
GTTGATGCATTGGCACTGTATCCCCCTTGCTGCAGCTTCTGTCTTTACAGTC
TCAAGTCTATTTTGGACTTTCTTAGATTCAGTTAAAAACTCCAAAGCATTT
TGGCAATCTCAGCAACACTGGCTCCTCGGAAAGCAGCTGCGACTGCTCCC
TGCTCCTTTGTCAGA
>LNC_012220
ATTAAAATAGACGGCTCTAAAAAAAATGGATTTCTCCCCTGTCCATAATTG
TCTATTCCAGATATTAATCTATTGAGATTTAACAAATCAAAGGAGGATCTT
AGAGCCTGACCAAGAGTAGTCAGTCCAGAAGCCTGGAGATTTTTCAGTTC
GCTCATGAATGTGGCATGATTTTCCTTCCAACCAGCCTTGATGCAGTAGGG
AGGCTCGTCGTAGGTGACCAGCATGTACCTGTCTCCGCGGCTGGCCGGGT
CCCGGGCGCGCAGCTTCAAGAATAACTCCACAGCGCCTTTGGCAATGTCC
AAATAGGAGGTGCCCAGGTCAGTGCGCTGGTTCATGGAGGCGGACGTATC
TATGAGGAACAGCAGGATGGGCATCTTCCACTACACCTCTGGTTCTGGTCC
CCACTTTCCCGGCCCCTTTGCCCTCAATGCCACGTATGGCTACCCAAATCC
TGAACTCTCAC
>ENSRNOT00000084332
ATGTAAGGGCAGTTGTATATGACATATGTCCATTTGCTGATCACCAAAGTT

GACATGGCTGACCTGGGTGGCTAGGTGGATGCTTAATTACCTCGCCACTC
AGCATGTTTTCTTCCTGAAACTCTCTGCACAAGTAGAGGAGTACCCTTCTT
TGTTCAAGAGTTTATCAATAGCTTCACTGCTCCATGGAAACCTCCAAACAA
GCGCACAAGGAAGCTATTTCAATATTTGACGCAAAAGGGTACAATTAT
